# The 2023 report of the *Lancet* Countdown on health and climate change: the imperative for a health-centred response in a world facing irreversible harms

**DOI:** 10.1016/S0140-6736(23)01859-7

**Published:** 2023-11-14

**Authors:** Marina Romanello, Claudia di Napoli, Carole Green, Harry Kennard, Pete Lampard, Daniel Scamman, Maria Walawender, Zakari Ali, Nadia Ameli, Sonja Ayeb-Karlsson, Paul J Beggs, Kristine Belesova, Lea Berrang Ford, Kathryn Bowen, Wenjia Cai, Max Callaghan, Diarmid Campbell-Lendrum, Jonathan Chambers, Troy J Cross, Kim R van Daalen, Carole Dalin, Niheer Dasandi, Shouro Dasgupta, Michael Davies, Paula Dominguez-Salas, Robert Dubrow, Kristie L Ebi, Matthew Eckelman, Paul Ekins, Chris Freyberg, Olga Gasparyan, Georgiana Gordon-Strachan, Hilary Graham, Samuel H Gunther, Ian Hamilton, Yun Hang, Risto Hänninen, Stella Hartinger, Kehan He, Julian Heidecke, Jeremy J Hess, Shih-Che Hsu, Louis Jamart, Slava Jankin, Ollie Jay, Ilan Kelman, Gregor Kiesewetter, Patrick Kinney, Dominic Kniveton, Rostislav Kouznetsov, Francesca Larosa, Jason K W Lee, Bruno Lemke, Yang Liu, Zhao Liu, Melissa Lott, Martín Lotto Batista, Rachel Lowe, Maquins Odhiambo Sewe, Jaime Martinez-Urtaza, Mark Maslin, Lucy McAllister, Celia McMichael, Zhifu Mi, James Milner, Kelton Minor, Jan C Minx, Nahid Mohajeri, Natalie C Momen, Maziar Moradi-Lakeh, Karyn Morrissey, Simon Munzert, Kris A Murray, Tara Neville, Maria Nilsson, Nick Obradovich, Megan B O’Hare, Camile Oliveira, Tadj Oreszczyn, Matthias Otto, Fereidoon Owfi, Olivia Pearman, Frank Pega, Andrew Pershing, Mahnaz Rabbaniha, Jamie Rickman, Elizabeth J Z Robinson, Joacim Rocklöv, Renee N Salas, Jan C Semenza, Jodi D Sherman, Joy Shumake-Guillemot, Grant Silbert, Mikhail Sofiev, Marco Springmann, Jennifer D Stowell, Meisam Tabatabaei, Jonathon Taylor, Ross Thompson, Cathryn Tonne, Marina Treskova, Joaquin A Trinanes, Fabian Wagner, Laura Warnecke, Hannah Whitcombe, Matthew Winning, Arthur Wyns, Marisol Yglesias-González, Shihui Zhang, Ying Zhang, Qiao Zhu, Peng Gong, Hugh Montgomery, Anthony Costello

**Affiliations:** Institute for Global Health, University College London, London, UK; School of Agriculture, Policy and Development, University of Reading, Reading, UK; Department of Global Health, University of Washington, Washington, DC, USA; Center on Global Energy Policy, Columbia University, New York, NY, USA; Department of Health Sciences, University of York, York, UK; Institute for Sustainable Resources, University College London, London, UK; Institute for Global Health, University College London, London, UK; Medical Research Council Unit The Gambia, London School of Hygiene and Tropical Medicine, London, UK; Institute for Sustainable Resources, University College London, London, UK; Institute for Risk and Disaster Reduction, University College London, London, UK; School of Natural Sciences, Macquarie University, Sydney, NSW, Australia; School of Public Health, Imperial College London, London, UK; School of Earth and Environment, University of Leeds, Leeds, UK; School of Population and Global Health, The University of Melbourne, Melbourne, VIC, Australia; Department of Earth System Science, Tsinghua University, Beijing, China; Mercator Research Institute on Global Commons and Climate Change, Berlin, Germany; Department of Environment, Climate Change and Health, World Health Organisation, Geneva, Switzerland; Institute for Environmental Sciences, University of Geneva, Geneva, Switzerland; Heat and Health Research Incubator, University of Sydney, Sydney, NSW, Australia; Barcelona Supercomputing Center, Barcelona, Spain; Institute for Sustainable Resources, University College London, London, UK; International Development Department, University of Birmingham, Birmingham, UK; Euro-Mediterranean Center on Climate Change Foundation, Lecce, Italy; Institute for Risk and Disaster Reduction, University College London, London, UK; Natural Resources Institute, University of Greenwich, London, UK; School of Public Health, Yale University, New Haven, CT, USA; Department of Global Health, University of Washington, Washington, DC, USA; Department of Civil & Environmental Engineering, Northeastern University, Boston, MA, USA; Institute for Sustainable Resources, University College London, London, UK; Department of Information Systems, Massey University, Palmerston North, New Zealand; Department of Political Science, Florida State University, Tallahassee, FL, USA; Tropical Metabolism Research Unit, University of the West Indies, Mona, Jamaica; Department of Health Sciences, University of York, York, UK; Yong Loo Lin School of Medicine, National University of Singapore, Singapore; Energy Institute, University College London, London, UK; Gangarosa Department of Environmental Health, Emory University, Atlanta, GA; Finnish Meteorological Institute, Helsinki, Finland; Carlos Vidal Layseca School of Public Health and Management, Cayetano Heredia Pervuvian University, Lima, Peru; Bartlett School of Sustainable Construction, University College London, London, UK; Interdisciplinary Center for Scientific Computing, Heidelberg University, Heidelberg, Germany; Centre for Health and the Global Environment, University of Washington, Washington, DC, USA; Energy Institute, University College London, London, UK; Institute for Global Health, University College London, London, UK; Centre for AI in Government, University of Birmingham, Birmingham, UK; Heat and Health Research Incubator, University of Sydney, Sydney, NSW, Australia; Institute for Global Health, University College London, London, UK; International Institute for Applied Systems Analysis Energy, Climate, and Environment Program, Laxenburg, Austria; Department of Environmental Health, Boston University, Boston, MA, USA; School of Global Studies, University of Sussex, Brighton and Hove, UK; Finnish Meteorological Institute, Helsinki, Finland; Engineering Mechanics, KTH Royal Institute of Technology, Stockholm, Sweden; Yong Loo Lin School of Medicine, National University of Singapore, Singapore; School of Health, Nelson Marlborough Institute of Technology, Nelson, New Zealand; Gangarosa Department of Environmental Health, Emory University, Atlanta, GA; Department of Earth System Science, Tsinghua University, Beijing, China; Center on Global Energy Policy, Columbia University, New York, NY, USA; Barcelona Supercomputing Center, Barcelona, Spain; Catalan Institution for Research and Advanced Studies, Barcelona, Spain; Department of Public Health and Clinical Medicine, Umeå University, Umeå, Sweden; Department of Genetics and Microbiology, Autonomous University of Barcelona, Bellaterra, Spain; Department of Geography, University College London, London, UK; Environmental Studies Program, Denison University, Granville, OH, USA; School of Geography, Earth and Atmospheric Sciences, The University of Melbourne, Melbourne, VIC, Australia; Bartlett School of Sustainable Construction, University College London, London, UK; Department of Public Health Environments and Society, London School of Hygiene and Tropical Medicine, London, UK; Data Science Institute, Columbia University, New York, NY, USA; Mercator Research Institute on Global Commons and Climate Change, Berlin, Germany; Bartlett School of Sustainable Construction, University College London, London, UK; Department of Environment, Climate Change and Health, World Health Organisation, Geneva, Switzerland; Preventive Medicine and Public Health Research Center, Psychosocial Health Research Institute, Department of Community and Family Medicine, Iran University of Medical Sciences, Tehran, Iran; Department of Technology Management and Economics, Technical University of Denmark, Kongens Lyngby, Denmark; Hertie School, Berlin, Germany; Medical Research Council Unit The Gambia, London School of Hygiene and Tropical Medicine, London, UK; Department of Environment, Climate Change and Health, World Health Organisation, Geneva, Switzerland; Department for Epidemiology and Global Health, Umeå University, Umeå, Sweden; Laureate Institute for Brain Research, Tulsa, OK, USA; Institute for Global Health, University College London, London, UK; Institute for Global Health, University College London, London, UK; Energy Institute, University College London, London, UK; School of Health, Nelson Marlborough Institute of Technology, Nelson, New Zealand; Iranian Fisheries Science Research Institute, Tehran, Iran; Center for Science and Technology Policy, University of Colorado Boulder, Boulder, CO, USA; Department of Environment, Climate Change and Health, World Health Organisation, Geneva, Switzerland; Climate Central, Princeton, NJ, USA; Iranian Fisheries Science Research Institute, Tehran, Iran; Institute for Sustainable Resources, University College London, London, UK; Grantham Research Institute on Climate Change and the Environment, London School of Economics and Political Science, London, UK; Interdisciplinary Center for Scientific Computing, Heidelberg University, Heidelberg, Germany; Harvard Medical School, Harvard University, Boston, MA, USA; Department of Public Health and Clinical Medicine, Umeå University, Umeå, Sweden; Department of Anesthesiology, Yale University, New Haven, CT, USA; World Meteorological Organization, Geneva, Switzerland; Melbourne Medical School, The University of Melbourne, Melbourne, VIC, Australia; Finnish Meteorological Institute, Helsinki, Finland; Faculty of Epidemiology and Population Health, London School of Hygiene and Tropical Medicine, London, UK; Department of Environmental Health, Boston University, Boston, MA, USA; Institute of Tropical Aquaculture and Fisheries, Universiti Malaysia Terengganu, Terengganu, Malaysia; Department of Civil Engineering, Tampere University, Tampere, Finland; UK Health Security Agency, London, UK; Barcelona Institute for Global Health, Barcelona, Spain; Interdisciplinary Center for Scientific Computing, Heidelberg University, Heidelberg, Germany; Department of Electronics and Computer Science, University of Santiago de Compostela, Santiago, Spain; International Institute for Applied Systems Analysis Energy, Climate, and Environment Program, Laxenburg, Austria; International Institute for Applied Systems Analysis Energy, Climate, and Environment Program, Laxenburg, Austria; Institute for Global Health, University College London, London, UK; Institute for Sustainable Resources, University College London, London, UK; Melbourne Climate Futures, The University of Melbourne, Melbourne, VIC, Australia; Centro Latinoamericano de Excelencia en Cambio Climaticoy Salud Cayetano Heredia Pervuvian University, Lima, Peru; Department of Earth System Science, Tsinghua University, Beijing, China; School of Public Health, University of Sydney, Sydney, NSW, Australia; Gangarosa Department of Environmental Health, Emory University, Atlanta, GA; Department of Geography, University of Hong Kong, Hong Kong Special Administrative Region, China; Department of Experimental and Translational Medicine and Division of Medicine University College London, London, UK; Institute for Global Health, University College London, London, UK

## Abstract

The *Lancet* Countdown is an international research collaboration that independently monitors the evolving impacts of climate change on health, and the emerging health opportunities of climate action. In its eighth iteration, this 2023 report draws on the expertise of 114 scientists and health practitioners from 52 research institutions and UN agencies worldwide to provide its most comprehensive assessment yet.

In 2022, the *Lancet* Countdown warned that people’s health is at the mercy of fossil fuels and stressed the transformative opportunity of jointly tackling the concurrent climate change, energy, cost-of-living, and health crises for human health and wellbeing. This year’s report finds few signs of such progress. At the current 10-year mean heating of 1·14°C above pre-industrial levels, climate change is increasingly impacting the health and survival of people worldwide, and projections show these risks could worsen steeply with further inaction. However, with health matters gaining prominence in climate change negotiations, this report highlights new opportunities to deliver health-promoting climate change action and a safe and thriving future for all.

**The rising health toll of a changing climate:**

In 2023, the world saw the highest global temperatures in over 100 000 years, and heat records were broken in all continents through 2022. Adults older than 65 years and infants younger than 1 year, for whom extreme heat can be particularly life-threatening, are now exposed to twice as many heatwave days as they would have experienced in 1986–2005 (indicator 1.1.2). Harnessing the rapidly advancing science of detection and attribution, new analysis shows that over 60% of the days that reached health-threatening high temperatures in 2020 were made more than twice as likely to occur due to anthropogenic climate change (indicator 1.1.5); and heat-related deaths of people older than 65 years increased by 85% compared with 1990–2000, substantially higher than the 38% increase that would have been expected had temperatures not changed (indicator 1.1.5).

Simultaneously, climate change is damaging the natural and human systems on which people rely for good health. The global land area affected by extreme drought increased from 18% in 1951–60 to 47% in 2013–22 (indicator 1.2.2), jeopardising water security, sanitation, and food production. A higher frequency of heatwaves and droughts in 2021 was associated with 127 million more people experiencing moderate or severe food insecurity compared with 1981–2010 (indicator 1.4), putting millions of people at risk of malnutrition and potentially irreversible health effects. The changing climatic conditions are also putting more populations at risk of life-threatening infectious diseases, such as dengue, malaria, vibriosis, and West Nile virus (indicator 1.3).

Compounding these direct health impacts, the economic losses associated with global heating increasingly harm livelihoods, limit resilience, and restrict the funds available to tackle climate change. Economic losses from extreme weather events increased by 23% between 2010–14 and 2018–22, amounting to US$264 billion in 2022 alone (indicator 4.1.1), whereas heat exposure led to global potential income losses worth $863 billion (indicators 1.1.4 and 4.1.3). Labour capacity loss resulting from heat exposure affected low and medium Human Development Index (HDI) countries the most, exacerbating global inequities, with potential income losses equivalent to 6·1% and 3·8% of their gross domestic product (GDP), respectively (indicator 4.1.3).

The multiple and simultaneously rising risks of climate change are amplifying global health inequities and threatening the very foundations of human health. Health systems are increasingly strained, and 27% of surveyed cities declared concerns over their health systems being overwhelmed by the impacts of climate change (indicator 2.1.3). Often due to scarce financial resources and low technical and human capacity, the countries most vulnerable to climate impacts also face the most challenges in achieving adaptation progress, reflecting the human risks of an unjust transition. Only 44% of low HDI countries and 54% of medium HDI countries reported high implementation of health emergency management capacities in 2022, compared with 85% of very high HDI countries (indicator 2.2.5). Additionally, low and medium HDI countries had the highest proportion of cities not intending to undertake a climate change risk assessment in 2021 (12%; indicator 2.1.3). These inequalities are aggravated by the persistent failure of the wealthiest countries to deliver the promised modest annual sum of $100 billion to support climate action in those countries defined as developing within the UN Framework Convention on Climate Change. Consequently, those countries that have historically contributed the least to climate change are bearing the brunt of its health impacts—both a reflection and a direct consequence of the structural inequities that lie within the root causes of climate change.

**The human costs of persistent inaction:**

The growing threats experienced to date are early signs and symptoms of what a rapidly changing climate could mean for the health of the world’s populations. With 1337 tonnes of CO_2_ emitted each second, each moment of delay worsens the risks to people’s health and survival.

In this year’s report, new projections reveal the dangers of further delays in action, with every tracked health dimension worsening as the climate changes. If global mean temperature continues to rise to just under 2°C, annual heat-related deaths are projected to increase by 370% by midcentury, assuming no substantial progress on adaptation (indicator 1.1.5). Under such a scenario, heat-related labour loss is projected to increase by 50% (indicator 1.1.4), and heatwaves alone could lead to 524·9 million additional people experiencing moderate-to-severe food insecurity by 2041–60, aggravating the global risk of malnutrition. Life-threatening infectious diseases are also projected to spread further, with the length of coastline suitable for *Vibrio* pathogens expanding by 17–25%, and the transmission potential for dengue increasing by 36–37% by midcentury. As risks rise, so will the costs and challenges of adaptation. These estimates provide some indication of what the future could hold. However, poor accounting for non-linear responses, tipping points, and cascading and synergistic interactions could render these projections conservative, disproportionately increasing the threat to the health of populations worldwide.

**A world accelerating in the wrong direction:**

The health risks of a 2°C hotter world underscore the health imperative of accelerating climate change action. With limits to adaptation drawing closer, ambitious mitigation is paramount to keep the magnitude of health hazards within the limits of the capacity of health systems to adapt. Yet years of scientific warnings of the threat to people’s lives have been met with grossly insufficient action, and policies to date have put the world on track to almost 3°C of heating.

The 2022 *Lancet* Countdown report highlighted the opportunity to accelerate the transition away from health-harming fossil fuels in response to the global energy crisis. However, data this year show a world that is often moving in the wrong direction. Energy-related CO_2_ emissions increased by 0·9% to a record 36·8 Gt in 2022 (indicator 3.1.1), and still only 9·5% of global electricity comes from modern renewables (mainly solar and wind energy), despite their costs falling below that of fossil fuels. Concerningly, driven partly by record profits, oil and gas companies are further reducing their compliance with the Paris Agreement: the strategies of the world’s 20 largest oil and gas companies as of early 2023 will result in emissions surpassing levels consistent with the Paris Agreement goals by 173% in 2040—an increase of 61% from 2022 (indicator 4.2.6). Rather than pursuing accelerated development of renewable energy, fossil fuel companies allocated only 4% of their capital investment to renewables in 2022.

Meanwhile, global fossil fuel investment increased by 10% in 2022, reaching over $1 trillion (indicator 4.2.1). The expansion of oil and gas extractive activities has been supported through both private and public financial flows. Across 2017–21, the 40 banks that lend most to the fossil fuel sector collectively invested $489 billion annually in fossil fuels (annual average), with 52% increasing their lending from 2010–16. Simultaneously, in 2020, 78% of the countries assessed, responsible for 93% of all global CO_2_ emissions, still provided net direct fossil fuels subsidies totalling $305 billion, further hindering fossil fuel phase-out (indicator 4.2.4). Without a rapid response to course correct, the persistent use and expansion of fossil fuels will ensure an increasingly inequitable future that threatens the lives of billions of people alive today.

**The opportunity to deliver a healthy future for all:**

Despite the challenges, data also expose the transformative health benefits that could come from the transition to a zero-carbon future, with health professionals playing a crucial role in ensuring these gains are maximised. Globally, 775 million people still live without electricity, and close to 1 billion people are still served by health-care facilities without reliable energy. With structural global inequities in the development of, access to, and use of clean energy, only 2·3% of electricity in low HDI countries comes from modern renewables (against 11% in very high HDI countries), and 92% of households in low HDI countries still rely on biomass fuels to meet their energy needs (against 7·5% in very high HDI countries; indicators 3.1.1 and 3.1.2). In this context, the transition to renewables can enable access to decentralised clean energy and, coupled with interventions to increase energy efficiency, can reduce energy poverty and power high quality health-supportive services. By reducing the burning of dirty fuels (including fossil fuels and biomass), such interventions could help avoid a large proportion of the 1·9 million deaths that occur annually from dirty-fuel-derived, outdoor, airborne, fine particulate matter pollution (PM_2·5_; indicator 3.2.1), and a large proportion of the 78 deaths per 100 000 people associated with exposure to indoor air pollution (indicator 3.2.2). Additionally, the just development of renewable energy markets can generate net employment opportunities with safer, more locally available jobs. Ensuring countries, particularly those facing high levels of energy poverty, are supported in the safe development, deployment, and adoption of renewable energy is key to maximising health gains and preventing unjust extractive industrial practices that can harm the health and livelihoods of local populations and widen health inequities.

With fossil fuels accounting for 95% of road transport energy (indicator 3.1.3), interventions to enable and promote safe active travel and zero-emission public transport can further deliver emissions reduction, promote health through physical activity, and avert many of the 460 000 deaths caused annually by transport-derived PM_2·5_ pollution (indicator 3.2.1), and some of the 3·2 million annual deaths related to physical inactivity. People-centred, climate-resilient urban redesign to improve building energy efficiency, increase green and blue spaces, and promote sustainable cooling, can additionally prevent heat-related health harms, avoid air-conditioning-derived emissions (indicator 2.2.2), and provide direct physical and mental health benefits.

Additionally, food systems are responsible for 30% of global greenhouse gas (GHG) emissions, with 57% of agricultural emissions in 2020 being derived from the production of red meat and milk (indicator 3.3.1). Promoting and enabling equitable access to affordable, healthy, low-carbon diets that meet local nutritional and cultural requirements can contribute to mitigation, while preventing many of the 12·2 million deaths attributable to suboptimal diets (indicator 3.3.2).

The health community could play a central role in securing these benefits, by delivering public health interventions to reduce air pollution, enabling and supporting active travel and healthier diets, and promoting improvements in the environmental conditions and commercial activities that define health outcomes. Importantly, the health sector can lead by example and transition to sustainable, resource-efficient, net-zero emission health systems, thereby preventing its 4·6% contribution to global GHG emissions, with cascading impacts ultimately affecting the broader economy (indicator 3.4).

Some encouraging signs of progress offer a glimpse of the enormous human benefits that health-centred action could render. Deaths attributable to fossil-fuel-derived air pollution have decreased by 15·7% since 2005, with 80% of this reduction being the result of reduced coal-derived pollution. Meanwhile the renewable energy sector expanded to a historical high of 12·7 million employees in 2021 (indicator 4.2.2); and renewable energy accounted for 90% of the growth in electricity capacity in 2022 (indicator 3.1.1). Supporting this, global clean energy investment increased by 15% in 2022, to $1·6 trillion, exceeding fossil fuel investment by 61% (indicator 4.2.1); and lending to the green energy sector rose to $498 billion in 2021, approaching fossil fuel lending (indicator 4.2.7).

Scientific understanding of the links between health and climate change is rapidly growing, and although coverage lags in some of the most affected regions, over 3000 scientific articles covered this topic in 2022 (indicators 5.3.1 and 5.3.2). Meanwhile, the health dimensions of climate change are increasingly acknowledged in the public discourse, with 24% of all climate change newspaper articles in 2022 referring to health, just short of the 26% in 2020 (indicator 5.1). Importantly, international organisations are increasingly engaging with the health co-benefits of climate change mitigation (indicator 5.4.2), and governments increasingly acknowledge this link, with 95% of updated Nationally Determined Contributions (NDCs) under the Paris Agreement now referring to health—up from 73% in 2020 (indicator 5.4.1). These trends signal what could be the start of a life-saving transition.

**A people-centred transformation: putting health at the heart of climate action:**

With the world currently heading towards 3°C of heating, any further delays in climate change action will increasingly threaten the health and survival of billions of people alive today. If meaningful, the prioritisation of health in upcoming international climate change negotiations could offer an unprecedented opportunity to deliver health-promoting climate action and pave the way to a thriving future. However, delivering such an ambition will require confronting the economic interests of the fossil fuel and other health-harming industries, and delivering science-grounded, steadfast, meaningful, and sustained progress to shift away from fossil fuels, accelerate mitigation, and deliver adaptation for health. Unless such progress materialises, the growing emphasis on health within climate change negotiations risks being mere healthwashing; increasing the acceptability of initiatives that minimally advance action, and which ultimately undermine—rather than protect—the future of people alive today and generations to come.

Safeguarding people’s health in climate policies will require the leadership, integrity, and commitment of the health community. With its science-driven approach, this community is uniquely positioned to ensure that decision makers are held accountable, and foster human-centred climate action that safeguards human health above all else. The ambitions of the Paris Agreement are still achievable, and a prosperous and healthy future still lies within reach. But the concerted efforts and commitments of health professionals, policy makers, corporations, and financial institutions will be needed to ensure the promise of health-centred climate action becomes a reality that delivers a thriving future for all.

## Introduction

Due to human activity, the global 10-year mean temperature reached 1·14°C above pre-industrial levels in 2013–22,^1^ triggering global climate and environmental changes that pose an unequivocal, immediate, and worsening threat to the health and survival of people worldwide.^2^ The past 8 years were the warmest ever registered;^3^ record-breaking extreme weather events occurred in every continent in 2022; and July, 2023, was the hottest month ever recorded; with detection and attribution studies showing the influence of climate change in making many of these events more severe or likely to occur.^4–16^ A record hot summer caused almost 62 000 deaths in Europe in 2022;^17^ extreme floods affected 33 million people in Pakistan and 3·2 million people in Nigeria;^16,18,19^ a record drought in the Greater Horn of Africa,^20^ made more severe by climate change, contributed to worsening local food insecurity, which now affects 46·3 million people;^21^ wildfires scorched parts of Europe,^22,23^ South America,^24,25^ and China;^4,26^ and less noticeable, but deeply damaging, slow-onset climate-related events are altering infectious disease distribution, affecting food security, impacting essential infrastructure, and undermining socioeconomic determinants of health.^2,27–31^ As a result, the impacts of climate change on physical and mental health are rapidly growing. Although no region is unaffected, the most vulnerable and minoritised populations, who often contributed least to climate change, are disproportionately affected—a direct consequence of structural injustices, and harmful power dynamics, both between and within countries.^2,32–34^

Although countries committed to pursuing “efforts to limit the temperature increase to 1·5°C above pre-industrial levels” in the 2015 Paris Agreement, GHG emissions reached record levels in 2021, and again in 2022.^35,36^ Unless urgently rectified, current policies will lead to a potentially catastrophic 2·7°C [range 2·2°C—3·4°C] of heating by 2100.^37^ Last year, the 2022 report of the *Lancet* Countdown found that global health lies at the mercy of fossil fuels,^38^ and with the threat of climate change increasing, further delays in meaningful climate change mitigation put the world at risk of missing “a rapidly closing window of opportunity to secure a liveable and sustainable future”.^39,40^

### Putting health at the centre of climate change action

Averting the worst impacts of climate change requires profound and immediate systemic changes, many with the potential to improve the health profile of world populations.^41^ To enable a healthy future, these changes must go beyond the treatment of the health symptoms of climate change, to put particular focus on primary prevention and rapidly accelerating mitigation efforts across all sectors, and ensure that climate change impacts stay within the bounds of the adaptive capacity of health and health-supporting systems (panel 1).

A zero-carbon transition will not only avoid the worst health impacts of climate change but can simultaneously deliver major health and socioeconomic co-benefits. Health-centred adaptation efforts are equally necessary to minimise the effects of now inevitable temperature rise on human health and survival and, by strengthening health and health-supporting systems, will have rippling benefits to public health. However, realising these health gains requires that human health and survival be central considerations in how international organisations, governments, corporations, and individuals understand and address climate change.

Conference of the Parties (COP)28 will be the first COP to feature health as a core theme—a substantial step forward for advancing health-centred climate change action (panel 2). The renewed demand for health-centred climate change action reflects years of engagement and continuous efforts of the scientific and health community and offers a unique opportunity to build a healthy future for all. However, this opportunity will become a hazard if short-term health promises are used as a screen to divert attention away from the imperative need to limit global temperature rise to 1·5°C, transition away from fossil fuels, and deliver transformational benefits to global health.

### A health stocktake for a thriving future

Ensuring that health-promoting climate action is delivered at the necessary speed and scale requires a regular exercise of stocktaking and monitoring. To fulfil this purpose, the *Lancet* Countdown: tracking progress on health and climate change was established as a multidisciplinary, international collaboration that works to annually take stock of the evolving links between health and climate change. Providing the most up-to-date assessment of the links between health and climate change, its findings are published ahead of the UN Climate Change Conference, focused on identifying the changing health impacts of climate change, and keeping countries accountable for their progress. Building on 8 years of iterative improvement of the monitoring framework, this year’s findings inform recommendations for key actions to enable a healthy, thriving future for all (panel 1).

The 2023 report of the *Lancet* Countdown represents the efforts, expertise, and dedication of 113 researchers from 52 academic and UN institutions from all continents but Antarctica, guided by the *Lancet* Countdown’s Scientific Advisory Group and High-Level Advisory Board.^53^ Its data are the product of 8 years of iterative improvements of 47 indicators (panel 3), built on the priorities identified through a global consultation among experts and policy makers.^54^ Following strict criteria of quality, scientific rigour, and relevance,^53^ the *Lancet* Countdown indicators are periodically refined, improving existing indicators and introducing new indicators as the availability of data and methods evolves (panel 4).^53^ An independent quality improvement process provides rigour and transparency to the collaboration’s data, incorporating input from independent experts on all new or substantially improved indicators to complement the *Lancet*’s peer review.^53^ Although methodological constraints and limits in the availability of data with adequate geographical and temporal coverage impedes the capacity to address persistent gaps in the *Lancet* Countdown’s indicator suite, the *Lancet* Countdown continues to work to address these gaps, welcoming contributions from fellow researchers for indicator improvement and development.

In this year’s report, most indicators have been substantially improved, including through methodological improvements, improved temporal coverage, and increased geographical coverage. New metrics now provide improved attribution of impacts to climate change, project future risks, and better account for the health co-benefits of climate change action and a zero-carbon financial transition. Complementing this report, data are presented in higher geographical and temporal detail in the *Lancet* Countdown’s freely available online data visualisation platform. Methodological details and further findings are presented in the appendix, alongside a description of the caveats and limitations of each indicator—making the appendix an essential companion to fully interpret the findings in this report.

### Elevating regional perspectives

Local contexts define the health impacts of climate change and opportunities for climate change action and must be understood to ensure climate change actions protect health, reduce inequities, and maximise associated health co-benefits. To this end, the *Lancet* Countdown has established regional centres worldwide, to generate regionally led policy-relevant evidence on the local links between health and climate change. The centres in Asia (Tsinghua University, China),^55^ South America (Universidad Peruana Cayetano Heredia, Peru),^56^ Europe (Barcelona Supercomputing Center, Spain),^57^ and Oceania (Macquarie University and the University of Sydney, Australia)^58^ have well established networks of regional researchers producing indicator reports for their respective regions or key countries within them. The growing Small Island Developing States (SIDS) centre (University of the West Indies, Jamaica) will publish their first report in 2024, and efforts are underway to develop a new African centre.

Driven by the expertise of the regional centres, a new section in this report provides a global comparison of the health impacts of climate change, and progress, opportunities, and constraints for climate change action across world regions (part A). This section complements the more detailed, regionally focused analysis in the *Lancet* Countdown’s regional indicator reports, which are due to be published in upcoming months and will cover the regional, national, and on occasion, subnational progress on health and climate change in more detail.

### An ambitious new phase to match the urgency of action

The path to a liveable future is becoming more difficult with every moment of inaction. In 2024, the *Lancet* Countdown will increase its ambition, with further-resources to monitor and inform an urgent and healthy transition. Efforts will focus on addressing persistent research gaps (including links with mental health, migration, and the disproportionate impacts of climate change on minoritised communities), and supporting decision makers and international negotiations to enact policies on the basis of this evidence. Across both global and regional efforts, the *Lancet* Countdown will deepen its strategic efforts to increase representation, equity, and inclusion in its collaboration and work. In its new phase, the *Lancet* Countdown will continue to welcome input from researchers worldwide to develop increasingly refined and globally representative metrics.^53^ By doing so, it will continue to foster a global and interdisciplinary collaboration working to produce timely and actionable evidence to support health-promoting climate change action, and a thriving future for all.

### Part A: evolving regional progress and inequities in health and climate change

Climate change impacts are experienced locally and a comprehensive assessment of the links between health and climate change requires local perspectives, experience, and knowledge. With the expertise of the *Lancet* Countdown’s regional centres, this part of the report draws on the findings of the indicators presented in Part B to provide an assessment of the climate change risks, responses, and opportunities across world regions. This section will be complemented by upcoming reports from the *Lancet* Countdown’s regional centres, which will explore in further detail the evolving health profile of climate change in each region, including highlighting (wherever possible) in-country inequities, through local high-quality data. More information on the *Lancet* Countdown’s regional groupings and indicator findings is provided in the appendix (pp 2–6).

### The unequal health impacts of climate change

Climate change is affecting people unequally around the world.^59^ Annually in 2018–22, people in SIDS, Africa, South and Central America, and Asia experienced the highest number of days of health-threatening temperatures attributable to climate change (103, 78, 72, and 47 days per person, respectively; indicator 1.1.5). With more frequent health-threatening temperatures and a growing population of people older than 65, Africa experienced the biggest increase in a heat-related mortality rate since 2000–05. However, Europe had the highest rate of heat-related mortality in recent years (2017–22; indicator 1.1.5).

Heat exposure limits labour productivity, undermining livelihoods. In 2013–22, it resulted in 189 potential labour hours lost annually per worker in Asia and 161 in Africa (indicator 1.1.4). As a result, Africa also saw the highest relative potential income loss in 2022, equivalent to 4·1% of its GDP, with 81% of potential income losses generally falling on often the most vulnerable and least protected agricultural workers with the lowest incomes (indicator 4.1.3). In addition, the WHO regions of Africa and the Western Pacific had higher proportions of outdoor workers (32·1% and 29·8%, respectively), placing workers in these regions at particularly heightened risk from climate hazards (indicator 1.1.4).^60^

Compounding rapidly rising temperatures, droughts increasingly affect global food security (indicator 1.4), water security, sanitation, supply chains, and energy generation.^27,61,62^ Africa was also the region most affected by droughts, with 64% of its land area affected by at least 1 month of extreme drought per year on average in 2013–22, up from 9% in 1951–60. In the Horn of Africa, some areas experienced a full 12 months of drought in 2022, pushing millions to the brink of famine.^21,23,63,64^ Mostly reflecting Australia’s record 2017–20 drought, Oceania was the second most affected region, with 55% of its land area experiencing extreme drought in 2013–22 (an increase from 14% in 1951–60).^65,66^ In South and Central America, 53% of land area was affected in 2013–22, including year-round droughts in parts of central Brazil’s Amazon rain-forest, increasing risks of forest dieback (indicator 1.2.2).^67^ Rising sea surface temperatures also threaten marine food yields.^68^ European and North American coasts saw the largest increases in sea surface temperature in 2022, compared with 1981–2010 (0·83°C and 0·73°C, respectively; indicator 1.4). As a result, many fishing communities, including Arctic Indigenous communities in North America, face rising food insecurity.^69–73^

In addition, heating seas and melting ice bodies increase hazards from sea level rise.^2^ Asia, SIDS, and Europe have the largest proportions of populations settled in areas less than 1 m above current sea level (2·8% of the Asian population, 2·0% of the SIDS population, and 1·5% of the European population), which face risks of coastal erosion, floods, and salinised land and water resources (indicator 2.3.3). The hotter seas are also making coastal brackish waters increasingly suitable for the transmission of some *Vibrio* pathogens (including *V parahaemolyticus, V vulnificus*, and non-toxigenic *V cholerae*): from 1982 to 2022, Europe experienced the biggest increase in the length of coastline suitable for *Vibrio* spp at any one time in the year (142 km annually, reaching 17% of its coastline). Meanwhile, annually, an additional 83 km of coastline in Asia became suitable for *Vibrio*, reaching 17% of its coastline in 2022, and leading to an estimated increase of 5000 cases of vibriosis annually, totalling some 421 000 cases in 2022 (indicator 1.3).

Changes in temperature, rainfall, and humidity are also altering environmental suitability for the transmission of many mosquito-borne diseases. As a result the transmission potential for dengue is also increasing, contributing to its rapid global expansion.^74^ From 1951–60 to 2013–22, South and Central America had the biggest increase in dengue transmission potential (R0), an increase of 0·86 and 1·03 for *Aedes aegypti* and *Ae albopictus*, respectively. (indicator 1.3). Meanwhile, the transmission season for malaria is lengthening in many regions, with the biggest increase in African highlands for *Plasmodium falciparum* (0·61 months) and in South and Central American highlands for *P vivax* (0·8 months). The transmission season lengthened by at least 1 week in North American lowlands and South and Central American highlands for both parasites, and in both highlands and lowlands in SIDS.

As climate change-related health risks increase, effective local adaptation (informed by an in-depth understanding of local vulnerabilities and hazards) is essential to protect human health and survival and reduce health inequities. However, measures to prepare and respond to health emergencies are lagging in all world regions (indicator 2.2.5). Moreover, although health system strengthening has reduced vulnerability to severe outcomes from mosquito-borne diseases in Africa, South and Central America, and SIDS since 1990, urbanisation is now increasing vulnerability worldwide largely due to high population density and built environment conditions that favour the breeding of some mosquito species and the transmission of mosquito-borne disease (indicator 2.3.1).

With 55% of the world population living in urban centres, city-level interventions hold enormous potential and must be informed by in-depth understanding of local risks and vulnerabilities. In 2022, between 80% and 92% of surveyed cities in Oceania, Europe, and North America reported that they had completed a climate risk and vulnerability assessment. However, the proportion was considerably lower in Africa (43 of 69 cities, 62%), South and Central America (149 of 268, 56%), and Asia (117 of 231, 51%; indicator 2.1.3), regions that are the most unprotected, and in which climate hazards are rapidly accelerating.

### The regional health inequities of an unjust transition

Energy-related emissions are the biggest single contributor to climate change, but these emissions vary greatly among world regions. In 2020, the regions with the highest average energy sector emissions per person were Oceania (13·4 tonnes of CO_2_ [tCO_2_]/person on average, mostly driven by Australia) and North America (12·9 tCO_2_/person); regions where low political engagement on climate change resulted in insufficient, and often negligible, climate change action.^75^ CO_2_ emissions per person in Oceania were 14 times higher than in Africa (0·97 tCO_2_/person), and 3·4 times higher than in Asia (3·9 tCO_2_/person; 7·4 tCO_2_/person in China). However, with 61% of the world’s population, Asia contributed 59% of all global energy-related CO_2_ emissions in 2020 (17·7 gigatonnes of CO2 [GtCO_2_], 57% of regional emissions from China; indicator 3.1.1).

Although renewable energy generation is increasing in all regions, it has not substantially replaced fossil fuels: North America reduced the carbon intensity of its energy sector by 15% between 1992 and 2020, a trend which the US Inflation Reduction Act of 2022 seeks to accelerate.^76^ However, at its 2011–20 decarbonisation pace, North America would take 82 years to fully decarbonise its energy sector. Similarly, the carbon intensity of Europe’s energy system decreased 22% between 1992 and 2020 and would take 80 years to fully decarbonise at the current pace (indicator 3.1.1). As countries seek new energy sources amid the current energy crisis, the situation could worsen, including as a result of the USA’s approval of the oil drilling Willow Project in Alaska and coal phase-out deceleration in European countries.^77,78^

Renewable investment is also unequally distributed. Only 1% of renewable energy investments in 2022 were in Africa.^42^ Despite plentiful renewable energy resources, clean renewables accounted for just 1·0% and 0·4% of the energy supply in Africa and SIDS in 2020, respectively, compared with 2·4% in North America, 2·7% in Asia and South and Central America, 3·0% in Europe, and 6·0% in Oceania (indicator 3.1.1).^79^ This situation perpetuates reliance on polluting fuels, particularly in regions with scarce access to energy. In 2020, biomass and waste burning still contributed to 84% of the household energy consumption in Africa, 46% in SIDS, 33% in South and Central America, and 32% in Asia, against 5% to 11% in North America, Oceania, and Europe (indicator 3.1.2). The global energy and economic crisis means investment and access to non-polluting household energy could decrease further.^80^ The highly unequal use, deployment of, and access to renewable energy across world regions contrasts starkly with the availability of the natural resources that energy technologies require, and results from—and perpetuates—harmful global power dynamics.^81^

Air pollution from the energy sector continues to generate long-lasting health impacts in every region, particularly in urban centres. Of all global deaths attributable to fuel-derived particulate matter less than 2·5 µm in diameter (PM_2·5_), 77% occurred in Asia (1·3 million). With coal still contributing 43% of the continent’s total energy, Asia had the highest mortality from coal-derived PM_2·5_ of all the regions, at 11 deaths per 100 000 (indicator 3.2.1). In Europe, air quality control measures coupled with a 5·2 percentage point reduction in the share of coal-derived energy since 2005 contributed to a 36% decrease in ambient PM_2·5_-related mortality, 44% of which was due to reduced coal-related pollution. Despite this decline, Europe still had the highest death rate from outdoor PM_2·5_ (69 deaths per 100 000) and dirty fuel (fossil fuels and biomass)-derived PM_2·5_ (38 deaths per 100 000) in 2020 (indicator 3.1.1 and indicator 3.2.1). Moreover, through imports, 33% of the PM_2·5_ emissions induced by European consumption contaminates the air in other world regions (indicator 4.2.5).

Despite these health harms, governments continue to hamper the transition to clean, renewable energies by subsidising fossil fuels.^82,83^ Of the 87 countries analysed (appendix p 189), which contribute 93% of global GHG emissions, all of the countries in Africa (n=8) and South and Central America (n=9) still provided net subsidies to fossil fuels, and had the lowest median net carbon prices of all regions in 2020. In contrast, the highest median net carbon prices (lowest effective subsidies) were in European countries (n=34) and North American countries (n=2), with North America being the only world region with net fossil fuel taxes, of 0·9 $/tonne (indicator 4.2.4). Although fossil fuel subsidies can improve energy access, they are inefficient and often regressive.^84^ These funds could be redirected to promoting access to clean renewable energy or to improving health and wellbeing, delivering net health benefits to forge a liveable future.^85^

Delayed mitigation in the food sector has also come at a high health cost. Oceania and North America, with high levels of red meat consumption, and South and Central America, with carbon-intensive meat production systems, had the largest emissions per person from red meat consumption in 2020, representing 86%, 70%, and 81% of their agricultural emissions, respectively. Emissions per person in these regions were 4·2-times (Oceania), 2·3-times (North America), and 2·6-times (South and Central America) higher than emissions per person in SIDS, the region with the lowest emissions per person from red meat consumption (indicator 3.3.1). Shifting towards more affordable and accessible plant-based diets can reduce these emissions, simultaneously delivering substantial health benefits. This is particularly true for populations in North America, Europe, and Oceania, which have the highest mortality from excess consumption of red and processed meat, and from insufficient consumption of fruits, vegetables, legumes, and whole grains (indicator 3.3.2).

These data reveal the deep global inequities that underpin delays in climate change mitigation, and underline the health imperative for building just, equitable, and environmentally sustainable systems for the extraction of, access to, and use of energy and natural resources^79^ that leave no one behind. To achieve this, the transition to a zero-carbon, healthy future must avoid reproducing harmful extractive practices that disproportionately harm the health of minoritised groups, including individuals living in low HDI countries, rural communities, and Indigenous peoples.^34,81^

### Growing but unequal engagement on health and climate change

To maximise the benefits to human health and survival, climate change action must be informed by evidence, understanding of and engagement with the local interactions between health and climate change, and harnessing the knowledge of Indigenous peoples and other communities at the frontline of climate change impacts. However, the generation of scientific evidence is uneven across world regions. The region most studied in 2022 was Asia, with 1095 peer-reviewed articles exploring the links between climate and health. The majority of these articles (59%) focused on China and India. North America and Europe followed, with 398 and 305 studies, respectively. 254 studies focused on Africa, 142 studies on South and Central America, and 80 on Oceania. With only 51 articles, SIDS was the least studied region, emphasising the urgency to expand research on health and climate change in these vulnerable states (indicator 5.3).

References to health in the first round of NDCs were common in the most vulnerable regions, with 84% to 100% of countries in South and Central America, Africa, Asia, and SIDS referring to them, but substantially less so in North America, Oceania, and Europe (50%, 33%, and 14% of NDCs, respectively). In the second round of NDCs, all South and Central American, SIDS, and North American countries, 97% of African countries, and 92% of European NDCs mentioned health. The trend was only reversed for Oceania, where no country mentioned health (indicator 5.4.1). However, the average proportion of countries referencing the climate–health nexus in the UN General Debate in 2018–20 was the lowestinSouthandCentralAmerica (18·7%), Asia(22·8%), and Africa (26·7%)—regions that suffer the most from climate impacts.

### Conclusion

Climate change is placing human health and survival at risk in every region of the world. However, these threats vary widely: although climate hazards are determined by wide-ranging climates and topographies, vulnerabilities depend on highly unequal, local epidemiological and socioeconomic characteristics. As a result, the most underserved countries and communities are currently disproportionately affected. A just transition that minimises global inequities and avoids negative impacts, and that ensures no one is left behind is essential to a healthy future.^86^ However, countries that have historically contributed the least to climate change often have scarce resources for and a lag in the implementation of adaptive solutions, further amplifying health inequities. Inadequate funding has been a major barrier to a just transition, aggravated by the undelivered Copenhagen Accord commitment of mobilising $100 billion annually to support climate change action in countries labelled as developing in the context of the UN Framework Convention on Climate Change (UNFCCC).^87^

Mitigation efforts have likewise been woefully inadequate and inequitable. Although the regions with the highest emissions per person (North America, Europe, and Oceania) are accelerating decarbonisation efforts, the current pace falls far short of Paris Agreement ambitions.^40^ Beyond exacerbating climate risks, this inaction has come at substantial health costs for local populations, with high mortality rates from fuel-derived air pollution. Meanwhile, countries in Africa, Asia, South and Central America, and SIDS are being left behind in the transition to non-polluting energy, despite their plentiful natural renewable energy resources. The resulting high levels of dirty fuel use, household air pollution, and poor energy access in these regions expose the health costs of unjust climate change action—stressing the need to foster equity in the access to and use of non-polluting energy technologies to support sustainable development, improve health, and reduce global inequities.^88,89^

To be effective, the transition to clean, zero-emission energy must be enabled through financial mechanisms and, importantly, be equitable. As such, it must ensure that lower HDI countries are empowered and enabled—including through financial and technical support—to develop and deploy local renewable energy technologies by (among other means) implementing robust policies and regulations that prevent the replication of harmful extractive industrial models that widen health inequities and disproportionately affect the health of populations in resource-rich, lower HDI countries.^90^

Realising the transformative public health opportunities of just and urgent climate change action requires a deep understanding of the links between climate and health at a local level. To support this understanding, the *Lancet* Countdown’s regional centres are working to produce locally relevant scientific evidence, led by local researchers. Upcoming regional reports will enhance the evidence provided in this section, to support decision makers in a healthy transition to a net-zero future. The extent to which scientific evidence is collectively acted upon will ultimately define the global health profile for generations to come.

## Part B: taking stock of progress on health and climate change

The following sections present the eighth annual update of the *Lancet* Countdown’s indicators, which monitor global progress on health and climate change. The indicators have been substantially improved this year, providing a more comprehensive and relevant global stocktake. Indicators tracking health hazards, exposures, and impacts now better distinguish the influence of changing climate from other drivers and, in a major shift, now also include projections whenever possible, building on an effort commissioned and supported by the Climate Vulnerable Forum for its third Climate Vulnerable Monitor.^91^ Newly introduced indicators and subindicators monitor high temperatures attributable to climate change, the environmental suitability for West Nile virus transmission, household air pollution, bank lending for fossil fuel and clean renewable energy industries, and the scientific assessment of the health impacts of extreme events, focusing on detection and attribution studies. A full account of the changes, alongside more detailed descriptions of the findings, are provided in the appendix (p 7).

## Section 1: health hazards, exposures, and impacts

Climate change is already affecting the physical, environmental, and socioeconomic conditions on which human health and survival depend. Section 1 tracks the health hazards, exposures, and impacts of climate change globally. The first group of indicators tracks the multidimensional effects of heat on health. The second group tracks the health threats and impacts of extreme weather and weather-related events. The two final indicators track slower onset events: climate suitability for infectious disease transmission, and the effects of changing climate on food insecurity. Most indicators track spatiotemporal changes in weather and climate, integrating demographic data to track health-related outcomes in exposed populations.^92,93^ New data track the health-threatening hot days attributable to climate change, the climate suitability for West Nile virus transmission, and the number of outdoor workers who are most exposed to climate hazards. As a major addition this year, this section builds on contributions to the third Climate Vulnerable Monitor,^91^ now including projections under a scenario in which action is taken to limit global mean surface temperature rise to 2°C, stabilising at 1·8°C by 2100 (shared socioeconomic pathway [SSP]1–SSP2.6), and under one that assumes no further mitigation, in which heating reaches 3·6°C above pre-industrial levels by 2100 (SSP3–SSP7.0).^94^ These projections show the risks of climate inaction and stress the urgency of accelerating mitigation efforts to limit global mean surface temperature rise to 1·5°C and increasing adaptation to ensure a liveable future.

### 1.1 Heat and health

Heat exposure can result in heat-related illness, exacerbate underlying health conditions, and lead to mental ill health and adverse pregnancy and birth outcomes.^95–102^ High temperatures also affect people’s capacity to work and their willingness to undertake physical activity.^103–105^

#### Indicator 1.1.1: exposure to heating—headline finding: from 1986–2005 to 2022, populations were exposed to an average increase in summer temperature three times the global mean

Land areas, and particularly urban areas, are heating up faster than the global average.^106,107^ This indicator tracks the population-weighted change in global summer temperatures and shows that humans experienced triple the mean global temperature increase between 2022 and the 1986–2005 baseline (a 0·9°C population-weighted summer temperature change compared with a 0·3°C global mean summer temperature change).

#### Indicator 1.1.2: exposure of vulnerable populations to heatwaves—headline finding: in 2013–22, infants and people older than 65 years experienced, on average, 108% more days of heatwave per year than in 1986–2005

Infants and older adults are particularly vulnerable to adverse health effects from heat exposure.^99,108^ This indicator monitors the exposure of these highly vulnerable age groups (children younger than 1 year and people older than 65 years) to heatwaves days (defined as a period of 2 or more days where both the minimum and maximum temperatures are above the 95th percentile of temperatures in 1986–2005).^109,110^

Compared with 1986–2005, the number of heatwave days during 2013–22 increased 94% globally. This increase resulted in each child younger than 1 year being exposed on average to 110% more days of heatwave, on average, in this time period (4·0 days in 1986–2005, increasing to 8·4 days in 2013–22), whereas each person older than 65 years was exposed on average to 96% more days of heatwave (increasing from 5·0 days to 9·8 days). Combined with demographic changes, the total person-days of heatwave exposure increased 134% for children younger than 1 year, and 228% for people older than 65 years.

Projections estimate a 1120% increase in heatwave exposure for people older than 65 years by 2041–60 compared with 1995–2014 under a scenario compatible with limiting global temperature rise to 2°C, increasing to a 2510% increase in exposure by 2080–2100. Under a scenario of no further mitigation, the projected increases are even higher, rising to 1670% by mid-century, and 6311% by 2080–2100.

#### Indicator 1.1.3: heat and physical activity—headline finding: in 2013–22, compared with 1991–2000, there were 241 additional hours annually, during which ambient heat posed a moderate or higher risk of heat stress during light outdoor physical activity

Regular physical activity provides health benefits throughout the lifecourse,^111,112^ and represents an effective, low-cost, and low-emission intervention for reducing the risk of non-communicable diseases and health-care demand.^113,114^ However, heat can be a motivational barrier to engagement in physical activity, and can increase the risk of heat illness for people who do engage in activity.^115^ This indicator incorporates temperature, humidity, and solar radiation to estimate the hours during which ambient conditions present a heightened risk of heat stress if undertaking outdoor exercise.

**Figure 1:**
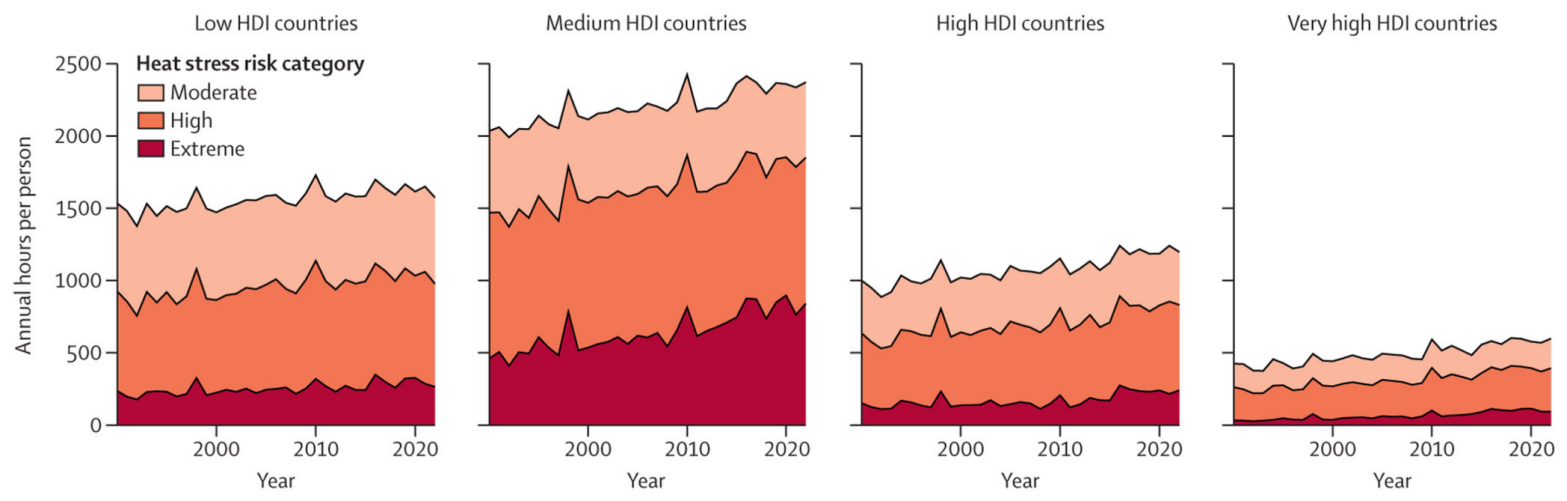
Average annual hours per person from 1991 to 2022 when light physical activity entailed at least a moderate, high, or extreme heat stress risk, arranged by HDI country groupings HDI=Human Development Index.

Compared with 1991–2000, the hours of at least moderate risk of heat stress for light outdoor physical activity (eg, walking) increased by an average of 241 hours per person (20·1% increase) annually during 2013–22. For moderate intensity activity (eg, jogging or cycling), there was an increase of 253 hours (19·0% increase; figure 1).

Under a scenario compatible with limiting global temperature rise to 2°C, an additional 426 hours per person would pose at least a moderate risk of heat stress during light physical activity on average annually by 2040–60 compared with 1995–2014. This number would rise to 596 hours per person under a scenario with no further mitigation. By the end of the century (2080–2100), the annual average number of hours per person posing at least a moderate risk of heat stress would increase slightly to 451 hours under a scenario compatible with 2°C of heating; under a scenario with no further mitigation, these would increase sharply to 1124 hours per person each year.

#### Indicator 1.1.4: change in labour capacity—headline finding: heat exposure led to the loss of 490 billion potential labour hours in 2022, a nearly 42% increase from 1991 to 2000

Heat exposure reduces labour productivity and puts workers’ health at risk, particularly for individuals undertaking physically strenuous labour, working in non-cooled environments, or working outdoors.^103^ The resulting loss of labour capacity undermines livelihoods and the socioeconomic determinants of health.^116^ This indicator monitors the potential work hours lost as a result of heat exposure, by associating temperature, humidity, and solar radiation (via wet-bulb globe temperature) with the typical metabolic rate of workers in specific economic sectors.

In 2022, heat exposure resulted in a loss of 490 billion potential labour hours, 42% more than the annual average in 1991–2000. On average, each worker in the world lost 143 potential hours of labour capacity. Over 1·3 billion workers, 39% of the global workforce, experienced losses greater than that, and 80% of these were from low or medium HDI countries. By contrast, 87% of workers experiencing losses below the average lived in high or very high HDI countries. By 2041–60 (and without further adaptation), a scenario compatible with limiting temperature rise to 2°C would result in more than a doubling of potential labour hours lost annually compared with the 1995–2014 period; with no further mitigation, this would be nearly 2·5-times higher. By the end of the century, losses relative to the 1995–2014 baseline will increase by 117% in a scenario compatible to 2°C of temperature rise, and by 458% in a scenario in which no further mitigation action is taken (assuming no further adaptation).

New to the 2023 report, the number and percentage of working-age, outdoor workers (the group most affected by heat-related labour capacity loss and heat-related health risks) were estimated for 195 countries or areas, with UN estimates of occupational sunlight exposures and working-age populations.^117,118^ Globally, in 2022, an estimated 1·6 billion paid workers—26·4% of the working-age population—worked outdoors. Men (38·4% of all men) and young or middle-aged groups (33·4% of people aged 25–54 years) were over-represented, although unpaid labour, to which women often dedicate more time than men, was not accounted for in these figures.^119^ Between 2000 and 2022, there were reductions in both the number of outdoor workers (–0·2 billion workers) and the percentage of working-age people who worked outdoors (–15·3%).

#### Indicator 1.1.5: heat-related mortality—headline finding: in 2018–22, people experienced, on average, 86 days of health-threatening high temperatures annually. 60% of these temperatures were made more than twice as likely to occur by human-caused climate change

Ageing populations, an increasing incidence of non-communicable diseases in many countries, and urbanisation are increasing populations’ vulnerability to extreme heat. Compounding with rising temperatures, this vulnerability is driving a rapid increase in heat-related deaths globally, a third of which are attributable to anthropogenic climate change.^120^

**Figure 2:**
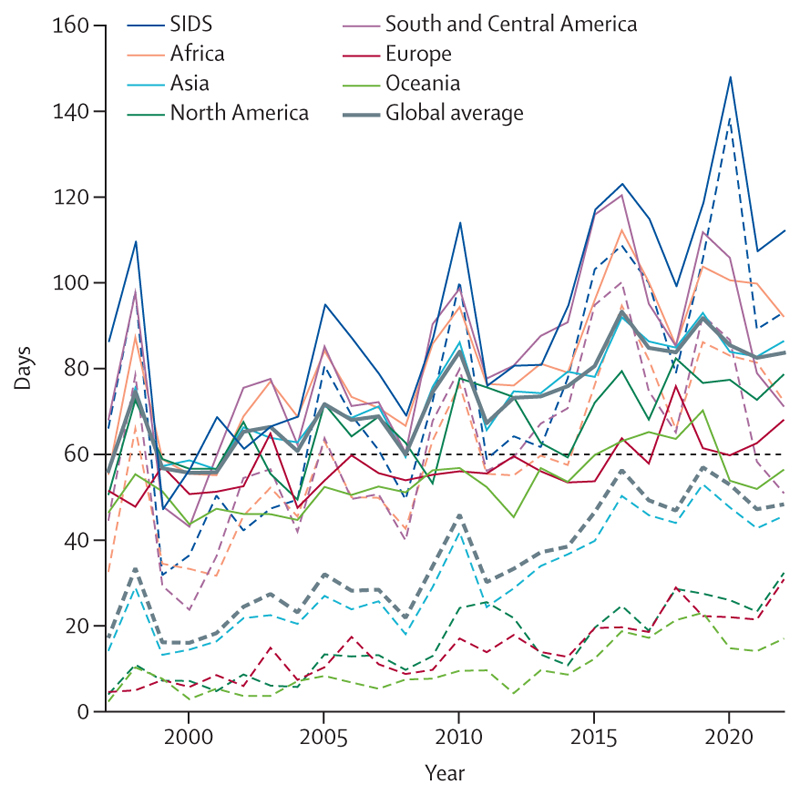
Population-weighted days of exposure to temperatures above the 84th percentile for 1986–2005 In a climate with no anthropogenic climate change, this value would be expected to be close to 60 days (dashed black line). The number of days of exposure to warm temperatures for different regions are displayed as solid lines; the heavy solid line is the global average. The number of days of exposure to warm temperatures made at least twice as probable due to climate change are plotted as dashed lines; the heavy dotted line is the global average. SIDS=Small Island Developing States.

The first part of this indicator identifies days on which temperatures exceed a conservative threshold over which heat-related deaths are likely to increase (above the 83·6th percentile of temperatures in 1986–2005) and calculates the extent to which human-caused climate change increased their likelihood. It finds that people were exposed, on average, to 86 days per year of health-threatening high temperature in 2018–22 (figure 2), 60% of which were made more than twice as likely due to anthropogenic climate change.

The second part of the indicator combines exposure to temperatures above this threshold with an exposure–response function to model the change in heat-related mortality in people older than 65 years.^121^ In 2013–22, compared with 1991–2000, the estimated average annual heat-related mortality increased by 85%, driven by both warming and changing demographics. A counterfactual simulation keeping temperatures unchanged from baseline values shows that demographic changes alone would have resulted in just a 38% increase in mortality in 2013–22, compared with 1991–2000.^122^

Annual heat-related mortality of people older than 65 years is projected to increase by 370% above 1995–2014 levels by 2041–60 under a scenario compatible with limiting global temperature rise to 2°C, and by 433% under a scenario in which no further mitigation occurs, assuming no further adaptation. By 2081–2100, these mortality levels are projected to increase by 683% and 1537% for the two scenarios, respectively.

### 1.2 Health and extreme weather-related events

Climate-change-driven increases in temperature and changes in rainfall patterns are increasing the frequency, intensity, and duration of life-threatening extreme weather and weather-related events.^123^ These events pose direct and indirect risks to physical and mental health.^50,98,100–102,124–141^ The indicators below track the changing incidence of key health-threatening extreme events. Efforts are ongoing to better capture the changing risk of floods—a crucial gap, which will be addressed in upcoming reports.

#### Indicator 1.2.1: wildfires—headline finding: the number of days in which people were exposed to very high or extremely high fire danger increased in 57% of countries between 2003–07 and 2018–22; however, exposure to wildfires decreased in 56 countries and increased in only seven during this period

Rising temperatures and incidence of drought increase the risk of wildfires, which affect health through thermal injuries, smoke exposure, loss of essential and health-supporting physical infrastructure, and effects on mental health.^59,142,143^

Population data and a Fire Danger Index, which captures meteorological fire risk,^144,145^ suggest that, on average, people globally were exposed to 6 more days of very high or extremely high wildfire danger in 2018–22 compared with 2003–07, with an increase observed in 57% of countries and no change or a decrease observed in the rest. However, potentially due to wildfire management and control, reduced availability of vegetation or other forms of fuel following previous fires, land use change (including urban expansion), or rural–urban migration continuing to concentrate populations in cities, 56 countries saw a statistically significant reduction in the days each person was exposed to active wildfires annually in 2018–22 compared with 2003–07, whereas only seven countries saw a statistically significant increase. During 2003–22, global average wildfire smoke concentrations, as estimated with the SILAM chemistry transport model and satellite observations of active fires,^146–149^ did not change significantly. However, there was a statistically significant increase in wildfire smoke concentrations in eastern Siberia, western USA, Canada, and India. In 2018–22, low and medium HDI countries were affected by 1·6-times higher wildfire smoke concentrations than high and very high HDI countries.

Compared with 1995–2014, the number of days of exposure to very high or higher wildfire risk is projected to increase by approximately 9 extra days per person (11% increase) by the middle of the century, both in a scenario compatible with limiting global temperature rise to 2°C, and in a scenario in which no further mitigation occurs. Towards the end of the century, a scenario with no further mitigation is projected to result in three times more days of exposure (27 more days than in 1995–2014) than in a scenario compatible with 2°C of heating (9·6 more days than in 1995–2014). Likewise, the global mean fire-related PM_2·5_ concentration would be 5% higher in the no-further-mitigation scenario than in a scenario compatible with 2°C of heating by 2041–60, but 50% higher by 2081–2100 assuming no further adaptation.

#### Indicator 1.2.2: drought—headline finding: the global land area affected by extreme drought per year increased from 18% in 1951–60 to 47% in 2013–22

In 2022, extreme drought affected every continent. Impacts on crop yields and loss of livestock worsened food insecurity, and water shortages affected water security in vulnerable areas.^20,21,23,65^ Low river flows hampered electricity generation and fluvial transportation, affecting energy access and economic activity.^23,25^ Women and girls, who are charged with water collection and distribution in many water-insecure settings, are likely to be increasingly exposed to gender violence and physical harms either as they have to travel longer distances to collect water, or as they are unable to fulfil these domestic tasks.^150^

**Figure 3:**
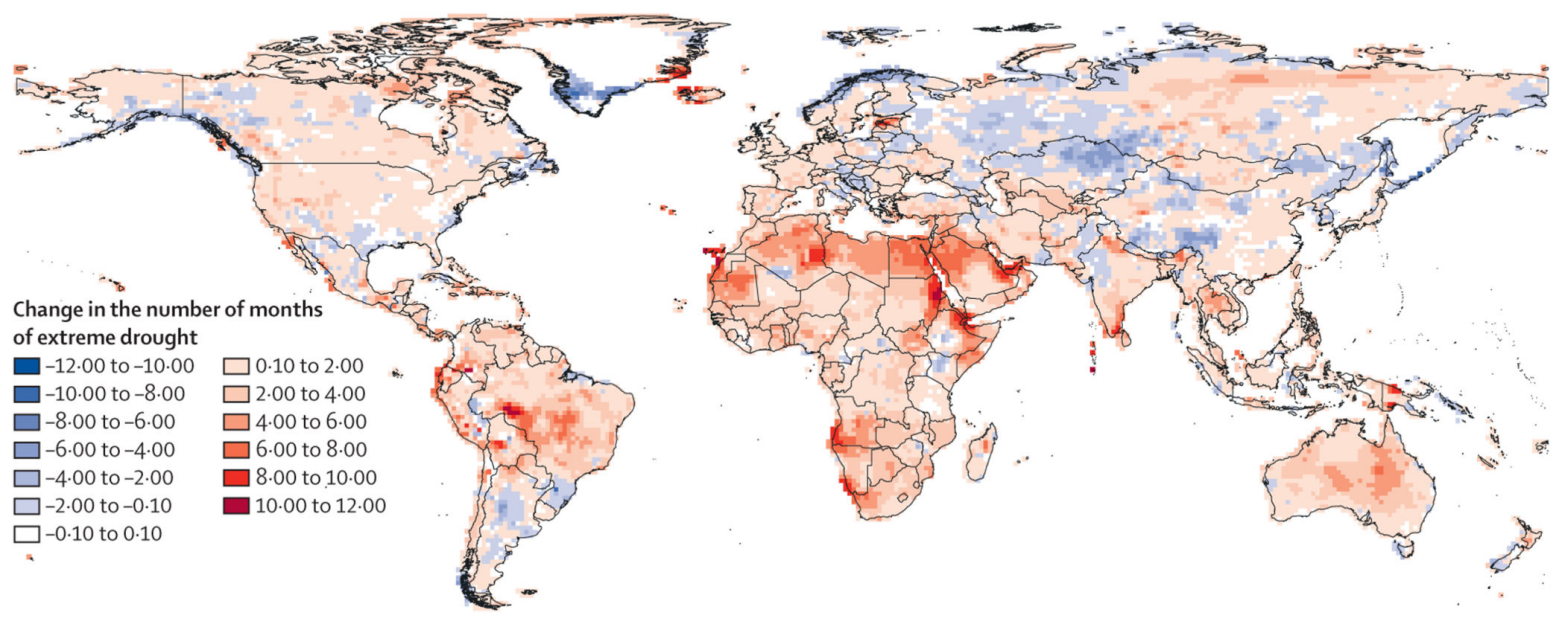
Change in the number of months of extreme drought per year from 1951–60 to 2013–22

Accounting for precipitation and heat-driven evapotranspiration,^151^ this indicator reports that the global land area affected annually by at least 1 month of extreme drought increased from 18% in 1951–60, to 47% in 2013–22 (figure 3). Year-round drought affected many vulnerable areas in 2022, including the Horn of Africa, the western region of the Sahara, and the southern Amazon region of Brazil.

#### Indicator 1.2.3: extreme weather and sentiment—headline finding: extreme weather in 2022 was associated with a record 0·53 percentage point reduction in online positive sentiment expression during heatwaves and a 0·31 percentage point reduction in positive sentiment expression during extreme precipitation days

Extreme weather events, including heatwaves and extreme precipitation, can affect people’s mental wellbeing.^131,140,152^ This indicator uses a multivariate fixed effects panel regression to monitor the effects of heatwaves and extreme precipitation on expressed online sentiments (as the share of tweets reflecting positive or negative expressions), adjusting for date, location, seasonality, and meteorological conditions.^38,152–154^ The lexical content of 8·2 billion tweets from 190 countries and roughly 44 000 localities was analysed. Social media use is more common in high and very high HDI countries, which also have greater ability to adapt to heat stress and other climate-related factors, compared with medium and low HDI countries. Thus, these estimates probably underestimate the true global impacts of environmental stressors on sentiment (appendix p 61).

Over the past 8 years, the adverse impact of heatwaves on both negative sentiment and positive sentiment increased in magnitude. Days with extreme precipitation had an increasingly negative effect on online positive sentiment expression between 2015 and 2022, whereas there was no noticeable trend for their effect on negative sentiment. Extreme weather in 2022 was associated with a record 0·53 percentage point reduction in online positive sentiment expression during heatwaves, and a 0·31 percentage point reduction in positive sentiment expression during extreme precipitation days.

### *Indicator 1.3: climate suitability for infectious disease transmission—headline finding: the annual average climatic suitability of West Nile virus transmission increased by 4·4% from 1951–60 to 2013–22; the transmission potential for dengue by* Ae aegypti *and* Ae albopictus *increased by 28·6% and 27·7%, respectively; and the coastline suitable for* Vibrio *spp transmission increased by 329 km annually since 1982*

Changing climatic conditions are altering the transmission potential of many vector-borne, water-borne, foodborne, and airborne infectious diseases.^155–157^ This indicator monitors the changing climatic suitability of West Nile virus, dengue, Zika virus, chikungunya, malaria, and non-cholera *Vibrio* pathogens.

West Nile virus is a climate-sensitive, mosquito-borne disease that circulates in birds and can spill over into humans via *Culex* mosquitoes.^158–161^ It can cause rare but severe illness involving the central nervous system.^162^ Over the past two decades, West Nile virus has emerged in the Americas and expanded in Europe, where it is becoming a public health concern.^163,164^ This new subindicator leverages experimentally established vector–pathogen–temperature relationships to track temperature-induced changes in the relative basic reproduction number for West Nile virus (WNV-R0), in regions where three relevant *Culex* vectors are present.^158,165^ The WNV-R0 was on average 4·4% higher in 2013–22 compared with 1951–60, with an increase in the very high (7·7%), high (6·6%), and medium (4·1%) HDI country groups, and a slight decrease in the low HDI country group (–0·7%).

Driven by climatic changes, urbanisation, and human movement, cases of dengue have doubled every decade since 1990, and almost half of the world population is now at risk of this life-threatening disease.^74,166^ With a mechanistic model that accounts for changes in temperature and precipitation, this subindicator shows that, relative to 1951–60, in 2013–22, the average R0 for dengue transmission by *Ae aegypti* and *Ae albopictus* increased by 28·6% and 27·7%, respectively. Other arboviruses are showing similar trends: the R0 for the transmission of chikungunya by *Ae albopictus* increased by 27·7%, and the R0 for the transmission of Zika by *Ae aegypti* increased by 31·9% during the same period. The length of the transmission season increased for these arboviruses by between 13·4% and 14·9%. The suitability for dengue transmission is expected to increase under all future scenarios of planetary heating. By mid-century, a scenario compatible with limiting global temperature rise to 2°C would see an increase in R0 of 19% for *Ae aegypti*, and an increase in R0 of 21% for *Ae albopictus* from 1995–2014 levels, whereas a scenario in which no further mitigation occurs would see increases of 27% and 30%, respectively. By the end of the century, R0 for *Ae aegypti* would increase 20%, whereas R0 *Ae albopictus* would increase 22% under the scenario compatible with 2°C of heating. Under the scenario with no further mitigation, R0 for *Ae aegypti* would increase 38% and R0 for *Ae albopictus* by 47%.

Fluctuations in the length of the malaria transmission season are inferred with the environmental and climatic requirements of the vector (*Anopheles* mosquitoes) and *Plasmodium* parasites.^167^ Overall, 9·85% of the land without suitable conditions for transmission of *P falciparum* in 1951–60 became suitable by 2013–22, whereas 17·34% became suitable for *P vivax*. Under a scenario compatible with limiting temperature rise to 2°C, 23% of areas not suitable for malaria transmission between 1995 and 2014 are projected to become suitable in 2041–60. However, under a scenario in which no further mitigation occurs, this figure rises to 26% in 2041–60.^91^ By the end of the century, although the amount of newly suitable area would not expand further in the scenario compatible with 2°C of heating, the suitable area would increase to 38% in a scenario with no further mitigation.

*Vibrio* pathogens are ubiquitous in coastal brackish waters, and can cause severe and sometimes life-threatening wound, ear, and gastrointestinal infections in people who come into direct contact with them.^168^ This indicator uses a threshold-based model to monitor suitability for the transmission of pathogenic *Vibrio* spp (excluding *V cholerae*) in global sea coastlines—better accounting for salinity this year. The total coastal area environmentally suitable for *Vibrio* transmission increased by 329 km annually since 1982. In 2022, the coastline suitable at any one point was 12·7% higher than in 1982–2010, reaching 9·9% of the global coastline (the third highest level after 2018 and 2008). A record 81 countries showed suitable areas for *Vibrio* through 2022. The total population living within 100 km of areas suitable for *Vibrio* spp, and therefore at risk of transmission, reached a record 1·4 billion people in 2022, 28% above the 1982–2010 average, leading to a record 609 900 estimated vibriosis cases. Under a scenario compatible with limiting temperature rise to 2°C, coastal area suitable for *Vibrio* spp is projected to increase by 17–25% and lead to 23–39% more cases by 2041–60. Under a scenario with no further mitigation, the length of suitable coastline would be 30–34% higher and lead to 45–46% more cases than in baseline years. By 2081–2100, the suitable coastal area is projected to grow by 10–35% under a scenario compatible with 2°C of heating and 64–84% under a scenario with no further mitigation. Correspondingly, the number of cases would increase by 2–22% and 102–140%, respectively.

### Indicator 1.4: food security and undernutrition—headline finding: the higher frequency of heatwave days and drought months in 2021 compared with 1981–2010 is associated with 127 million more people experiencing moderate or severe food insecurity

Globally, 735 million people faced hunger in 2022 and 3·1 billion people (42%) were unable to afford a healthy diet in 2021.^169^ Through multiple and interconnected pathways, climate change is exacerbating food insecurity: by undermining crop yields; affecting the labour capacity of agricultural workers; and threatening the food security of populations dependent on marine resources through coastal sea surface temperature elevation, reduced ocean oxygenation, ocean acidification, and coral reef bleaching;^170–172^ disrupting supply chains; and reducing food access.^173,174^ Minoritised communities, including Indigenous peoples and subsistence farmers, are particularly affected, as their access to primary and traditional food sources can be compromised, resulting in poorer health outcomes.^175–177^ Increased food insecurity can also contribute to malnutrition, which can have irreversible negative effects on child health and development.

**Figure 4:**
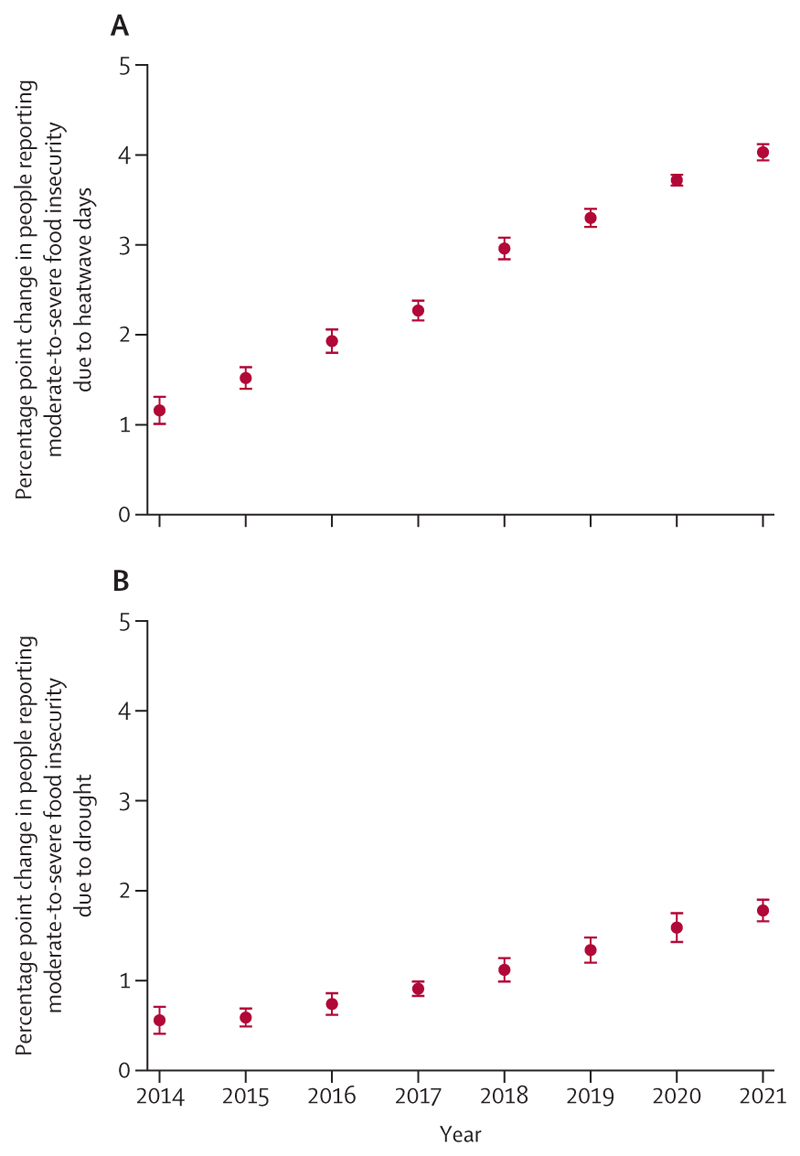
Change in (A) the share of the population (percentage point change) reporting moderate-to-severe food insecurity (as defined by the Food Insecurity Experience Scale) due to heatwave days and (B) the frequency of drought months during the four major crop (maize, rice, sorghum, and wheat) growing seasons from 2014 to 2021

The first part of this indicator combines data from the Food and Agriculture Organization Food Insecurity Experience Scale (FIES)178 from 122 countries (up from 103 countries in the 2022 report of the *Lancet* Countdown) with the frequency of heatwave days and drought months (with the 12-monthly standardised precipitation evapotranspiration index) during the growth seasons of maize, rice, sorghum, and wheat, using a time-varying panel regression. Compared with 1981–2010, a higher number of heatwave days was associated with 4·03 percentage points higher moderate or severe food insecurity (as defined by FIES) in 2021, whereas increasing drought frequency resulted in food insecurity being 1·78 percentage-points higher, equivalent to approximately 127 million more people experiencing food insecurity (figure 4). Under a scenario compatible with limiting temperature rise to 2°C, 524·9 million additional people are projected to experience food insecurity by 2041–60 compared with the 1995–2014 baseline. The global health co-benefits of a scenario compatible with 2°C of heating compared with 3·6°C of heating are projected to include 530 million fewer people experiencing food insecurity by 2041–60, and 1·1 billion fewer people by 2081–2100.

Marine food yields are threatened by sea surface temperature elevation through ocean acidification, coral reef bleaching, reduced oxygenation, and reduced primary productivity.^170,172,179^ An increase in sea surface temperature (SST) is threatening marine food productivity and the livelihoods of many coastal populations.^180^ The second part of this indicator monitors changes in SST—this year with improved geographical and temporal coverage.^181^ The SST in global coastal areas increased by 0·51°C in 2020–22 compared with 1981–2010. SST is projected to increase by 0·99°C by mid-century and by 1·23°C by the end of the century in a scenario compatible with limiting global mean atmospheric heating to 2°C. SSTs are projected to rise even further under a scenario in which no further mitigation occurs, reaching 1·15°C by mid-century and 2·64°C by the end of the century.^182^

## Conclusion

Exposure to climate-related health risks and their impacts is increasing, including from exposure to extreme heat (indicators 1.1.1–1.1.5), wildfire danger (indicator 1.2.1), environmental suitability for infectious diseases (indicator 1.3), and fewer safe hours to work or exercise outdoors (indicators 1.1.3 and 1.1.4). Populations are increasingly exposed to a multitude of greater climate risks that lead to worsening health outcomes. The inclusion of projections for the first time in this year’s report makes clear the potential benefits of more rapid mitigation to limit temperature rise to 1·5°C, and the clear need for (and importance of) increasing adaptation efforts, underlining the health harms that can be avoided by meeting the goals of the Paris Agreement. Ongoing efforts are focused on improving the attribution of observed changes in health hazards and risks to anthropogenic climate change.

Despite efforts to develop an increasingly comprehensive assessment of the evolving threats and impacts of climate change on human health, limitations in data availability and modelling mean that multiple gaps remain. Previous *Lancet* Countdown reports covered the link between climate change and mental health in panels, but efforts are ongoing to advance the development of an indicator to cover this crucial aspect of climate change and health. In addition, the scarcity of data disaggregated by gender, ethnicity, race, indigeneity, migrant status, and other characteristics limits our capacity to track the impact of climate change on vulnerable and minoritised groups, and, consequently, our capacity to assess and address growing health inequities. Given the current disproportionate impact of climate change on these groups,^34,183^ reflecting these inequities is a challenge that the *Lancet* Countdown will continue to pursue.

Due to the complexity of systems modelling, the indicators in this section do not examine the potential negative impacts of interactions and synergies among impacts, or social and climate tipping points, which could considerably increase negative effects on human health. Avoiding these extreme risks requires urgent measures to tackle the health harms of climate change by rapidly accelerating both mitigation and adaptation as non-exclusive and essential interventions.

## Section 2: adaptation, planning, and resilience for health

Section 1 shows that health is already being impacted by climate change, and that related hazards will worsen with further climate change. Protecting people against rising risks requires increasing the adaptation of health and health-supporting systems, while simultaneously reducing GHG emissions to keep climate change within the limits of adaptive capacity.^39^ To be effective, adaptation measures should be informed by climate change-health risk assessments, their implementation adequately funded, and their effectiveness in reducing climate change risks evaluated and iteratively improved. With the understanding, conceptualisation, and definition of a path to achieving the Paris Agreement’s Global Goal on Adaptation being set in 2023 as the 2-year Glasgow-Sharm el-Sheikh work programme comes to a close,^184^ prioritising health in these considerations will ensure people are protected in a heating world.

The first group of indicators in this section monitors progress in the assessment and planning of health adaptation; the second group assesses the enabling conditions for health adaptation; and the last set tracks vulnerabilities, risk, and resilience to climate change.^185^

### 2.1 Assessment and planning of health adaptation

To be effective, adaptation plans must be informed by an in-depth understanding of the health risks of climate change across demographics and populations. Indicators in this section monitor how risks are being assessed, and how health adaptation is being planned at national and city levels.

#### Indicator 2.1.1: national assessments of climate change impacts, vulnerability, and adaptation for health—headline finding: in 2022, 11 of the 64 countries that committed to building climate-resilient health systems through the 26th Conference of the Parties (COP26) Health Programme reported having completed a vulnerability and adaptation assessment

The COP26 Health Programme was established in 2021 to support countries in developing health systems that are climate resilient, low carbon, or both. Within the programme, countries commit to conducting climate change and health vulnerability and adaptation assessments and to using these to inform Health National Adaptation Plans (HNAPs) and facilitate access to climate change funding for health.^44,185^ The Alliance for Transformative Action on Climate and Health (ATACH), led by WHO, supports countries with their implementation of these commitments. To date, 64 countries have committed to building climate-resilient health systems through this programme. A baseline review indicates that 11 health vulnerability and adaptation assessments had been completed between January, 2020, and December, 2022. These assessments identify a range of key health risks, with infectious and respiratory diseases the most common among them.

#### Indicator 2.1.2: national adaptation plans for health—headline finding: between 2020 and 2022, four of 64 countries that made COP26 commitments developed or updated HNAPs

HNAPs are a key mechanism for health systems to prepare for the growing climate burden. Adaptation priorities outlined in HNAPs should be informed by vulnerability and adaptation assessments and integrated into national adaptation plans (NAPs). HNAPs aim to mainstream climate resilience across health governance, service delivery, health workforces, finance, health information systems, and essential medicines and technologies.

Of the 64 countries making COP26 commitments, only four developed or updated HNAPs between 2020 and 2022, with funding remaining a key limitation. In response, the ATACH established a Financing Working Group to address this barrier. Progress on funding for the implementation of COP26 commitments will be tracked in future reports.

#### Indicator 2.1.3: city-level climate change risk assessments—headline finding: in 2022, 848 of 898 cities that reported data to the CDP (94%) reported they had completed or were undertaking a city-level climate change risk assessment, up from 713 cities in 2021

Urban centres are home to 55% of the world’s population.^186^ City-level interventions therefore hold a lot of potential in preventing climate change-related health impacts. This indicator evaluates progress in assessing city-level climate change-related health risks using data reported to the Carbon Disclosure Project.^187^

The number of cities reporting that they have completed, are in the process of conducting, or intend to conduct a climate change risk assessment within 2 years increased from 713 cities in 2021 to 848 cities in 2022 (94%, N=898). Of the 50 cities (6%) that reported they were not undertaking a risk assessment, 11 (22%) indicated that this was due to low financial capacity, 12 (24%) that this was due to low technical capacity, and 20 (40%) that this was due to both.

Of the 525 cities that responded to the question, 351 cities (67%) declared concern about climate change impacting public health outcomes and 141 cities (27%) declared concern about their health systems being overwhelmed. The most frequently identified hazards were extreme heat (378 of 525 cities; 72%), heavy precipitation (205 of 525; 39%), and urban flooding (200 of 525; 38%).

Low and medium HDI countries had the highest proportion of cities not intending to undertake a climate change risk assessment (12 of 100 cities, 12%), with financial constraints or poor technical skills given as key contributing factors.

### 2.2 Enabling conditions, adaptation delivery, and implementation

Adaptation measures must protect the populations most at risk from climate change-related health impacts, must be integrated across sectors, and must avoid unintended harm. The following indicators monitor health adaptation and the conditions that enable adaptation, highlighting areas for plausible improvements.

#### Indicator 2.2.1: climate information for health—headline finding: in 2022, 81% (157 of 193) of the World Meteorological Organization’s 193 members reported working with the health sector; the most frequent type of service provided by members was data service (143 of 193 members, 74%)

Climate services for health are essential in helping the health sector conduct research and make climate-informed decisions for planning, preparedness, and responses to climate-sensitive diseases and extreme weather (panel 5). This requires close cooperation between meteorological and health services and support for health services to be able to access, understand, and act upon health-relevant meteorological information.^44^ In 2022, 157 (81%) of the World Meteorological Organization’s 193 members reported working with the health sector. The most frequent types of service provided were data provisioning (143 of 193 members; 74%), climate monitoring (117 of 193; 61%), climate analysis (114 of 193; 59%), climate prediction (99 of 193; 51%), and products tailored to the health sector (96 of 193; 50%). Relationship and capacity building between the health and climate sectors need further investment and development.

#### Indicator 2.2.2: benefits and harms of air-conditioning—headline finding: in 2021, air-conditioning provided cooling in a third of households but consumed about 1900 terawatt hours (TWh) of electricity (approximately the total electricity consumption of India and Brazil combined)

**Figure 5:**
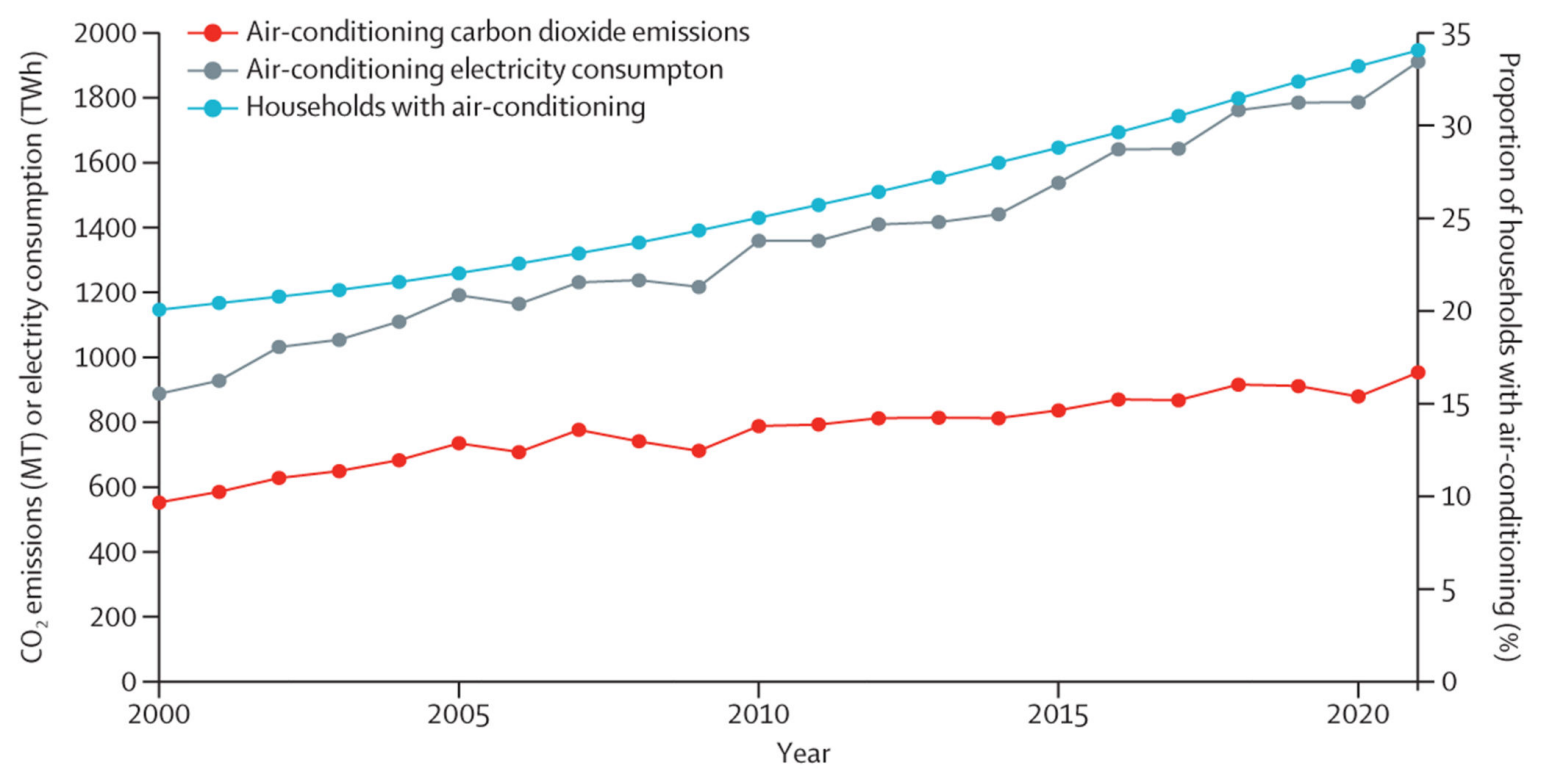
Global household prevalence of air-conditioning, and electricity consumption from air-conditioning and carbon dioxide emissions from air-conditioning MT=megatonne. TWh=terawatt hours.

Air-conditioning can prevent heat-related illnesses and death.^189^ However, it is energy intensive and can exacerbate climate change, air pollution, urban heat islands, energy poverty, and health inequities.^190,191^ According to International Energy Agency data, between 2000 and 2021, the proportion of households with air-conditioning increased by 70%, reaching a third of households. In 2021, air-conditioning consumed roughly 1900 TWh of electricity, over twice its energy consumption in 2000, and approximately the total electricity consumption of India and Brazil combined.^192^ This energy demand strained power grids, caused associated CO_2_ emissions to increase by 73% to almost 1 Gt in 2021, and led to about 23 000 deaths from exposure to associated PM_2·5_ emissions in 2020 (figure 5). In many hot, low-income regions, air-conditioning remains largely inaccessible.^193^ To deliver sustainable cooling for all, over 30 countries have completed or are developing National Cooling Action Plans.^194^ The Cool Coalition recommends an integrated approach, including passive cooling, highly efficient active cooling, climate-friendly refrigerants, and expanded access to cooling for all, but only where and when it is needed.^195^

#### Indicator 2.2.3: urban greenspace—headline finding: the proportion of urban centres with moderate or higher levels of greenness decreased from 18% in 2015 to 13% in 2022 in low HDI countries, with little variation across other HDI groups

Green spaces can reduce the intensity of heat at the neighbourhood scale in urban centres, while positively affecting physical and mental health, providing local improvements in air quality, and helping reduce the risk of urban floods by reducing water runoff.^196–200^ This indicator uses satellite measurements of vegetation (measured with the normalised difference vegetation index [NDVI]) overlayed with population data to estimate greenspace exposure for 1041 urban centres with over 500 000 inhabitants across 174 countries. Global urban greenness has remained low (mean NDVI=0·34) since 2015. In very high HDI countries, 36% of urban centres had at least moderate levels of greenness, against 18% in high HDI countries, 36% in medium HDI countries, and 13% in low HDI countries. Concerningly, low HDI countries are the only HDI group to experience a reduction in exposure to moderate or higher levels of urban greenspace, with a drop from 18% in 2015 to 13% in 2022.

#### Indicator 2.2.4: global multilateral funding for health adaptation programmes—headline finding: $1·16 billion of Green Climate Fund (GCF) financing was dedicated to adaptation projects in 2022, with 17·66% ($205 million) going towards projects with potential health benefits

Financing is a key mechanism of country-level health adaptation; multilateral funding organisations can provide meaningful financing contributions to countries’ health-related adaptation efforts.^2^ This indicator reports on multilateral funding assigned by the Green Climate Fund (GCF), one of seven multilateral funding mechanisms under the UNFCCC,^201,202^ for health-related adaptation projects. In 2022, 20 projects on adaptation, or on both adaptation and mitigation, were approved for a total of $1·16 billion. Of this funding, 17·66% ($205 million) went to projects with potential health benefits, with 12 of the 15 health-specific projects focusing on improved water and food security. As this indicator considers only GCF funding, it does not capture all climate funding, but does provide a representative sample of funding priorities.

#### Indicator 2.2.5: detection, preparedness, and response to health emergencies—headline finding: 126 of 180 countries reported high to very high implementation status for health emergency management in 2022

With climatic suitability for the transmission of multiple infectious diseases increasing in many locations (indicator 1.3), reducing the risk of outbreaks and epidemics requires robust health emergency preparedness and response systems. This indicator reports on the implementation of the legally binding International Health Regulation (IHR)’s core capacities on health emergency management.

In 2022, 126 of 180 (70%) countries reported high to very high implementation (capacity score of 61–100) of health emergency management. However, although 85% of very high HDI countries reported high to very high implementation, only 44% of low HDI countries and 54% of medium HDI countries did.

The Strategic Partnership for Health Security and Emergency Preparedness Portal tracks progress and gaps in IHR implementation through independent external evaluations, simulations, post-review exercises, and the development of national action plans for health security and resource mapping.^203^ Since 2020, 25 country-level after-action reviews have been conducted, of which eight covered epidemics and pandemics, eight covered human-induced events, and nine covered extreme natural events.

Following the COVID-19 pandemic, a review of the IHR identified over 300 possible amendments to strengthen country capacity and compliance. A final proposal on IHR amendments will be presented at the 2024 World Health Assembly.^203,204^

### 2.3 Vulnerabilities, health risk, and resilience to climate change

Although mitigation can reduce climate change hazards, adaptation measures seek to equitably manage climate risks. The following three indicators monitor changing vulnerabilities to climate hazards and health adaptation progress and gaps.

#### *Indicator 2.3.1: vulnerability to mosquito-borne disease—headline finding: low HDI countries experienced a 37% decrease in vulnerability to* Aedes*-borne disease between 1990 and 2021, partly due to improvements in access to health care*

The spread of *Aedes*-borne diseases is rapidly increasing, fuelled by climatic changes (indicator 1.3), the movement of people, and the population density and infrastructure conditions associated with urbanisation.^205^ Reducing vulnerabilities to their most adverse health outcomes is essential to minimising health risks. This indicator captures relative vulnerability to severe *Aedes*-borne disease outcomes, by combining increased susceptibility from urbanisation^205,206^ and coping capacity from improved health-care access and quality.

Vulnerability to severe disease outcomes increased by 6·6% in very high HDI countries from 1990 to 2021, primarily driven by a 9% increase in urbanisation. In low HDI countries, health-care access and quality improved by 48% over the same period, driving a 37% reduction in *Aedes*-borne disease vulnerability. City-level dengue transmission risk assessments, improved waste and territorial management, and operationalised early warning systems are increasingly necessary to reduce the disease burden of *Aedes*-borne diseases.

#### Indicator 2.3.2: lethality of extreme weather events—headline finding: the lethality of floods and storms decreased significantly in high and very high HDI countries between 1990–99 and 2013–22

The frequency, intensity and duration of extreme weather events is increasing worldwide as a result of anthropogenic climate change.^39,59^ Well implemented adaptive measures can, however, avoid a proportional increase in deaths.^207^ This indicator tracks the changing risk of death from climate-related extreme events (defined as the proportion of people affected that died in the event) and the proportion of climatic events that were deadly using the Centre for Research on the Epidemiology of Disasters’ emergency events database (EM-DAT). The risk of death from floods or storms did not change significantly after 1990 across any world region or HDI group. However, the lethality of floods and storms declined on average from 86 deaths to 16 deaths per event in high HDI countries (p=0·007) and from 11 deaths to 8 deaths in very high HDI countries (p=0·02) between 1990–99 and 2013–22, while remaining statistically unchanged in other groups.

#### Indicator 2.3.3: rising sea levels, migration, and displacement—headline finding: in 2022, 153·8 million people were living less than 1 m above current sea levels

Global mean sea level increased 4·68 mm per year between 2013 and 2022, and is projected to reach 0·29–1·10 m by 2100 (relative to 1986–2005), depending on emission scenarios and environmental responses.^208–210^ Sea level rise can affect human health through episodic flooding, permanent inundation, erosion, soil and drinking water contamination, vector-borne and waterborne disease, and mental health impacts.^211–214^ Using land elevation and population data, this indicator estimates that 153·8 million people were living less than 1 m above sea level in 2022, an increase of 7·9% from 2010.

In many cases, populations can adapt in situ to climate hazards such as those of sea level rise. However, diverse (including environmental) factors can push people to relocate (forced or otherwise) or render them immobile. Different forms of human mobility (which includes planned relocation, circular labour mobility or seasonal migration, temporary and permanent migration, short-term evacuation, or trapped and immobile people) are influenced by a complex interplay of sociocultural, political, psychological, economic, demographic, and environmental factors.^215^ Migration can bring varied benefits and difficulties for migrants and other populations in sites of origin, migration routes, and destinations. As of December, 2022, 52 policies connecting climate change and migration were identified across 40 countries. No policy mentioned immobility,^216^ and they tended to refer to health issues, even if peripherally, without explicitly connecting health to climate change and migration—or explaining why links might not exist. Little engagement with or basis on science is evident across the policies.

### Conclusion

Indicators in this section show that health adaptation, which is essential to minimising the impacts of climate change, continues to be insufficient. Low and middle HDI countries are often lagging, with scarce funding continuing to be a barrier (indicators 2.1.1–2.1.3, 2.2.4–2.2.5).

Scarce planned action has also resulted in maladaptation. The use of electricity for air conditioning increased 70% from 2000 to 2021, pushing associated emissions up by 73% and contributing to 23 000 deaths from PM^2·5^ in 2020 alone (indicator 2.2.2). Meanwhile, urban greenness remains low globally, exposing unleveraged potential for sustainable cooling with multiple health co-benefits (indicator 2.2.3). As climate change impacts increase, the cost and challenges of adaptation will continue to rise steeply, leaving reduced scope for averting rapidly accelerating health harms.

However, there are some signs of progress. A rapidly growing number of countries are committing to delivering resilient, sustainable health systems (indicators 2.1.1 and 2.1.2), more cities identify and assess health risks yearly (indicator 2.1.3), and health system strengthening reduced vulnerability to mosquito-borne diseases, particularly in the most exposed countries (indicators 2.3.1 and 2.3.3).

With further climate change now unavoidable, adaptation needs will keep increasing, and adaptation efforts must be urgently enhanced to protect the health of all populations. The key to success will be fostering global knowledge exchange between different actors, particularly focusing on learnings from low HDI countries, and harnessing the wealth of knowledge of Indigenous peoples.^217^ As the challenges to adaptation increase, monitoring progress will become increasingly important. Despite the refinement of indicators over the past 7 years, the challenge of capturing effective adaptation remains, and many indicators rely on self-reported data, which are inherently biased. In addition, adaptation efforts led by grassroots organisations, communities, or civil society are still not being recorded in a standardised manner, limiting the capacity to monitor progress at a global level. Future efforts will focus on assessing the effectiveness of adaptation and capturing its impact on different communities—although scarce reported data is likely to limit the capacity to effectively monitor adaptation.

## Section 3: mitigation actions and health co-benefits

In recognition of the urgent need to accelerate climate action in this crucial decade, the Mitigation Work Programme was established at COP27 to urgently scale up mitigation ambition and implementation. Advancing mitigation actions holds the potential to enable us to avoid the most catastrophic human impacts of climate change and improve the environmental and socioeconomic conditions that good health requires.

This section documents progress on climate change mitigation and its health implications. It explores the health impacts of the existing energy system and its current level of transition. This section then explores the impacts of changes in outdoor and indoor air pollution exposure with an improved indicator monitoring the health impacts of dirty household fuels. It highlights the health burden and emissions associated with existing diets and with the health-care sector.

### 3.1 Energy use, energy generation, and health

The energy system is the world’s biggest single contributor to GHG emissions. The global energy crisis and an increase in the number of people without access to electricity highlights the urgent need to transition away from fossil fuels and towards more equitable and decentralised renewable energy.^80^ This section monitors mitigation progress in the energy sector and its potential health co-benefits.

#### *Indicator 3.1.1: energy systems and health—headline finding: CO*_*2*_ *emissions from the global energy system increased by 0·9% in 2022 due to reopening economies following the lift of COVID-19-related restrictions*

Despite renewable energies becoming increasingly cost-competitive,^218^ fossil fuels contributed 80% of the global total energy supply in 2022, with little change in this proportion since 1990.^219^

**Figure 6:**
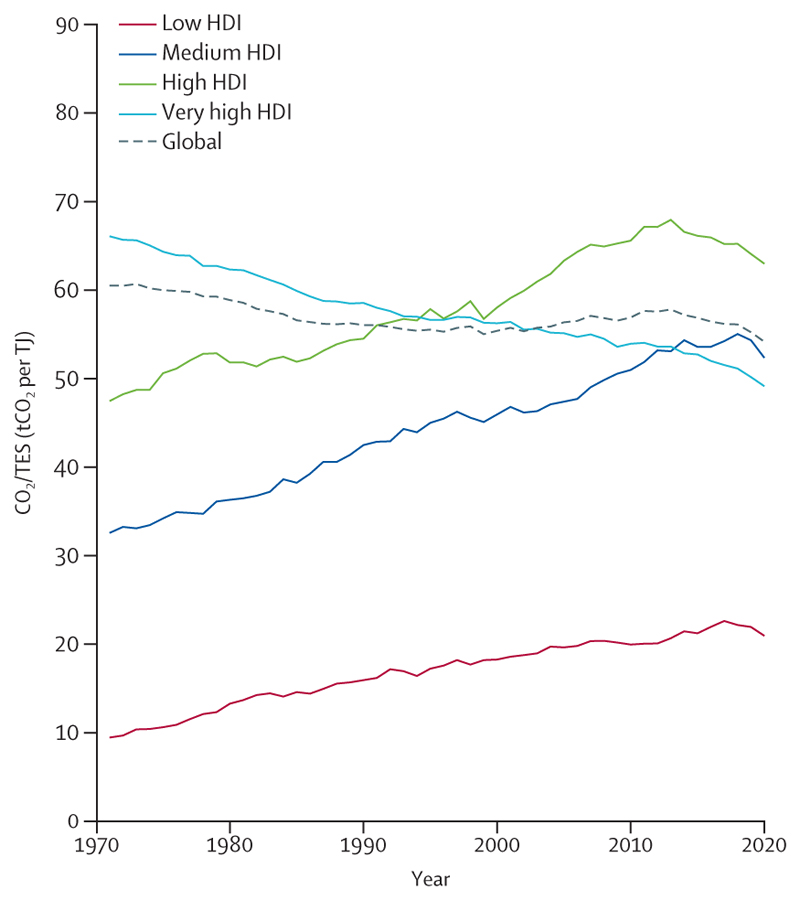
Carbon intensity of the energy system both globally and by HDI country group (tCO_2_/TJ) HDI=Human Development Index. tCO_2_=tonnes of CO_2_. TES=total energy supply. TJ=terajoule.

The CO_2_ emissions intensity of the global energy system fell by only 0·87% between 2015 and 2020, although it experienced a 1·9% reduction to 54·2 tCO_2_/terajoule (TJ) in 2020, driven by transport restrictions during the COVID-19 pandemic (figure 6). However, this reduction was reversed following a lifting of restrictions worldwide, with global energy-related carbon emissions rising by 0·9% in 2022.^220^

An analysis of this indicator by HDI country grouping reflects global inequities in the transition to healthy, clean fuels. The very high HDI country group, which has better access to renewable energy technologies, has been making steady but insufficient progress towards transitioning, with a decadal reduction in the carbon intensity of their energy systems of 2·9 tCO_2_/TJ. Meanwhile, with rapid industrialisation, the carbon intensity in medium HDI countries has increased at a rate of 4·7 tCO_2_/TJ per decade. Low HDI countries, which have little industrial development and show an over-reliance on biomass burning, have the lowest carbon intensity of all (21 tCO_2_/TJ in 2020), which is rising by 2·5 tCO_2_/TJ per decade.

Global use of coal in the energy system, a major contributor to air pollution and GHG emissions, has remained above 150 exajoules since 2010, accounting for 26·7% of total energy supply in 2020. Despite commitments at COP27 to “[accelerate] efforts to phase down unabated coal power”,^184^ the number of coal-fired power stations grew in 2022, and 59% of all newly commissioned capacity occurred in China.^221,222^ Coal burning was responsible for 560 000 deaths related to PM_2·5_ exposure in 2020 (indicator 3.2.1). The long-term health impacts of coal-derived air pollution will be claiming lives for decades, unless urgent action is taken to phase out coal.

The share of modern renewables in electricity generation (mainly wind and solar energy) reached 9·5% in 2020, an increase of 360% compared with 2010. Modern renewables accounted for 90% of new electricity capacity in 2022.^220^ However, limiting temperature increase to 1·5°C would require an annual growth rate of renewables 13 times what it is now, and renewables must make up 77% of the world’s primary energy supply by 2050.^42^ Crucially, although modern renewables make up 11% of all electricity generated in very high HDI countries, they only account for 2·3% of electricity in low HDI ones. The shock in global fossil fuel prices in early 2022 and the lower price of renewable energy might bolster a shift to healthy, more secure, and sustainable sources.^223^ Nonetheless, the economic crisis and high costs of capital are making renewable technologies increasingly unaffordable in lower-income countries, potentially increasing inequities in the adoption of these clean technologies.^224^

Accelerating efforts to phase out fossil fuels in favour of energy efficiency and decentralised modern renewable energy can help expand electricity access in remote and low-resourced areas—reducing energy poverty and enabling universal access to quality health care (panel 6). In pursuing these efforts, particular care is needed to avoid perpetuating harmful extractive industrial practices that disproportionately affect the health of minoritised communities, and act to amplify—rather than reduce—global health inequities.

#### Indicator 3.1.2: household energy use—headline finding: globally, only 32% of the domestic energy used per person is non-polluting at the point of use, whereas 92% of the energy used in low HDI houses comes from polluting biofuels

Access to stable, non-polluting energy is crucial for advancing health and wellbeing.^231–233^ The number of people without access to electricity worldwide is set to increase in 2022 to 775 million.^224^ Most of these people live in sub-Saharan Africa and South Asia. This indicator draws on data from the International Energy Agency (IEA) to monitor the sources of energy used in people’s homes. Globally, only 32% of the domestic energy used per person is non-polluting at the point of use (electricity, district or geothermal heat energy, or solar thermal energy), whereas 31% is solid biomass (wood and dung), and 35% is fossil fuels. However, big global inequities exist. Biomass burning is highly polluting, and accounts for 92% of the household energy in low HDI countries compared with 7*·*5% of household energy in very high HDI countries. Meanwhile, non-polluting energy use has been growing rapidly in high (353% higher in 2020 than in 1990) and very high (49% higher in 2020 than in 1990) HDI countries. The overall use of energy also reflects global inequities, with people in very high HDI countries using, on average, 152%, 322%, and 106% more energy in their homes than people in high HDI countries, medium HDI countries, and low HDI countries, respectively.

#### Indicator 3.1.3: sustainable and healthy road transport—headline finding: despite record growth in electric vehicle sales, fossil fuels still account for 95% of all road transport energy

Global transport emissions increased by 8% in 2021 following the easing of COVID-19 restrictions, and transport-derived PM_2·5_ is responsible for up to 460 000 deaths annually (indicator 3.2.1).^234^ The IEA estimated that electric vehicle sales exceeded 10 million in 2022 (14% of all new cars sold, an increase of 9% from 2021).^235^ Yet maximal health benefits will only be attained by encouraging urban morphologies that support increased walking and cycling, and by reducing particulate air pollution emitted by vehicle brakes and tyre wear.^234^ Meanwhile, interventions to expand safe public transport could reduce the estimated 1·3 million annual deaths occurring annually worldwide from passenger vehicle accidents, while increasing active travel could contribute towards avoiding some of the 3·2 million annual deaths attributable to insufficient physical activity.^236,237^

Drawing on data from the IEA,^238^ this indicator reports that, despite a temporary reduction in road transport energy use due to COVID-19 lockdowns in 2020, the global use of electricity for road transport per capita increased by 97% between 2015 and 2020. However, the global proportion of total per capita road transport energy increased at a rate of only 0·04 percentage points each year. Fossil fuels still account for around 95% of all road transport energy, and total energy use in the transport sector has increased since 1990.

### 3.2 Air pollution and health co-benefits

Exposure to air pollution increases the risk of respiratory and cardiovascular disease, cancer, diabetes, neurological disorders, and adverse pregnancy outcomes.^239^ Many of the major sources of GHG emissions also contribute substantially to air pollution. These indicators track the health burden of energy sector air pollution and the potential health co-benefits of mitigation efforts that prioritise improved air quality.

#### Indicator 3.2.1: mortality from ambient air pollution by sector—headline finding: the number of global deaths attributable to fossil-fuel-derived PM_2·5_ decreased from 1·4 million in 2005 to 1·2 million in 2020; reduced coal pollution contributed to about 80% of the decrease

With extended temporal coverage this year, this indicator estimates the mortality attributable to ambient PM_2·5_, using the greenhouse gas–air pollution interactions and synergies (or GAINS) atmospheric model.^240^

**Figure 7:**
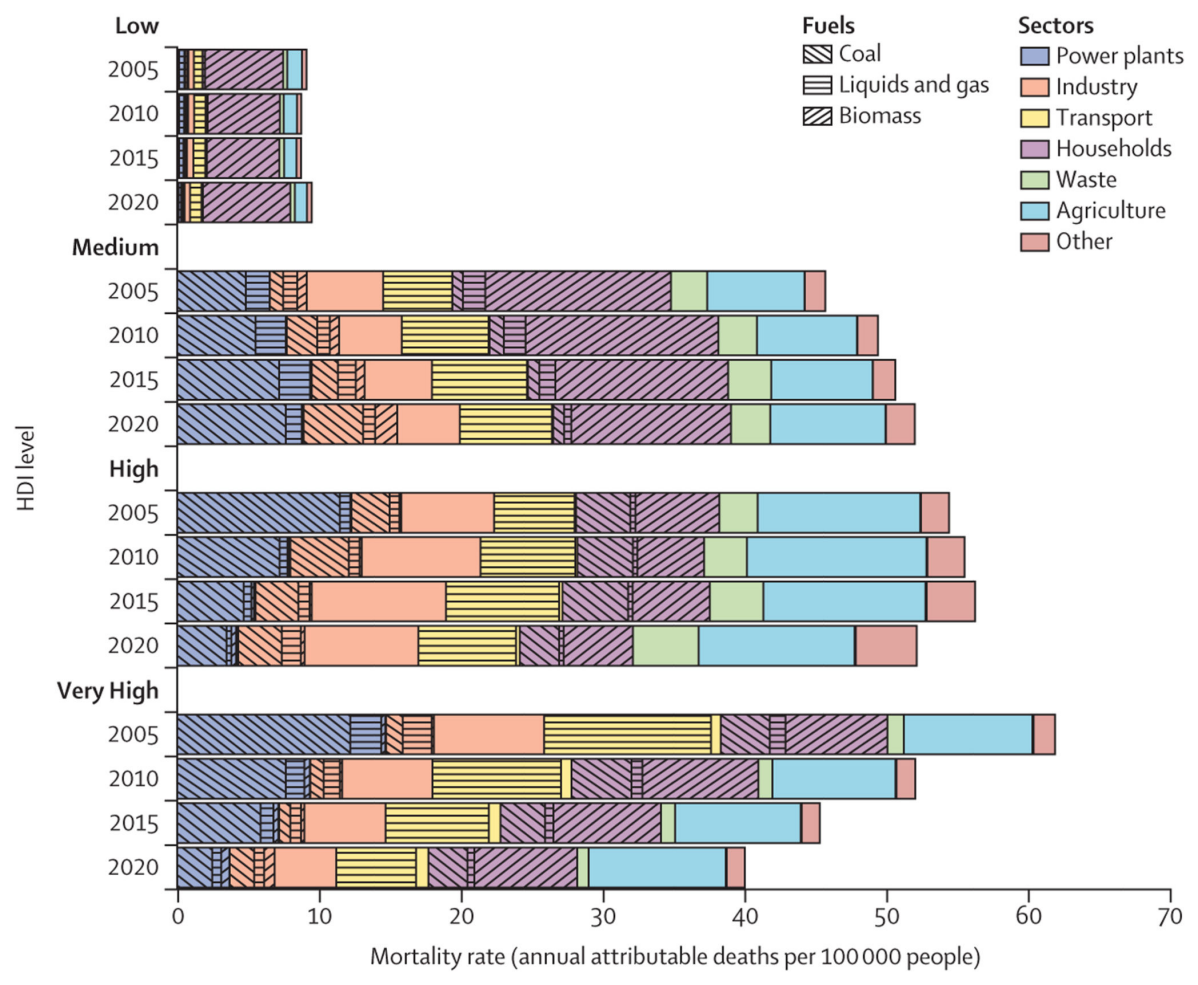
Mortality rate attributable to PM_2·5_ concentration by fuel, sector, year, and HDI country level HDI=Human Development Index.

In 2020, anthropogenic PM_2·5_ was responsible for 3·3 million deaths globally. Deaths attributable to fossil-fuel-derived PM_2·5_ decreased from 1·4 million in 2005 to 1·2 million in 2020 (15·7%). Of this reduction, 80% was due to reduced coal-derived air pollution (figure *7*). However, coal still contributed to 53% (640 000) of all deaths attributable to fossil-fuel-derived PM_2·5_ in 2020. Meanwhile, biomass burning contributed to 653 000 deaths. In the high and very high HDI groups, exposure to PM_2·5_ decreased from 2005, due to emission controls and fuel switching. However, deaths have increased in medium HDI countries, where access to non-polluting energy and air quality control measures are lagging.

#### Indicator 3.2.2: household air pollution—headline finding: the use of polluting fuels resulted in 140 deaths per 100 000 associated with household air pollution in 2020 in 62 countries reviewed, 56% of which were due to the use of solid fuels

Globally, 2·4 billion people still use polluting and inefficient fuels and technologies for cooking, and 733 million people live without electricity.^81^ The use of dirty fuels in the household sector results in exposure to toxic concentrations of air pollution inside people’s homes and is responsible for 55% of anthropogenic ambient PM_2·5_ air pollution in low HDI countries, virtually all of which (97%) comes from biomass burning (indicator 3.2.1). The surge in energy prices and economic pressures from energy and economic crises could mean that 100 million more people revert to burning biomass. With women and girls often tasked with household energy-related activities, the burden of disease associated with air pollution from dirty fuels in the domestic sector falls disproportionately on them.^241,242^ In addition, women and girls are often tasked with searching for biomass, which exposes them to violence and injuries and, due to the time allocated to these activities, limits their capacity to access education, economic independence, and personal growth activities.^241,242^ Paucity in the adoption of clean fuels in the domestic sector is therefore underpinned by, and exacerbates, gender health inequities and injustices.

This indicator estimates exposure to PM_2·5_ derived from household air pollution in 62 countries. It uses a Bayesian hierarchical model developed with sample data on indoor air quality from 282 peer-reviewed studies and accounts for sociodemographic and epidemiological characteristics,^38,243,244^ and estimates attributable mortality through a comparative risk assessment.

In the 62 countries analysed, household air pollution led to 140 deaths per 100 000 in 2020. Roughly half of their total population used dirty fuels (biomass, charcoal, or coal) as the primary source of energy for cooking and heating in 2020, representing most of the global population reliant on dirty fuels. This use resulted in 151 µg/m^3^of PM_2·5_ (95% CI 133–169) on average inside their homes. Within this population, rural households had 171 µg/m^3^of PM_2·5_ (153–189) on average, and urban households 92 µg/m^3^ of PM_2·5_ (77–106). This household air pollution is estimated to have contributed to an average of 78 deaths per 100 000 (95% CI 69–87), with a rural average of 82 (73–90) and an urban average of 66 (57–75) deaths per 100 000.

Shifting to less polluting fuels, such as biogas, natural gas, liquefied petroleum gas, and alcohol fuel (as well as solar and electric energy), can help reduce this health burden. However, combustion of these fuels still resulted in the population primarily reliant on them (roughly 50·1% of the total population of the 62 countries analysed) being exposed on average to 69 µg/m^3^ (95% CI 62–76) of PM_2·5_, with a rural average of 76 µg/m^3^(69–83) and an urban average of 49 µg/m^3^ (46–53). The associated mortality is estimated at 62 deaths per 100 000 (95% CI 54–70), with a rural average of 66 (58–74) and an urban average of 52 (47–57) deaths per 100 000. These data reflect the health impacts of so-called transition fuels (natural gas and liquefied petroleum gas) and biogas, and underline the untapped potential offered by expanding access to non-polluting, renewable energy to improve health, reduce health inequities, and minimise energy poverty.

### 3.3 Food, agriculture, and health co-benefits

Food systems contribute around 30% of global GHG emissions, remaining incompatible with mitigation targets.^245,246^ This section monitors the potential for mitigation and health co-benefits in the agriculture and food sector.^247^

#### Indicator 3.3.1: emissions from agricultural production and consumption—headline finding: global agricultural emissions increased by 22% from 2000 to 2020, with red meat and dairy responsible for 57% of agricultural emissions in 2020

This indicator shows that, although agricultural emissions per person remained stable at approximately 0·9 tonnes of CO_2_ equivalent (tCO_2_e), the growing population increased emissions by 22% from 2000 to 2020. In 2020, roughly 57% of agricultural emissions were the result of red meat and dairy production.

**Figure 8:**
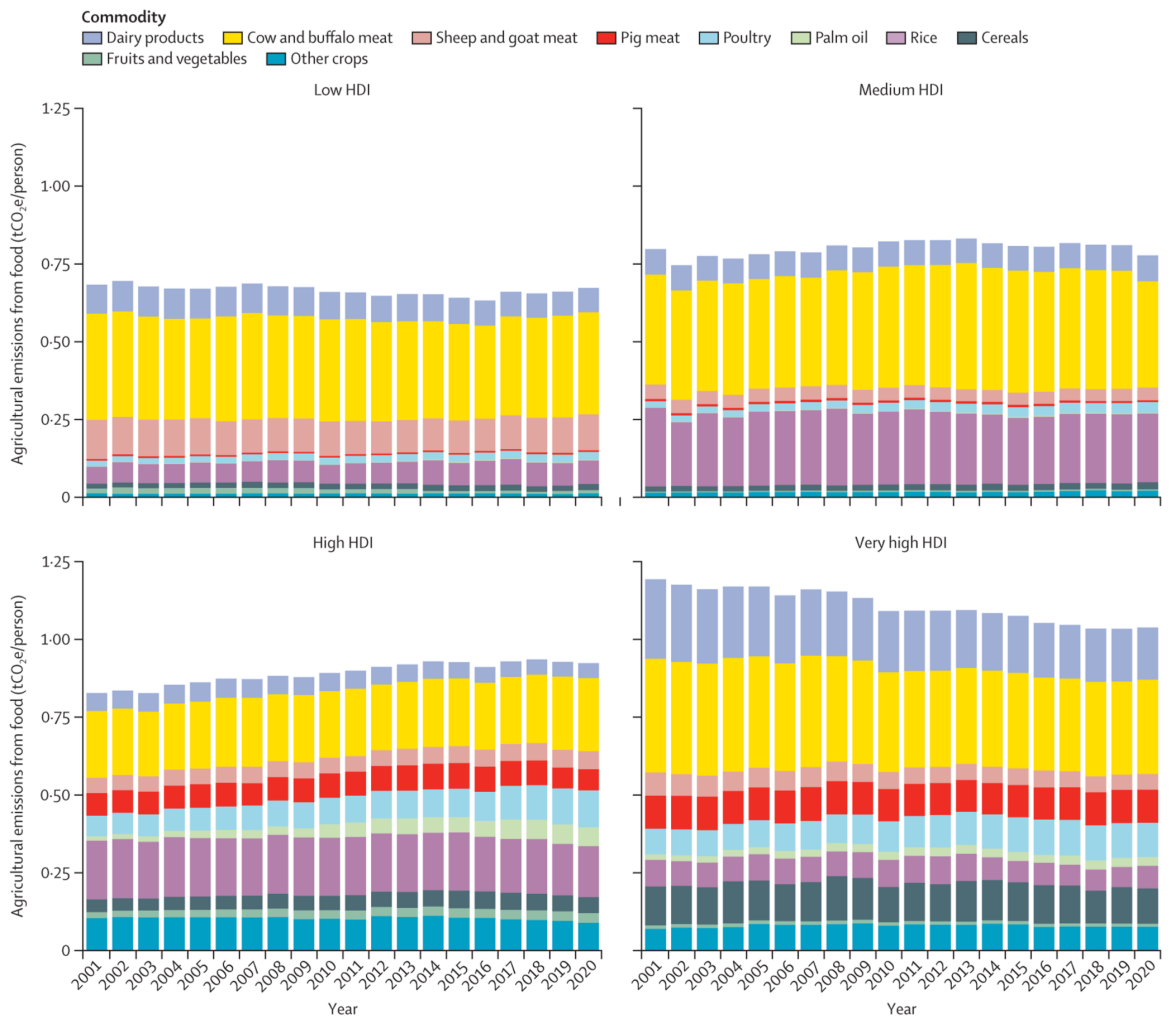
Emissions of greenhouse gases on farms associated with the consumption of agricultural products (production and net imports) per person by HDI level HDI=Human Development Index. tCO_2_e=tonnes of CO_2_ equivalent.

Consumption-based emissions per person in 2020 were 43% higher in the very high HDI country group than in the low HDI group, revealing inequities in the global food system. However, agricultural practices and environmental pressures in low HDI countries, notably in Africa, led to low productivity in animal rearing and resulted in high emissions per unit of consumption (figure 8).^248,249^ As food systems become increasingly strained by environmental changes, supporting healthy, low-carbon diets will require shifts towards less polluting and more inclusive and resource-efficient foods and food production systems, with sustainable management practices and a reduced reliance on fossil fuels.^250,251^ This shift will require robust regulation of the agricultural and food industries, protecting smallholder farmers and Indigenous food systems, and promoting equitable and inclusive access to agricultural technology that aligns with local cultures and beliefs.^251^ Harnessing and integrating the knowledge and technologies of traditional farmers and Indigenous peoples in the sustainable management of natural agricultural resources can offer particular benefits in the transition to low-carbon, sustainable, and efficient food systems.^252^

#### Indicator 3.3.2: diet and health co-benefits—headline finding: in 2020, 7·8 million deaths were associated with insufficient consumption of nutritious plant-based foods, and 1·9 million deaths were associated with excessive consumption of dairy, and red and processed meat

Suboptimal diets are a leading risk factor for non-communicable diseases globally.^247^ Promoting shifts to healthier, more plant-based diets, can substantially reduce GHG emissions, while also delivering major benefits for public health.^253^

**Figure 9:**
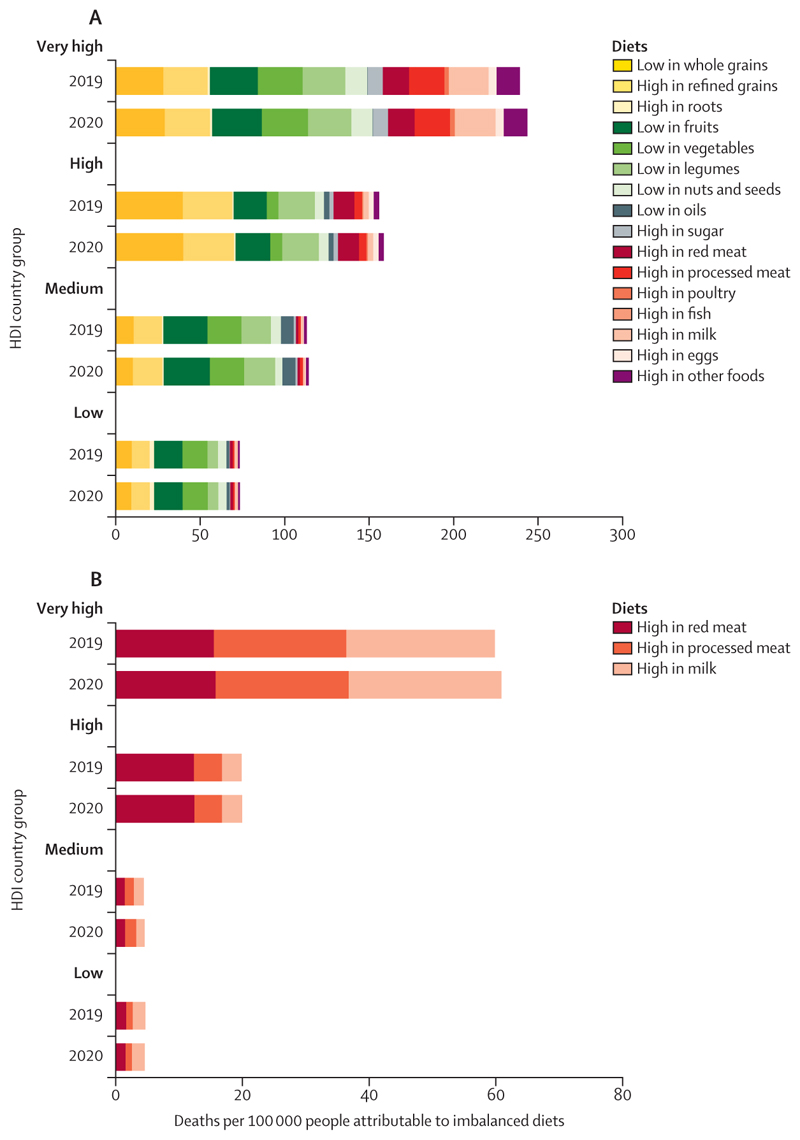
Deaths per 100 000 inhabitants attributable to dietary risk factors by HDI country group (A) Deaths attributable to all dietary risk factors. (B) Deaths attributable to excess consumption of red and processed meat. HDI=Human Development Index.

This indicator combines relative risk factors from a regularly updated meta-analysis, with population and mortality data to estimate the annual deaths attributable to dietary risk factors (appendix p 160). It estimates that 12·2 million deaths in 2020 were attributable to dietary risks that could be reduced through balanced, low-emission diets, an increase of 282 000 deaths from 2019. Of the total, 7·8 million deaths were associated with an insufficient consumption of fruits, vegetables, legumes, wholegrains, nuts, and seeds, whereas an excess consumption of diary and red and processed meat, which contributed to 57% of agricultural emissions (indicator 3.3.1), was responsible for 16% of all diet-related deaths (1·9 million). Very high HDI countries experienced the highest diet-related death rate (deaths per 100 000), 2·4-times higher than the rate in low HDI countries. The very high HDI group also had the highest death rate related to excess dairy and red and processed meat consumption, 6·7-times higher than the average mortality for countries in other HDI groups (figure 9).

#### Indicator 3.4: health-care sector emissions and harms—headline finding: increases in COVID-19-related health-care spending in 2020 counterbalanced decreases in the carbon intensity of electricity, with global health-care emissions remaining at 4·6% of total GHG emissions; health-care-associated PM_2·5_ and ozone were responsible for approximately 4 million Disability-Adjusted Life Years (DALYs) annually

The COVID-19 pandemic response pushed global health expenditures in 2020 to $9 trillion, 11% of gross world product.^254^ Quality health care requires the use of energy, goods, services, and infrastructure, all of which contribute to global GHG emissions. This indicator models emissions from the global health-care sector using environmentally extended multi-region input–output (EE-MRIO) models combined with national health-care expenditure data. New this year, it also estimates health-care-related emissions of PM_2·5_, ozone, and their health damages.

**Figure 10:**
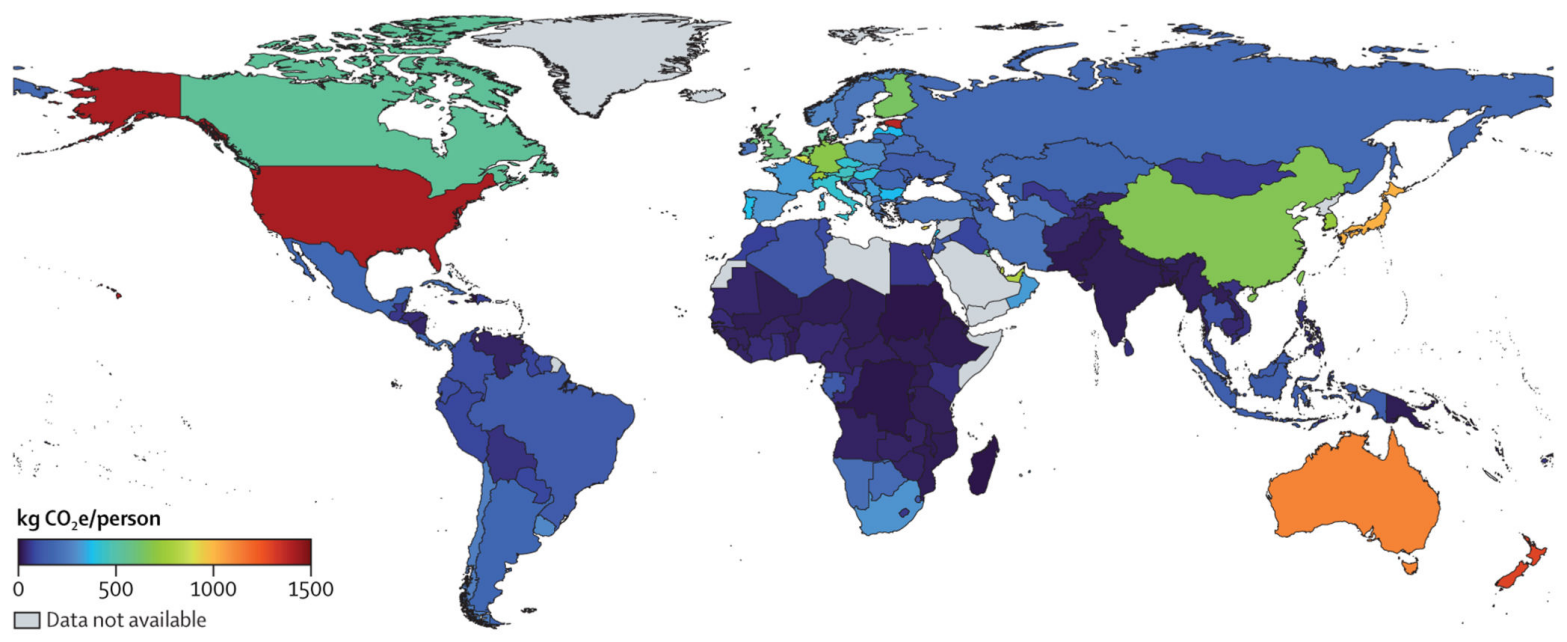
National GHG emissions per capita from the health-care sector in 2020 Data are not available for the countries in grey. CO_2_e=CO_2_ equivalent. GHG=greenhouse gas emissions.

The global health-care sector still contributed around 4·6% of global GHG emissions in 2020, even while absolute emissions decreased by 3·7% from 2019. Globally, air pollution associated with health-care delivery and supply chains contributed to an estimated loss of 4 million DALYs in 2020. More than half of these estimated health harms were due to health-care sector activities in China, whereas 12% are attributable to the USA (figure 10).

### Conclusion

To reach net-zero by 2050, anthropogenic CO_2_ emissions must decrease by roughly 45% from 2010 levels by 2030.^123^ Fossil fuels must be urgently phased out to reach this goal, tackle climate change, and reduce its risk to human health and survival. Yet, they still provide 80% of global energy, 26% of which comes from coal and 68% of household energy still comes from polluting fuels (indicator 3.1.2). Due to the global energy and economic crises, 100 million people risk returning to biomass for fuel, and many countries risk turning or are already turning to coal.^223^ Agricultural sector emissions add to the problem, increasing by 22% from 2000 to 2020 (indicator 3.3.1). Although the use of renewable energies is increasing, the pace has so far not been sufficient to curb increasing emissions from growing fossil fuel use (indicator 3.1.1). In addition, global patterns of access to and the deployment of renewable energy technologies, with low HDI countries left behind in the transition, contrast sharply with the availability of natural renewable energy resources.

Urgent mitigation can still keep temperatures within adaptability thresholds, simultaneously delivering health co-benefits in the immediate term. Increasing access to zero-carbon energy can, if delivered with health as a key priority, not only reduce energy poverty, but also improve air quality, saving millions of deaths each year (indicators 3.2.1 and 3.2.2). Transitioning to healthier, low-carbon diets could prevent up to 12·2 million deaths annually (indicator 3.3.2), and shifting to accessible active, public, and electric travel could avoid 460 000 deaths annually from travel-related PM_2·5_ emissions and encourage increased physical activity (indicator 3.1.3). Additionally, these gains would reduce health-care demand, helping minimise health-care-related emissions and their associated health impacts (indicator 3.4).

Realising this ambition requires concerted efforts. Those concerned with protecting health need to actively engage with energy and finance sectors, civil society actors, Indigenous peoples, and minoritised communities to capture interdisciplinary and traditional knowledge and ensure that the health and equity benefits of the zero-carbon transformation are maximised. A just transition must include preventing harmful extractive and exploitative industrial practices, which disproportionately affect minoritised communities and further amplify global health inequities; focusing efforts on ensuring universal access to clean, healthy, zero-emission energy for all; and harnessing Indigenous knowledge and technologies.^252,255^

Monitoring progress towards delivering mitigation ambitions is crucial, particularly in this narrow window of opportunity to keep the goals of the Paris Agreement within reach. The *Lancet* Countdown will continue strengthening its indicators to support countries being held accountable for their mitigation progress, and to identify opportunities for increased health co-benefits from climate change actions. This work will include quantifying progress towards delivering further co-benefits, including that of enhancing active travel, energy efficiency, or the health gains from nature-based solutions, and that of reducing the adverse health impacts of CO_2_-emission-related extractive industries. Importantly, future efforts will also be focused on identifying potential unintended harms stemming from the transition to net-zero emissions, and on capturing the knowledge of minoritised peoples. These challenges will require contributions from the broader scientific community, which the *Lancet* Countdown will continue to welcome and foster.

## Section 4: economics and finance

The delays in climate change action have meant the health impacts of climate change continue to increase (section 1), as do the associated economic health-related losses and damages. Limiting global mean temperature rise to 1·5°C requires rapid decarbonisation in all economic sectors. Although the required up-front investment is substantial, the immediate economic and health co-benefits, alongside the loss and damage avoided, would vastly outweigh these costs.^43,256,257^ With the right incentives, market conditions, and governance, the necessary funds are available. However, the transition will only deliver these health gains if it is just, breaks harmful and entrenched global power dynamics, and improves global health equity.

In 2022, COP27 resulted in a breakthrough agreement on the need for loss and damage funding for the countries most affected by climate change and calls for a transformation of financial systems to support the zero-carbon transition.^258^ The details of the agreement are still to be established,^259^ and negotiations will take place amid a global energy crisis, which is increasing inflation and interest rates globally, saddling governments with debt, and restricting the capital available for new investment while simultaneously stimulating the search for new sources of energy. On an individual level, the energy crisis is further increasing vulnerabilities and climate change impacts by driving a cost-of-living crisis, deepening energy poverty, and exposing the human costs of a fossil-fuel-dependent global energy system.

Indicators in this section explore the economic costs of climate change and monitor progress—or the lack thereof—in the transition to a low-carbon, healthy, and just global economy, understanding that the transition to a net-zero economic system is an essential component of the transition to a healthy, thriving future.

### 4.1 Economic impact of climate change and its mitigation

The health impacts of climate change are causing additional economic losses and damages, including through increased health-care demands and the loss of labour capacity. These losses, in turn, undermine lives and livelihoods, increase the cost of adapting to climate change, and further restrict the resources available to finance accelerated climate change action. Indicators in this section track the economic costs associated with the health impacts of climate change.

#### Indicator 4.1.1: economic losses due to weather-related extreme events—headline finding: global economic losses due to weather-related extreme events were $264 billion in 2022; whereas 57·1% of economic losses in very high HDI countries were insured, 92·8% of losses in other countries were uninsured

The loss of infrastructure and resulting economic losses due to extreme weather events can exacerbate health impacts through disruption of essential services and effects on the socioeconomic determinants of health. This indicator tracks economic losses from weather-related extreme events using data provided by Swiss Re.^260^

From 2010–14 to 2018–22, total measurable annual mean economic losses induced by climate-related extreme events increased by 23% in real terms. The percentage of global losses that were uninsured, however, fell from 67% to 55%. In 2022 alone, weather-related extreme events induced losses of $264 billion, with 78% of these losses occurring in very high HDI countries. These losses were equivalent to 0·32% of GDP in the very high HDI country group compared with 0·16% for other countries, although lower losses in other countries might reflect the lower monetary value of the property damaged rather than the disruption and hardship caused. Whereas 42·9% of losses in very high HDI countries were insured, 99·6%, 96·2%, and 88·8% of losses in low HDI countries, medium HDI countries, and high HDI countries, respectively, were uninsured. These high levels of uninsured losses exacerbate the economic burden of climate change in low HDI countries, with losses being unreplaced or replacement costs falling directly on individuals and institutions.

#### Indicator 4.1.2: costs of heat-related mortality—headline finding: at $164 billion, the average annual monetised global losses due to heat-related mortality for 2018–22 were 146% higher than the 2000–04 average

This indicator estimates the economic value of heat-related mortality losses by combining the years of life lost (YLL) from indicator 1.1.5 and the value of a statistical life year (VSLY). In 2018–22, the average annual monetised heat-related mortality losses were $164 billion, about 0·17% of the average gross world product. This was the highest loss in the past two decades (146% higher than the 2000–04 average) and equivalent to the loss of 12·8 million average incomes (expressed as per-capita GDP). The growth in the value of average heat-related mortality losses from 2000–04 to 2018–22, in terms of average incomes, was the highest in low HDI countries (131%), with growth of 84% in medium HDI countries, 110% in high HDI countries, and 61% in very high HDI countries.

#### Indicator 4.1.3: loss of earnings from heat-related reduction in labour capacity—headline finding: the global potential loss of income from reduction in labour capacity due to extreme heat was $863 billion in 2022; the agricultural sector was the most severely affected, incurring 82% and 68% of the average losses in low HDI countries and medium HDI countries, respectively

Heat exposure endangers the health of workers, reduces labour productivity, and generates income and economic losses, which cascade through the economies of nations. This indicator quantifies the loss of earnings that could result from heat-related labour capacity loss, combining data from indicator 1.1.4 with hourly wage data from the International Labour Organization.

The global potential loss of earnings was $863 billion in 2022, equivalent to 0·87% of gross world product. Low HDI countries and medium HDI countries experienced the highest average income losses, equivalent to 6·1% and 3·8% of their GDP, respectively. Of all global losses, 40% occurred in the agricultural sector, with 31% in construction. Agricultural workers in low and medium HDI countries, often among the world’s poorest people,^261–263^ endured on average 82% and 68% of the losses in those countries, respectively.

#### Indicator 4.1.4: costs of the health impacts of air pollution—headline finding: in 2020, the monetised costs of premature mortality due to air pollution amounted to $2·2 trillion, the equivalent of 2·4% of gross world product

**Figure 11:**
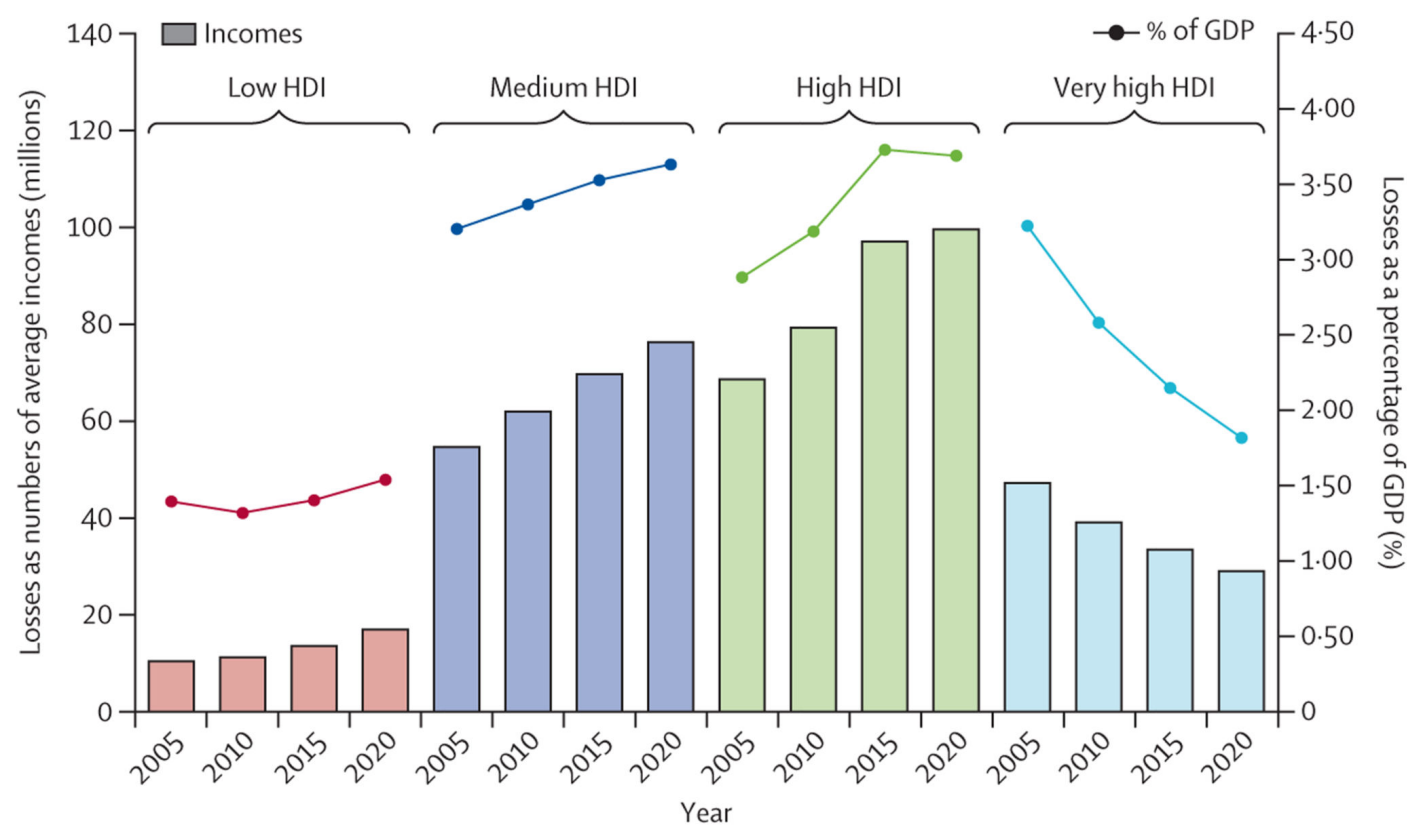
Monetised losses from premature mortality due to air pollution according to HDI group Columns represent losses as numbers of average incomes, lines respresent losses expressed as a percentage of GDP. GDP=gross domestic product. HDI=Human Development Index.

Many of the 3·3 million annual deaths resulting from exposure to anthropogenic PM_2·5_ air pollution, could be avoided through ambitious mitigation (indicator 3.2.1). This indicator places an economic value on YLLs from exposure to anthropogenic ambient PM_2·5_. Although global costs relative to GDP and to average incomes (expressed as per-capita GDP) decreased slightly between 2005 and 2020, in 2020 the total costs amounted to $2·2 trillion, or the equivalent of 2·4% of gross world product. The high HDI country group had the greatest costs relative to per-capita income, equivalent to the annual average income of 99·6 million people in 2020 (figure 11). This group also had the greatest costs relative to the size of its countries’ collective economies in 2020, equivalent to 3·69% of its GDP, closely followed by the medium HDI group (3·63% of GDP). Although the total cost is the highest in very high HDI countries, this cost fell substantially between 2005 and 2020. Moreover, the cost in very high HDI countries relative to their high GDPs (and high average incomes) is lower.

### 4.2 Economics of the transition to net zero-carbon economies

Protecting health from a changing climate and realising the health co-benefits of climate action require a zero-carbon and just transition of the whole economy, involving a rapid decline in the production and use of health-harming fossil fuels. If delivered while keeping people and their health as a core priority, this transition can also result in major health benefits from reduced inequities and improved socioeconomic determinants of health. The following indicators monitor this shift, tracking jobs and investment in zero-carbon energy, fossil fuel divestment, net carbon pricing and subsidising instruments, the effect of global trade on emissions, and the compliance of oil and gas firms with the 1·5°C climate target. Estimates suggest that 70% of the required green energy investment is likely to come from private sources and an increasing amount will be mobilised as debt.^264^ A new indicator on bank financing compares amounts of fossil fuel and green lending, and monitors changes in lending to the fossil fuel sector since the Paris Agreement. Panel 7 explores the impacts of the current geopolitical turmoil on the financing of and energy transition to a zero-carbon future.

#### Indicator 4.2.1: clean energy investment—headline finding: investment in global clean energy increased by 15% in 2022 to $1·6 trillion and exceeded fossil fuel investment by 61%

Investing in zero-carbon energy and energy efficiency is essential for both mitigating climate change and for reducing air pollution. Reaching net-zero emissions can lead to economic growth, which in turn can lead to further investment in clean energy.^257^ Drawing on data from the IEA, this indicator monitors trends in global investment in energy supply and energy efficiency.^275^

Clean energy investment exceeded fossil fuel investment by 61% in 2022. Global clean energy investment in 2022 was 15% higher than in 2021 and 51% higher than in 2015, reaching $1·6 trillion. Meanwhile, fossil fuel investment in 2022 was 10% higher than in 2021, but 24% lower than in 2015, at $1·0 trillion. Energy efficiency accounted for 15% of all energy investment in 2022—the same as in 2021. To fulfil global net-zero emissions by 2050, investment in clean energy needs to nearly triple by 2030, whereas investment in fossil fuels needs to reduce to less than half its current value.^275,287^

#### Indicator 4.2.2: employment in renewable energy and fossil fuel industries—headline finding: direct and indirect employment in renewable energy increased by 5·6% in 2021 to a record high of nearly 12·7 million employees, whereas direct employment in fossil fuel extraction increased by nearly 20%

Transition away from fossil fuels and towards renewable energy can lead to the net generation of employment.^43^ Employees in fossil fuel extraction industries, particularly in coal mining, have a greater risk of non-communicable disease than the general population.^288^ Increasing employment in the renewable energy industry could improve both public health outcomes and livelihoods. This indicator uses data from the International Renewable Energy Agency and IBISWorld to compare employment in renewable energy with employment in fossil fuel extraction.^289–291^

**Figure 12:**
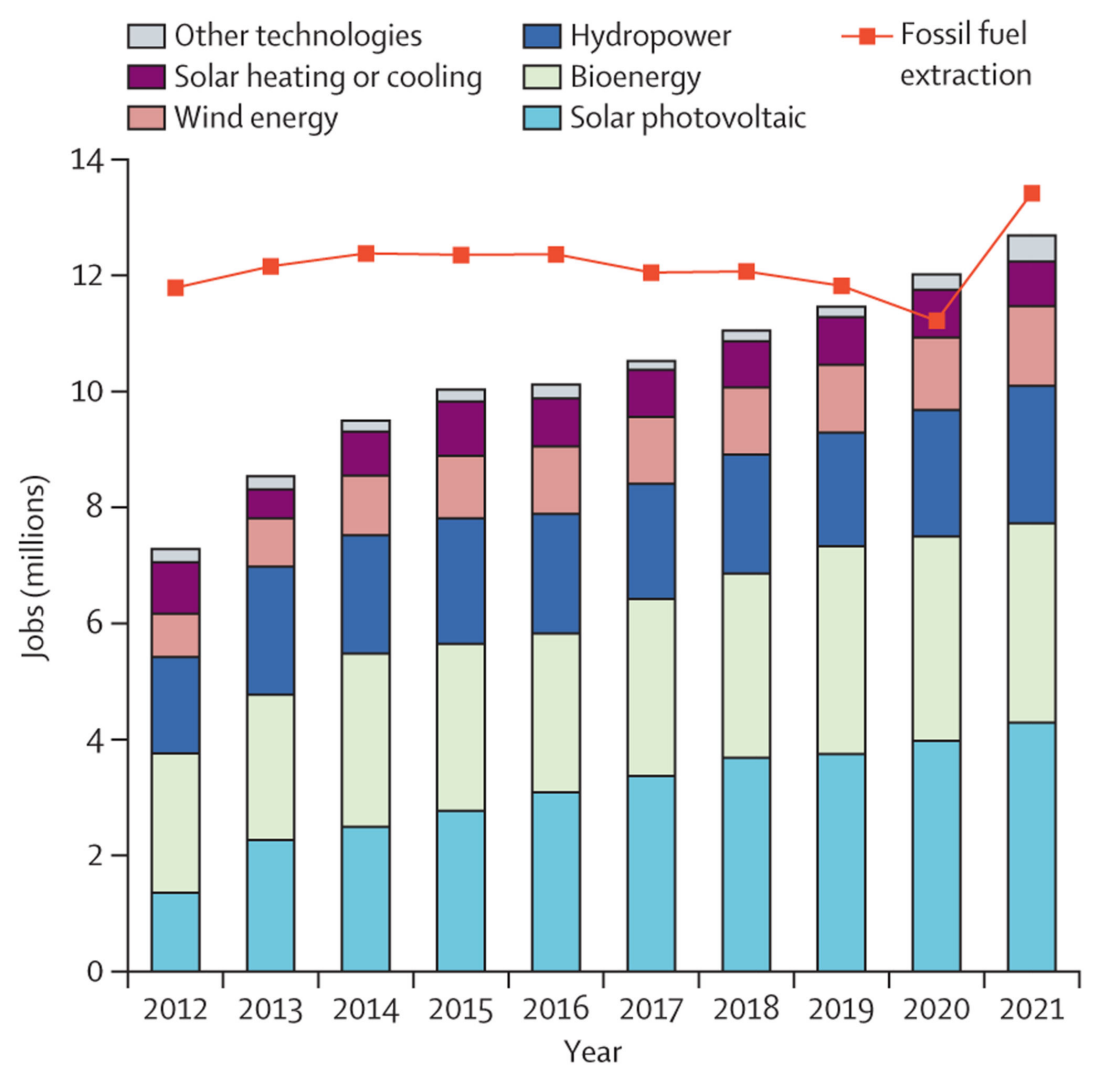
Direct and indirect employment numbers in the renewable energy sector and direct employment numbers in fossil fuel extraction

In 2021, nearly 12·7 million people were employed directly or indirectly by the renewable energy industry (figure 12). This figure marks the largest ever workforce in renewables and represents an increase of 5·6% from 2020 and 74·3% from 2012. Employment in renewables also exceeded direct employment in fossil fuel extraction for the first time in 2020. However, as the global economy recovered from COVID-19, direct employment in fossil fuel extraction rebounded by 19·6% in 2021 to a record 13·4 million people. Nonetheless, over the medium term, employment is growing faster in the renewable energy sector.

#### Indicator 4.2.3: funds divested from fossil fuels—headline finding: between 2008 and the end of 2022, the global value of funds committed to fossil fuel divestment was $40·51 trillion, with health-care institutions accounting for $54 billion

By divesting holdings in fossil fuel companies, organisations can both remove their financial support and reduce their social licence to operate. They can also reduce their risk of losses due to stranded assets in a decarbonising world.^292,293^ Health-care institutions can take a lead in divesting from holdings in fossil fuel companies, thereby advancing their mission to protect health. This indicator tracks the value of funds divested from fossil fuels with data provided by Stand.earth.

From Jan 1, 2008, to Dec 31, 2022, 1558 organisations (with assets worth at least $40·51 trillion) committed to divestment. Only 27 of these organisations are health-care institutions, with assets totalling $54 billion. Of the 49 organisations (with a value of $58·7 billion) that committed to divestment in 2022, none were health-care institutions.

#### Indicator 4.2.4: net value of fossil fuel subsidies and carbon prices—headline finding: 68 (78%) of the 87 countries reviewed had a net-negative carbon price in 2020, generating a net subsidy to fossil fuels of $305 billion; the value of the resulting net subsidies exceeded 10% of national health budgets in nearly 30% of these countries

Carbon prices help economies transition away from high-carbon fuels, whereas fossil fuel subsidies provide incentives for health-harming emissions and slow the low-carbon transition.^82,83^ This indicator compares carbon prices and monetary fossil fuel subsidies to calculate net economy-wide average carbon prices and revenues in 87 countries that emit 93% of global CO_2_ emissions.

In 2020, despite 45 countries having a carbon pricing mechanism in place, simultaneous subsidies meant that only 18 produced a net-positive carbon price—all but one of which were very high HDI countries. 68 (78%) of 87 countries reviewed had net-negative carbon prices (ie, they provided a net subsidy to fossil fuels) for a net total of $305 billion that year alone. The median subsidy value in these 68 countries was $1·3 billion, with eight countries each exceeding $10 billion. Net subsidies exceeded 10% of national health spending in 26 (30%) countries, and 50% in ten countries (11%). COVID-19 restrictions in the highly subsidised transport sector meant the total net subsidy spent was 47% lower in 2020 than in 2019. However, the sharp increase in energy costs that followed is expected to have substantially increased net subsidies in 2021–22.

Redirecting government support from subsidising fossil fuels to incentivising the expansion and affordability of low-carbon power, health protection, public health promotion, and health care, and to providing other means of support to the individuals who might be most affected by potential increases in energy prices would deliver net benefits to health and wellbeing and support a just transition.^294,295^ International financing mechanisms are needed to support low-income countries, which are vulnerable to energy costs, in their transition to sustainable energy sources, particularly in light of the ongoing energy crisis, and to safeguard all dimensions of human health.^295^

#### Indicator 4.2.5: production-based and consumption-based attribution of CO_2_ and PM_2·5_ emissions—headline finding: in 2021, 4·2% of global CO_2_ emissions and 5·2% of global PM_2·5_ emissions occurred in low HDI countries, medium HDI countries, or high HDI countries due to the net import of goods and services consumed in very high HDI countries

Consumption-based emission accounting allocates emissions to countries according to their consumption of goods and services, even when the physical emissions associated with those goods and services occurred abroad. This indicator uses an EE-MRIO model^240,296,297^ to quantify countries’ consumption-based and production-based contribution to CO_2_ and PM_2·5_ emissions.

In 2021, the production of foods and services traded between countries with different HDI levels accounted for 18·9% of global CO_2_ emissions and 18·8% of global PM_2.5_ emissions. The very high HDI group is the largest CO_2_ emitter in both production-based (44·7% of total global CO_2_ emissions) and consumption-based accounting (48·9%), followed by the high HDI group (43·7% and 39·5%, respectively). Furthermore, the net imports of goods and services into very high HDI countries resulted in CO_2_ and PM_2·5_ emissions in countries with lower HDI, accounting for 4·2% and 5·2% of global emissions, respectively. Very high HDI countries were the only group with higher consumption-based than production-based emissions of both pollutants, resulting in pollution in countries with lower HDI levels. Compared with 2020, in 2021, the global share of CO_2_ from both production-based and consumption-based emissions increased in all HDI groups except the high HDI country group.

#### Indicator 4.2.6: compatibility of fossil fuel company strategies with the Paris Agreement—headline finding: the strategies of the 20 largest oil and gas companies as of February, 2023, would lead to production exceeding their share of levels consistent with 1·5°C of heating by 173% in 2040, an increase from the 112% expected as of February, 2022

Emissions from oil and gas need to be reduced to keep global mean temperature rise below 1·5°C and enable a healthy future.^86,298^ This indicator assesses the alignment of current oil and gas company production strategies with Paris Agreement goals, with data on actual commercial activities from the Rystad Energy database based on the actual commercial activities for the 20 largest oil and gas companies by their projected 2040 production rates.^299^ These companies include 11 state-owned national oil and gas companies (NOCs) and nine publicly listed international oil and gas companies (IOCs) responsible for 37% and 15·5% of total global production, respectively, in 2022 (52·5% overall). Projected emissions under current strategies are compared with the IEA’s Net Zero Emissions pathway compliant with 1·5°C of heating,^300^ assuming constant market shares at the 2015–20 average.

**Figure 13:**
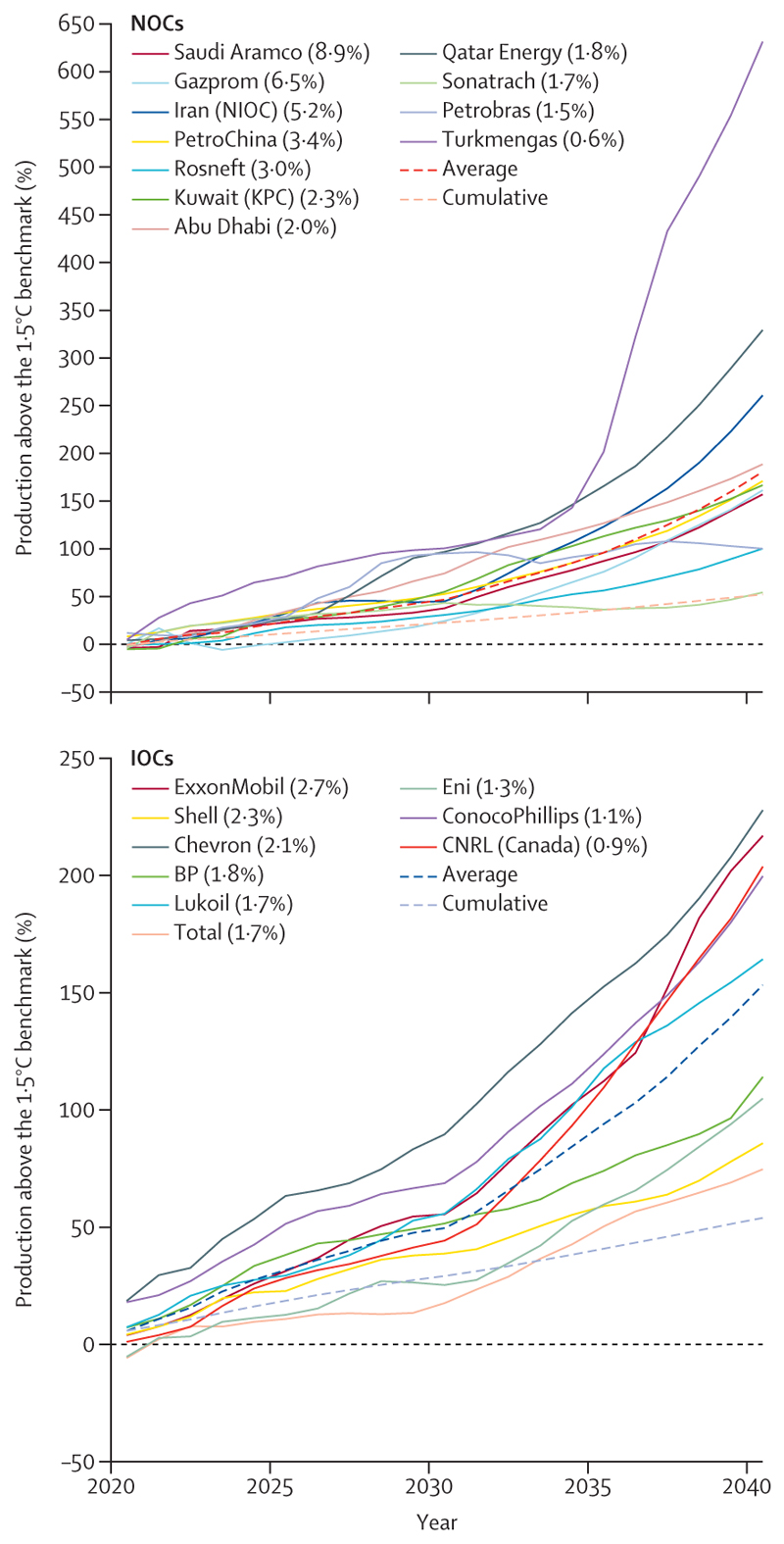
Compatibility of 20 large oil and gas company production strategies with the Paris 1·5°C climate target Percentages in brackets in the legend represent the average 2015–20 global market share. CNRL=Canadian Natural Resources Limited. IOCs=International oil and gas companies. KPC=Kuwait Petroleum Corporation. NIOC=National Iranian Oil Company. NOCs=national oil and gas companies.

Data indicate that, regardless of their claims and commitments, the compatibility of the plans of these oil and gas companies with international climate commitments has decreased further from 2021 to 2022. Their production strategies as of February, 2023, would generate GHG emissions in 2030 that exceed their annual share compatible with 1·5°C of heating by an average of 48% (47% for NOCs and 50% for IOCs), increasing to 173% in 2040 (181% for NOCs and 153% for IOCs; figure 13). These represent sizeable increases from the companies’ February, 2022, strategies, which would have resulted in combined emission excesses of 43% in 2030 and 112% in 2040. Although some of these increases are due to a slight tightening of the post-2030 Net Zero Emissions pathway, these still represent substantial increases in the extent to which oil and gas production ambitions in company strategies are inconsistent with the goals of the Paris Agreement.

#### Indicator 4.2.7: fossil fuel and green bank lending—headline finding: green sector lending has risen sharply since 2016, to $498 billion in 2021, and is approaching fossil fuel lending; however, 22 of the top 40 private banks have increased their fossil fuel lending

**Figure 14:**
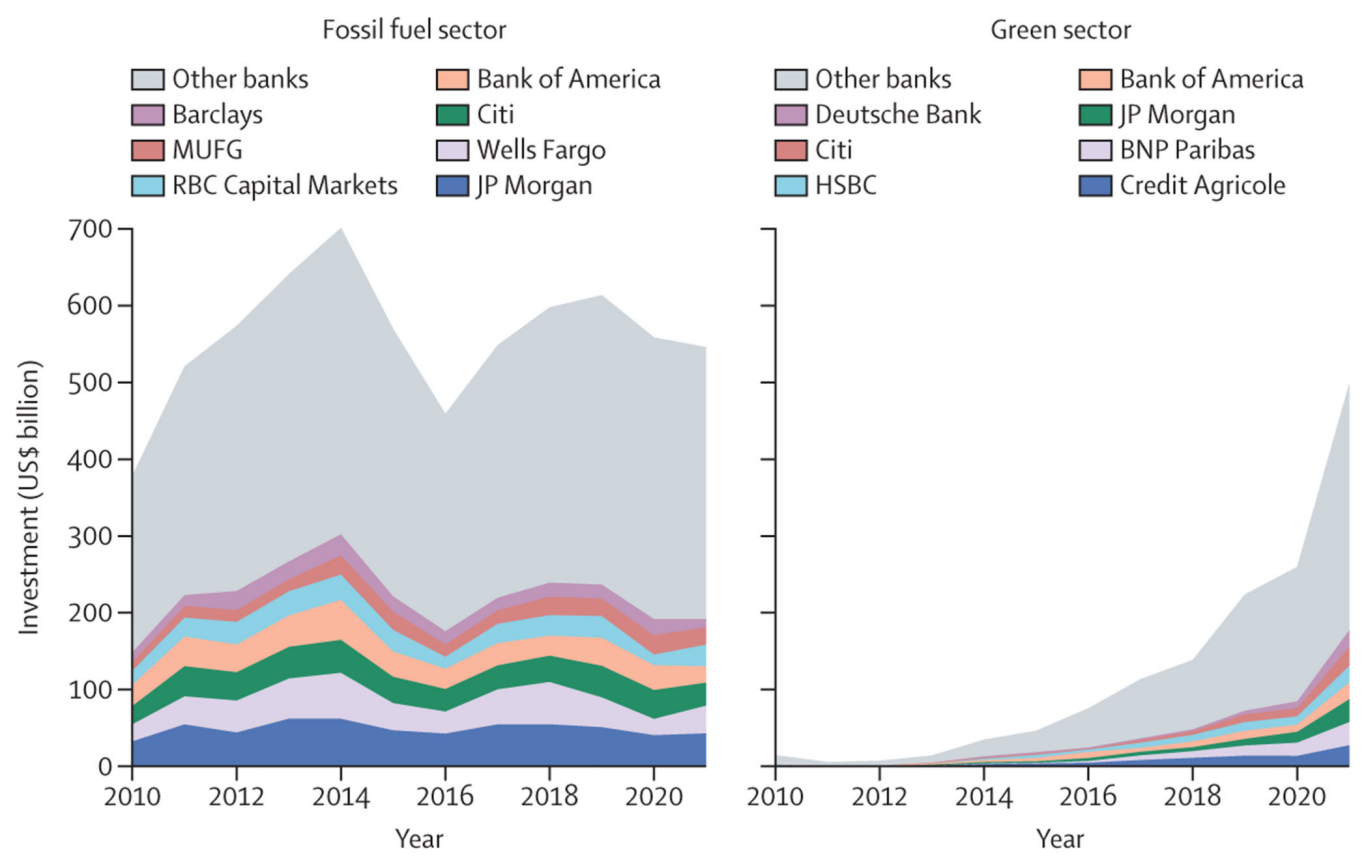
Total lending to the fossil fuel (left) and green sectors (right) between 2010 and 2021 The contributions of the seven top banks (ranked by cumulative investment) are also shown. MUFG=Mitsubishi UFJ Financial Group.

Redirecting the finance sector away from fossil fuels and towards clean renewables, energy efficiency, and carbon sinks is essential for a healthy, just transition to net-zero emissions. The Net-Zero Banking Alliance (NZBA), which currently represents 40% of global banking assets, was convened by the UN Environment Programme in 2021 to promote this goal. Estimates suggest that 70% of the required investment in green energy will come from private sources (ie, non-government sources), and an increasing amount will be mobilised as debt.^264^ New to this year’s report, this indicator draws on data from Bloomberg to monitor lending from private banks to the fossil fuel sector and green sector (comprising renewable energy and energy efficiency, carbon sinks, and other energy sustainability; appendix p 204). Average annual lending to the fossil fuel sector in the years before the Paris Agreement entered into force (2010–16), was $549 billion, and annual lending increased slightly in the following years (2017–21) to $572 billion (figure 14). The top seven lending banks, dominated by US institutions, account for 39% of total lending to the fossil fuel sector over the past decade.

In 2017–21, the 40 banks that lent the most to the fossil fuel sector in the past decade invested, on average, $489 billion yearly in oil and gas—87% of total global bank lending to the fossil fuel sector. Of these banks, 22 (55%) had increased their average annual fossil fuel lending from 2010–16. Despite being NZBA members, three Japanese banks (Sumitomo Mitsui, MUFG, and Mizuho Financial) dominated this cohort in terms of absolute spend and relative increase. By contrast, five European banks led fossil fuel finance reductions (Nordea, UBS, DNB ASA, Deutsche Bank, and Credit Suisse), having reduced their fossil fuel lending in 2017–21 by over 25% compared with 2010–16.

The biggest fossil fuel lenders (Citi, Wells Fargo, and JP Morgan), which together provide 22% ($616 billion) of total fossil fuel lending provided by the top 40 lending banks in 2017–21, have made negligible progress on reducing their fossil fuel lending, despite being NZBA members.

Green lending by the banking sector globally, on the other hand, has increased substantially from $75 billion in 2016 to $498 billion in 2021, when it approached the degree of lending to the fossil fuel sector. Of the top seven institutions that together provided 34% of total green finance in the past decade, four were European banks. Year-on-year growth increased from 16% in 2020 to 92% in 2021, reflecting the increased cost competitiveness of renewables following the COVID-19 pandemic.

### Conclusion

International loss and damage negotiations have put economics and finance at the centre of climate change discussions. This section exposes some of the extensive economic losses and damages from the health impacts of climate change currently affecting people worldwide (indicators 4.1.1–4.1.4), which in turn further undermine the socioeconomic determinants of health, and further restrict the scarce resources available to foster the transition to a healthy future.

Despite these impacts, substantial and sustained investment today can still deliver the economic transformation needed to avert the most catastrophic health impacts of climate change and forge a fairer, prosperous future. However, governments continue to incentivise a carbon-intensive, health-harming economy, allocating amounts often equivalent to substantial proportions of their health budgets to subsidising fossil fuels (indicator 4.2.4). Meanwhile, oil and gas companies are increasing their non-compliance with the 1·5°C heating target of the Paris Agreement as high energy prices incentivise oil and gas investments (indicator 4.2.6), and leading banks have maintained high levels of lending to fossil fuel companies despite their commitments to the NZBA (indicator 4.2.7).

Yet, some indicators show promising trends. Investments and employment in the renewable energy sector continue to increase (indicators 4.2.1 and 4.2.2). Green lending has accelerated since 2010 and is approaching the level of fossil fuel lending (indicator 4.2.7). Increased investment in clean renewable sources of energy can help countries respond to the energy crisis, improve energy security, and reduce air pollution, thereby helping to achieve a net-zero-emission, healthy, and equitable future.

A financial transformation will be essential to achieving these goals. This transition will require comprehensive cost–benefit analyses, which consider the economic costs of the health impacts of climate change, including the impacts on the health system and the broader economy, and the potential savings from avoiding them. The current capacity to capture such effective costs is limited by the scarcity of data on health spending and economic performance, and is a gap that the *Lancet* Countdown will continue to seek to address. A further gap relates to our limited capacity to monitor the potential local economic benefits and harms of the transition away from fossil fuels and towards renewable energies, particularly for communities at sites of extraction—a gap that will be crucial to address in the future to monitor and support a just transition.

## Section 5: public and political engagement with health and climate change

Previous sections made clear the fact that climate change is an increasing threat to health, driven by high-emitting countries but impacting most the communities least protected from its adverse effects.^301–303^ Action to date has failed to reverse the upward trends in energy-related carbon emissions,^304^ global temperatures,^305^ and their associated health-damaging exposures and impacts. Putting people at the centre of the climate conversation can help expose the human impacts of inaction, and the benefits of an accelerated response to meet the ambitions of the Paris Agreement.

This section focuses on societal actors with a key role to play in accelerating action. It tracks engagement by the media, individuals, scientists, governments, international organisations, and the corporate sector. Where possible, indicators examine engagement with the health benefits of climate change action. For all indicators, methods, data sources, and further analyses are provided in the appendix (pp 208–303).

### Indicator 5.1: media engagement with health and climate change—headline finding: in 2022, global newspaper coverage of health and climate change continued its upward trend, with 24% of all climate change articles mentioning health

Traditional media outlets (newspaper, radio, or television) are major platforms for public engagement, and continue to play an important agenda-setting role within today’s multimedia landscape.^306–311^ This indicator tracks coverage of health and climate change in 66 newspapers (both print and online formats) from 36 countries, including the People’s Daily, the media outlet that best represents mainstream politics in China.^312–314^ There is poor sample coverage in low-income countries, particularly in sub-Saharan Africa, thus under-representing reporting in these highly vulnerable countries.

Media engagement continued its upward trend in the global set of newspapers. In 2022, 24% of climate change articles (14 134 of 58 395) referred to health, the highest proportion to date. The number of articles engaging with health and climate change also increased, by 12% between 2021 and 2022, and by over 200% from 2017. However, there is little mention of the health co-benefits of climate change action. In English-language newspapers, fewer than 1% of articles (20 of 12 273) in 2022 relating to health and climate change refer to health co-benefits. In the People’s Daily, coverage of climate change in 2022 reached its highest recorded level. The increase was related to China’s carbon neutrality goal, pledged at the end of 2020. However, only a small proportion of climate change articles (7 of 2132; <1%) referred to health.

### Indicator 5.2: individual engagement with health and climate change—headline finding: individual engagement with health and climate change remained low in 2022; of all click views that led to health-related articles, only 0·03% came from climate change-related articles, and only 0·36% of click views that led to climate change-related articles came from health-related articles

This indicator tracks individual engagement with health and climate change by monitoring searches on Wikipedia, the online information source with a wider population reach than traditional encyclopaedias.^320–324^ With its content created and edited by users, Wikipedia also influences the agenda of other media sources.^311^ The analysis was based on the English-language Wikipedia, which represents roughly 50% of global traffic to all Wikipedia language editions.^320,321^

The indicator measures an individual’s clicks between an article on health and one on climate change (or vice versa), known as their clickstream activity. As in previous years, individuals seldom move between health and climate change; instead, co-click activity is predominantly within sets of articles on either health or climate change. Among all click views that led to a health-related article, only 0·03% came from a climate change-related article; among all click views that led to a climate change-related article, only 0·36% came from a health-related article. Across the 2018–22 period, there was no consistent trend in engagement between health and climate change as reflected in clickstream activity; the increase noted in 2021, which was driven by COVID-19 related searches, did not continue in 2022.

## 5.3 Scientific engagement with health and climate change

Peer-reviewed journals are the primary source of scientific evidence for the media, national governments, and the public.^322^ The following indicators track engagement on health and climate change in the scientific literature.

### Indicator 5.3.1: scientific articles on health and climate change—headline finding: after rapid growth in 2020 and 2021, the number of scientific papers investigating the links between health and climate change in 2022 fell by 2% compared with 2021, but remained three-times higher than in 2012

This indicator uses a machine-learning methodology to monitor the number of peer-reviewed scientific articles on health and climate and reports a growing body of scientific literature on climate and health, 80% of which has been published since 2012. In 2022, a total of 3149 papers were published, 3·7-times more than in 2012. This total represents a reduction of 2% between 2021 and 2022; although whether this reduction heralds a slowdown or a return to previous trends after exceptionally high years in 2020 and 2021 remains to be seen. Climate and health research continues to be dominated by studies on weather-related impacts, with little research on the links between health and mitigation and adaptation policies.

### Indicator 5.3.2: scientific engagement on the health impacts of climate change—headline finding: there are global inequalities in the location of studies referring to the health impacts of human-influenced climate drivers: 6·89 studies per 1 million people in very high HDI countries, and 1·61 and 1·51 studies per 1 million people for medium HDI countries and low HDI countries, respectively; of 37 extreme events analysed for detection and attribution between 2022 and 2023, 31 (84%) more likely, more severe, or more likely and more severe due to climate change

New to this year’s report is a set of subindicators capturing health impacts that can be tentatively attributed to climate change by using different methods to estimate these climate-related health impacts.

Building on indicator 5.3.1, the first subindicator maps the volume of studies published between January, 1985, and December, 2022, referring to health impacts related to climate variables, for which changes in the climate driver can be attributed to human influence. Across this period, 16 700 studies refer to health impacts from events that can be attributed to anthropogenic climate change, the largest proportion of which (41%; n=6767) are related to infectious diseases.

**Figure 15:**
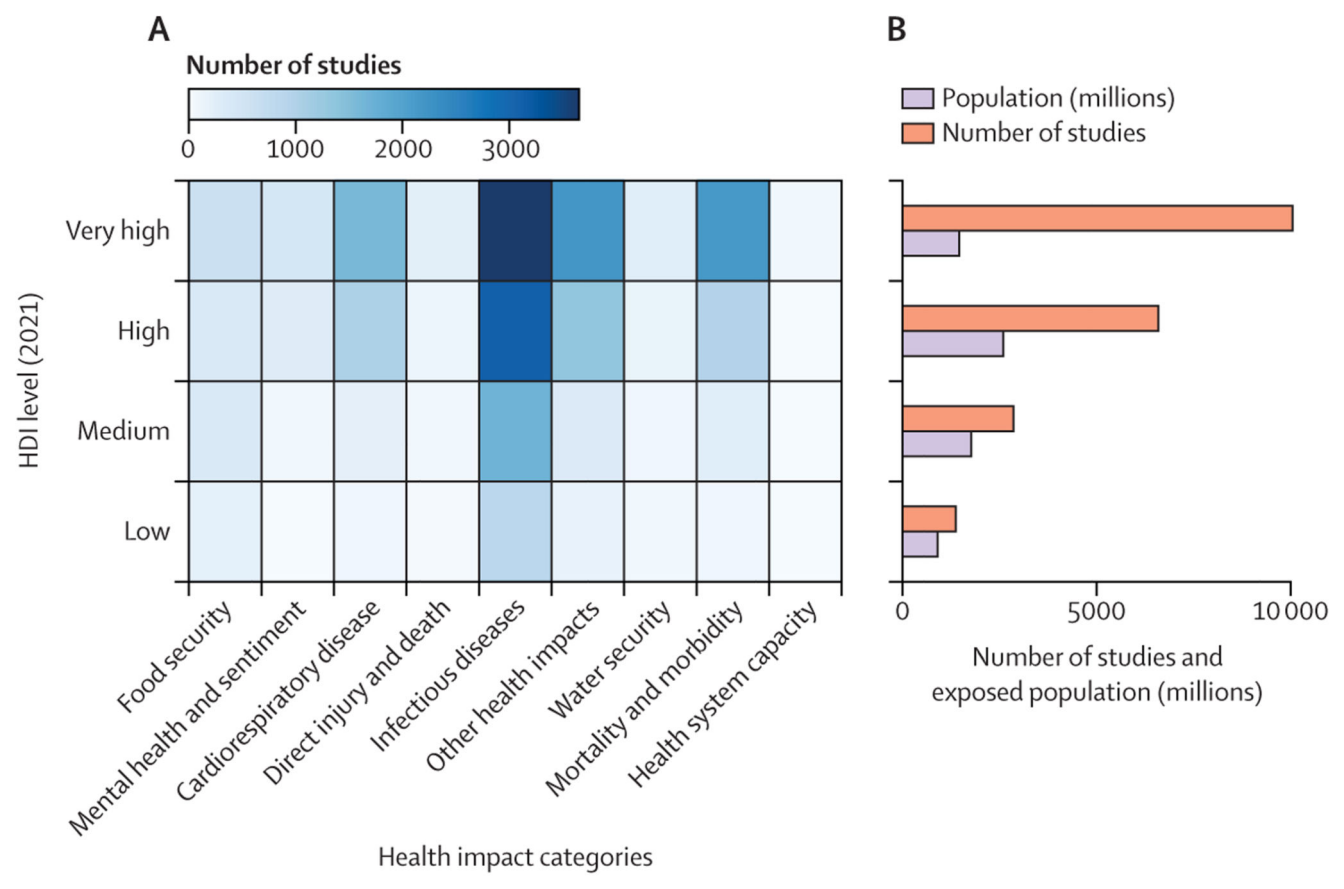
Studies linking health impacts to attributable climate changes (A) Studies linking health impacts to attributable climate changes by topic area, grouped by HDI level. (B) Number of studies linking health impacts to attributable climate changes and number of people (millions) exposed to attributable climate changes in countries grouped by HDI level. HDI=Human Development Index.

However, there is a marked geographical inequality in the location of these studies (figure 15). In very high HDI countries, there is a ratio of 6·89 studies per 1 million people exposed to human-influenced climate drivers; in high HDI countries, the ratio stands at 2·53 studies per 1 million people. In contrast, for medium HDI countries and low HDI countries, the ratios stand at 1·61 and 1·51 studies per 1 million people, respectively.

Detection and attribution studies evaluate the causal role of climate change in weather-related events.^323^ In this indicator, a literature review is used to monitor published detection and attribution studies, combining them with the associated morbidity and mortality values reported by EM-DAT.

From January, 2022, to March, 2023, 40 attribution studies analysed 37 events across six regions. Of these events, 31 (84%) were found to have been made more likely, severe, or both due to climate change. Extreme heat and flooding were the deadliest of the events analysed, causing 7991 and 3460 fatalities, respectively. However, these figures are probably underestimates, as they do not include delayed or indirect deaths that can be substantial in prolonged events.^324^

Seven studies (18%) pertained to events that occurred in low and medium HDI countries, an increase from four studies (6%) in 2021, and three studies (12%) in 2020.^38^ Additionally, 17 studies (43%) made direct reference to health impacts, consistent with an overall increase in impact attribution research linking attribution science directly to environmental and socioeconomic outcomes, including health.

## 5.4 Political engagement with health and climate change

Engagement from political leaders is central to climate interventions that protect human health. The following indicators monitor political engagement through national leaders’ statements at the UN General Debate, the cornerstone of the annual UN General Assembly;^325,326^ and NDCs, the key policy instrument for protecting people and the planet from climate change.^327^ In addition, a new indicator tracks engagement from international organisations with the intersection of health and climate change.

### Indicator 5.4.1: government engagement—headline finding: 50% of countries mentioned the intersection of health and climate change at the UN General Debate in 2022, a 10% decrease from 2021; 95% of updated NDC documents refer to health, an increase from 73% in the first submission

The UN General Assembly is the policy-making body of the UN,^325,328^ and (through the annual UN General Debate) provides the major global platform for national governments to highlight challenges requiring action by the international community. In 2022, engagement on heath and climate change declined compared with 2021, when 60% of government leaders discussed health and climate change, many in the context of the COVID-19 pandemic. Nonetheless, 50% of national leaders discussed the health–climate change nexus; SIDS represented 64% of those, continuing to lead engagement.

The speeches made by countries least responsible but most affected by climate change continued to highlight the human and environmental devastation of climate change. For example, Pakistan’s address focused on the floods the country experienced in 2022, stating, “In that ground-zero of climate change, 33 million people, including women and children, are now at high risk from health hazards, with 650 000 women giving birth underneath makeshift tarpaulins… in peril from disease and malnutrition.”^329^ Although most of the references focused on the impacts of climate change on health, there were also references to the health co-benefits of mitigation. For example, Fiji’s address stated, “We legislated a net-zero commitment by 2050, …which will make us more energy secure, protect us from energy price shocks beyond our control, and provide us with cleaner air, better health, and better jobs.”^330^

The second part of this indicator measures engagement with health and climate change in NDCs. In compliance with the Paris Agreement, countries must periodically report increasingly ambitious contributions towards international climate commitments.^45^ UN member states’ second NDCs (as of February, 2023) point to increasing engagement on health compared with their first NDCs. Engagement in the first NDCs was led by the countries most affected by climate change and all countries in the low HDI group referred to health. In subsequent NDCs, engagement increased sharply among the high HDI country group (from 86% to 100% of countries) and very high HDI country group (from 33% to 87%). Of all updated NDCs, 95% now refer to health—an increase from 73% in the first round. However, across all three rounds of NDCs, the very high HDI group was least likely to refer to health. As a further indicator of government engagement, panel 8 presents evidence on how national laws can serve as instruments of climate change action by mandating mitigation and adaptation actions with health co-benefits.

### Indicator 5.4.2: engagement by international organisations—headline finding: tweets mentioning the health co-benefits of climate change action reached a record of 22% of all monthly tweets from international organisations in November 2022, in a continuously upward trend

International organisations (eg, international and regional development agencies and supranational bodies such as the EU, African Union, and UN agencies) are playing an increasingly important role in climate change action.^349–351^ This new indicator tracks engagement on the health co-benefits of climate mitigation on the official X (formerly known as Twitter) accounts of international organisations, key platforms for their communication with journalists and the general public.^352^

**Figure 16:**
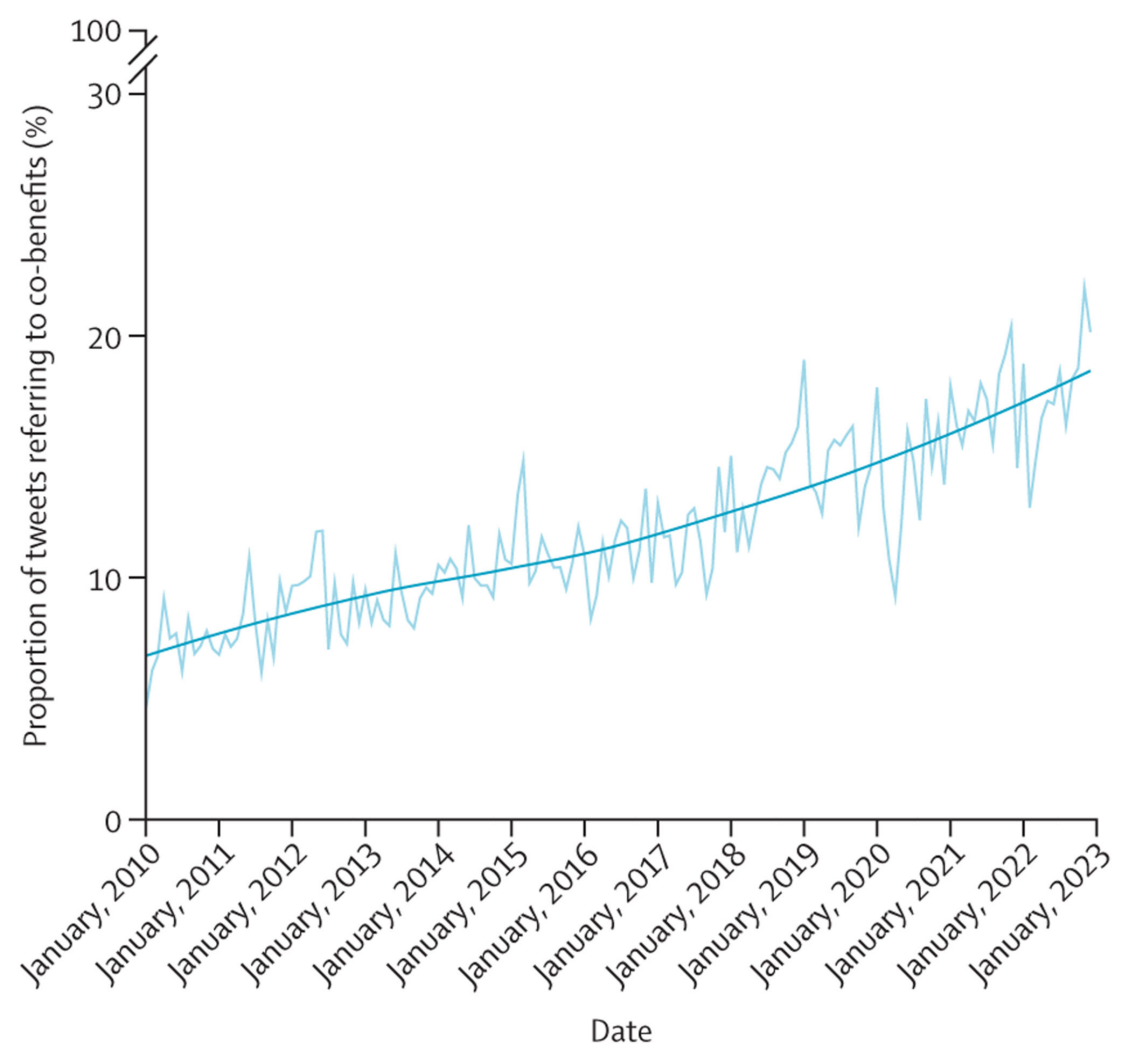
Engagement with the health co-benefits of climate change mitigation in the tweets of 41 international organisations, 2010–22 The smooth line represents a local regression with locally estimated scatterplot smoothing; the thinner line represents the actual number of tweets over time.

The indicator focuses on 41 international organisations with an operational focus on climate mitigation or adaptation across a broad range of sectors (eg, development and disaster risk management, trade and finance, energy policy, or food and agriculture). The dataset consisted of 1 392 892 tweets between 2010 and 2022, of which 1 354 924 were English-language tweets, which were analysed to identify tweets engaging with the health co-benefits of climate change mitigation. Engagement on these co-benefits (as a proportion of the total number of tweets by each international organisation) increased across this period: by November, 2022, a record 22% of tweets mentioned health co-benefits, an increase from an average of 7% in 2010 (figure 16). There were clear differences among sectors, with the greatest engagement on health co-benefits coming from international organisations from the energy, environment, food and agriculture, and global development banking sectors.

### Indicator 5.5: corporate sector engagement with health and climate change—headline finding: corporate sector engagement with health and climate change reached its highest recorded level in 2022, with 38% of companies referring to the health dimensions of climate change

The UN Global Compact (UNGC) is the largest global corporate sustainability framework,^353^ and one associated with improved environmental and social responsibility among participating companies.^354,355^ This indicator monitors engagement on health and climate change from the over 20 000 companies from 162 countries that signed up to the UNGC by tracking mentions of health and climate change in their annual Global Compact Communication of Progress reports. Engagement in 2022 reached its highest recorded level, with 2337 of 6089 companies (38%) referring to the health dimensions of climate change. However, a higher proportion of companies continue to engage with either health (88% in 2022 *vs* 82% in 2011) or climate change (75% in 2022 *vs* 63% in 2011).

## Conclusion

Public and political engagement on health and climate change continued its upward trend across 2022, reaching the highest recorded level of engagement among government leaders and companies signed up to the UN sustainability charter, while maintaining recent higher engagement in global newspapers. There is also evidence of increasing engagement on the health co-benefits of climate mitigation among international organisations, for example. The *Lancet* Countdown will continue to track key sites of engagement. It will address gaps in the current coverage by extending the global reach of existing indicators and by introducing new indicators that directly capture people’s perceptions of health and climate change within and between countries.

Engagement with the climate change–health nexus is growing. But, as this section demonstrates, there is greater engagement with health and climate change as separate issues, as evidenced by the digital footprint of Wikipedia users, government leaders at the UN General Debate, and companies in the UNGC. In addition, profound inequities in engagement persist. Scientific research is concentrated in countries and regions more protected from the adverse consequences of climate change, with much less focus on communities vulnerable to climate change impacts in Africa, Asia, and South and Central America. At the same time, government engagement has been led by countries bearing the brunt of a climate crisis to which they contributed little. These stark contrasts point to the negative impacts of climate change on inequality and the importance of tracking the distributional effects of climate change action both across and within countries.^303,356,357^

## Conclusion: the 2023 report of the *Lancet* Countdown

The 2022 report of the *Lancet* Countdown warned that global health is at the mercy of fossil fuels and noted a unique opportunity, as countries responded to the energy crisis, to deliver transformative climate change action for a thriving future.^38^ This year’s report finds few, if any, signs of the urgently needed progress, in a world still bound to fossil fuel ambitions.

With extreme weather records breached in all continents through 2022, risks to human health and survival are increasing across all the dimensions monitored. Around the world, people face increased heat-related illness and extreme weather-related risks, infectious disease spread, and worsened food insecurity (section 1). The associated economic losses add to the health burden, eroding the socioeconomic building blocks of health (indicators 1.1.4, 4.1.1, and 4.1.3). Despite the rising risks, adaptation efforts fall short of the necessary action to protect people’s health, particularly in lower HDI countries where structural inequities limit access to funding and technical capacity (section 2). This scarcity is aggravated by the rising economic losses from climate change impacts, and the persistent failure of wealthier countries reach the promised sum of $100 billion annually to support countries most affected by climate change.^358^ As a result, the most vulnerable and minoritised communities are left the least protected, and the deep within-country and between-country health inequities are further exacerbated.

The impacts observed at the global 10-year average of 1·14°C of heating offer an early glimpse into a future that increasingly threatens people’s health. New to this year’s report, projections reveal the potential human cost of further delayed action, with every health hazard assessed projected to increase even under a scenario compatible with a 2°C mean temperature rise (section 1). Accelerating adaptation remains essential to minimising the associated health impacts. However, with various limits to adaptation already being rapidly reached,^2^ these data underline the crucial health imperative to urgently strengthen mitigation efforts to restrict global mean temperature rise to 1·5°C.

However, the pace and scale of mitigation efforts continue to fall very far short of those required to safeguard people’s safety. Current policies put the world on track for a potentially catastrophic 2·7°C of heating by 2100, and energy-related emissions reached a new record high in 2022 (indicator 3.1.1). Meanwhile, high energy prices yielded $4 trillion in profits for oil and gas companies (panel 7), incentivising fossil fuel expansion. Indeed, oil and gas companies allocated only roughly 4% of their capital investment to renewables and further reduced the compliance of their strategies with international climate change goals (panel 7 and indicator 4.2.6).^275^ The finance sector is also contributing to growing health threats, as 55% of the private banks that provide the most finance to fossil fuels are increasing their lending (indicator 4.2.7).^77,276–280^ Rather than discouraging health-harming fossil fuel burning, most governments keep incentivising it through subsidies, often for sums equivalent to substantial proportions of their health budgets (indicator 4.2.4). Meanwhile, agricultural emissions also continue to increase, alongside a global food system that supports unhealthy, carbon-intensive diets (indicators 3.3.1 and 3.3.2).

Despite a rapidly growing use of clean renewable energy, renewables still account for only 9·5% of the world’s electricity (indicator 3.1.1). The share is even less in low HDI countries, where (often despite vast availabilities of natural renewable energy resources) clean renewables account for just 2·3% of electricity generation, and 92% of domestic energy still comes from polluting fuels (indicators 3.1.1 and 3.1.2). This resulted in 1·8 million deaths from ambient fossil-fuel-derived air pollution globally in 2020, and the use of dirty fuels inside homes caused, on average, 140 deaths per 100 000 inhabitants across 62 assessed countries (indicators 3.1.2, 3.2.1, and 3.2.2). Populations in low HDI countries are exposed not only to dirty fuels, but also to persistent energy poverty (indicator 3.1.2 and 3.2.2). In addition, communities living in proximity to fossil fuel extraction sites and renewable industries often see their health affected by the harms of poorly regulated local industrial activity.^34,79^ This inequitable energy transition is leaving the most underserved populations behind, exacerbating heath inequities and perpetuating harmful extractive practices that undermine human health, wellbeing, and the social, economic, and environmental conditions on which they depend (part A). Notwithstanding the insufficient progress identified, this report reveals the path to a healthy future. Redirecting subsidies, lending, investment, and other financial flows away from fossil fuels is crucial to supporting a healthy future. Funds are available to support a just clean energy transition, health-promoting activities, and reduced inequities (indicator 4.2 and panel 7). Empowering countries and local communities in the safe development, deployment, and adoption of clean energies can reduce energy poverty by supporting access to decentralised energy. In turn, this can promote access to quality health-supporting services and promote local skills, generate jobs, and support local economies—strengthening the socioeconomic determinants of health (indicator 4.2.2 and panel 6).^34,79,81,359^ Health-centred urban redesign can promote safe active travel, reduce building and transport-based air pollution and GHG emissions, and increase resilience to climate hazards (indicators 3.1.3 and 3.2.1). Increasing urban green spaces can also offer local cooling, increase carbon sequestration, and provide direct benefits to physical and mental health (indicators 2.2.2 and 2.2.3). Providing further support for climate and health risk assessments and adaptation planning can support increased resilience to unavoidable climate change, delivering stronger health systems for all (indicators 2.1.1–2.1.3, 2.3.1, and 2.3.2). The health benefits of climate action could be transformative, protecting lives and livelihoods and paving the way to a thriving future.

Achieving these ambitions requires guidance and leadership on health-promoting climate policies, and steadfast, sustained commitments to deliver a just transition. Driven by the mandate to protect people’s health, wellbeing, and survival above all else, health professionals are uniquely positioned to guide actions to safeguard the human right to heath and a healthy environment.

Encouragingly, following decades of the health sector raising the alarm,^360^ engagement with health and climate change is increasing among key actors and decision makers (indicators 5.1, 5.4, and 5.5). The renewed focus on health within forthcoming UNFCCC negotiations offers an unprecedented opportunity to foster climate action (panel 2).^361^ Making the most of this opportunity will require coordinated efforts grounded in science to hold decision makers accountable and counteract the growing lobbying and influence of the fossil fuel sector and other health-harming industries. To truly protect health, climate negotiations must drive a rapid and sustained shift away from fossil fuels, accelerate mitigation, and increase support for health adaptation. Anything less would amount to healthwashing—increasing the acceptability of initiatives that minimally advance climate change action to the detriment of billions of people alive today.

With climate change claiming millions of lives annually and its threats rapidly growing, seizing the opportunity to secure a healthier future has never been more vital. Ensuring that a thriving future remains in reach will require the coordinated action of health professionals, policy makers, corporations, and financial institutions.

## Supplementary Material

Supplementary appendix

## Panel 1 11 priorities to deliver a healthy, thriving future

Data in this report underscore the health imperative for accelerated action to limit climate change and its health impacts. Numerous previous efforts have laid out roadmaps and policy recommendations to meet the goals of the Paris Agreement.^39,40,42,43^ Building on these, the recommendations below identify priorities to maximise the benefits of climate change action for people’s health and wellbeing. As such, they are intended to shape the priorities of international organisations, national or subnational decision makers, business, financial institutions, and health systems as they implement the policies needed to meet their commitments under the Paris Agreement. In all cases, regular monitoring and course correction are crucial to ensure that benefits to health and wellbeing are achieved and social and health inequities are reduced through their implementation.

### Accelerate the phasing out of fossil fuels, prioritising interventions with health co-benefits in the energy sector and food system

#### 2 Reduce the health harms of energy poverty by supporting a just zero-carbon transition

Empower countries with a high reliance on dirty fuels (ie, fossil fuels, particularly coal, and biomass) and high levels of energy poverty to locally develop, deploy, maintain, and use modern renewable energy sources; and to develop autonomous energy systems. In doing so, and through knowledge and technology transfer programmes and financial support, support the development of local skills and promote healthy local employment. Prioritise interventions that deliver energy to energy-poor regions, focusing on electrifying homes and health-care facilities, and enabling access to quality health-supporting services (indicators 3.1.1, 3.1.2, 4.2.1, 4.2.2, 4.2.7, and panel 5).

#### 3 Accelerate mitigation in food systems through support for, and promotion of, healthier, low-carbon diets

Support investment in climate-smart horticulture, through for example (and where locally relevant), research and development, subsidies, improved extension services, and better market access. Support consumers through policies that improve equitable access to affordable, culturally relevant, low-carbon, plant-forward diets that meet nutritional needs, through for example, their integration into social programmes (including maternal and child health initiatives and safety nets), targeted subsidies, support to reduce food waste, and improved public health messaging; in addition to regulating against the production, sale, and promotion of unhealthy foods (indicators 3.3.1 and 3.3.2).

### Promote leadership from the health sector by delivering health-promoting climate change action

#### 4 Deliver public health programmes that simultaneously improve public health and reduce greenhouse gas emissions

Consider the risks, needs, culture, and preferences of local communities. Include policies to tackle air pollution by rapidly phasing out fuel burning, especially near populations who are vulnerable to the associated health risks (eg, around hospitals, schools and care facilities, and inside people’s homes); supporting, facilitating, and enabling a transition to healthy, affordable, low-carbon diets; enhancing green prescribing; supporting locally tailored and sustainable heat-coping behaviours that protect health; and promoting, enabling, and facilitating increased physical activity, including for travel (indicators 1.1.2, 1.1.5, 2.2.2, 2.2.3, 3.2, and panel 4).

#### 5 Lead by example by building sustainable, efficient, and zero-emission health systems in alignment with the ambitions of the 26th Conference of the Parties (COP26) Heath Programme

Prioritise clean energy, energy efficiency, and resource stewardship. Foster the safe replacement of high-carbon medical resources with low-carbon alternatives (including anaesthetic gases and inhalers). Promote transparency on the impacts of climate change, and the decarbonisation of suppliers (indicator 3.4).

### Accelerate climate change adaptation for health

#### 6 Accelerate the development of climate-resilient health systems, in agreement with the COP26 Health Programme

Scale up technical and financial support at the national and local level, particularly in low HDI countries and medium HDI countries, to conduct thorough national and subnational health and climate change risk and vulnerability assessments and Health National Adaptation Plans (indicators 2.1.1–2.1.3 and 2.2.4).

#### 7 Increase the capacity of health systems to prepare for, and respond to, climate-related health risks

Include the implementation of climate-informed health surveillance, and early warning and response systems for key health risks threatening local populations. Ensure adequate technical and financial support and increased alignment between health and meteorological services, including by establishing formal collaboration between these sectors^44^ (indicators 2.2.1 and 2.2.5).

### Transform financial systems to support a healthy, sustainable future

#### 9 Rapidly phase out all subsidies for, lending to, and investment in oil and gas companies and projects, including their exploration and extraction activities

Redirect financial support towards developing, scaling up, and deploying healthy, zero-carbon energy and energy efficiency, and towards activities that simultaneously improve the health, wellbeing, and livelihoods of all populations, particularly the communities most vulnerable to the withdrawal of subsidies. Increase investment in zero-carbon energy and energy efficiency and accelerate divestment from fossil fuel funding (indicators 4.2.1, 4.2.3, 4.2.5, and 4.2.7).

### Increase resources and support to continue expanding the knowledge base, understanding, and engagement of key actors in health and climate change

#### 10 Strengthen global capacity for health and climate change research and knowledge generation

Focus on identifying and informing effective and cost-efficient public health and climate change adaptation and mitigation interventions; on monitoring these interventions to reduce health burdens and inequities; and on characterising the health impacts of the commercial activities of the fossil fuel and other carbon-intensive industries, and of interventions to prevent them. In doing so, promote and facilitate the active involvement of young people and minoritised groups in identifying health and climate change solutions that minimise or eliminate health inequities and foster learning from people in the front line of climate change impacts. Focus particularly on harnessing and capturing the knowledge of Indigenous communities through meaningful engagement (indicator 5.3).

#### 11 Increase support to maintain and strengthen monitoring of health and climate change at global, regional, and national levels

Monitor the health benefits and any unintended harms of climate action within the UNFCCC’s Global Stocktake and Global Goal on Adaptation. Establish national observatories on health and climate change with standardised frameworks and indicators to evaluate progress and inform decision making (panel 2).

## Panel 2 UN climate negotiations for a healthy future

The 2015 Paris Agreement was ratified by 195 countries. Legally binding and science-driven, it commits governments to pursue efforts to limit global mean temperature rise to 1·5°C; protect the human right to health; prevent harm; and promote the right to a clean, healthy, and sustainable environment.^45,46^

The Paris Agreement’s potential to deliver immediate health benefits and avoid the threat to human survival posed by climate change prompted it to be labelled as potentially the most important public health agreement of the 21st century.^47^ Delivering such ambition requires placing health protection and promotion at the heart of climate negotiations, with many of the negotiation areas offering opportunities to achieve this, as laid out below.

### Mitigation

Mitigation ambition and implementation must be urgently scaled up in this crucial decade to improve and protect global health and equity.^48^ Acknowledging common but differentiated global responsibilities, Nationally Determined Contributions and Long-term Low Emissions and Development Strategies offer the possibility for countries to put forward their climate plans and could offer a platform to ensure these plans maximise health benefits while minimising trade-offs. Ongoing UN Framework Convention on Climate Change (UNFCCC) negotiations on response measures also provide a mechanism to encourage governments to quantify and maximise health co-benefits from climate change actions.^49^

### Adaptation

Placing public health at the centre of transformational adaptation targets can help ensure that people’s health, and particularly the health of the people most vulnerable to climate change impacts, is protected.^50^ The 28th Conference of the Parties (COP28) will finalise the work programme on the Global Goal on Adaptation (GGA).^51^ Positioning health and wellbeing as a core pillar of adaptation within the GGA could foster actions to protect the health of world populations from rising climate change-related health risks.

### Loss and damage

The health impacts of climate change represent the human face of non-economic loss and damage. COP27 established a new fund, which is expected to be operationalised by the start of COP28, to help what the UNFCCC refers to as developing countries respond to losses and damages caused by climate change. These include loss of life and damage to health and health systems.^46^

### Economics and finance

Negotiations to transform the global financial system are taking place in 2023.^46^ COP28 offers an opportunity to make finance flows “consistent with a pathway towards low [greenhouse gas] emissions and climate-resilient development”.^45^ This includes delivering on the 2009 Copenhagen Accord commitment to mobilise US$100 billion per year to support countries classified as developing within the UNFCCC in their climate transition and to double adaptation finance.^52^ This commitment offers an opportunity to eliminate harmful finance flows, including fossil fuel subsidies, and to support a healthy future by redirecting funds to interventions that protect and promote equity, health, and survival.

### Food and agriculture

The role of the global food system in responding to climate change is organised under the Sharm el-Sheikh joint work programme (2023–26).^46^ This programme mandates that governments strengthen the role of their food systems in nurturing human health and wellbeing, including through safeguarding food security; ending hunger; improving nutrition security; and building inclusive, sustainable, and climate-resilient agricultural systems. Realising these ambitions could provide major health benefits to global populations, particularly populations suffering from food insecurity and malnutrition.

### Stocktaking and monitoring

The first Global Stocktake, an exercise of the UNFCCC concluding at COP28, will assess global progress in the delivery of the goals of the Paris Agreement. Considering public health, wellbeing, and survival as primary goals against which to monitor progress can ensure countries’ actions are tailored and refined to maximise the health benefits of climate action as they work to deliver the ambitions of the Paris Agreement.

## Panel 3 The indicators of the 2023 report of the *Lancet* Countdown

### 1 Health hazards, exposures, and impacts

1.1 Heat and health
  1.1.1 Exposure to heating
  1.1.2 Exposure of vulnerable populations to heatwaves
  1.1.3 Heat and physical activity
  1.1.4 Change in labour capacity
  1.1.5 Heat-related mortality
1.2 Health and extreme weather-related events
  1.2.1 Wildfires
  1.2.2 Drought
  1.2.3 Extreme weather and sentiment
1.3 Climate suitability for infectious disease transmission
1.4 Food security and undernutrition

#### 2 Adaptation, planning, and resilience for health

2.1 Assessment and planning of health adaptation
  2.1.1 National assessments of climate change impacts, vulnerability and adaptation for health
  2.1.2 National adaptation plans for health
  2.1.3 City-level climate change risk assessments
2.2 Enabling conditions, adaptation delivery, and implementation
  2.2.1 Climate information for health
  2.2.2 Benefits and harms of air-conditioning
  2.2.3 Urban greenspace
  2.2.4 Global multilateral funding for health adaptation programs
  2.2.5 Detection, preparedness, and response to health emergencies
2.3 Vulnerabilities, health risk, and resilience to climate change
  2.3.1 Vulnerability to mosquito-borne disease
  2.3.2 Lethality of extreme weather events
  2.3.3 Rising sea levels, migration, and displacement

#### 3 Mitigation actions and health co-benefits

3.1 Energy use, energy generation, and health
  3.1.1 Energy systems and health
  3.1.2 Household energy use
  3.1.3 Sustainable and healthy road transport
3.2 Air pollution and health co-benefits
  3.2.1 Mortality from ambient air pollution by sector
  3.2.2 Household air pollution
3.3 Food, agriculture, and health co-benefits
  3.3.1 Emissions from agricultural production and consumption
  3.3.2 Diet and health co-benefits
  3.4 Health-care sector emissions

#### 4 Economics and finance

4.1 Economic impact of climate change and its mitigation
  4.1.1 Economic losses due to weather-related extreme events
  4.1.2 Costs of heat-related mortality
  4.1.3 Loss of earnings from heat-related reduction in labour capacity
  4.1.4 Costs of the health impacts of air pollution
4.2 Economics of the transition to zero-carbon economies
  4.2.1 Clean energy investment
  4.2.2 Employment in renewable energy and fossil fuel industries
  4.2.3 Funds divested from fossil fuels
  4.2.4 Net value of fossil fuel subsidies and carbon prices
  4.2.5 Production-based and consumption-based attribution of CO_2_ and PM_2·5_ emissions
  4.2.6 Compatibility of fossil fuel company strategies with the Paris Agreement
  4.2.7 Fossil fuel and green bank lending

#### 5 Public and political engagement with health and climate change

5.1 Media engagement with health and climate change
5.2 Individual engagement with health and climate change
5.3 Scientific engagement with health and climate change
  5.3.1 Scientific articles on health and climate change
  5.3.2 Scientific engagement on the health impacts of climate change
5.4 Political engagement with health and climate change
  5.4.1 Government engagement
  5.4.2 Engagement by international organisations
5.5 Corporate sector engagement with health and climate change

## Panel 4 The *Lancet* Countdown’s evolving monitoring system

The *Lancet* Countdown was established in 2016 to offer an independent, rigorous, and comprehensive assessment of progress on health-promoting climate change action. The indicator domains covered in its monitoring system are the product of 8 years of iterative improvements. In the initial phase of the *Lancet* Countdown, indicator domains were selected through consultation with a variety of experts and policy makers, and subsequently refined by academic working groups at a series of multidisciplinary workshops throughout 2016.^54^ With the publication of the inaugural report in 2016, the *Lancet* Countdown initiated an open consultation to further refine the indicator domains and metrics.^54^ After 2016, the suite of indicators was iteratively improved yearly through internal consultation within the academic working groups of the *Lancet* Countdown, and complemented by an ongoing and inclusive process of engagement with the broader scientific community. The *Lancet* Countdown maintains an open approach and continues to invite direct input on the content, methods, and data of its indicators, and proposals for new indicators and indicator domains, through its website.^53^

To ensure the relevance and quality of the Countdown metrics, all proposals for new indicators or indicator improvements (both those made by members of the *Lancet* Countdown and those proposed by other colleagues) are evaluated through an independent assessment process, in which external experts evaluate the quality and fit of the indicators, providing rigour and transparency to the collaboration’s data.

Each indicator in the *Lancet* Countdown’s assessment must comply with the following criteria:

- Track an aspect of the relationship between health and climate change that is well evidenced in the literature and not adequately covered through other indicators in the report
- Use data from a reliable source that are available at adequate temporal and spatial scales to enable trends to be observed at a global level
- Be updatable periodically—ideally annually or more regularly

The indicators must also be:

- Meaningful: an indicator must track an aspect of the relationship between health and climate change that is well evidenced in the literature and relevant at a global level
- Relevant: the area being tracked by an indicator must be of relevance to policy and decision makers or represent an important contribution to the field of science of climate change and health
- Scientifically sound and reproducible: an indicator must use well established, internationally accepted, and ideally previously published scientific methods
- Temporally representative: an indicator should provide annual data for the recent past and up to as recent a year as possible; it must be available across an adequate timescale to allow for attribution to climate change, where relevant
- Geographically representative: an indicator should ideally be available at a country-level resolution (or higher); its geographical coverage should be enough for global trends to be observed, covering at least 40 countries evenly distributed across the four World Bank income contexts, the four Human Development Index Groups, and the five WHO regions initially, with the possibility of expansion to 150 countries at least; in the case of indicators tracking aspects relevant to restricted locations, over 80% of the relevant countries must be covered
- Reliable and updatable: an indicator should use data from a reliable source that is fit for its purpose; publicly available databases, and especially those developed by international organisations, governmental bodies, or academic institutions, are preferred. Data sources must be regularly updated.

Abiding by these criteria enables the *Lancet* Countdown to maintain a globally representative, annually updateable, and relevant monitoring system. However, these criteria also pose restrictions, limiting the possibility of capturing aspects for which comprehensive data coverage is not available globally, not regularly updated, or not quantifiable. As a result, some important gaps remain in the monitoring system, including the mental health impacts of climate change; the links between climate change, migration, and health; the health benefits of shifts to active travel; the economic losses associated with the health impacts of climate change; and other areas more broadly neglected within data collection and research efforts.

Importantly, the scarcity of data disaggregated by gender, race, indigeneity, socioeconomic position, religion, nationality, or other minoritised characteristics restricts the capacity of these indicators to capture the inequities that underpin climate change impacts and climate change action. As the *Lancet* Countdown enters a new phase, we will revisit the indicator domains and focus efforts on guiding the collection of data that can support an increasingly relevant, comprehensive, and actionable monitoring system.

## Panel 5 Early-warning systems for heatwaves: protecting people’s health in a heating climate

During the summer of 2022, England saw deadly, record-breaking heatwaves, with temperatures of 40·3°C prompting the first ever Level 4 Heat-Health Alert (HHA) and Red Extreme Heat (EH) weather warnings. To understand and inform measures to reduce health impacts from heatwaves, the UK Health Security Agency (UKHSA) routinely assesses and reports all-cause excess mortality during episodes of heat.^188^ In 2022, observed excess mortality was calculated accounting for COVID-19 deaths. In addition, a novel analysis was performed to model the mortality which would have been expected on the basis of the relationship between temperature and mortality and, with multiple lines of evidence, assess seasonal impacts. Although the confidence intervals of each estimate overlap, the observed excess mortality associated with five episodes of extreme heat in 2022 (2985 deaths) was 23% below the mortality modelled (3889 deaths) on the basis of observed temperatures across England. This difference increased to 32% during the heat episode for which the Level 4 HHA and Red EH weather warnings were issued (1256 observed deaths *vs* 1852 modelled deaths).

The relationship between heat and mortality, and particularly when assessing the impact of implemented interventions on reduced mortality, is very complex, particularly considering estimate uncertainty and other challenges regarding attribution. However, UK Meteorological Office customer research suggests that 98% of the public took some degree of action following the Level 4 HHA and Red EH warnings, and warnings, clear heat messaging, public buy-in, and a joint meteorological and public health communication effort are likely to have contributed to a lower observed mortality than that predicted through modelling. Heatwaves in the summer of 2022 in England could have had a more disastrous effect, but hundreds of lives were potentially saved through interventions. Further work is needed to reduce heat-related mortality, as highlighted by the 2985 excess heat-related deaths that occurred. During the heat episode that occurred late in the season, the observed excess mortality was higher than expected. Factors that might have contributed to this include the fact that the alert level was not as high as in previous episodes (Level 3 HHA), which might have led to a lower perceived risk by the public and therefore a decrease in health-protective action.^188^ UKHSA’s next phase will move towards impact-based alerting, using maximum and minimum temperature thresholds to guide a dynamic risk assessment process. This added detail and flexibility in the alerts themselves is anticipated to provide additional information for more informed decisions. Through additional assessments of humidity, cloud cover, wind, and access to cooling mechanisms there can be a more complex understanding of heatwave health impacts globally.

As reported in indicator 2.2.1, many other countries are beginning to integrate meteorological efforts into health-based warning systems, with city-level actions tending to dominate. Within central and South and central America, only Argentina, Chile, and Uruguay discuss implementing early warning systems for heat in their National Adaptation Plans. Of these three countries, only Argentina has expanded such systems to non-urban contexts. Increasing the effectiveness of heatwave early warning systems requires interdisciplinary approaches, the use of meteorological services to provide advanced weather forecasts and public health communication, and mechanisms for monitoring and evaluation. In addition, tailoring definitions of heatwaves and their thresholds to each region can ensure that early warning systems are most effective in protecting people’s health during heat extremes.

## Panel 6 Powering health-care delivery through renewable energy

Reliable electricity in health-care facilities is essential for quality health-care provision. Electricity is needed for basic amenities, such as lighting and clean water; it powers crucial devices in health-care settings, including vaccine and medication refrigeration, oxygen concentrators, and some diagnostic and surgical equipment; and it enables the use of key tools for maternal and child health, including ultrasounds, fetal heart monitors, and baby warmers.^225^ Health centres without electricity perform fewer deliveries and have fewer patients.^226^ Yet, close to 1 billion people are still served by health-care facilities without reliable electricity. At least 25 000 health-care facilities in sub-Saharan Africa have no electricity access, and at least 68 000 health-care facilities have unreliable electricity.^227^

Clean, renewable energy presents an opportunity to provide electricity to remote settings or energy-poor settings, with the potential of saving lives and improving the health of vulnerable populations. Although extending national grids can be expensive and slow, options such as solar photovoltaics are cost effective and offer non-polluting energy without the need for grid connection or fuel input.

Because electrification has been shown to improve vaccination rates, emergency capabilities, and the quality of maternity care, it could be a key tool in limiting gender and health disparities.^228^ With widely unequal access to electricity across rural and urban settings, electrification can also help reduce within-country health inequities. Moreover, access to decentralised renewable energy drastically increases the climate resilience of health-care facilities, making them independent from volatile energy markets.^225^

Some laudable case studies exemplify the power of renewable energy in improving access to life-saving energy. An onsite solar array provides energy for Kalungi Hospital (over 100 km away from Kampala, Uganda),^229^ allowing it to stay open later, to refrigerate vaccines and supplies, to increase the surgery patient load, and to power diagnostic tools and the sterilisation of medical instruments, all while lowering annual fuel costs and increasing clean water access and security by pumping water at a well site with the electricity.^229^

The SELCO Foundation has worked with hundreds of health-care facilities in India to establish decentralised energy systems. The installation and use of solar systems in health-care facilities in the state of Manipur has resulted in 80–95% of facilities reporting fewer wasted vaccine dosages, the extension of their clinic hours, and an overall ease of operations.^225^ After a rural electrification programme in Gujarat, there were increases in the presence of functional operating tables and delivery tables and higher immunisation rates.^230^

In a joint report this year, WHO, the World Bank, the International Renewable Energy Agency, and Sustainable Energy for All provided a comprehensive update on the status of health-care facility electrification, identifying technical and economic solutions, estimating the amount of investment needed, and analysing the policy frameworks enabling accelerated electrification of health-care facilities.^225^ This update stresses that there is urgent need for US$4·9 billion in funding to bring health-care facilities in the select countries that were assessed up to a minimal or intermediate standard of electrification, ensuring the long-term functionality of decentralised energy systems, supporting local skills and renewable energy markets, providing access to medical devices and training, and increasing cooperation between energy and health sectors and various institutions. Efforts must be focused on leveraging synergies, maximising impact, and unlocking resources to improve access to health care in these countries.

## 7 Strengthening climate finance in a time of geopolitical turmoil

Last year, the *Lancet* Countdown reported that the world faced multiple compounding crises. As countries devised responses to the converging energy, cost-of-living, and climate crises, the 2022 report identified the opportunity for an aligned response to enable a thriving, healthy future. Finance plays a key role in enabling such action and is a primary focus in climate negotiations (panel 2). The 2022 Emissions Gap Report outlined six approaches for financial sector reform, comprising: making financial markets more efficient; introducing carbon pricing; nudging financial behaviour through government interventions; creating markets for low-carbon technology; mobilising central banks; and creating local climate clubs of cooperating countries.^265^

Against this backdrop, economic sanctions and oil and gas supply disruptions in 2022 and 2023 (linked to the war in Ukraine), demands of post-COVID-19 economic rebound, and extreme weather events affecting energy production have led to a sharp increase in energy prices and a rapid pursuit of new energy sources.^266^ In some places, this caused a reversion to dirty fossil fuels, including coal, and renewed oil and gas exploration.^77^ High energy prices and the associated inflation pushed governments to borrow heavily to subsidise energy bills. High energy prices led to soaring profits for global oil and gas firms of US$4 trillion in 2022, roughly 270% higher than the average of $1·5 trillion in recent years, with the bulk of these profits going to major oil and gas exporting states.^267,268^ Profits of six major oil companies reached a record of over $200 billion in 2022.^269–274^ Only minor taxes, if any, were applied to these windfall profits. Over half of oil and gas company outflows went to shareholder returns and debt repayments in 2022, the highest level in over 15 years; the $1·5 trillion returned to shareholders in 2020–22 could have fully covered the clean fuel investments required between 2023 and 2030 in the International Energy Agency’s Net Zero Emissions Scenario. Although clean energy spending by oil and gas companies quadrupled between 2020 and 2022 to roughly $20 billion, this spending only represented roughly 4% of their overall capital investment.^275^ In fact, given the current situation of hyperprofitability, a number of oil and gas companies have backtracked on clean renewable energy activities and increased oil and gas production plans.^276–280^

However, expensive fossil fuel imports and an increased desire for energy security are also driving uptake in renewables, and the uptick in coal appears to be temporary.^281,282^ The USA’s 2022 Inflation Reduction Act (IRA) included $369 billion for improving energy security and accelerating the zero-carbon energy transition and is expected to increase the reduction in US greenhouse gas emissions from 26% of 2005 levels to 40% of 2005 levels by 2030.^76^ The subsidising of renewable energy under the IRA has sparked a subsidy race,^283^ causing the EU, in response, to launch the Green Deal Industrial Plan in 2023 to seek to keep green projects in Europe.^384,285^

The urgent need to accelerate the net-zero transition means fossil fuel firms must be pressed to abandon a near-term focus on returns and redirect profits to fund the green transition.^286^

## Panel 8 Protecting health through climate legislation and litigation

The law is an important arena for action on health and climate change.^331,332^ Across different countries, climate-related legislation has been passed at national and subnational levels, and citizens have turned to litigation to force governments to strengthen mitigation and adaptation actions. These legal instruments have incorporated health in various ways.

### Health impact and adaptation

A growing number of countries have enacted laws establishing national emissions reduction targets and strengthening climate governance.^333,334^ Many of these laws include direct references to the health impacts of climate change—both in terms of justifying the legislation and including adaptation provisions focusing on citizens’ health. For example, Portugal’s 2021 Framework Climate Law includes an article on public health that stipulates that the government must support the “assessment of global and national risks and the preparation of action, prevention and contingency plans in the face of extreme climatic phenomena, the emergence of new diseases or the worsening of the incidence of diseases as a result of climate change”.^335^ Climate laws that include health adaptation provisions have been introduced in places such as Ecuador,^336^ Nauru,^337^ and the EU.^338^

### Mitigation and health co-benefits

Other countries have introduced laws targeting emissions reductions and environmental protections that explicitly refer to health co-benefits (eg, North Macedonia’s Law on Ambient Air Quality).^339^ An emphasis on health can also be seen in laws that might not primarily target climate change. For example, the USA’s 2022 Inflation Reduction Act (IRA) aims to curb inflation by, among other steps, investing in domestic energy production and promoting healthy, zero-emission energy.^340^ Through various health and climate-related provisions, the IRA could play a crucial role in protecting health from the adverse impacts of climate change.^341^

### Litigation

Evidence relating to the health impacts of climate change has also been used in litigation cases, such as civil legal proceedings against governments, to accelerate climate change action.^342^ In a landmark court case in Germany in 2020, a group of young people filed a legal challenge to Germany’s Federal Climate Protection Act on the basis that the target of reducing greenhouse gas emissions by 55% between 1990 and 2030 was insufficient for the country to meet its Paris Agreement targets, which therefore violated their human rights as protected in Germany’s constitution.^343^ A key component of the claimants’ case was evidence of the health impacts of climate change, which they argued violated their right to life and physical integrity.^344^ The court ruled in their favour, leading to the German Government revising their mitigation targets.^343^ An emphasis on the health impacts of climate change can also be observed in other recent and ongoing litigation cases at the international level. For example, the UN Human Rights Committee ruling in favour of Indigenous Torres Straight Islanders—who had lodged a complaint against the Australian Government for failing to adequately cut emissions or implement necessary adaptation measures—referred to the health impacts of climate change for Indigenous people on the low-lying islands.^345^ A focus on the health dimensions of climate change is also likely to be central to the climate-related litigation pursued by Small Island Developing States (SIDS). In December, 2022, the Commission of Small Island States on Climate Change and International Law made an unprecedented formal request for an advisory opinion from the International Tribunal for the Law of the Sea.^346^ The case documents include a strong focus on the health impacts of climate change, particularly in relation to how these undermine the right to health and the right to a clean, healthy, and sustainable environment for citizens of SIDS.^347^

Along with being effective instruments for action on health and climate change, legislation and litigation can increase engagement in other key societal domains, including in the media, the public, and the corporate sector.^331,348^
