# Supplementary material for: The 2023 report of the Lancet Countdown on health and climate change: the imperative for a health-centred response in a world facing irreversible harms
Source: Lancet. Author manuscript; Available in PMC 2024 Nov 15. (PMC7616810; doi:10.1016/S0140-6736(23)01859-7)
Supplement: Supplementary appendix [file EMS200005-supplement-Supplementary_appendix.pdf]

THE LANCET

Supplementary appendix

This appendix formed part of the original submission and has been peer reviewed. We post it as supplied by the authors.

Supplement to: Romanello M, di Napoli C, Green C, et al. The 2023 report of the *Lancet* Countdown on health and climate change: the imperative for a health-centred response in a world facing irreversible harms. *Lancet* 2023; published online Nov 14. https://doi.org/10.1016/S0140-6736(23)01859-7.

The 2023 Report of the *Lancet* Countdown on Health and Climate Change

Appendix

Table of Contents

The 2023 Report of the *Lancet* Countdown on Health and Climate Change 2
Part A: Evolving Regional Progress and Inequities in Climate Change and Health 4
Part B: Taking Stock of Progress on Climate Change and Health 10
Section 1: Health Hazards, Exposures, and Impacts 10
1.1: Heat and Health 10
Indicator 1.1.1: Exposure to Heating 10
Indicator 1.1.2: Exposure of Vulnerable Populations to Heatwaves 14
Indicator 1.1.3: Heat and Physical Activity 20
Indicator 1.1.4: Change in Labour Capacity 25
Indicator 1.1.5: Heat-Related Mortality 40
1.2: Health and Extreme Weather-related Events 47
Indicator 1.2.1: Wildfires 47
Indicator 1.2.2: Drought 59
Indicator 1.2.3: Extreme weather and Sentiment 64
Indicator 1.3: Climate Suitability for Infectious Disease Transmission 70
Indicator 1.4: Food Security and Undernutrition 94
Section 2: Adaptation, Planning, and Resilience for Health 102
2.1: Assessment and Planning of Health Adaptation 102
Indicator 2.1.1: National Assessments of Climate Change Impacts, Vulnerability and Adaptation for Health 102
Indicator 2.1.2: National Adaptation Plans for Health 104
Indicator 2.1.3: City-Level Climate Change Risk Assessments 106
2.2: Enabling Conditions, Adaptation Delivery and Implementation 109
Indicator 2.2.1: Climate Information for Health 109
Indicator 2.2.2: Air Conditioning: Benefits and Harms 110
Indicator 2.2.3: Urban Green Space 112
Indicator 2.2.4: Global Multilateral Funding for Health Adaptation Programs 127
Indicator 2.2.5: Detection, Preparedness, and Response to Health Emergencies 129
2.3: Vulnerabilities, Health Risk, and Resilience to Climate Change 131
Indicator 2.3.1: Vulnerability to Mosquito-Borne Diseases 131
Indicator 2.3.2: Lethality of Extreme Weather Events 133
Indicator 2.3.3: Migration, Displacement and Rising Sea Levels 137
Section 3: Mitigation Actions and Health Co-Benefits 140
Indicator 3.1: Energy Use, Energy Generation, and Health 140
3.1.1: Energy Systems and Health 140
3.1.2: Household Energy Use 144
3.1.3: Sustainable and Healthy Road Transport 147
3.2: Air Pollution and Health Co-benefits 149
Indicator 3.2.1: Mortality from Ambient Air Pollution 149
Indicator 3.2.2 Household Air Pollution 150
3.3: Food, Agriculture, and Health Co-benefits 152
Indicator 3.3.1: Emissions from Agricultural Production and Consumption 152
Indicator 3.3.2 Diet and Health Co-benefits 156

Indicator 3.4: Healthcare Sector Emissions 164
Section 4: Economics and Finance 164
4.1: The Economic Impact of Climate Change and its Mitigation.................... 165
Indicator 4.1.1: Economic Losses due to Weather-Related Extreme Events 165
Indicator 4.1.2: Costs of Heat-related Mortality 167
Indicator 4.1.3: Potential Loss of Earnings from Heat-Related Labour Capacity Reduction 171
Indicator 4.1.4: Costs of the Health Impacts of Air Pollution 179
4.2: The Economics of the Transition to Net Zero-Carbon Economies 182
Indicator 4.2.1: Zero-carbon Energy Investment 182
Indicator 4.2.2: Employment in Renewables and Fossil Fuel Industries 183
Indicator 4.2.3: Funds Divested from Fossil Fuels 185
Indicator 4.2.4: Net Value of Fossil Fuel Subsidies and Carbon Prices 187
Indicator 4.2.5: Production and Consumption-based Attribution of CO_2 and $PM_{2.5}$ Emissions 192
Indicator 4.2.6: Compatibility of Fossil Fuel Company Strategies with the Paris Agreement 201
Indicator 4.2.7: Fossil Fuel and Green Bank Lending 204
Section 5: Public and Political Engagement 206
Indicator 5.1: Media Engagement in Health and Climate Change 206
Indicator 5.2: Individual Engagement in Health and Climate Change 217
5.3: Scientific Engagement in Health and Climate Change 240
Indicator 5.3.1: Scientific Articles on Health and Climate Change 1990-2022 240
Indicator 5.3.2: Scientific Engagement on the Health Impacts of Climate Change 245
5.4: Political Engagement in Health and Climate Change 252
5.4.1. Government Engagement 252
5.4.2: Engagement by International Organisations 277
Indicator 5.5: Corporate Sector Engagement in Health and Climate Change 291

The 2023 Report of the *Lancet* Countdown on Health and Climate Change

This appendix provides methodological details on each of the *Lancet* Countdown's indicators, alongside data sources used and caveats. Wherever suitable, future plans for the indicators and further analysis are also presented.

Wherever possible and appropriate, each indicator is disaggregated into very high, high, medium, and low human development index (HDI) country groups, as defined by the UNDP. For this purpose, the attained level of HDI in the latest year of data available during the writing of this report (2021) is used, acknowledging that the achievement of a HDI level is the product of several years of work towards improving the parameters that define it. The HDI captures three core dimensions: a long and healthy life (using life expectancy as a proxy), education (monitored by the mean of years of Schooling in a given country), and standard of living (using per-capita gross national income as a proxy).

With the addition of the regional section of this report, indicators are also disaggregated by world region (often referred to as "LC Regions" in analyses and figures), as shown in Table 1. To allow for global coverage, the country groupings often differ from the regions covered by the *Lancet* Countdown's regional centres.

Table 1: List of countries included in each global region for regional analyses.

Region	Countries Included
**Africa**	Algeria, Angola, Benin, Botswana, Burkina Faso, Burundi, Cameroon, Central African Republic, Chad, Congo, Cote d'Ivoire, Democratic Republic of the Congo, Djibouti, Egypt, Equatorial Guinea, Eritrea, Eswatini, Ethiopia, Gabon, Gambia, Ghana, Guinea, Kenya, Lesotho, Liberia, Libya, Madagascar, Malawi, Mali, Mauritania, Morocco, Mozambique, Namibia, Niger, Nigeria, Rwanda, Senegal, Sierra Leone, Somalia, South Africa, South Sudan, Sudan, Togo, Tunisia, Uganda, United Republic of Tanzania, Zambia, Zimbabwe
**Asia**	Afghanistan, Armenia, Azerbaijan, Bahrain, Bangladesh, Bhutan, Brunei Darussalam, Cambodia, China, Cyprus, Democratic People's Republic of Korea, Georgia, India, Indonesia, Iraq, Islamic Republic of Iran, Israel, Japan, Jordan, Kazakhstan, Kuwait, Kyrgyzstan, Lao People's Democratic Republic, Lebanon, Malaysia, Mongolia, Myanmar, Nepal, Occupied Palestinian territory, Oman, Pakistan, Philippines, Qatar, Republic of Korea, Saudi Arabia, Sri Lanka, Syrian Arab Republic, Tajikistan, Thailand, Turkey, Turkmenistan, United Arab Emirates, Uzbekistan, Vietnam, Yemen
**Europe**	Albania, Andorra, Austria, Belarus, Belgium, Bosnia and Herzegovina, Bulgaria, Croatia, Czechia, Denmark, Estonia, Finland, France, Germany, Greece, Hungary, Iceland, Ireland, Italy, Latvia, Liechtenstein, Lithuania, Luxembourg, Malta, Monaco, Montenegro, Netherlands, North Macedonia, Norway, Poland, Portugal, Republic of Moldova, Romania, Russian Federation, San Marino, Serbia, Slovakia, Slovenia, Spain, Sweden, Switzerland, Ukraine, United Kingdom
**Northern America**	Canada and United States of America
**Oceania**	Australia and New Zealand
**SIDS**	Anguilla, Antigua and Barbuda, Aruba, Bahamas, Barbados, Belize, Bermuda, British Virgin Islands, Cabo Verde, Cayman Islands, Comoros, Cook Islands, Cuba, Curaçao, Dominica, Dominican Republic, Federated States of Micronesia, Fiji, Grenada, Guadeloupe, Guinea Bissau, Guyana, Haiti, Jamaica, Kiribati, Maldives, Marshall Islands, Martinique, Mauritius, Montserrat, Nauru, Niue, Palau, Papua New Guinea, Puerto Rico, Saint Kitts and Nevis, Saint Lucia, Saint Vincent and the Grenadines, Samoa, Sao Tome and Principe, Seychelles, Singapore, Sint Maarten, Solomon Islands, Suriname, Timor-Leste, Tonga, Trinidad and Tobago, Turks and Caicos, Tuvalu, US Virgin Islands, Vanuatu
**South and Central America**	Argentina, Bolivarian Republic of Venezuela, Bolivia, Brazil, Chile, Colombia, Costa Rica, Ecuador, El Salvador, French Guiana, Guatemala, Honduras, Mexico, Nicaragua, Panama, Paraguay, Peru, Uruguay

Unless otherwise specified, the indicators that incorporate retrospective climate data make use of the climate reanalysis datasets, mostly ERA5, but also including ERA5-Land and ORAS5. These datasets incorporate vast amounts of historical observations, including those from satellites, to provide the most complete description of the observed climate as it has evolved during recent decades. Due to their temporal and geographical coverage, these are the most appropriate data for the purposes of the *Lancet* Countdown indicators. Slight discrepancies might exist between reanalysis datasets, and other types of retrospective climatological modelling, which however would only have slight impacts on findings of the indicators here presented.

All monetary values in the *Lancet* Countdown are expressed in 2022 US dollars, unless stated otherwise in the main text or cited sources.

Part A: Evolving Regional Progress and Inequities in Climate Change and Health

The data in part A provides a regional breakdown of the global indicators of the 2023 *Lancet* Countdown report. Using global indicators at a regional level, the data below in many cases will not match exactly that presented in the forthcoming regional reports of the *Lancet* Countdown, which often use local data sources available with higher resolution, coverage and quality. As such, it is intended to support comparisons between regions, rather than provide a thorough assessment of each individual region as is provided in the reginal reports of the *Lancet* Countdown..

Table 2: Indicator data per region. Given different geographical breakdowns and database used, the numbers below might differ from those in the regional reports that will be published in the first half of 2024, as these use different geographical breakdowns and often different data sources than available globally. Red tones indicate higher impacts, worsening trends, or climate inaction as per the first value in each cell. Green tones indicate improving trends, reduced impacts, or more climate action as per the first value in each cell.

Indicator Number	Description of Indicator Component	Africa	Asia	Europe	North America	Oceania	SIDS	South & Central America
**1.1.4**	**Average annual potential labour hours lost per worker in 2013-2022**	161 hours per worker	189 hours per worker	3 hours per worker	14 hours per worker	7 hours per worker	116 hours per worker	59 hours per worker
**1.1.5**	**Average annual change in heat-related deaths 2017-2022 compared to 2000-2005**	+ 11 deaths per 100,000	+ 0.6 deaths per 100,000	+ 9 deaths per 100,000	+ 3 deaths per 100,000	- 1 death per 100,000	+ 2 deaths per 100,000	+ 7 deaths per 100,000
**1.1.5**	**Change in average annual number of days of health-threatening high temperatures n 2018-2022 compared to 1998-2002**	+32 days	+24 days	+13 days	+15 days	+11 days	+49 days	+26 days
**1.1.5**	**Proportion of days of stressful temperatures in 2018-2022 that were more likely because of climate change**	80.3%	53.7%	37.8%	35.0%	30.2%	85.5%	77.5%

Indicator Number	Description of Indicator Component	Africa	Asia	Europe	North America	Oceania	SIDS	South & Central America
1.1.5	Days of health-threatening temperatures per year attributable to climate change in 2018-2022	79.6	47.8	25.5	28.0	18.5	103.4	72.4
1.1.5	Average heat-related mortality in 2017-2022, and average annual change from 2000-2005	43 deaths per 100,000 (+11 deaths per 100,000)	36 deaths per 100,000 (+0.6 deaths per 100,000)	70 deaths per 100,000 per year (+9 deaths per 100,000)	37 deaths per 100,000 (+3 deaths per 100,000)	35 deaths per 100,000 (-1 deaths per 100,000)	11 deaths per 100,000 (+2 deaths per 100,000)	30 deaths per 100,000 (+7 deaths per 100,000)
1.2.2	Proportion of land area affected by at least one month of extreme drought in 2013-2022 vs 1951-1960	9% vs 64%	17% vs 43%	27% vs 36%	28% vs 33%	14% vs 55%	9% vs 45%	13% vs 53%
1.3	Absolute change in dengue R0, comparing 2012-2021 to 1951-1960 (Aedes albopictus mosquitos)	0.28	0.28	0.06	0.11	0.03	0.42	1.03
1.3	Absolute change in dengue R0, comparing 2012-2021 to 1951-1960 (Aedes aegypti mosquitos)	0.24	0.31	-3.8E-05	0.41	0.03	0.36	0.86
1.3	Proportion of coastline suitable for vibrio transmission in 2022 (and average annual change in percentage of coastline suitable between 1982 and 2022)	16.6% (-2km)	16.6% (+83 km)	16.8% (+142 km)	2.9% (+52km)	0.8% (-0.8km)	2.9% (+13km)	16.8% (+42km)

Indicator Number	Description of Indicator Component	Africa	Asia	Europe	North America	Oceania	SIDS	South & Central America
**1.3**	**Average annual change in Vibriosis incidence**	+1074 cases	+4998 cases	+342 cases	+421 cases	+0.4 cases	+10 cases	+570 cases
**1.4**	**Change in sea surface temperature in 2022, compared to 1981-2010**	+0.10°C	+0.54°C	+0.83°C	+0.73°C	+0.55°C	+0.51°C	-0.08°C
**2.1.3**	**Proportion of cities responding to CDP survey that have completed a climate risk and vulnerability assessment (number of cities with completed assessment/total cities responding in region)**	62% (43/69)	51% (117/231)	82% (141/171)	80% (175/220)	92% (23/25)	50% (1/2)	56% (149/268)
**2.3.3**	**Proportion of regional population settled within 1m of sea level**	0.6%	2.8%	1.5%	0.6%	0.7%	2.0%	0.4%
**3.1.1**	**Average energy-related emissions per person in 2020**	0.97 tCO2/person	3.9 tCO2/person	6.2 tCO2/person	12.9 tCO2/person	13.4 tCO2/person	3.2 tCO2/person	2.1 tCO2/person
**3.1.1**	**Change in carbon intensity of energy system, 2020 compared to 1992 and (time needed to fully decarbonise at pace seen between 2011 and 2020)**	+3% (387.2 years)	+10% (160.4 years)	-22% (81.7 years)	-15%, (80.3 years)	-7%, (220.2 years)	-14%, (50.0 years)	-5%, (91.6 years)
**3.1.1**	**Proportion of total primary energy supply coming from coal in 2020**	13.6%	42.6%	12.4%	9.9%	27.1%	2.2%	5.0%

Indicator Number	Description of Indicator Component	Africa	Asia	Europe	North America	Oceania	SIDS	South & Central America
3.1.1	Proportion of total primary energy supply coming from renewable energies in 2020	1.0%	2.7%	3.0%	2.4%	6.0%	0.4%	2.7%
3.2.1	Deaths from fuel-derived PM2.5 in 2020	77,000	1300000	280,000	17,000	158	565	24,000
3.2.1	Mortality attributed to coal-derived PM2.5 in 2020	0.7 deaths per 100,000	11 deaths per 100,000	8 deaths per 100,000	0.8 deaths per 100,000	0.2 deaths per 100,000	2 deaths per 100,000	0.2 deaths per 100,000
3.2.2	Mortality attributed to dirty fuels' PM2.5 in 2020 (percentage from coal)	6 deaths per 100,000 (11.7%)	30 deaths per 100,000 (36.7%)	38 deaths per 100,000 (21.1%)	5 deaths per 100,000 (16.0%)	0.5 deaths per 100,000 (40.0%)	5 deaths per 100,000 (40.0%)	4 deaths per 100,000 (5.0%)
3.2.2	Proportion of household energy consumption from biomass and waste burning	83.6%	32.4%	11.2%	4.8%	10.8%	45.9%	33.1%
3.2.2	Proportion of household energy from clean energies	6.8%	33.4%	37.4%	48.3%	54.2%	34.7%	33.9%
3.3.1	Emissions per person from the consumption of red meat and dairy	0.42	0.36	0.35	0.58	1.05	0.25	0.66
3.3.2	Mortality from insufficient consumption of fruits and vegetables	25 deaths per 100,000	36 deaths per 100,000	77 deaths per 100,000	37 deaths per 100,000	52 deaths per 100,000	38 deaths per 100,000	31 deaths per 100,000
3.3.2	Mortality attributable to excess consumption of red and processed meat	3 deaths per 100,000	6 deaths per 100,000	37 deaths per 100,000 people	38 deaths per 100,000 people	29 deaths per 100,000 people	9 deaths per 100,000	13 deaths per 100,000

Indicator Number	Description of Indicator Component	Africa	Asia	Europe	North America	Oceania	SIDS	South & Central America
**4.1.3**	**Potential income losses due to heat-related labour hours lost, as percent of GDP**	4.1%	2.6%	0.1%	0.2%	0.1%	2.7%	1.3%
**4.1.3**	**Percent of heat-related labour income losses that were in agricultural sector**	81.4%	52.6%	38.6%	8.0%	11.6%	68.3%	41.3%
**4.2.4**	**Median net carbon price**	-43.7 $/t	-12.6 $/t	-1.4 $/t	+0.9 $/t	-9.6 $/t	-9.8 $/t	-19.4 $/t
**5.3.1**	**Number of peer-reviewed articles studying the region**	254	1095	305	398	80	51	142
**5.4.1**	**Proportion of countries mentioning health in first round of nationally determined contributions (total number NDCs)**	98% (47)	88% (42)	14% (43)	50% (2)	33% (3)	84% (38)	100% (16)
**5.4.1**	**Proportion of countries mentioning health in second round of nationally determined contributions (total number NDCs)**	98% (41)	88% (33)	92% (38)	100% (2)	0% (2)	100% (31)	100% (16)
**5.4.1**	**Average proportion of countries referring to the climate-health nexus in the United Nations General Debate in 2013-2020**	26.7%	22.8%	28.7%	35.0%	45.0%	44.9%	18.7%

Indicator Number	Description of Indicator Component	Africa	Asia	Europe	North America	Oceania	SIDS	South & Central America
		0.236105	0.312581	-3.8E-05	0.40631	0.027157	0.364097	0.856134

Part B: Taking Stock of Progress on Climate Change and Health

The indicators of the Lancet Countdown are designed and selected based on well-defined criteria, to ensure their capacity to track progress on health and climate change. These criteria are presented on the collaboration's website and reproduced below:[1]

All indicators must track an aspect of the relationship between health and climate change, well evidenced in the literature and not adequately covered through other indicators in the report; utilise data from a reliable source, available at adequate temporal and spatial scales to enable trends to be observed at a global level; and be updatable periodically, ideally annually or more regularly.

More specifically, all indicators must be:

- Meaningful: Track an aspect of the relationship between health and climate change that is well evidenced in the literature, and relevant at a global level.
- Relevant: The area being tracked by the indicator must be of relevance to policy and decision makers, and/or represent an important contribution to the field of science of climate change and health
- Scientifically sound and reproducible: The indicator must use a well-established, internationally accepted, and ideally previously published scientific methods.
- Temporally representative: The indicator should provide annual data for the recent past and to a year as recent as possible. It must be available across an adequate timescale to allow for attribution to climate change, where relevant.
- Geographically representative: The indicator should be ideally available at a country, or higher level of resolution. Its geographical coverage should be enough for global trends to be observed, covering at least 40 countries evenly distributed across the four World Bank income contexts, the four Human Development Index Groups, and the five WHO regions initially, with possibility of expansion to 150 countries at least. In the case of indicators tracking aspects relevant to restricted locations, over 80% of relevant countries must be covered by the indicator.
- Reliable and use updatable sources: The indicator should use data from a reliable source, fit for its purpose. Publicly available databases, and especially those developed by international organisations, governmental bodies or academic institutions, are preferred. Data sources must be regularly updated.

Section 1: Health Hazards, Exposures, and Impacts

Lead Author: Prof Elizabeth J. Z. Robinson

Research Fellow: Dr Claudia DiNapoli

1.1: Heat and Health

Indicator 1.1.1: Exposure to Heating

Indicator author

Dr Jonathan Chambers

Methods

The input data for this indicator have been extended for the 2023 report.

The indicator uses monthly temperature from European Centre for Medium-Range Weather Forecasts (ECMWF) ERA5 climate reanalysis dataset. From this, a baseline global mean temperature grid was first calculated as the average of summer temperatures (June, July, August for the northern hemisphere; December, January, February for the southern hemisphere) from 1986–2005, the same period used by the Intergovernmental Panel on Climate

Change (IPCC AR5).[2] Then global summer temperature changes relative to the 1986–2005 average were calculated for every grid point for every year and weighted by true pixel area to obtain a year-by-year global average. The 'population-weighted' average was calculated by weighting each grid cell by the fraction of the total world population contained within that grid cell. This method allows the difference between global effects of climate change and the effects experienced by the human population to be highlighted.

Population data from 2000 to present are from NASA GPWv4 dataset at 0.25° x 0.25° spatial resolution, the same as ECMWF ERA5. Population data from 1980 to 2000 are from the ISIMIP Histsoc dataset at 0.5° x 0.5° spatial resolution. In the main text both the Histsoc-derived findings (1980–2000) and the GPWv4-derived findings (2000–2021) are presented.

Data

1. Climate data from the European Centre for Medium-Range Weather Forecasts (ECMWF) ERA5 reanalysis.[3]
2. Population data from the NASA Socioeconomic Data and Applications Center (SEDAC) Gridded Population of the World (GPWv4) and The Inter-Sectoral Impact Model Intercomparison Project (ISIMIP) Histsoc dataset.[4,5]

Additional analysis

Population weighted temperatures are increasing 2.5 times faster than global mean temperatures (linear regression slope of 0.027˚C per year compared to 0.011˚C per year; p-value <0.05). In 2022, the global mean summer anomaly was 0.30˚C while the population weighted summer anomaly was 0.85˚C relative to the 1986–2005 baseline (Figure 1). Locally, these anomalies can be significantly higher at over 6˚C (Figure 2). Analysis of the anomaly grouped by HDI (Human Development Index) level and WHO (World Health Organisation) region show that the trends are global and do not indicate any particular difference across levels or regions (Figure 3, Figure 4).

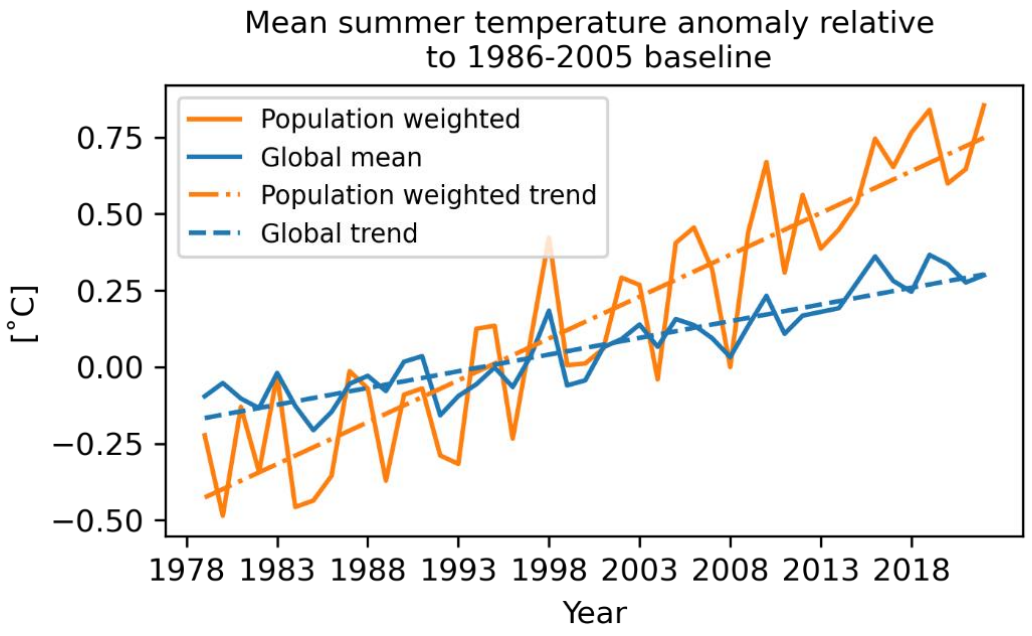

Figure 1: Global mean trends of summer temperature anomaly compared to the population weighted trend (relative to the 1986–2005 baseline). Results before 2000 are drawn from the 2020 edition of the *Lancet* Countdown and are calculated on the lower 0.5˚ grid resolution.

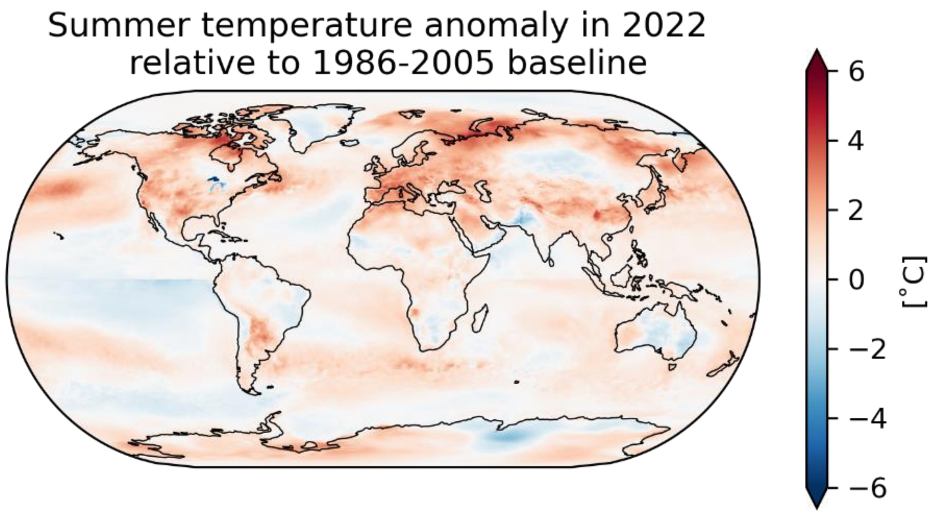

Figure 2: Map of summer temperature anomaly for 2022 relative to the 1986–2005 baseline.

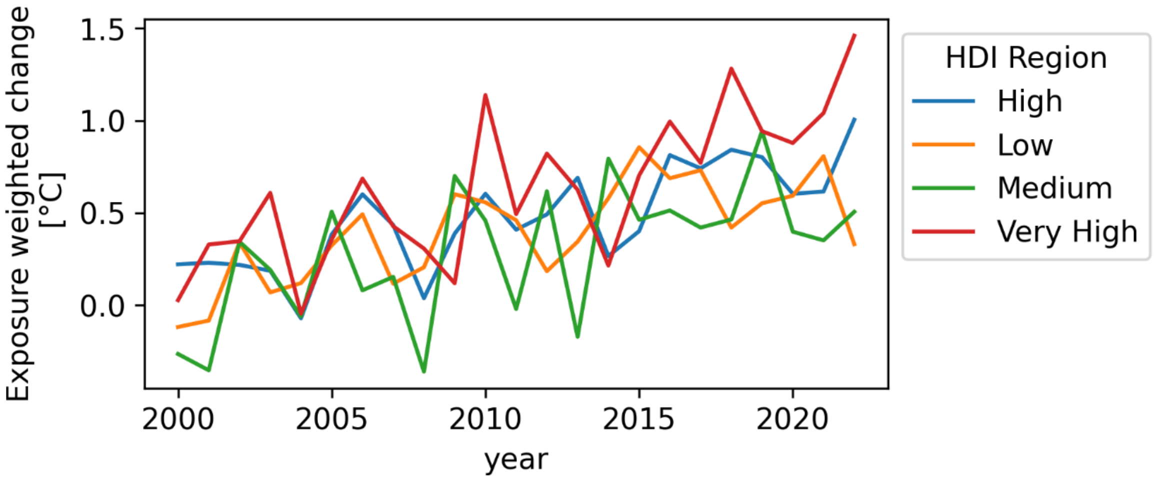

Figure 3: Exposure weighted change in summer temperatures relative to 1986–2005 baseline by HDI level.

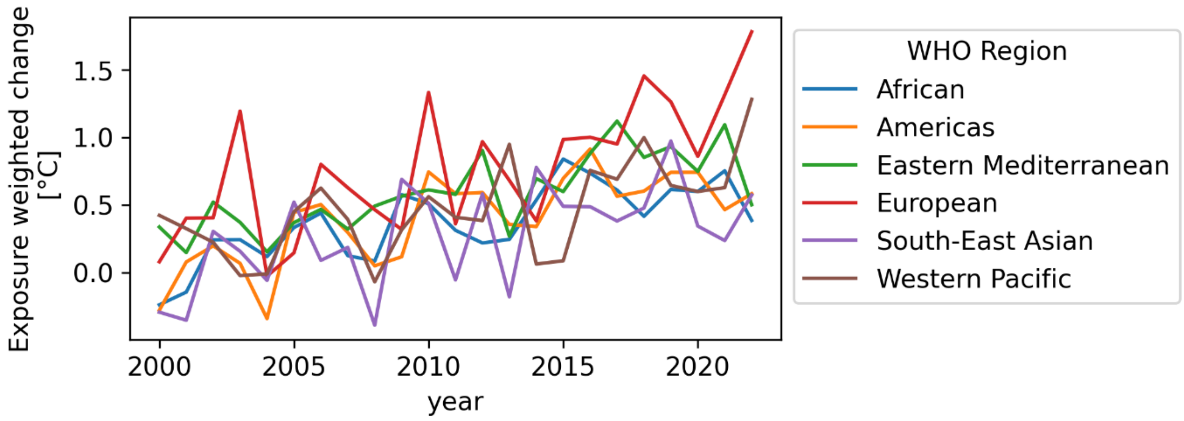

Figure 4: Exposure weighted change in summer temperatures relative to 1986–2005 baseline by WHO region.

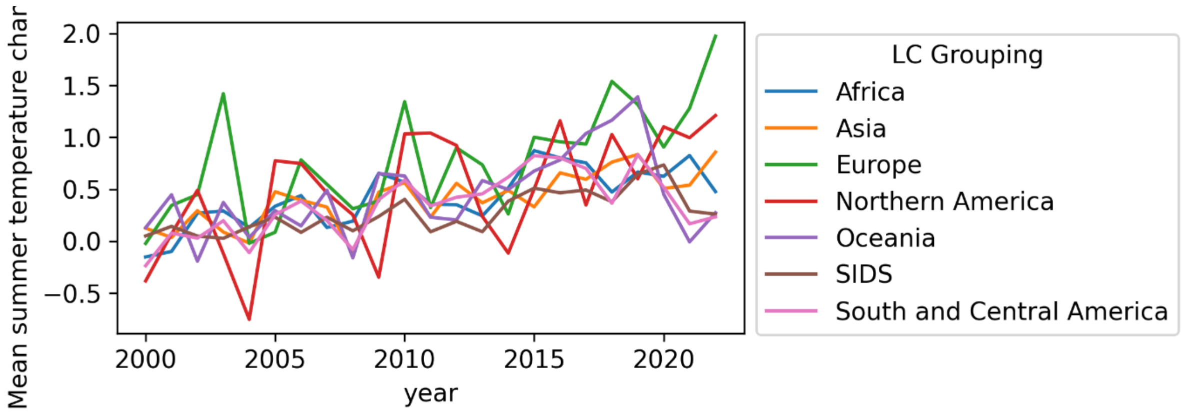

Figure 5: Exposure weighted change in summer temperatures relative to 1986–2005 baseline by *Lancet* Countdown group region.

Indicator 1.1.2: Exposure of Vulnerable Populations to Heatwaves

Indicator author

Dr Jonathan Chambers

Methods

The input data for this indicator have extended for the 2023 report and include future climate projections.

The indicator defines a heatwave as a period of 2 or more days where both the minimum and maximum temperatures are above the 95th percentile of the local climatology (defined on the 1986–2005 baseline). This reflects the definition from published scientific literature on the topic.[6] It also aims to capture the health effects of both direct heat extremes (i.e., caused by high maximum temperatures) and the problems associated with lack of recovery (i.e., caused by high minimum temperatures) over persisting hot periods.[7] The gridded 95th percentile of daily minimum and maximum temperatures, taken from the European Centre for Medium-Range Weather Forecasts (ECMWF) ERA5 dataset, were calculated on a 0.25° x 0.25° global grid for 1986–2005. For each year from 1980 to 2020, the number of heatwave events and total days of heatwaves per year was calculated according to the definition above.

Data were used from ISIMIP3b for the baseline period 1995–2014 and 5 GCM variants for SSP1-2.6 and SSP3-7.0 were used for future climate projections. Days of heatwave per year were calculated for each year 1995–2014, 2021–2040, 2041–2060, and 2081–2100 at a 0.5° x 0.5° grid resolution. The number of days of heatwave per year per grid cell was averaged for each time period.

Vulnerable populations are defined as those above the age of 65 and infants between 0 and 1 years old. Previous research has identified these groups as being particularly vulnerable to heatwave impacts on health.[8]

Data inspection has shown that increasing heatwave length can result in fewer discrete heatwave events as they merge into single long events – this is therefore better captured by the person-days metric. To reflect that, the exposure of vulnerable populations to heatwaves is computed as person-days, i.e., by multiplying the number of heatwave days by vulnerable population count. In this way, the indicator captures both the changes in duration and changes in frequency of heatwaves, as well as the changing demographics that might mean more vulnerable people are at risk.[9]

Population and demographic data from NASA GPWv4 were used for the period 2000–2020 as the resolution matches ECMWF ERA5's. For the period pre-2000, the ISIMIP Histsoc dataset was used after being up-sampled to a 0.25° x 0.25° resolution via a 2D linear interpolation of population densities with land area data from NASA GPWv4. As the population data are discontinuous, there can be some inconsistencies between the pre and post 2000 values. Therefore, the indicator is presented as exposure to change rather than change in exposure, as this avoids calculating changes in population across the data discontinuity. The hybrid dataset, available on open access[9] and new to last year's 2022 report, refers to a population older than 65 years old.

The number of births minus the mortality rate of children under 1 was used as an approximation of the number of children under 1 year old. The United Nation World Population Prospects (UN WPP) data for birth rates were used. UN WPP provides Crude Birth Rate (CBR) and Infant Mortality Rate (IMR) values per country as averages for 5-year periods. To estimate the spatial distribution of births within a country, it was assumed that the spatial distribution of children under one year of age is the same as the spatial distribution of children under 5 as given by the NASA GPWv4 dataset. Furthermore, it was assumed that the IMR within a country is constant for all locations, as sub-national data cannot be applied for this study. For each country, the total number of births was calculated for the mid-period year of the 5-year time periods as *Country population * CBR * (1 - IMR)*. Spatial weighting matrices were derived from the NASA GPWv4 demographic data for under-5s and used to estimate the total births and number of infants for each grid cell for each country. Finally, the estimates for the years in between the mid-period years were calculated through linear interpolation.

The heatwave exposure projections were obtained by multiplying the heatwave days per grid cell averaged over each time period by the vulnerable population per grid cell in the middle of the time period.

Data

1. Climate data from the European Centre for Medium-Range Weather Forecasts (ECMWF) ERA5 reanalysis.[10]
2. Future climate projections from ISIMIP 3b protocol.[11]
3. Hybrid gridded demographic data for the world, 1950–2020, 0.25° resolution.[12]
4. Demographic data from the United Nation World Population Prospects (UN WPP).[13]
5. Future demographic data from Briggs et al 2022.[14]

Caveats

In order to estimate the time evolution of demographics, data from diverse sources were combined in order to obtain estimates of both the spatial and temporal characteristics. This has been subject to limited validation. Some regions have limited demographic data. Others show changes in political boundaries which can cause discontinuities in the spatial assignment of demographic values.

Future form of the indicator

Future versions of the indicator aim to use ECMWF ERA5-Land data at 0.1° x 0.1° spatial resolution. The increased data volume at the global level, plus the need to adapt corresponding population data, requires upgrades to the data processing.

Additional analysis

Figure 6 summarises the change in number of heatwave days in 2022 relative to the baseline. Intense events in Western USA, central Europe, Russia, the Middle east, Northwest Africa, Central Africa, South-West Africa and Madagascar are evident. Figure 7 highlights that absolute exposures are larger in the over-65 age group. However, as shown in Figure 10, in the ‘low’ HDI class countries the exposure of over 65s is much lower than the other classes whereas the values for infants follow the same pattern as the other classes. This is likely related to lower life expectancy in countries in the ‘low’ HDI class. This trend is not reflected in the breakdown by country or WHO region (Figure 8, Figure 11).

To better understand the magnitude of changes in heatwave exposure, comparisons of the total exposure counts (i.e., not relative to the baseline heatwave count) between first and second decades of the 20th century are carried out. Figure 9 illustrates that some regions (notably Africa) have experienced changes of over +400% person-days of heatwave (i.e., 5 times as many person-days) between these two decades.

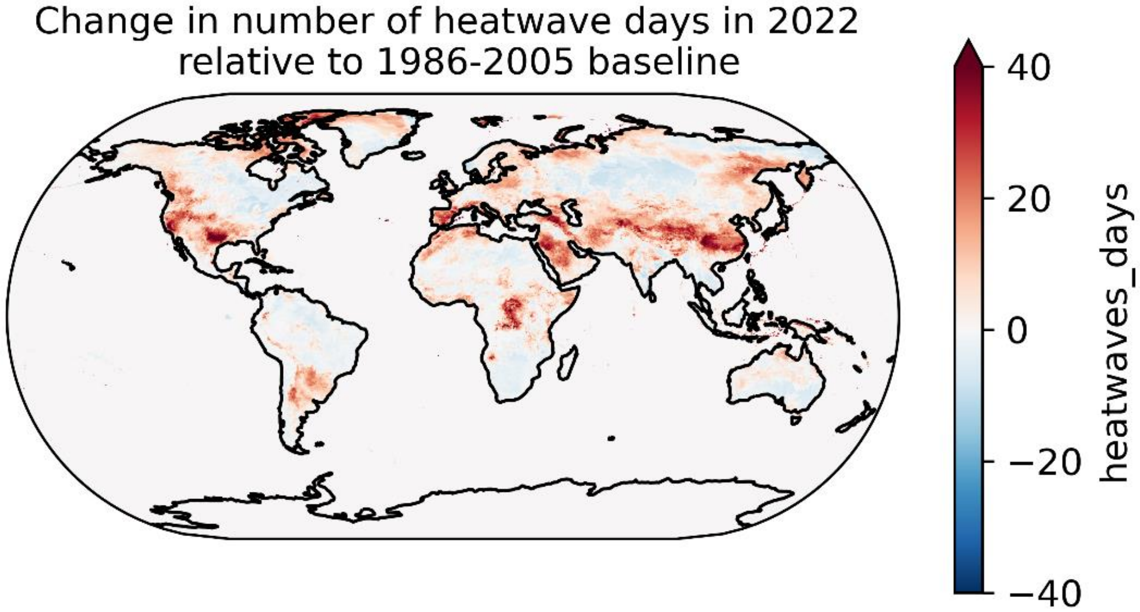

Figure 6: Map of the change in number of heatwave days over land in 2022 relative to the 1986–2005 baseline.

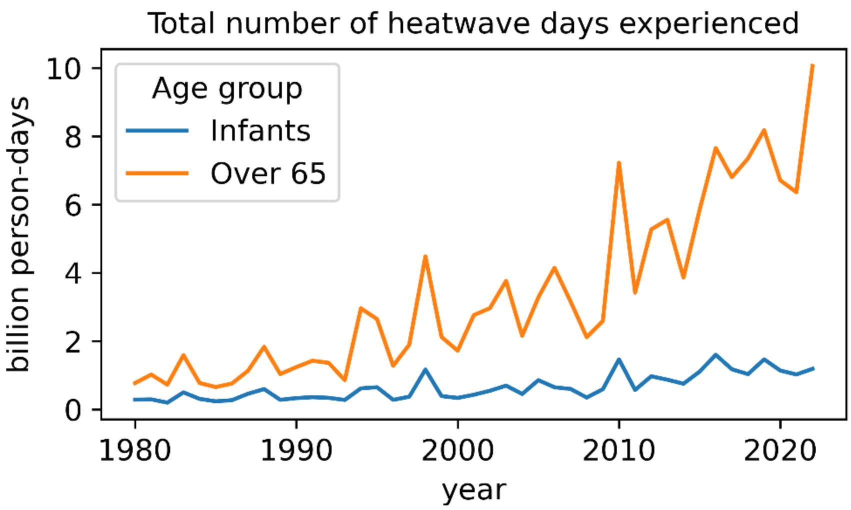

Figure 7: Total person-days of heatwave experienced by people over 65 and infants under 1 year old to heatwave days. ISIMIP population data is used for 19802000, GPWv4 population data is used for 2000–2022.

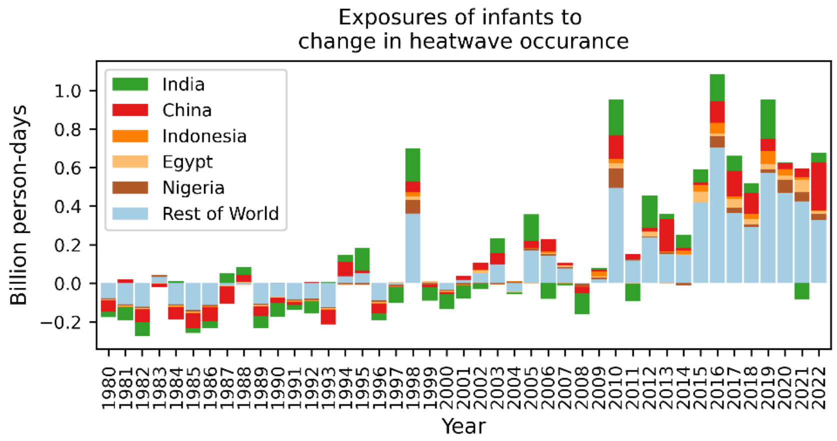

a)

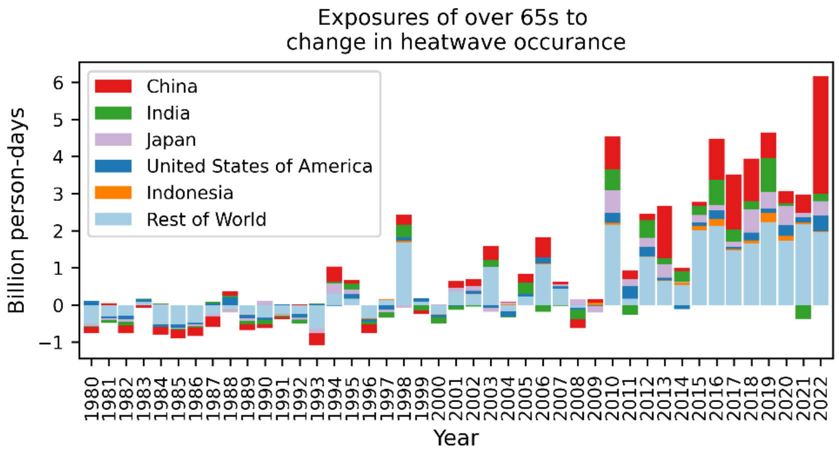

b)

Figure 8: Total exposure of a) infants and b) people over 65 to change in number of heatwave days relative to the 1986–2005 baseline mean number of days.

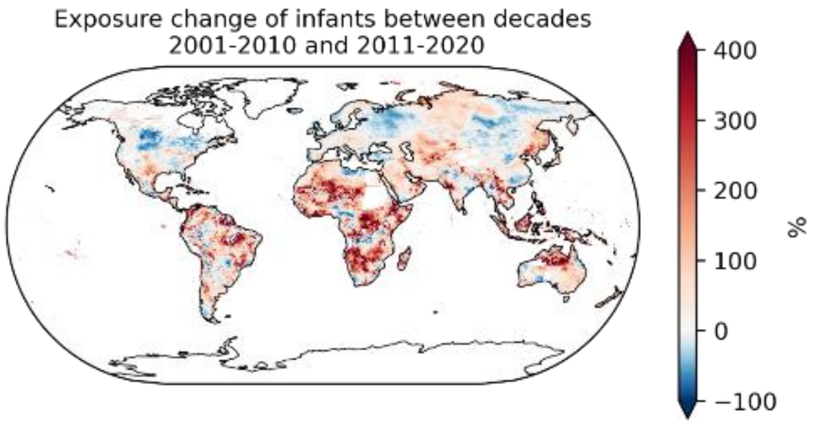

a)

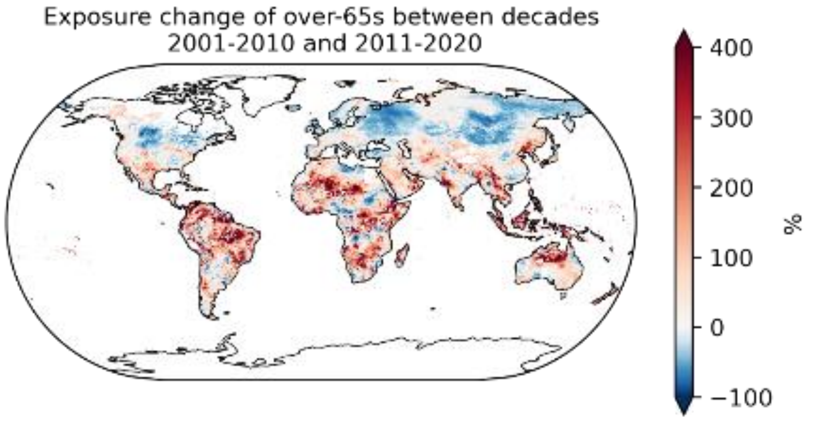

b)

Figure 9: Percentage change in heatwave exposure person-days per year between the 10-year average of 2001–2010 and 2011–2020 for a) infants and b) over-65s.

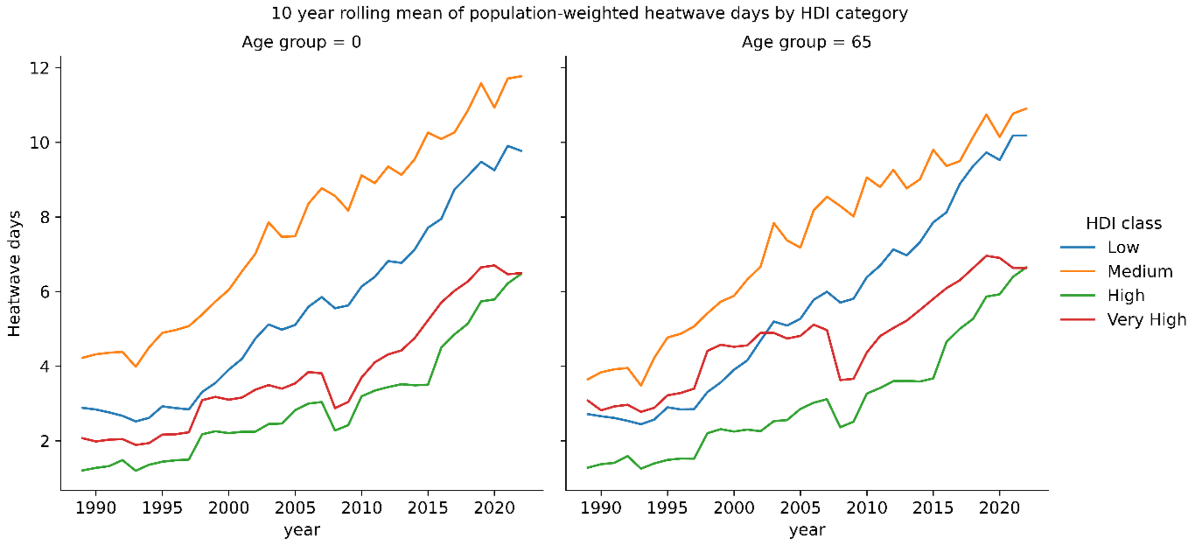

Figure 10: 10-year rolling mean of exposure weighted heatwave days aggregated by HDI level.

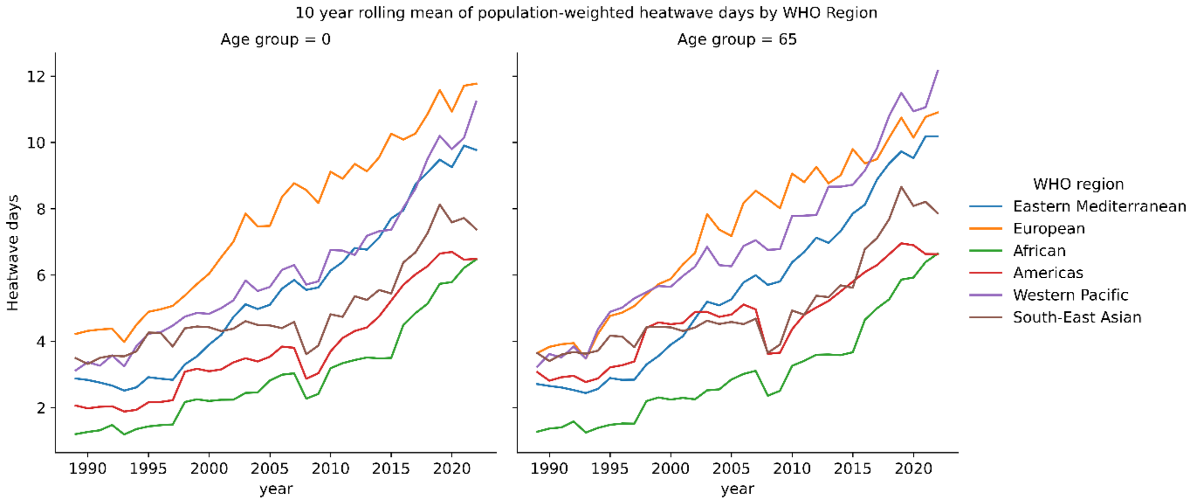

Figure 11: 10-year rolling mean of exposure weighted heatwave days aggregated by WHO region.

Indicator 1.1.3: Heat and Physical Activity

Indicator authors

Dr Troy J Cross, Dr Samuel H Gunther, Prof Ollie Jay, Dr Jason KW Lee

Methods

The methodology for this indicator has been updated and improved from the 2022 report of the *Lancet* Countdown.[15] In addition to calculating our indicator for different physical activity intensities, our estimation of moderate-, high–, and extreme-risk hours now incorporates the thermal effects of solar radiation by restricting the analyses to local sunlight hours only, as the underlying policy assumes solar radiation and is therefore not applicable to night-time hours. We have also included results for moderate-intensity as well as light-intensity exercise.

Hourly temperature, and dew point temperature data were retrieved from European Centre for Medium–Range Weather Forecasts (ECMWF) ERA5 climate reanalysis datasets. While ERA5 data are available from 1979, data from the years 1991 to 2022 were considered for the purposes of this analysis.

Heat stress risk was estimated from these variables in accordance with the 2021 Sports Medicine Australia Extreme Heat Policy, which stratifies estimated heat stress risk into four categories – low, moderate, high, and extreme – based on ambient temperature and relative humidity.[16] Sports and activities are further classified into five risk classification groups – ranging from leisurely walking to mountain biking – based on intensity of the activity and clothing worn.[16] The present analysis includes assessments of heat stress risk for Risk Classification 1, which we term "low intensity" (e.g., leisurely walking) and Risk Classification 3, which we term "moderate intensity" (e.g., jogging, cycling).

The number of hours in each grid cell with a recorded temperature and humidity combination that exceeded at least the threshold for "moderate", "high", and "extreme" heat stress risk for Risk Classifications 1 and 3 were tabulated for each year from 1990 to 2022. Specifically, the temperature–dependent humidity thresholds were defined using the following functions:

For Risk Classification 1 (light intensity):
Moderate heat stress risk:
$f(x) = 2762.197061 – 381.325699*x + 22.540429*x^2 – 0.686335*x^3 + 0.010572*x^4 – 0.000066*x^5$
High heat stress risk:
$f(x) = 580.945552 – 34.947074*x + 0.861360*x^2 – 0.012029*x^3 + 0.000126*x^4 – 0.000001*x^5$
Extreme heat stress risk:
$f(x) = 526.925669 – 26.920743*x + 0.492041*x^2 – 0.002915*x^3 – 0.000010*x^4 – 0.000000*x^5$

For Risk Classification 3 (moderate intensity):
Moderate heat stress risk:
$f(x) = 1329.057633 – 133.978794*x + 5.779444*x^2 – 0.128679*x^3 + 0.001425*x^4 –0.000006*x^5$
High heat stress risk:
$f(x) = 1242.422310 – 116.614628*x + 4.713013*x^2 – 0.098193*x^3 + 0.001011*x^4 – 0.000004*x^5$
Extreme heat stress risk:
$f(x) = 1507.777140 – 144.871106*T + 5.952094*x^2 – 0.124422*x^3 + 0.001276*x^4 –0.000005*x^5$

where: *x* is 2–meter temperature in a given hour and *f(x)* is 2–meter relative humidity derived from dew point temperature in a given hour.

These threshold functions are defined by Sports Medicine Australia as the boundary above which the risk of exertional heat illness changes, and the following preventive action should be taken:[16]

- "moderate" heat stress risk: additional rest breaks should be undertaken;
- "high" heat stress risk: active cooling strategies (e.g., water dousing) should be implemented;
- "extreme" heat stress risk: activities should be suspended due to excessive heat stress risk.

The functions in the 2021 Sports Medicine Extreme Heat Policy extend to a minimum ambient temperature of 26°C. Accordingly, any values recorded below this temperature, irrespective of ambient humidity, were determined as presenting a "low" heat stress risk.

Using the sunrise and sunset times determined using the same *suncalc* R package,[17] the hours of daylight for each day and derived hourly temperature in the sun were determined using equation 2 as described by Luedeling.[18] The average daily temperature and average relative humidity for a given day were then used to back–calculate dew point temperature (assuming it is constant throughout the day). Plotting hourly temperature against hourly relative humidity allowed calculation of the number of hours above the risk threshold. The total number of sunlight hours per year exceeding each threshold in each grid cell was then weighted by population. Population weighting was performed by multiplying the number of hours per year that at least exceeded each threshold by the population, as provided by the GPWv4.11 (UN) WPP Adjusted population count dataset, in the respective grid cell. The population–weighted potential hours at least exceeding each threshold in a single year were added up for all grid cells in a given country, and these values were divided by the total population of the country in that year to calculate the number of hours per person that at least exceeded the "moderate", "high" and "extreme" heat stress risk thresholds for each Risk Classification group.

For projection of future high–risk hours, ISIMIP3b bias–adjusted models were used to derive hourly estimates of future temperatures,[11] while future population data were obtained from a gridded dataset which combined CIESIN data and the gridded population data set version 2 by David Briggs.

Data

1. Climate data from the European Centre for Medium-Range Weather Forecasts (ECMWF) ERA5 reanalysis.[3]
2. Population data from the NASA Socioeconomic Data and Applications Center (SEDAC) Gridded Population of the World (GPWv4) - UN WPP-Adjusted Population Count, v4.11.[19]
3. Future temperature and humidity projections from the Inter-Sectoral Impact Model Intercomparison Project (ISIMIP3b).[11]

Caveats

Heat stress risk for each exercise intensity classification may differ among people due to various risk factors. For example, older adults may have age-related reduction in sweating which compromises their ability to keep cool and could elevate their exertional heat stress risk at a given combination of temperature and humidity. Other groups that may have a greater heat stress risk include young children, people wearing heavy clothing or living with disabilities or chronic diseases. A more detailed interpretation model of the heat effects of exercise would incorporate individual factors such as age, health status, and clothing.[20] While the 2023 analysis excluded hours without sunlight, the SMA policy assumes summertime strength of solar radiation. Heat stress risk in early spring and later autumn months may therefore be overestimated. It was also assumed that population averages for an entire year were applicable to each hourly grid cell, which may not be true, but this approach still provides a rough estimate of population assuming an even rate of influx and outflux from each cell at the country level.

Future form of the indicator

Results will be updated using each new year of available climate data and, as sports authorities issue their updated threshold guidelines, they will be expressed according to the latest policy developments. Subsequent versions of the indicator will integrate solar radiation data to overcome the current assumption of summertime levels of solar radiation intensity throughout the year. Future versions will explore the derivation of different heat stress risk thresholds for different subpopulation groups, e.g., acclimatized, unacclimatized, elderly.

Additional analysis

The primary analysis for this indicator was conducted for leisurely walking (risk classification 1) and cycling or running (risk classification 3) at the global level. But our analyses were also carried out at the level of LC country group, WHO country group, and HDI country group.

<u>LC Country Group</u>

For risk classification 1 (Figure 12), when separated by LC country group, relative to a baseline of 1991 to 2000 (inclusive) the number of hours that exceeded the threshold for moderate, high, and extreme heat stress risk increased in the period between 2013 and 2022 (inclusive), as follows:

Europe: by 27 (63% increase), 6 (112% increase), and 0 (0% increase) hours per person per year, respectively
Oceania: by 27 (18% increase), 5 (8%), and 1 (16%) hour per person per year, respectively
Northern America: by 115 (27% increase), 68 (31%), and 15 (81%) hours per person per year, respectively
South and Central America: by 198 (20% increase), 170 (37%), and 43 (84%) hours per person per year, respectively
SIDS: by 410 (20% increase), 429 (44%), and 39 (189%) hours per person per year, respectively
Africa: by 229 (20% increase), 218 (38% increase), and 62 (160% increase) hours per person per year, respectively
Asia: by 248 (16% increase), 269 (24%), and 211 (58%) hours per person per year, respectively

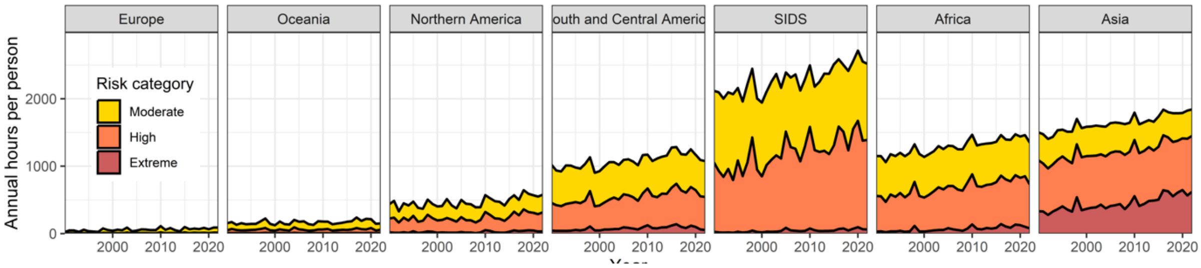

Figure 12: Average annual hours per person that light physical activity (SMA Risk Classification 1) entailed at least a moderate, high, or extreme heat stress risk by LC country (Europe, Oceania, Northern America, South and Central America, SIDS, Africa, Asia) grouping, 1991–2022.

For risk classification 3 (Figure 13), when separated by LC country group, in 2022 to 2013 (inclusive) the number of hours exceeding the threshold for moderate, high, and extreme heat stress risk increased, relative to a baseline of 1991 to 2000 (inclusive), as follows:

Europe: by 248 (15% increase), 216 (14% increase), and 1 (118% increase) hour per person per year, respectively
Oceania: by 43 (20% increase), 10 (11%), and 3 (16%) hours per person per year, respectively
Northern America: by 134 (26% increase), 90 (28%), and 36 (46%) hours per person per year, respectively
South and Central America: by 201 (17% increase), 190 (27%), and 97 (60%) hours per person per year, respectively
SIDS: by 393 (17% increase), 408 (26%), and 155 (104%) hours per person per year, respectively
Africa: by 248 (18% increase), 230 (26% increase), and 144 (78% increase) hours per person per year, respectively
Asia: by 248 (15% increase), 256 (19%), and 257 (39%) hours per person per year, respectively

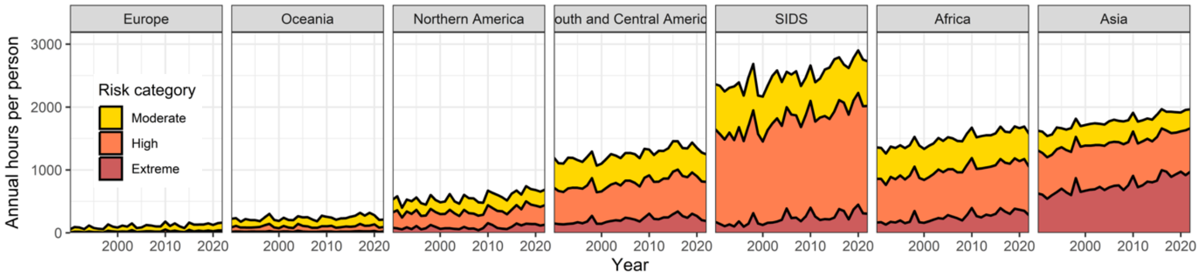

Figure 13: Average annual hours per person that moderate physical activity (SMA Risk Classification 3) entailed at least a moderate, high, or extreme heat stress risk by LC country (Europe, Oceania, Northern America, South and Central America, SIDS, Africa, Asia) grouping, 1991–2022.

WHO Country Group

For risk classification 1 (Figure 14), when separated by WHO country group, relative to a baseline of 1991 to 2000 (inclusive) the number of hours that exceeded the threshold for moderate, high, and extreme heat stress risk increased in the period between 2022 and 2013 (inclusive), as follows:
Africa: by 220 (18% increase), 229 (36% increase), and 72 (165% increase) hours per person per year, respectively
Americas: by 186 (23% increase), 146 (38%), and 33 (89%) hours per person per year, respectively
Eastern Mediterranean: by 196 (18% increase), 149 (22%), and 91 (29%) hours per person per year, respectively
Europe: by 42 (62% increase), 11 (85%), and 1 (60%) hour per person per year, respectively
SE Asia: by 252 (11% increase), 320 (19%), and 302 (54%) hours per person per year, respectively
Western Pacific: by 195 (20% increase), 204 (29% increase), and 119 (62% increase) hours per person per year, respectively

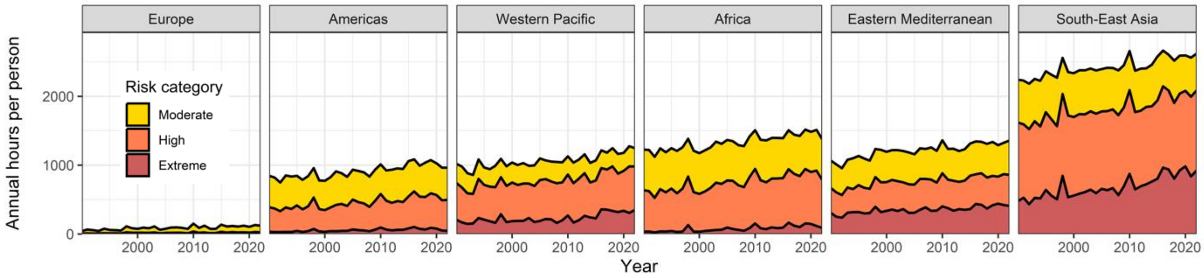

Figure 14: Average annual hours per person that light physical activity (SMA Risk Classification 1) entailed at least a moderate, high, or extreme heat stress risk by WHO country (Africa, Americas, Eastern Mediterranean, Europe, SE Asia, Western Pacific) grouping, 1991–2022.

For risk classification 3 (Figure 15), when separated by WHO country group, in 2022 to 2013 (inclusive) the number of hours exceeding the threshold for moderate, high, and extreme heat stress risk increased, relative to a baseline of 1991 to 2000 (inclusive), as follows:
Africa: by 246 (18% increase), 226 (24% increase), and 163 (80% increase) hours per person per year, respectively
Americas: by 197 (20% increase), 168 (28%), and 80 (62%) hours per person per year, respectively
Eastern Mediterranean: by 204 (15% increase), 180 (20%), and 111 (24%) hours per person per year, respectively
Europe: by 63 (52% increase), 23 (74%), and 3 (83%) hours per person per year, respectively
SE Asia: by 230 (9% increase), 283 (14%), and 338 (34%) hours per person per year, respectively
Western Pacific: by 201 (19% increase), 196 (23% increase), and 172 (46% increase) hours per person per year, respectively

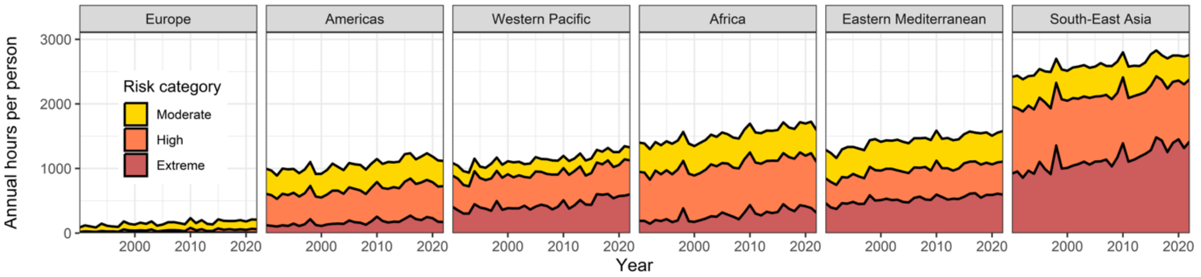

Figure 15: Average annual hours per person that moderate physical activity (SMA Risk Classification 3) entailed at least a moderate, high, or extreme heat stress risk by WHO country (Africa, Americas, Eastern Mediterranean, Europe, SE Asia, Western Pacific) grouping, 1991–2022.

<u>HDI Country Group</u>

For risk classification 1, when separated by HDI country group, relative to a baseline of 1991 to 2000 (inclusive) the number of hours that exceeded the threshold for moderate, high, and extreme heat stress risk increased in the period between 2022 and 2013 (inclusive), as follows:

Low: by 127 (9% increase), 147 (17% increase), and 62 (28% increase) hours per person per year, respectively
Medium: by 232 (11% increase), 276 (18%), and 259 (48%) hours per person per year, respectively
High: by 185 (19% increase), 174 (28%), and 78 (55%) hours per person per year, respectively
Very High: by 141 (33% increase), 117 (45%), and 57 (141%) hours per person per year, respectively

For risk classification 3 (Fig.6), when separated by HDI country group, in 2022 to 2013 (inclusive) the number of hours exceeding the threshold for moderate, high, and extreme heat stress risk increased, relative to a baseline of 1991 to 2000 (inclusive), as follows:
Low: by 149 (9% increase), 136 (11% increase), and 117 (27% increase) hours per person per year, respectively
Medium: by 217 (9% increase), 253 (14%), and 284 (30%) hours per person per year, respectively
High: by 194 (18% increase), 179 (22%), and 128 (42%) hours per person per year, respectively
Very High: by 158 (32% increase), 126 (37%), and 90 (78%) hours per person per year, respectively

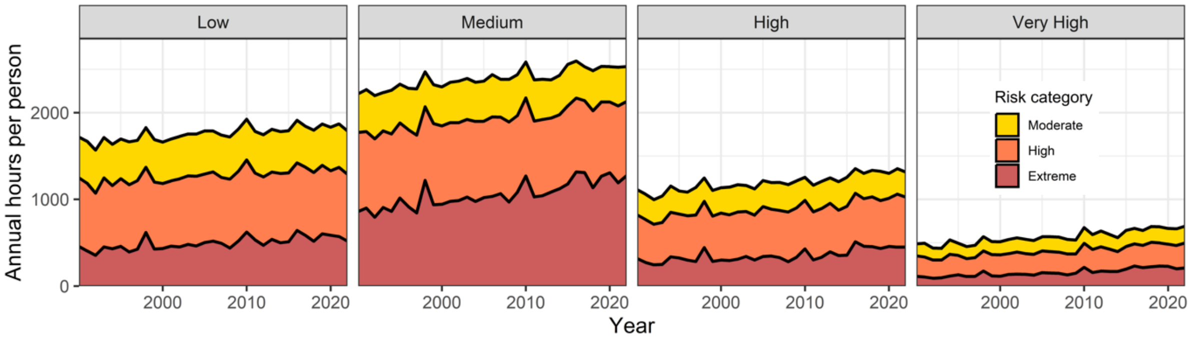

Figure 16: Average annual hours per person that moderate physical activity (SMA Risk Classification 3) entailed at least a moderate, high, or extreme heat stress risk by HDI country (Low, Medium, High, Very High) grouping, 1991-2022.

Indicator 1.1.4: Change in Labour Capacity

Work hours lost

Indicator authors

Chris Freyberg, Dr Bruno Lemke, Matthias Otto

Methods

The input data for this indicator have extended for the 2023 report and include future climate projections.

The indicator is based on 68,940 grid cell data (0.5 x 0.5 degrees with boundaries exactly on the degree and half degree co-ordinates) for climate and population. The focus is on trends since the end of the 20th century and on a method that can calculate labour capacity loss at country level. The model data chosen for the calculations were the European Centre for Medium-Range Weather Forecasts (ECMWF) ERA5 reanalysis hourly data on single year levels, and the analysis method is described in detail in the paper by Kjellstrom et al., 2018.[21]

Analysis starts from hourly ambient (t2m) and dew point temperatures (d2m), as well as short wave (solar) radiation downward (ssrd). These inputs are used to derive the hourly heat stress index Wet Bulb Globe Temperature (WBGT) and, from that, the work loss factor (WLF) at three different metabolic rates in both the shade and the sun is calculated. The inclusion of the solar component represents a novelty for the 2022 report.

The full Liljegren formula for calculating WBGT in the sun was used for one year (2010) for all grid cells. This involved also downloading ERA5 surface pressure, surface thermal radiation downwards, total sky direct solar radiation at surface. With this data, a good approximation for WBGT *uplift* in the sun was determined from WBGT in the shade. Tested in warm to hot Koppen climate regions, this uplift was 0.0035 * ssrd, which matched the Liljegren WBGT calculation to ±0.2 C. As the Liljegren WBGT calculation[15] also requires air speed, an air movement of 1 m/s was used as the approximate minimum apparent wind speed generated by the movement of arms and legs during work.

For indoor work, exposure was assumed to be atmospheric heat in the shade without effective air conditioning. The impact of heat on labour capacity depends on clothing (assuming light clothing for all) and metabolic rate based on physical work activity. The methodology considers 3 metabolic rates: 200W (light work, sitting or moving around slowly), 300W (medium intensity work) and 400W (heavy labour).

The function relating WLF (the fraction of work hours lost) to an hourly WBGT level is given by the cumulative normal distribution (ERF) function:

$$Loss\ fraction = \frac{1}{2}\left(1 + \mathrm{ERF}\left(\frac{\mathrm{WBGThourly} - \mathrm{WBGTaver}}{\mathrm{WBGT}_{\mathrm{SD}} * \sqrt{2}}\right)\right)$$

where WBGTaver and WBGT_{SD} are the parameters (Table 3) in the function for a given activity level.

The data were then aggregated to provide estimates of annual WLF between the hours of 6 am–6 pm local solar time for each grid-cell.

Table 3: Input values for labour loss fraction calculation.

Metabolic rate	WBGTaver	WBGT_{SD}
200 Watts	35.5	3.9
300 Watts	33.5	3.9
400 Watts	32.5	4.2

For each grid cell, the working age population (15+ years old; as in the ILOSTAT data) for each time period is used as input data as well as the percentages of people in this age range working in 4 sectors: agriculture, construction, manufacturing, and "other" sectors, which include the service sector (based on ILOSTAT data). Populations in grid cells that overlap country borders have been apportioned to the countries involved based on population distribution within the cell (variable CountryPop% in the formulas below).

For the work hours lost (WHL), ILO sector proportions are assigned to metabolic rates and sun, or indoors/shade calculations applied as shown in Table 4:

Table 4: Employment sector to metabolic rate assignment.

Metabolic rate:	200W (shade),<br>light work	300W (shade),<br>moderate work	400W (sun),<br>heavy labour
Employment sector:	Other<br>(mainly services)	Manufacturing	Agriculture +<br>Construction

The total annual work hours lost (WHL) for each metabolic rate and country (as well as a global aggregate) are calculated by, first, for each grid cell multiplying each employment sector population by the relevant work loss factor and then, second, summing the resulting sector work hours lost over all grid-cells in each country:

Annual WHL200W (per country) =
Σ(for each country grid-cell): Pop15plus * CountryPop% * Other% * WLF200W

Annual WHL300W (per country) =
Σ(for each country grid-cell): Pop15plus * CountryPop% * Manuf% * WLF300W

Annual WHL400W (per country) =
Σ(for each country grid-cell): Pop15plus * CountryPop% * (Agr% + Constr%) * WLF400W

Then: Total Annual WHL (country) = Annual WHL200W + Annual WHL300W + Annual WHL400W

The annual work hours lost per person (WHLpp) are arrived at by dividing the total annual country WHLs by the total number of employed people in each country for each year. The annual total number of employed people for each country is calculated like:

Annually Employed People (per country) =
(Agr% + Manuf% + Constr% + Other%) * Σ(for each country grid-cell): Pop15plus * CountryPop%

To estimate the effects of modelled climate projections on productivity loss, the input data for this indicator to the CVM 2022 report[22] were used. The CVM data contain work-loss factors (WLF, percentages) for each country for 4 time periods: 1995–2014 (the baseline), 2021–2040, 2041–2060 and 2081–2100, and for two socioeconomic and RCP pathways (SSP) 126 and 370. WLF calculated from five climate models (GFDL, IPSL, MPI, MRI, UKESM) have been averaged. In order to compare WLF projections based on the ISIMIP 3b dataset with WLF derived from ERA5 historical climate data, the projected work-loss figures have been scaled to the equivalent baseline time-period of the historical data. Specifically:

For each country and metabolic rate (200, 300, 400W and 400Wsun):

WLFbl_hist = average (WLFhist1995…WLFhist2014) [baseline, historical WLF]

For each post-baseline time period and SSP in the projections:

WLFp = WLFbl_hist + WLFcvm – WLFbl_cvm [scaled projected WLF]
where WLFcvm is WLF used in CVM, WLFbl_cvm WLF from CVM baseline (1995–2014)

For the whole world, country WLFp were population-weighted to produce global WLFp (for each post-baseline time period, SSP and metabolic rate). The populations used are each country's working age population (age 15-64) which is relevant for weighting work-loss.

Results of the analysis combining the historical work capacity losses with future projections are shown in **Figure 19: Historical and projected work capacity loss under two Socioeconomic Pathways (126 and 370). All working conditions in the shade unless indicated otherwise.Figure 19**.

Data

1. Climate data from the European Centre for Medium-Range Weather Forecasts (ECMWF) ERA5 reanalysis.[23]

2. Population data from the NASA Socioeconomic Data and Applications Center (SEDAC) Gridded Population of the World (GPWv4).[4]
3. Sector employment data from ILOSTAT.[24]
4. Future climate projections from ISIMIP 3b protocol.[11]

Caveats

The distribution of agricultural, construction, manufacturing and other sector workers is only reported at country level, hence this proportion is distributed evenly to all grid cells within each country, and thus does not capture the geographical differences in the proportion of people working in the different sectors.

Analysis performed with the above-described methodology has shown that the ERA5 data regularly understate maximum air temperatures. The ERA5 deviation from the ensemble average of several other data sources varies by location, is generally in the order of 1–4°C lower and is especially pronounced in coastal regions. Combined with often high population concentrations near the coast, the WHL results presented here are conservative. As a comparison, when applying the WHL calculations to climate data input sourced from ISIMIP or weather stations, WHL estimates increase by 40%. Differences between disparate climate datasets have been compensated for when combining projections with historical data for this indicator (see tail-end in the Methods section).

Future form of the indicator

Improved methods are currently under development for estimating labour capacity loss from climate and demographic data. Future versions of the indicator may employ these improvements after they have been tested and validated. There may be alternative proxies for productivity loss other than the number of work hours lost. For example, the team is exploring the concept of *Safe Work Hours*.

Additional analysis

Across the globe, in 2022, 490 billion work hours may have been lost due to heat, equivalent to an average of 143 work hours per employed person (Table 5). Twelve countries, the three most populous at each HDI (Human Development Index) level, account for >70% of the total global work hours lost. The three worst affected countries are on the low and medium HDI level and rank highest in the world's work hours lost per employee, 2.5 times the world average. The three biggest countries in the highest HDI category account for the smallest numbers of employment hours lost.

Table 5: Annual heat-related work hours lost per employed person (agriculture & construction exposed to the sun, all other sectors in shade or indoors) and total WHL in populous countries. Three countries with largest populations in each of the four HDI categories are ranked by WHLpp in 2022.

	**ISO3 code**	**Human development level**	**Work hours lost per employed person 1991–2000**	**Work hours lost per employed person in 2022**	**Billions of work hours lost in 2022**	**% of global**
Global			139.2	143.4	490.7	
Pakistan	PAK	Low	338.3	355.2	26.4	5.4%
India	IND	Medium	341.6	353.7	190.6	38.9%
Bangladesh	BGD	Medium	392.6	349.9	25.5	5.2%
Democratic Republic of the Congo	COD	Low	198.9	217.7	7.7	1.6%
Nigeria	NGA	Low	268.2	213.4	15.9	3.2%
Indonesia	IDN	High	236.1	196.5	25.3	5.2%
China	CHN	High	84.8	68.5	50.6	10.3%
Brazil	BRA	High	87.0	53.7	5.2	1.1%
Ethiopia*	ETH	Low	39.6	35.3	1.8	0.4%
Japan	JPN	Very High	20.4	26.7	1.7	0.3%
United States of America	USA	Very High	14.2	17.3	2.9	0.6%

Russian Federation	RUS	Very High	2.3	2.0	0.1	0.0%
Rest of the world			103.1	104.1	137.0	27.9%

** The low impact per employee is linked to the high altitude (with cooler climate) of most of this country*

Occupational heat exposure leads to another aspect of social and health inequity: it is the difference between the impacts on the people in labouring jobs that require high physical intensity and those in office or service jobs with less straining work. The impact of heat exposure on labour capacity increases significantly with the physical intensity of the work.

Agricultural workers are the worst affected in many countries, with the burden often shifting to those in construction in higher income countries, such as the USA. Employment hours lost per worker are lower than the baseline in several countries. While weather variability year on year can be expected, and 2022 data cannot be taken as an estimate of trends, the reduction in the agricultural workforce, mainly in favour of the service industry, is the main driver of long-term static or negative work loss trends in a number of countries.

The global distribution of work hours lost (WHL) in the four workforce sectors is shown in Figure 17. Agriculture dominates but stays largely constant due to reductions of the agricultural workforce in many low- and middle-income countries. The impact of rising heat is increasing the fastest in construction and *other* sectors (mainly in the service industry).

Because of its definition, this indicator is influenced by the changes in population numbers and the distribution of the workforce within countries as well as climate change. WLF (work loss factor) is defined as the fraction of work hours lost for one worker at a specific metabolic rate, and thus describes work capacity loss due to heat independently from population and employment statistics. Figure 18 shows global WLF trends attributable to climate alone. In addition, this chart includes loss factor trends when an agricultural worker (400W metabolic rate) is exposed to the sun. The WLF rate doubling when solar radiation is included is an illustration of how disproportionate an increase by a few degrees of WBGT (solar *uplift* outlined above, typically between 1 and 2.5 degrees) affects human work capacity.

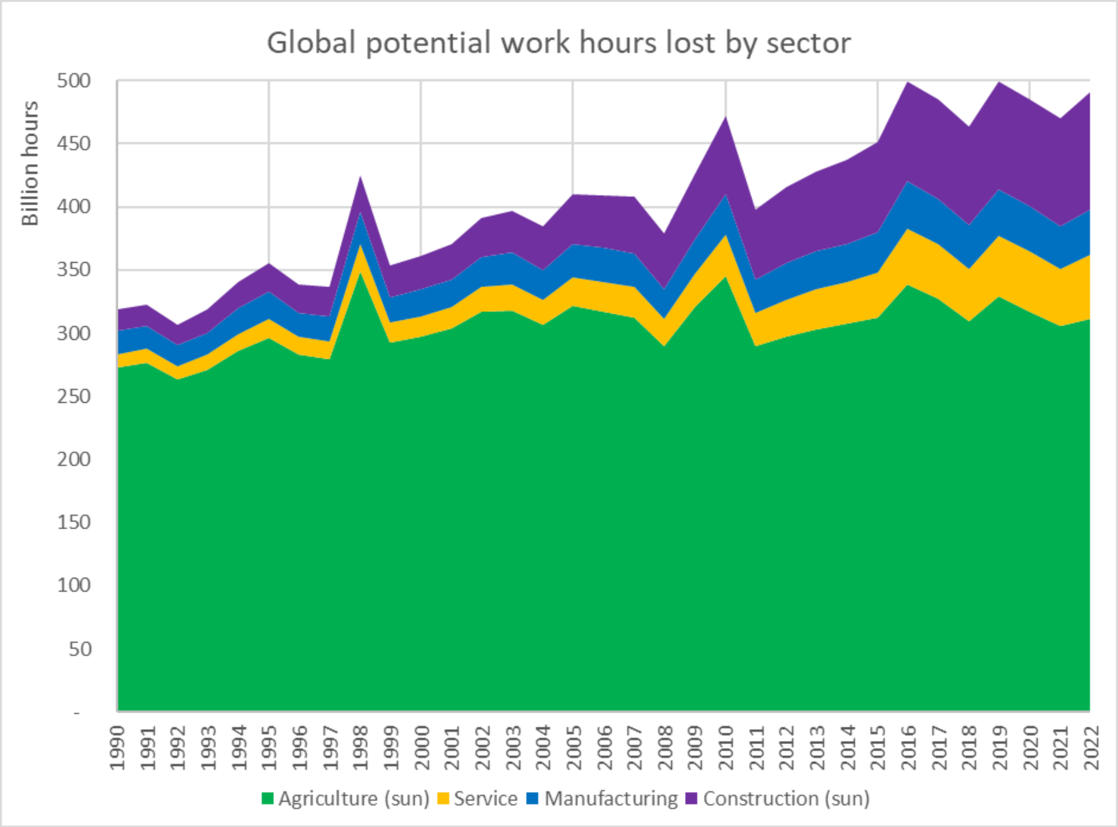

Figure 17. Global potential work hours lost (billions) due to heat by employment sector, 1990–2022.

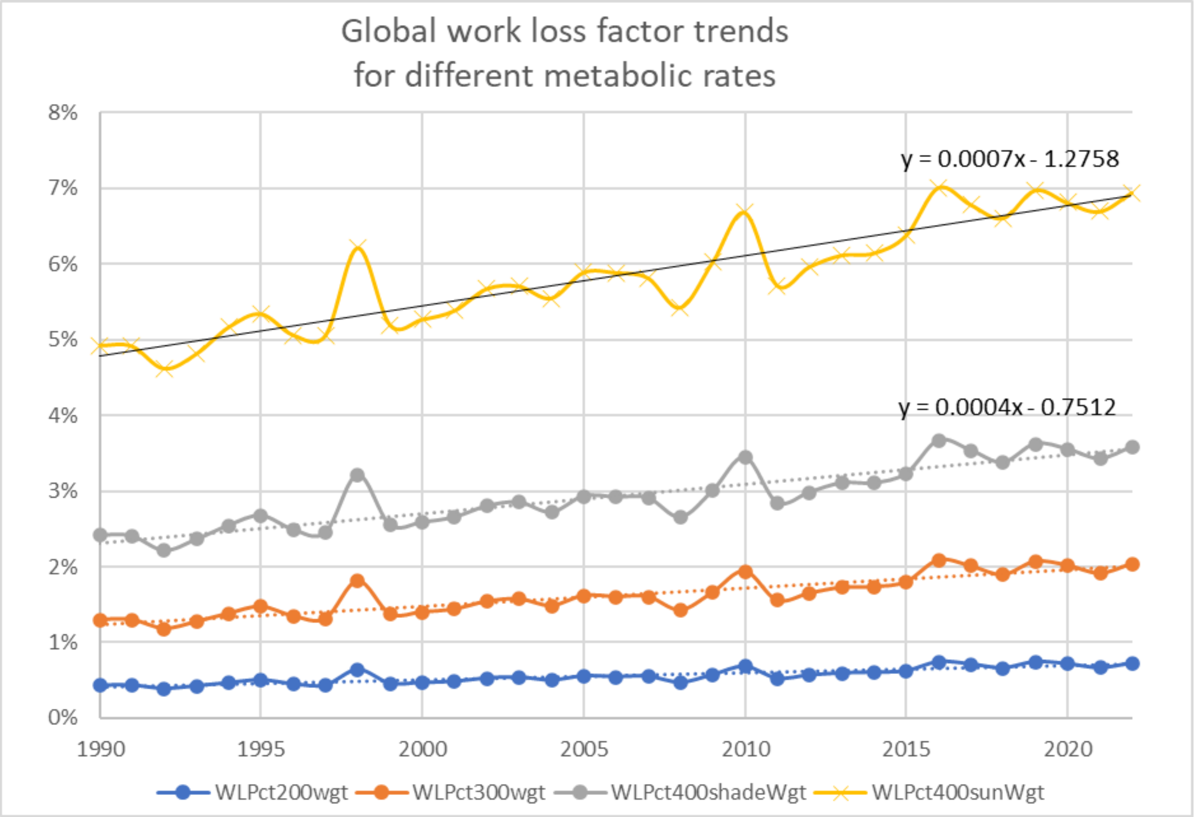

Figure 18. Work hours lost (% of annual hours) depending on physical work intensity, global means, 1990–2022.

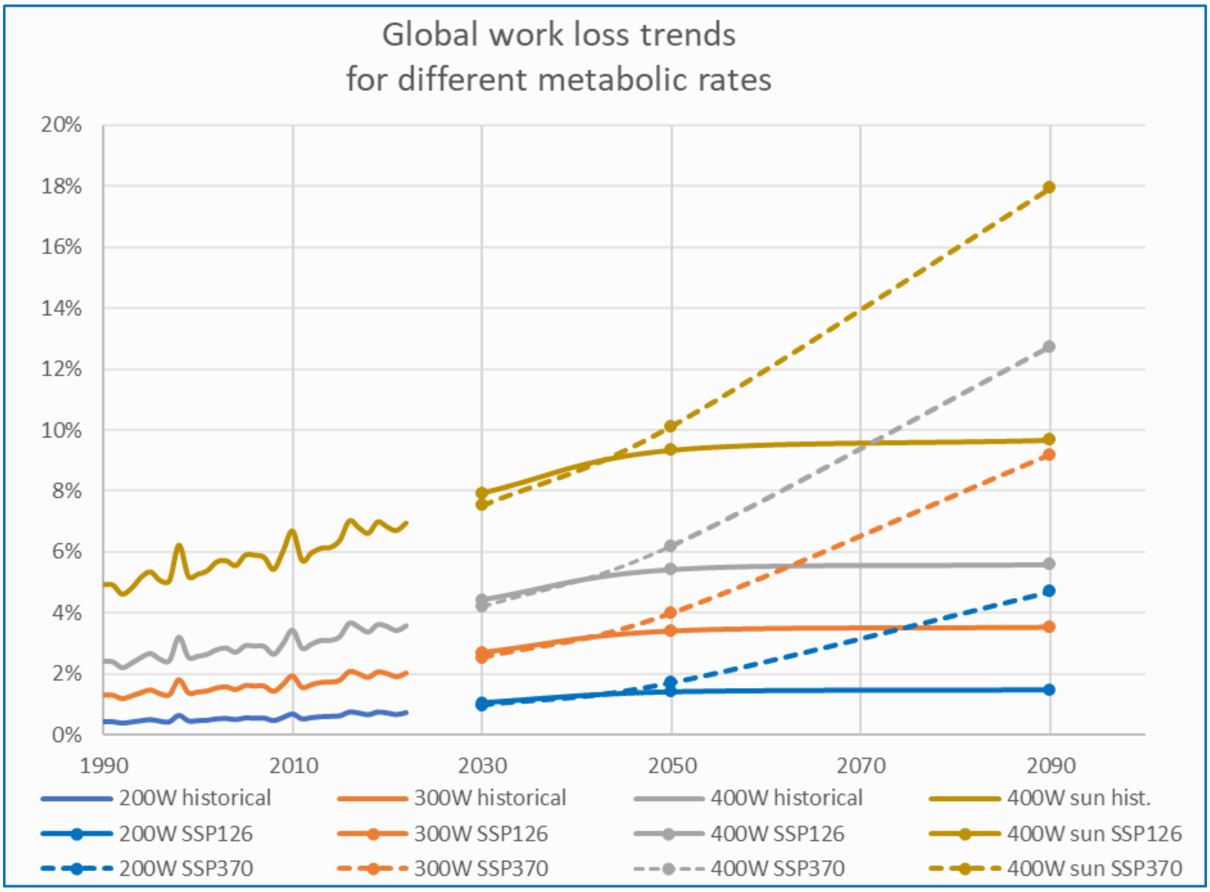

Figure 19: Historical and projected work capacity loss under two Socioeconomic Pathways (126 and 370). All working conditions in the shade unless indicated otherwise.

Numbers of outdoor workers

Indicator authors

Dr Natalie C. Momen, Dr Frank Pega

Methods

The World Health Organization (WHO) and the International Labour Organization (ILO) produce the WHO/ILO Joint Estimates of the Work-related Burden of Disease and Injury (WHO/ILO Joint Estimates).[25–28] These are the United Nations' official occupational burden of disease estimates, and the first such interagency estimates of their Specialized Agencies responsible for health and labour. The estimates are produced at the levels of the world, WHO region and country/area, and disaggregated by sex and age group, enabling health inequalities analysis between and within countries.[29]

A new set of WHO/ILO Joint Estimates has been produced of the proportion of the population who are occupationally exposed to solar ultraviolet radiation (or sunlight) for 195 countries/areas and the years 2000, 2010 and 2019.[30] WHO and the ILO, supported by the WHO Technical Advisory Group on Occupational Burden of Disease Estimation,[31] have estimated this exposure by proxy of occupation with outdoor work.[30] A job-exposure matrix was produced by occupational epidemiologists at WHO and labour statisticians and occupational hygienists at ILO, systematically classifying the 436 occupation unit group codes of the International Standard Classification of Occupations 2008 (ISCO-08)[32] at the four-digit level into occupations with outdoor work (assigned to the exposure category of "any (or high) occupational exposure to solar ultraviolet radiation", or outdoor worker) versus those without outdoor work (assigned to "no (or low) occupational exposure to solar ultraviolet radiation", or indoor worker). ISCO-08 codes were cross-walked to the International Standard Classification of Occupations 1988 codes,[33] using the official, standard cross-walking tables provided in the

ISCO-08 handbook. This matrix of occupations classified as outdoor workers provides the basis for assignment to occupational exposure to solar ultraviolet radiation (see annex 1 in Pega et al 2023[30]).

The input data for these WHO/ILO Joint Estimates were 166 million observations from 763 surveys for 96 countries/areas collected between 01 January 1996 and 31 December 2021 (for a list of the included surveys by country/area and year see annex 1 in Pega et al 2023[30]). All these surveys were official labour force surveys conducted by national or area-level statistical offices. Data from at least one survey are available for 49.2% of all countries/areas globally (Table 6). One-third (35.9%) of the global population of working age (≥15 years) in 2019 are covered with data from at least one survey. In each WHO region, at least 38.0% of countries/areas are covered by one or more surveys (Table 6). At least 30.7% of regional populations are covered, with the only exception being that data are available for just 6.0% of the Western Pacific population.

Table 6: Coverage of surveys and countries/areas in the WHO/ILO Joint Estimates of occupational exposure to solar ultraviolet radiation. Sourced from Pega et al 2023[30]. The programmatic allocation of the countries/areas to the WHO regions followed the WHO Coronavirus (COVID-19) Dashboard.[34]

	**WHO region**						**World**
	**African Region**	**Region of the Americas**	**Eastern Mediter-ranean Region**	**European Region**	**South-East Asia Region**	**Western Pacific Region**	
Number of countries/areas	47	35	22	53	11	27	195
Surveys (N)	69	168	41	391	49	45	763
Countries/areas with ≥1 survey (N) (% of countries)	18 (38.0%)	15 (42.9%)	9 (40.9%)	33 (62.3%)	8 (72.7%)	13 (48.1%)	96 (49.2%)

The proportion of the working-age population with prevalent occupational exposure to solar ultraviolet radiation was estimated,[30] using standard multilevel models,[35–37] applied in previous WHO[27,35–37] and WHO/ILO[27] estimations. This established statistical method is used by WHO to produce Sustainable Development Goal indicators (e.g., Indicator 3.9.1 "Mortality rate attributed to household and ambient air pollution")[38] and has therefore passed the approval of the United Nations Statistical Commission.

Based on these WHO/ILO Joint Estimates of the proportion of the population occupationally exposed to solar ultraviolet radiation for the years 2000, 2010, and 2019 (point prevalence sourced from annex 1 in Pega et al. 2023),[30] the percentage of the working-age population who were outdoor workers was estimated for the year 2022, assuming a linear trend. These outdoor worker estimates were produced for the world, the six WHO regions and seven *Lancet* Countdown regions, and 195 countries/areas (Table 7). They were also produced disaggregated by sex (three categories: females and males; females; males) and age group (≥15 years, and 17 categories of five-year age groups: 15–19, 20–24, ..., 90–94, ≥95 years). To estimate the total number of this exposed worker population, for each cohort defined by country (or region or global), sex and age group, its percentage of outdoor workers was multiplied with its total population for the year 2022. These total population numbers were sourced from the official projections of the United Nations Population Division.[39] Indicators for occupational exposure to climatic risk factors that are disaggregated by socioeconomic characteristics are a priority for workers' health data and monitoring at the global, regional and national levels, because they enable tracking of the social and climatic determinants of health and health equity among populations of workers.[40]

Table 7: Countries/areas covered by the indicator by WHO region. The programmatic allocation of the countries/areas to the WHO regions followed the WHO Coronavirus (COVID-19) Dashboard.[34]

WHO region (Number of countries/areas)	Country or area
African Region (47)	Algeria; Angola; Benin; Botswana; Burkina Faso; Burundi; Cabo Verde; Cameroon; Central African Republic; Chad; Comoros; Congo; Côte d'Ivoire; Democratic Republic of the Congo; Equatorial Guinea; Eritrea; Eswatini; Ethiopia; Gabon; Gambia; Ghana; Guinea; Guinea-Bissau; Kenya; Lesotho; Liberia; Madagascar; Malawi; Mali;

	Mauritania; Mauritius; Mozambique; Namibia; Niger; Nigeria; Rwanda; Sao Tome and Principe; Senegal; Seychelles; Sierra Leone; South Africa; South Sudan; Togo; Uganda; United Republic of Tanzania; Zambia; and Zimbabwe
Region of the Americas (35)	Antigua and Barbuda; Argentina; Bahamas; Barbados; Belize; Bolivia (Plurinational State of); Brazil; Canada; Chile; Colombia; Costa Rica; Cuba; Dominica; Dominican Republic; Ecuador; El Salvador; Grenada; Guatemala; Guyana; Haiti; Honduras; Jamaica; Mexico; Nicaragua; Panama; Paraguay; Peru; Saint Kitts and Nevis; Saint Lucia; Saint Vincent and the Grenadines; Suriname; Trinidad and Tobago; United States of America; Uruguay; and Venezuela (Bolivarian Republic of)
Eastern Mediterranean Region (22)	Afghanistan; Bahrain; Djibouti; Egypt; Iran (Islamic Republic of); Iraq; Jordan; Kuwait; Lebanon; Libya; Morocco; occupied Palestinian territory, including east Jerusalem; Oman; Pakistan; Qatar; Saudi Arabia; Somalia; Sudan; Syrian Arab Republic; Tunisia; United Arab Emirates; and Yemen
European Region (53)	Albania; Andorra; Armenia; Austria; Azerbaijan; Belarus; Belgium; Bosnia and Herzegovina; Bulgaria; Croatia; Cyprus; Czechia; Denmark; Estonia; Finland; France; Georgia; Germany; Greece; Hungary; Iceland; Ireland; Israel; Italy; Kazakhstan; Kyrgyzstan; Latvia; Lithuania; Luxembourg; Malta; Monaco; Montenegro; Netherlands; North Macedonia; Norway; Poland; Portugal; Republic of Moldova; Romania; Russian Federation; San Marino; Serbia; Slovakia; Slovenia; Spain; Sweden; Switzerland; Tajikistan; Türkiye; Turkmenistan; Ukraine; United Kingdom; and Uzbekistan
South-East Asia Region (11)	Bangladesh; Bhutan; Democratic People's Republic of Korea; India; Indonesia; Maldives; Myanmar; Nepal; Sri Lanka; Thailand; and Timor-Leste
Western Pacific Region (27)	Australia; Brunei Darussalam; Cambodia; China; Cook Islands; Fiji; Japan; Kiribati; Lao People's Democratic Republic; Malaysia; Marshall Islands; Micronesia (Federated States of); Mongolia; Nauru; New Zealand; Niue; Palau; Papua New Guinea; Philippines; Republic of Korea; Samoa; Singapore; Solomon Islands; Tonga; Tuvalu; Vanuatu; and Viet Nam

Data

1. WHO/ILO Joint Estimates of the population exposed to solar ultraviolet radiation at the workplace, sourced from Pega et al 2023.[30]
2. United Nations projections under the medium scenario of the total number of the population, sourced from the 2022 Revision of the World Population Prospects.[39]

Caveats

The estimates of the number and percentage of outdoor workers were produced based on the assumption of a linear trend in these variables over time. This is a common assumption made across many exposure estimations, including by WHO and ILO for estimating diverse occupational risk factor exposures.[27,30]

The estimates cover workers in the informal economy for regions, countries and areas, where national or area-level statistical offices have collected occupation data on these workers in the Labour Force Surveys that were used as input data for the WHO/ILO Joint Estimates of the proportion of the population occupationally exposed to solar ultraviolet radiation. Unpaid work is not captured.

The coronavirus (COVID-19) pandemic has changed and continues to change occupations and their work tasks, including those performed outdoors. The indicator was produced using data collected in labour force surveys up until and including the year 2021. While these data may have captured some of the labour force changes that may have occurred for occupations regarding outdoor work, several labour force surveys may have been interrupted in collection or processing during the pandemic (e.g., during lockdowns). Future estimations will show if changes in the number or the percentage of outdoor workers occurred over the short or longer term.

Future form of the indicator

The indicator could be further developed to capture the number and percentage of outdoor workers who are occupationally exposed to specific climatic risk factors (e.g., heat) above a threshold defined by one or both of exposure intensity and exposure period (e.g., occupational exposure limit). This should result in an indicator like the existing Lancet Countdown Indicator 1.1.1 "Exposure to warming" but singling out the particularly vulnerable outdoor workers. Additionally, the indicator could also be further advanced through the geocoding of the outdoor worker numbers and percentages at subnational levels, such as local authority or city. Further disaggregation could also be added, including rural-urban area, socio-economic status, and migrant status.

Additional analysis

Globally in 2022, an estimated total of 1.6 billion people of working age were outdoor workers. In relative terms, approximately 26.4% of working-age people worked outdoors. However, there are several differences between WHO regions, countries, sexes, and age groups in both the number and percentage of outdoor workers.

By WHO region, in absolute terms, the largest number of outdoor workers of working age resided in the South-East Asia Region (0.5 billion) and the Western Pacific Region (0.4 billion) (Figure 20). In relative terms however, the WHO regions with the largest percentage of the population working outdoors were the African Region (32.1%) and the South-East Asia Region (29.8%) (Figure 20). The number and proportion of outdoor workers by country/area are presented in Figure 21 and Figure 22, respectively.

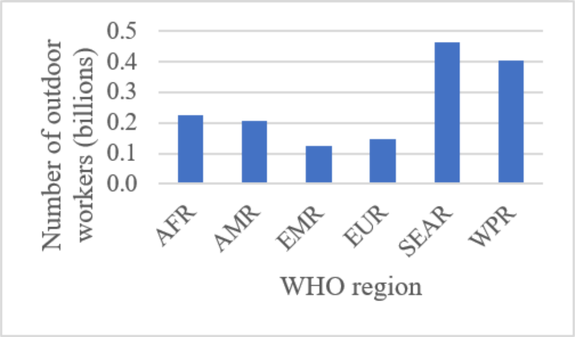

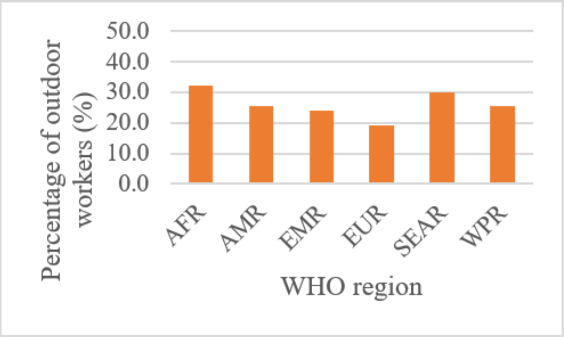

Figure 20: Number and percentage of outdoor workers by WHO region, population of working age (≥15 years), 195 countries/areas, 2022 (WHO estimates). Legend: AFR African Region; AMR Region of the Americas; EMR Eastern Mediterranean Region; EUR European Region; SEAR South-East Asia Region; WPR Western Pacific Region.

Figure 21: Number of outdoor workers (in billions), population of working age (≥15 years), 195 countries/areas, 2022 (WHO estimates).

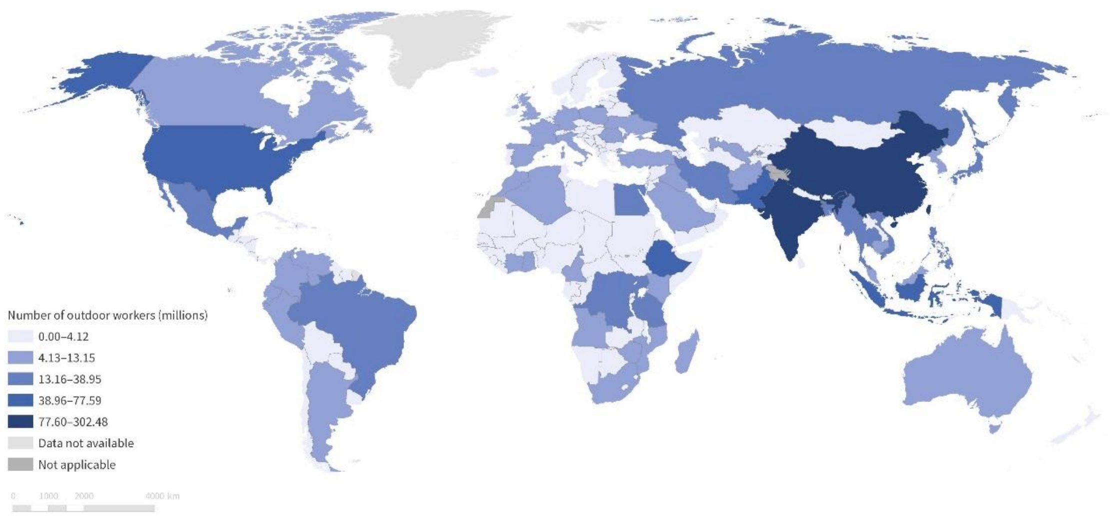

Figure 22: Percentage of outdoor workers, population of working age (≥15 years), 195 countries/areas, 2022 (WHO estimates).

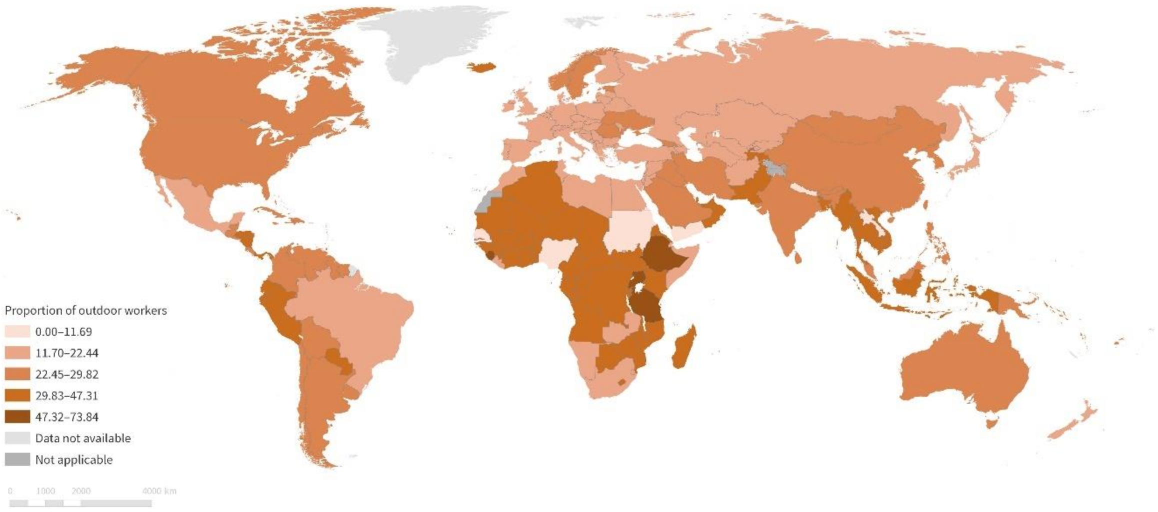

There are large differences in the outdoor workers indicator by sex. Globally in 2022, among every four outdoor workers, three are estimated to be males (1.1 billion), and one is estimated to be female (0.4 billion). Similarly, 38.4% of all working-age males globally are outdoor workers, compared with only 14.6% of the working-age population of females. There are absolute and relative differences between WHO regions in their numbers and percentages of females compared with males who are outdoor workers (Figure 23, Figure 24). For the number of outdoor workers, the South-East Asia and the Western Pacific Regions have the largest absolute differences between females and males, whereas the Eastern Mediterranean Region and the Region of the Americas have the largest relative differences by sex (Figure 23). For the percentage of outdoor workers, the Eastern Mediterranean Region and the Region of the Americas have the largest absolute and relative sex differences (Figure 24). Of note, relative between-sexes differences in the number and proportion of outdoor workers are by far lowest within the African Region. This more equal distribution by sex is driven by the higher percentage of females working outdoors in this WHO region, compared with in the other WHO regions, rather than relatively lower percentages of male outdoor workers in this region, meaning both sexes are equally exposed at relatively high levels (Figure 23, Figure 24). However, males consistently have substantially larger numbers and percentages of outdoor workers than females in all regions.

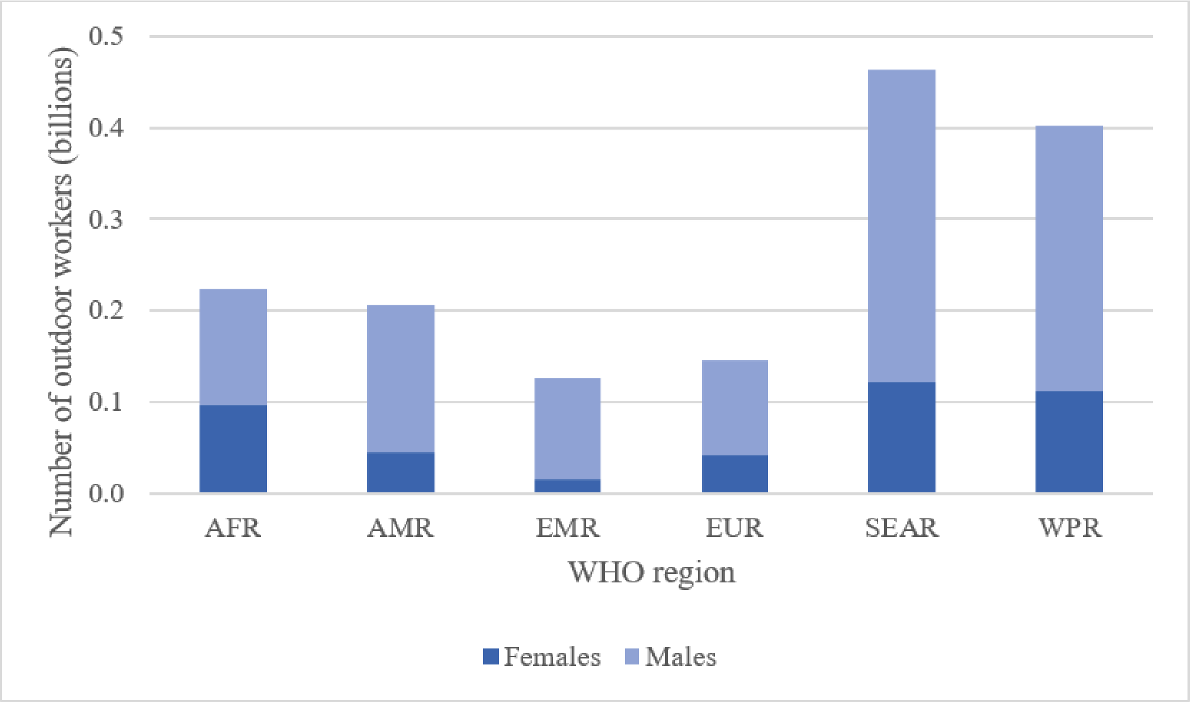

Figure 23: Number of outdoor workers by WHO region and by sex, population of working age (≥15 years), 195 countries/areas, 2022 (WHO estimates). Legend: AFR African Region; AMR Region of the Americas; EMR Eastern Mediterranean Region; EUR European Region; SEAR South-East Asia Region; WPR Western Pacific Region.

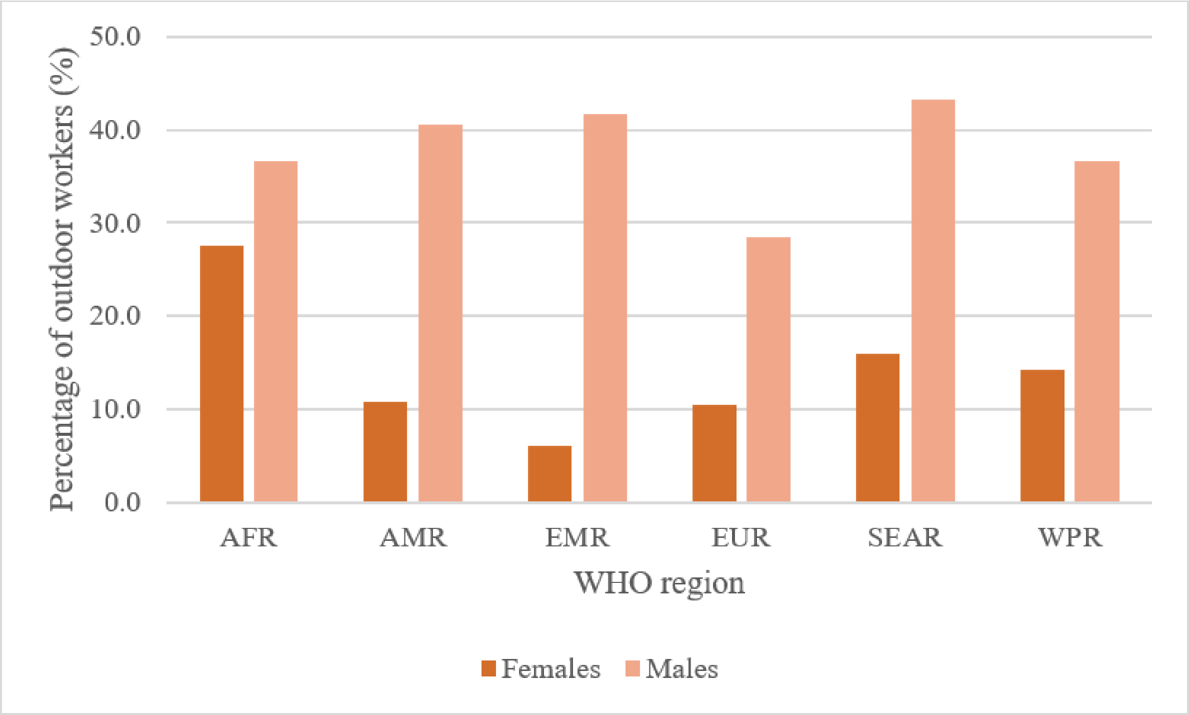

Figure 24: Percentage of outdoor workers by WHO region and by sex, working-age population (≥15 years), 195 countries/areas, 2022 (WHO estimates). Legend: AFR African Region; AMR Region of the Americas;

EMR Eastern Mediterranean Region; EUR European Region; SEAR South-East Asia Region; WPR Western Pacific Region.

There are also substantial age group differences in outdoor worker numbers and percentages. Both globally and within all WHO regions, age groups in early and middle adulthood comprise the largest numbers and percentages of outdoor workers (Figure 25, Figure 26).

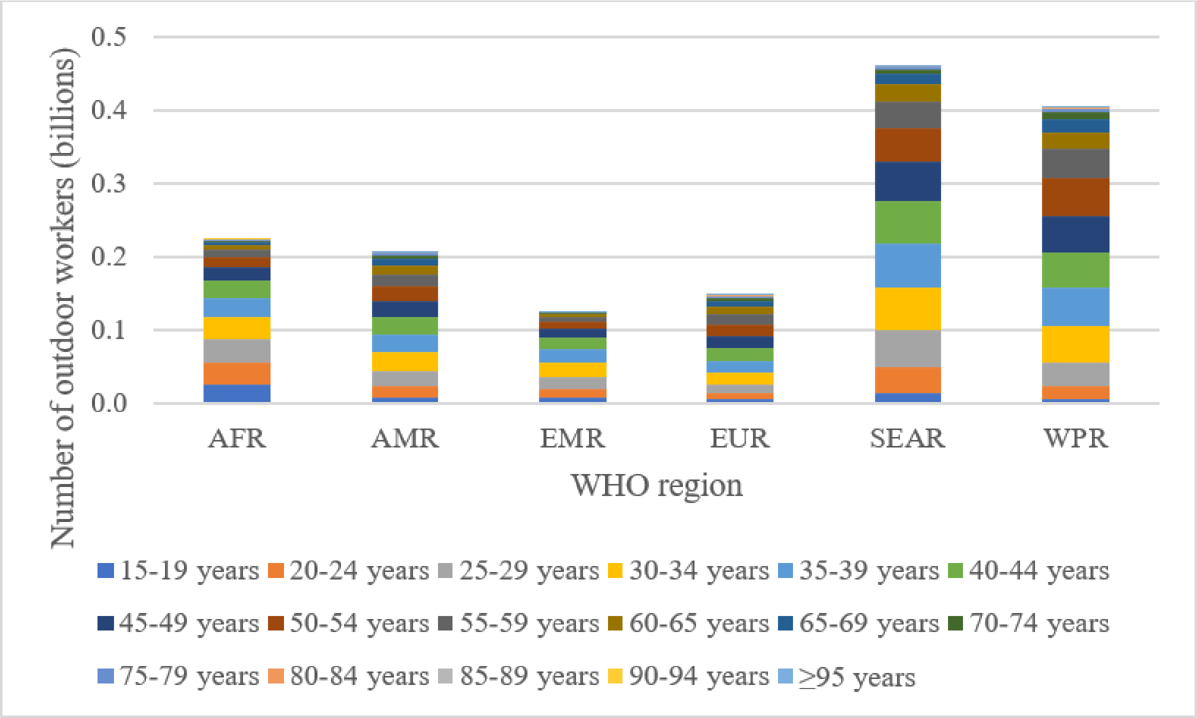

Figure 25: Number of outdoor workers by WHO region and by age group, working-age population (≥15 years), 195 countries/areas, 2022 (WHO estimates). Legend: AFR African Region; AMR Region of the Americas; EMR Eastern Mediterranean Region; EUR European Region; SEAR South-East Asia Region; WPR Western Pacific Region.

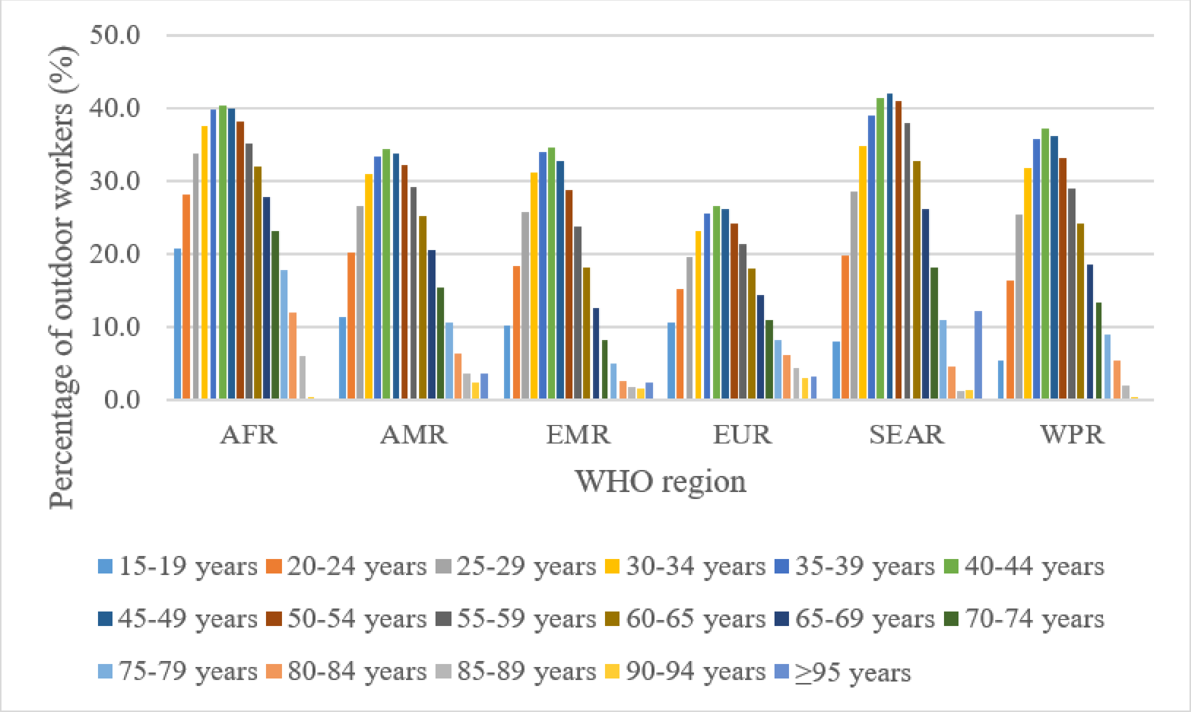

Figure 26: Percentage of outdoor workers by WHO region and by age group, working-age population (≥15 years), 195 countries/area, 2022 (WHO estimates). Legend: AFR African Region; AMR Region of the Americas; EMR Eastern Mediterranean Region; EUR European Region; SEAR South-East Asia Region; WPR Western Pacific Region.

Globally between the years 2000 and 2022, there were reductions in both the number of outdoor workers (-0.2 billion) and the percentage of working-age people who worked outdoors (-15.3 percentage points).

Change in the absolute number of outdoor differed by WHO region. The global reduction was primarily driven by the reduction observed in the Western Pacific Region (-0.3 billion) (Figure 27). The change in the number of outdoor workers by country/area is presented in Figure 28. The reduction in the number of outdoor workers in the People's Republic of China (-0.3 billion) strongly drove the reductions globally and in the Western Pacific Region (Figure 28). The absolute number of outdoor workers increased in the Eastern Mediterranean Region by 0.1 billion people and in the European Region, Region of the Americas, and African Region by <0.1 billion people.

The change in the percentage of outdoor workers from the year 2000 to the year 2022 also differed between WHO regions. The largest reductions in the percentage points of people who worked outdoors were estimated for the Western Pacific Region by 31.8 percentage points, followed by the African Region by 23.9 percentage points (Figure 27). These regional trends were driven by the two most populous countries in these regions (Figure 27). The People's Republic of China had an estimated 32.4 percentage point reduction in the proportion of people who were outdoor workers. Nigeria had the largest estimated reduction over the same period: -90.8 percentage points. The only regional increases were estimated for the European Region (4.0 percentage points) and the Eastern Mediterranean Region (0.4 percentage points) (Figure 27). Estimated changes in the percentages of outdoor workers varied substantively at the national level, with reductions and increases of differing sizes estimated for different countries (

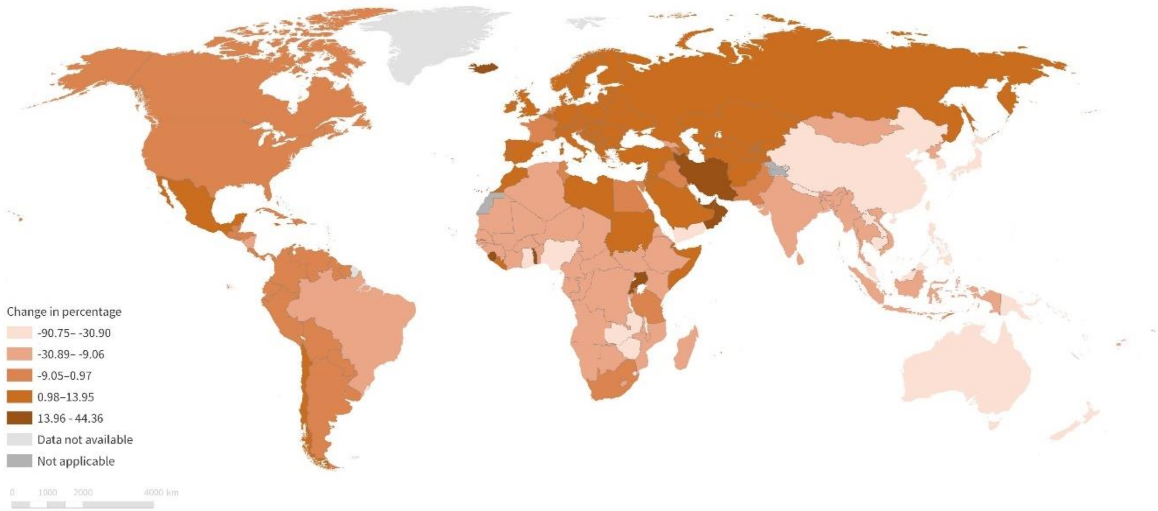

Figure 29).

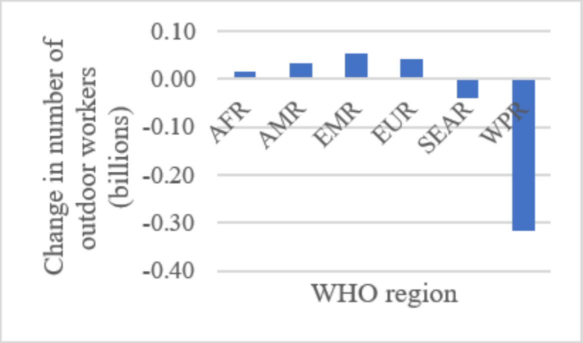

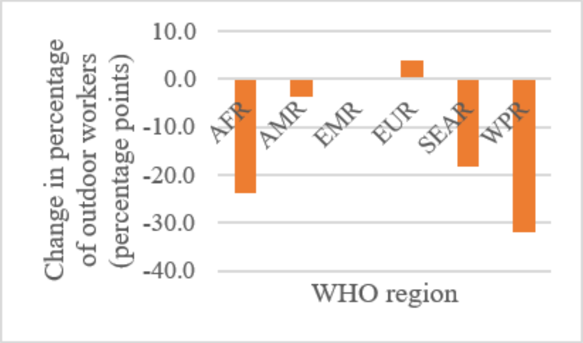

Figure 27: Change in the number and percentage of outdoor workers between the years 2000 and 2022 by WHO region, working-age population (≥15 years), 195 countries/area (WHO estimates). Legend: AFR African Region; AMR Region of the Americas; EMR Eastern Mediterranean Region; EUR European Region; SEAR South-East Asia Region; WPR Western Pacific Region.

Figure 28: Change in the number of outdoor workers between the years 2000 and 2022, working-age population (≥15 years), 195 countries/area (WHO estimates)

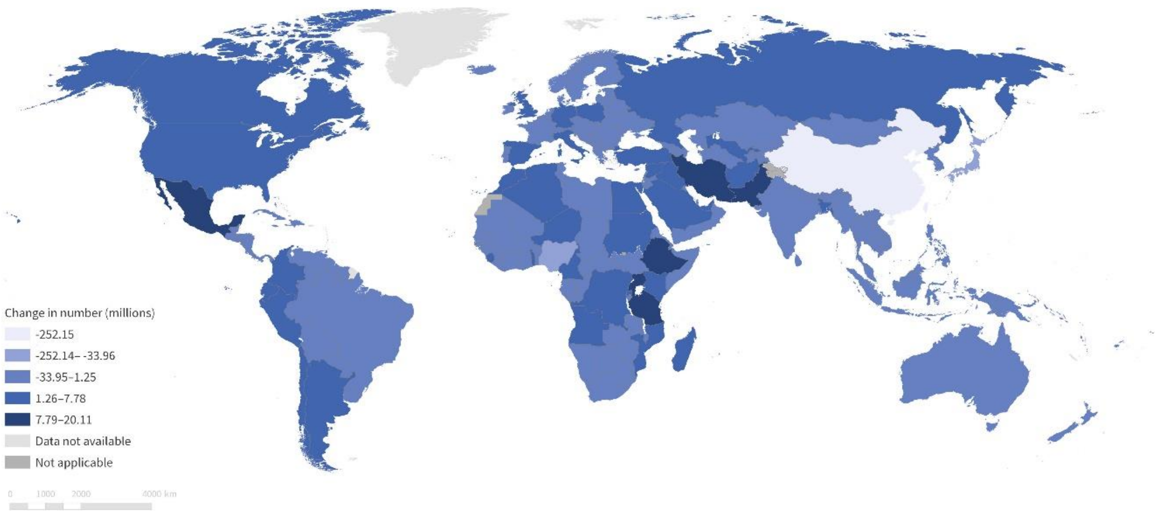

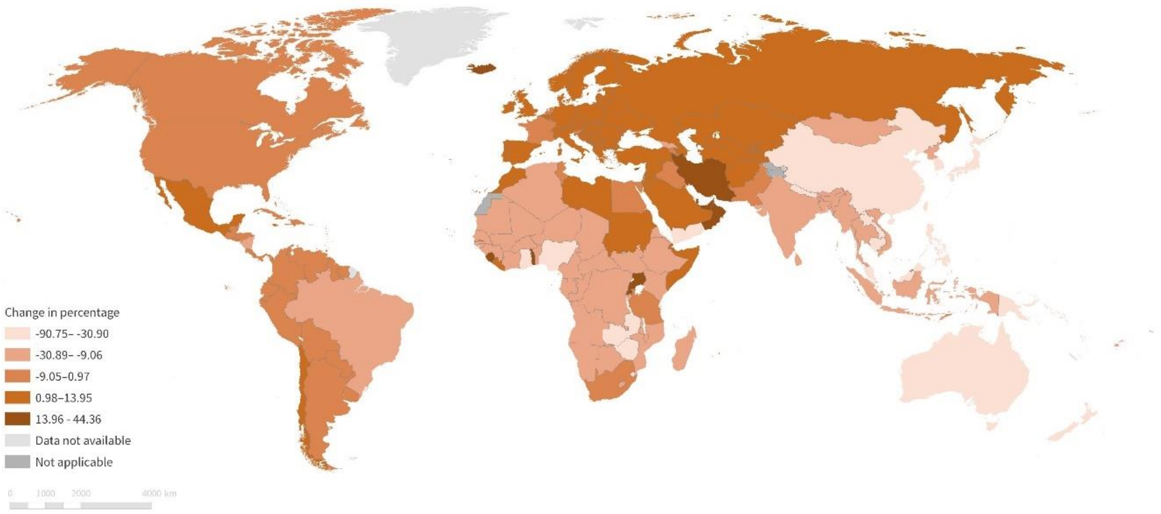

Figure 29: Change in the percentage of outdoor workers between the years 2000 and 2022, working-age population (≥15 years), 195 countries/area (WHO estimates)

Indicator 1.1.5: Heat-Related Mortality

Attribution of human exposure to health-threatening temperatures

Indicator authors

Dr Andrew Pershing

Methods

The median, quantile, and model-based methods defined[41] were used to develop a multi-method estimate of the likelihood of temperatures in the modern and counterfactual climates. For each approach, the year was divided into 24 overlapping periods of 31 days. The modern climate was defined based on the global mean temperature (GMT) from the sample year (HadCRUT 5 relative to 1850–1899). The counterfactual climate had GMT=0°.

Median method: The median method assumed climate change only affects the median, not the variance or shape of distribution of daily temperatures. For each period, the frequency of daily temperatures (T) were approximated by fitting a skew normal distribution ($SN_{ref}(T)$) to the data from the reference period 1991-2020. Linear regression was used on data from 1950-2020 to calculate β=the local climate sensitivity (change in median local temperature for a 1° change in GMT) The likelihood of T in a climate defined by GMT was then $SN_{ref}(T-\beta\,(GMT-GMT_{ref}))$ where GMT_{ref} is the GMT of the reference period.

Quantile method: The quantile method allowed the shape of the distribution to change. For each period, the local climate sensitivity (β_q) was calculated for 21 evenly spaced quantiles q between 0·01 and 0·99. To create the distribution for GMT, the quantile distribution was shifted from the reference period by $\beta_q\,(GMT-GMT_{ref})$. A least-squares procedure was used to find the skew normal parameters that approximate the shifted distribution.

Model-based method: Twenty-four models from CMIP6 with forced (historical and either SSP3-7.0 or SSP5-8.5) and control runs were used. The models were debiased using[42] relative to the ERA5 data from the reference period. For each model, the year when its global mean temperature first exceeded GMT was determined. A skewed normal distribution was fit for each period using the 31 years of data surrounding this year. This allowed computation of the modern likelihood. The counterfactual likelihood was computed from skew normal distribution fit to the control runs.

Synthesis: Each method computation of $C_{method}(T)=\log_2(L_{modern}(T)/L_{counter}(T))$, where $L_{climate}$ is the likelihood of T in the specific climate using the prescribed method. All estimates were combined into a single value:

$$C_{combined}(T) = [(C_{median} + C_{quantile})/2 + \sum_{model} C_{model}/N]/2$$

where N is the number of models (usually 24). The cutoffs for attributable mortality days were $C_{combined}(T) = 1$ (implying a ratio of 2) for warm events, and $C_{combined}(T) = -1$ for cool events. Note there were occasions where $C_{method}(T)$ could not be computed. Median and quantile methods and 50% of the models were required to return a value to use the data at that location and time.

Data

Daily average temperatures from ERA5 3 were used to quantify location-specific minimum mortality temperatures (MMT), defined as the 83rd percentile temperature of all daily temperatures at a location over the period 1986–2005.

The number of days in each year from 1997–2022 that were above this threshold were counted. The likelihoods of encountering each daily temperature in the climate of the corresponding year (termed the modern climate) and the likelihoods in a counterfactual climate with no anthropogenic warming were estimated using a multi-method approach[41] that historical conditions from ERA5 and 24 climate models from CMIP6 1 .

Days where the temperature exceeded MMT and where the ratio of the modern to counterfactual likelihoods was two or higher were categorized (and counted) as attributable mortality days. An analogous process was used for temperatures colder than the 17th percentile. The likelihood ratio of less than 0·5 was used as evidence that climate change is making these temperatures less likely. Indices of human exposure were calculated using the population-weighted average of the days above MMT and the attributable mortality days. This was done globally and for individual countries. The averages from individual countries were aggregated to geographic and socio- economic regions.

Caveats

There are several limitations on the detection and attribution work that can be read in detail in the methodology described above.[42] Many of the limitations from the Gilford et al methodology were addressed either mathematically or through updating the data used (such as transferring from using CMIP5 data to CMIP6 and moving to ERA5).

This indicator only considers exposure to potentially stressful temperatures and does not consider the consequences of that exposure. Indicator 1.1.2 is currently focused on attributing the health impacts of heatwave

days. Considering these two indicators together provides a more comprehensive view of the impact of climate change on health impacts across the various routes of health burden.

Future form of the indicator

There are several possibilities going forward for this indicator. The first is that it can work with a temperature threshold, either an absolute temperature level (such as the freezing point) or a percentile-based threshold like the minimum mortality temperature. It can also be extended to quantify the impact of climate change over larger regions or longer time periods. In the future, the approach could be expanded to include other weather events with health impacts, including but not limited to fire weather days, drought-days, extreme weather days, etc. It could also be combined with more detailed analyses of the consequences of high or low temperatures, allowing to extend from exposure to impacts.

Additional analysis

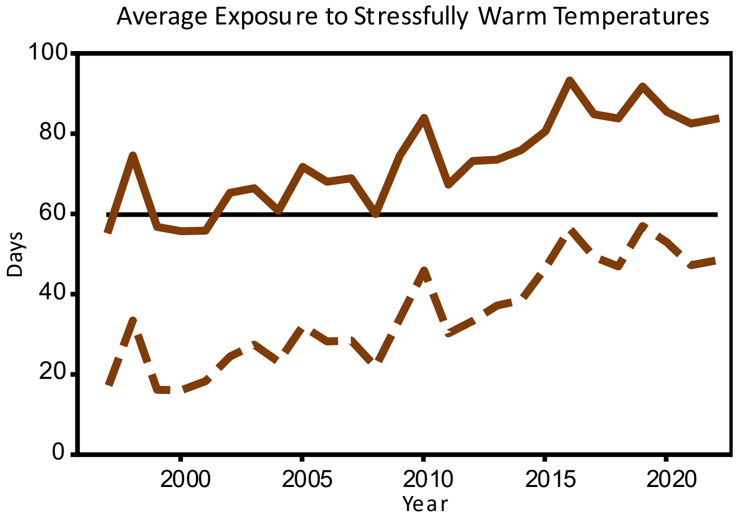

Figure 30 Population-weighted exposure to days above the 83rd percentile (solid line). In a stable climate we would expect this value to close to 62 days (black line). The number of days of exposure to warm temperature that were made at least twice as likely by climate change is plotted as a dashed line.

		Days above/below				Attributable Days			
	**Region**	**1998-2002**	**2018-2022**	**Δ**	**2022**	**1998-2002**	**2018-2022**	**Δ**	**2022**
**warm**	**SIDS**	72.1	120.9	***48.8***	***116.5***	53.3	103.4	***50.1***	***95.7***
	**Africa**	67.5	99.1	31.6	94.7	43.3	79.6	36.3	73.9
	**South and Central America**	67.2	93.4	26.2	73.7	45.7	72.4	26.7	52.3
	**Asia**	65.2	89.0	23.8	89.1	19.6	47.8	28.2	46.8
	**Northern America**	64.9	80.0	15.1	81.0	8.0	28.0	20.0	32.8
	**Europe**	54.1	67.5	13.4	69.9	6.8	25.5	18.7	31.3
	**Oceania**	50.6	61.2	10.6	58.3	6.2	18.5	12.3	17.5

Figure 31 Population-weighted exposure aggregated to Lancet Countdown regions. For each region, the average days above are listed for the periods 1998-2002 and 2018-2022. The change between these periods and the exposure for 2022 were calculated, as were the number of these days attributable using 2x criteria. The regions were sorted based on the change in exposure to warm events. The maximum change and 2022 values are highlighted.

Heat-related mortality

Indicator authors

Dr Zhao Liu

Methods

The methodology for this indicator, which tracks the global total number and spatial pattern of heat-related mortality from 2000 to 2022, remains similar to that described in the 2022 report of the *Lancet* Countdown.[15]

The heat-related excess mortality in one day *E* is expressed as

$$E = y_0 \times Pop \times AF \qquad (1)$$

where y_0 is the non-injury mortality rate on that day, *Pop* is the population size and *AF* is the attributable fraction on that day. Because every day's mortality rate is hard to obtain, y_0 is computed as the yearly non-injury mortality rate from the Global Burden of Disease data, divided by 365.

AF is calculated via the relative risk (*RR*) which represents the increase in the risk of mortality resulting from the temperature increase. *RR* is regressed as $RR = exp^{\beta(t-OT)}$, so *AF* is calculated as

$$AF = \frac{RR-1}{RR} = 1 - exp^{-\beta(t-OT)} \qquad (2)$$

where *t* is the daily maximum temperature, *β* is the exposure-response factor and OT is optimum temperature, and both parameters were adopted from Honda et al. (2014).[43] The method was applied to gridded daily temperature data from ECMWF ERA5 dataset, and gridded population data from NASA GPWv4 population dataset and ISIMIP Histsoc records, as with Indicator 1.1.1.[18] As the indicator focuses on a population that is 65 years old or older, age-structure data from United Nation World Population Prospects was also used. Because the mortality rate data of 2020–2022 has not yet been released, and the real data were highly affected by Covid-19 affecting the accuracy of the results, 2019 data were used instead.

The heat-related mortality was first calculated at grid level at 0.5° spatial resolution. Then it was accumulated to global level to produce a time-series analysis.

The calculation of the counterfactual scenario kept the data temperature unchanged at the baseline period level, and calculated mortality changing all other variables. This enabled the calculate of the change in heat-related mortality that would have been expected due to population change only, without influence of temperature change.To estimate the effects of modelled climate projections on heat-related mortality data, the input data and methodology for this indicator to the CVM 2022 report[22] was used.

Data

1. Climate data from the European Centre for Medium-Range Weather Forecasts (ECMWF) ERA5 reanalysis.[23]
2. Population data from the NASA Socioeconomic Data and Applications Center (SEDAC) Gridded Population of the World (GPWv4) and The Inter-Sectoral Impact Model Intercomparison Project (ISIMIP) Histsoc dataset.[4,5]
3. Demographic data from the United Nation World Population Prospects (UN WPP).[13]
4. Mortality rate and life expectancy data are from the Global Burden of Disease.[44]
5. Future climate projections from ISIMIP 3b protocol.[11]

Caveats

This indicator applies a unique exposure-response function across all locations and times. While its use has been demonstrated in different geographies, it does not capture local differences in the health impacts from heat exposure, which can be significant. Also, this analysis assumes exposure-response function is constant. It does not capture changes in response to heat exposure that might happen over time, as a result of acclimation and adaptation. Not capturing these changes could result in an over-estimation of heat-related deaths in later calendar years. Annual average mortality rates are used, rather than daily mortality rates (y_0). Given baseline mortality can be higher in colder months, this may lead to an overestimation of overall mortalities. Nonetheless, the trends of change in mortality due to heat exposure should still be conserved.

Only the heat-related mortality of the 65-and-older population was calculated this time, but more work needs to be done to include working group people.

Additional analysis

The change in global heat-related mortality and years of life lost is presented in Figure 32. On a global scale, the change in the number of heat-related deaths per year in the 2020–2022 period is quite small, but still maintained an increasing trend since 2000. On a regional scale, however, the changes in the number of heat-related deaths in different regions still show certain characteristics. For LC groups, the increase in the number of deaths in Asia and Europe is still relatively significant, while Oceania, Central and South America and SIDS have a more pronounced decreasing trend (Table 8). For the different WHO regions, the decline in heat-related deaths was more pronounced in the Americas, while the other continents maintained a more stable increasing trend (Table 9). For the different HDI regions, only the very high HDI country group decreased in heat-related mortality in 2022 and 2021 compared to 2020, the rest of the groups' death toll is both up and down in 2021 and 2022 compared with 2020 (Table 10). As to the change from 2000–2005 to 2017–2022, all groups under different grouping criteria show an increasing trend (Table 11, Table 12, Table 13). For instance, for the LC groups, there are three groups' heat-related mortality increased over 100%, which are South and Central America, Africa, and SIDS, and Europe increased the least of 43%. The global average growth was 65%.

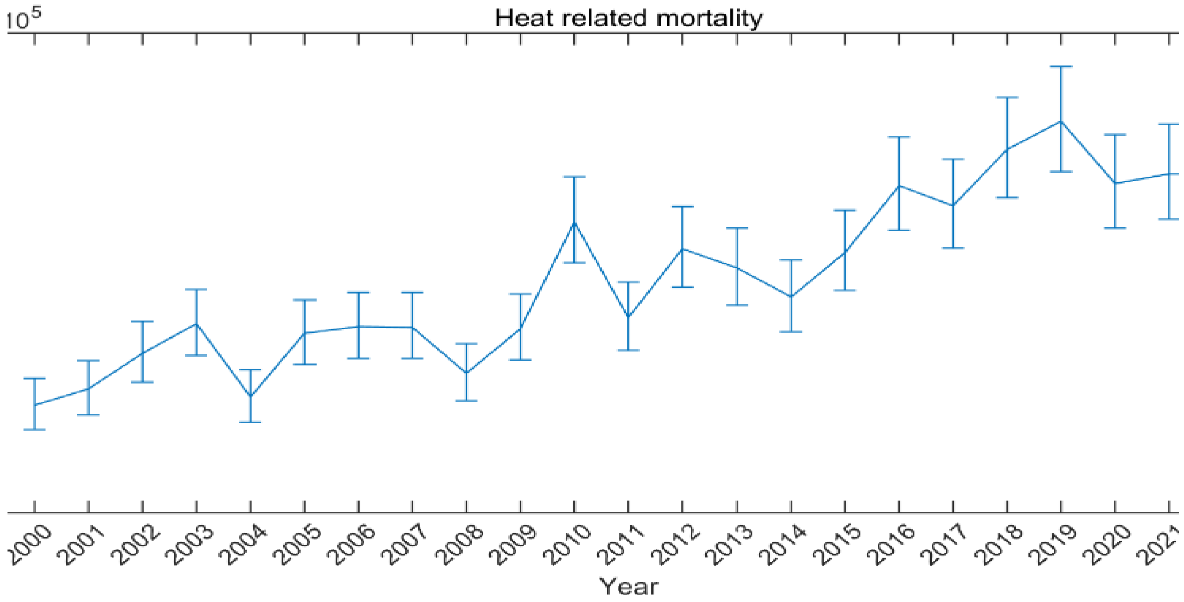

Figure 32: Heat-related mortality from 2000 to 2022.

Table 8: Change of heat-related mortality for the 65-and-older population between 2020 and 2022 for different LC groups.

LC groups	2020	2021	2022	Change in mortality (2021 to 2020)	Change in mortality (2022 to 2020)

Africa	21,879	23,756	21,937	1,877	59
Asia	136,059	145,987	150,080	9,928	14,021
Europe	94,652	96,279	99,926	1,627	5,275
Northern America	25,150	23,502	25,365	-1,648	216
Oceania	1,683	1,247	960	-436	-723
SIDS	1,152	831	389	-320	-763
South and Central America	21,641	16,239	10,696	-5,402	-10,945
Global	302,215	307,839	309,354	5,624	7,139

Table 9: Change of heat-related mortality for the 65-and-older population between 2020 and 2022 for different WHO regions.

WHO regions	2020	2021	2022	Change in mortality (2021 to 2020)	Change in mortality (2022 to 2020)
Africa	15,857	16,582	14,517	725	-1,340
Americas	47,736	40,365	36,339	-7,372	-11,397
Eastern Mediterranean	15,264	18,101	17,288	2,838	2,024
Europe	102,887	106,370	107,559	3,483	4,672
South-East Asia	34,196	32,651	37,581	-1,545	3,386
Western Pacific	86,273	93,769	96,070	7,496	9,798

Table 10: Change of heat-related mortality for the 65-and-older population between 2020 and 2022 for different HDI levels.

HDI level	2020	2021	2022	Change in mortality (2021 to 2020)	Change in mortality (2022 to 2020)
Very High	137,443	134,979	135,402	-2,463	-2,041
High	109,446	118,402	113,761	8,956	4,315
Medium	38,145	36,237	42,870	-1,908	4,725
Low	16,001	16,722	16,430	721	430

Table 11: Change of heat-related mortality between 2020 and 2022 for different LC groups.

LC groups	Average mortality of 2000–2005	Average mortality of 2017–2022	Change from 2000–2005 to 2017–2022
Africa	10,299	21,514	109%
Asia	87,648	150,411	72%
Europe	68,863	98,188	43%
Northern America	13,652	22,897	68%
Oceania	1,135	1,802	59%
SIDS	381	774	103%
South and Central America	7,151	16,521	131%
Total	189,129	312,106	65%

Table 12: Change of heat-related mortality between 2017–2022 and 2000–2005 for different WHO regions.

WHO regions	Average mortality of 2000–2005	Average mortality of 2017–2022	Change from 2000–2005 to 2017–2022

Africa	7,572	15,197	101%
Americas	21,078	40,031	90%
Eastern Mediterranean	8,685	16,425	89%
Europe	73,223	106,569	46%
South-East Asia	26,355	41,853	59%
Western Pacific	52,214	92,029	76%

Table 13: Change of heat-related mortality between 2017–2022 and 2000–2005 for different HDI levels.

HDI level	Average mortality of 2000–2005	Average mortality of 2017–2022	Change from 2000–2005 to 2017–2022
High	61,285	114,149	86%
Low	9,071	15,816	74%
Medium	27,839	44,803	61%
Very High	90,135	136,000	51%

1.2: Health and Extreme Weather-related Events

Indicator 1.2.1: Wildfires

Indicator authors

Dr Yun Hang, Prof Yang Liu, Qiao Zhu

Methods

This indicator has been updated and improved from the 2022 report of the *Lancet* Countdown by (a) extending the satellite-based wildfire population exposure estimate with cloud corrections, (b) applying a much more precise filtration of non-fire hot spots reported by MODIS, (c) revising the vertical distribution of the fire plume in SILAM model allowing for a more accurate representation of near-surface concentrations, and (d) introducing future fire and fire smoke projections by using a new Fire Forecasting Model as well as projecting the exposure to very high or extremely high wildfire danger till the end of this century.

Wildfire

The change in population exposure to wildfire is represented as the change in the average annual number of person-days exposed to wildfire in each country. Satellite-observed active fire spots were aggregated and spatially joined with gridded global population data from the NASA SEDAC GPW v4.11 dataset on a global 0.1° × 0.1° resolution grid. Grid cells with a population density ≥400 persons/km^2 were excluded to remove urban heat sources unrelated to wildfires. Cloud cover information was incorporated into each grid cell of the satellite-observed active fire data to address the issue of fire spot underestimation due to cloud obscuration. The mean annual number of person-days exposed to wildfire during the most recent five years (2018–2022) was compared with the baseline period of 2003–2007 (Figure 33).

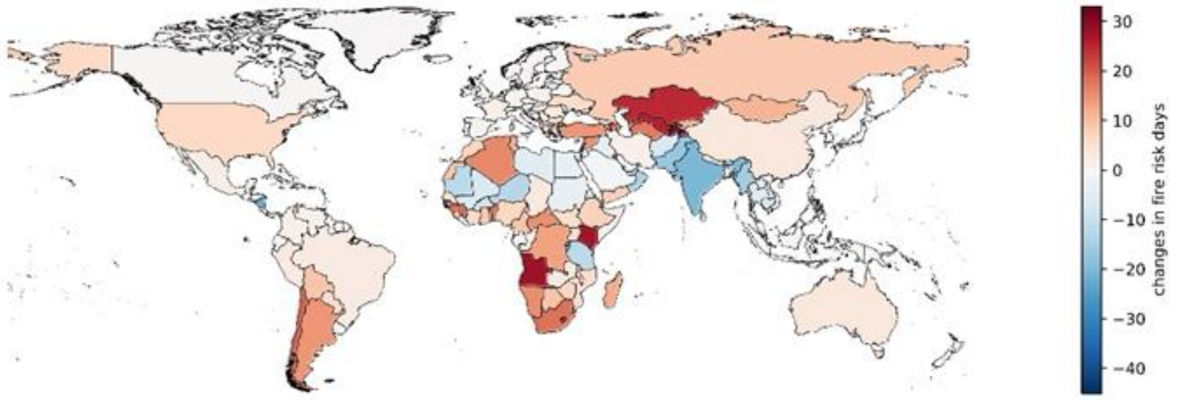

Figure 33: Population-weighted mean changes in very high and extremely high fire danger days in 2018–2022 compared with 2001–2005. Large urban areas with population density ≥ 400 persons/km^2 are excluded.

The fire danger risk, represented in terms of the Fire Danger Index (FDI), tracks human exposure to days in the meteorological danger risk of wildfires. It accounts for air temperature, relative humidity, wind speed and drought effects to capture the risk of a fire starting, its rate of spread, its intensity, and its difficulty of suppression. The historical FDI data were provided by ECMWF ERA5 atmospheric reanalysis, FDI is a numeric rating with values 1-6 representing very low, low, medium, high, very high and extreme fire danger risk, respectively. Daily FDI data were aggregated so as to obtain the yearly number of days of each fire danger risk level at every 0.25° x 0.25° grid cell. The changes in mean number of days exposed to very high or extremely high fire danger risk (defined as FDI ≥ 5) were collected for the most recently available period, 2018 to 2022, and compared with a baseline from 2001 to 2005. For projection analysis, FDI was determined by daily Fire Weather Index (FWI). Specifically, the Global ECMWF Fire Forecasting (GEFF) model was performed to calculate daily FWI values. The input data were daily gridded climate data at 0.5° × 0.5° resolution derived from the ISIMIP3b dataset, for five general

circulation models (GCMs) under SSP1-2.6 (i.e., low-emission scenario) and SSP3-7.0 (i.e., high-emission scenario), respectively. The modelling periods included 1995–2014 (baseline), 2021–2040 (near-term), 2041–2060 (medium-term), and 2081–2100 (long-term). In the second step, the FWI values were categorized into six levels of FDIs by the European Forest Fire Information System. The changes in mean number of days exposed to very or extremely high wildfire risks (defined as FDI≥5) were collected for three projection periods (i.e., near-term, medium-term, and long-term), compared with the baseline period.

Data

1. MODIS Fire Radiative Power (FRP) observations MOD14/MYD14 from the NASA Fire Information for Resource Management System (FIRMS).[45]
2. Cloud cover data from the EarthEnv Global 1-km Cloud dataset.[46,47]
3. Fire danger indices historical data produced by the Copernicus Emergency Management Service for the European Forest Fire Information System (EFFIS).[48]
4. Population data from the NASA Socioeconomic Data and Applications Center (SEDAC) Gridded Population of the World (GPWv4) and from the Hybrid gridded demographic data for the world, 1950–2020 (1.0).[49]
5. Gridded population data from SSP1 and SSP3 scenarios (for SSP1-2.6 and SSP3-7.0, respectively) from CIESIN data with a spatial resolution of 0.5° × 0.5.[50]

Caveats

There are two caveats. First, the fire danger index represents a potential fire risk calculated by meteorological parameters. It does not represent actual fire events. The actual fire events can also be influenced by anthropogenic factors, such as human-induced land use and land cover changes, industrial-scale fire suppression, and human induced ignition. The fire danger index does not account for the potential fertilizer effect of CO_2 and the associated changes in vegetation and thus the fuel load of fire. Further, it does not consider potential changes in lightning ignitions, which can be affected by climate change, but the effect is highly uncertain.

Second, in projection of fire danger index, the FWI calculation requires daily temperature, relative humidity, wind speed at 12:00 am local time and precipitation at 12:00 am local time accumulated over the previous 24 hours. Since the daily temperature, relative humidity, wind speed at 12:00 am local time are difficult to obtain for projection, they were replaced with the daily maximum temperature, minimum relative humidity, and maximum wind speed. To ensure consistency, the same input parameters were used to calculate FWI values for the baseline period.

Future form of the indicator

Active fire spots as obtained from MODIS represent raw fire information and do not differentiate between wildfire and prescribed burns. Furthermore, the spatial resolution of the indicator is 0.1° which may underestimate wildfire exposure and introduce a bias. To correct this, future improvements could consider increasing the indicator's resolution to 0.01° using global 1-km datasets. As this is computational- and time-consuming, these improvements will be introduced in the 2024 report. The replacement of climate input data for fire danger index projection would increase the uncertainty of outcomes. Thus, a more reliable and accurate method will be developed to improve the input data for projection in the 2024 report.

Additional analysis

1.1 Wildfire risk indicator by HDI level, WHO region and Lancet Countdown region

The population-weighted mean changes in extremely high and very high fire danger days in 2018–2022 relative to 2001–2005 for each HDI category, each WHO region or Lancet Countdown region are shown in the figures and tables below (Table 14, Table 15, Table 16, Figure 34, Figure 35, Figure 36). Low HDI countries and African countries appear to have the largest growth in climatological danger of wildfire.

Table 14: Population-weighted mean changes in very high or extremely high fire danger days in 2018–2022 compared with 2001–2005 by HDI level. The number and percentage of countries with increased exposure by HDI level are calculated. Large urban areas with population density ≥400 persons/km^2 are excluded.

HDI level	Population-weighted mean changes	
	Mean Change in Fire Danger Days	Number (%) of Countries with Increased Fire Danger Days
Low	3.2	21 (66%)
Medium	3.1	23 (56%)
High	3.0	25 (57%)
Very High	1.4	41(67%)

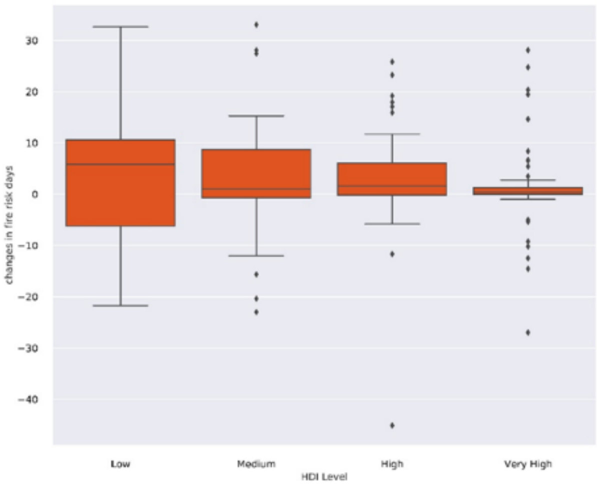

Figure 34: Population-weighted mean changes in very high or extremely high fire danger days in 2018–2022 compared with 2001–2005 by HDI level.

Table 15: Population-weighted mean changes in very high or extremely high fire danger days in 2018–2022 compared with 2001–2005 by WHO region. The number and percentage of countries with increased exposure by WHO region are calculated. Large urban areas with population density ≥ 400 persons/km^2 are excluded.

**WHO region**	Population-weighted mean changes	
	Mean Change in Fire Danger Days	Number (%) of Countries with Increased Fire Danger Days
**African**	5.6	34 (72%)
**Americas**	0.9	18 (46%)
**Eastern Mediterranean**	-2.1	9 (41%)
**European**	4.7	44 (88%)
**South-East Asian**	-4.6	2 (20%)
**Western Pacific**	0.2	6 (25%)

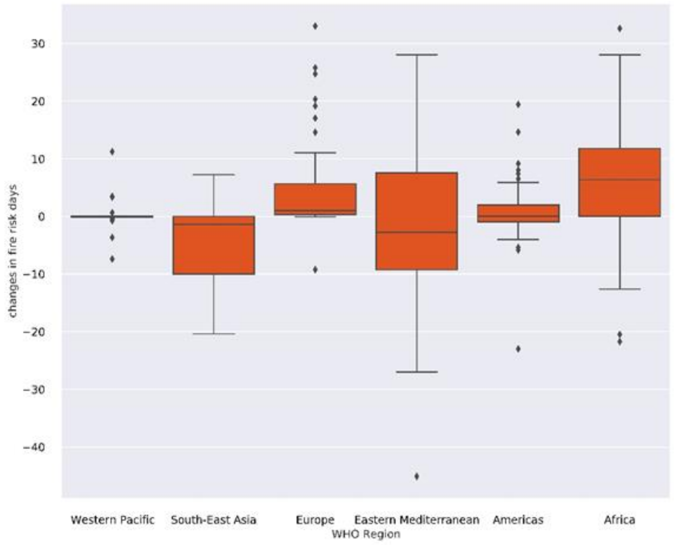

Figure 35: Population-weighted mean changes in very high or extremely high fire danger days in 2018–2022 compared with 2001–2005 by WHO region.

Table 16: Population-weighted mean changes in very high or extremely high fire danger days in 2018–2022 compared with 2001–2005 by *Lancet* Countdown region. The number and percentage of countries with increased exposure by *Lancet* Countdown region are calculated. Large urban areas with population density ≥400 persons/km^2 are excluded.

Lancet Countdown region	Population-weighted mean changes	
	Mean Change in Fire Danger Days	Number (%) of Countries with Increased Fire Danger Days
**Africa**	5.5	35 (73%)
**Asia**	1.2	19 (42%)
**Europe**	1.6	34 (87%)
**Northern America**	3.5	2 (100%)
**Oceania**	2.1	2 (100%)
**SIDS**	0.7	11 (28%)
**South and Central America**	1.5	11 (61%)

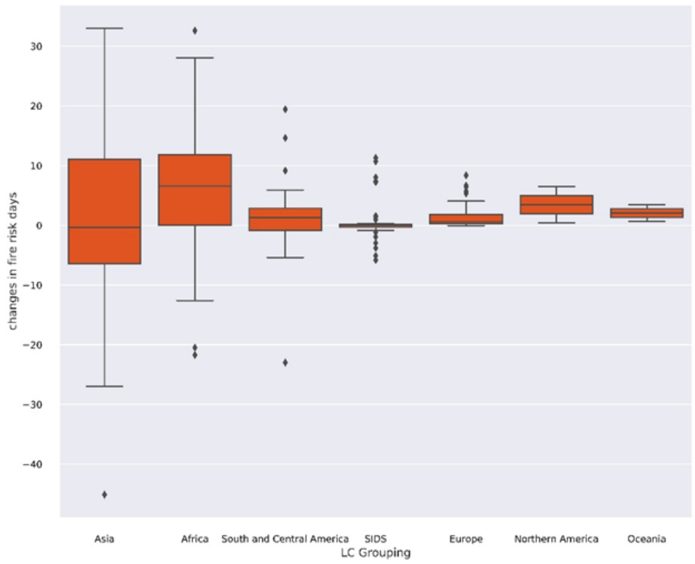

Figure 36: Population-weighted mean changes in very high or extremely high fire danger days in 2018–2022 compared with 2001–2005 by *Lancet* Countdown Region.

1.2 Projected trend of wildfire risk indicator by *Lancet* Countdown region

At regional level (defined by *Lancet* Countdown region), the largest increase of days exposed to very high or extremely high by the end of the century under the high-emission scenario is projected to occur in Asia (37days), followed by Africa (36 days) and South and Central America (20 days). While the increase in high wildfire risk days is projected to be much less under the low-emission scenario, with 19 days for Asia, 11 days for Africa and 4 days for South and Central America. Figure 36 and Figure 37 indicate the most pronounced increasing "climate-related hazard" trend was observed and projected in Africa.

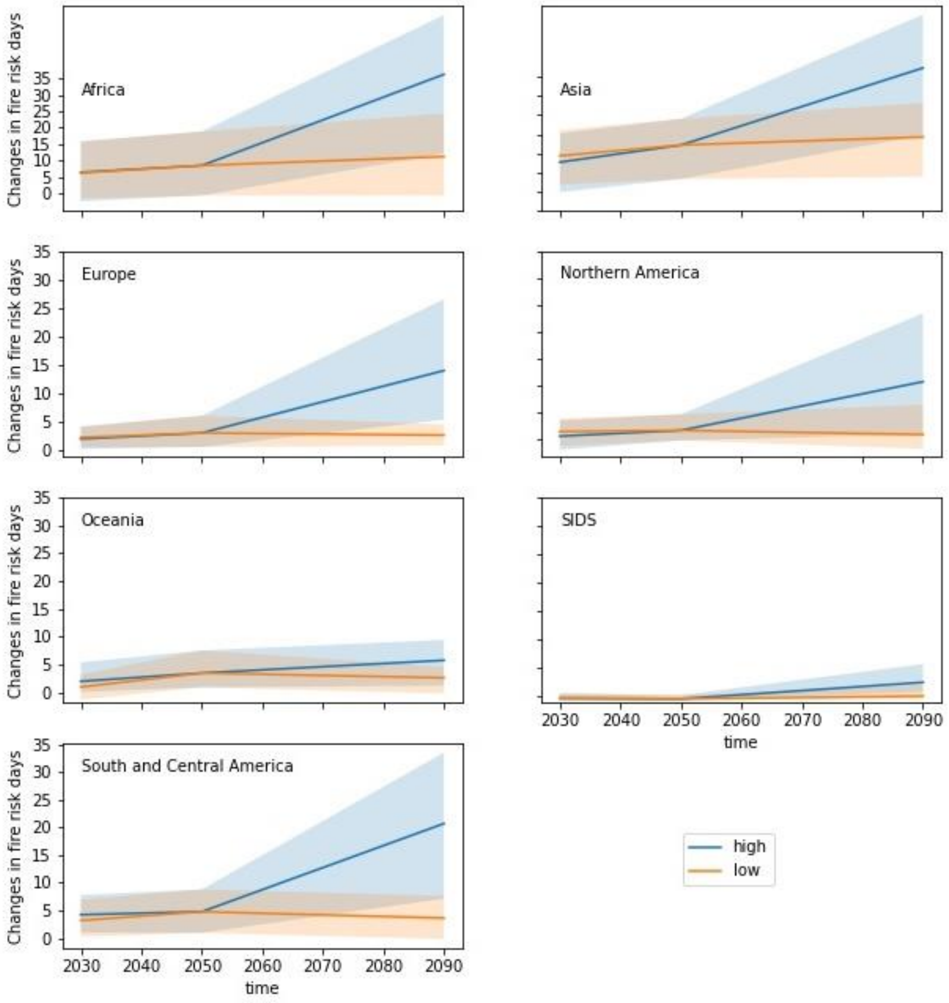

Figure 37: Population-weighted mean changes in very high and extremely high fire danger days in the high-emission and in the low-emission by *Lancet* Countdown region. The shaded areas represent the range between the maximum and minimum values obtained using the 5 GCM.

1.3 Satellite-based wildfire exposure indicator

The change in the annual mean number of person-days exposed to wildfire by *Lancet* Countdown (LC) region, WHO region, and HDI level in 2018–2022 compared with 2003–2007 are shown in Table 17. As for the LC and WHO regions, after correcting for global cloud cover, Africa appears to have the largest increase in wildfire exposure (+0.3 million persons). Moreover, 62% of Eastern Mediterranean countries experienced an increase in wildfire exposure. European, South-East Asia, Western Pacific, South and Central America appeared to have a decrease in wildfire exposure. Low HDI countries appeared to have the largest increase in wildfire exposure (+0.5 million persons). 62.5% of low HDI and 50% of medium HDI countries experienced an increase in wildfire exposure. High and very high HDI countries appeared to have a decrease (-0.04 and -0.01 million persons) in wildfire exposure.

Table 17: The annual mean number of person-days exposed to wildfire by LC region (A), WHO region (B), and HDI level (C) in 2018–2022 compared with 2003–2007 (unit: 10,000 persons). The number and percentage of countries with increased exposure are calculated. Large urban areas with a population density ≥400 persons/km^2 are excluded.

A

LC Region	Change of Exposure	Number (%) of Countries with Increased Exposure
South and Central America	-44.6	7 (43.8%)
Africa	27.5	29 (61.7%)
Asia	-23.6	19 (43.2%)
SIDS	-0.8	15 (42.9%)
Oceania	2.6	1 (50.0%)
Europe	-15.6	12 (29.3%)
Northern America	0.7	1 (50.0%)

B

WHO Region	Change of Exposure	Number (%) of Countries with Increased Exposure
Americas	-22.0	15 (44.1%)
Africa	27.2	27 (57.5%)
South-East Asia	-120.7	2 (20.0%)
Western Pacific	-10.1	11 (47.8%)
Europe	-13.1	16 (30.8%)
Eastern Mediterranean	22.7	13 (61.9%)

C

HDI Level	Change of Exposure	Number (%) of Countries with Increased Exposure
High	-39.9	22 (44.9%)
Medium	5.3	21 (50.0%)
Low	48.2	20 (62.5%)
Very High	-14.6	21 (32.8%)

Wildfire smoke

Indicator authors

Dr Risto Hänninen, Dr Rostislav Kouznetsov, Prof Mikhail Sofiev

The indicator shows personal exposure to fire-originated fine particles ($PM_{2.5}$) at the global scale during the last 20 years, 2003–2022, which correspond to the complete period available from MODIS instruments onboard Aqua and Terra satellites (Figure 39). The smoke dispersion is computed with resolution of 0.5°.[51] The atmospheric emission of fire-originated fine particles is computed by the Integrated System for vegetation fires IS4FIRES,[52–55] which is interfaced to the System for Integrated modeLling of Atmospheric coMposition SILAM[51,56,57]. The input data for the computations are the active-fire retrievals of Fire Radiative Power (FRP) by MODIS instrument

onboard Aqua and Terra satellites.[58] FRP serves as a proxy for estimating the amount of pollutants released by a fire to the atmosphere[59] and, combined with meteorological parameters, is used to calculate the vertical profile of the smoke injection in the air.[53,54] Subsequent transport, transformations, and removal from the atmosphere are computed by SILAM following the usual procedures of atmospheric composition modelling. The obtained hourly global distribution maps are aggregated over time and space, weighted upon necessity with the population density, thus providing the set of output parameters: gridded and country-integrated time-resolving individual and population exposure to fire-induced smoke.

There are several peculiarities and improvements of the 2023 fire smoke indicator in comparison with its 2022 version. MODIS active-fire retrievals include many hot spots not related to wildland fires: large-scale metallurgy plants, oil refineries, volcanoes, etc. Their removal is a strong challenge because such heat sources may appear and disappear randomly.[60] Previous non-fire mask of IS4FIRES was developed in 2015[55] and needed an update, which has been implemented in the new dataset strongly reducing the non-fire related false positives. Vertical distribution of the smoke injection followed the same procedure as in the 2022 report[53,54] but a small correction in its implementation slightly increased the near-surface concentrations, thus more accurately reflecting the distribution of smoke from small fires adjacent to the main burning area. The adjustment has led to ~10% increase of near-surface concentrations in the vicinity of the large fires.

The Fire Forecasting Model, FFM, is based on a machine learning approach that relates the meteorological, land use, and geophysical variables to the fire energy released under similar conditions in the past. Assuming that this relation will be valid also in future, the fire activity and the corresponding exposure can be calculated for distant years (Figure 40).

Data

1. MODIS Fire Radiative Power (FRP) observations MOD14/MYD14 from the NASA Fire Information for Resource Management System (FIRMS).[45]
2. Population data from the NASA Socioeconomic Data and Applications Center (SEDAC) Gridded Population of the World (GPWv4) and from the Hybrid gridded demographic data for the world, 1950-2020 (1.0).[49]
3. Daily surface concentration of fire-related PM2.5 from the Finnish Meteorological Institute.[51]

Caveats

MODIS fire radiative power and other fire products constitute the longest homogeneous global fire time series. However, low-orbit retrievals are available at each specific place only a couple of times per day. As a result, the instrument misses the fire if the scene is obscured by clouds, or the fire is too small. This omission error probability depends on region and season varying from ~20–30% in Europe in summer up to 70% in some equatorial areas. The smallest fire that can be detected by MODIS at night in clear-sky conditions and nadir view is about 4 MW, but the detection limit is close to 40 MW at the edge of the observed swath during day.

Corrected caveat. The non-fire hot spots issue has been largely reduced in the current indicator release and can be considered negligible. As seen from Figure 39, the new non-fire mask is much more efficient in removal industry-related hot spots: e.g., the region of Persian Gulf is no longer reported as a wildfire-prone area with heavy upward trend, certain reductions of the trend artifacts are visible in industrial regions of the US, Siberia, etc. This was a significant caveat of the 2022 indicator, eliminated in the current release.

Future form of the indicator

In 2022, both Aqua and Terra satellites exited their constellation orbits: the satellites no longer have fuel for correcting the orbits and maintain the equatorial crossing time with accuracy of two minutes. By October 2022, Terra has reached 15 minutes shift from its usual timing. Also, both satellites were lowered in their orbits from 705 km, by about 7 km. Since fire intensity has a strong diurnal cycle and retrievals are sensitive to the observation geometry, starting from 2023, the MODIS data will no longer constitute a homogeneous fire dataset. Therefore, the wildfire indicator will be rebased to VIIRS and SLSTR instruments, which have been operational for a few years and provide similar variables, albeit with different features. Merging the new instruments into the existing time series and continuing the harmonized line will be a challenge for the forthcoming reports.

Additional analysis

Exposure by HDI grouping

When comparing the last five years (2018–2022) with first five years (2003–2007) one may notice that the biggest drop by 30% in personal fire-PM exposure has appeared in countries with high HDI, while the biggest increase of 34% can be seen in countries with very high HDI, strongly driven by exceptionally high concentrations in Russia in 2021, plus increases in USA and Canada (Figure 38). If considering the population exposure, the largest drop of 18% is still in high HDI countries, but the strongest increase of 55% appears in low HDI countries, followed by 44% increase in medium HDI countries, both driven by the strongly increasing population.

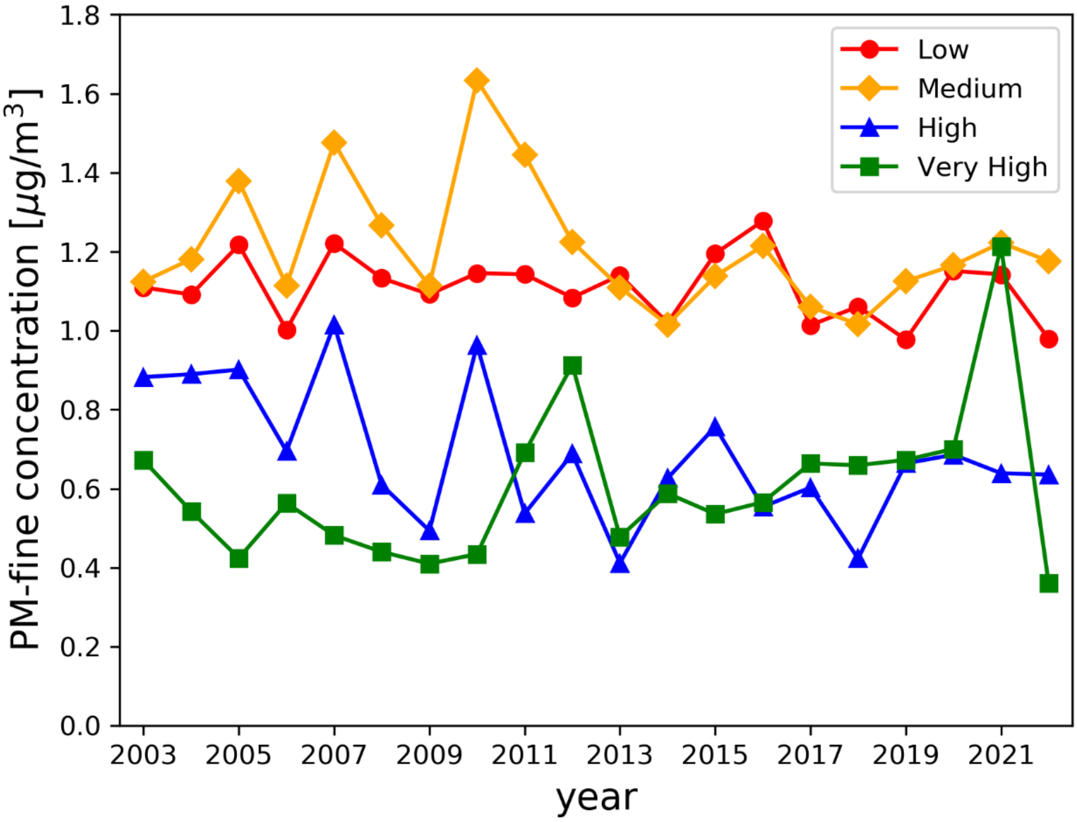

Figure 38. Fine PM area-weighted concentration for countries with different Human Development Index.

Exposure over Lancet Countdown (LC) and WHO regions.

Regional analysis reveals the differences between the mean individual and population exposure in different regions (Figure 41). Small Island Development States (SIDS, LC region) experience the lowest mean individual and population exposure. The maximum individual exposure is experienced in Africa and South and Central Americas, episodically also in Oceania (largely depending on the fire intensity in Indonesia). The population exposure is additionally controlled by the population density; hence Asia and Africa show the highest levels. Year-to-year variations are the highest in Oceania and Europe, with some annual spikes exceeding the mean level by several times. The fire occurrence varies from year to year, but particularly large variations are in Oceania (driven by El-Niño and related droughts) and Europe, which has a comparatively low exposure on average but can experience high peaks if the fire occurrences coincide with wind that brings the plumes to densely populated areas.

Trends (Figure 39 for gridded trends and Figure 42 for country-averaged ones) computed over the 20 years and with improved non-fire mask largely confirm the conclusions of the 2022 report, except for Middle East and Persian Gulf, where both exposure and its upwards trend were essentially zeroed. Four regions with upwards exposure are North America, Russia, India, and Pakistan, as well as Democratic Republic of Congo. Among those, the only statistically significant ($p<0.05$) trends were in India and Pakistan (the trends in North America and Russia was close to the significance threshold). Statistically significant negative trends were in equatorial Africa, China, Kazakhstan, Myanmar, and Vietnam, however the tendency of the exposure reduction, albeit not

statistically significant, were visible in many countries. This is related to too short time series of MODIS (albeit the longest homogeneous ones available) and to a very high variability of the fire occurrence. It is therefore necessary to obtain the longer time series by combining and homogenising observations from several satellites, and to make use of more sophisticated trend analysis procedures that would allow a more reliable trend assessment with existing data.

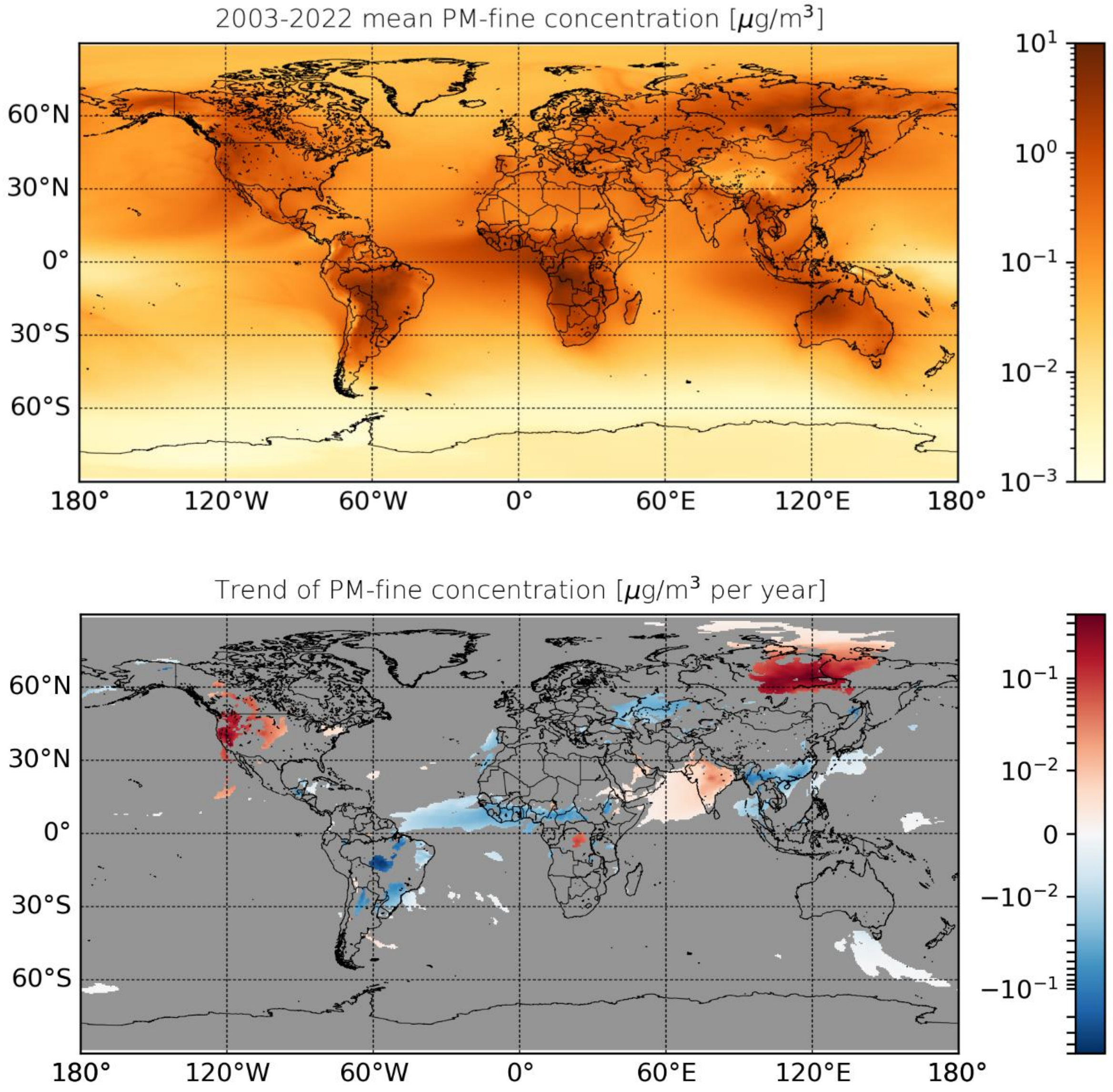

Figure 39: Gridded mean personal exposure rate to fire-induced PM (upper panel) and its trends (lower panel), 2003–2022. Only statistically significant trends ($p<0.05$) are shown.

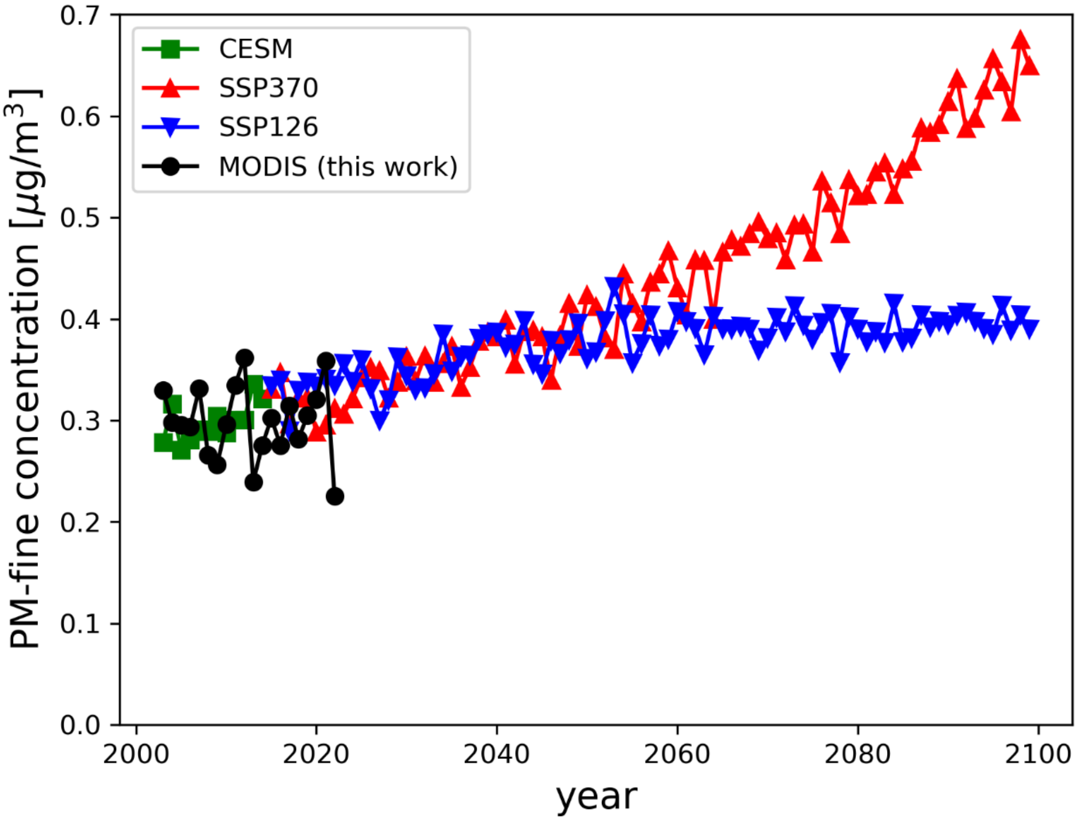

Figure 40: Individual exposure rate for MODIS period (satellite-retrieved fires, black curve) and for future scenarios SSP 3-70 (red curve) and SSP 1-26 (blue curve). The scenario exposure estimates are scaled (a factor of 2.25) to meet the global mean of the MODIS time period.

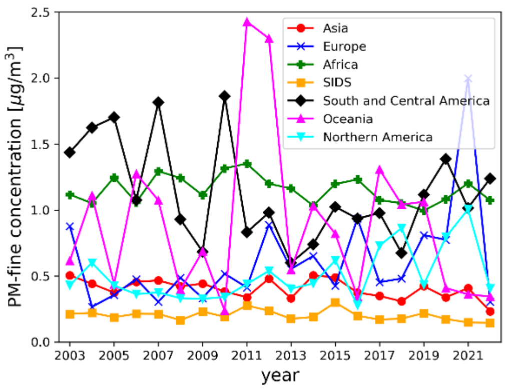

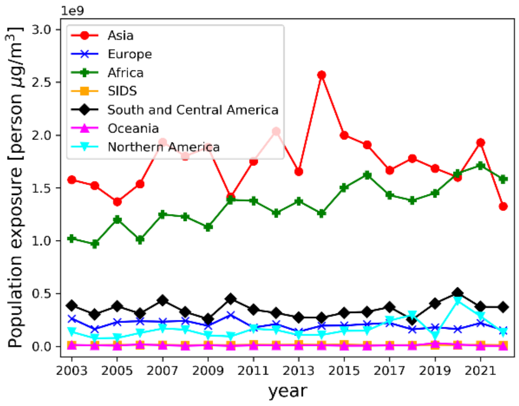

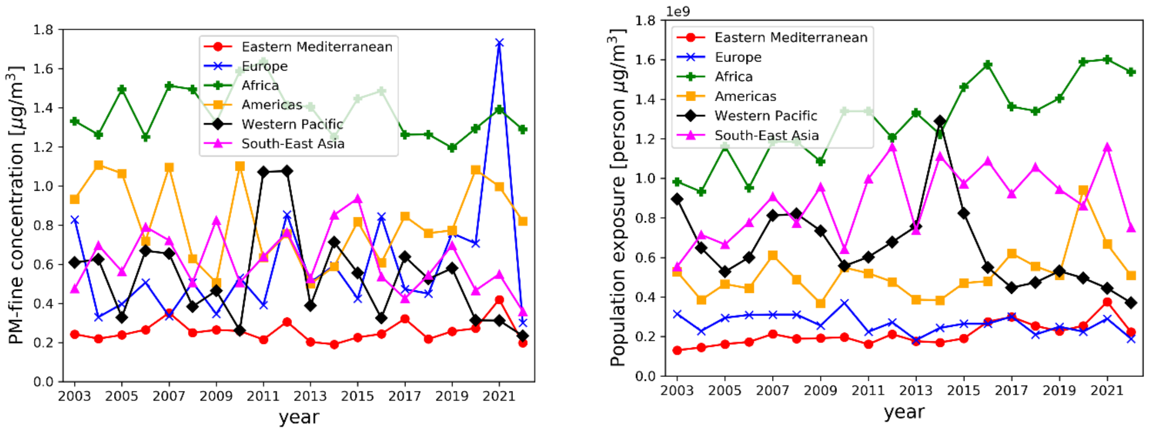

Figure 41: Mean individual (left column) and population (right column) exposure for the LC (upper row) and WHO (lower row) regions.

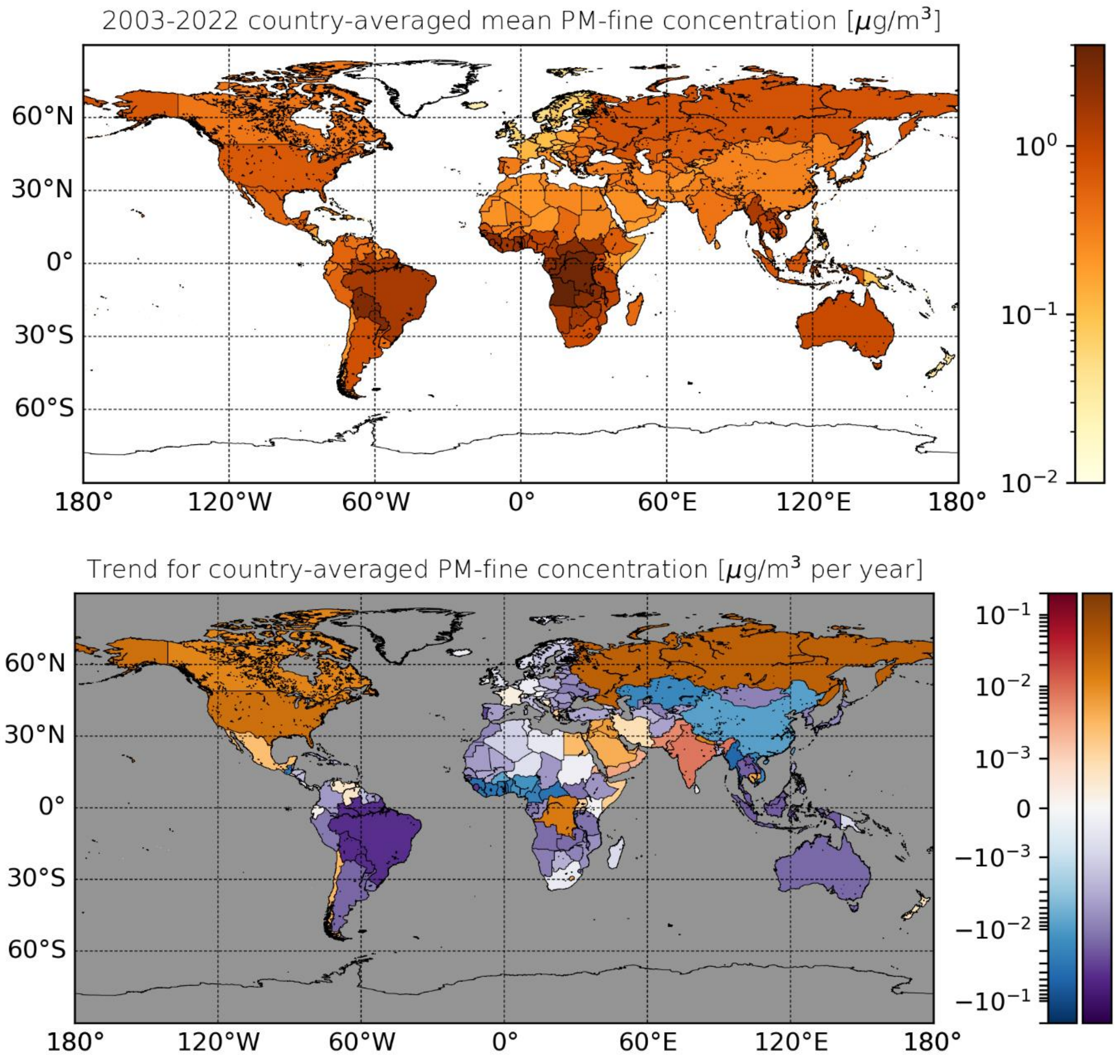

Figure 42 Country-averaged individual PM2.5 exposure (upper panel) and its trend (lower panel). Statistically significant trends (P<0.05) are shown via blue-red colours, where not significant ones are shown via violet-brown colours.

Indicator 1.2.2: Drought

Indicator authors

Dr Marina Romanello

Methods

The data source for this indicator has been updated for the 2023 report.

The drought indicator uses the 6-monthly Standard Precipitation Evapotranspiration Index (SPEI6)[61] as a measure of the land surface affected by drought events. This index allows for both the intensity and the duration of droughts to be taken into account. It captures the influence of both altered precipitation patterns, and of potential evapotranspiration on drought severity.

SPEI6 data were obtained from the SPEI Global Drought monitor. The Global Drought monitor uses mean temperature data from the NOAA NCEP CPC GHCN-CAMS gridded dataset[62] and monthly precipitation data from the 'first guess' Global Precipitation Climatology Centre (GPCC).[63] GCPC data, which have an original spatial resolution of 0.5º x 0.5°, are interpolated to the resolution of 1° x 1°. Potential evapotranspiration is calculated using the Thornthwaite equation.

The SPEI Global Drought Monitor calculates SPEI values using constantly updated climate data at a global scale with a 1° x 1° spatial resolution and a monthly time resolution. SPEI time scales between 1 and 48 months are provided. For the indicator the 6-monthly SPEI value is used (SPEI6) and the calibration period is set to January 1950 to December 2010. SPEI6 data for 1950-present were downloaded from the Global SPEI Database.

Droughts were defined according to three severity levels using the SPEI thresholds indicated in Table 18, as defined by the Federal Office of Meteorology and Climatology MeteoSwiss.[64] In order to detect excess (unusual) drought events, "excess severe drought events" were defined as yearly counts of months in drought for each grid cell which exceed two standard deviations above the mean of the yearly counts of months in drought for the baseline period of 1986–2005. The excess events were defined for each SPEI severity level of drought independently, and the percentage of land area exposed to excess drought events at the different severity levels was calculated.

Table 18: Summary of drought severity thresholds as defined by the Federal Office of Meteorology and Climatology MeteoSwiss.

SPEI value	Description	Frequency of event in respective month
< -1.3	severe drought	1-2 x in 20 years (i.e., 10% if the time)
< -1.6	extreme drought	1-2 x in 40 years (i.e., 5% of the time)
< - 2	exceptional drought	1 x in 50 years or less (i.e., ≤2% of the time)

Data

1. SPEI6 data from the Global SPEI Database, SPEIbase (Consejo Superior de Investigaciones Cientificas).[65]

Caveats

A limitation of this indicator is that it only captures the impacts of climate change on meteorological drought but does not capture the impacts of climate change on hydrological or agricultural drought, which can have major

health impacts too. Moreover, it does not measure the direct relationship between a drought and the population living in, or depending on, drought-affected areas. It is not possible to do a population-based weighting because many people affected by a drought may not live in the area affected, e.g., in the case of droughts affecting agricultural areas (which are generally sparsely populated) with impacts on the food supply. It is therefore difficult to determine the trends in persons affected by drought from the trends of severe drought areas.

Further work is required to link reported drought damages in societies to climatic indicators. This would require a better understanding of the exposure factors of populations.

Future form of the indicator

Further development of the indicator will focus on using a combination of indices that capture agricultural hydrological drought, and meteorological drought, and better capture the health implication of drought events.

Additional analysis

The percentage of global land area affected by extreme drought has been increasing since the early 1990s. A linear regression shows that, from 1978 to 2021, the percentage of global land area affected by at least 1 month of extreme drought has been increasing by 0.87% each year ($p < 2x10^{-16}$, $R^2 =$ 0.827; Figure 43). The year 2022 saw 12 months in extreme drought in the Southern Amazon region, Western Sahara, and Horn of Africa (Figure 44), all regions which have seen over 8 months more in extreme drought per year from 1951–1960 to 2013–2022. An increasing percentage of the global land area is affected by more extreme drought, with sharp increases in the global land area affected by severe, extreme, and exceptional drought events in any given month over the past 20 years (Figure 45), and an increasing number of months in drought affecting the global land area (Figure 46).

At a regional level, the WHO region that experienced the highest increase in the incidence of extreme drought is the Eastern Mediterranean region, followed by the African region (Figure 47A). Africa, a region in which millions are already grappling with food water insecurity, was the most affected continental region in 2013–2022, with 64% of its total land area affected by at least one month in extreme drought. Africa was also the continental region which saw the biggest increase in the incidence of drought, with 55% more of its land area affected by extreme drought in 2013–2022 than in 1951–1960 (Figure 47B).

Looking at HDI country groups, countries with low HDI saw the biggest increase in drought incidence from 1951–1960 to 2013–2022, with an additional 46% of the total land area affected by at least one month in extreme drought in recent years (Figure 47C).

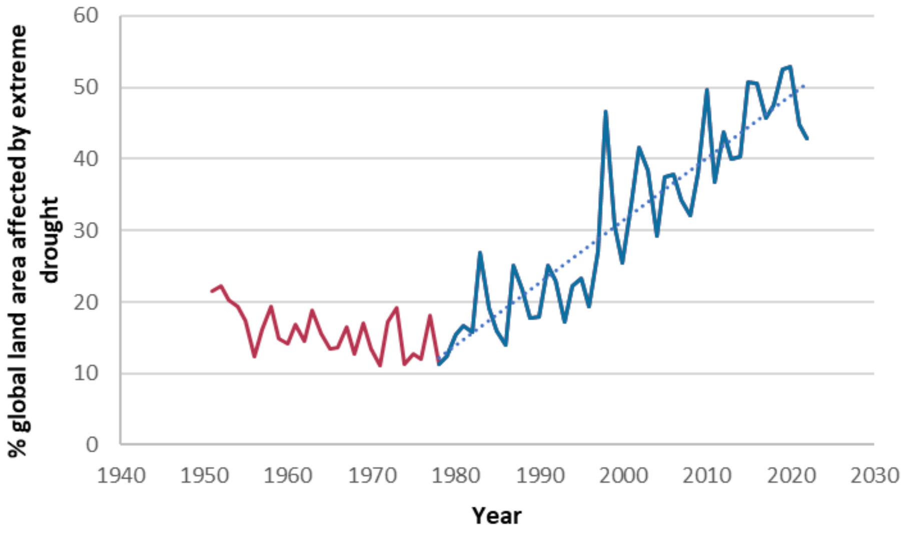

Figure 43: Percentage of the global land area affected by at least 1 month in extreme drought (SPEI6 ≤ -1.6). The dashed blue line represents the linear regression of the segment with the highest linear correlation,

marked as a continuous blue line (1978-2022). The linear regression has slope = 0.872, $p < 2x10^{-16}$, R^2 = 0.827.

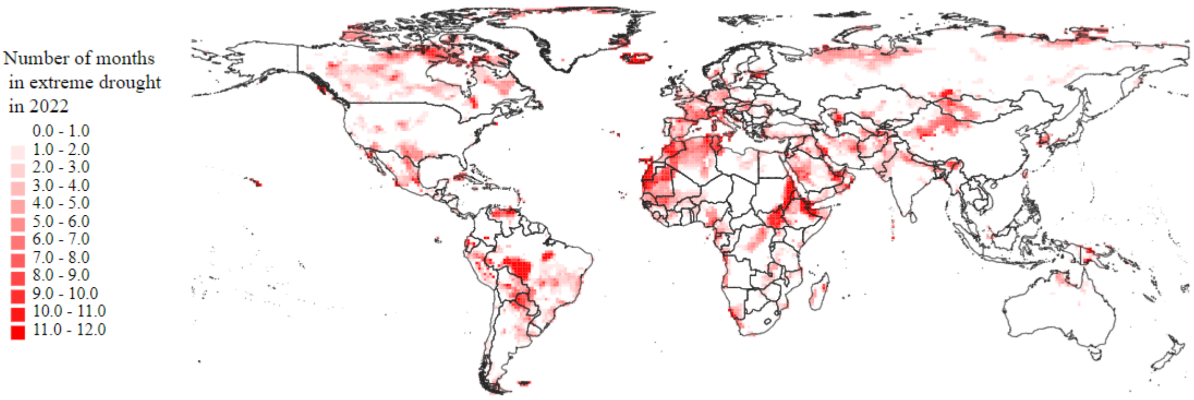

Figure 44: Number of months affected by extreme drought in 2022.

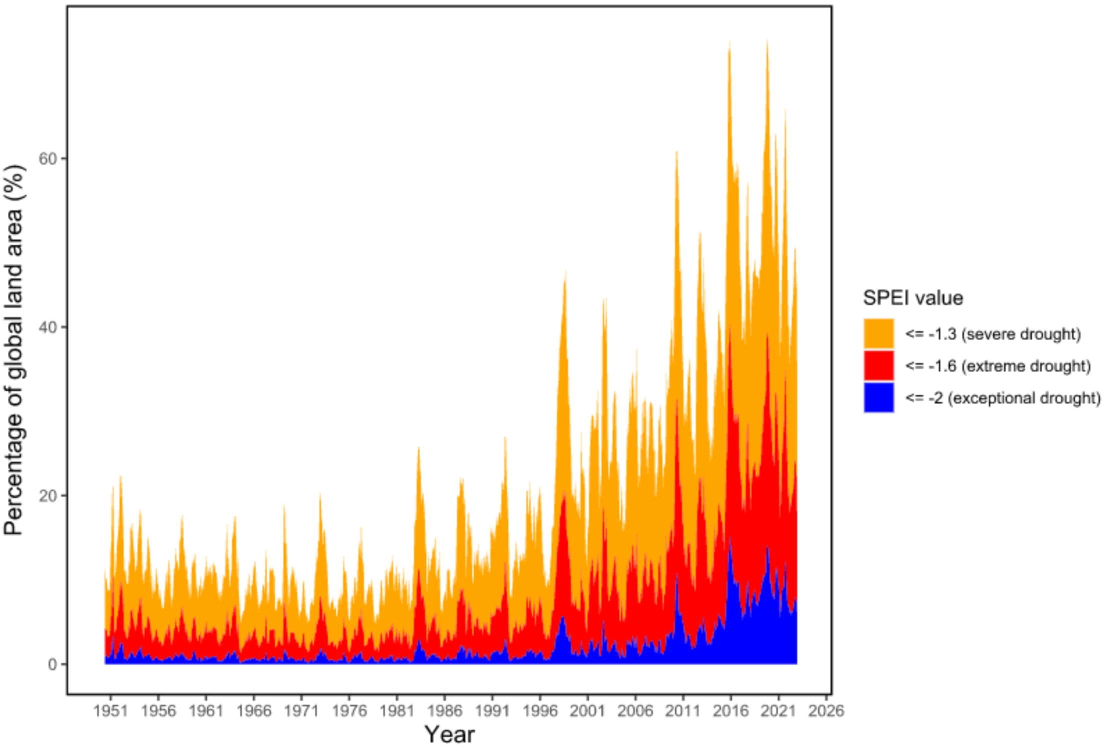

Figure 45: Percentage of the global land area affected by drought events per month, from 1950 to 2022. Severe drought is defined by a SPEI of ≤1.3; extreme drought is defined by a SPEI of ≤1·6 and exceptional drought is defined by a SPEI of ≤2.

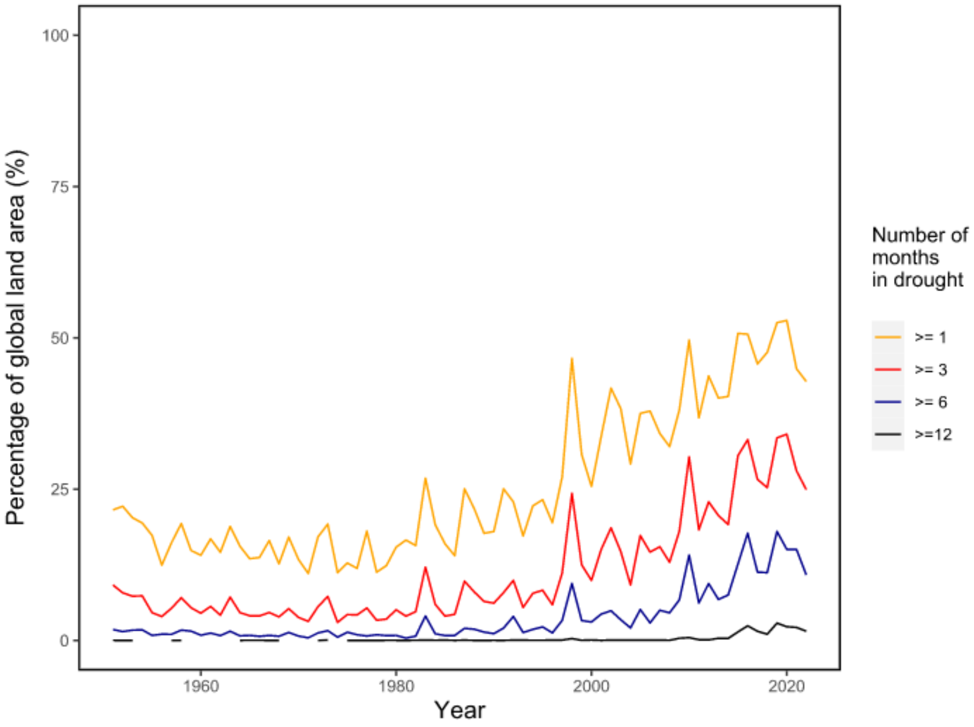

Figure 46: Percentage of the global land area affected by 1, 3, 6 or 12 months of extreme drought (SPEI ≤1·6) per year.

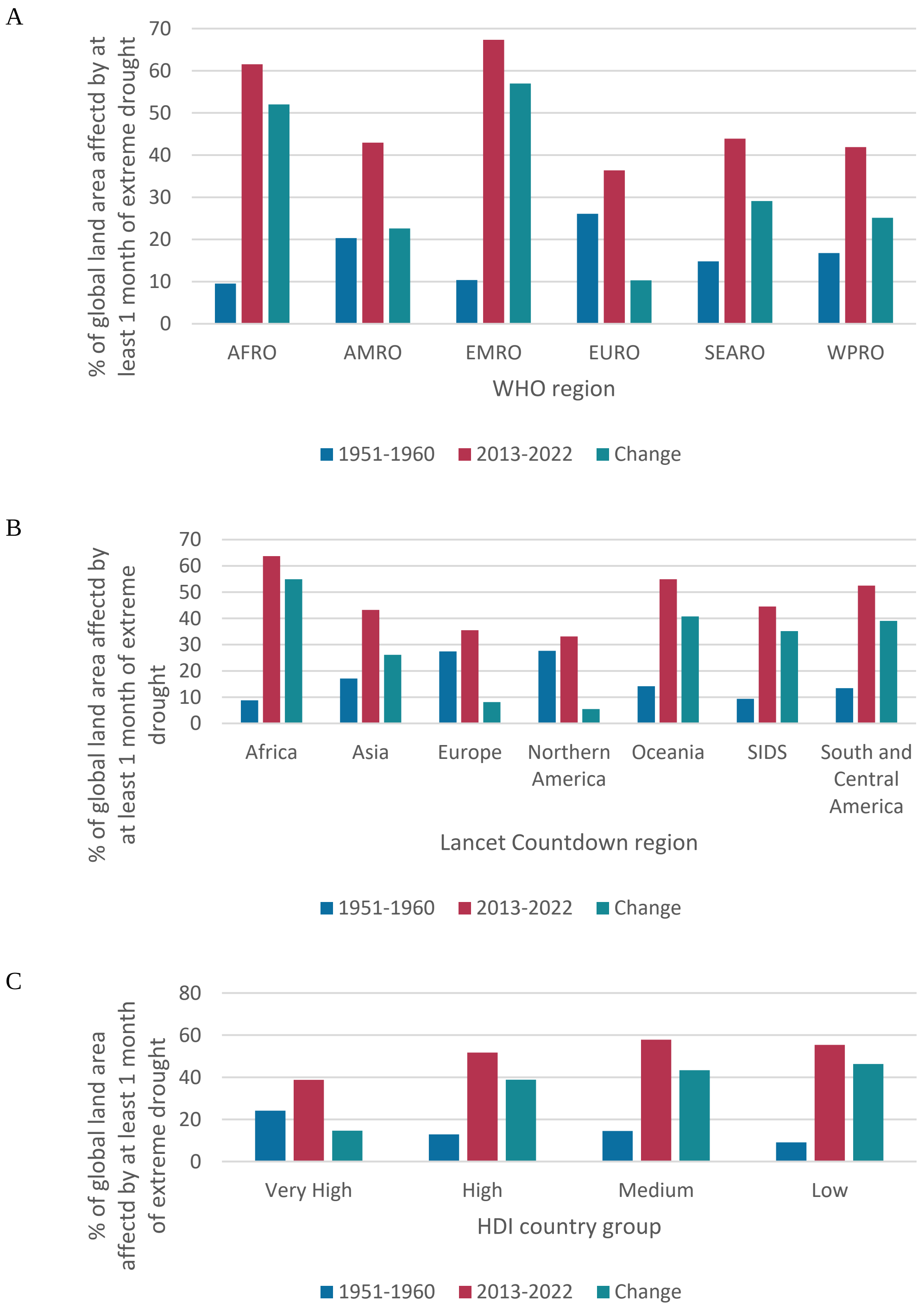

Figure 47: Total average percentage of the global land area affected by extreme drought in per year in 1951–1960 (blue), 2013–2022 (red), and change in the percentage land area affected from 1951–1960 to 2013–2022 (teal) by: (A) WHO region; (B) *Lancet* Countdown region; and (C) HDI country group.

Indicator 1.2.3: Extreme weather and Sentiment

Indicator authors

Dr Kelton Minor, Dr Nick Obradovich

Methods

The data source for this indicator has been updated for the 2023 report.

The indicator is based on a dataset consisting of billions of social media posts, representing nearly all globally geo-localised tweets (within the daily volume limits of the full Twitter Firehose) from 2015–2022. The geo-tagged tweets constitute approximately two percent of all tweets, and thus may be somewhat limited in their generalisability due to opt-in geo-localisation. That said, consistent functional responses to meteorological variables have been uncovered across social media platforms, including massive samples of status posts from Facebook, Chinese Weibo (Twitter-style) posts, and Twitter geo-located data from multiple countries.[66–69] There appears to be little reason to suspect that the Twitter data are substantially biased from the overall relationship between climatic variables and emotional expressions. The functional relationships are nearly identical across platform and location and the estimated effects of heat are consistent with those uncovered in a high frequency national survey study[70].

The analysis for this indicator followed the methodological approach employed in multiple peer-reviewed publications.[66–68,71–73] Climate econometric methods were employed[74,75] to track the causal relationship between observationally measured sentiment expressions and exposure to varying ambient heat and precipitation extremes.

The social media data consisted of more than 8.2 billion geolocated tweets collected via the Amazon Web Services servers from the Twitter Streaming API between 2015 and 2022. These tweets spanned the globe, with a median number of unique active daily users of approximately 900,000. Activity was highest in more populous and wealthier countries, though Twitter use continues to expand globally (Figure 48).

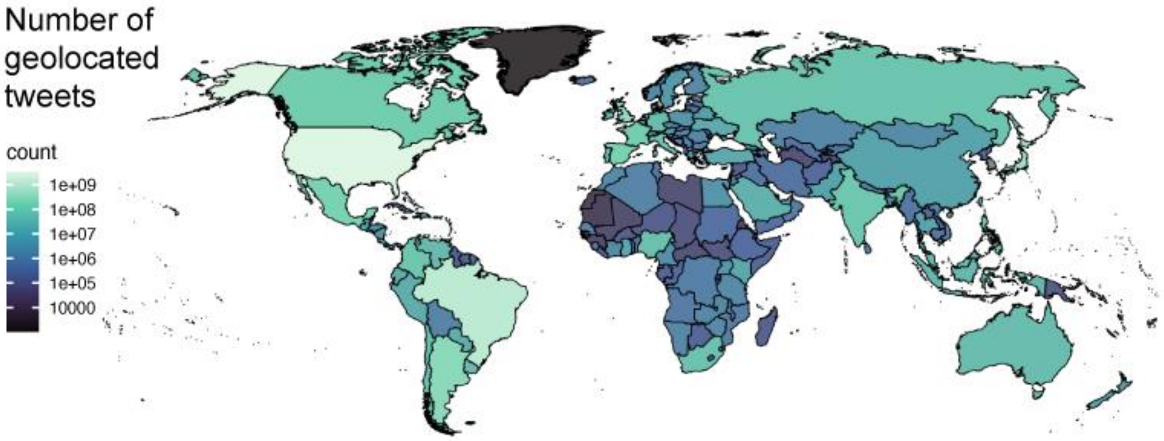

Figure 48: Country-level count of geolocated tweets, 2015–2022. Data includes posts from over 190 countries and ~44000 administrative-2 divisions (ex. counties).

The positive and negative valence[76] of each of the Twitter posts was classified using the Linguistic Inquiry Word Count (LIWC) sentiment classification tool[77–79] across thirteen available languages: Dutch, English, French, German, Italian, Japanese, Mandarin, Portuguese, Romanian, Russian, Serbian, Spanish, and Ukrainian which provided broad geographic coverage for the sample. Table 19 presents the by-language breakdown in the distribution of tweets from the 2022 indicator. Tweets with a 'lang' field matching each respective language are classified using that language's dictionary.

Table 19: By-language breakdown in the distribution of collected tweets.

Included language and LIWC dictionary	% of total geolocated tweets in data 2015–2022

Dutch	0.51%
English	65.25%
French	1.94%
German	0.51%
Italian	0.84%
Japanese	5.94%
Mandarin	0.17%
Portuguese	12.11%
Romanian	0.07%
Russian	1.15%
Serbian	0.02%
Spanish	11.44%
Ukrainian	0.06%

LIWC is one of the most highly validated psychometric sentiment classification tools and has been employed in multiple studies on the relationship between climatic variables and online emotional expressions.[77,78,80–83] Further, the effects observed via the LIWC classifier have also been observed via the use of alternative classifiers in both the U.S.[66] and Chinese[68] context.

To enable the analysis that underpins this indicator, geolocated social media posts were geo-spatiotemporally matched with daily 30km gridded ECMWF ERA5 reanalysis ambient 2m air temperature data,[84] precipitation totals, and meteorological controls at the 2nd-administrative level (GADM version 3.6). This ECMWF product provides globally consistent spatial and temporal coverage. Daily 30km gridded meteorological data were employed from the ECMWF ERA5 reanalysis product from 2015 to 2022. Heatwave metrics were calculated employing the methods used by indicator 1.1.2. Further, measures of r99p extreme daily precipitation (>99th percentile precipitation for a given location during the recent historical record using the same 1986–2005 climate normal used in The *Lancet* Countdown's heatwave definition), cloud cover, relative humidity, diurnal temperature range and wind speed were incorporated from the ERA5 data. The r99p "extremely wet day" threshold is an established climatological index for extreme precipitation events and has been widely used to track global increases in extreme precipitation over land in recent decades.[85–88] To aggregate the meteorological variables, weather timeseries were extracted from the gridded ERA5 raster data at the second administrative division-resolution for each day in the data.

The primary spatial unit of analysis for the statistical investigation was the second administrative division-level (ex. county-level). The temporal unit of analysis was the calendar date, resulting in second-administrative-unit-by-day-of-observation analyses.

To aggregate the sentiment measures to this unit of analysis, procedures previously described were followed.[66] Namely, for both positive and negative sentiment, each tweet was coded as either zero if the tweet contained no matching sentiment terms or one if it contained terms that match the corresponding sentiment. A tweet can express both positive and negative sentiment, only one of the two, or neither. For each day in the data, the average positive sentiment and the average negative sentiment was calculated for each unique user on that day, multiplying by 100 to produce a percentage. Users' scores were then averaged within the same second-division administrative unit together to produce the daily administrative sentiment measures. These measures ranged between 0 and 100.

Models drawn from climate econometrics were employed to estimate the effect of exposure to heatwaves on positive and negative sentiment; modelling the dependent variables as positive and negative sentiment, respectively, the primary independent variable an indicator of whether an administrative-unit-day was experiencing a heatwave. The model additionally included an indicator variable for whether a location was experiencing an extremely (>99th percentile) wet day, and controls for other meteorological conditions. To control for potentially confounding factors that may vary over time across different locations calendar-month-by-2nd-administrative region fixed effects were included in the models. Calendar date (ex. "2019-11-01", "2020-11-01") fixed effects for each unique date of observation was also included to account for idiosyncratic day-specific effects and global trends in internet and social media use.[69,75,89–92]

The multivariate fixed effects model estimated largely replicated that estimated in Baylis et al[66] and is as follows:

$$Y_{jmt} = \beta HEAT_{jmt} + \delta HPRCP_{jmt} + \mathrm{h}(\mu) + \gamma_t + \nu_{jm} + \epsilon_{jmt}$$

Here j indexed 2nd-level administrative region units, m indexed unique calendar months, and t indexed unique calendar dates. Y_{jmt} represented the dependent variables of positive and negative sentiment rates, respectively, $HEAT_{jmt}$ represented the binary heatwave indicator, which equals one if the date is classified as a heatwave in location m and equals zero otherwise. $HPRCP$ represented the extreme precipitation indicator. β was the main coefficient of interest, the effect of a heatwave on positive and negative sentiment rates in percentage points. δ was the secondary coefficient of interest, the effect of an extreme precipitation event on sentiment rates, $\mathrm{h}(\mu)$represented the meteorological controls, which included 20 percentage point percentile-bin controls for the temperature observations (with the omitted category of the 40th-60th temperature percentile bin serving as the omitted reference category for $HEAT_{jmt}$). $\mathrm{h}(\mu)$ also included flexibly binned control variables for cloud cover percentages, relative humidity, and wind speed.

Further, γ_t represented date-specific fixed effects that controlled for any idiosyncratic shocks in the data as well as factors that trended similarly over time across all locations. ν_{jm}indicated second-administrative-unit-by-calendar-month fixed effects that controlled for any location-specific seasonal and secular trends that might confound inference. ϵ_{jmt} represented the error term. Based on methodology in Baylis et al.,[66] errors on administrative-unit-by-month and date were clustered and the regressions by the number of unique twitter posts in each administrative-unit-day and estimated the model for each year within the data were weighted, giving a β for each year presented.

Lastly, an exploratory subgroup analysis across human development groups by stratifying the global Twitter data according to the UN's Human Development Index (HDI) was conducted. The data were grouped into "high development" countries (operationalized as "very high" and "high" HDI countries) and "developing" country contexts ("medium" and "low" HDI countries), following the HDI-defined classifications,[93] and employed the same model specification as above on the two subgroups. Similar stratified subgroup analyses were conducted for each of the WHO geographic regions and Lancet Countdown regions (Appendix Figure X4).

Data

1. Climate data from the European Centre for Medium-Range Weather Forecasts (ECMWF) ERA5 reanalysis.[94]
2. Geolocated tweets collected via the Twitter Streaming API.

Caveats

Although this indicator has many inferential strengths, particularly as compared to existing survey-based and surveillance-based methods, it is neither a perfect nor exhaustive measure of the subclinical mental health burden of heatwaves and weather extremes.

Countries that did not have Twitter broadly available to the public—such as China—were underrepresented in the indicator, despite the addition of Mandarin tweets this year. Second, geo-tagged tweets constitute approximately two percent of all tweets and thus may be somewhat limited in their generalisability due to opt-in geo-localisation. However very similar effects have been consistently documented across social media platforms, including massive multi-country samples of status posts from Facebook, Chinese Weibo (Twitter-style) posts, and Twitter geo-located data.[66,68,73] There appears to be little reason to suspect that the Twitter data are substantially biased from the overall relationship between climatic variables and emotional expressions. The functional relationships are nearly identical across platform and location.

Third, since higher income populations likely have greater access to adaptive amenities (air conditioning, etc.), the estimates produced by the identification strategy may be conservative (biased towards zero) for those disproportionately exposed to some of the hottest conditions in poorer socioeconomic contexts. However, a recent national analysis in China[68] suggests similar functional response forms across socioeconomic contexts, with very similar magnitudes observed for extreme heat-related responses, suggesting that added income may only smooth the relationship to a more moderate degree, and primarily for cold temperatures rather than warm ones.

Future form of the indicator

Global internet use and social media connectivity are expected to continue to increase over the coming decade, likely further expanding the global reach and coverage of the sample. Whilst the focus of this current version of the indicator is on sentiment responses to heatwaves and precipitation extremes, future iterations can expand to cover expressed responses to additional climate-related environmental stressors, including floods, hurricanes/cyclones/typhoons, fires, and smoke. Mirroring the approach taken with heatwaves in the current

indicator, these extreme events can be registered using standard definitions, including those specified directly by The *Lancet* Countdown in future annual reports.

Additional analysis

From 2015–2022, both heatwaves and extreme precipitation days consistently worsened human sentiment, with the slopes of fitted trend lines indicating increasing effect sizes over the eight-year period for all outcomes (Figure 49). Over the past eight years, the impact of heatwaves in amplifying negative sentiment expression increased at a rate of an extra 0·23 percentage points per decade, while their impact in reducing positive sentiment is increasing by an additional 0·21 percentage points per decade. The impact of days with extreme precipitation in reducing positive sentiment rose by an extra 0·23 percentage points per decade, while their impact on negative sentiment remained nearly unchanged. The average effect of a heatwave in 2022 on reduced positive sentiment was 44% larger than the impact of losing an hour due to the spring daylight savings time transition (DST), whereas the effect of a heatwave on increased negative sentiment was 5·32 times the DST impact.

Adopting the extreme event analysis approach introduced in the 2022 report, this year's indicator assesses the sentiment impacts of specific extreme climate events that underwent attribution analyses by the Worldwide Weather Attribution initiative (Figure 50).[95,96] For 2022, the impacts of both the April 2022 East South Africa Extreme Rainfall event and July 2022 UK Heatwave were examined, both which were found to have been made more likely due to human-induced climate change[95,96]. Analysing geolocated tweets that originated from these regions during these extremes shows that the East South Africa Extreme Rainfall event significantly and substantially amplified negative sentiment by 2·09 percentage points, the largest negative sentiment impact yet recorded by the indicator. This event also severely lowered the share of positive sentiment tweets by 1·63 percentage points. The UK Heatwave significantly reduced the share of tweets expressing positive sentiment by 1·94 percentage points, the largest reduction in positive sentiment registered thus far by this indicator. This same heatwave elevated negative sentiment by 0·17 percentage points – nearly twice the average impact of a heatwave observed between 2015-2022 – although this increase was not statistically significant.

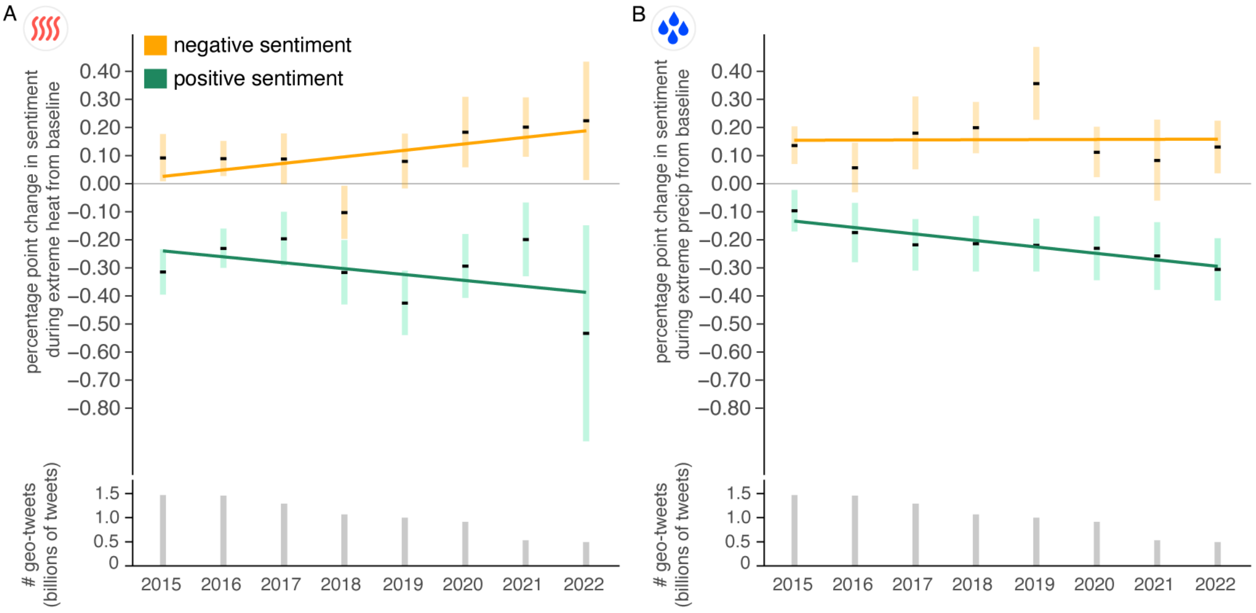

Figure 49: (A) Effect of heatwave exposure on positive (green) and negative (orange) sentiment expressions, derived from the textual expressions of over 8.2 billion Twitter posts. Intervals depict 95% CIs of the estimated average percentage point change in the share of sentiment expressions during days with heatwaves compared to control days without heatwaves in the same place, month, and year. Coloured lines depict the 2015-2022 fitted trends tracking the change in the impact of extreme weather on positive (green) and negative (orange) sentiments. Lines sloping away from the grey "zero" line indicate increasing impacts over time (B) Effect of exposure to extremely wet (>99th percentile daily precipitation) days on expressed sentiments compared to local daily average precipitation. Grey bars depict the geolocated Tweet count by year of record.

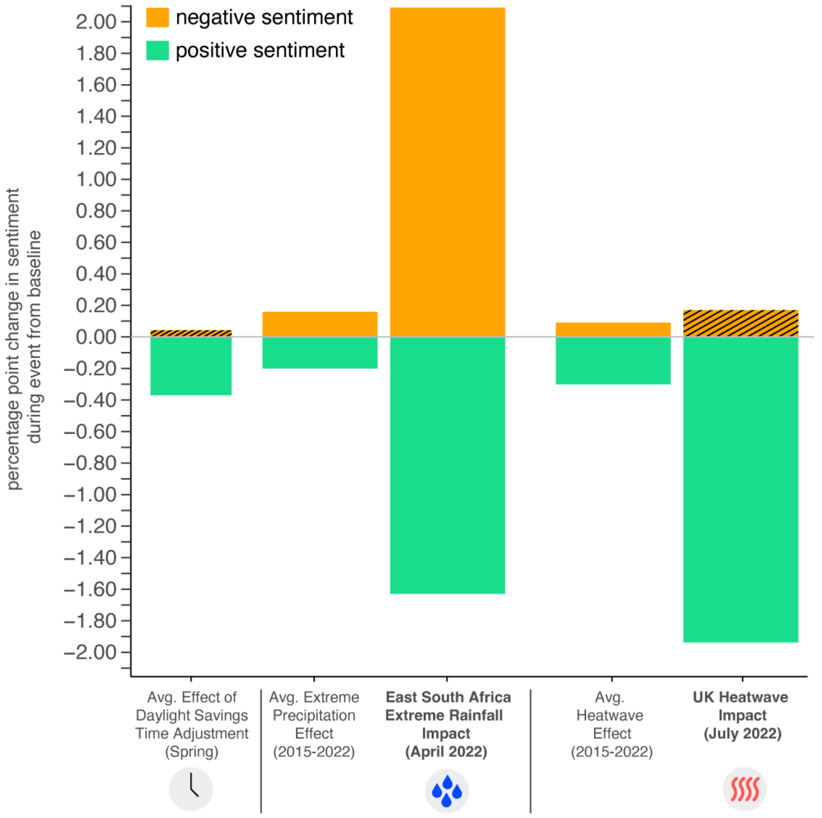

Figure 50: Estimated sentiment effects of the 2022 Eastern South Africa extreme rainfall event and July UK heatwave on the percentage of positive (green) and negative (orange) sentiment expressions on Twitter. For comparison, sentiment effect sizes are also shown for the impact of the spring daylight savings time change, average 2015–2022 extreme precipitation response, and average 2015–2022 heatwave impact. Solid bars without stripes indicate response estimates that were also statistically significant at the p <·05 level.

Figure 51 shows the estimated sentiment responses for very high-high UN Human Development Index (HDI) countries, and medium-low HDI countries. Heatwaves increased negative sentiment twice as much in medium-low HDI countries (0·16 percentage points) compared to very high-high HDI countries (0·08 percentage points), but only significantly reduced positive sentiment in very high-high HDI countries (-0·31 percentage points). Heatwaves only slightly reduced positive sentiment (-0·01 percentage points) in medium-low HDI countries, although the confidence interval contained zero.

Extreme precipitation significantly reduced positive sentiment in both very high-high HDI countries (-0·20 percentage points) and medium-low HDI countries (-0·17 percentage points). By contrast, extreme precipitation only significantly elevated online negative sentiment expressions in very high-high HDI countries (0·16 percentage points), but not medium-low HDI countries (0·04 percentage points). See the appendix of the 2021 report for additional analyses and discussion about differential responses across HDI contexts[71].

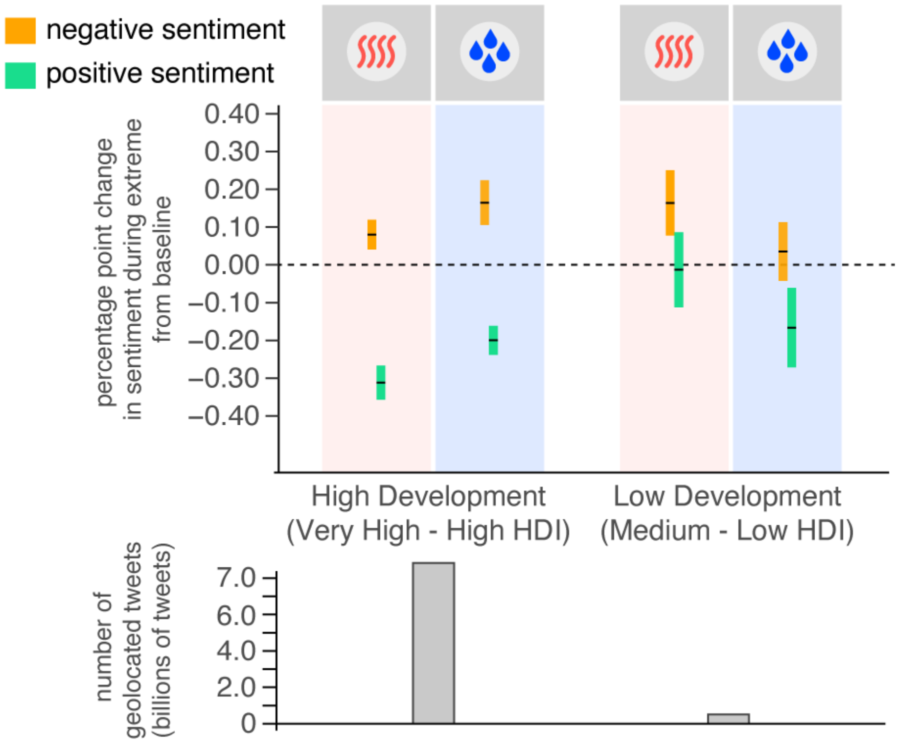

Figure 51: Average impacts of extreme heat and extreme precipitation exposure on positive and negative sentiment expressions by Human Development Index groups from 2015-2022. Bottom grey bars plot the

This year's indicator also features an investigation of the responses to extreme heat and precipitation across global geographic regions using the *Lancet* Countdown's regional groupings (Figure 52). Responses for WHO regions are additionally reported as supplementary data. *Lancet* Countdown regional estimates indicate that heatwaves increased the share of negative sentiment Twitter posts for all regions except Europe during the 2015–2022 period, with statistically significant negative sentiment elevations observed in Africa and North America. In order of effect magnitude, heatwaves increased negative sentiment by 0·13 percentage points in Africa, by 0·10 percentage points in Oceania, by 0·09 percentage points in North America, by 0·06 percentage points in Asia, by 0·04 percentage points in South Central America, and by 0·02 percentage points in SIDS (Small Island Developing States). Conversely, heatwaves were associated with a 0·06 percentage point reduction in negative sentiment in Europe, but this effect was not statistically significant.

Heatwaves also reduced the percentage of tweets with positive sentiment expressions for all regions, with statistically significant positive sentiment attenuation evident in all regions except for Africa, SIDS, and Europe. In descending order of effect size, heatwaves reduced positive sentiment by 0·36 percentage points in South and Central America, by 0·23 percentage points in Asia, by 0·20 percentage points in Oceania, by 0·11 percentage points in North America, by 0·09 percentage points in Africa, by 0·03 percentage points in SIDS, and by 0·01 percentage points in Europe.

Extreme precipitation days increased negative sentiment for all regions except for South Central America, with statistically significant negative sentiment elevation evident in Asia, Oceania, SIDS, North America, and Europe. In order of effect size, extreme precipitation increased the share of tweets with negative sentiment by 0·49 percentage points in Asia, 0·27 percentage points in Oceania, 0·17 percentage points in SIDS, 0·14 percentage points in North America, 0·14 percentage points in Europe, and by 0·03 percentage points in Africa. By contrast, extreme precipitation slightly reduced negative sentiment by 0·01 percentage points in South Central America, although this reduction was not statistically significant.

Extremely wet days also reduced the share of tweets with positive sentiment expressions across all geographic regions, with statistically significant reductions detected in North America, Africa, South Central America, Asia, and Europe. In order of magnitude, extreme precipitation reduced positive sentiment by 0·26 percentage points in North America, 0·22 percentage points in Oceania, 0·21 percentage points in Africa, 0·15 percentage points in South and Central America, 0·14 percentage points in Asia, 0·13 percentage points in SIDS, and by 0·10

percentage points in Europe. While globally extensive, Twitter coverage is not equally distributed (Figure 48) limiting the precision of estimates in regions with sparse coverage.

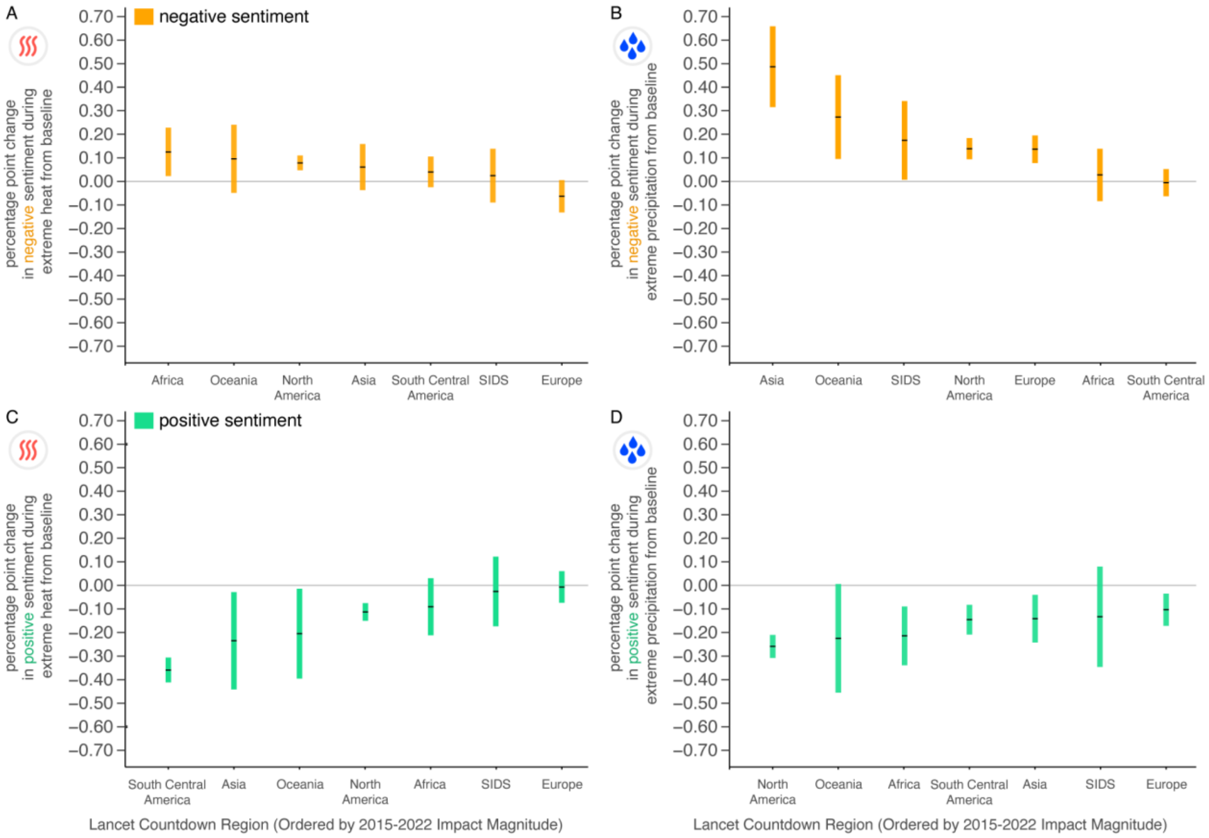

Figure 52: Effects of extreme weather on the shares of positive and negative sentiments of geolocated Twitter expressions from 2015–2022, stratified by LC geographic regions. (A, C) The estimated average effects of a heatwave day during the 2015–2022 observation period on negative sentiment (orange) and positive sentiment (green) responses for each region, ordered by impact magnitude. Intervals depict 95% CIs of the estimated average percentage point change in the share of sentiment expressions during days with extremes compared to the meteorological baseline for each location. (B, D) The estimated regional sentiment responses to extremely wet precipitation days.

Indicator 1.3: Climate Suitability for Infectious Disease Transmission

West Nile virus

Indicator authors

Julian Heidecke, Prof Joacim Rocklöv, Dr Marina Treskova

Methods

This is the first year this indicator has been included in a *Lancet* Countdown report.

The global West Nile virus (WNV) indicator tracks the thermal suitability for the transmission of WNV and changes in its basic reproduction number driven by climate change.

WNV is a mosquito-transmitted pathogen that can cause severe disease with central nervous system involvement in birds, humans, and other mammals. As of today, there are no pharmaceutical prevention or treatment options for WNV infection in human hosts. WNV is transmitted primarily by the bite of infected mosquitoes that acquire the virus from infected birds.[97] Mosquito vectors of WNV, mainly from the genus *Culex* (*Cx.*), are widely distributed, allowing WNV to occur almost globally. Over the past two decades, Europe has experienced an expansion of the geographical range and intensity of WNV transmission marked by unprecedented outbreaks in 2010 and 2018. The 2018 outbreak was characterised by 2,083 reported locally acquired infections in humans representing a 7.2-fold increase compared to the previous year.[98] In the USA, since the first invasion of WNV into the country in 1999, more than 51,000 human clinical cases have been reported, including over 2,300 deaths.[99] Due to various direct and indirect effects of environmental conditions on the WNV-vector-host nexus, climate change impacts WNV transmission in multifaceted ways.[100] Ambient temperature has been recognized as one of the major drivers of WNV transmission in Europe and the USA.[101–103] For other parts of the world (Central/South America, Africa, Asia, and Australia), the evidence is scarce, likely due to underreporting. It stands to reason, however, that global warming also affects WNV transmission patterns in these regions.[104]

Spatial-temporal patterns of WNV epidemiology can be explored using mathematical models. Related literature includes mechanistic models approximating the temperature dependence of the basic reproduction number $\boldsymbol{R_0}$, i.e., the expected number of secondary host infections arising from a single infected host in a completely susceptible population, or of related transmission metrics. Although these suitability metrics only consider specific aspects of WNV transmission, they are based on well-studied relationships and able to explain geographical and seasonal patterns of WNV transmission to a certain extent.[101,105,106] The equation below represents a model for temperature dependence of the basic reproduction number. Here, the temperature $(\boldsymbol{T})$ dependent $\boldsymbol{R_0}$ is modelled as a Ross-Macdonald type expression as follows:[107]

$$\boldsymbol{R_0(T) = \frac{m(T)a(T)^2 b_m(T) p(T)^{n(T)} b_h}{-\ln\big(p(T)\big) r_h}}$$

which includes the temperature dependent biting rate $\boldsymbol{a(T)}$, the vector competence $\boldsymbol{b_m(T)}$, daily mosquito survival probability $\boldsymbol{p(T)}$ (given by $\boldsymbol{e^{-\mu_M(T)}}$, where $\boldsymbol{\mu_M(T)}$ is the mosquito mortality rate), the length of the extrinsic incubation period $\boldsymbol{n(T)}$ (the inverse of the pathogen development rate), the mosquito to host ratio $\boldsymbol{m(T) = M(T)/N}$, as well as the host infection probability $\boldsymbol{b_h}$, and host recovery rate $\boldsymbol{r_h}$. Using laboratory data measured at constant temperatures, Shocket et al. applied Bayesian inference to fit temperature response functions for several vector-pathogen traits and vector-virus pairs.[101] This indicator utilises the response functions derived for *Cx. pipiens*, *Cx. tarsalis*, and *Cx. quinquefasciatus* because they are well-studied key WNV vectors and were reported as having good data availability for most of their traits. Together, these species inhabit a vast geographical area globally.[101] For this first iteration, the indicator uses the median function coefficients resulting from the fitting procedure by Shocket et al. to parametrize the temperature responses for each vector-pathogen trait (note: notation of parameters is changed from Shocket et al.) and thus for $\boldsymbol{R_0(T)}$.[101]

To account for the impact of temperature-dependent mosquito life history traits on mosquito abundance, a proxy of $\boldsymbol{M(T)}$ was derived utilising the mosquito traits included in Shocket et al.[101] To this end, a simplified version of the stage-structured mosquito population dynamics model by DiSerra et al. was considered (adapted to the traits studied by Shocket et al.).[108] The corresponding differential equations read as follows:

$$\boldsymbol{\dot{J} = \beta(T)M - \left(1 + \frac{J}{K}\right)\mu_J(T)J - \gamma(T)J}$$
$$\boldsymbol{\dot{M} = \omega\gamma(T)J - \mu_M(T)M}$$

where $\boldsymbol{J}$ denotes the juvenile aquatic phase of the mosquito life cycle (i.e., encompasses eggs, larvae, and pupae) and $\boldsymbol{M}$ represents the adult female mosquito population. The incorporated temperature dependent parameters are defined as: $\boldsymbol{\beta(T)}$ denotes the oviposition rate (eggs laid per female mosquito per day), $\boldsymbol{\mu_J(T)}$ is the juvenile mortality rate (at low juvenile density), $\boldsymbol{\gamma(T)}$ denotes the mosquito development rate, and $\boldsymbol{\mu_M(T)}$ is the adult mortality rate as before. In addition, the model includes the proportion of female mosquitoes at adult emergence (here assumed as 0.5), and a juvenile carrying capacity parameter $\boldsymbol{K}$. To prevent unlimited population growth, the model assumes a density-dependent increase in mortality arising from competition in the juvenile stage similar as in the model by DiSerra et al.[108] The juvenile mortality rate $\boldsymbol{\mu_J(T)}$ was calibrated such that at low juvenile density $(\boldsymbol{J \approx 0})$, the proportion of juveniles surviving to the adult stage, given by $\boldsymbol{\gamma(T)/(\gamma(T) + \mu_J(T))}$, corresponds

with the egg-to-adult survival probability $\boldsymbol{p_{EA}(T)}$ in Shocket et al.[101] The population equilibrium was evaluated as a proxy for mosquito abundance in the $\boldsymbol{R_0}$ model following the simplifying assumption that at the point of introduction of WNV the modelled populations are at demographic equilibrium (essentially assuming a static environment in accordance to the lab data measured at constant temperatures). The temperature-dependent adult population equilibrium is given by:

$$\boldsymbol{M(T) = K\frac{\omega^2\beta(T)\gamma(T)^2}{\mu_M(T)^2\mu_J(T)}\left(1 - \frac{\mu_M(T)}{\omega\beta(T)p_{EA}(T)}\right)}$$

with $\boldsymbol{M(T) = 0}$ if the population reproduction number $\boldsymbol{\omega\beta(T)p_{EA}(T)/\mu_M(T)}$ is less than or equal to one. The juvenile carrying capacity parameter $\boldsymbol{K}$ depends on a multitude of factors such as the availability of mosquito breeding habitat (determined by land cover, precipitation patterns, and human water storing behaviour). By excluding $\boldsymbol{K}$ from the expression of $\boldsymbol{M(T)}$, a dimensionless proxy of relative mosquito abundance is derived that isolates the impact of temperature via the mosquito life-history traits. This allows us to consider these additional relationships when measuring thermal suitability for transmission on a global scale.

Since the host recovery rate $\boldsymbol{r_h}$, the host infection probability $\boldsymbol{b_h}$, and the host density $\boldsymbol{N}$, are not temperature dependent (and hard to estimate given the numerous host species involved in WNV transmission), they were removed from the formulation of $\boldsymbol{R_0}$. All the remaining parameters are species-specific temperature dependent vector-pathogen traits. Due to the removal of host density, recovery rate, and host susceptibility, in addition to neglecting factors like mosquito host preference, mosquito habitat availability, host movements, and vector control efforts, the resulting relative $\boldsymbol{R_0}$ cannot be interpreted as an absolute measure of secondary cases (i.e., the basic reproduction number) but as a relative measure of the transmission risk space with an upper and lower thermal limit for transmission and an optimum, all of which are mosquito species-specific. To emphasise the interpretation of the relative $\boldsymbol{R_0}$ as a transmission suitability index, it was rescaled to a value ranging from zero to one by dividing by the value reached at optimal temperature. The strong and nonlinear relationships of the vector-pathogen traits to temperature result in a unimodal response of $\boldsymbol{R_0}$ to temperature with transmission potential peaking between 23°C and 26°C and thermal limits and optima for transmission varying between vector species.

Relative $\boldsymbol{R_0}$ was computed at 0.1x0.1° spatial resolution using the ERA5-Land temperature data.[109–111] To account for the species-specific responses to temperature, the mosquito distribution maps in Shocket et al. were georeferenced and used to determine the distribution of each species.[101] The combined global indicator was calculated by combining the vectors' distribution ranges and applying each vectors' $\boldsymbol{R_0}$ in their respective range. In areas where *Cx. pipiens* and *Cx. quinquefasciatus* overlap in the Americas, Asia, and Australia, their relative $\boldsymbol{R_0}$ temperature responses were averaged. Since there is substantial introgression and hybridization between the species in these regions, it was assumed that the temperature response of the hybrid populations falls "in between" the responses of the two species. For the overlapping regions in Africa (where *Cx. pipiens* and *Cx. quinquefasciatus* do not hybridise and are widely found at different elevations) as well as the overlapping regions with *Cx. tarsalis* (which has different habitat type than the other two species), the maximum out of the relative $\boldsymbol{R_0}$'s was taken. This was based on the simplifying assumption that the species with the highest transmission suitability would dominate disease spread. The gridded $\boldsymbol{R_0}$ were extracted and aggregated by Country, WHO regions, LC groupings, and human development index (HDI).

Data

1. Monthly temperature data from the European Centre for Medium-Range Weather Forecasts (ECMWF) ERA5-Land reanalysis.[112]
2. Thermal response data: Data from Shocket et al. showing thermal response of vector-pathogen traits fed into a relative R_0 model for specific WNV-mosquito species combinations.[101]
3. Vector species distribution maps shown in Shocket et al.[101]

Caveats

Although the indicator considers three key WNV mosquito species that enable good spatial coverage, some regionally important species (e.g., *Cx. modestus* in Europe or *Cx. annulirostris* in Australia) are not included. Therefore, only the transmission suitability of the considered species is measured, and only in the regions where they are present (see, however, the additional analysis where the individual species' relative $\boldsymbol{R_0}$ were applied to the global temperature data). In addition, potential intraspecific differences between the thermal response of vector

populations from different areas were not accounted for. While global warming is the best documented climatic impact on WNV transmission, other changes in climatic conditions, such as altered precipitation patterns and more frequent droughts, are also affecting local conditions for WNV mosquitoes and transmission but have not yet been included in the indicator. The vector distribution in the indicator is based on the current knowledge of their distributions and does not track potential shifts due to climate change. Moreover, the indicator does not include WNV host aspects such as distribution, abundance, composition, and movement, which are also affected by climate change. Thus, the indicator can only be interpreted as a relative measure of transmission suitability isolating warming impacts on vector-pathogen traits, but it cannot be interpreted as a threshold parameter for outbreaks or an absolute measure of secondary infections such as the classical basic reproduction number. Therefore, the percentage change in the relative $\boldsymbol{R_0}$ should not be confused with a specific change in the number of WNV cases or outbreaks, although it indicates whether the risk increases or decreases. Additionally, the indicator does not consider whether WNV is present in a certain area, such as the Americas before 1999. Nonetheless, measuring the climate suitability of WNV in these areas is still informative about the change in the risk of introduction and establishment of the virus, and it is thus still deemed informative of disease emergence. In its current version the indicator utilises monthly averaged temperature data. Temperature variations on finer temporal scales are not accounted for.

Future form of the indicator

Future versions of the indicator will seek to address the caveats listed above. In particular, the aim will be to improve the mosquito population approximation, potentially considering factors beyond temperature such as breeding habitat availability. A more complete description of the environmental conditions favourable for the WNV vectors could allow to track changes in modelled vector distributions, eliminating the necessity to include static distribution maps from the literature. The thermal response of vector-pathogen traits will be updated should additional data become available. Finer resolution temperature data will be considered, including the incorporation of within month and daily temperature fluctuations. Furthermore, work will be put towards estimating changes in the basic reproduction number in absolute values by incorporating avian host abundance estimates and trends.

Additional analysis

The results of the global (combined mosquito ranges) indicator and the breakdown by HDI country groups are shown in Figure 53. In addition to the global combined indicator, changes in thermal suitability as given by the individual species-specific $\boldsymbol{R_0}$ were considered applying them to the global temperature data as well as restricting the calculations to species' distribution (Figure 54). Thermal suitability for transmission by *Cx. pipiens* and *Cx. tarsalis* decreased on a global scale from 1951-1960 to 2013-2022. However, there was an increase by 7.1% for *Cx. pipiens* and 11.9% for *Cx. tarsalis* when restricting the calculations to the vectors' distribution range, underlining the escalating impact of global warming on WNV risk in these areas. Having a higher thermal optimum, transmission suitability for *Cx. quinquefasciatus* increased both globally and in the vectors' range, although the trend has been levelling off since the early 2000s. The combined indicator was further calculated by WHO regions and by LC groupings (Figure 55, Figure 56). The Eastern Mediterranean WHO region has experienced a decrease in thermal suitability for WNV transmission (-8.1%) from 1951-1960 to 2013-2022, while all other regions have experienced increases in the same period varying between regions: 3.2% for Africa, 7.3% for the Americas, 18.3% for Europe, 2.9% for South-East Asia, and 4.3% in the Western Pacific WHO region. Similar trends can be seen when considering LC groupings. Here, Oceania is the only group showing a decrease from 1951-1960 to 2013-2022 (-2.9%) while Europe (+36.3%) and Northern America (+14.4%) present the most notable percentage increase in WNV relative $\boldsymbol{R_0}$. It should be noted that the high magnitude of percentage increase in these regions has to be interpreted with caution since these regions were also the ones having the lowest baseline relative $\boldsymbol{R_0}$ values in the reference period (0.05 for Europe and 0.17 for Northern America). Nonetheless, the steep percentage change observed for Europe is in line with the expansion and increasing trend in the number of cases that has been observed in this region over the last two decades. Changes in the seasonality of the combined indicator and for each of the three mosquito species (restricted to distribution ranges) were additionally considered (Figure 57). While relative $\boldsymbol{R_0}$ exhibits a unimodal seasonality in the ranges of *Cx. pipiens* and *Cx. tarsalis*, peaking around July and August, transmission potential by *Cx. quinquefasciatus* shows two peaks within the year but stays somewhat stable over the year (mind the different scaling of the axes), in accordance with the more tropical distribution of this species. The combined indicator peaks around August. It should be noted that these values were calculated on the total mosquito ranges and seasonality is expected to differ on a regional level.

Overall, transmission suitability during peak months has increased over the years (Figure 57 shows the relative $\boldsymbol{R_0}$ over the year for every second year in the period 1950-2022 for better visibility).

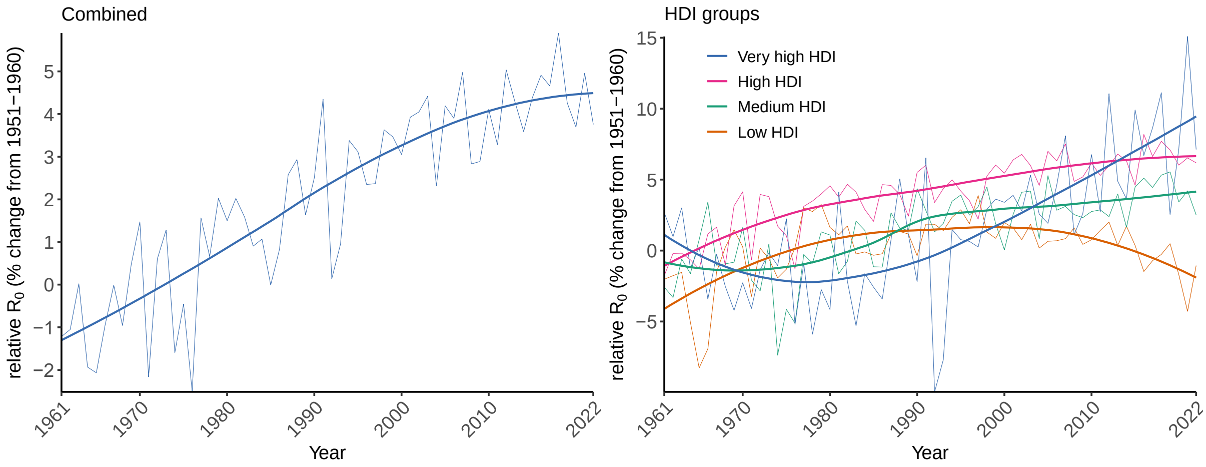

Figure 53: Percentage change in WNV relative $\boldsymbol{R_0}$ in the period 1960-2022 compared to 1951-1960 for the global combined indicator as well as by HDI country group.

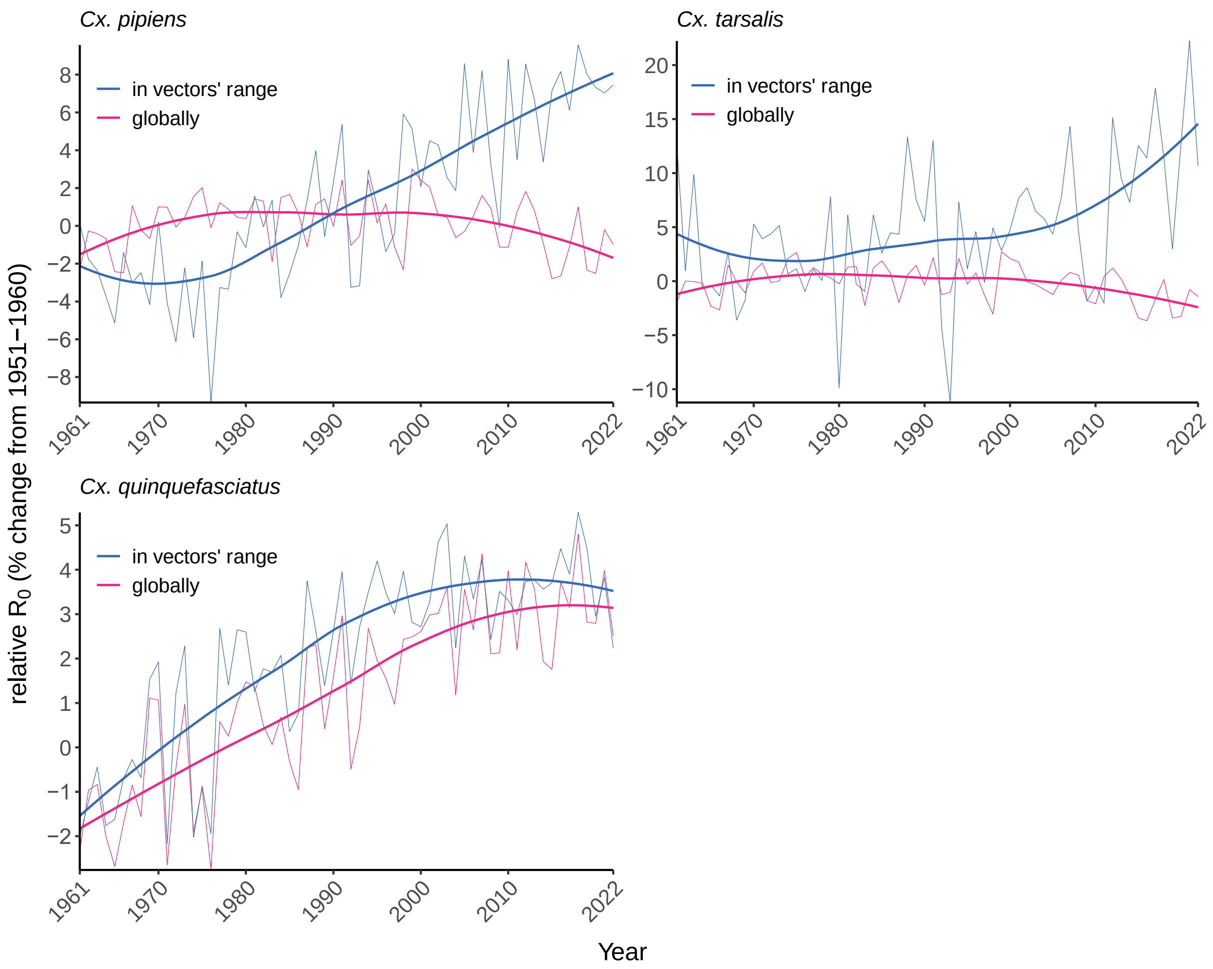

Figure 54: Percentage change in WNV relative R_0 in the period 1961-2022 compared to 1951-1960 for the species-specific R_0 of *Cx. pipiens*, *Cx. tarsalis*, and *Cx. quinquefasciatus* individually applied to the global temperature data as well as restricted to the species' distribution ranges.

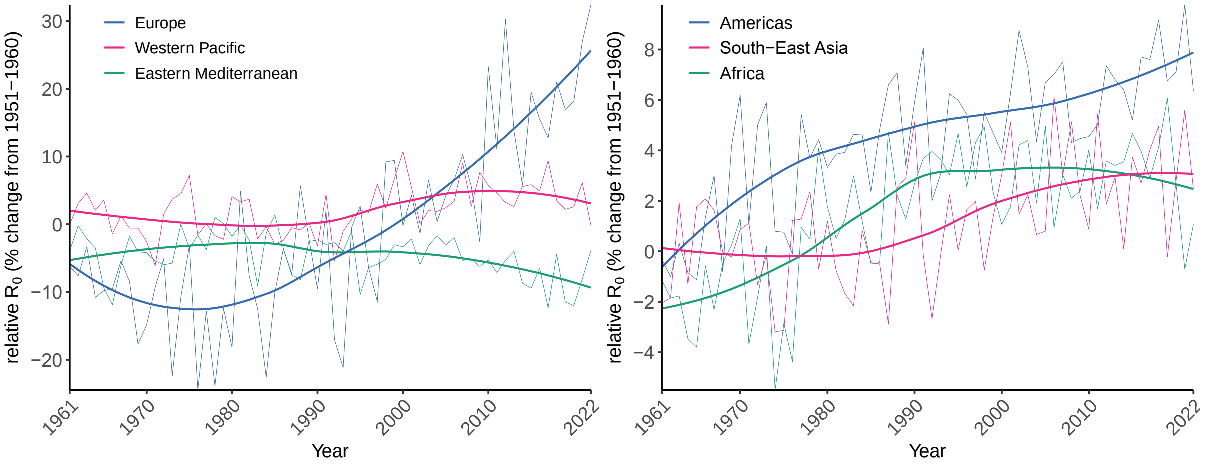

Figure 55: Percentage change in WNV relative R_0 in the period 1961-2022 compared to 1951-1960 by WHO region.

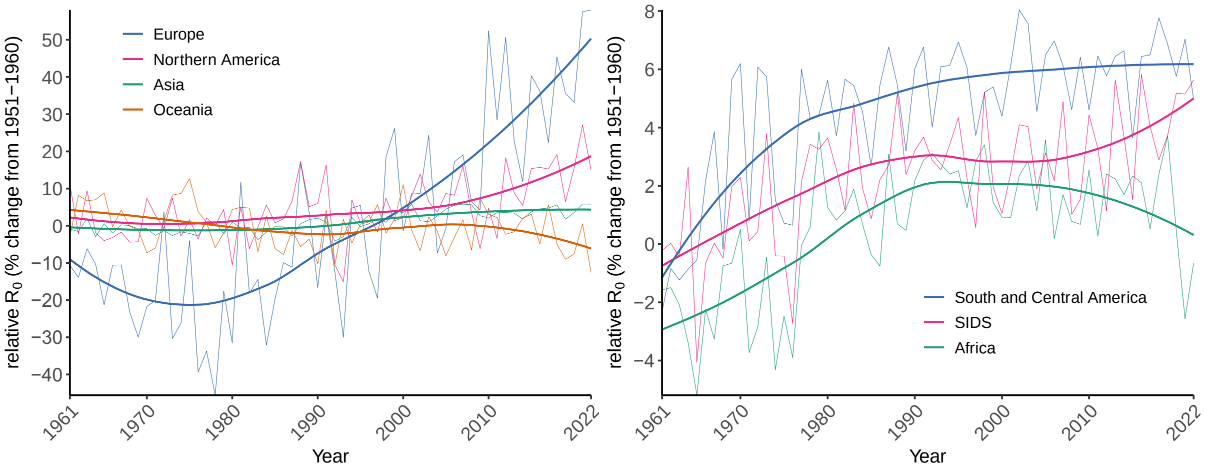

Figure 56: Percentage change in WNV relative R_0 in the period 1961-2022 compared to 1951-1960 by LC groupings.

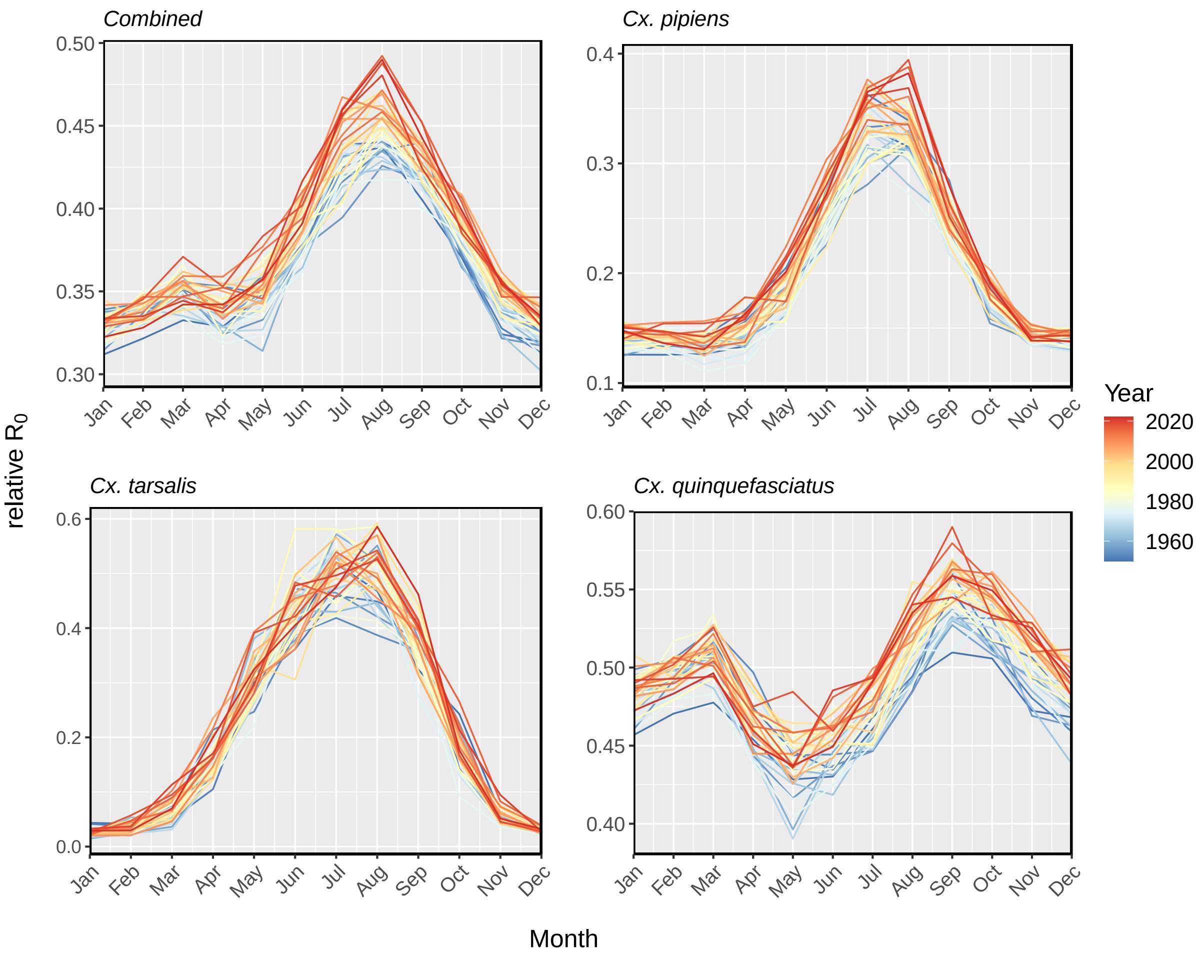

Figure 57: Change in seasonality of WNV relative R_0 in the period 1950-2022 for the global combined indicator as well as the species-specific R_0 of *Cx. pipiens*, *Cx. tarsalis*, and *Cx. quinquefasciatus* individually applied to the temperature data restricted to the species' distribution ranges. The relative R_0 over the year for every second year is shown for better visibility.

Dengue, Chikungunya and Zika

Indicator authors

Dr Maquins Odhiambo Sewe, Prof Joacim Rocklöv

Methods

The input data for this indicator have been extended for the 2023 report.

Cases of dengue have doubled every decade since 1990, with 58.4 million (23.6 million–121.9 million) apparent cases in 2013, accounting for over 10,000 deaths and 1.14 million (0.73 million–1.98 million) disability-adjusted life-years.[113] Beside global mobility, climate change has been suggested as one potential contributor to this increase in burden.[114] *Aedes aegypti* and *A. albopictus*, the principal vectors of dengue, also carry other important emerging or re-emerging arboviruses, including Yellow Fever, Chikungunya, Mayaro, and Zika viruses, and are likely to be similarly responsive to climate change.

R_0, i.e. the basic reproduction number, which is the expected number of secondary infections resulting from one single primary infected person case in a totally susceptible population ,was computed using the formula $R_0 = Vb_h/r_h$.[115] The vectorial capacity (V), which express the average daily reproductive rate of subsequent cases in a

susceptible population resulting from one infected case, was computed using the formula $V = ma^2 b_m p^n / -lnp$ where a is the average vector biting rate, b_m is probability of vector infection and transmission of virus to its saliva, n is the extrinsic incubation period while p is the daily survival probability. All these parameters are temperature dependent and are further described in the work by Rocklöv et al.[115–117]

The ratio between number of mosquitoes to the number of humans, is central to V and the R_0 value (*m)*, but often it is left out or estimated in a simple way. Here a model is used to estimate mosquito populations of *Aedes aegypti* and *Aedes albopictus* separately. The original mosquito-population models provide results in terms of the number of individuals of *Ae. aegypti* per breeding site (*X*), or the number of *Ae. albopictus* per hectare (*Y*). In order to appropriately estimate *m*, i.e. mosquito population density per human population density (*p*), *X* was multiplied by $f(p,a,c) = a * g(p,c)$ where *a* equals to the number of breeding-sites per human, and *Y* by $f(p,a/b,c) = a * g(p,c)/b$ where *b* equals the average number of breeding sites per hectare. The function $g(p,c) = p^2/(c^2 + p^2)$ is an increasing sigmoidal function that equals the viability of domesticated mosquito-populations in relation to human population density. Accordingly, $f(p,a,c)$ is the multiplicative factor m in V, which allowed to straightforwardly estimate correct values for *a, a/b* and *c* by fitting R_0 to R_0-data that was available for a subset of the spatiotemporal points.[118]

Numerically V and abundance estimates was computed at 0.5°x0.5° spatial resolution based on ERA5-Land[109] data resampled from the 0.1°x0.1° original resolution. V and vector abundance were run for both *Aedes* aegypti and *Aedes* albopictus vectors. Gridded population from HYDE 3.2 (History Database of the Global Environment) were used in the computation of R_0. For Dengue (albopictus) and Chikungunya, *Aedes albopictus* vector abundance estimates were used in the computation of *m* while for Dengue (aegypti) and Zika *Aedes aegypti* abundance estimates were used. Further annual length of transmission season (LTS) was computed by summing the number of months in a year when R_0 was greater than 1 following the work by Colón-González et al.[118]

The gridded R_0 and LST for Dengue (Aedes *aegypti),* Dengue (Aedes *albopictus),* Chikungunya (*Aedes albopictus*) and Zika (*Aedes aegypti*) were extracted and averaged by Country, WHO regions and according to human development index (HDI).

Data

1. Monthly climate data (2m air temperature, total precipitation) from the European Centre for Medium-Range Weather Forecasts (ECMWF) ERA5-Land reanalysis.[110,111]
2. Future climate projections from ISIMIP 3b protocol.[11]

Caveats

Key caveats and limitations of the V model and its parameterisation are fully described in works by Liu-Helmersson et al.[119,120] and Rocklöv et al.[115] The predicted R_0 should not be confused with actual dengue cases, although it is an indicator of the potential for outbreaks.[116,117]

Malaria

Indicator authors

Martín Lotto Batista, Prof Rachel Lowe

Methods

Malaria is widely recognised as a climate-sensitive infectious disease due to the climate sensitivity observed in both the vector, *Anopheles* mosquitoes, and the *Plasmodium* parasites.[121] Although there are five species within the *Plasmodium* genus, two of them are of major public health concern: *Plasmodium vivax* and *Plasmodium falciparum,* [122] with the latter being the dominant species around the world.

Temperature, precipitation, and relative humidity are climate factors that influence the abundance and feeding cycle rate of *Anopheles* mosquitoes, which transmit the *Plasmodium* parasites that cause malaria. Temperature also drives the development rate of *Plasmodium* parasites within the mosquito vectors. Temperatures within the range 18°C to 32°C are considered most suitable for *P. falciparum* parasites, while *P. vivax* requires temperatures

between 14.5ºC and 33ºC. Below this lower limit the development of the parasite ceases while in high temperatures the survival of the mosquito is compromised.[121,123] Additionally, relative humidity needs to be greater than 60% to allow mosquitoes to survive long enough to carry infectious parasites. Rainfall and availability of water bodies are necessary for adult mosquitoes to lay eggs and for larvae to survive.[123] Evidence suggests that at least 80 mm of monthly rainfall is necessary for suitable transmission.

Together with climatic conditions, the environment determines the spatial distribution of *Anopheles* mosquitoes, by providing breeding sites and optimal survival conditions.[124,125] Moreover, Lyon et al., (2017) found a significant association between increasing low temperature in higher altitudes and the development of malaria parasites in Ethiopia.[126] Increasing temperatures in the region are eroding the perceived barrier to malaria transmission, allowing more favourable conditions to begin climbing into densely populated highland areas. The malaria indicator focuses on determining global changes in the number of months per year suitable for transmission of the malaria parasites over time between high- and lowland areas according to different categories of the UNDP Human Development Index (Figure A1)

Historical monitoring of malaria suitability (1951-2022)

The length of the transmission season, measured as the number of months suitable for malaria transmission per year from 1951 to 2022 was calculated on grid with a resolution of 0.1° x 0.1°/ 9km x 9km. Climate suitability was based on empirically derived thresholds of precipitation, temperature, and relative humidity for *Plasmodium falciparum* and *Plasmodium vivax*.

Monthly climate information between 1951 and 2022 was obtained from the ERA5-Land repository.[127] Relative humidity in percentage was calculated using the August-Roche-Magnus equation, which derives this value by combining dew point temperature and temperature, using the formula below[128]

$$\boldsymbol{RH = 100 * \frac{\exp\left(\frac{aT_d}{b+T_d}\right)}{\exp\left(\frac{aT}{b+T}\right)}}$$

Where *a* and *b* are the coefficients 17.625 and 243.04, respectively, and T and T_d are temperature and dew point temperature in °C.

Elevation data were extracted from the JISAO repository, University of Washington (http://research.jisao.washington.edu/data_sets/elevation/).

Land cover classes from 2019 were extracted from the Copernicus Global Land Monitoring Service repository at 100m resolution (https://land.copernicus.eu/pan-european/corine-land-cover) in tag image file format (*.tif*) and was assumed to be constant across the entire time series. Suitable land classes were determined according to the literature about the environmental requirements and limitations of different dominant vector species (DVS) of human malaria[124,125,129] Namely, close, and open forests, herbaceous wetlands, cultivated and managed vegetation/ agriculture, and permanent water bodies, were considered as potentially suitable areas for settlement of *Anopheles* mosquito populations.

Suitability for a particular month was defined as the coincidence of precipitation accumulation greater than 80 mm, average temperature between 18°C and 32°C for *P. falciparum,* and 14.5ºC and 33ºC for *P. vivax*, and relative humidity greater than 60% [123,130]. These combined values reflected the climatic limits for potential transmission of parasites. The number of months per year with suitable conditions were then stratified by elevation using a threshold of 1500 m.a.s.l. to differentiate low from highland areas (highlands >= 1500 m.a.s.l.). Averages by country, HDI category, and WHO and Lancet Countdown regions were computed, weighted by the amount of suitable land cover classes.

Results were visualised using time series line plots (Figures A2 and A4), and maps and tables containing the change in the number of suitable months between the decades 1951-1960 and 2013-2022 (Tables A1 to A3 and Figure A5).

Climate change projections (1995-2100)

The climatic limits for potential transmission of *P. falciparum* parasites described above were used for producing climate change projections in the context of scenarios SSP1-2.6 (sustainable future with low-emissions) and SSP3-7 (future with significant inequalities and high-emissions). Daily climate variables were extracted from the ISIMIP3b simulation round and grouped by month. Mean number of months suitable for malaria transmission in the periods 2021-2040, 2041-2060, and 2081-2100 were compared to the baseline period 1995-2014. The median percentage change between periods was computed across GCMs and differences between models were incorporated as a measure of spread of the predictions.

Data

Historical monitoring of malaria suitability (1951-2022)

Variable	Source	Frequency of update	Spatial resolution	Temporal range
Monthly 2-meter dew point temperature	ERA5-Land	Monthly with a 3-month delay relative to present	0.1°/ 9 km	Jan 1951 to 2022
Monthly 2-meter temperature				
Monthly total precipitation				
Altitude	JISAO	-	0.5°	-
Land cover	Copernicus Global Land Service	Annually	100 m	2019

Climate change projections (1995-2100)

Variable	Source	Models	Spatial resolution	Temporal range
Near surface air temperature	ISIMIP3b	GFDL-ESM4<br>IPSL-CM6A-LR<br>MPI-ESM1-2-HR<br>MRI-ESM2-0<br>UKESM1-0-LL	0.5º	Historical: 1995 – 2014<br>Future: 2015 – 2100
Precipitation				
Near surface relative humidity				

Caveats

These results are based on climatic data, not malaria case data. The malaria suitability climate thresholds used are based on a consensus of the literature. In practice, the optimal and limiting conditions for transmission are dependent on the parasite and vector species.[131] Control efforts might limit the impact of these climate changes on malaria or conversely, the climate suitability may either enhance or hamper control efforts.[132]

The inclusion of land suitability assumes a constant distribution of land cover classes as reported in 2019. However, dynamics in malaria transmission are highly correlated to changes in land use patterns, such as deforestation and urbanization.[129,133] Additionally, different *Anopheles* species have adapted to different types of

forests.[129] This indicator assumes a strict relationship between forest type and suitability for vector development, hence omitting disease dynamics at lower scales.

Additional analysis

Historical monitoring of malaria suitability (1951-2022)

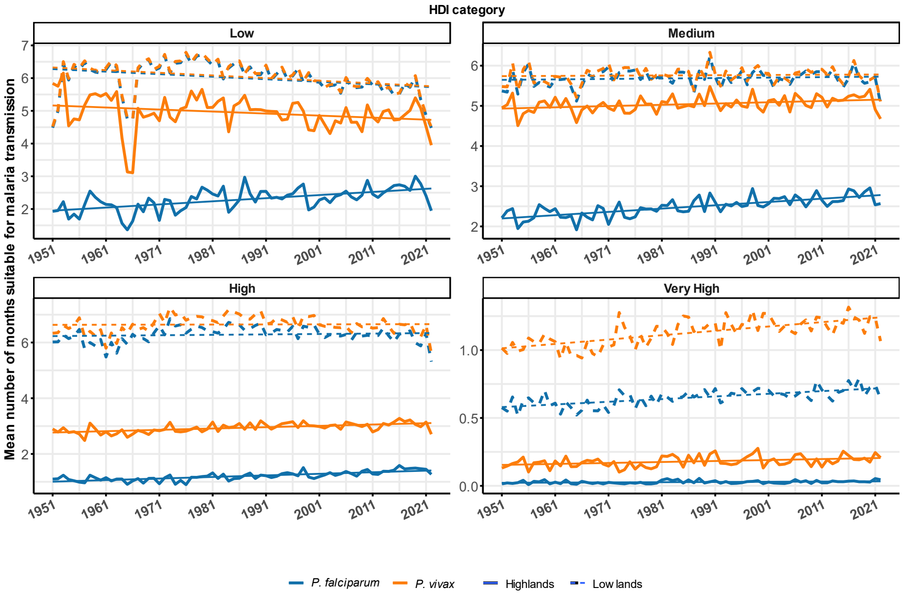

Figure 58. Mean number of months suitable for malaria transmission between 1951 and 2022, weighted by the amount of land suitability for *Anopheles* mosquitoes. Stratification by HDI level and elevation (highlands above or equal to 1500 masl). Linear regression was used for trend estimation.

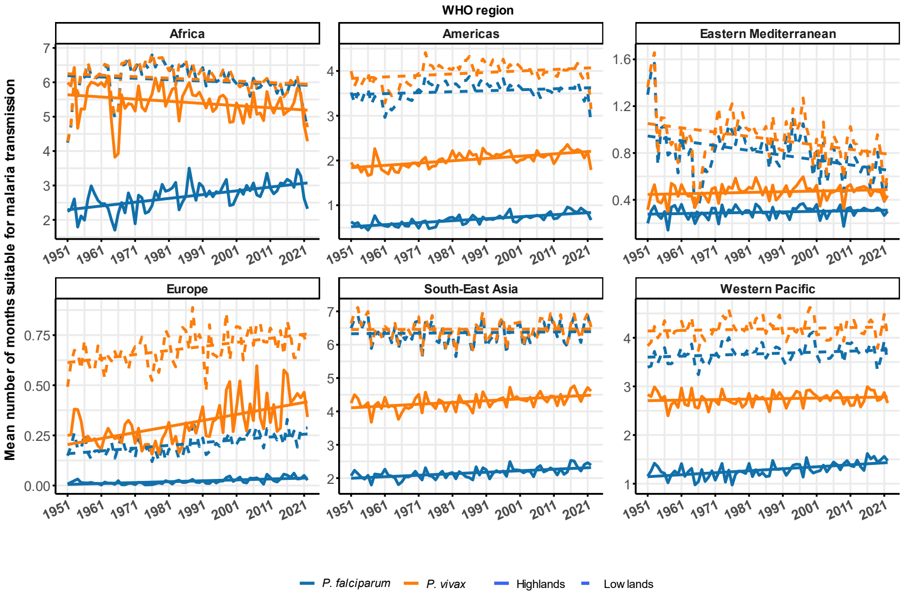

Figure 59. Mean number of months suitable for malaria transmission between 1951 and 2022, weighted by the amount of land suitability for *Anopheles* mosquitoes. Stratification by WHO region and elevation (highlands above or equal to 1500 masl). Linear regression was used for trend estimation.

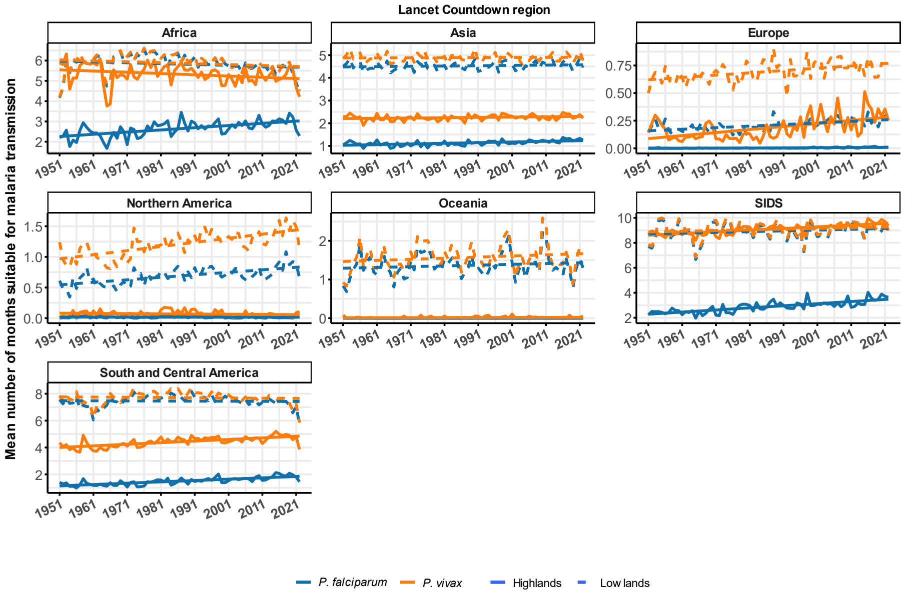

Figure 60. Mean number of months suitable for malaria transmission between 1951 and 2022, weighted by the amount of land suitability for *Anopheles* mosquitoes. Stratification by Lancet Countdown region and elevation (highlands above or equal to 1500 masl). Linear regression was used for trend estimation.

**HDI level**	***P. falciparum***		***P. vivax***	
	**Highlands** *N months (%)*	**Lowlands** *N months (%)*	**Highlands** *N months (%)*	**Lowlands** *N months (%)*
Low	0.6 (29.5%)	-0.67 (-10.8%)	-0.54 (-9.84%)	-0.69 (-10.9%)
Medium	0.39 (17%)	0.15 (2.77%)	0.24 (4.92%)	0.13 (2.33%)
High	0.37 (33.6%)	0.21 (2.39%)	0.33 (11.7%)	0.17 (2.69%)
Very high	0.01 (45.4%)	0.1 (16.5%)	0.03 (19.1%)	0.16 (15.6%)

Table 20. Percentage change in median number of months suitable for malaria transmission between 1951-1960 and 2013-2022 stratified by Human Development Index (HDI) and altitude (highlands above or equal to 1500 masl).

**WHO Regions**	***P. falciparum***		***P. vivax***	
	**Highlands** *N months (%)*	**Lowlands** *N months (%)*	**Highlands** *N months (%)*	**Lowlands** *N months (%)*

Africa	0.63 (25.7%)	-0.44 (-7.25%)	-0.62 (-10.5%)	-0.47 (-7.56%)
Americas	0.28 (48.8%)	0.12 (3.57%)	0.36 (19.4%)	0.21 (5.47%)
Eastern Mediterranean	0.01 (3.95%)	-0.3 (-32.3%)	0.04 (10.2%)	-0.22 (-21.4%)
Europe	0.02 (172%)	0.09 (48.9%)	0.19 (75.4%)	0.11 (17.2%)
South-East Asia	0.27 (12.9%)	0.02 (0.32%)	0.32 (7.52%)	-0.03 (-0.52%)
Western Pacific	0.26 (21.3%)	0.18 (5.16%)	0.01 (0.42%)	0.08 (2.02%)

Table 21. Percentage change in median number of months suitable for malaria transmission between 1951-1960 and 2013-2022 stratified by WHO region and altitude (highlands above or equal to 1500 masl).

Lancet Countdown Regions	*P. falciparum*		*P. vivax*	
	**Highlands** *N months (%)*	**Lowlands** *N months (%)*	**Highlands** *N months (%)*	**Lowlands** *N months (%)*
Africa	0.61 (25.6%)	-0.47 (-7.87%)	-0.61 (-10.6%)	-0.49 (-8.12%)
Asia	0.18 (17%)	0.13 (2.89%)	0.04 (1.69%)	0.11 (2.18%)
Europe	0.01 (642%)	0.09 (49.6%)	0.16 (104%)	0.11 (18%)
Northern America	0.001 (15.2%)	0.25 (42.1%)	0.001 (13.5%)	0.4 (37.9%)
Oceania	0 (0%)	0.02 (1.59%)	0 (0%)	0.08 (5.74%)
SIDS	1.03 (41.5%)	0.45 (5.15%)	0.73 (8.21%)	0.43 (4.92%)
South and Central America	0.62 (48.9%)	0.02 (0.33%)	0.86 (21.4%)	0.01 (0.09%)

Table 22. Percentage change in median number of months suitable for malaria transmission between 1951-1960 and 2013-2022 stratified by Lancet Countdown group and altitude (highlands above or equal to 1500 masl).

HDI level	*P. falciparum*			*P. vivax*		
	**Highlands**	**Lowlands**	**Overall**	**Highlands**	**Lowlands**	**Overall**
Low	26.9%	19.05%	25.1%	25.36%	21.66%	21.74%
Medium	11.98%	10.29%	10.95%	13.53%	12.85%	13.11%
High	9.99%	17.44%	14.31%	9.13%	13.48%	11.79%
Very high	0.68%	9.51%	9.07%	4.07%	19.57%	18.39%

Table 23. Percentage of areas unsuitable for malaria transmission in the period 1951-1960 that became suitable by the period 2013-2022, stratified by Human-Development Index (HDI) and altitude (highlands above or equal to 1500 masl).

WHO Regions	*P. falciparum*			*P. vivax*		
	**Highlands**	**Lowlands**	**Overall**	**Highlands**	**Lowlands**	**Overall**
Africa	40.6%	34.83%	36.32%	47.16%	35.62%	37.86%
Americas	7.22%	8.86%	8.5%	5.91%	19.68%	17.37%
Eastern Mediterranean	1.86%	3.57%	3.23%	2.24%	5.68%	4.98%
Europe	1.63%	11.04%	10.8%	13.64%	20.93%	20.70%
South-East Asia	4.72%	36.6%	9.56%	8.03%	33.95%	9.52%
Western Pacific	4.54%	8.84%	7.79%	4.74%	9.73%	8.63%

Table 24. Percentage of areas unsuitable for malaria transmission in the period 1951-1960 that became suitable by the period 2013-2022, stratified by WHO region and altitude (highlands above or equal to 1500 masl).

Lancet Countdown Regions	*P. falciparum*			*P. vivax*		
	**Highlands**	**Lowlands**	**Overall**	**Highlands**	**Lowlands**	**Overall**
Africa	38.17%	16.95%	24.08%	34.99%	17.71%	19.68%
Asia	3.60%	7.35%	5.98%	5.55%	6.25%	6.02%
Europe	0.62%	11.69%	11.48%	11.53%	22.39%	22.15%
Northern America	0.26%	8.69%	7.83%	0.74%	20.13%	17.59%
Oceania	-	5.85%	5.84%	-	10.56%	10.54%
SIDS	16.93%	38.27%	20.34%	27.04%	45.45%	28.56%
South and Central America	17.56%	14.24%	15.11%	21.59%	17.01%	19.45%

Table 25. Percentage of areas unsuitable for malaria transmission in the period 1951-1960 that became suitable by the period 2013-2022, stratified by Lancet Countdown group and altitude (highlands above or equal to 1500 masl).

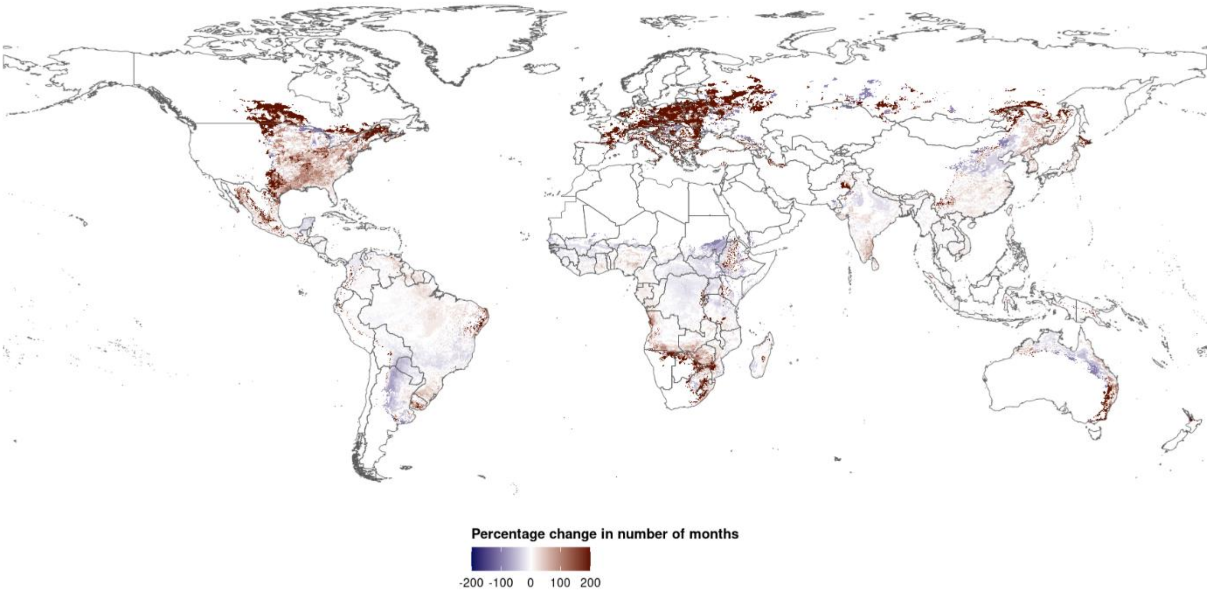

Figure 61. Change in the length of transmission season for *P. falciparum* from 1951-1960 to 2013-2022 in suitable land cover classes suitable for *Anopheles* mosquitoes.

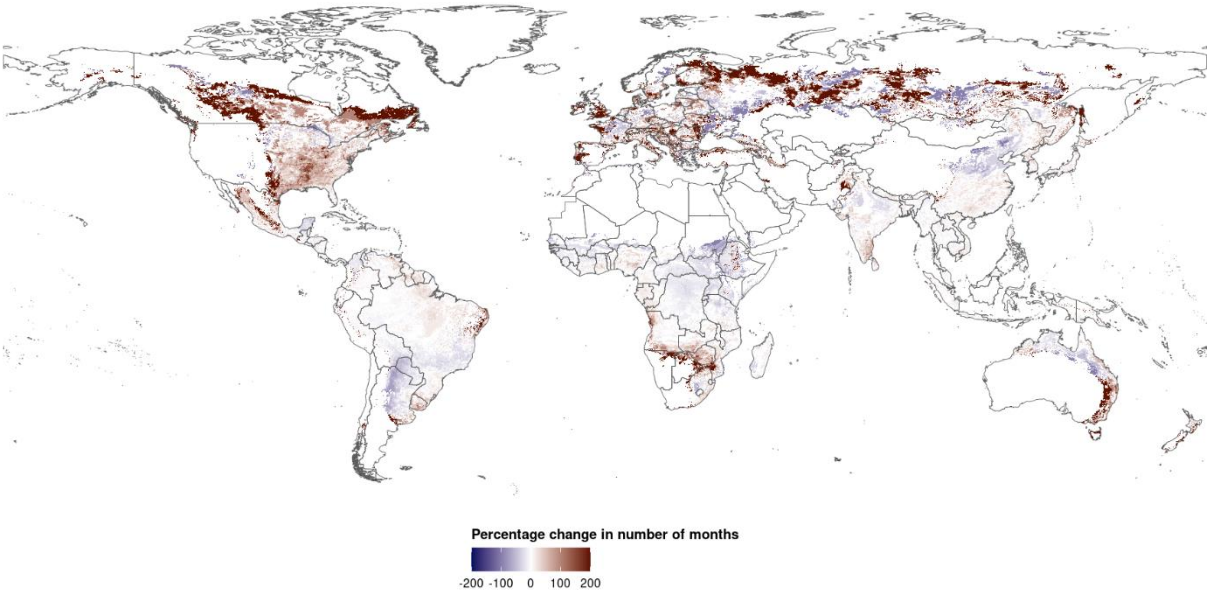

Figure 62. Change in the length of transmission season for *P. vivax* from 1951-1960 to 2013-2022 in suitable land cover classes suitable for *Anopheles* mosquitoes.

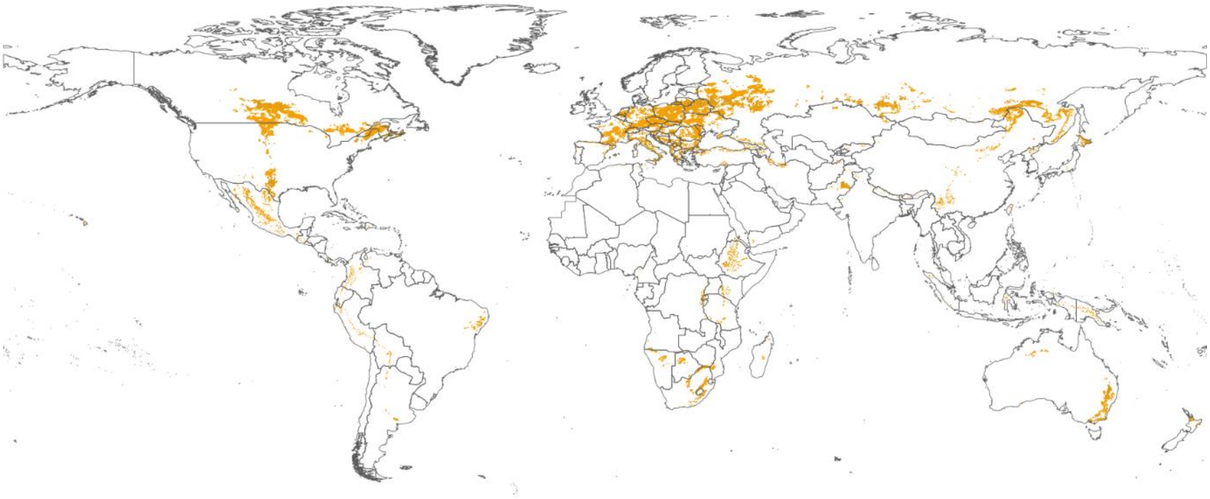

Figure 63. Newly suitable areas for transmission of *P. falciparum* in the period 2013-2022 (orange), compared to 1951-1960, in land cover classes suitable for *Anopheles* mosquitoes.

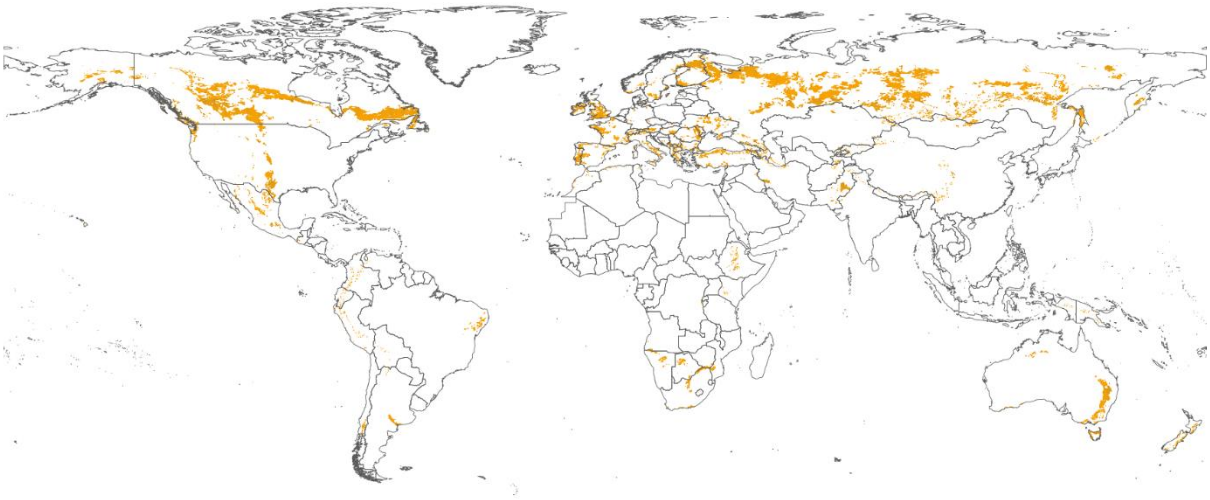

Figure 64. Newly suitable areas for transmission of *P. vivax* in the period 2013-2022 (orange), compared to 1951-1960, in land cover classes suitable for *Anopheles* mosquitoes.

P. falciparum			***P. vivax***		
**Highlands**	**Lowlands**	**Overall**	**Highlands**	**Lowlands**	**Overall**
8.01%	10.18%	9.85%	7.3%	18.92%	17.34%

Table 26: Percentage of global areas unsuitable for malaria transmission in the period 1951-1960 that became suitable by the period 2013-2022.

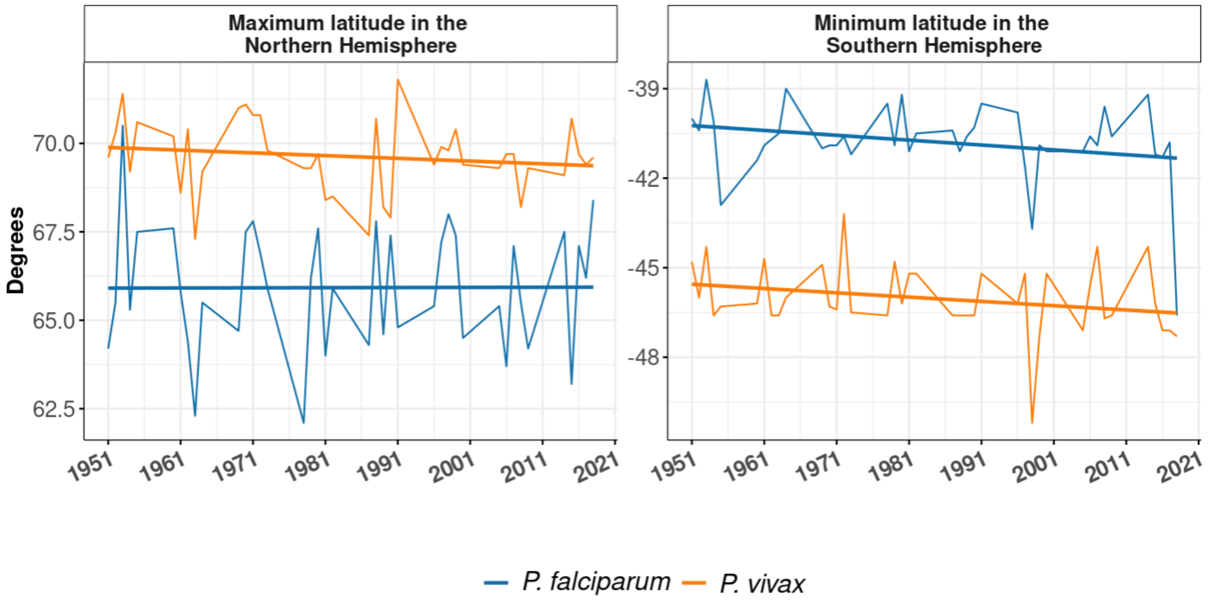

Figure 65. Maximum latitude in the Northern Hemisphere and minimum latitude in the Southern Hemisphere with at least one suitable month for malaria transmission per year. Difference in degrees between the period 1951-1960 and 2013-2022: 0.95º and 1º in the Southern Hemisphere for *P. falciparum* and *P. vivax*, respectively, and 0.6º and -0.65º in the Northern Hemisphere.

Climate change projections (1995-2100)

Projections of the number of months suitable for malaria transmission showed great spatial heterogeneity (Figure A6). Overall, our findings suggest that a 23% of areas not permissive for malaria circulation in the baseline period (1995-2014) could become suitable in the medium term (2041-2060) in the context of low emissions, compared to a 26% in a high-emission scenario. By the end of the century, although the amount of newly suitable areas would not expand further in the low-emission scenario, it would increase to 38% in a high-emission setting. Results showed an increase in the length of transmission season in Northern Asia, Europe, Central and North America, and the Middle East, by the period 2041-2060 in the SSP1-2.6 scenario. After this peak, there is a reduction in the transmission season towards the end of the century. In a high emission scenario (SSP3-7.0), the indicator showed a strong rise in the length of the transmission season in the Middle East, as well as in higher latitudes, namely North America, Northern Asia, and Europe. In contrast, there was a shortening in the length of the transmission season in low latitudes, such as South and Central Americas, the Caribbean, Africa, and Australasia. These results were published in the Climate Vulnerability Monitor, 3rd edition [22].

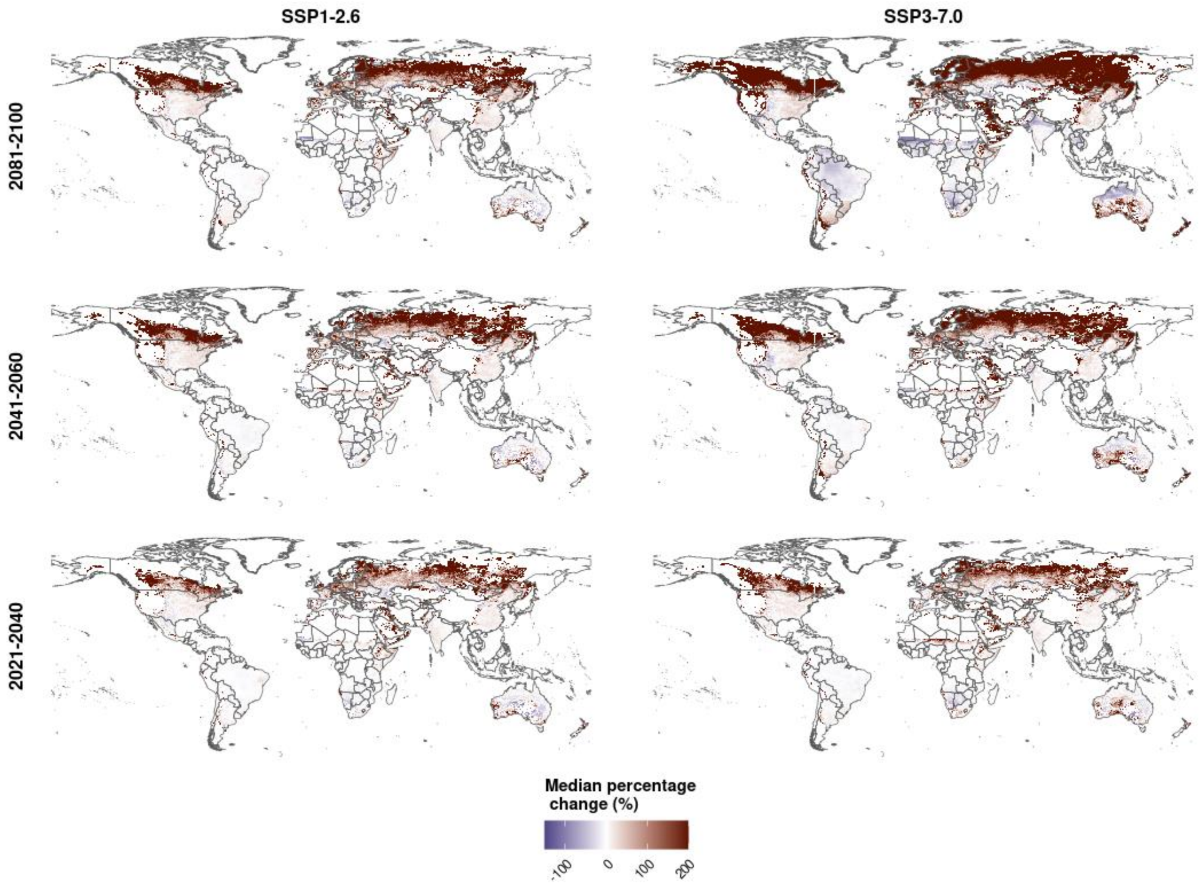

Figure 66. Change in the length of transmission season for *P. falciparum*, compared to 1995-2014. Change in length of the malaria transmission season measured as number of months per year with precipitation accumulation greater than 80mm, average temperature between 18°C and 32°C and relative humidity greater than 60%. Projections were done using the ISIMIP3b simulation round for scenarios SSP1-2.6 and SSP3-7.0 (adapted from the Climate Vulnerability Monitor, 3rd edition [22]).

Vibrio

Indicator authors

Prof Jaime Martinez-Urtaza, Prof Jan C. Semenza, Joaquin A. Trinanes

Methods

The methodology and input data for this indicator have been improved and extended for the 2023 report.

This indicator focuses on mapping environmental suitability for pathogenic *Vibrio* spp. in coastal zones globally (<10km from coast). *Vibrio* spp. are globally distributed aquatic bacteria that are ubiquitous in warm estuarine and coastal waters with low to moderate salinity. *V. parahaemolyticus*, *V. vulnificus*, and non-toxigenic *V. cholerae* (non-O1/non-O139) are pathogenic in humans. These *Vibrio* species are associated with sporadic cases of gastroenteritis, wound infections, ear infections, or septicaemia in circumscribed localities.

Vibrio ecology, abundances, distributions, and patterns of infection are often strongly mediated by environmental conditions.[134–137] On the basis of the consensus in the literature on what environments *Vibrio* infections may thrive, the indicator uses thresholds of >18°C for Sea Surface Temperature (SST) and <28 PSU for Sea Surface Salinity (SSS). The *Vibrio* suitability regions were determined based on a threshold-based approach for sea surface temperature and sea surface salinity estimates. Those areas showing temperatures above 18ºC and salinities below 28 PSU were flagged as suitable for *Vibrio*. These thresholds were used considering previous studies[138,139] and match the values currently being used for the global operational *Vibrio* suitability fields. The threshold value for

salinity is well below the usual ranges in most of the open ocean and takes into account the potential local decreases due to freshwater fluxes into the ocean (e.g., precipitation, runoff), making it a conservative estimate. For SST and SSS, only those cells closer than 10km to the global coastline were analysed. This band represents the areas where human exposure to *Vibrio* via direct contact with water is maximum, and also the region where most of the aquaculture-related activities, another core source of vibriosis, take place.

In previous Lancet Countdown reports, the *Vibrio* indicator was estimated based on the two environmental factors, seawater temperature and salinity, missing other key elements related to exposure and transmission of *Vibrio* illness, such as socioeconomic and demographic aspects. The advent of a new generation of models, such as those participating in CMIP6 (Coupled Model Intercomparison Project 6)[140], in combination with the new Shared Socioeconomic Pathways (SSPs)[141], has provided an exceptional opportunity to introduce a wider prospect and more robust projections into the models, integrating an increasing resolution and with key socioeconomic drivers (economic growth, demography, education and technological development). For the historical period, the *Vibrio* indicator has been estimated from datasets of SST and SSS that assimilated in-situ and satellite observations.

For *Vibrio* indicator projections, data from CMIP6 (AWI-CM-1-1-HR and CNRM-CM6-1-HR) were used to estimate areas and periods of *Vibrio* suitability and population at risk. Additionally, the Inter-Sectoral Impact Model Intercomparison Project (ISIMIP) Project 2b annual global population data were employed to compute the population at risk. The population potentially affected by exposure to Vibrio has been selected based on the ad-hoc distance of 100km between areas showing Vibrio suitability and the centre of the population cell for that time period. Climate, population and socioeconomic projections were combined to generate more accurate estimates of changes in Vibrio suitability and provide a global estimate of the population at risk of vibriosis for 2022 compared to a 1995-2014 baseline with data coverage from 1982 to present. A conservative assumption of infection rate per 100,000 population of 0.3 reported for the USA was applied (as estimated by both COVIS-CDC and FoodNet for the USA)[142] and took in consideration the limitations of surveillance data and underreporting in the USA, scaled up the number of infections 143 times[143] to calculate a more probable incidence of disease.

Finally, the climate, population and socioeconomic projections included into the framework of the Shared Socioeconomic Pathways (SSPs)[144] were considered to provide accurate estimates of future changes in *Vibrio* suitability and population at risk and generate projections for a low- and high-emission scenarios (SSP1-2.6. and SSP3-7.0 respectively) by the end of the century compared to the pre-industrial period.

Here suitability is reported at two levels. the length in Km of coastline that experienced suitable conditions for *Vibrio* infections and the period of suitable conditions for Vibrio in days per year. These two indicators were calculated globally (for all coastal countries) and the results summarised by country and compared to historical data since the records began in 1982.

Data

1. Sea surface temperature data from the Global Ocean OSTIA Sea Surface Temperature and Sea Ice Reprocessed dataset between 1982-2022.[145]
2. Sea surface salinity data from the Mercator Ocean Reanalysis.[146]
3. AWI-CM-1-1-HR and CNRM-CM6-1-HR sea surface temperature (SST) and sea surface salinity (SSS) from CMIP6 (2015-2100) SSP126 and SSP370 experiments.[147]
4. ISIMIP2b annual global population data at 0.5º resolution for the period 2006-2100 (SSP245&SSP585) and 1850-2015 (historical).[148]
5. Coastline length dataset from the World Factbook data.[149]

Caveats

The results are derived on the basis of suitable SST and SSS conditions only, and do not include other potentially important drivers (e.g., globalisation), environmental predictors of pathogenic *Vibrio* infections (e.g., chlorophyll-*a*, turbidity) or disease case data. Nevertheless, these associations have been explored and are reported in the supporting references included above.

In the global analysis, the slope of the trendlines over the time series is mostly flat for the tropical/subtropical region and the southern Hemisphere. However, the SST-only suitability shows a strong upward trend in the southern hemisphere, indicating that on average temperature conditions are also improving growth conditions for *Vibrio* in these areas, while SSS is generally limiting. However, locally suitable SSS conditions will also occur in

these regions based on, for example, variation in local rainfall and river runoff, which can make these regions sporadically suitable for *Vibrio* infections.

Additional analysis

Projections for future changes in Vibrio suitability and population at risk by the end of the century compared to the pre-industrial period were generated for a low- and high-emission scenarios (SSP1-2.6 and SSP3-7.0 respectively). Models showed a steady increase in number of cases and the extension of the coastal areas (Figure 2) showing Vibrio suitability until the end of the 21st century, with a larger slope for SSP370. The exception Is AWI-CM-1-1-HR SSP126, whose slope shifts from slightly positive to slightly negative after the middle of the century (Fig 5). The mid-term projection shows a general increase in the number of cases and coastline length suitable for Vibrio, but SSP126 model trends stabilize near zero or even show a decrease for AWI-CM-1-1-HR SSP126 in the second half of the century. The average values of coastline length for the 2041-2060 period are 25% above the baseline (2015-2022) for SSP126 CMNR, 17% for SSP126 AWI, 34% for SSP370 CMNR, and 30% for SSP370 AWI. When the estimated number of cases is considered, those values are 39% (SSP126 CMNR), 23% (SSP126 AWI), 46% (SSP370 CMNR) and 45% (SSP370 AWI). In summary, under the sustainable development scenario SSP126 CMNR (AWI), 4.52% (4.58%) of the global coastal area is projected to be suitable for transmission of Vibrio by the middle of the century. For the scenario with high GHG emissions SSP370 CMNR (AWI), the values increase to 4.54% (4.84%).

As for historical data, all WHO regions but the Eastern Mediterranean Region, which could be considered an outlier due to its low values when compared to the rest, show a positive trend in the number of estimated cases and coastline length, with the highest (resp. lowest) trend values in the Western Pacific Region (resp. European Region) for the number of cases and in the European Region (resp. African Region) for the coastline length (Figure 67, Figure 68). The shift in the number of cases from countries with lower HDI levels (Figure 69) to those with higher levels, is mostly the result of those countries increasing their HDI scores. A similar trend can be detected in the length of coastline showing suitable conditions for *Vibrio* (Figure 70).

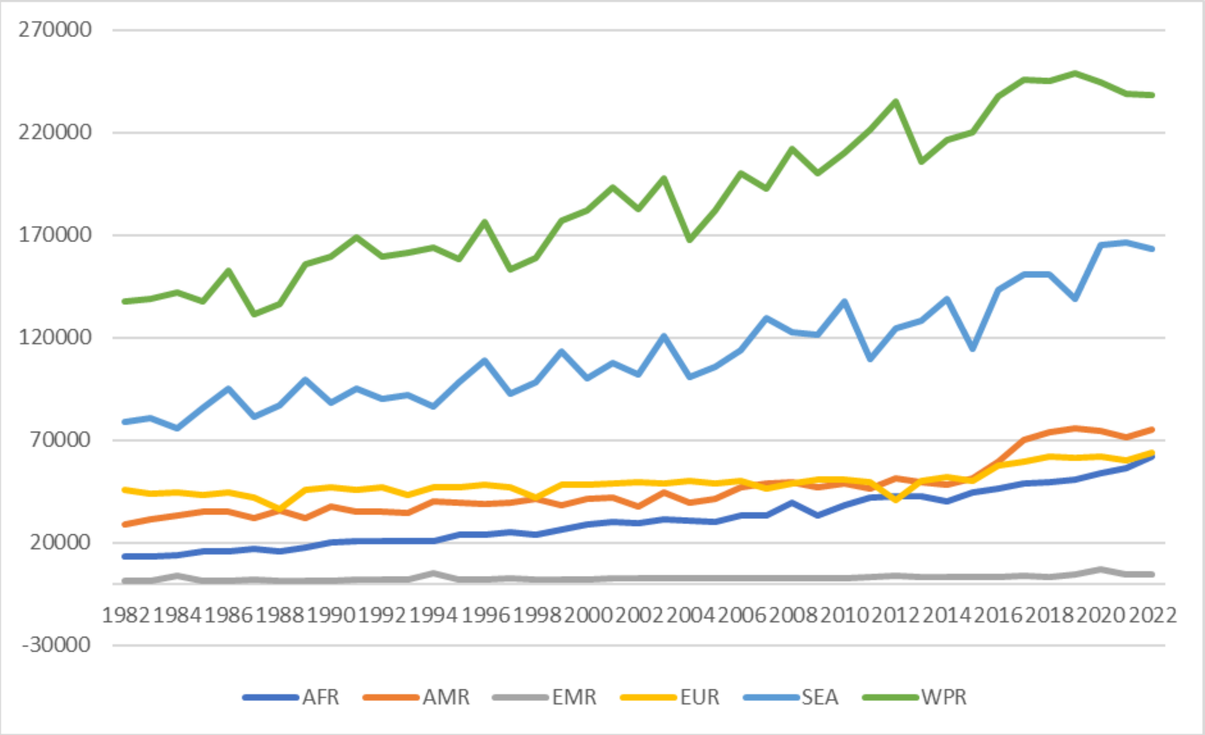

Figure 67: Temporal evolution of the number of cases per WHO region. The figure shows a positive trend over the study period with higher values in more densely populated areas in the Western Pacific Region and South-East Asia.

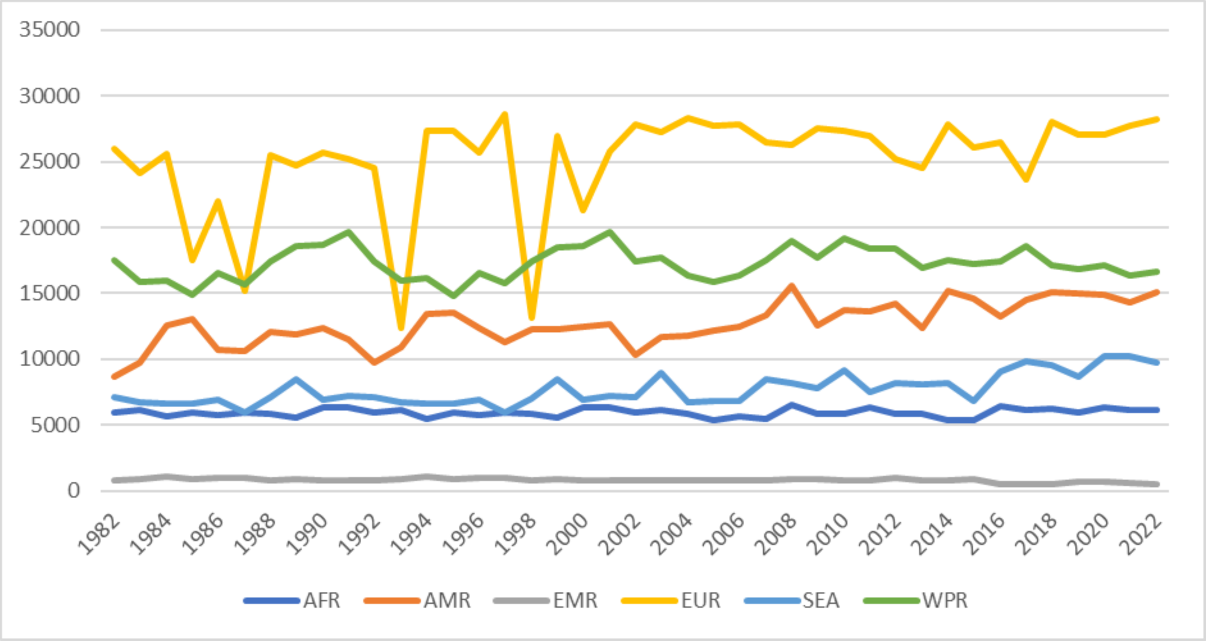

Figure 68: Length of coastline showing suitable conditions for Vibrio per WHO region. The increasing trend is larger in the European Region and the Region of the Americas.

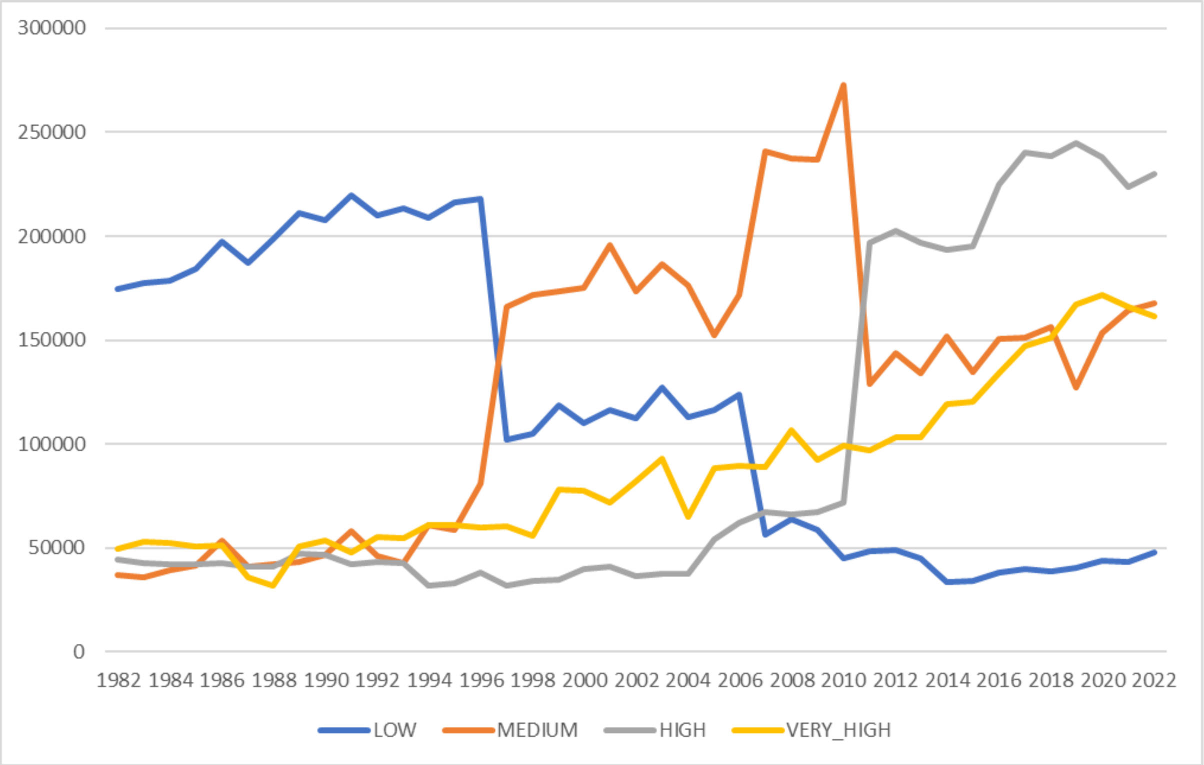

Figure 69: Temporal changes in the number of cases per HDI category. The abrupt changes in the time series correspond to when countries transition from one HDI category to another (e.g. China moving from low to medium in 1997, and from medium to high in 2011).

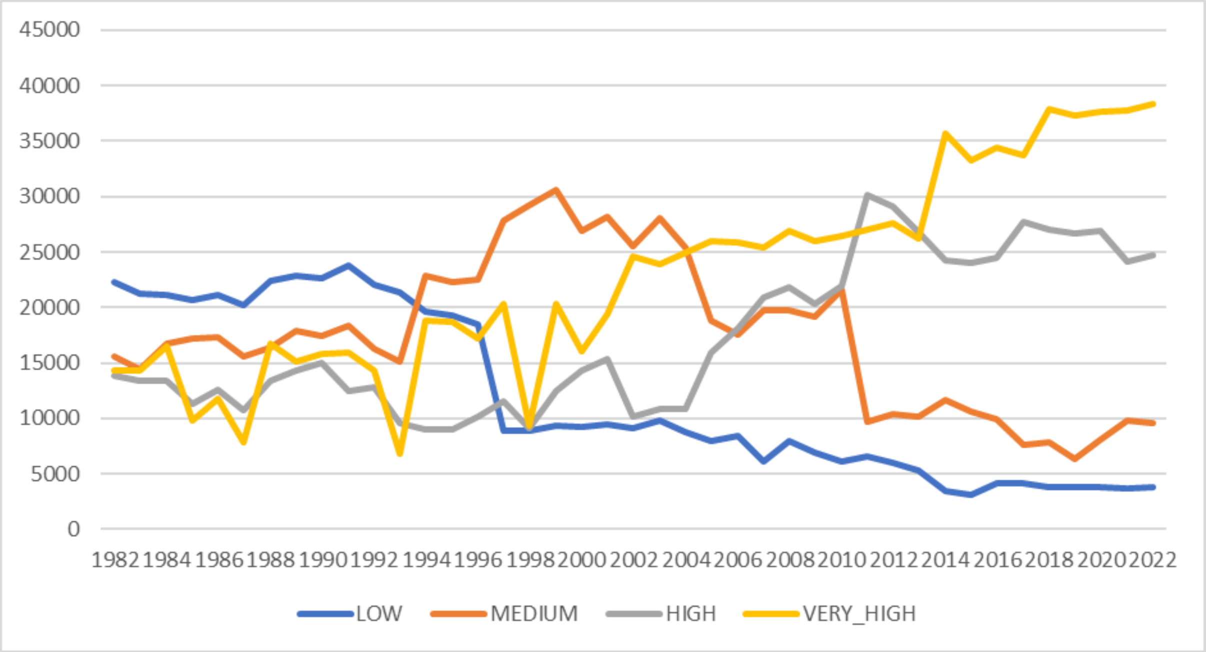

Figure 70: Length of coastline (in km) showing Vibrio suitability at some point of the year per HDI region. The trend varies across HDI categories and it is more pronounced for High and Very High, as more countries migrated from lower levels.

Indicator 1.4: Food Security and Undernutrition

This indicator consists of two sub-indicators. The first tracks risks to marine food security by monitoring changes in sea surface temperature and the consumption of farmed- or catch-based fish products. The second sub-indicator tracks the impact of climate change and income on the incidence of food insecurity.

Food insecurity

Indicator authors

Dr Shouro Dasgupta, Prof Elizabeth J.Z. Robinson

Methods

The methodology of this indicator is based on previously published models.[150] To track the impact of climate change and income on the incidence of food insecurity, a panel data regression was used with coefficients that vary over time. To operationalise the concept of climate change, the focus was on the number of heatwave days, and the frequency of droughts, during the four major crop growing seasons in each region[151]. A heatwave is defined as a period of at least two days where both the daily minimum and maximum temperatures are above the 95th percentile of the respective climatologies (Indicator 1.1.2) in each region. The gridded 95th percentile of daily minimum and maximum temperatures, taken from the ERA5-Land hourly reanalysis[152], were calculated for 1986-2005. The lagged number of heatwaves and frequency of droughts (measured by SPEI-12) was used during the crop growing seasons for each year during 2014-2021.

Increase in the number of heatwave days can affect food insecurity through multiple pathways[153]. These can variously be through the impacts of heat stress and droughts on crop yields, on agricultural labour and therefore crop production and agricultural income, on non-agricultural labour and non-agricultural income, on health and the ability to earn enough to afford food, on food prices and therefore the affordability of food, and on food supply chains and therefore the variety of food (which can be summarised as income and food supply effects). Our regression also includes twelve-month Standardized Precipitation Evapotranspiration Index (SPEI) as a measure of drought. SPEI-12 was computed using precipitation data from ERA5-Land monthly averaged reanalysis[152] and the SPEI package in R[154].

Two dependent variables were considered: first, the probability of moderate to severe food insecurity; and second the probability of severe food insecurity from the FAO Food Insecurity Experience Scale (FIES)[155]. To account for unobserved heterogeneity such as differences in food and storage policies across countries and changes in the prices of food items from year to year, our specification also includes both location and time (year) fixed-effects. The standard errors are clustered at the country-level[156]. Panel data specification can be written as follows:

$$FIES_{it} = \beta_1(\tau_t) + V_{(it)} + \gamma'(\tau_t)X_{(it)} + \alpha_{(i)} + \mu_{(it)}$$

where $FIES_{it}$ is the probability of moderate or severe food insecurity or probability of severe food insecurity, $\boldsymbol{V}_{it}$ is a vector of change in the number of heatwave days and the frequency of drought months during the four major crop growing season, and $\boldsymbol{X}_{it}$ is a vector of relevant variables affecting food insecurity – income and a dummy to control for the COVID-19 pandemic in 2020. $\boldsymbol{\mu}_{it}$ is a random error term. All variables are recorded for different locations with index $i = 1, \dots, N$ and over a number of years $t = 1, \dots, T$. The time-varying coefficients allowed us to examine whether the relationship between temperature anomaly and food insecurity had evolved over time.

In the second-step, a counterfactual analysis was conducted to explore the extent to which food insecurity may have been affected by climate change[150]. To do this the cumulative impacts of increasing frequency of heatwaves and frequency of drought months above the historical norms over the period 1981–2010 were computed. The counterfactual impact of climate change on food insecurity is derived by combining the coefficients from the time-varying regression with the historical norm average and each year for which food security data are available. The effects of increases in the frequency of heatwaves and frequency of drought months was considered over compared to the baseline (1981–2010) under which frequency of heatwaves increases according to its historical trend. Regression results are summarised in Table 28.

Data

1. Hourly climate data (2m air temperature) from the European Centre for Medium-Range Weather Forecasts (ECMWF) ERA5-Land reanalysis.[157]
2. Monthly climate data (total precipitation) from ECMWF ERA5-Land reanalysis.[112]
3. Food insecurity from the FAO Food Insecurity Experience Scale.[158]

Caveats

The main caveat for temperature anomaly food insecurity indicator is the possible recall bias in the survey data and the bias that may have been induced to interviews during the pandemic being conducted by phone instead of in-person visits.

Future form of the indicator

In the future, disaggregated analysis will be provided by HDI groups.

Additional analysis

We estimate food insecurity under a no climate change scenario (1995-2014) using the historical data from five GCMs from in ISIMIP3b, and then compare the food insecurity outcomes from this scenario against climate projections from three-time epochs, 2021–2040 (near-term), 2041–2060 (medium-term), and 2081-2100 (long-term) under both SSP1-2.6 and SSP3-7.0, to obtain the change in future food insecurity outcomes compared to synthetic historical food insecurity. The output is percentage-point change in food insecurity indicators due to future climate change compared to a reference period of 1995-2014, aggregated to the country level.

During 2021–2040, under a lower-emissions scenario of SSP1-RCP2.6, moderate or severe food insecurity is projected to be 3.7 percentage points higher compared to the 1995–2014 reference period, but 1.9 percentage points higher during 2081–2100, reflecting the benefits of achieving a net zero target by 2050 (Table 27). Under the high-emissions and near catastrophic SSP3-RCP7.0 scenario, food insecurity is projected to be 4.1 percentage points higher during 2021–2040, but 12.8 percentage points higher during the 2081–2100 period. These findings highlight the global food security co-benefits of mitigation and achieving low emission scenarios including net-zero by 2050, with food insecurity projected to be significantly worse under the higher warming scenarios (Figure 71).

Table 27: Change (percentage-point) in moderate-severe food insecurity due to climate change-induced change in the number of heatwave days with respect to the 1995-2014 baseline.

Scenario	SSP1-RCP2.6			SSP3-RCP7.0		
	2021-2040	2041-2060	2081-2100	2021-2040	2041-2060	2081-2100
Moderate or severe food insecurity	3.7	2.3	1.9	4.1	6.6	12.8

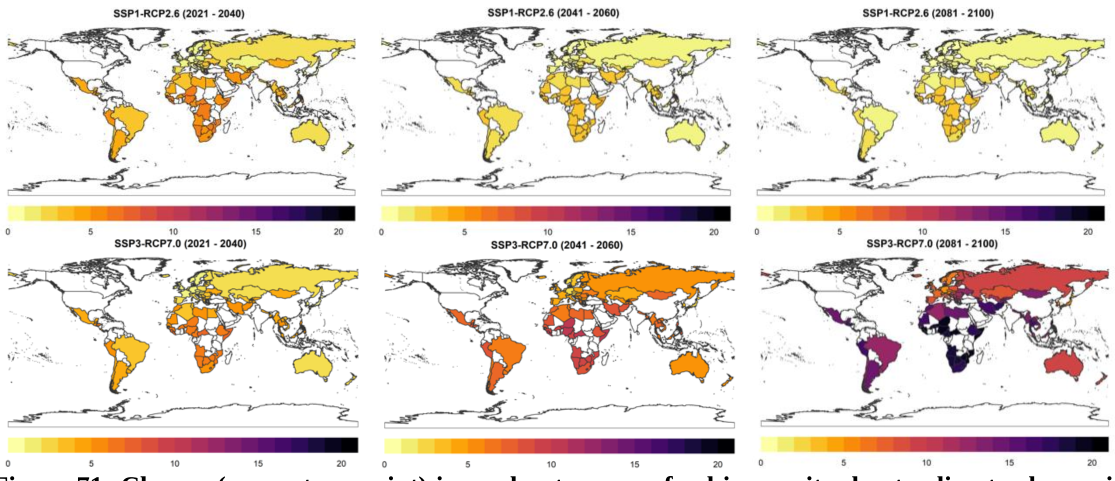

Figure 71: Change (percentage-point) in moderate-severe food insecurity due to climate change-induced change in the number of heatwave days with respect to the 1995-2014 baseline.

Table 28: Relationship between frequency of heatwaves and droughts, and food insecurity during 2014-2021 using a time-varying regression. 95% confidence intervals in parentheses.

	Moderate or severe food insecurity
Low income	0·183
	(0·173, 0·193)
High income	-0·115
	(-0·109, -0·121)
COVID-19 dummy	0·158
	(0·150, 0·166)
Heatwave frequency$_{(t)}$	
2014	1·16
	(1·01, 1·31)
2015	1·52
	(1·40, 1·64)
2016	1·93
	(1·80, 2·06)
2017	2·27
	(2·16, 2·38)
2018	2·96
	(2·84, 3·08)
2019	3·3
	(3·20, 3·40)
2020	3·72
	(3·66, 3·78)
2021	4·03
	(3·94, 4·12)
Drought frequency$_{(t)}$	
2014	0·56
	(0·41, 0·71)
2015	0·59
	(0·49, 0·69)
2016	0·74
	(0·62, 0·86)
2017	0·91
	(0·83, 0·99)
2018	1·12
	(0·99, 0·1·25)
2019	1·34
	(1·20, 1·48)
2020	1·59
	(1·43, 1·75)
2021	1·78
	(1·66, 1·90)

Marine Food productivity

Indicator authors

Prof Maziar Moradi-Lakeh, Dr Fereidoon Owfi, Dr Mahnaz Rabbaniha, Prof Meisam Tabatabei

Methods

The methodology for this indicator applies to a wider geographical area and more countries compared with the 2022 Lancet Countdown report.[15] Sixteen major FAO (Food and Agriculture Organization of the United Nations) marine basins and two inland water basins which are important in terms of projected impacts and vulnerabilities associated with climate change were selected. One-hundred forty-eight countries located in these basins were chosen to assess changes in sea surface temperature (SST), as well as the decreased consumption of capture-based fish.

The input SST data for this indicator were retrieved from the ORAS5 global ocean reanalysis dataset from 1980 to 2022. A total number of 768,582 grid cells were used to have an accurate estimate for SST variations.

Moreover, the data concerning capture-based and farmed-based per capita fish consumption in the investigated countries from 1980 to 2020 were collected and analysed. Disability-adjusted life years (DALYs) attributed to diet low in seafood ω3 by the Global Burden of Diseases was provided based on LC grouping, WHO regions and HDI (Human Development Index) levels.

Data

1. Ocean data from the European Centre for Medium-Range Weather Forecasts (ECMWF) ORAS5 reanalysis.[159]
2. Capture-based and farmed-based fish consumption per capita from FAO, 1980-2020.[160]
3. Attributable DALYs to diet low in seafood ω3 from GBD, 1990–2019.[44]

Caveats

There is a lack of information and data in the available databases such as FAO on fish species composition of the captured and farmed fish products. This could, in turn, lead to some concerns about the methodological approach used to calculate ω3 intake. More specifically, most of the approaches are based on fish intake, which usually ignores or underestimates variations in ω3 contents of different types of fishes, and especially capture-based compared with farmed-based fish. It should also be highlighted that GBD estimates for the association between this dietary risk factor and cardiovascular diseases, as the primary reference for human health impacts, are not based on type and source of seafood products either.

Fish production data were used as a surrogate for fish consumption. This is not a completely accurate assumption, but there is no comprehensive alternative source of data for all the investigated countries.

Future form of the indicator

Further analysis will be required to connect the different components of the causality chain, i.e., between SST and health impacts.

Additional analysis

Although the North America and European regions still have the lowest average SST, they are projected to experience the highest increase by the year 2060 (Table 29). Figure 72 shows changes in 3-year moving average SST for all countries/territories with coastal waters in 2020-2022 compared to 1981-2010 baseline. The projected SST values under the SSP1-2.6 and SSP3-7.0 scenarios compared to the 1981-2010 baseline are shown in Figure 73. Despite a general increase in per capita fish consumption globally, the share of capture-based in total fish consumption has continued to decrease in 2020 (Figure 74). The increasing sea surface temperature well supports the consequent decline in fish capture (Figure 75).

Table 29: Sea Surface Temperature (SST) in 1980 and 2022, projected SST based on SSP1-2.6 and SSP3-7.0 compared to the reference period (1981-2010) for the coastal waters, by the Lancet Countdown (LC) grouping, WHO regions and Human Development Index (HDI) levels.

	**Weights: Number of countries (Total: 142)**						**Weights: Number of data points (Total: 768,582 for measured SST and 717,840 for projected SST)**					
	Measured SST(◦C)				Mean projected SST(◦C)		Mean measured SST(◦C)				Mean projected SST(◦C)	
Locations	1981-2010 (Reference)	1980	2022	Difference*	2041-2060 SSP1-2.6	2041-2060 SSP3-7.0	1981-2010 (Reference)	1980	2022	Difference*	2041-2060 SSP1-2.6	2041-2060 SSP3-7.0
LC grouping												
Africa	25.15	24.89	25.33	0.307	26.43	26.54	23.67	23.40	23.77	0.251	24.98	25.08
Asia	24.88	24.83	25.35	0.635	25.21	25.25	25.49	25.41	26.03	0.599	26.02	26.10
Europe	12.40	11.73	13.35	0.943	13.61	13.48	9.28	8.77	10.11	0.806	10.75	10.64
Northern America	7.30	7.04	8.03	0.669	9.65	9.67	7.35	7.08	8.07	0.667	9.69	9.71
Oceania	18.80	18.63	19.58	0.547	18.79	19.13	20.75	20.64	21.30	0.407	21.03	21.33
SIDS	27.63	27.63	27.93	0.354	27.68	27.86	27.99	27.94	28.50	0.484	28.04	28.22
South and Central America	24.13	24.20	24.17	0.184	25.40	25.53	22.08	22.10	22.01	0.118	23.40	23.54
WHO regions												
Africa	25.82	25.60	25.96	0.284	27.17	27.32	24.65	24.44	24.68	0.194	26.16	26.33
Americas	24.92	24.95	25.15	0.312	25.57	25.70	17.38	17.30	17.68	0.375	18.87	18.96
Eastern Mediterranean	24.89	24.79	25.27	0.566	24.98	25.00	24.61	24.46	24.97	0.500	24.90	24.92
Europe	13.33	12.71	14.25	0.960	14.53	14.41	10.01	9.52	10.83	0.825	11.48	11.37
South-East Asia	28.55	28.63	28.80	0.310	28.80	28.95	28.76	28.75	29.14	0.401	29.14	29.32
Western Pacific	25.88	25.81	26.33	0.457	25.95	26.11	23.54	23.41	24.19	0.590	23.90	24.06
HDI classification												
Low	27.23	27.07	27.47	0.336	28.04	28.20	27.34	27.24	27.56	0.298	27.89	28.06
Medium	26.77	26.71	27.01	0.339	27.57	27.71	27.21	27.11	27.61	0.430	27.83	27.99
High	24.96	24.82	25.27	0.416	25.53	25.63	25.48	25.36	25.78	0.387	26.23	26.35
Very High	16.87	16.52	17.56	0.754	17.75	17.73	13.66	13.41	14.30	0.631	14.98	15.00
N/A	26.8	26.8	26.96	0.238	26.63	26.78	26.79	26.76	27.02	0.323	26.4	26.58
All	22.93	22.73	23.34	0.499	23.66	23.73	19.69	19.51	20.18	0.517	20.70	20.78

* The coastal SST average for 2020-22 compared to the 1981-2010 baseline.

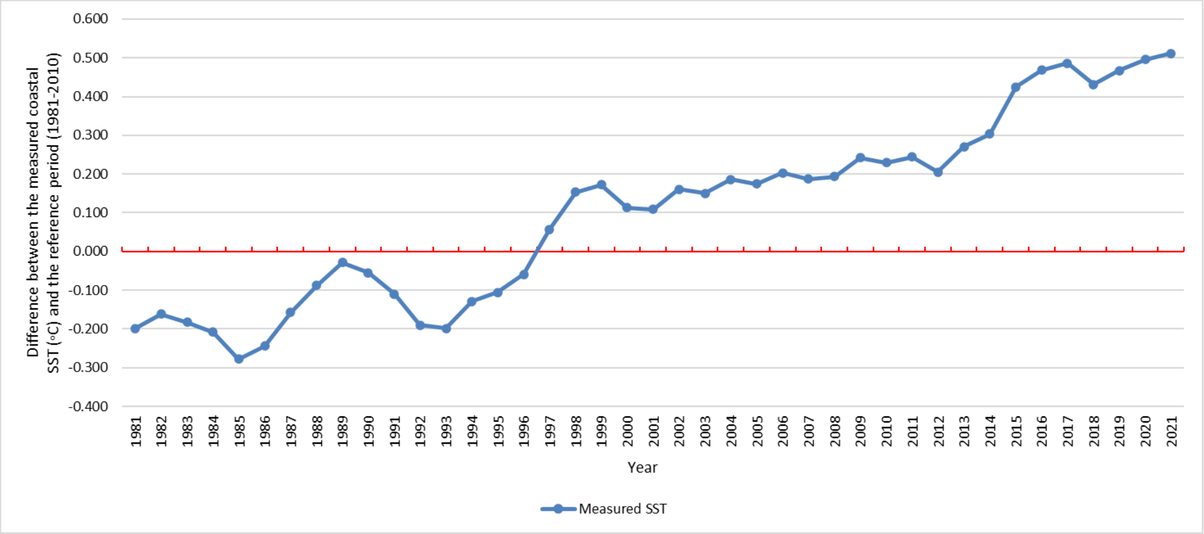

Figure 72: Change in the measured 3-year moving average of the global coastal sea surface temperature (SST) compared to the 1981-2010 baseline.

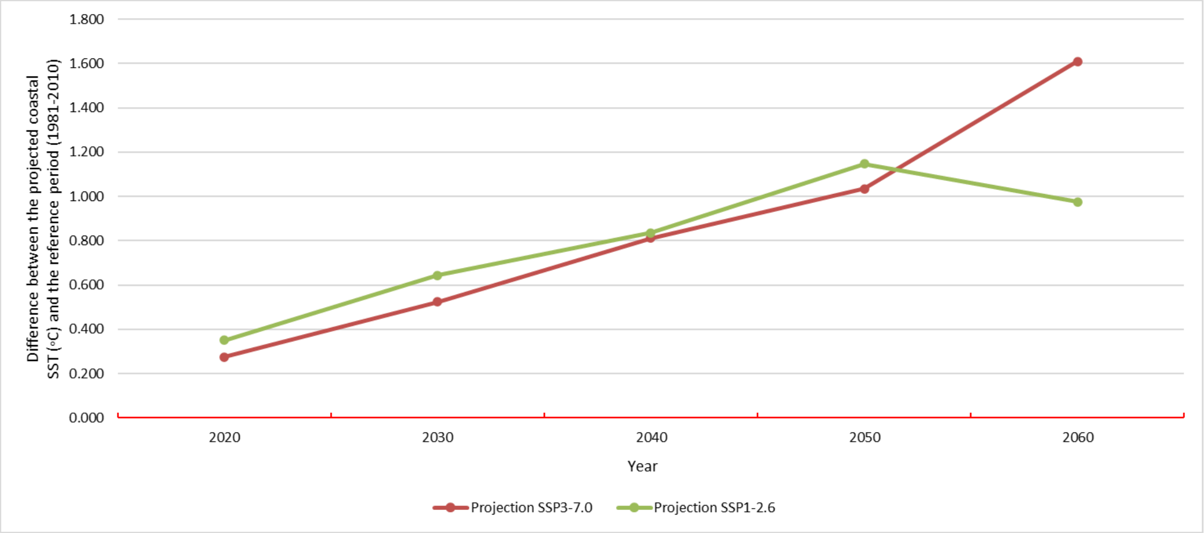

Figure 73: Projected change in global coastal sea surface temperature (SST) under the ssp1-2.6 and SSP3-7.0 scenarios with respect to the 1981-2010 baseline.

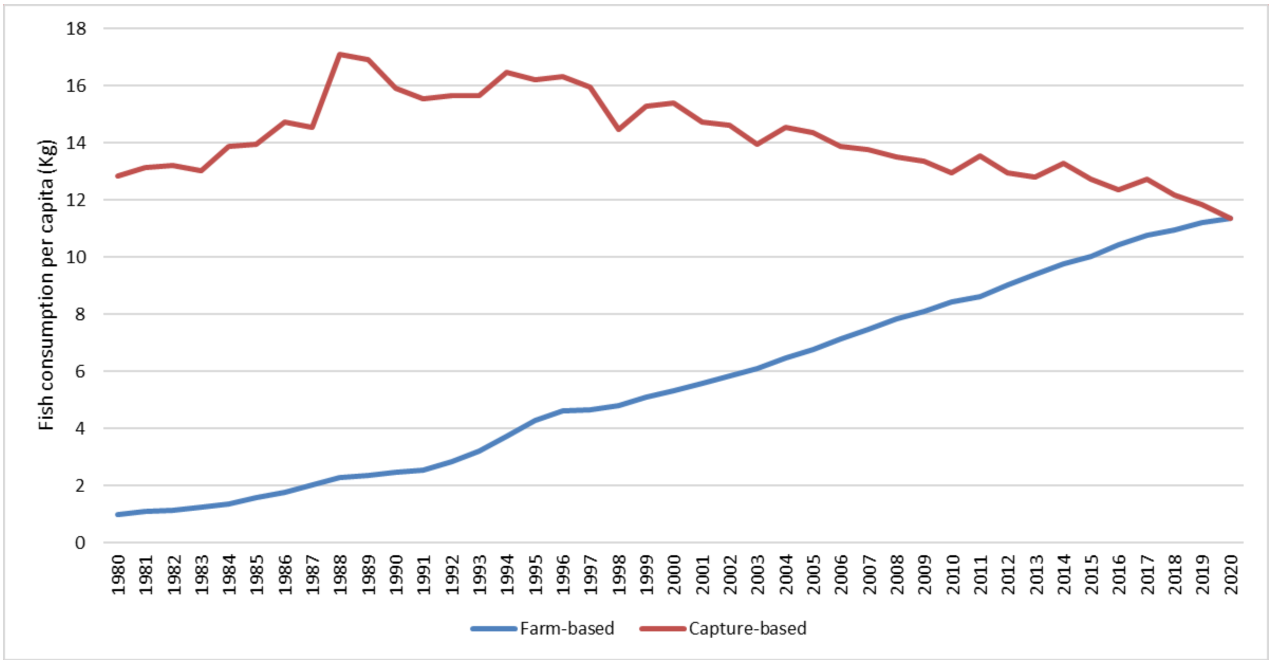

Figure 74: Population weighted average fish consumption per capita in 177 investigated countries/territories, separated by the origin of fish (marine capture-based and farm-based) from 1980 to 2020.

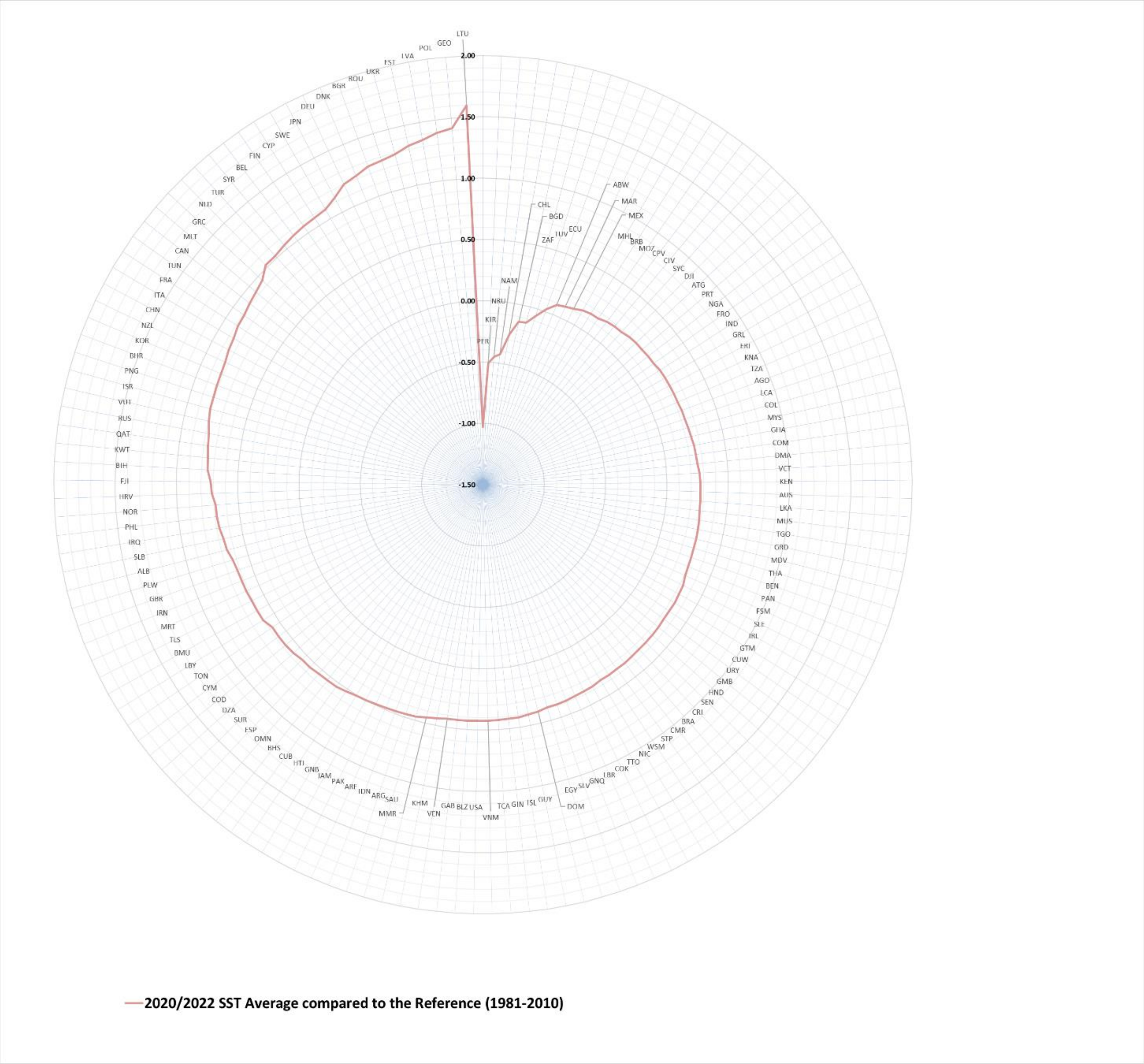

Figure 75: Changes in 3-year moving average sea surface temperature (ºC) for the countries/territories with coastal waters: 2020-22 compared to the 1981-2010 baseline.

Section 2: Adaptation, Planning, and Resilience for Health

Lead Authors: Prof Kristie L. Ebi and Prof Maria Nilsson

Research Fellow: Carole Green

2.1: Assessment and Planning of Health Adaptation

Indicator 2.1.1: National Assessments of Climate Change Impacts, Vulnerability and Adaptation for Health

Indicator authors

Dr Diarmid Campbell-Lendrum, Tara Neville

Methods

Data for this indicator was obtained from two sources. First, the number of countries that have committed to the COP26 Health Programme initiative to build a climate resilient health system [162] is tracked through the Alliance for Transformative Action on Climate and Health (ATACH)[162]. The commitment to build a climate resilient health system includes three key components: 1) conducting a climate change and health vulnerability and adaptation (V&A) assessment; 2) developing a health national adaptation plan (HNAP) informed by the results of the climate and health V&A assessment; and using the V&A and HNAP to facilitate access to climate change funding for health. Progress on the COP26 commitments are tracked through regular reporting to the ATACH. Data for this indicator represents the total number of countries that had made a commitment to the COP26 Health Programme as of April 30, 2023. For the most recent list of country commitments please see the ATACH website at: https://www.who.int/initiatives/alliance-for-transformative-action-on-climate-and-health/country-commitments.[161]

Global progress on the number of countries that have conducted climate change and health vulnerability and adaptation assessments is also tracked through the WHO Health and Climate Change Global Survey (2021). This voluntary, triennial survey is sent to all WHO Member States and a small number of non-Member territories. The survey is completed by Ministry of Health focal points and often as part of a multistakeholder consultation. The last global survey was published in 2021. Of the 194 WHO member states and non-Member territories, 95 participated in the 2021 global survey, providing representation from all six WHO regions. The 2021 global survey results provided the baseline data for the COP26 commitments with updates on the progress until 2022 coming from regional and country reporting through the ATACH.

Validation of the 2021 country reported global survey data was undertaken in multiple steps. First, survey responses were reviewed for missing information or inconsistencies with follow-up questions directed to survey respondents. A summary of responses was shared with WHO regional focal points and key informants for review, comments, and validation. Source documents including national health strategies and plans, and climate change and health vulnerability and adaptation assessments were collected. A desktop review of these source documents was conducted to compare with survey results with follow-up to survey respondents to seek clarification or additional documentation. Findings were also cross referenced with existing external publications. Data detailing all the ministries, institutions and national stakeholders that provided contributions to or review of the survey responses were collected in order to provide insight into the national consultation process of each survey submission. Of the 95 country submissions, 69 surveys were completed in consultation with one to six different stakeholders, ministries, or institutions. Five countries consulted between 10 to 12 stakeholders, ministries, or institutions. 15 countries did not consult with other entities or health programmes. Information was not available for the remaining six countries. Finally, all respondents reviewed and acknowledged the WHO data policy statement on the use and sharing of data collected by WHO in Member States outside the context of public health emergencies.

Of note, due to the ongoing pandemic, the standard data collection procedures were modified to reduce reporting burden on countries that wished to participate in the global survey but that were facing human resource constraints due to pandemic response. In eight cases, WHO prepared pre-filled survey questionnaires

with data provided by ministries of health in the previous 2018 survey cycle or using data the countries had published in the 2020/2021 WHO UNFCCC health and climate change country profile when available. These countries were requested to review, revise, and complete the hard copy questionnaires. These hard copy questionnaires were then entered into the online platform by WHO. The same data validation steps as described above were then followed. Additionally, a number of countries requested an extension of the reporting period.

As such, there may be a slight increase in the total number of participating countries and the WHO Health and Climate Change Global Survey Report and associated dynamic data dashboard will provide the definitive summary of findings.

Further information on the WHO Health and Climate Change Global Survey, its methodology, and the WHO UNFCCC Health and Climate Change Country Profile Project can be found at https://www.who.int/activities/monitoring-science-and-evidence-on-climate-change-and-health/health-andclimate-global-survey

The WHO questionnaire asks countries whether they have conducted a climate change and health vulnerability and adaptation assessment, defined as "a process and a tool that allows countries to evaluate which populations are most vulnerable to different kinds of health effects from climate change, to identify weaknesses in the systems that should protect them, and to specify interventions to respond. Assessments can also improve evidence and understanding of the linkages between climate and health within the assessment area, serve as a baseline analysis against which changes in disease risk and protective measures can be monitored, provide the opportunity for building capacity, and strengthen the case for investment in health protection".

More information on climate change and health vulnerability and adaptation assessments, can be found in: https://www.who.int/teams/environment-climate-change-and-health/climate-change-and-health/capacitybuilding/toolkit-on-climate-change-and-health/vulnerability

Data

1. Alliance for Transformative Action on Climate Change and Health (ATACH)[161]
2. 2021 WHO Health and Climate Change Global Survey.[162]

Caveats

The global survey sample is not a representative sample of all countries as this survey was voluntary, however, the inclusion of 95 countries in this survey, despite a global pandemic, demonstrates significant global coverage.

Data for this indicator represents the total number of countries that had made a commitment to the COP26 Health Programme as of April 30, 2023. For the most recent list of country commitments please see the ATACH website at: https://www.who.int/initiatives/alliance-for-transformative-action-on-climate-and-health/country-commitments.[161]

Additional analysis

Full list of countries participating in the 2021 WHO Health and Climate Change Global Survey: Argentina, Azerbaijan, Bahamas, Bahrain, Barbados, Belize, Benin, Bhutan, Bolivia (Plurinational Bolivia State of), Brazil, British Virgin Islands, Brunei Darussalam, Bulgaria, Cabo Verde, Cambodia, Cameroon, Canada, China, Colombia, Comoros, Costa Rica, Côte d'Ivoire, Croatia, Cuba, Cyprus, Czech Republic, Dominica, Dominican Republic, Egypt, El Salvador, Eritrea, Estonia, Ethiopia, Germany, Ghana, Grenada, Guatemala, Guinea, Guyana, Haiti, India, Iran (Islamic Republic of), Israel, Italy, Jamaica, Jordan, Kazakhstan, Kenya, Kyrgyzstan, Lebanon, Lithuania, Madagascar, Malawi, Marshall Islands, Micronesia (Federated States of), Mozambique, Netherlands, Nicaragua, Nigeria, North Macedonia, Occupied Palestinian Territory, Oman, Palau, Papua New Guinea, Paraguay, Peru, Philippines, Poland, Portugal, Republic of Moldova, Rwanda, Saint Kitts and Nevis, Saint Lucia, San Marino, Sao Tome And Principe, Saudi Arabia, Serbia, Seychelles, Sierra Leone, Slovakia, South Africa, Sri Lanka, Suriname, Sweden, Thailand, Togo, Trinidad and Tobago, Turkmenistan, United Republic of Tanzania, United States of America, Uruguay, Vanuatu, Yemen, Zambia, Zimbabwe.

COP26 Health Programme

Supported by the UK government, as the Presidency of COP26, the World Health Organization (WHO) and other partners, the COP26 Health Programme enables transformational change to protect the health of people and the planet.

Initiatives under the COP26 Health Programme include:

- Building climate resilient health systems
- Developing low carbon sustainable health systems
- Adaptation Research for Health
- The inclusion of health priorities in Nationally Determined Contributions
- Raising the voice of health professionals as advocates for stronger ambition on climate change

Two of the Programme's key initiatives support countries in developing Climate Resilient and Low Carbon Sustainable Health Systems, with countries expected to announce their commitments to these initiatives by COP26 in November 2021. Commitments are anticipated to be implemented in the coming years and will allow countries to develop a roadmap for future investments in climate resilient and low carbon sustainable health systems and facilities.

Commitment 1: Climate resilient health systems

- Commit to conduct climate change and health vulnerability and adaptation assessments (V&As) at population level and/or health care facility level by a stated target date
- Commit to develop a health National Adaptation Plan informed by the health V&A, which forms part of the National Adaptation Plan to be published by a stated target date
- Commit to use the V&A and HNAP to facilitate access to climate change funding for health (e.g., project proposals submitted to the Global Environmental Facility, Green Climate Fund, Adaptation Fund, or GCF Readiness programme).

Commitment 2: Sustainable low carbon health systems

- High ambition/high emitters: Commit to set a target date by which to achieve health system net zero emissions (ideally by 2050)
- All countries: Commitment to deliver a baseline assessment of greenhouse gas emissions of the health system (including supply chains)

All countries: Commit to develop an action plan or roadmap by a set date to develop a sustainable low carbon health system (including supply chains) which also considers human exposure to air pollution and the role the health sector can play in reducing exposure to air pollution through its activities and its actions

Indicator 2.1.2: National Adaptation Plans for Health

Indicator authors

Dr Diarmid Campbell-Lendrum, Tara Neville

Methods

Data for this indicator was obtained from two sources. First, the number of countries that have committed to the COP26 Health Programme initiative to build a climate resilient health system [162] are tracked through the Alliance for Transformative Action on Climate and Health (ATACH)[162]. The commitment to build a climate resilient health system includes three key components: 1) conducting a climate change and health vulnerability and adaptation (V&A) assessment; 2) developing a health national adaptation plan (HNAP) informed by the results of the climate and health V&A assessment; and using the V&A and HNAP to facilitate access to climate change funding for health. Progress on the COP26 commitments are tracked through regular reporting to the ATACH. Data for this indicator represents the total number of countries that had made a commitment to the COP26 Health Programme as of April 30, 2023. For the most recent list of country commitments please see the

ATACH website at: https://www.who.int/initiatives/alliance-for-transformative-action-on-climate-and-health/country-commitments.[161]

Global progress on the number of countries that have completed a health national adaptation plan (HNAP) is also tracked through the WHO Health and Climate Change Global Survey (2021). This voluntary, triennial survey is sent to all WHO Member States and a small number of non-Member territories. The survey is completed by Ministry of Health focal points and often as part of a multistakeholder consultation. The last global survey was published in 2021. Of the 194 WHO member states and non-Member territories, 95 participated in the 2021 global survey, providing representation from all six WHO regions. The 2021 global survey results provided the baseline data for the COP26 commitments with updates on the progress until 2022 coming from regional and country reporting through the ATACH.Validation of the 2021 country reported global survey data was undertaken in multiple steps. First, survey responses were reviewed for missing information or inconsistencies with follow-up questions directed to survey respondents. A summary of responses was shared with WHO regional focal points and key informants for review, comments, and validation. Source documents including national health strategies and plans, and climate change and health vulnerability and adaptation assessments were collected. A desktop review of these source documents was conducted to compare with survey results with follow-up to survey respondents to seek clarification or additional documentation. Findings were also cross referenced with existing external publications. Data detailing all the ministries, institutions and national stakeholders that provided contributions to or review of the survey responses were collected in order to provide insight into the national consultation process of each survey submission. Of the 95 country submissions, 69 surveys were completed in consultation with one to six different stakeholders, ministries, or institutions. Five countries consulted between 10 to 12 stakeholders, ministries, or institutions. 15 countries did not consult with other entities or health programmes. Information was not available for the remaining six countries. Finally, all respondents reviewed and acknowledged the WHO data policy statement on the use and sharing of data collected by WHO in Member States outside the context of public health emergencies.

Of note, due to the ongoing pandemic, the standard data collection procedures were modified to reduce reporting burden on countries that wished to participate in the global survey but that were facing human resource constraints due to pandemic response. In eight cases, WHO prepared pre-filled survey questionnaires with data provided by ministries of health in the previous 2018 survey cycle or using data the countries had published in the 2020/2021 WHO UNFCCC health and climate change country profile when available. These countries were requested to review, revise, and complete the hard copy questionnaires. These hard copy questionnaires were then entered into the online platform by WHO. The same data validation steps as described above were then followed. Additionally, a number of countries requested an extension of the reporting period. As such, there may be a slight increase in the total number of participating countries and the WHO Health and Climate Change Global Survey Report and associated dynamic data dashboard will provide the definitive summary of findings.

Further information on the WHO Health and Climate Change Global Survey, its methodology, and the WHO UNFCCC Health and Climate Change Country Profile Project can be found at https://www.who.int/activities/monitoring-science-and-evidence-on-climate-change-and-health/health-andclimate-global-survey

The questionnaire asks whether countries have a national health and climate change plan/strategy in place, defined as "a government plan or strategy which considers the health risks of climate change, and health adaptation and/or health resilience to climate change. It could be part of a broader national climate change plan/strategy that includes health". If they have it, countries are requested to upload the plan documentation.

More information on health national adaptation plans (HNAPS), can be found here: https://www.who.int/teams/environment-climate-change-and-health/climate-change-and-health/capacitybuilding/toolkit-on-climate-change-and-health/adaptation

Data

1. Alliance for Transformative Action on Climate Change and Health (ATACH)[161]
2. 2021 WHO Health and Climate Change Global Survey.[162]

Caveats

The global survey sample is not a representative sample of all countries as this survey was voluntary, however, the inclusion of 95 countries in this survey, despite a global pandemic, demonstrates significant global coverage.

Data for this indicator represents the total number of countries that had made a commitment to the COP26 Health Programme as of April 30, 2023. For the most recent list of country commitments please see the ATACH website at: https://www.who.int/initiatives/alliance-for-transformative-action-on-climate-and-health/country-commitments.[161]

Future form of the indicator

The WHO Health and Climate Change Global Survey is a triennial survey and will continue to be the primary source of data to track this indicator.

The future evolution of this indicator will explore the use of evidence (particularly findings from vulnerability and adaptation assessments) to inform the development of strategies/plans and progress on level of implementation of strategies/plans. With more countries initiating the national adaptation plan (NAP) process, alignment of the health component with the overall NAP will also be more closely monitored and examined. Interim information regarding the specific content of national strategies/plans, as explored in this qualitative analysis, may be re-assessed in the future.

Additional analysis

Full list of countries participating in the 2021 WHO Health and Climate Change Global Survey: Argentina, Azerbaijan, Bahamas, Bahrain, Barbados, Belize, Benin, Bhutan, Bolivia (Plurinational Bolivia State of), Brazil, British Virgin Islands, Brunei Darussalam, Bulgaria, Cabo Verde, Cambodia, Cameroon, Canada, China, Colombia, Comoros, Costa Rica, Côte d'Ivoire, Croatia, Cuba, Cyprus, Czech Republic, Dominica, Dominican Republic, Egypt, El Salvador, Eritrea, Estonia, Ethiopia, Germany, Ghana, Grenada, Guatemala, Guinea, Guyana, Haiti, India, Iran (Islamic Republic of), Israel, Italy, Jamaica, Jordan, Kazakhstan, Kenya, Kyrgyzstan, Lebanon, Lithuania, Madagascar, Malawi, Marshall Islands, Micronesia (Federated States of), Mozambique, Netherlands, Nicaragua, Nigeria, North Macedonia, Occupied Palestinian Territory, Oman, Palau, Papua New Guinea, Paraguay, Peru, Philippines, Poland, Portugal, Republic of Moldova, Rwanda, Saint Kitts and Nevis, Saint Lucia, San Marino, Sao Tome And Principe, Saudi Arabia, Serbia, Seychelles, Sierra Leone, Slovakia, South Africa, Sri Lanka, Suriname, Sweden, Thailand, Togo, Trinidad and Tobago, Turkmenistan, United Republic of Tanzania, United States of America, Uruguay, Vanuatu, Yemen, Zambia, Zimbabwe.

-

Indicator 2.1.3: City-Level Climate Change Risk Assessments

Indicator authors

Prof Karyn Morrisey

Methods

Indicator 2.1.3 captures data on at the city level on:

1. cities that have undertaken a climate change risk or vulnerability assessment and;
2. the perceived vulnerability city leaders of their public health assets to climate change

Data

1. 2022 CDP Annual Cities Survey[163]

Caveats

This is a self-reported survey, non-compulsory survey as such data provided may be subjective and response rates can fluctuate, with low uptake in certain areas, particularly the Middle East.

Future form of the indicator

The CDP collect this data annually and it is foreseen that the data collection will continue to 2030. Additional analyses may be conducted using data from the CDP annual survey to monitor associations between city-level health vulnerabilities and track reporting trends over time.

Additional analysis

Table 30 Cities responses to climate change and health questions

**Details of Health outcomes calculation**			
Health **outcomes**	*351	525	0.66857143
Health **systems**	*141	525	0.26857143
Areas **outside** the health sector	*33	525	0.06285714
cities responding CC impacts Health	525		
**Unable** to measure climate change related health impacts	*140		
Do not know (**DNK**)	*22		
cities responding to this question: adjusted for duplicates:	*670		
*Adjusted for duplicate city records in data: see stata file			
**Overall Cities Responding CC is impacting health**			
cities responding to survey overall	**986**		
CC does not impact (null RESPONSES)	**316**		
Overall cities responding CC impacts Health.	53% (525/986)		

Table 31 Cities climate change assessment by HDI grouping

**Climate Change Assessment by HDI**	Number.	Percent
**Low HDI**		
No, and we are not intending to undertake due to lack of expertise/technical capacity	2	9%
No, and we are not intending to undertake due to lack of financial capacity	1	4%
No, but we are currently undertaking one and it will be complete in the next year	2	9%
No, but we are intending to undertake one in the next two years	4	17%
Yes, a climate risk and vulnerability assessment has been undertaken	14	61%
Total	23	
**Medium HDI**		
No, and we are not intending to undertake due to lack of expertise/technical capacity	2	3%
No, and we are not intending to undertake due to lack of financial capacity	3	4%
No, and we are not intending to undertake due to lack of financial capacity and expertise/technical capacity	4	5%

No, but we are currently undertaking one and it will be complete in the next year	9	12%
No, but we are intending to undertake one in the next two years	9	12%
Yes, a climate risk and vulnerability assessment has been undertaken	50	65%
Total	77	
**High HDI**		
No, and we are not intending to undertake due to other higher priorities	2	1%
No, and we are not intending to undertake due to lack of expertise/technical capacity	5	2%
No, and we are not intending to undertake due to lack of financial capacity	6	2%
No, and we are not intending to undertake due to lack of financial capacity and expertise/technical capacity	9	4%
No, and we are not intending to undertake due to other priorities	2	1%
No, but we are currently undertaking one and it will be complete in the next year	24	10%
No, but we are intending to undertake one in the next two years	54	22%
Yes, a climate risk and vulnerability assessment has been undertaken	144	59%
Total	246	
**Very High HDI**		
No, and we are not intending to undertake due to other higher priorities	1	0%
No, and we are not intending to undertake due to lack of expertise/technical capacity	3	1%
No, and we are not intending to undertake due to lack of financial capacity	1	0%
No, and we are not intending to undertake due to lack of financial capacity and expertise/technical capacity	6	1%
No, and we are not intending to undertake due to other priorities	2	0%
No, but we are currently undertaking one and it will be complete in the next year	39	7%
No, but we are intending to undertake one in the next two years	58	11%
Yes, a climate risk and vulnerability assessment has been undertaken	440	80%
Total	550	

Table 32 Cities question on undertaking climate change assessment in Low and Medium HDI Countries

Reason Climate Change assessment not undertaken in Low and Middle HDI Countries		
No, and we are not intending to undertake due to other higher priorities	4	8%
No, and we are not intending to undertake due to lack of financial capacity and expertise/technical capacity	20	40%
No, and we are not intending to undertake due to lack of financial capacity	11	22%
No, and we are not intending to undertake due to lack of expertise/technical capacity	12	24%
other	3	6%
	50	

2.2: Enabling Conditions, Adaptation Delivery and Implementation

Indicator 2.2.1: Climate Information for Health

Indicator authors

Dr Joy Shumake-Guillemot

Methods

The number of World Meteorological Organization (WMO) national meteorological and hydrological services (NMHS) providing climate services to the health sector is calculated based on self-reported information provided by NMHS through the Country Profile Database Integrated questionnaire. The questionnaire is one of the main sources of information to the WMO Country Profile database and is open all year round for WMO members to update their profile information.

Reported data reflects answers to Question number 7.6 of this questionnaire: “Please indicate which user communities/sectors your NMHS provides with climate products/information and estimate the extent to which these products are used to improve decisions”. “Human Health” is one of multiple sectors which can be chosen.

Data

1. World Meteorological Organization Country Profile database.[164]

Caveats

The current data source from WMO only considers climate services provided by NMHS. It is unclear the degree to which other providers, such as academic institutions and research projects, private sector products, products from other Ministries, or regional and global products and services are being used, in proportion to services made available by NMHS.

The open questionnaire can be updated at any time by WMO members, therefore the figures reported here may change over the year. As each country may update their profile information at different moments in time, snapshots do not reflect progress for any given year but rather information provided until a certain date.

The current questionnaire does not record the number of WMO members that do not provide climate services to the health sector.

The questionnaire captures information on the provision of climate services, the status of service provision to the health sector and the type of services provided (divided in 5 categories). Questions do not capture the source or quality of the service and only one of the answer options covers the utility of the climate services. They do not capture whether data originates from national meteorological observations or is resulting from regional or global products. They do not capture the potential use of all-sector forecasts or outlooks which are accessed and used by the health sector.

The WMO and WHO have some differences in their individual Member States. Responses collected from WMO Member States were reclassified according to WHO Region. WMO members that are not individual WHO members were excluded from the analyses and include Macao and Hong Kong (reported as China), Curaçao, French Polynesia, and St. Maartens. The following WHO Members are not members of WMO (and therefore representative data is not available): Andorra, Equatorial Guinea, Marshall Islands, Nauru, Palau, San Marino.

Future form of the indicator

In 2019, WMO began implementation of new survey instruments to provide greater insight on the status of climate service provision for the health sector and the type of service provided. Other complementary WMO surveys capturing specific product types, user satisfaction, and application areas, may be publicly available in the future to inform future editions of this indicator.

The WHO Health and Climate Change Country Survey now contains indicators on the inclusion of meteorological information in integrated risk monitoring and early warning systems for climate-sensitive diseases. This information may be used to improve this indicator in future publications.

Indicator 2.2.2: Air Conditioning: Benefits and Harms

Indicator authors

Prof Robert Dubrow

Methods

Premature deaths from ambient PM $_{2.5}$ exposure due to electricity use for air conditioning.

To estimate country/region-specific premature deaths from ambient $PM_{2.5}$ exposure due to electricity use for air conditioning, the proportion of total electricity final consumption used for air conditioning (obtained from IEA) was multiplied by the estimated country/region-specific premature deaths due to $PM_{2.5}$ emissions from electric power plants, taken from Indicator 3.3. Indicator 3.3 estimated premature deaths from ambient $PM_{2.5}$ exposure for 140 countries. To calculate premature deaths from ambient $PM_{2.5}$ exposure for each IEA-defined region, premature deaths from ambient $PM_{2.5}$ exposure across the countries classified into each region were summed.

Data

The IEA kindly provided data for 2000–2021, including revisions based on improved IEA analyses of its 2000–2020 data used in the 2022 *Lancet* Countdown report. These data included the proportion of households with air conditioning; total electricity final consumption used for air conditioning (TWh); CO_2 emissions due to air conditioning (megatons); and proportion of total electricity final consumption used for air conditioning (used in the calculation of premature deaths from ambient $PM_{2.5}$ exposure due to electricity use for air conditioning).

Proportion of total electricity final consumption used for air conditioning was provided for 12 individual countries and for 14 IEA-defined regions that did not include the 12 individual countries. The countries and regions together constituted the entire world.

The following are the 12 individual countries: Canada, Brazil, China, India. Indonesia, Japan, Mexico, Republic of Korea, Russian Federation, South Africa, United Kingdom, United States

The following are the 14 regions (the 12 individual countries were not included in the regions):

1. Caspian: Armenia, Azerbaijan, Georgia, Kazakhstan, Kyrgyzstan, Tajikistan, Turkmenistan, Uzbekistan
2. European Union A: Italy, France, Germany
3. European Union B: Austria, Belgium, Czechia, Denmark, Estonia, Finland, Greece, Hungary, Ireland, Latvia, Lithuania, Luxembourg, Netherlands, Poland, Portugal, Slovakia, Slovenia, Spain, Sweden
4. European Union C: Bulgaria, Croatia, Cyprus, Malta, Romania
5. Other Europe A: Iceland, Israel, Norway, Switzerland, Turkey
6. Other Europe B: Albania, Belarus, Bosnia and Herzegovina, Gibraltar, Holy See, Kosovo, Montenegro, North Macedonia, Republic of Moldova, Serbia, Ukraine
7. North Africa: Algeria, Egypt, Libya, Morocco, Tunisia
8. Other Africa: Angola, Benin, Botswana, Burkina Faso, Burundi, Cabo Verde, Cameroon, Central African Republic, Chad, Comoros, Congo, Côte d'Ivoire, Democratic Republic of the Congo, Djibouti, Equatorial Guinea, Eritrea, Eswatini, Ethiopia, Gabon, Gambia, Ghana, Guinea, Guinea-Bissau, Kenya, Lesotho, Liberia, Madagascar, Malawi, Mali, Mauritania, Mauritius, Mozambique, Namibia, Niger, Nigeria, Réunion, Rwanda, Sao Tome and Principe, Senegal, Seychelles, Sierra Leone, Somalia, South Sudan, Sudan, Togo, Uganda, United Republic of Tanzania, Zambia, Zimbabwe

9. Chile and Colombia: Chile, Colombia
10. Other Latin America: Antigua and Barbuda, Argentina, Aruba, Bahamas, Barbados, Belize, Bermuda, Bolivia, Bolivarian Republic of Venezuela, British Virgin Islands, Cayman Islands, Costa Rica, Cuba, Curaçao, Dominica, Dominican Republic, Ecuador, El Salvador, Falkland Islands (Malvinas), French Guiana, Grenada, Guadeloupe, Guatemala, Guyana, Haiti, Honduras, Jamaica, Martinique, Montserrat, Caribbean Netherlands, Nicaragua, Panama, Paraguay, Peru, Saint Kitts and Nevis, Saint Lucia, Saint Pierre and Miquelon, Saint Vincent and the Grenadines, Sint Maarten (Dutch part), Suriname, Trinidad and Tobago, Turks and Caicos Islands, Uruguay
11. Middle East: Bahrain, Iraq, Islamic Republic of Iran, Jordan, Kuwait, Lebanon, Oman, Qatar, Saudi Arabia, Syrian Arab Republic, United Arab Emirates, Yemen
12. Association of Southeast Asian Nations (ASEAN) countries: Brunei Darussalam, Cambodia, Lao People's Democratic Republic, Malaysia, Myanmar, Philippines, Pitcairn Island, Singapore, Thailand, Vietnam
13. Other Asia: Afghanistan, Bangladesh, Bhutan, Cook Islands, Democratic People's Republic of Korea, Fiji, French Polynesia, Kiribati, Macao SAR (China), Maldives, Mongolia, Nepal, New Caledonia, Pakistan, Palau, Papua New Guinea, Samoa, Solomon Islands, Sri Lanka, Taiwan, Timor-Leste, Tonga, Vanuatu
14. Australia and New Zealand: Australia, New Zealand

The following countries were not included in the calculation of IEA-defined-region-level number of premature deaths due to $PM_{2.5}$ emissions from electric power plants because, although they were included in IEA regions, they were not included in the assessment of number of premature deaths due to $PM_{2.5}$ emissions from electric power plants in Indicator 3·3:

Other Europe B: Gibraltar, Holy See, Kosovo

Other Africa: Réunion, Sao Tome and Principe, Seychelles, South Sudan

Other Latin America: Antigua and Barbuda, Aruba, Bahamas, Barbados, Belize, Bermuda, British Virgin Islands, Caribbean Netherlands, Cayman Islands, Costa Rica, Cuba, Curaçao, Dominica, Dominican Republic, El Salvador, Falkland Islands (Malvinas), French Guiana, Grenada, Guadeloupe, Guatemala, Guyana, Haiti, Honduras, Jamaica, Martinique, Montserrat, Nicaragua, Panama, Saint Kitts and Nevis, Saint Lucia, Saint Pierre and Miquelon, Saint Vincent and the Grenadines, Sint Maarten (Dutch part), Suriname, Trinidad and Tobago, Turks and Caicos Islands

Middle East: Bahrain, Iraq, Jordan, Kuwait, Lebanon, Oman, Qatar, Syrian Arab Republic, United Arab Emirates, Yemen

ASEAN countries: Pitcairn Island

Other Asia: Cook Islands, Fiji, French Polynesia, Kiribati, Macao SAR (China), Maldives, New Caledonia, Palau, Papua New Guinea, Samoa, Solomon Islands, Taiwan, Timor-Leste, Tonga, Vanuatu

Caveats

Estimate of number of premature deaths due to $PM_{2.5}$ emissions from air conditioning:

To estimate the number of premature deaths due to $PM_{2.5}$ emissions from air conditioning, the finer the spatial resolution, the more accurate the estimates. The data available for proportion of total electricity final consumption used for air conditioning were at the country or region level. Thus, in a given country/region, it was by necessity assumed that the electricity market is completely connected, so that the share of electricity used for air conditioning can be equally applied to power plant emissions throughout the country/region. This assumption may not be accurate, especially for larger countries/regions.

Notably, the sustainability of air conditioning could be increased through generation of electricity by renewable energy and more efficient air conditioning technology. These measures would reduce both CO_2 emissions and the number of premature deaths due to $PM_{2.5}$ emissions from air conditioning. Greenhouse gas emissions from air conditioning could be further reduced through phase-out of hydrochlorofluorocarbon refrigerants in favour of

refrigerants that are not greenhouse gases, as called for in the 2016 Kigali Amendment to the Montreal Protocol. Technology to capture and recycle waste heat would make air conditioning even more efficient and would reduce its contribution to the urban heat island effect.

Future form of the indicator

The indicator in the 2021 report estimated the number of heat-related deaths averted by air conditioning in the 65-and-older population by country/region and for the world. There were a number of limitations to these estimates, such that they were considered to be “ballpark” estimates that would need considerable refinement in future years. The intention is to present improved estimates in future years, including all age groups. In addition, city-level case studies to estimate number of lives saved from air conditioning versus premature deaths from exposure to $PM_{2.5}$ due to air conditioning may be performed. The indicator may be updated each year as new data become available on air conditioning use. Trends in vulnerability to heatwave-related mortality could be assessed with cooling degree days. Finally, metrics related to more efficient cooling (e.g., national building codes, minimum energy performance standards, labelling rules for air conditioners) and progress on implementing the Kigali Amendment may be tracked in the future.

Indicator 2.2.3: Urban Green Space

Indicator authors

Prof Patrick Kinney, Dr Jennifer D. Stowell

Methods

Urban centre spatial extents were defined by the Global Human Settlement (GHS) program of the European commission.[165] The GHS uses remote sensing and demographic data to define more than 10,000 urban centres worldwide. Urban centres chosen for the indicator were identified as urban centres larger than 500,000 inhabitants. To include countries with no urban centres that met this threshold, we selected the most populated city where possible, giving a final count of 1,041 urban centres and 174 countries. Due to missing data in either the GHS or the Normalized Difference Vegetation Index (NDVI) data, 22 countries (mostly small island states) were not represented in the analysis.

Data on population size for all years were collected from the Center for International Earth Science Information Network (CIESN, Columbia University), which models the distribution of human population at 30 arc-second output resolution.[166]

Green space was estimated using the normalized difference vegetation index (NDVI), the most commonly used satellite-based vegetation index. NDVI calculates the ratio of the differences between near infrared radiation and visible radiation to the sum of these two measures. NDVI values range from -1·0 to 1·0 with values less than 0 indicating water and values close to 1 indicating high levels of vegetation density.[167] For this process, we used publicly available data from the Landsat satellite, a joint program of the USGS and NASA.[168] Landsat images the Earth’s surface at 30-meter resolution approximately every two weeks (~16 days). To account for seasonal fluctuations, we computed NDVI for each of the following time periods (with season labels based on the northern hemisphere):

- Winter—December 1 of previous year through February 28
- Spring—March 1 through May 31
- Summer—June 1 through August 31
- Fall—September 1 through November 30

We did this for four different years: 2015, 2020, 2021, and 2022. In previous iterations of this indicator, 2010 was also included. However, due to a known equipment malfunction in Landsat7, we have elected to remove 2010 from this update. Landsat 8 (2015, 2020, 2021) and Landsat 9 (2022) were used to estimate values for the included years. For each year and city, a total of four exposure metrics were calculated: peak NDVI (maximum NDVI across the four seasons); annual mean NDVI based on the four-season average NDVI; population-weighted average peak NDVI; and population-weighted mean NDVI. The population weighted NDVI was computed for each city by multiplying each NDVI value (peak and four-season average) by the population size

of the corresponding year within the same 1x1 km raster, summing up over the weighted values within the urban extent, and dividing by the sum of the weights, as shown by the equation below:

$$\frac{\sum_{i=1}^{n}(NDVI_i * population_i)}{\sum_{i=1}^{n}(population_i)}$$

Additional analyses include subsetting the data by levels of the Human Development Index (HDI, see Figure 1), climate regions as defined by the Köppen Climate Classification System (see Figure 2), *Lancet* Countdown regional country groupings, and WHO region.[169] Google Earth Engine was used to generate raw data and the R Statistical Software was used for data analysis and management and to compute the four metrics described above. 'Level of Greenness' was defined according to the table below:

Table 33 Categorization of Greenness Levels

Level of Greenness	Population-Weighted Peak NDVI
**Exceptionally Low**	<0·20
**Very low**	0·20-0·29
**Low**	0·30-0·39
**Moderate**	0·40-0·49
**High**	0·50-0·59
**Very High**	0·60-0·69
**Exceptionally High**	≥0·70

Data

1. Global Human Settlement Programme of the European Commission (GHS) used to identify urban centres.[165]
2. Population size identified from NASA GPWv4.[170]
3. Satellite data were downloaded from the publicly available Landsat satellite, a joint program of the US Geological Survey and NASA.[168]
4. Global climate regions from the Köppen Climate Classification System.[169]

Caveats

This approach has some limitations. First, although satellite-based measures of vegetation have been used extensively to measure greenness, NDVI does not provide information on the quality of greenness (e.g., curated park vs vacant lot), the type of green space (e.g., park vs. forest), the type of vegetation (e.g., shrubs vs. trees) or social characteristics (e.g., level of security). However, studies have demonstrated that NDVI performs adequately when compared with environmental psychologists' evaluations of green spaces.[171] In addition, reviews of the literature on greenness and health have been undertaken and found consistent and strong evidence of associations of higher greenness measured by NDVI, with improvements in birthweights, physical activity, lower mortality rates, and lower levels of depression.[172] Second, missing values from GHS or from Landsat data due to cloud cover or other factors limit the generalizability of the findings.

Future form of indicator

Future versions of this indicator will continue to examine trends over time and will aim to estimate the proportion of each city that is green space, in addition to the overall average greenness of an urban centre and

we are considering the addition of urban blue space. We will also explore options to integrate the greenness indicator with other indicators to investigate the associations between urban green space and multiple measures, including heat-related exposures and health effects, exposure of vulnerable populations, and loss of physical activity and/or labour capacity.

Additional analysis

Table 34 Global percent moderate or above (population-weighted average peak-season NDVI ≥ 0.40)

Year	% > Moderate Greenness
2015	28%
2020	28%
2021	27%
2022	27%

Table 35 Global average population-weighted peak-season NDVI

Year	Pop-weighted average peak-season NDVI
2015	0.34
2020	0.34
2021	0.34
2022	0.34

Table 36 Population-weighted peak-season NDVI by HDI

HDI-level		2015	2020	2021	2022
**Low**		0.31	0.30	0.29	0.29
**Medium**		0.37	0.37	0.37	0.37
**High**		0.31	0.32	0.31	0.32
**Very High**		0.36	0.36	0.35	0.35

Table 37 Population-weighted peak-season NDVI by climate region

Climate Region	2015	2020	2021	2022
**Arid**	0.24	0.25	0.24	0.24
**Continental**	0.37	0.39	0.38	0.38
**Polar**	0.14	0.13	0.13	0.14
**Temperate**	0.35	0.35	0.35	0.35

**Tropical**	0.39	0.38	0.38	0.38

Table 38 Population-weighted peak-season NDVI by WHO region

WHO Region	2015	2020	2021	2022
**African**	0.33	0.32	0.32	0.31
**Americas**	0.34	0.34	0.33	0.34
**E Mediterranean**	0.22	0.22	0.21	0.20
**European**	0.37	0.37	0.37	0.37
**SE Asian**	0.40	0.40	0.41	0.40
**W Pacific**	0.31	0.32	0.31	0.32

Table 39 Percent moderate or above by HDI (population-weighted average peak-season NDVI ≥0.40)

HDI-level	2015	2020	2021	2022
**Low**	18%	18%	15%	13%
**Medium**	37%	36%	40%	36%
**High**	17%	17%	16%	18%
**Very High**	37%	38%	33%	36%

Table 40 Climate region percent moderate or above (population-weighted average peak-season NDVI ≥0.40)

Climate Region	2015	2020	2021	2022
**Arid**	4%	5%	6%	5%
**Continental**	48%	44%	43%	51%
**Polar**	0%	0%	0%	0%
**Temperate**	28%	27%	26%	24%
**Tropical**	39%	42%	40%	40%

Table 41 Percent moderate or above by WHO region (population-weighted average peak-season NDVI ≥0.40)

WHO Region	2015	2020	2021	2022
**African**	21%	20%	18%	18%
**Americas**	28%	28%	25%	25%
**E Mediterranean**	5%	6%	5%	4%

**European**	44%	45%	40%	44%
**SE Asian**	47%	46%	49%	45%
**W Pacific**	9%	8%	8%	11%

Table 42 Percent moderate or above by Lancet Countdown region (population-weighted average peak-season NDVI ≥0.40)

LCD Region	2015	2020	2021	2022
**African**	17%	16%	15%	14%
**Asia**	24%	24%	25%	24%
**European**	55%	56%	50%	55%
**North American**	60%	58%	49%	51%
**Oceania**	17%	0%	0%	17%
**SIDS**	27%	41%	45%	50%
**South/Central America**	12%	11%	13%	11%

Table 43 Estimates of Urban Green Space by HDI (2015)

HDI-level	Peak NDVI	Four-season NDVI	Pop. weighted Peak NDVI	Pop. weighted Four-season NDVI
**Low**	0.32	0.26	0.31	0.25
**Medium**	0.38	0.31	0.37	0.31
**High**	0.34	0.27	0.31	0.25
**Very High**	0.37	0.29	0.36	0.28
**Global Mean**	0.35	0.29	0.34	0.27

Table 44 Estimates of Urban Green Space by HDI (2020)

HDI-level	Peak NDVI	Four-season NDVI	Pop. weighted Peak NDVI	Pop. weighted Four-season NDVI
**Low**	0.31	0.26	0.30	0.25
**Medium**	0.38	0.32	0.37	0.31
**High**	0.35	0.28	0.32	0.25
**Very High**	0.36	0.29	0.36	0.28
**Global Mean**	0.35	0.29	0.34	0.27

Table 45 Estimates of Urban Green Space by HDI (2021)

HDI-level	Peak NDVI	Four-season NDVI	Pop. weighted Peak NDVI	Pop. weighted Four-season NDVI
**Low**	0.31	0.25	0.30	0.24
**Medium**	0.38	0.31	0.37	0.30
**High**	0.34	0.27	0.31	0.25
**Very High**	0.36	0.28	0.35	0.27
**Global Mean**	0.36	0.28	0.35	0.27

Table 46 Estimates of Urban Green Space by HDI (2022)

HDI-level	Peak NDVI	Four-season NDVI	Pop. weighted Peak NDVI	Pop. weighted Four-season NDVI
**Low**	0.30	0.25	0.29	0.24
**Medium**	0.37	0.31	0.37	0.30
**High**	0.34	0.28	0.32	0.26
**Very High**	0.36	0.28	0.35	0.27
**Global Mean**	0.35	0.28	0.34	0.27

Table 47 Estimates of Urban Green Space by Climate Region (2015)

Climate Region	Peak NDVI	Four-season NDVI	Pop. weighted Peak NDVI	Pop. weighted Four-season NDVI
**Arid**	0.25	0.21	0.24	0.20
**Continental**	0.38	0.26	0.37	0.25
**Polar**	0.17	0.14	0.14	0.12
**Temperate**	0.37	0.30	0.35	0.28
**Tropical**	0.41	0.35	0.39	0.33

Table 48 Estimates of Urban Green Space by Climate Region (2020)

Climate Region	Peak NDVI	Four-season NDVI	Pop. weighted Peak NDVI	Pop. weighted Four-season NDVI
**Arid**	0.26	0.21	0.25	0.20
**Continental**	0.40	0.27	0.39	0.26
**Polar**	0.15	0.13	0.13	0.11
**Temperate**	0.36	0.30	0.35	0.29
**Tropical**	0.40	0.35	0.39	0.33

Table 49 Estimates of Urban Green Space by Climate Region (2021)

Climate Region	Peak NDVI	Four-season NDVI	Pop. weighted Peak NDVI	Pop. weighted Four-season NDVI
**Arid**	0.25	0.21	0.24	0.20
**Continental**	0.39	0.26	0.38	0.25
**Polar**	0.16	0.12	0.13	0.10
**Temperate**	0.36	0.29	0.35	0.28
**Tropical**	0.40	0.34	0.38	0.33

Table 50 Estimates of Urban Green Space by Climate Region (2022)

Climate Region	Peak NDVI	Four-season NDVI	Pop. weighted Peak NDVI	Pop. weighted Four-season NDVI
**Arid**	0.25	0.21	0.24	0.20
**Continental**	0.39	0.27	0.38	0.26
**Polar**	0.17	0.13	0.14	0.11
**Temperate**	0.36	0.30	0.35	0.28
**Tropical**	0.40	0.34	0.38	0.33

Table 51 Estimates of Urban Green Space by WHO region (2015)

WHO Region	Peak NDVI	Four-season NDVI	Pop. weighted Peak NDVI	Pop. weighted Four-season NDVI
**African**	0.35	0.28	0.33	0.26
**Americas**	0.36	0.31	0.34	0.29
**E Mediterranean**	0.23	0.20	0.22	0.19
**European**	0.38	0.29	0.37	0.28
**SE Asian**	0.41	0.34	0.41	0.34
**W Pacific**	0.33	0.25	0.30	0.23

Table 52 Estimates of Urban Green Space by WHO region (2020)

WHO Region	Peak NDVI	Four-season NDVI	Pop. weighted Peak NDVI	Pop. weighted Four-season NDVI
**African**	0.34	0.27	0.32	0.26
**Americas**	0.36	0.30	0.34	0.29
**E Mediterranean**	0.23	0.20	0.22	0.19
**European**	0.38	0.29	0.37	0.28
**SE Asian**	0.41	0.35	0.40	0.34
**W Pacific**	0.34	0.26	0.32	0.24

Table 53 Estimates of Urban Green Space by WHO region (2021)

WHO Region	Peak NDVI	Four-season NDVI	Pop. weighted Peak NDVI	Pop. weighted Four-season NDVI
**African**	0.33	0.27	0.32	0.25
**Americas**	0.36	0.30	0.33	0.28
**E Mediterranean**	0.22	0.19	0.21	0.18
**European**	0.38	0.28	0.37	0.27
**SE Asian**	0.41	0.34	0.41	0.33
**W Pacific**	0.33	0.26	0.31	0.24

Table 54 Estimates of Urban Green Space by WHO region (2022)

WHO Region	Peak NDVI	Four-season NDVI	Pop. weighted Peak NDVI	Pop. weighted Four-season NDVI
**African**	0.33	0.27	0.31	0.25
**Americas**	0.36	0.30	0.34	0.28
**E Mediterranean**	0.22	0.19	0.20	0.17
**European**	0.38	0.28	0.37	0.28
**SE Asian**	0.41	0.34	0.40	0.33
**W Pacific**	0.34	0.26	0.32	0.25

Table 55 Estimates of Urban Green Space by LCD region (2015)

LCD Region	Peak NDVI	Four-season NDVI	Pop. weighted Peak NDVI	Pop. weighted Four-season NDVI
**Africa**	0.33	0.27	0.31	0.25

**Asia**	0.35	0.28	0.34	0.27
**Europe**	0.41	0.30	0.40	0.29
**Northern America**	0.40	0.32	0.39	0.32
**Oceania**	0.34	0.31	0.35	0.31
**SIDS**	0.38	0.34	0.38	0.33
**South & Central America**	0.34	0.30	0.31	0.27

Table 56 Estimates of Urban Green Space by LCD region (2020)

LCD Region	Peak NDVI	Four-season NDVI	Pop. weighted Peak NDVI	Pop. weighted Four-season NDVI
**Africa**	0.32	0.26	0.30	0.24
**Asia**	0.35	0.29	0.34	0.28
**Europe**	0.41	0.31	0.40	0.29
**Northern America**	0.40	0.31	0.40	0.31
**Oceania**	0.33	0.30	0.33	0.30
**SIDS**	0.39	0.34	0.38	0.33
**South & Central America**	0.33	0.29	0.30	0.26

Table 57 Estimates of Urban Green Space by LCD region (2021)

LCD Region	Peak NDVI	Four-season NDVI	Pop. weighted Peak NDVI	Pop. weighted Four-season NDVI
**Africa**	0.31	0.26	0.29	0.24
**Asia**	0.35	0.28	0.34	0.27
**Europe**	0.41	0.30	0.40	0.29
**Northern America**	0.38	0.31	0.38	0.30
**Oceania**	0.35	0.32	0.35	0.32
**SIDS**	0.38	0.35	0.38	0.34
**South & Central America**	0.33	0.29	0.30	0.26

Table 58 Estimates of Urban Green Space by LCD region (2022)

LCD Region	Peak NDVI	Four-season NDVI	Pop. weighted Peak NDVI	Pop. weighted Four-season NDVI
**Africa**	0.31	0.26	0.29	0.24

**Asia**	0.35	0.28	0.34	0.27
**Europe**	0.40	0.30	0.39	0.29
**Northern America**	0.29	0.31	0.39	0.31
**Oceania**	0.34	0.31	0.34	0.31
**SIDS**	0.39	0.35	0.38	0.34
**South & Central America**	0.33	0.29	0.30	0.26

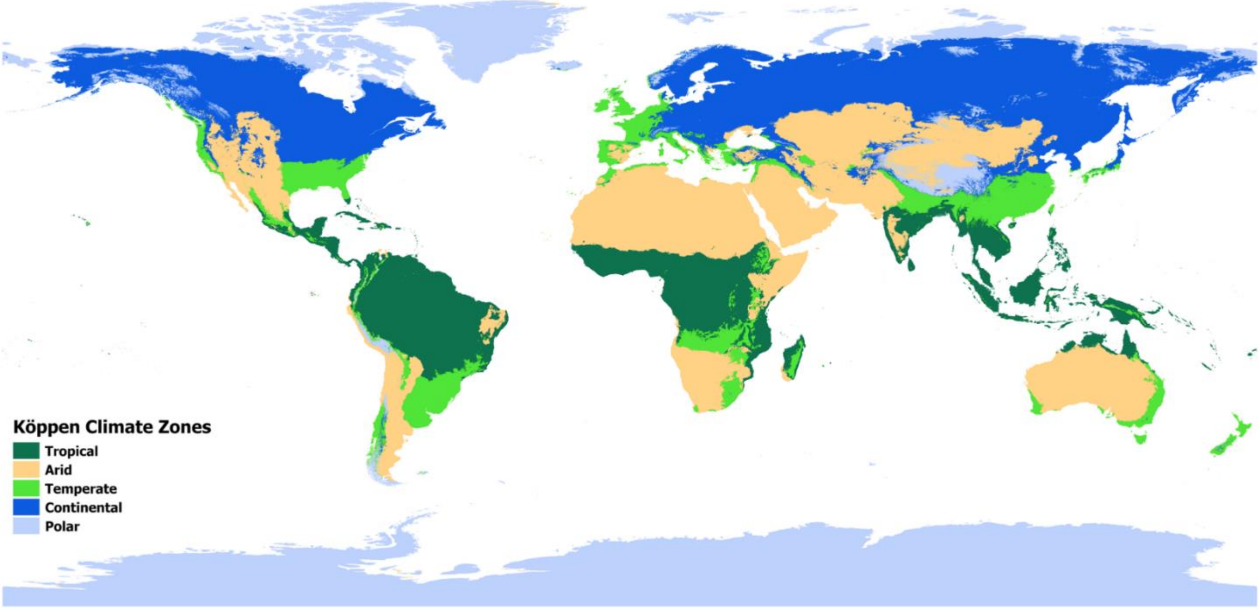

Figure 76 Köppen Climate Regions. Designated climate regions of the world using the Köppen Climate Zones system

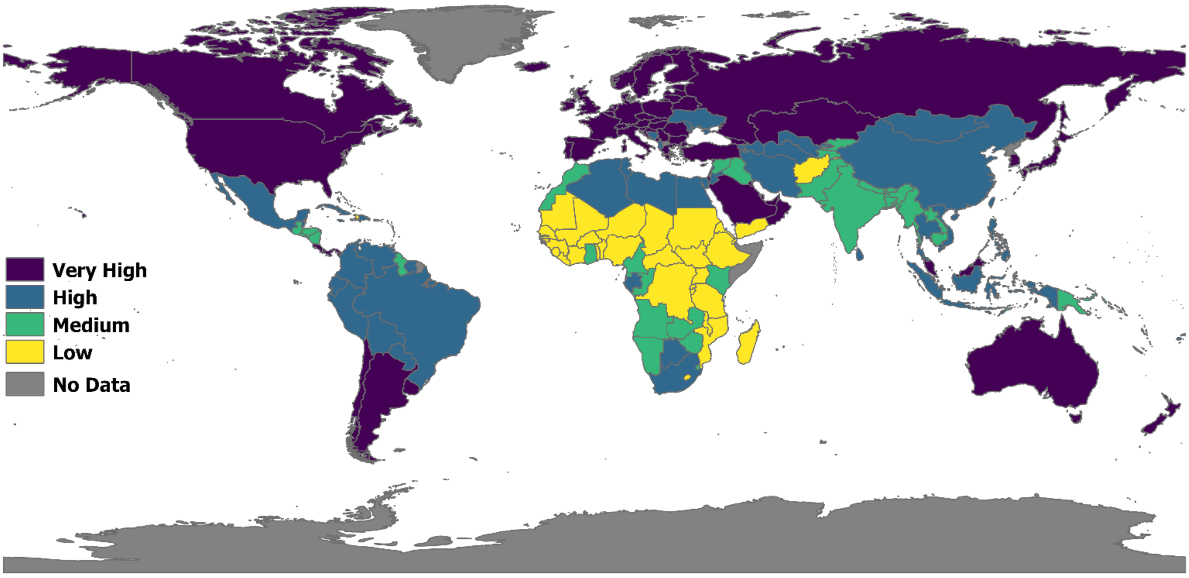

Figure 77 Country Development Level. Development level as denoted by the Human Development Index or HDI.

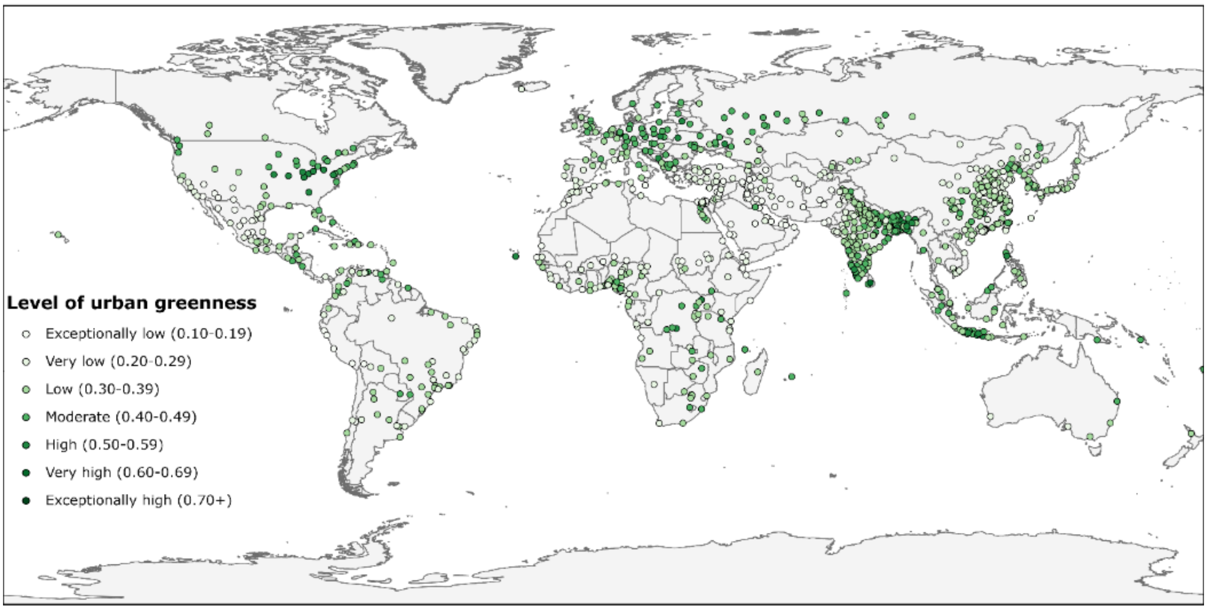

Figure 78 Urban greenness in 1,041 urban centres in 2022.

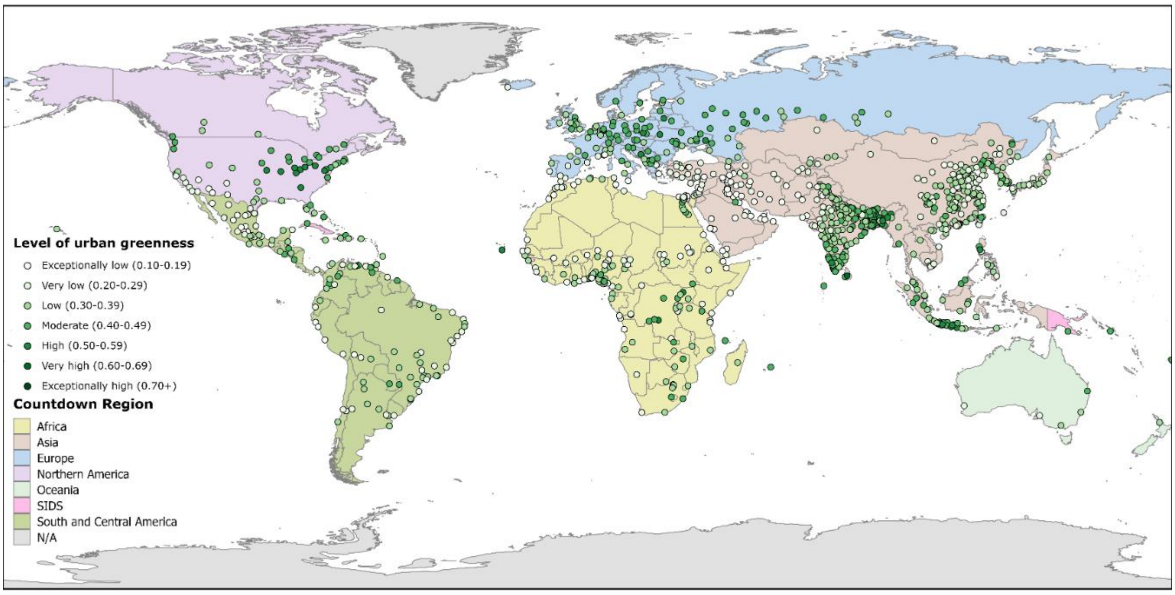

Figure 79 Urban greenness in 1,041 urban centres in 2022. Levels of urban greenness were quantified on the basis of mean population-weighted peak-season normalized difference vegetation index (NDVI). The NDVI is a standard, satellite-based measurement used to estimate vegetation on a scale of -1.0 to 1.0

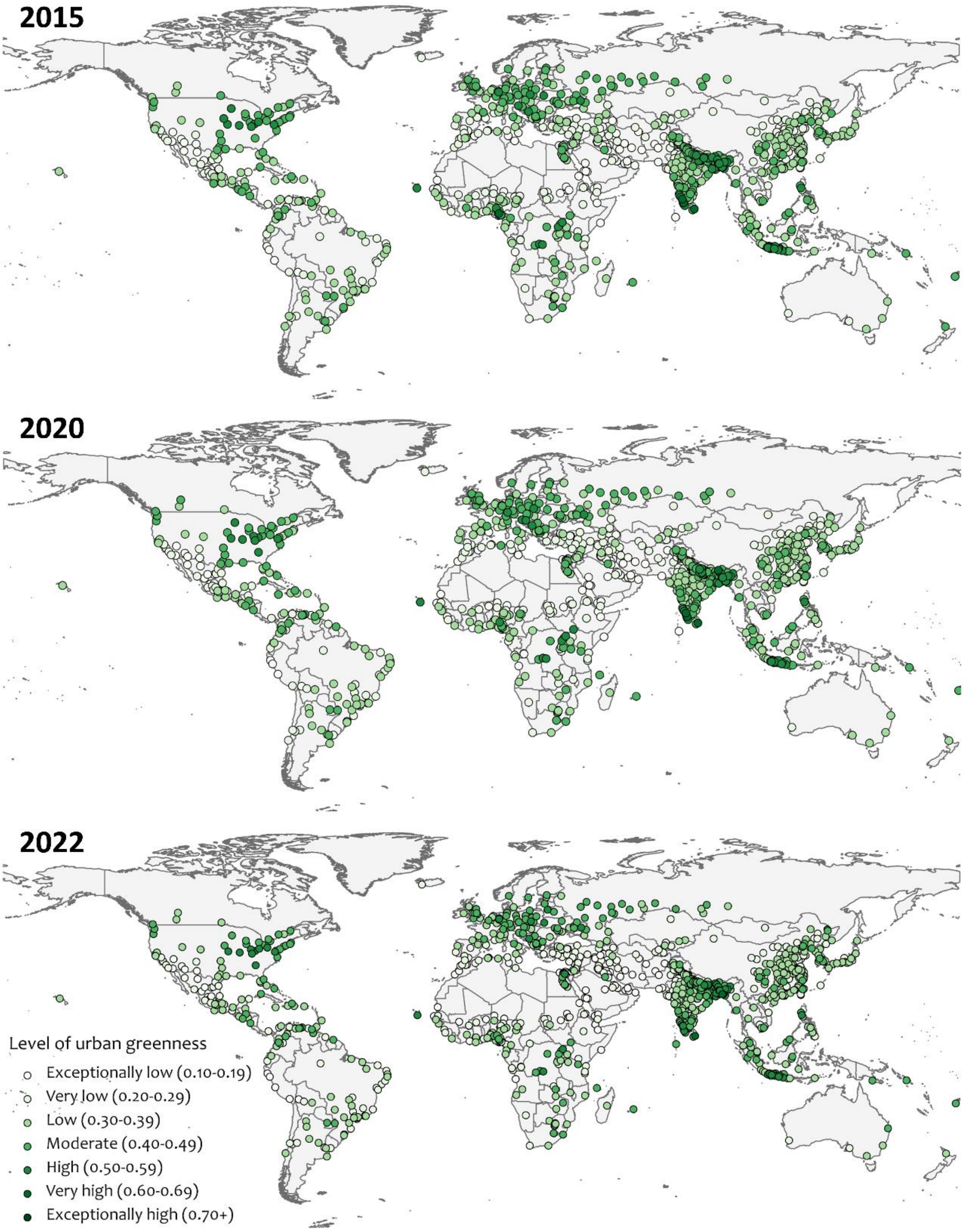

Figure 80 Temporal changes in urban greenness. Levels of urban greenness change between 2015, 2020, 2021, 2022

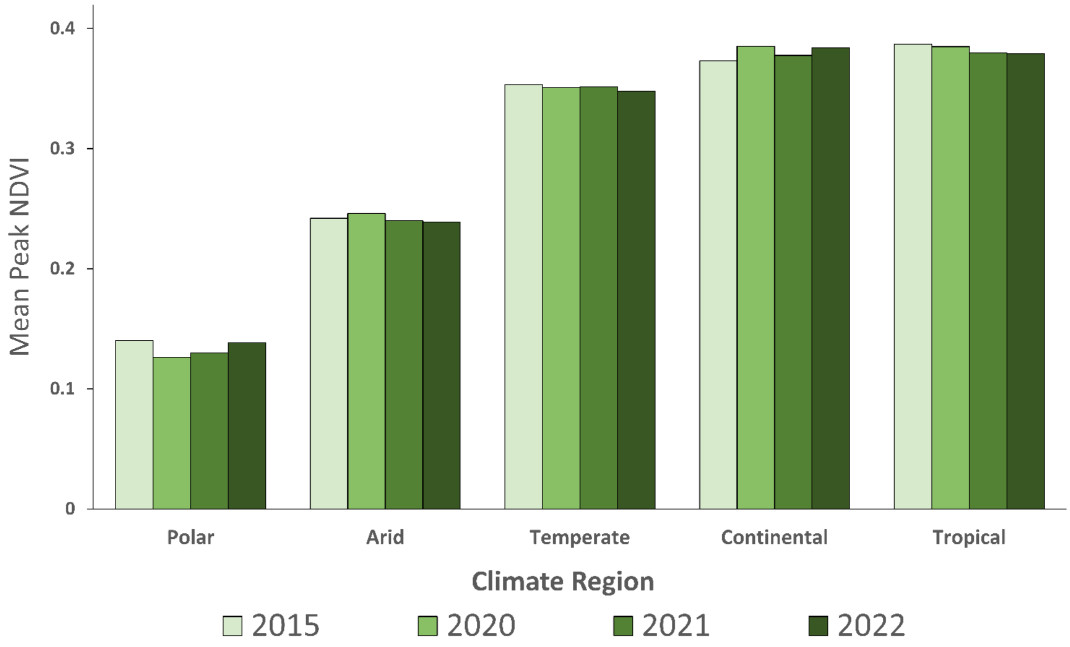

Figure 81 Mean, population-weighted, peak-season NDVI by climate region and year

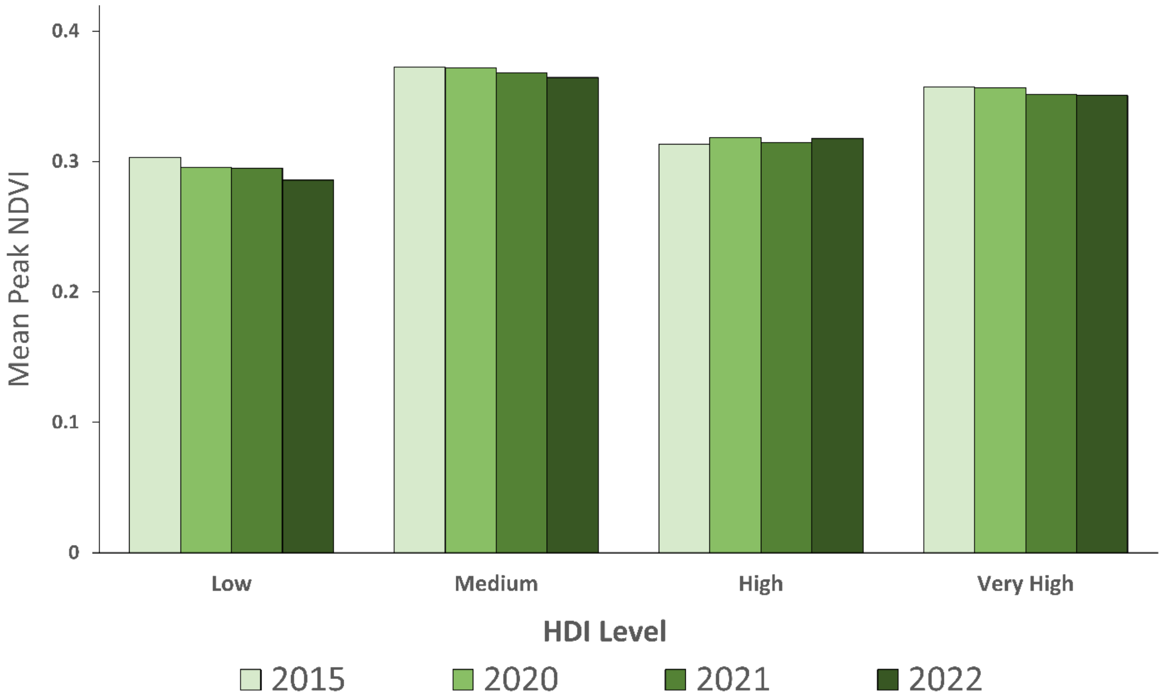

Figure 82 Mean, population-weighted, peak-season NDVI by HDI and year

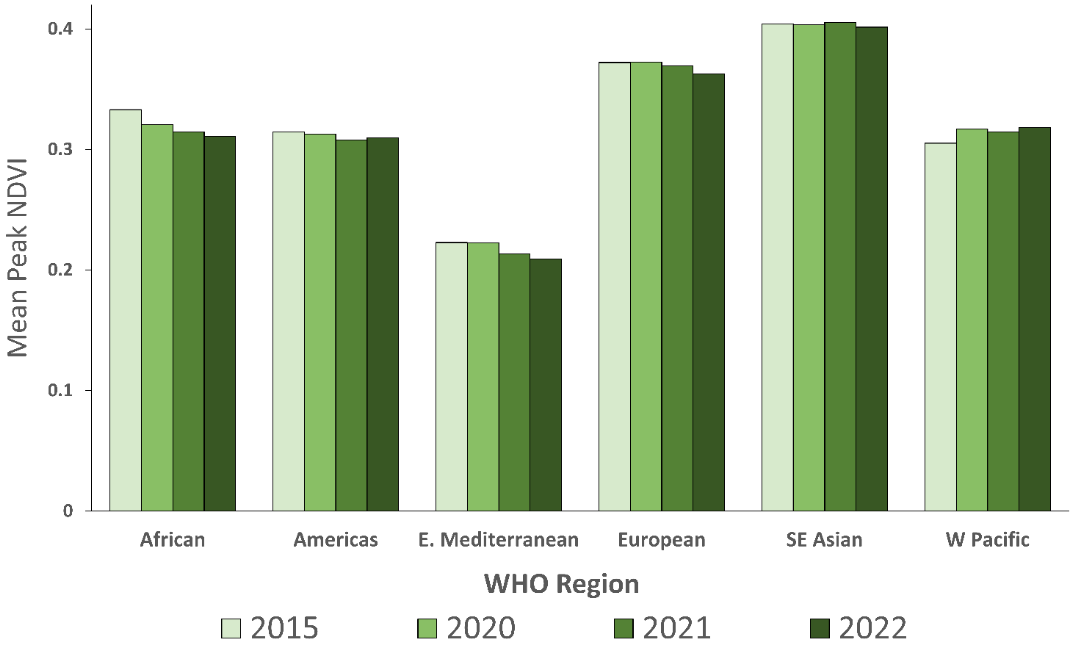

Figure 83 Mean, population-weighted, peak-season NDVI by WHO region and year.

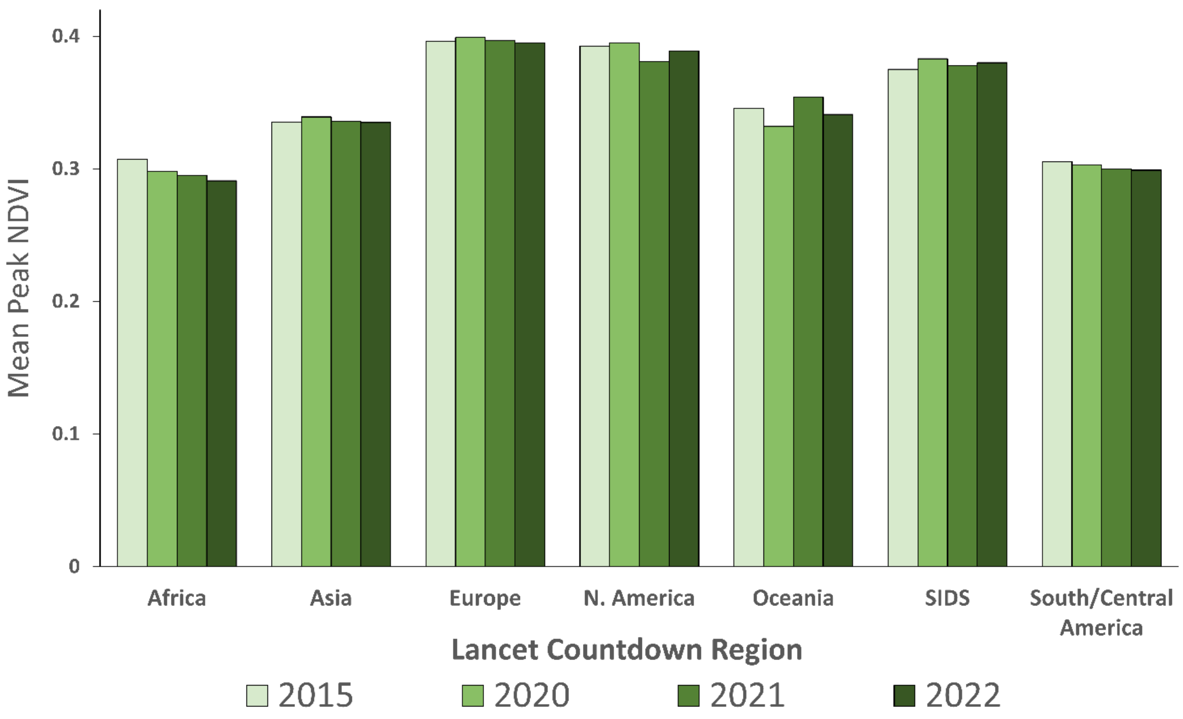

Figure 84 Mean, population-weighted, peak-season NDVI by Lancet Countdown Region designation and year.

Indicator 2.2.4: Global Multilateral Funding for Health Adaptation Programs

Indicator authors

Carole Green

Methods

1. Data Collection:
   a. Data Collection on Funding Approved for Adaptation and Cross-Cutting Projects:

Data were collected from PDF files of Project Approval Documents, accessed via the GCF Project Portfolio, and collated into a spreadsheet.

The GCF Project Portfolio is accessible online from the GCF Website following the Prompts: https://www.greenclimate.fund/ > Projects & Programmes > Lists of Projects. The filter functionality was used to filter projects by ‘Theme’ [Adaptation] or [Cross-Cutting] and ‘Date’ [2022].

PDF files of the Project Approval Documents were downloaded from each of the relevant filtered projects, reviewed individually, and key data points were transferred into a spreadsheet, including:

- Project Reference Number
- Project Name
- Project Region
- Total GCF Financing
- GCF Financing Instrument (Concessional Loan, Grant, Equity, Guarantee)
- Percentage of Financing for Adaptation Elements of the Project, outlined in section A.4 Result Area(s)
- Percentage of Finance for ‘Health, Food, and Water Security’ Elements of the Project, outlined in section A.4 Result Area(s)
- Project objective described in Section B.3. Project/Programme Description of the Project Approval Document

- Presence of GCF Outcome A2.0: Increased resilience of health and wellbeing within the Logical Framework of the Project Approval Document

b. Other Data Collected for Additional Analysis

In addition to the two sub-indicators above, data were collected on projects which received approval for Project Preparation Funding in 2022, and projects which received approval for Readiness Support in 2022. The methodology for collection of these additional data corresponds to the methodology for the collection of data on Concept Notes, with the slight change that the project ‘Type’ filter was adjusted for the respective types of document.

2. Financing for adaptation elements for each of these projects was calculated as:

   Financing for Adaptation Elements per Project = “Total GCF Financing Approved” * “Percentage of Financing for Adaptation Elements” in section A.4 Result Area(s)

Calculating GCF Funding Approved for Adaptation Projects with Health Co-Benefits

- Had funding approved in 2022 AND
- Had >0% financing approved for Adaptation Elements in section A.4 Result Area(s) of the Approved Funding Proposal, consequently either representing ‘Adaptation’ projects of ‘Cross-Cutting’ Projects AND
- Had elaborated a metric for GCF Outcome A2.0: Increased resilience of health and wellbeing within the Logical Framework of the Project Approval Document AND
- Had indicated GCF Contribution towards Increased Resilience of Health and well-being, and food and water security in section A.4 Result Area(s) AND

Funding directed towards Health Co-Benefits for adaptation elements of these projects was calculated as:

> Financing for Health Co-Benefits of Adaptation Elements per Project = “Total GCF Financing Approved” * “Percentage of Financing for Adaptation Elements” in section A.4 Result Area(s) * “Percentage of Financing for Increased Resilience of Health and well-being, and food and water security in section A.4 Result Area(s)”

Funding directed towards health system adaptation elements of these projects was calculated as:

> Financing for Health System Adaptation per Project = “Total GCF Financing Approved” * “Percentage of Financing for Adaptation Elements” in section A.4 Result Area(s) * “Percentage of Financing for Increased Resilience of Health and well-being, and food and water security in section A.4 Result Area(s)”

Total GCF Funding Approved for Adaptation Projects with Health Co-Benefits represented the sum of Financing for Health System Adaptation per Project.

Data

This indicator used data from the Green Climate Fund (GCF https://www.greenclimate.fund/) and the Climate Funds Update (CFU http://www.climatefundsupdate.org) data dashboard.

Caveats

This indicator provides in-depth analysis of funding approved by the GCF in 2022 for Health Adaptation projects. While this likely represents a good indicator of Climate Change funding trends for multilateral financing, it is possible that other Funds show a different trend. Moreover, this indicator is limited through reliance on manual data-transfer from the GCF website into a spreadsheet.

Future Form of the Indicator

In future years, the ambition is to work with Climate Change funds, starting with the Green Climate Fund, to consistently collect data on funding approved for Health Adaption which will improve the data quality of this indicator.

Indicator 2.2.5: Detection, Preparedness, and Response to Health Emergencies

Indicator authors

Diarmid Campbell-Lendrum, Tara Neville Methods

This indicator takes data from the International Health Regulations (IHR (2005)) State Party Self-Assessment Annual Reporting Tool (SPAR). Data reported here were updated on April 28, 2023. Under the IHR (2005) all States Parties are required to have or to develop minimum core public health capacities to implement the IHR (2005) effectively. IHR (2005) also states that all States Parties should report to the World Health Assembly annually on the implementation of IHR (2005). In order to facilitate this process, WHO developed an IHR Monitoring questionnaire, interpreting the Core Capacity Requirements in Annex 1 of IHR (2005) into 20 indicators for 13 capacities. Since 2010, this self-reporting IHR monitoring questionnaire is sent annually to National IHR Focal Points (NFPs) for data collection. It contains a checklist of 20 indicators specifically developed for monitoring the development and implementation of 13 IHR capacities. The method of estimation calculates the proportion/percentage of attributes (a set of specific elements or functions which reflect the level of performance or achievement of a specific indicator) reported to be in place in a country.

The core capacities to implement the IHR (2005) have been established by a technical group of experts, as those capacities required to detect, assess, notify, and report events, and to respond to public health risks and emergencies of national and international concern. To assess the development and strengthening of core capacities, a set of components are measured for each of the core capacities, by considering a set of one to three indicators that measure the status and progress in developing and strengthening the IHR core capacities. Each indicator is assessed by using a group of specific elements referred to as ‘attributes’ that represents a complex set of activities or elements required to carry out this component. The annual questionnaire has been conducted since 2010 with a response rate of 72% in 2012, 66% in 2016 and 85% in 2017, and 100% of countries reporting at least once since 2010. Annual reporting results are complemented by after action reviews, exercises, and joint external evaluation (JEE).

At the beginning of 2018, in compliance with the recommendations of the IHR Review Committee on Second Extensions for Establishing National Public Health Capacities and on IHR Implementation and following formal global consultations with States Parties held in 2015, 2016, and 2017, and 2018, the WHO Secretariat replaced the IHR Monitoring questionnaire by the “IHR State Party Self-assessment Annual Reporting (SPAR) Tool”.

Between 2018 and 2020, the SPAR tool provided results for IHR core capacity 8 (C8), National Health Emergency Framework. This core capacity had 3 indicators C8.1 Planning for emergency preparedness and response mechanism, C8.2 Management of health emergency response operations, and C8.3 Emergency resource mobilization. However, adjustments were made in the SPAR tool in 2021. In 2021, core capacity 7 (C7) Health Emergency Management, reported on three indictors: C.7.1 planning for health emergencies, C.7.2 management of health emergency response, and C.7.3 emergency logistic and supply chain management. The indicator C8.3 no longer exists in this eSPAR version 2. This makes C.7 Health emergency management in version 2 incomparable with C8 version 1. Therefore, findings in the 2023 Lancet Countdown Report cannot be compared to findings in the 2021 or earlier reports.

The SPAR tool scoring system, has remained the same since 2018. It is summarized in the table below.

Table 59: SPAR tool scoring system of IHR core capacities

Scoring System

Indicator Level	Score	Score Range	Color
1	20	0-20	
2	40	21-49	
3	60	41-60	
4	80	61-80	
5	100	81-100	

Table 61 details the Lancet Countdown classification of level of implementation of core capacity 7 of the IHR SPAR tool. This follows the same classification as in the 2022 report.

Scoring System				
Indicator Level	Score	Score Range	Color	2022 Lancet Countdown classification of level of implementation
1	20	0-20		Very low
2	40	21-49		Low
3	60	41-60		Medium
4	80	61-80		High
5	100	81-100		Very high

Table 60 Lancet Countdown classification of level of implementation of core capacity 7 of the IHR SPAR tool.

A total of 185 State Parties had reported to the Sepf-Assessment Annual Reporting Tool as of April 28, 2023. State Parties that did not have an HDI classification were removed from the analysis (5 countries). This left a total of 180 countries included in the findings.

The Strategic Partnership for Health Security and Emergency Preparedness Portal tracks progress and gaps in IHR implementation through independent external evaluations, simulations, after-review exercises, and the development of national action plans for health security and resource mapping.

Data

1. International Health Regulations (2005) Annual Reporting. Data is available through the Global Health Observatory Data Repository for 2010–2017, and through the electronic IHR State Parties Self-Assessment Annual reporting Tool (e-SPAR) for 2022.[173] https://extranet.who.int/e-spar.
2. World Health Organization. Strategic Partnership for Health Security and Emergency Preparedness (SPH) Portal. World Health Organization. https://extranet.who.int/sph/.[174]

Caveats

There are some limitations to considering these capacities as proxies of health system adaptive capacity and system resilience. Most importantly, IHR monitoring questionnaire responses are self-reported. Secondly, the countries that report IHR implementation differ from year to year within these regional aggregate scores.

Thirdly, IHR Core Capacity Requirements are not specific to climate change, and hence whilst they provide a proxy baseline, they do not directly measure a country's adaptive capacity in relation to climate driven risk changes. Fourthly, these findings capture potential capacity – not action. Finally, the quality of surveillance for early detection and warning is not shown and neither is the impact of that surveillance on public health. Response systems have been inadequate in numerous public health emergencies and thus the presence of such plans is not a proxy for their effectiveness. Nevertheless, these capacities provide a useful starting point to consider the potential adaptive capacity of health systems globally.

Future form of the indicator

The World Health Assembly resolution WHA73.1 requested the WHO Director-General to initiate a process of impartial, independent and comprehensive evaluation of the WHO-coordinated international health response to COVID-19, including the mechanisms in place under the IHR. Future forms of this indicator will need to evolve along with the outputs of this review.

2.3: Vulnerabilities, Health Risk, and Resilience to Climate Change

Indicator 2.3.1: Vulnerability to Mosquito-Borne Diseases

Indicator authors

Prof Jan C. Semenza, Marisol Yglesias González

Methods

This indicator tracks the vulnerability of countries to severe adverse health outcomes from *Aedes*-borne diseases (Dengue, Chikungunya and Zika) considering urban population (UP) as a susceptibility variable and health care access and quality (HCAQ) as a coping capacity variable. The results are aggregated by WHO, UNDG human development index (HDI) and Lancet Countdown grouping. For the analysis, the period of consideration ranges from 1990 to 2021.

Vulnerability is computed by dividing the percentage of UP scaled from 1 to 100, by the percentage of a proxy of HCAQ scaled 1 – 100. HCAQ results from the subtraction of 100 - % of deaths by communicable diseases and maternal, prenatal and nutrition conditions obtained the Global Burden of Disease Study 2019. The results have been scaled to display a vulnerability indicator that ranges from 0 to 100.

$$\boldsymbol{Vulnerability} = \boldsymbol{UP}/\ \boldsymbol{HCAQ}$$

Data

1. Global Burden of Disease Collaborative Network. Global Burden of Disease Study 2019 (GBD 2019) Reference Life Table. Seattle, United States of America: Institute for Health Metrics and Evaluation (IHME), 2021
2. World Bank, World Development Indicators. Urban population (% of total population). Available from: https://data.worldbank.org/indicator/SP.URB.TOTL.IN.ZS

Caveats

HCAQ values have been updated for 2020 and 2021 by extrapolating 2019 data from the Global Burden of Disease Study 2019. The indicator is extrapolated to country level, no estimations at subnational level to differentiate vulnerability between rural and urban settings have been done. Countries that reported a high value of vulnerability (Ivory Coast, Zambia and Zimbabwe) showed an influence lifting the vulnerability average for the other countries, therefore affecting the results for WHO regions and HDI groups too. In that case, these countries were excluded from the analysis to avoid misleading results.

Future form of the indicator

An improved version of this indicator will be developed in the future, incorporating other factors linked to vulnerability to dengue in the literature.

337,338333332,339334332340339341–343327329,330,339342344345346328–
330347348333,339333345347348349350351352353354355354356357358172172,359360354361362363360362,363364365366367368369369370371,372364

Additional analysis

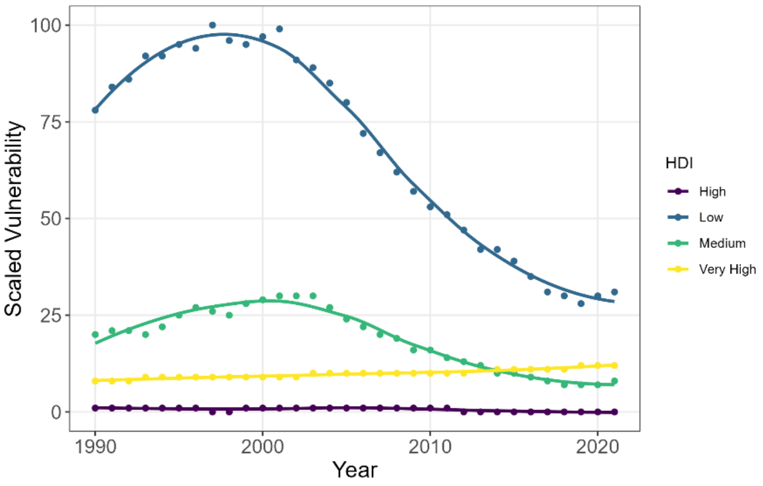

Figure 85: Global scaled vulnerability to mosquito-borne disease, by HDI, from 1990 to 2021.

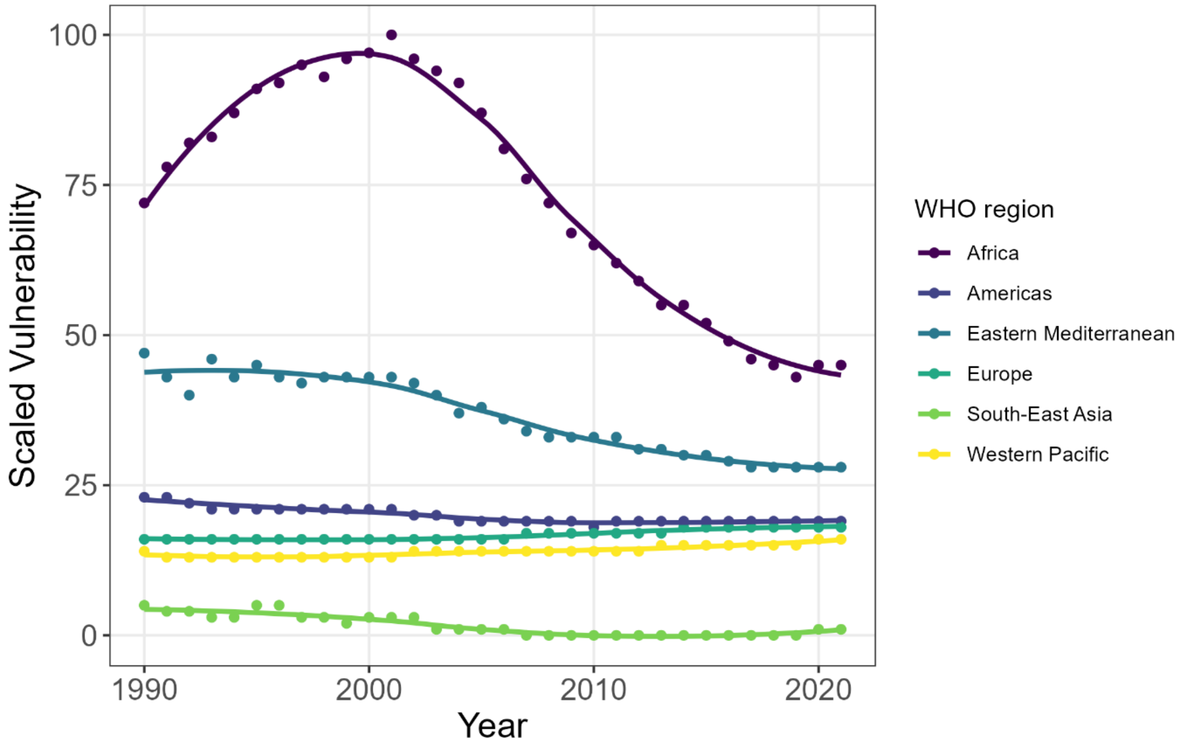

Figure 86: Global scaled vulnerability to mosquito-borne disease, by WHO region, from 1990 to 2021.

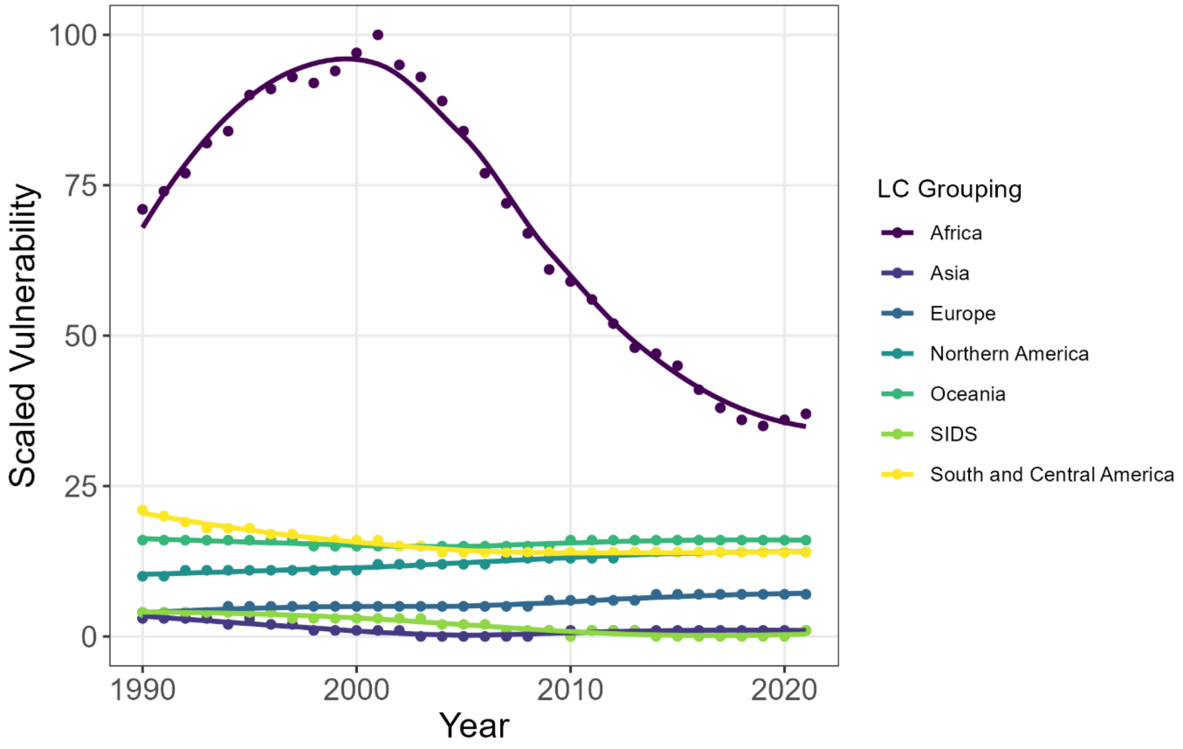

Figure 87: Global scaled vulnerability to mosquito-borne disease, by LC region, from 1990 to 2021.

Indicator 2.3.2: Lethality of Extreme Weather Events

Indicator authors

Prof Dominic Kniveton

Methods

The methodology for this indicator remains similar to that described in the 2020 report of the *Lancet* Countdown.[175] The number of occurrences of weather-related disasters (drought, storms, wildfires, floods and extreme temperatures), the number of people affected in each disaster, and the lethality of these events have however been grouped according to the 2019 HDI level for each country over the period from 1990 to 2020.

The methodology uses data from the Centre for Research on the Epidemiology of Disasters (EM-DAT).[176] Here, deaths, as proxy of the lethality of weather-related disasters, are defined as the number of people who lost their life because the disaster happened. People affected are defined as those requiring immediate assistance during a period of emergency; hence requiring basic survival needs such as food, water, shelter, sanitation, and immediate medical assistance.

Data

1. EM-DAT at the Centre for Research on the Epidemiology of Disasters (CRED) at the Université Catholique de Louvain, Belgium[176]
2. Human Development Index (HDI) at the United Nations Development Programme, Human Development Reports[177]

Caveats

The EM-DAT database contains a number of possible biases. Firstly, there is a possible bias in missing some disaster events because of under-reporting. EM-DAT classifies an event as a disaster if 10 or more people die; 100 or more people are affected; there is a declaration of a state of emergency; or a call for international

assistance. Similarly, there are likely biases in how countries report both the number of deaths and people affected. Numbers of deaths for example may not include mortality from the cascading risks of natural hazards or those that occur as a result of longer causal chains from the hazard. Secondly, estimates of the numbers of people affected have different biases for different countries because of how the concept of “affected people” is defined. This must be considered when comparing countries.

Additional Analysis

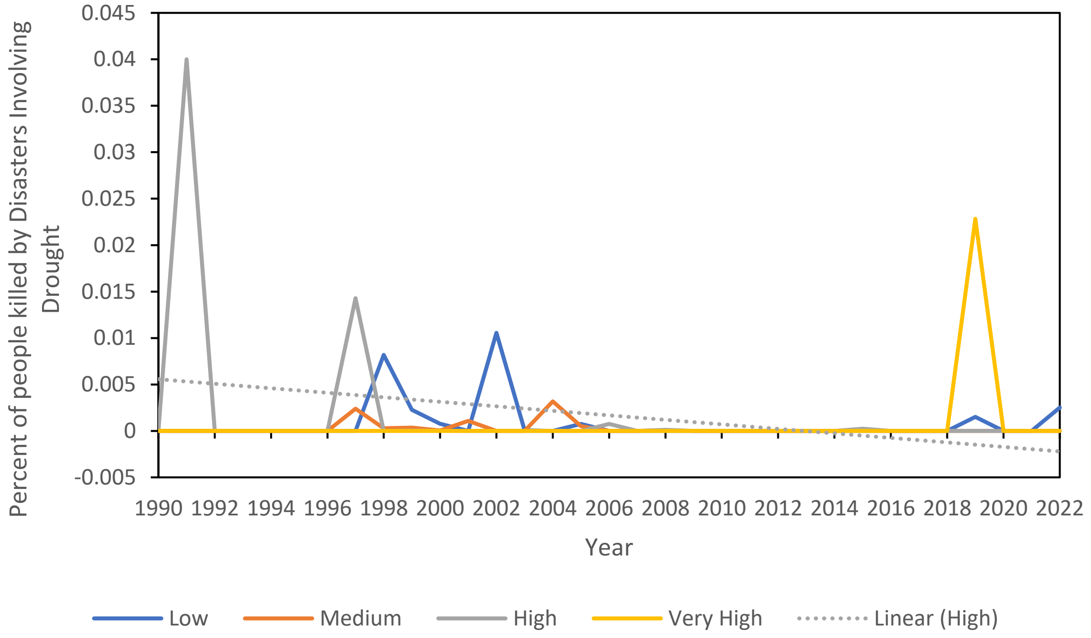

Figure 88 Number of People Killed by Disasters Involving a Drought as a Percentage of Those Affected

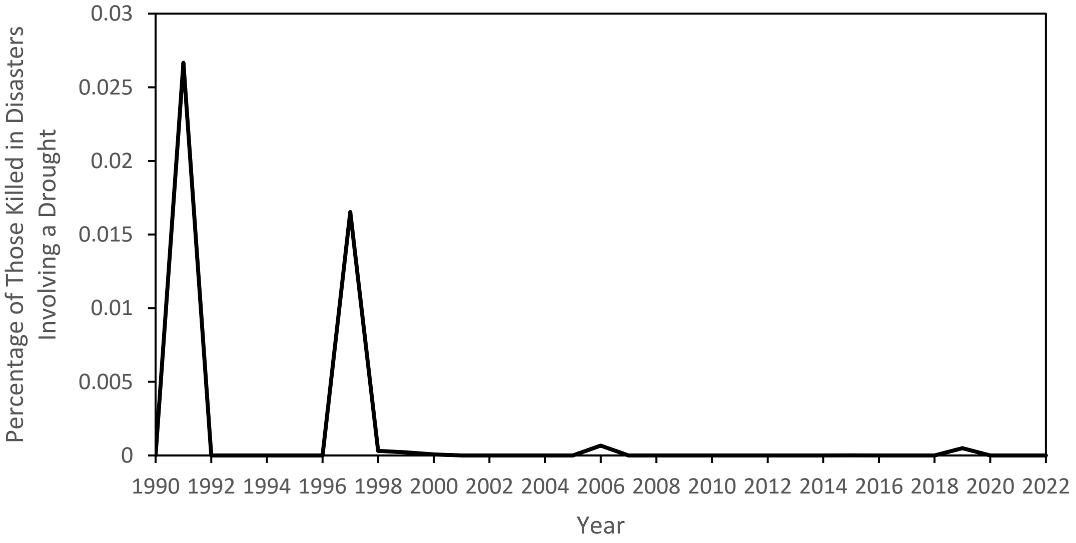

Figure 89 Number of People Killed by Disasters Involving a Drought in Asia as a Percentage of Those Affected

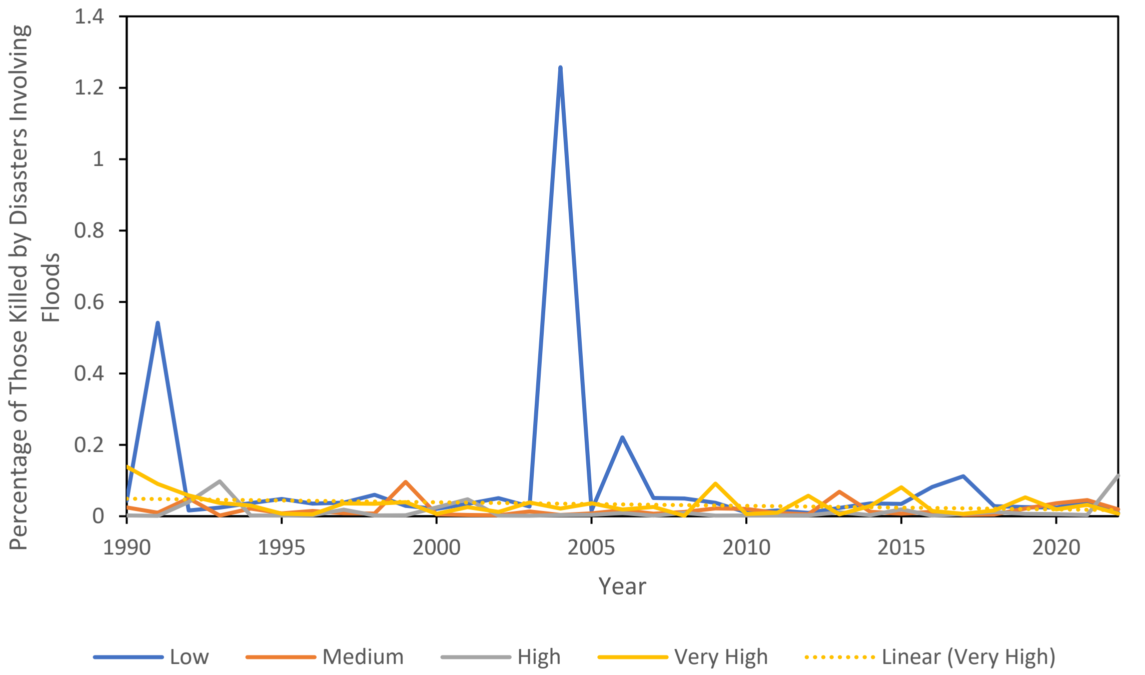

Figure 90 Number of People Killed by Disasters Involving Floods as a Percentage of Those Affected

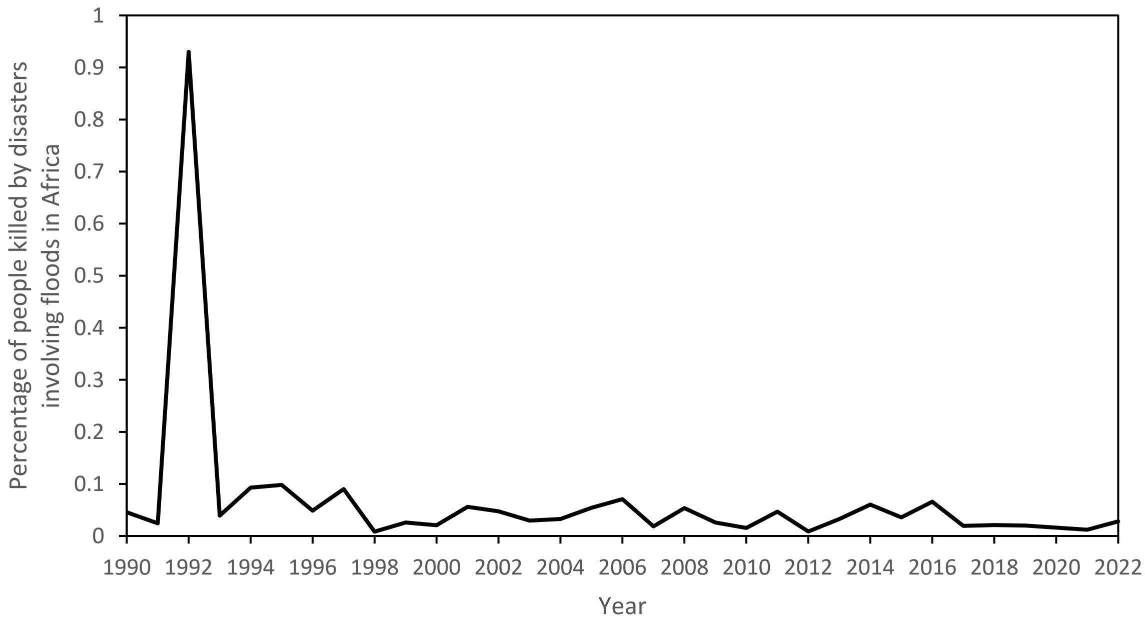

Figure 91 Number of People Killed by Disasters Involving Floods In Africa as a Percentage of Those Affected

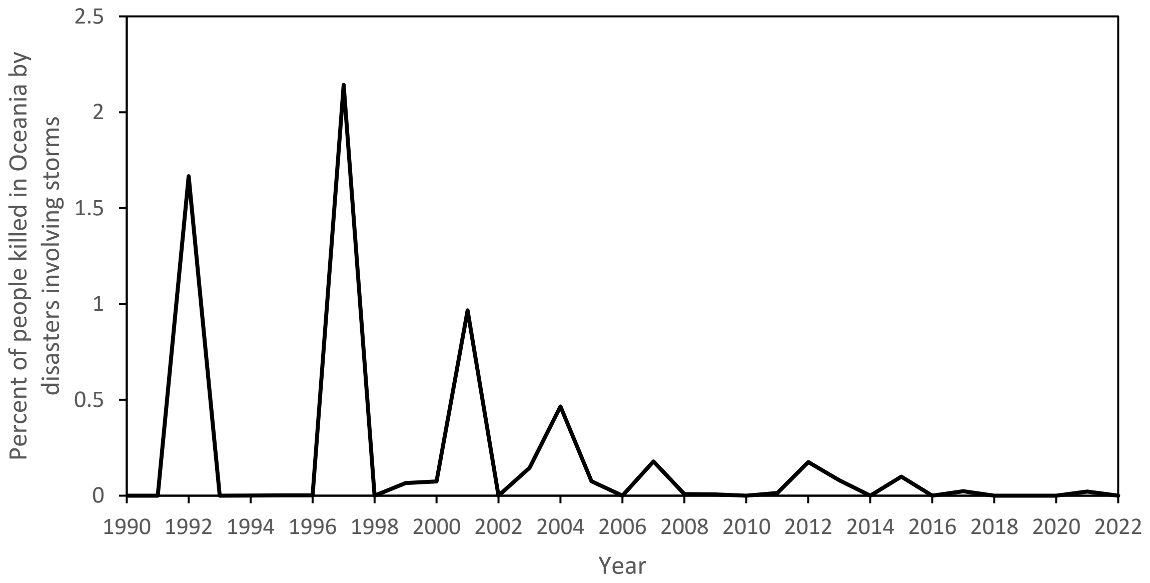

Figure 92 Number of People Killed by Disasters Involving Storms in Oceania as a Percentage of Those Affected

Indicator 2.3.3: Migration, Displacement and Rising Sea Levels

Population exposure to global mean sea level rise

Indicator authors

Dr Sonja Ayeb-Karlsson, Dr Shouro Dasgupta, Prof Ilan Kelman, Prof Celia McMichael

Methods

The methodology for this indicator remains similar to that described in the 2022 report of the *Lancet* Countdown.[41] By using a bathtub model, this indicator overlays future Global Mean Sea Level Rise (GMSLR) of 1 m with coastal elevation value grid-cells to delineate areas of potential inundation and current global population distribution grid-cells to delineate populations living in areas exposed to absolute GMSLR of 1 m.

In the first step, the Coastal Digital Elevation Model (CoastalDEM) dataset was used to categorise inundated grid-cells under 1m of GMSLR.[178] In the second step a gridded population dataset [179] was overlaid to estimate population exposure values. These grid-cells were then matched with country boundaries using the Global Administrative Areas (GADM) Dataset (version 4.0.4). Then the grid-cell level data were aggregated to country level (i.e. national population numbers exposed to 1m of GMSLR).

Data

1. GMSLR: Estimated global mean increases in sea-levels[180]
2. Elevation: Coastal Digital Elevation Model (CoastalDEM)[178]
3. Hybrid gridded demographic data for the world [179]
4. Global Administrative Areas (GADM) version 4.0.4, http://www.gadm.org/

Caveats

The global mean sea level rose 4.68 mm per year between 2013-2022[181] and is projected to reach 0·29–1·10 m by 2100 (relative to 1986–2005), depending on emission scenarios and environmental responses.[182–184] Due to uncertainty in the Greenland and Antarctic ice sheet melt processes, GMSLR of 2 m by 2100, 5 m by 2150 and 15 m by 2300 under very high greenhouse gas emission scenarios cannot be ruled out.[185,186]

Estimates of population exposure to GMSLR vary according to datasets, timeframes, emissions and socioeconomic scenarios, and analytical method.[187] For this indicator, the datasets used, and analytical approach determine results. CoastalDEM (3-arc second; 90m) is a global coastal digital elevation model that is adjusted to reduce SRTM error.[182] While SLR-related hazards could potentially displace people living in sites of coastal risk, population exposure to SLR is not a proxy indicator for SLR-related population displacement. Displacement can be prevented or forestalled through protection (e.g., armouring coasts) and accommodation (e.g. measures that facilitate living with SLR impacts); some may be unable or unwilling to leave; and people migrate into low-lying coastal sites.[188] When protection and accommodation are exhausted or not feasible, retreat from sites of SLR-related risk may occur.

SLR and associated flooding and erosion can contribute to health risks including saltwater intrusion of drinking water, food insecurity (e.g., loss or arable land, reduced crop yields), altered infectious disease ecology, and mental health impacts.[189–191] Empirical studies identify diverse consequences of relocation and retreat from sites of coastal risk, including for mental health, food security, water supply, sanitation, infectious diseases, injury, and health care access.[192,193]

Future form of the indicator

Plans to improve the methodology, data sources, and/or temporal and geographical coverage of this indicator in subsequent reports. As new, higher spatial resolution and more precise datasets become available, we will update our methods to produce robust estimates of population exposure to future GMSLR.

Additional analysis

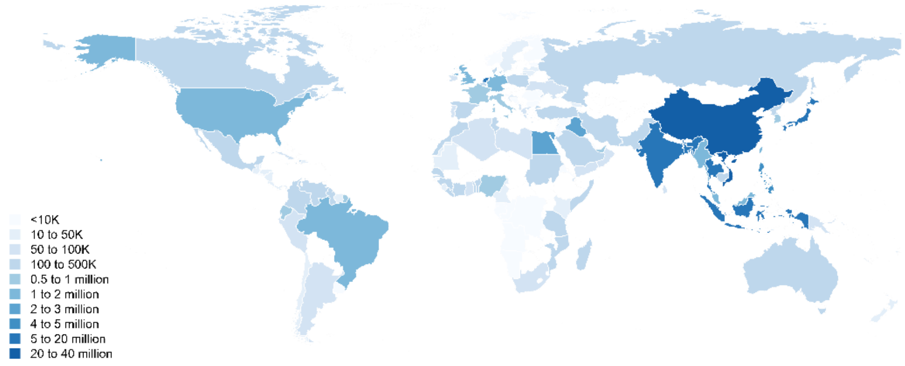

Figure 93 Population exposure to 1m GMSLR

National Policies on Migration

Methods

This component of this indicator on national policies reports:

1a. The number of currently valid national-level policies including legislation for migrants, migration, displacement, displaced people, relocation, and relocated people specifically related to climate change (not climate or disasters), including immobility (trapped populations/non-migration/non-displacement).

1b. The number of such policies mentioning health or well-being along with a qualitative analysis of how health and/or well-being are/is mentioned.

2a. The number of countries with at least one such policy.

2b. The number of such countries whose policies mention health or well-being along with a qualitative discussion of how health or well-being is mentioned.

"Country" refers sovereign state or autonomous non-sovereign territory (not just a sub-national jurisdiction). Multi-lateral, inter-governmental, and international policies are specifically excluded. Explicit mentions of "climate change" and "health" or "well-being" must be present, not implied definitions or references to wider

contexts which might (or might not) encompass these points, e.g., “climate”, “climate disasters”, “humanitarian”, and “environment”.

The method for identifying national-level policies is:

1. A systematic review, using the keywords which define the indicator
2. Crowd-sourcing and expert queries[194]

Because this search can never know what might have been missed, the numbers reported for this indicator represent minimum counts. Each policy included is also categorised by:

1. (a) Migration/mobility/displacement/relocation from a location,
   (b) migration/mobility/displacement/relocation to a location, and
   (c) immobility/trapped populations.
2. (a) Domestic migration/mobility/displacement/relocation and
   (b) international migration/mobility/displacement/relocation — all immobility, by definition, is domestic

A given policy might be counted in more than one category for 1abc and for 2ab. Some policies do not have an end date, and some do, with both included. Policies which are now out-of-date are retained in a separate list as well as a list of policies considered but not included in this indicator.

Caveats

As documented in previous *Lancet* Countdown reports[41,175,195–197] and supporting publications,[198] the main caveats with using migration or displacement as a climate change and health indicator are:

1. Attributing movement or immobility to climate change or climate change impacts is not straightforward.

2. Attributing health outcomes to movement or immobility is not straightforward.

These two attribution relationships are debated whether or not (i) there are or will be links between climate change and migration, displacement, (im)mobility, relocation and (ii) there are or will be links between migration, displacement, (im)mobility, relocation and health/well-being.
However, there is a growing body of research that indicates climate change and extreme weather conditions, events and variability contribute to human migration and displacement, albeit strongly influenced by economic, social, political and demographic processes.[199]

This indicator assists in overcoming the attribution problem by:

1. Examining written policies, so attribution is not a concern, because the policies exist, even if attribution is inappropriate.
2. Examining how policies mention health/well-being, so again actual attribution is not a concern, because the text on health or well-being either exists or does not exist, even if attribution is inappropriate.

If spurious attributions are made in the policies between (i) climate change and migration/displacement/immobility or (ii) migration/displacement/immobility and health or well-being, then this indicator can analyse those attributions and why they might not be defensible, based on the scientific literature. Thus, this indicator provides what is happening at the national level and the appropriateness of these policies in terms of the scientific literature. The key to this approach and to overcoming the caveats is keeping the indicator simple and straightforward, which is why the indicator has been designed in the proposed manner.

Selecting policies, and in particular national policies, does not cover all possibilities, but it serves as an indicator. As well, it is an indicator of how national governments perceive the climate change / (im)mobility / health links, without making a statement on the actual links, which the literature explains is exceptionally difficult. This approach to the indicator also means that misattributions are easily filtered out, such as reporting migration and health links to disasters or climate, both of which are different from links to climate change. Using ‘climate change’ synonymously with ‘climate’, ‘climate-related disasters’, and/or ‘disasters’, is a common mistake in many policies reviewed as well as in the academic literature.

The main caveat is that most of the data is confined to documents in English, with a few other languages on occasion. The advantage is that policies which are not available in English have typically been discussed in English publications, including blogs and news reports, suggesting that much relevant material has been captured.

Nonetheless, the numbers reported can only be taken as the minimum, as in 'at least so many' policies match the criteria stated. One minor caveat is that the number of countries sometimes changes year-to-year, providing a different baseline. These changes are rarely more than one or two countries per year out of a sample of around 200. Substantial changes to the numbers of countries will be reported if this occurs.

Future Form of the Indicator

Plans to improve the methodology, data sources, and/or temporal and geographical coverage of this indicator in subsequent reports.

The indicator design helps in overcoming these caveats by reporting that the counts provided must be only minimum numbers, because we cannot know what we would have missed. Through publicity, publication, crowd sourcing, and expert connections, this limitation will be overcome because people will provide examples of what we missed. As an indicator, it is important to accept that the numbers are not comprehensive but provide only minimum numbers as a lower-bound baseline.

Section 3: Mitigation Actions and Health Co-Benefits

Lead Author: Prof Ian Hamilton

Research Fellow: Dr Harry Kennard

Indicator 3.1: Energy Use, Energy Generation, and Health

3.1.1: Energy Systems and Health

Indicator authors

Dr Harry Kennard, Dr Matthew Winning

Carbon Intensity of the Energy System

Methods

This indicator contains two components:

- Carbon intensity of the energy system, both at global and regional scales, (1971–2020), in tCO_2/TJ
- Global CO_2 emissions from energy combustion by fuel, in $GtCO_2$ (1972–2020). Global emissions without fuel breakdown are also provided for 2021 and provisionally for 2022.

The technical definition is the tonnes of CO_2 emitted for each unit (TJ) of primary energy supplied.

The rationale for the indicator choice is that carbon intensity of the energy system will provide information on the level of fossil fuel use, which has associated air pollution impacts. Higher intensity values indicate a more fossil dominated system, and one that is likely to have a higher coal share. As countries pursue climate mitigation goals, the carbon intensity is likely to reduce with benefits for air pollution.

The indicator is calculated based on total CO_2 emissions from fossil fuel combustion divided by Total Energy Supply (TES). TES reflects the total amount of primary energy used in a specific country, accounting for the flow of energy imports and exports.

The data is available for most countries of the world, for the period 1971–2020.

Data

1. This indicator is based on based on the IEA dataset, CO_2 Emissions From Fuel Combustion: CO_2 Indicators, accessed via the UK data service,[200] and supplemented with additional data for 2022[201]

Caveats

IEA data are generated using both direct input from national governments and modelling. As such, while they represent the best available data on national CO_2 emissions from fuel, they are subject to caveats which vary by energy commodity and country. Full details are given in the CO_2 Emissions From Fuel Combustion documentation.[202]

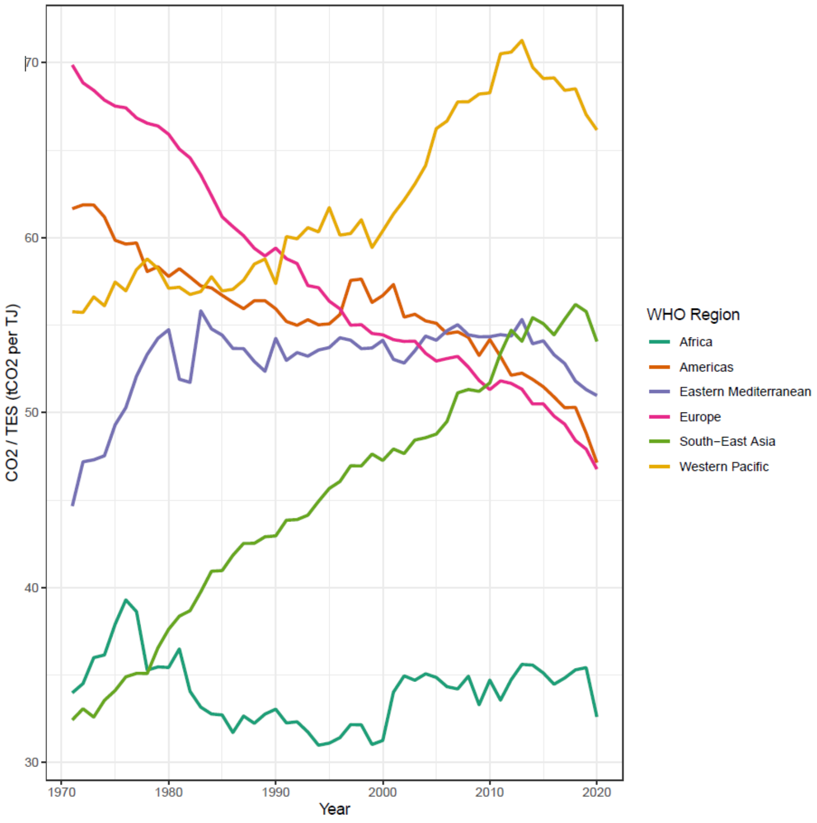

Figure 94 Carbon intensity of the energy system by WHO region, 1971–2020 (tCO_2/TJ)

Coal Phase-Out

Methods

Two indicators are used here:

1. Total primary coal supply by region / country (in exajoules, EJ);
2. Share of electricity generation from coal (% of total generation from coal) and global generation from coal (in TWh)

These indicators are important to enable tracking of changes in coal consumption at a regional and country level. Due to the level of coal used for power generation, a second indicator tracks the contribution to electricity generation from coal power plants in selected countries. As countries pursue climate mitigation goals, the use of coal is likely to reduce with resulting benefits for air pollution.

The indicator on primary energy coal supply is an aggregation of all coal types used across all sectors (from the IEA energy balances). The data are available for most countries of the world, for the period 1978–2020.

The indicator on the share of electricity generation from coal is estimated based on electricity generated from coal plant as a percentage of total electricity generated. Regional data are available from 1990–2020; pre-1990 data are not used due to incomplete time series.

Countries or regions with large levels of coal use (as a share of generation, or in absolute terms), have been selected to show in the figures.

The following types of coal are added to produce the total primary coal supply:

‘Anthracite’, ‘Coking coal’, ‘Lignite’, ‘Other bituminous coal’, ‘sub-bituminous coal’

Data

1. This indicator is based on the extended energy balances from the International Energy Agency. The specific dataset is called World Extended Energy Balances (for 2022), and is sourced via the UK data service[203]

Caveats

IEA data are generated using both direct input from national governments and modelling. As such, they are subject to caveats which vary by energy commodity and country. Full details are given in the IEA World Energy Balances documentation.[204] This documentation also covers changes to methodology in previous editions of IEA World Energy Balances. A typical example of the way data can be impacted by methodology updates by reporting countries is as follows, relating to Belgium ‘New data on consumption cause breaks in time series for primary solid biofuels between 2011 and 2012’. However, since data are aggregated, the impacts on overall trends is minimal.

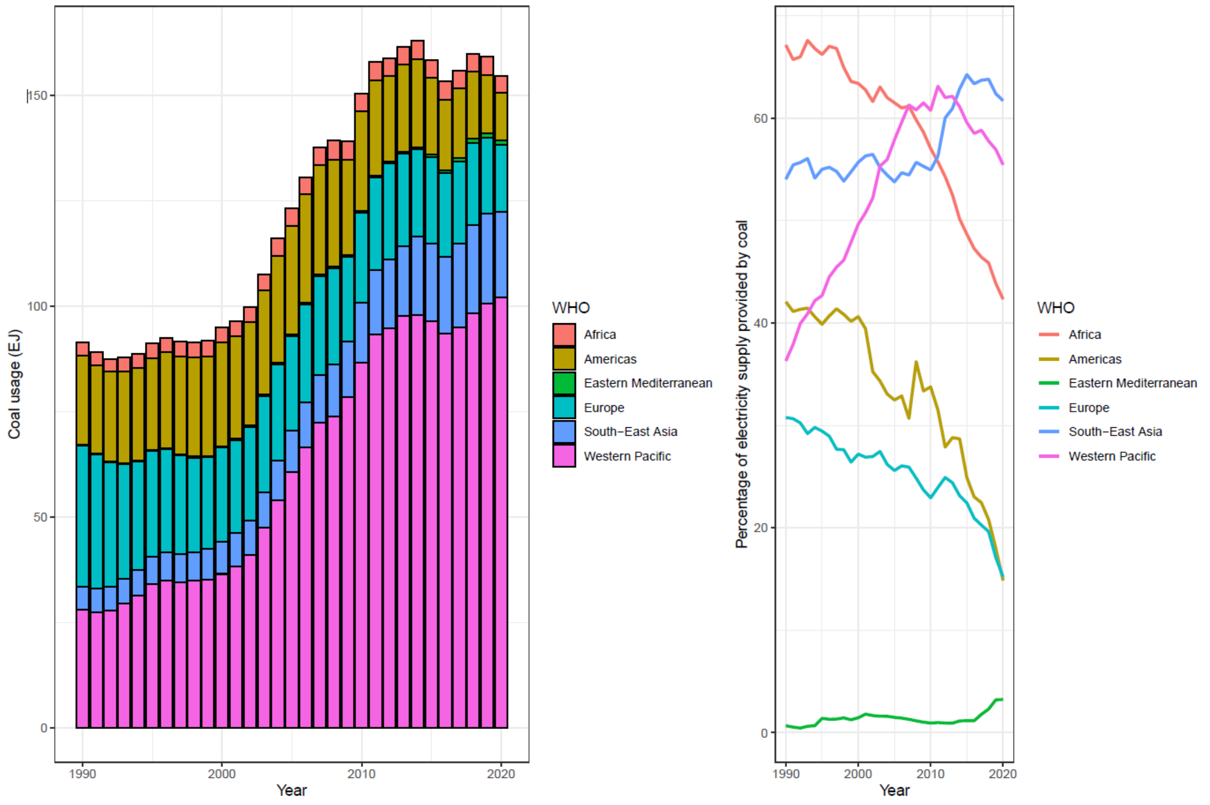

Figure 95 Left: Coal use by WHO region, 1990–2020 (EJ) and right: share of electricity supply provided by coal by WHO region, 1990–2020 (%)

Zero-Carbon Emission Electricity

Methods

Two indicators are used here, and presented in two ways:

1. Total low carbon electricity generation, in absolute terms (TWh) and as a % share of total electricity generated (to include nuclear, and all renewables); and

2. Total modern renewable generation (wind and solar), in TWh, and as a % share of total electricity generated

The increase in the use of low carbon and renewable energy for electricity generation will push other fossil fuels, such as coal, out of the mix over time, resulting in an improvement in air quality, with benefits to health.

The renewables (wind and solar) indicator has been used to allow for the tracking of rapidly emergent renewable technologies. For both indicators, generation, rather than capacity, has been chosen as a metric as the electricity generated from these technologies is what actually displaces fossil-based generation. Countries with large levels of low carbon generation (as shares, or in absolute terms), or with higher fossil dependency, have been selected.

The data are taken from the IEA extended energy balances.[203] The absolute level indicators are total gross electricity generated aggregated from the relevant technology types. The share indicators are estimated as the low carbon or renewable generation as a % of total generation.

The data are available for most countries of the world, for the period 1971–2020. Only the period from 1990 has been used, due to data gaps for selected countries prior to 1990.

The following IEA variable names are added to produce total low carbon electricity generation:

'Nuclear, 'Hydro', 'Geothermal', 'Solar photovoltaics', 'Solar thermal', 'Tide, wave and ocean', 'Wind'

The following IEA variable names are added to produce total modern renewable electricity generation:

'Geothermal', 'Solar photovoltaics', 'Solar thermal', 'Tide, wave and ocean', 'Wind'

Data

1. This indicator is based on the extended energy balances from the International Energy Agency. The specific dataset is called World Extended Energy Balances, and is sourced via the UK data service (http://stats.ukdataservice.ac.uk/)[203]

Caveats

IEA data are generated using both direct input from national governments and modelling. As such, they are subject to caveats which vary by energy commodity and country. Full details are given in the IEA World Energy Balances documentation.[204] This documentation also covers changes to methodology in previous editions of IEA World Energy Balances. A typical example of the way data can be impacted by methodology updates by reporting countries is as follows, relating to Belgium 'New data on consumption cause breaks in time series for primary solid biofuels between 2011 and 2012'. However, since data are aggregated, the impacts on overall trends is minimal.

Additional analysis

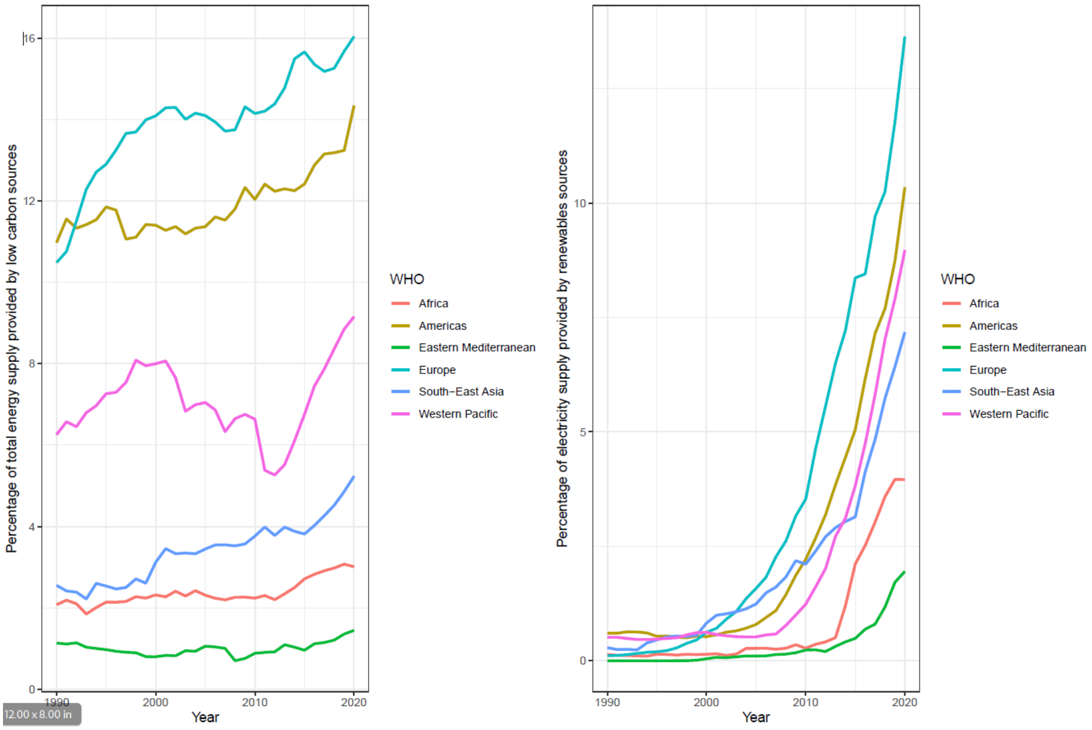

Figure 96. Left: Share of total energy supply provided by low carbon energy sources by WHO region, 1990–2020. Right: Share of electricity generation provided by modern renewables (wind, solar and geothermal) by WHO region, 1990–2020

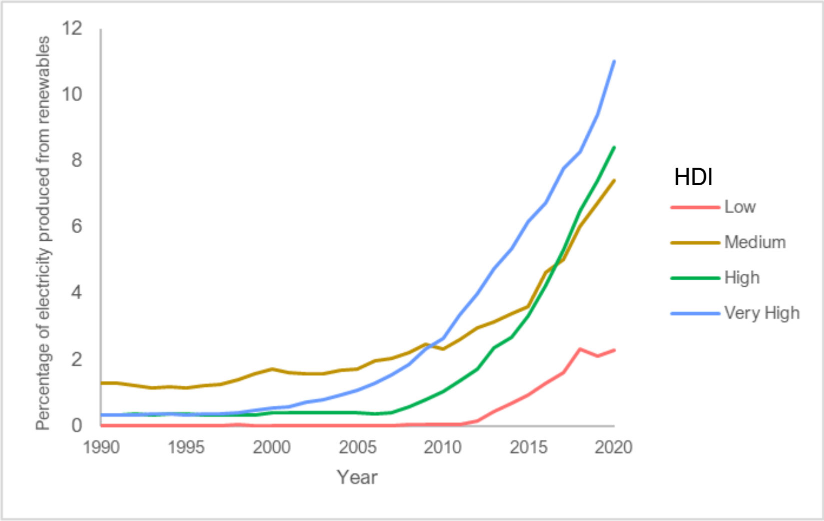

Figure 97: Share of electricity generation provided by renewables (wind, solar and geothermal) by HDI grouping, 1990–2020

3.1.2: Household Energy Use

Indicator authors

Prof Ian Hamilton, Prof Stella Hartinger, Dr Harry Kennard

Access to clean energy is defined by the IEA (2020) as:

"a household having reliable and affordable access to both clean cooking facilities and to electricity, which is enough to supply a basic bundle of energy services initially, and then an increasing level of electricity over time to reach the regional average".[205]

The use of energy in the residential sector is drawn from the IEA extended global residential modelling produced in the World Energy Outlook from the ‘World Extended Energy Balances’ 2022 edition, which covers all countries or major regions in the world.[203] The values are measured in EJ and cover all fuels supplied for consumption within the residential sector (IEA flow code QGFLOW076) final energy demand.

The specific IEA variables were combined in the following way:

`Solid biofuels` = Charcoal + `Primary solid biofuels`

`Coal, coke and peat` = `Hard coal (if no detail)` + BKB + `Petroleum coke` + `Patent fuel`+`Coke oven coke`+`Brown coal (if no detail)`+Peat+`Gas coke`+`Peat products`+`Coking coal`+`Sub-bituminous coal`+`Other bituminous coal`+Lignite+Anthracite+Bitumen

`Other biofuels` = `Other liquid biofuels` + Biogasoline + `Non-specified primary biofuels and waste`+`Biogases`+`Biodiesels`

`Liquid fossil fuels` =`Paraffin waxes`+`Other oil products`+`Naphtha`+ `Gas/diesel oil excl. biofuels` + Lubricants + `Natural gas liquids`+ `Other kerosene`+`Liquefied petroleum gases (LPG)`+`Fuel oil`+`Motor gasoline excl. biofuels`+`Crude oil`

`Waste & other` = `Municipal waste (non-renewable)`+`Municipal waste (renewable)`+`Industrial waste`+`Refinery gas`+ `Blast furnace gas`+`Gas works gas`+`Coke oven gas`+`Oil shale and oil sands`,

Finally, Natural gas, Heat, Solar thermal, Geothermal and Electricity variables were provided directly from IEA flow QGFLOW076.

The visualisation accompanying this indicator shows the principal household energy sources by country. The full breakdown of the fuels used in these regions is given in Table 61.

**Principle household energy sources**	**Heat**	**Liquid fossil fuels**	**Solid biofuels**	**Natural gas**	**Coal, coke and peat**	**Electricity**
Mixed including district heat & coal	27.3%	4.2%	18.9%	12.6%	10.9%	25.1%
Fossil Gas & electricity	4.1%	7.6%	10.5%	52.5%	0.8%	24.5%
Solid biofuels	0.5%	4.2%	80.9%	2.2%	0.2%	11.8%
Liquid fossil fuels & electricity, some biomass	0.4%	38.2%	16.9%	4.9%	1.1%	36.7%
Electricity	0.1%	14.2%	9.2%	4.0%	0.3%	70.2%

Table 61 The mean shares of household energy source by regional type (2019)

Data

1. Healthy fuels for cooking were provided by the WHO[206,207]
2. The additional energy usage and access is based on data from the IEA World Energy Balances 2022[203]

Caveats

The data from the IEA on residential energy flows and energy access provide an indication of both the access to electricity and the proportion of the different types of energy used within the residential sector. These provide an important picture on how access and use might be interacting.

IEA data are generated using both direct input from national governments and modelling. As such, they are subject to caveats which vary by energy commodity and country. Full details are given in the IEA World Energy Balances documentation.[204] This documentation also covers changes to methodology in previous editions of IEA World Energy Balances. A typical example of the way data can be impacted by methodology updates by reporting countries is as follows, relating to Belgium 'New data on consumption cause breaks in time series for primary solid biofuels between 2011 and 2012'. However, since data are aggregated here by HDI level, the impacts on overall trends is minimal.

Future form of the indicator

The WHO are in the process of updating the household energy survey database which underpins this indicator. Future forms of the indicator may be able to be coupled more directly with the negative health outcomes related to the use of dirty fuels in the home.

3.1.3: Sustainable and Healthy Road Transport

Indicator authors

Dr Harry Kennard, Dr Melissa Lott

Methods

Fuel use data (by fuel type) from the IEA World Extended Energy Balances are divided by corresponding population statistics from the United Nations, Department of Economic and Social Affairs, Population Division (Figure 94).

The fuel flows from the IEA are combined in the following way:

Biofuels = Biodiesels +Biogasoline +Biogases + Other liquid biofuels

Fossil fuels = Natural gas liquids+Natural gas+Motor gasoline excl. biofuels+Liquefied petroleum gases (LPG)+Refinery gas+White spirit & SBP+Kerosene type jet fuel excl. biofuels+Gas/diesel oil excl. biofuels+Lubricants+Naphtha+Fuel oil+Other kerosene+Other oil products+Bitumen

Electricity is given by the existing IEA total.

Totals for a given year and country are then divided by the corresponding country population, and then summed to produce the final estimate. This avoids including the population of the countries that are not covered by the IEA.

Data

1. Fuel use data is from the IEA, World Extended Energy Balances[203]
2. UN Population estimates, 2019 edition[208]

Caveats

This indicator captures change in total fuel use and type of fuel use for transport, but it does not capture shifts in modes of transport used. In particular, it does not capture walking and cycling for short trips, which can yield substantial health benefits through increased physical activity.[209]

Alongside the fossil fuel combustion pollutants, tyre wear accounts for an estimated 3–7% of airborne $PM_{2.5}$ particulates worldwide[210]

Future form of the indicator

An ideal fuel use indicator would capture the direct health impacts of the use of transport fuels, with country- and urban-level specificity within the global coverage. In turn, the co-benefits of transitioning to less-polluting fuels would be quantified directly in terms of reduced exposures to air pollution and their corresponding health impact.

To capture sustainable uptake more fully a future indicator could collate information on the proportion of total distance travelled by different modes of transport based on comprehensive local survey data. Other data on sustainable travel infrastructure, for instance the presence of cycle schemes, would also be useful. The data described below in the additional analysis section provided from smartphone data serves to expand the picture provided by IEA data alone. Further development of data of this type may be possible in future reports.

Additional analysis:

Figure 98 shows the global per capita use of fuels for road transport, as well as the percentage energy provided by biofuels and electricity with the countries with the highest adoption of these fuels for road transport.

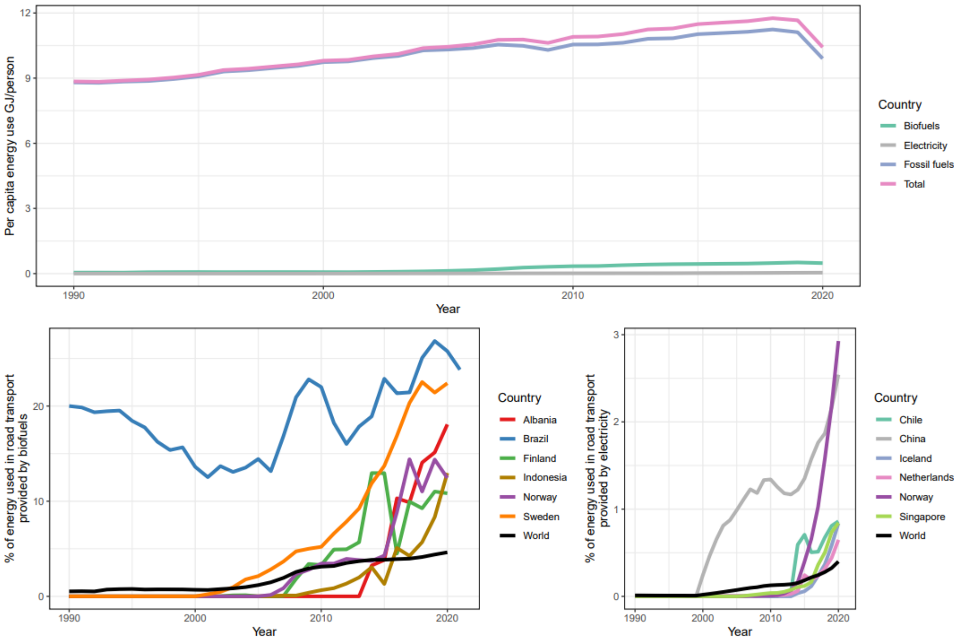

Figure 98 Top panel: Global per capita energy use on road transport by fuel (GJ/Person). Bottom panel: % of road transport energy provided by biofuels (left) and electricity (right) for select countries.

3.2: Air Pollution and Health Co-benefits

Indicator 3.2.1: Mortality from Ambient Air Pollution

Indicator authors

Dr Gregor Kiesewetter, Laura Warnecke

Methods

This indicator quantifies contributions of individual source sectors to ambient $PM_{2.5}$ exposure and its health impacts. Contributions from coal have been highlighted across all sectors.

Estimates of sectoral source contributions to annual mean exposure to ambient $PM_{2.5}$ were calculated using the GAINS model,[211] which combines bottom-up emission calculations with atmospheric chemistry and dispersion coefficients.

Energy statistics are taken from the IEA World Energy Statistics for 2015, from the IEA World Energy Outlook 2021[212] for 2020 and from the World Energy Outlook 2022[213] for 2021. Data on energy consumption in individual sectors are imported into GAINS, matching the sectors of the World Energy Statistics and downscaling to the 180 GAINS global regions. They are then merged with GAINS information on application of emission control technologies in each region and their emission factors to calculate emissions of $PM_{2.5}$ and its precursor gases SO_2, NOx, NH_3, and non-methane VOC.

Ambient $PM_{2.5}$ concentrations are calculated from the region and sector specific emissions by applying atmospheric transfer coefficients, which are a linear approximation of full chemistry-transport models. Atmospheric transfer coefficients in GAINS are based on full year perturbation simulations with the EMEP Chemistry Transport Model[214] at 0.1°×0.1° resolution (for low-level sources) / 0.5°×0.5° resolution (for all other sources) using meteorology of 2015. In Europe, the resolution is slightly different but the principle is the same. Calculations for Europe are described in detail by Kiesewetter et al. (2015),[215] calculations for the rest of the world are described by Amann et al.[216] Calculated ambient $PM_{2.5}$ concentrations have been validated against in-situ observations from the WHO's Urban Ambient Air Pollution Database (2018 update),[217] and other sources where available (e.g., Chinese statistical yearbook) and show in general good agreement with monitoring data up to urban background level (local variation at roadside stations is not captured by the resolution of a few kilometres).

Deaths from total ambient $PM_{2.5}$ for regions other than Europe are calculated following the methodology of the Global Burden of Disease studies. Exposure-response relationships have been updated for this report to be consistent with the Global Burden of Disease 2019 study.[218] The MR-BRT curves were obtained from the public release site[219] and relative risks for six diseases IHD, COPD, stroke, lung cancer, ALRI, and type 2 diabetes calculated from them. The latter has been added this year. We used 1000 draws of the MRBRT curve for each disease and age group (where age specific) and scaled them to have RR=1 at the theoretical minimum-risk exposure level (taken from 1000 corresponding draws, average 4.15μgm^{-3}). Exposure levels below the TMREL level are assigned RR=1.

The update to the GBD-2019 exposure-response relationships resulted in a significant increase in attributable mortality beyond the numbers published in the previous editions of the *Lancet* Countdown, which were based on the integrated exposure response relationships (IERs) developed within the Global Burden of Disease 2013 study.[220]

Disease and age specific baseline mortality rates are taken from the GBD Results database[221] and have been updated to the 2019 data. The shares of different diseases were applied to age-specific total deaths taken from UN World Population Prospects (2017 update);[222] for 2019, the statistics were interpolated linearly between 2015 and 2020.

For Europe, this indicator follows the WHO Europe methodology and applies Exposure-response relationships for all-cause non-accidental mortality among the total population over 30 years of age. This year, concentration-response relationships have been updated to those reported in the systematic review for the 2021 WHO Air Quality Guidelines.[223] Other details are described in Kiesewetter et al. (2015).[215]

Attribution of estimated deaths from AAP to polluting sectors was done proportional to the contributions of individual sectors to population-weighted mean $PM_{2.5}$ in each country.

Data

1. Energy: IEA World Energy Balances for 2015,[224] World Energy Outlook 2021 (for the year 2020),[212] World Energy Outlook 2022 (for the year 2021)[213]
2. Other activities: Agricultural livestock data are based on FAO statistics and projections[225] and fertiliser use is based on data from the International Fertilizer Association
3. UN World Population Prospects, 2017 update[226]
4. Global Burden of Disease 2019 study,[218] MR-BRT curves obtained from the public release site[219]

Caveats

The indicator relies on model calculations which are inherently uncertain. The resolution of approximately seven to ten km is deemed appropriate for urban background levels of $PM_{2.5}$ but may underestimate exposure in case of strong local $PM_{2.5}$ increments. The meteorology year is fixed to 2015.

Uncertainty in the shape of integrated exposure-response relationships (IERs) make the quantification of health burden inherently uncertain.

Different dose-response relationships are used for Europe[223] and rest of the world.[218]

The non-linearity of the CRFs used for non-European countries complicates the translation between the mortality burden attributed to an individual source, which is calculated proportional to the source contribution to ambient $PM_{2.5}$, and the effect of mitigating this source. While a reduction of emissions would lead to a roughly proportional reduction of ambient $PM_{2.5}$, this would not necessarily result in a proportional reduction of the health burden. In highly polluted environments, the health benefits of a marginal reduction of emissions would be disproportionately smaller than the relative change in concentrations.

Indicator 3.2.2 Household Air Pollution

Indicator authors

Prof Michael Davis, Shih-Che Hsu, Dr James Milner, Dr Nahid Mohajeri, Dr Jonathon Taylor

Methods

Existing estimates of global household air pollution attributable mortality from GBD and WHO are based on information on the frequency of use of different fuels in the population. These are presented relative to the outdoor air pollution estimates (e.g., the additional mortality caused by household fuels above that caused by outdoor air pollution). The new indicator complements this work via a method tailored for the *Lancet* Countdown process which can 1) link the health effects of household fuels to their role in climate change accounting for the GHG and $PM_{2.5}$ emissions, and 2) complement how outdoor air pollution mortality is estimated in the *Lancet* Countdown by using the same inputs, and 3) be updated yearly.

A Bayesian hierarchical $PM_{2.5}$ exposure model was developed using sample data of personal exposure from an updated WHO Global HAP database,[227] [206] while wood, crop residues, and dung is combined into the category of 'biomass' and LPG, Natural gas, and biogas into category of 'gas'.[207] Variables were selected from monitored data available in 282 peer-reviewed studies covering the years 1996 to 2021 to develop Bayesian models for the personal $PM_{2.5}$ exposure (sample size, n=260). Bayesian hierarchical models were built to generate accurate $PM_{2.5}$ exposure coefficients and variance around the estimates from the sample data and apply to IIASA GAINS modelled data for predicting $PM_{2.5}$ personal exposure globally. This model provides estimates on $PM_{2.5}$ personal exposure levels based on average 24-hour period.

The hierarchical model incorporating the following predictors for each country:
(i) fuel types (biomass, charcoal, coal, gas, electricity);

(ii) traditional/improved stove;
(iii) urban/rural location;
(iv) population weighted heating degree days;
(v) population weighted ambient $PM_{2.5}$;
(vi) GNI index;
(vii) Education index;
(viii) season (winter/summer/whole year)

Annual population-weighted average $PM_{2.5}$ personal exposure were estimated for 62 countries in five WHO regions (African Region, Eastern Mediterranean Region, Region of the Americas, South-East Asian Region, Western Pacific Region). We exclude the European Region due to high uncertainty in the estimated exposure values.

Attributable premature mortality due to personal exposure is estimated at national level (per 100,000 population) using the standard comparative risk assessment (CRA) approach. This involves calculation of population attributable fractions based on the estimated $PM_{2.5}$ personal exposure for each country (and separately for urban and rural populations).[227,228] This exposure is then converted into an estimate of excess deaths using Global Burden of Disease functions. We use three following weighted averages to quantify the mortality rates for the number of attributable deaths per 100,000 individuals for solid fuels at national level: (i) Proportion of people using each fuel type (biomass, charcoal, coal, gas, electricity) in each country and for urban and rural settings.[207] (ii) Proportion of people using each stove type (traditional, improved) in each country for urban and rural settings. (iii) Proportion of people living in urban and rural setting in each country.

The household air pollution model includes ambient $PM_{2.5}$ exposure from GAINS as an input. The mortality estimates currently include some degree of overlap with estimates of mortality due to ambient air pollution, which is also the case for the WHO estimates.[229]

Data

- Ambient $PM_{2.5}$ concentrations for 2020 from IIASA[216]
- Fuel Type: IIASA GAINS model via IEA[212]
- Stove Type[230]
- Heating Degree Days for the year 2000 (1985–2015) provided by NASA[231]
- Education index and GNI index provided by United Nations Development Programme (UNDP). Year 2019
- WHO Global HAP Database.[206]
- WHO. Household air pollution attributable death rate (per 100 000 population)[229]
- WHO. Proportion of population with primary reliance on fuels and technologies for cooking, by fuel type (%)[232]
- Baseline mortality data: GBD national estimates for males and females[218]
- Exposure-response functions for attributable premature mortality: GBD2019 MR-BRTs, cause, and age specific, for six diseases[221]

Caveats

The indicator provides useful information as to the variation of $PM_{2.5}$ exposure for a given fuel use and stove type and urban/rural locations as well as their health impacts. The inclusion of ambient air pollution for urban and rural locations (obtained from IIASA GAINS modelled gridded data) and the heating degree days for the same urban and rural areas are the two unique predictors used here for the first time in Bayesian $PM_{2.5}$ exposure models at global scale.

One challenge is the combination/overlap of ambient $PM_{2.5}$ and household $PM_{2.5}$ exposure, which may lead to double counting mortality.

Indoor air pollution is complex and impacted by a number of different factors including housing characteristics (e.g., ventilation rate, kitchen locations, window in kitchen, roofing materials) which are not typically captured in

all the monitored data. Updating the sample data with information on these and related factors should greatly improve the future predictions as to household air pollution.

Another challenge concerns the measured/monitored household air pollution data (e.g., studies included in the WHO database). More specifically, the concerns are as follows: rather limited number of households monitored in each study; each study uses different monitoring technology to collect the data; and data collected from different measurement periods as well as different analytic methods used for data processing in each study. Nevertheless, using Bayesian predictive models developed in this study allows us to explore a wide range of $PM_{2.5}$ exposures depending on fuel use, stove types, and for differences urban and rural locations of countries worldwide.

As regards COVID-19, several studies reported the increase in indoor exposure to $PM_{2.5}$ during the lockdown particularly in households located in rural areas.[233,234] This could potentially have negative impact on human health. Further data from the year 2020 are needed to estimate accurately the exposure and the health impact of global COVID-19 lockdowns, especially in rural areas where the use of solid fuels is very common.

Additional analysis

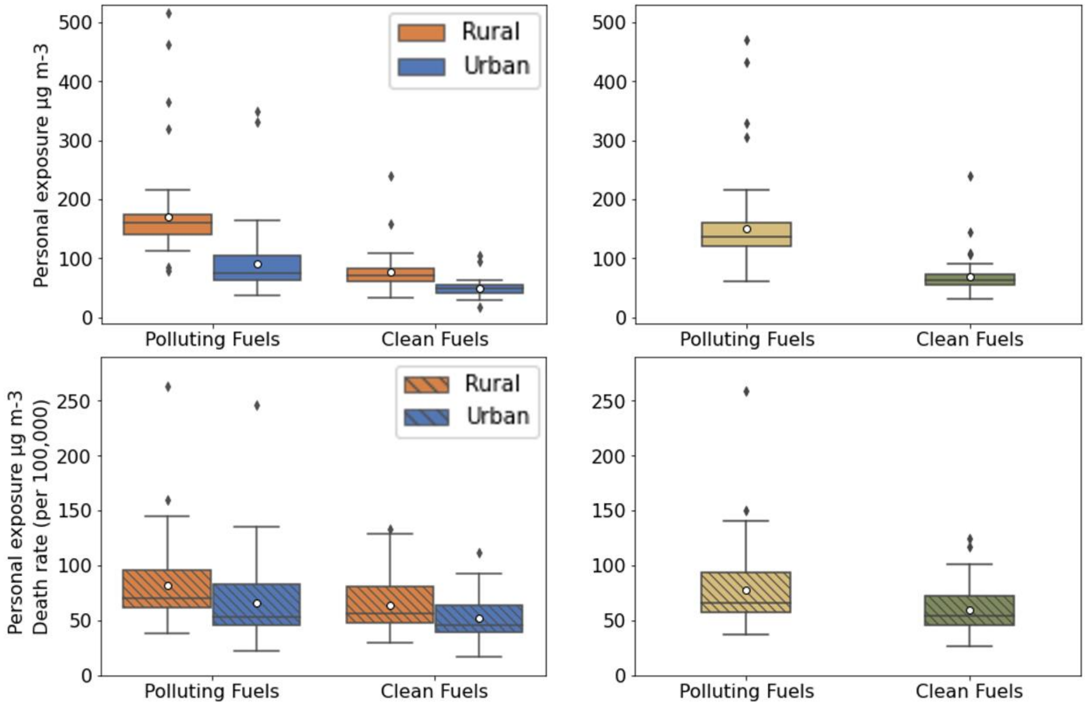

Figure 99 The estimated urban, rural, and national-level annual weighted average HAP-PM2.5 personal exposure (in µg/m3) and related attributable premature death rate (per 100,000 population) of polluting solid fuels and clean fuels.

3.3: Food, Agriculture, and Health Co-benefits

Indicator 3.3.1: Emissions from Agricultural Production and Consumption

Indicator authors

Dr Carole Dalin, Dr Harry Kennard

Methods

The 2023 update of this indicator radically increases the number of commodities considered. GHG emissions from agricultural production and consumption now incorporates new classes of fruits, vegetables, nuts, pulses and legumes and other crops. While these additional crops tend to have much lower carbon intensity than animal derived products, their inclusion provides a more complete picture of the agricultural commodities used in the global food system.

The methods by which the estimates of GHG for food products is devided into two sections, one covering livestock and the second covering crops.

Livestock products

Emissions intensities for the year 2000 are calculated in the following manner as in Dalin et al.[235] The following livestock species are included:

Ruminant	Non-Ruminant
Cattle, dairy<br><br>(FAO Item Code 960)	Chicken, broilers<br><br>(FAO Item Code 1053)
Cattle, non-dairy<br><br>(FAO Item Code 961)	Chicken, layers<br><br>(FAO Item Code 1052)
Buffaloes<br><br>(FAO Item Code 946)	Swine, market<br><br>(FAO Item Code 1049)
Goats<br><br>(FAO Item Code 1016)	Swine, breeding<br><br>(FAO Item Code 1079)
Sheep<br><br>(FAO Item Code 976)	

All livestock categories also include secondary products—such as cheese in the case of milk—where data were available. Cattle products comprise beef meat and milk and buffalo meat and milk. Sheep and goat products comprise meat and milk. Poultry products comprise meat and eggs of chickens, geese, ducks, and turkeys. Swine products include pork and secondary processed commodities, such as ham and bacon.

Emissions from enteric fermentation and manure management are obtained from Herrero et al.[236]

For manure left on pasture, rates from the GLOBIOM model were used[237] and a linear N_2O emission model applied.[238]

This information is presented in tonne carbon dioxide equivalent (CO_2e) per tropical livestock unit (tlu), which is converted to livestock head using the table below.[239]	Head per tlu
Bovine (Buffalo, Cattle (dairy), Cattle(non-dairy))	1.43
Small Ruminants (Goats, Sheep)	10
Poultry (Chicken)	100
Swine	5

The emissions per head are divided into world regions (as in the GLOBIOM model) and, for ruminants, livestock system (combination of climates from arid to humid, and practices from rangeland to feedlots, c.f. Herrero et al. 2013).[236] To convert these emissions to country values, an average is made across the region-system pairs within each country, weighted by the number of animals.

To obtain the emissions from grazing, the synthetic fertilizer applied to grassland from Chang et al.[240] is used as input to the N_2O emission model.[238] Animal products' emissions are also incorporated into the feed crop-related emissions proportionally to the feed ingredients consumed by animals—by species, region and systems— using feed data from Herrero et al. 2013.[236] These emissions from feed crops and grazed grasslands are then added to the direct livestock emissions (from enteric fermentation, manure management, and manure left on pasture) to provide overall emissions rates for each livestock species in the year 2000.

Finally, emissions intensity values for each livestock commodity (egg, meat, milk) and country are obtained by dividing CO_2e values by the output of milk/meat/egg per head from Herrero et al. 2013.[236]

Crop Products:

The emissions from fertiliser (synthetic and manure) application, rice cultivation, and cultivation from organic soils for 172 crops for the year 2000 are obtained from Carlson et al. 2017,[241] who use IPCC methodology and a non-linear N_2O emission model. Crop types corresponding to "fodder" and "fibre" types are then excluded for this report, leaving 147 crops which are directly consumed by humans.

Crops used for livestock feed are excluded from the "crops" emissions, as they are included in the intensity of livestock production; the FAO reports this in the following way: "Cereal crops harvested for hay or harvested green for food, feed or silage or used for grazing are therefore excluded".[239]

Production values 2001–2019

Since the emission intensity of production is not constant over time, its values by commodity (for both livestock and crop products) were scaled using the FAO values as an index. The FAO produces GHG emissions intensity values by animal commodity and broad crop category (distinguishing rice, which, unlike other crops, emits large amounts of methane) for the countries covered by their analysis. However, these values are volatile at the country level, so regional values were used here. The percentage change from the year 2000 value was applied to the values derived from Herrero et al.,[236] Chang et al.,[240] and Carlson et al.,[241] outlined above (methodology from Dalin et al.[235]). At the time of publication, the values for 2018 had not been published by the FAO, so the intensity scaling was assumed to be the same as in 2017. This will be updated in future years. Any missing values in scaling factor were assumed to be 1 (constant emission intensity). Any intensity values missing for a given country were given the regional average for that year and commodity, although practically this had little impact, because missing values only corresponded to countries which had very low or no production of the commodity in question.

Consumption emissions

The GHG emissions associated with agricultural commodity consumption uses FAO production and trade data to estimate the total GHG emissions footprint associated with each of the commodities considered in a given country. This method is used by Dalin et al.[242] for tracing water consumption in global food networks but is adapted here to calculated GHG footprint. The basic equation the indicator follows is:

Consumption = production + imports - exports

FAO production and trade data are used in the following manner. For a given commodity the national production values in tonnes are converted into CO_2e values using the GHG emissions intensity values supplied by indicator 3.5.1 GHG production estimates (via Carlson et al. 2017)[241] associated with producing that tonnage of the commodity. Next, secondary commodities are converted in primary equivalent values by multiplying the trade tonnage by the value derived from Dalin et al. 2017.[242] For example, the primary equivalences for wheat products are as shown in Table 62:

Bran, wheat	1.01
Bread	0.88

Bulgur	1.05
Cereals, breakfast	1.18
Flour, wheat	1.01
Macaroni	1.01
Pastry	0.88
Wafers	0.88
Wheat	1.00

Table 62: Primary equivalences for wheat products

These values are then converted into GHG emissions equivalent, based on the GHG emissions intensity. For a given year, the trade balances are corrected to take into account that a given commodity may have been produced in one country, processed in another and finally imported into a third, using an algorithm developed by Kastner et al 2011.[243]

Data

1. National annual production of animal products items (tonnes) – FAOSTAT (2022 update)[239]
2. National annual trade (country-country) of animal products items (tonnes) – FAOSTAT (2022 update) [239]
3. Correspondence of items across item lists with different grouping – FAOSTAT[239]
4. GHG production estimates including grassland and feed crop emissions (via Herrero et al. 2013 and Dalin et al. 2019)[235,236] Definitions: Animal types: bovine cattle (beef and buffalo), sheep and goat ruminants, pigs, poultry (chicken, ducks, geese and turkeys)
5. National annual production of crops (tonnes) – FAOSTAT (2022 update) [239]
6. National annual trade (country-country) of crop products (tonnes) – FAOSTAT (2022 update) [239]
7. GHG emissions intensity of crop products for each country– provided by Carlson et al. (2017)[241]

Caveats

In the context of this indicator, *consumption* refers to the net balance of food products entering a country within a given year, i.e., national production and *net* imports together, which could also be referred to as "national supply". Here net imports refers to imports minus exports. It does not refer to the total GHG emissions attributable to food consumed by individuals. Indeed, at present, this indicator only considers the emissions associated with food production described above and does not take into account emissions associated with food transport and processing, storage and decomposition, and use change and deforestation.[244]

This indicator does not account for emissions associated with land conversion to agriculture (such as deforestation) but does consider emissions form cultivation of organic soils (such as peatland).

For livestock, data on stock numbers has been extracted from FAO database, however, some data is missing for some years, most notably Somalia (missing data 2000–2011) for non-dairy cattle. Data on grazing emissions from small islands is also missing, and therefore imputed using regional average values as described above.

The emission factors differ from FAO numbers:

- For livestock, this is due to calculation of emissions of enteric fermentation, manure management and manure left on pasture at GLOBIOM region (n=29) and livestock system (n=8) level whereas the FAO use subcontinental (n=9) and climatic level (n=3).[239]

- For crops, this is due to the FAO assuming slightly higher synthetic N application, greater manure N inputs, and a linear emissions factor of 1%, in contrast to a mean of 0.77% used by the non-linear model of Carlson et al. (2017).[241]

Agricultural consumption emissions estimates are derived directly from FAO trade values (re-organised as producer-consumer trade only with the algorithm), as described above. Therefore, these values differ from the production estimates, which are based on extrapolating year 2000 figures. On average across all years, the estimate of total emissions due to consumption are 2.25% above production values, and do not differ by more than 10% in any given year. The sole exception to this is the estimates of the differences between production and consumption by WHO region shown in the figure in the main text. For this figure the production values are derived directly from FAO values.

Additional analysis

A substantial amount of CO_2e is associated with food that it is not consumed, whether that be during the food production process, transportation loses, or being wasted at the plate. The volumes of food considered here include food that is wasted or lost in transport, but not the additional emissions associated with the decomposition of food waste. The IPCC estimates that between 8–10% of total anthropogenic GHG emissions are associated with food loss and waste.[245] However, wastage is not equally distributed by country, with one analysis finding that high income nations waste six times by weight the amount that low-income ones.[246]

Indicator 3.3.2 Diet and Health Co-benefits

Indicator authors

Dr Harry Kennard, Prof Marco Springmann

Methods

Baseline consumption data

Baseline food consumption was estimated by adopting estimates of food availability from the FAO's food balance sheets, and adjusting those for the amount of food wasted at the point of consumption.[260,261] This proxy for food consumption was disaggregated by age and sex by adopting the same age and sex-specific trends as observed in dietary surveys.[247]

An alternative would have been to rely on a set of consumption estimates that has been based on a variety of data sources, including dietary surveys, household budget and expenditure surveys, and food availability data.[248,249] However, neither the exact combination of these data sources, nor the estimation model used to derive the data have been made publicly available. For some individual countries, using dietary surveys would also have been an alternative. However, underreporting is a persistent problem in dietary surveys,[250,251] and regional differences in survey methods would have meant that the results would not be comparable between countries. In contrast to dietary surveys, waste-adjusted food-availability estimates indicate levels of energy intake per region that reflect differences in the prevalence of overweight and obesity across regions.[252]

Food balance sheets report on the amount of food that is available for human consumption.[253] They reflect the quantities reaching the consumer, but do not include waste from both edible and inedible parts of the food commodity occurring in the household. As such, the amount of food actually consumed may be lower than the quantity shown in the food balance sheet depending on the degree of losses of edible food in the household, e.g., during storage, in preparation and cooking, as plate-waste, quantities fed to domestic animals and pets, or thrown away.

The waste-accounting methodology developed by the FAO was followed to account for the amount of food wasted at the household level that was not accounted for in food availability estimates.[254] Table 63 provides an overview of the parameters used in the calculation.

For each commodity and region, food consumption was estimated by multiplying food availability data with conversion factors (*cf*) that represent the amount of edible food (e.g., after peeling) and with the percentage of food wasted during consumption (*1-wp(cns)*). The difference in wastage for roots and tubers, fruits and vegetables, and fish and seafood, also accounted for differences between the proportion that is utilised fresh

(pct_{frsh}) and the proportion that utilised in processed form (pct_{prcd}). The equation used for each food commodity and region was:

$$Consumption = Availability \cdot \frac{pct_{frsh}}{100} \cdot cf_{frsh} \cdot \left(1 - \frac{wp(cns_{frsh})}{100}\right)$$

$$+ Availability \cdot \frac{pct_{prcd}}{100} \cdot cf_{prcd} \cdot \left(1 - \frac{wp(cns_{prcd})}{100}\right)$$

Food group	Item	Region						
		Europe	USA, Canada, Oceania	Indus-trialized Asia	Sub-Saharan Africa	North Africa, West and Central Asia	South and Southeast Asia	Latin America
cereals	wp(cns)	25	27	20	1	12	3	10
roots and tuber	pct_{prcd}	73	73	15	50	19	10	80
	wp(cns)	17	30	10	2	6	3	4
	wp(cns_{prcd})	12	12	12	1	3	5	2
oilseeds and pulses	cns	4	4	4	1	2	1	2
fruits and vegetables	pct_{prcd}	60	60	4	1	50	5	50
	wp(cns)	19	28	15	5	12	7	10
	wp(cns_{prcd})	15	10	8	1	1	1	1
milk and dairy	wp(cns)	7	15	5	0.1	2	1	4
eggs	wp(cns)	8	15	5	1	12	2	4
meat	wp(cns)	11	11	8	2	8	4	6
fish and seafood	pct_{prcd}	*40% for low-income countries, and 96% for all others.*						
	wp(cns)	11	33	8	2	4	2	4
	wp(cns_{prcd})	10	10	7	1	2	1	2

Conversion factors: maize, millet, sorghum: 0.69; wheat, rye, other grains: 0.78; rice: 1; roots: 0.74 (0.9 for industrial processing); nuts and seeds: 0.79; oils: 1; vegetables: 0.8 (0.75 for industrial processing); fruits: 0.8 (0.75 for industrial processing); beef: 0.715; lamb: 0.71; pork: 0.68; poultry: 0.71; other meat: 0.7; milk and dairy: 1; fish and seafood: 0.5; other crops: 0.78

Table 63. Percentage of food wasted during consumption (cns), and percentage of processed utilisation (pctprcd). The percentage of fresh utilisation is calculated as 1-pctprcd. Conversion factors to edible portions of foods are provided below the table.

Comparative risk assessment

The mortality and disease burden attributable to dietary and weight-related risk factors was estimated by calculating population impact fractions (PIFs) which represent the proportions of disease cases that would be avoided when the risk exposure was changed from a baseline situation to a counterfactual situation. For calculating PIFs, using the general formula:[255–257]

$$PIF = \frac{\int RR(x)P(x)dx - \int RR(x)P'(x)dx}{\int RR(x)P(x)dx}$$

where $RR(x)$ is the relative risk of disease for risk factor level x, $P(x)$ is the number of people in the population with risk factor level x in the baseline scenario, and $P'(x)$ is the number of people in the population with risk factor level x in the counterfactual scenario. It was assumed that changes in relative risks follow a dose-response relationship,[256]and that PIFs combine multiplicatively, i.e. $PIF = 1 - \prod_i (1 - PIF_i)$ where the i's denote independent risk factors. [256,258]

The number of avoided deaths due to the change in risk exposure of risk *i*, $\Delta deaths_i$, was calculated by multiplying the associated PIF by disease-specific death rates, *DR*, and by the number of people alive within a population, *P*:

$$\Delta deaths_i(r, s, a, d) = PIF_i(r, s, a, d) \cdot DR(r, s, a, d) \cdot P(r, s, a)$$

where PIFs are differentiated by region *r*, sex *s*, age group *a*, and disease/cause of death *d*; the death rates are differentiated by region, sex, age group, and disease; the population groups are differentiated by region, sex, and age group; and the change in the number of deaths is differentiated by region, sex, age group, and disease.

Publicly available data sources were used to parameterize the comparative risk analysis. Mortality and population data were adopted from the Global Burden of Disease project.[259] Baseline data on the weight distribution in each country were adopted from a pooled analysis of population-based measurements undertaken by the NCD Risk Factor Collaboration.[252]

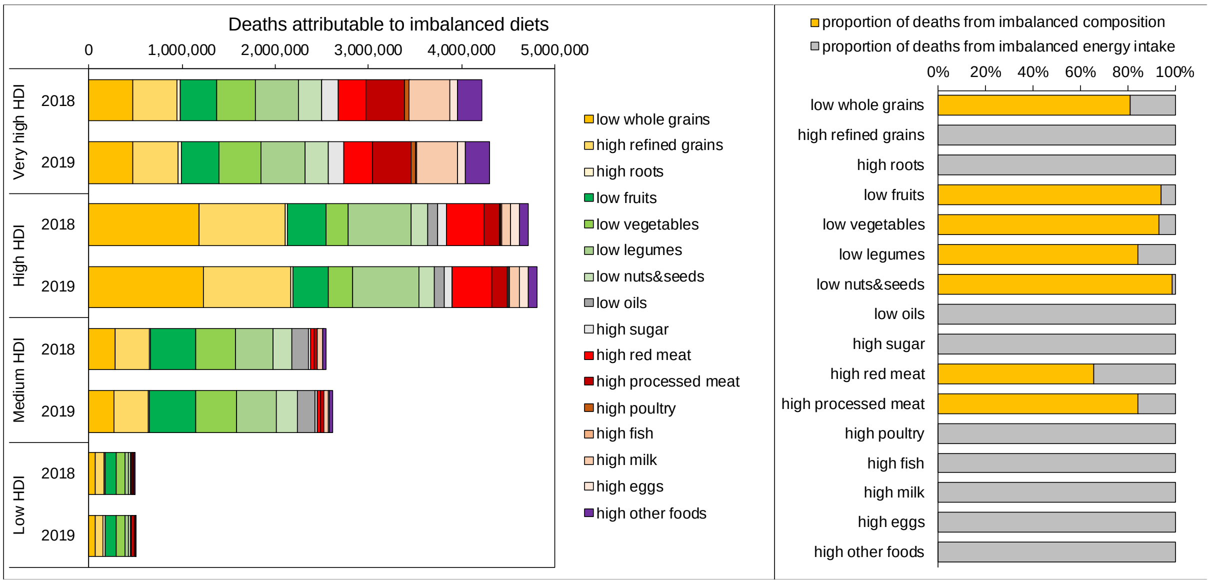

Figure 100: Deaths attributable to diet-related risks in 2018 and 2019 by risk factor and development region (left) and composition of attributable deaths into those from imbalanced composition and imbalanced energy intake (right).

The relative risk estimates that relate the risk factors to the disease endpoints were adopted from meta-analyses of prospective cohort studies for dietary and weight-related risks.[260] In line with the meta-analyses, non-linear dose-response relationships were included for fruits, vegetables, and nuts and seeds, and assumed linear dose-response relationships for the remaining risk factors. As the analysis was primarily focused on mortality from chronic diseases, the focus was on adults aged 20 year or older, and the relative-risk estimates were adjusted for attenuation with age based on a pooled analysis of cohort studies focussed on metabolic risk factors,[261]in line with other assessments.[257,262]

Table 64 provides an overview of the relative-risk parameters used. For the counterfactual scenario, minimal risk exposure levels (TMRELs) was defined as follows: 300 g/d for fruits, 500 g/d for vegetables, 100 g/d for legumes, 20 g/d for nuts and seeds, 125 g/d for whole grains, 0 g/d for red meat, 0 g/d for processed meat, and no underweight, overweight, or obesity. The TMRELs are in line with those defined by the Nutrition and Chronic Diseases Expert Group (NutriCoDE),[262] with the exception that a higher value for vegetables was used,

and zero was used as minimal risk exposure for red meat, in each case based on a more comprehensive meta-analysis.[263,264]

The selection of risk-disease associations used in the health analysis was supported by available criteria used to judge the certainty of evidence, such as the Bradford-Hill criteria used by the Nutrition and Chronic Diseases Expert Group (NutriCoDE),[262] the World-Cancer-Research-Fund criteria used by the Global Burden of Disease project,[278] as well as NutriGrade (Table 64).[265] The certainty of evidence supporting the associations of dietary risks and disease outcomes as used here were graded as moderate or high with NutriGrade,[264,266,267] and/or assessed as probable or convincing by the Nutrition and Chronic Diseases Expert Group,[262]and by the World Cancer Research.[268] The certainty of evidence grading in each case relates to the general relationship between a risk factor and a health outcome, and not to a specific relative-risk value.

Food group	Endpoint	Unit	RR mean	RR low	RR high	Reference
Processed meat	CHD	50 g/d	1.27	1.09	1.49	Bechthold et al (2019)
	Stroke	50 g/d	1.17	1.02	1.34	Bechthold et al (2019)
	Colorectal cancer	50 g/d	1.17	1.10	1.23	Schwingshackl et al (2018)
	Type 2 diabetes	50 g/d	1.37	1.22	1.55	Schwingshackl et al (2017)
Red meat	CHD	100 g/d	1.15	1.08	1.23	Bechthold et al (2019)
	Stroke	100 g/d	1.12	1.06	1.17	Bechthold et al (2019)
	Colorectal cancer	100 g/d	1.12	1.06	1.19	Schwingshackl et al (2018)
	Type 2 diabetes	100 g/d	1.17	1.08	1.26	Schwingshackl et al (2017)
Fruits	CHD	100 g/d	0.95	0.92	0.99	Aune et al (2017)
	Stroke	100 g/d	0.77	0.70	0.84	Aune et al (2017)
	Cancer	100 g/d	0.94	0.91	0.97	Aune et al (2017)
Vegetables	CHD	100 g/d	0.84	0.80	0.88	Aune et al (2017)
	Cancer	100 g/d	0.93	0.91	0.95	Aune et al (2017)
Legumes	CHD	57 g/d	0.86	0.78	0.94	Afshin et al (2014)
Nuts	CHD	28 g/d	0.71	0.63	0.80	Aune et al (2016)
Whole grains	CHD	30 g/d	0.87	0.85	0.90	Aune et al (2016b)
	Cancer	30 g/d	0.95	0.93	0.97	Aune et al (2016b)
	Type 2 diabetes	30 g/d	0.65	0.61	0.70	Aune et al (2016b)
Underweight	CHD	15<BMI<18.5	1.17	1.09	1.24	Global BMI Collab (2016)
	Stroke	15<BMI<18.5	1.37	1.23	1.53	Global BMI Collab (2016)
	Cancer	15<BMI<18.5	1.10	1.05	1.16	Global BMI Collab (2016)
	Respiratory disease	15<BMI<18.5	2.73	2.31	3.23	Global BMI Collab (2016)
Overweight	CHD	25<BMI<30	1.34	1.32	1.35	Global BMI Collab (2016)
	Stroke	25<BMI<30	1.11	1.09	1.14	Global BMI Collab (2016)
	Cancer	25<BMI<30	1.10	1.09	1.12	Global BMI Collab (2016)
	Respiratory disease	25<BMI<30	0.90	0.87	0.94	Global BMI Collab (2016)
	Type 2 diabetes	25<BMI<30	1.88	1.56	2.11	Prosp Studies Collab (2009)
Obesity (grade 1)	CHD	30<BMI<35	2.02	1.91	2.13	Global BMI Collab (2016)
	Stroke	30<BMI<35	1.46	1.39	1.54	Global BMI Collab (2016)
	Cancer	30<BMI<35	1.31	1.28	1.34	Global BMI Collab (2016)
	Respiratory disease	30<BMI<35	1.16	1.08	1.24	Global BMI Collab (2016)
	Type 2 diabetes	30<BMI<35	3.53	2.43	4.45	Prosp Studies Collab (2009)
Obesity (grade 2)	CHD	30<BMI<35	2.81	2.63	3.01	Global BMI Collab (2016)
	Stroke	30<BMI<35	2.11	1.93	2.30	Global BMI Collab (2016)
	Cancer	30<BMI<35	1.57	1.50	1.63	Global BMI Collab (2016)
	Respiratory disease	30<BMI<35	1.79	1.60	1.99	Global BMI Collab (2016)
	Type 2 diabetes	30<BMI<35	6.64	3.80	9.39	Prosp Studies Collab (2009)
Obesity (grade 3)	CHD	30<BMI<35	3.81	3.47	4.17	Global BMI Collab (2016)
	Stroke	30<BMI<35	2.33	2.05	2.65	Global BMI Collab (2016)
	Cancer	30<BMI<35	1.96	1.83	2.09	Global BMI Collab (2016)
	Respiratory disease	30<BMI<35	2.85	2.43	3.34	Global BMI Collab (2016)
	Type 2 diabetes	30<BMI<35	12.49	5.92	19.82	Prosp Studies Collab (2009)

Table 64 Relative risk parameters (mean and low and high values of 95% confidence intervals) for dietary risks and weight-related risks.

Not all available risk-disease associations that were graded as having a moderate certainty of evidence and showed statistically significant results in the meta-analyses that included NutriGrade assessments were included in the analysis. [264,266,267] That was because for some associations, such as for milk and fish, more detailed meta-analyses (with more sensitivity analyses) were available that indicated potential confounding with other major

dietary risks or health status at baseline.[269–271] Such sensitivity analyses were not presented in the meta-analyses that included NutriGrade assessments, but they are important for health assessments that evaluate changes in multiple risk factors (Table 65).

Food group	Endpoint	Association	Certainty of evidence
Fruits	CHD	reduction	NutriCoDE: probable or convincing;<br>NutriGrade: moderate quality of meta-evidence
	Stroke	reduction	NutriCoDE: probable or convincing<br>NutriGrade: moderate quality of meta-evidence
	Cancer	reduction	WCRF: strong evidence (probable) for some cancers<br>NutriGrade: moderate quality of meta-evidence for colorectal cancer
Vegetables	CHD	reduction	NutriCoDE: probable or convincing<br>NutriGrade: moderate quality of meta-evidence
	Cancer	reduction	WCRF: strong evidence (probable) for non-starchy vegetables and some cancers<br>NutriGrade: moderate quality of meta-evidence for colorectal cancer
Legumes	CHD	reduction	NutriCoDE: probable or convincing<br>NutriGrade: moderate quality of meta-evidence
Nuts and seeds	CHD	reduction	NutriCoDE: probable or convincing<br>NutriGrade: moderate quality of meta-evidence
Whole grains	CHD	reduction	NutriCoDE: probable or convincing<br>NutriGrade: moderate quality of meta-evidence
	Cancer	reduction	WCRF: strong evidence (probable) for colorectal cancer<br>NutriGrade: moderate quality of meta-evidence for colorectal cancer
	Type-2 diabetes	reduction	NutriCoDE: probable or convincing<br>NutriGrade: high quality of meta-evidence
Red meat	CHD	increase	NutriGrade: moderate quality of meta-evidence
	Stroke	increase	NutriGrade: moderate quality of meta-evidence
	Cancer	increase	WCRF: strong evidence (probable) for colorectal cancer<br>NutriGrade: moderate quality of meta-evidence for colorectal cancer
	Type-2 diabetes	increase	NutriCoDE: probable or convincing<br>NutriGrade: high quality of meta-evidence
Processed meat	CHD	increase	NutriCoDE: probable or convincing<br>NutriGrade: moderate quality of meta-evidence
	Stroke	increase	NutriGrade: moderate quality of meta-evidence
	Cancer	increase	WCRF: strong evidence (convincing) for colorectal cancer<br>NutriGrade: moderate quality of meta-evidence for colorectal cancer
	Type-2 diabetes	increase	NutriGrade: high quality of meta-evidence

NutriCoDE: Nutrition and Chronic Diseases Expert Group

NutriGrade: Grading of Recommendations Assessment, Dvelopment, and Evaluation (GRADE) tailored to nutrition research

WCRF: World Cancer Research Fund

Table 65 Overview of existing ratings on the certainty of evidence for a statistically significant association between a risk factor and a disease endpoint. The ratings include those of the Nutrition and Chronic Diseases Expert Group (NutriCoDE),[262] the World Cancer Research Fund,[268] and NutriGrade.[264,266,267] The ratings relate to the risk-disease associations in general, and not to the specific relative-risk factor used for those associations in this analysis.

Weight-related risks are connected to imbalanced energy intake. To highlight this connection, the weight-related disease burden was attributed to consuming too much or too little of specific foods. For that purpose, the current energy intake by food group in each country was first compared to a dietary pattern that minimises both diet and weight-related risks, and then attributed the proportion of energy intake of under and over-consumed foods to the proportion of deaths attributable to underweight on the one hand and to overweight and obesity on the other. The minimal-risk patterns were based on recommendations for optimal energy intake given the sex, age, and height structure of each country,[252,272] the TMREL values used in the dietary risk assessment,[218,260,262,263,266,273,274] and food-based recommendations for healthy and sustainable diets for the

remaining food groups.[291] The recommendations were implemented as minimum and maximum values, which preserved a country's intake if it was within recommendations (Table 66).

Food group	Recommended intake		Source
	Min	Max	
Fruits	300	>300	Aune et al (2017)
Vegetables	500	>500	Aune et al (2017)
Legumes	100	>100	Micha et al (2017), Afshin et al (2017)
Nuts and seeds	20	>20	Micha et al (2017), Aune et al (2016a)
Whole grains	125	225	Micha et al (2017), Aune et al (2016b)
Red meat	0	0	GBD 2019 (2020), Bechthold et al (2017)
Processed meat	0	0	GBD 2019 (2020), Bechthold et al (2017)
Oils	40	80	Willett et al (2019)
Sugar	0	31	Willett et al (2019)
Roots	0	100	Willett et al (2019)
Milk	0	250	Willett et al (2019)
Eggs	0	13	Willett et al (2019)
Poultry	0	29	Willett et al (2019)
Fish	0	28	Willett et al (2019)

Table 66 Food-based recommendations used to construct minimal risk dietary patterns. The recommendations include minimal risk exposure levels for dietary risks (upper rows) and food-based recommendations for a healthy and sustainable diets (lower rows).

For the different diet scenarios, uncertainty intervals were calculated associated with changes in mortality based on standard methods of error propagation and the confidence intervals of the relative risk parameters. For the error propagation, the error distribution was approximated of the relative risks by a normal distribution and used that side of deviations from the mean which was largest. This method leads to conservative and potentially larger uncertainty intervals as probabilistic methods, such as Monte Carlo sampling, but it has significant computational advantages, and is justified for the magnitude of errors dealt with here (<50%) (see e.g., IPCC Uncertainty Guidelines).

Data

Table 67 provides an overview of the data sources used for this indicator.

Type	Coverage	Source
Exposure data:		
Food consumption data	Country-level	Food availability data adjusted for food waste at the household level and for age and sex-specific trends.[247,253,254] Estimates of energy intake were in line with trends in body weight across countries.[252]
Weight estimates	Country-level	Baseline data from pooled analysis of measurement studies differentiated by sex and age with global coverage.[252]
Health analysis:		

Relative risk estimates	General	Adopted from meta-analysis of prospective cohort studies.[260,263,264,266,267,273,275] The certainty of evidence for the risk-disease associations were rated as moderate to high by NutriGrade.[264,266,267]
Mortality and population data	Country-level	Adopted from the Global Burden of Disease project by country, sex, and age group.[259]

Table 67 Overview of data sources

Caveats

In the comparative risk assessment, relative risk factors were used that are subject to the caveats common in nutritional epidemiology, including small effect sizes and potential measurement error of dietary exposure, such as over and underreporting and infrequent assessment.[276] For the calculations, it was assumed that the risk-disease relationships describe causal associations, an assumption supported by the existence of statistically significant dose-response relationships in meta-analyses, the existence of plausible biological pathways, and supporting evidence from experiments, e.g., on intermediate risk factors.[260,262,263,266,266,267,274,275,277–279] However, residual confounding with unaccounted risk factors cannot be ruled out in epidemiological studies. Additional aspects rarely considered in meta-analyses are the importance of substitution between food groups that are associated with risks, and the time lag between dietary exposure and disease.

To address potential confounding, risk-disease associations were omitted that became non-significant in fully adjusted models, in particular those related milk intake,[269,270] and to fish intake.[271,278,280] The quality of evidence in meta-analyses that covered the same risk-disease associations as used here was graded with NutriGrade as moderate or high for all risk-disease pairs included in the analysis (Table 64). [264,266,267] In addition, the Nutrition and Chronic Diseases Expert Group and the World Cancer Research Fund graded the evidence for a causal association of ten of the 12 risk-disease associations included in the analysis as probable or convincing,[262,268] The relative health ranking of leading risk factors found in the analysis was similar to existing rankings that relied on different relative-risk parameters and exposure data.[267]

As exposure data, a proxy of food consumption was used that was derived from estimates of food availability that were adjusted for the amount of food wasted at the point of consumption.[253,254] An alternative would have been to rely on a set of consumption estimates that has been based on a variety of data sources, including dietary surveys, household budget and expenditure surveys, and food availability data.[248,249] However, neither the exact combination of these data sources, nor the estimation model used to derive the data have been made publicly available. For some individual countries, using dietary surveys would also have been an alternative. However, underreporting is a persistent problem in dietary survey,[250,251] and regional differences in survey methods would have meant that the results would not be comparable between countries. In contrast to dietary surveys, waste-adjusted food-availability estimates indicate levels of energy intake per region that reflect differences in the prevalence of overweight and obesity across regions.[252]

Indicator 3.4: Healthcare Sector Emissions

Indicator authors

Dr Matthew Eckelman, Dr Jodi D. Sherman

Methods

This indicator is in the form of healthcare-associated GHG emissions per capita per year, including direct emissions from healthcare facilities as well as emissions from the consumption of goods and services supplied by other sectors. Results are calculated by assigning aggregate national health expenditures from WHO to final demand for 'Health and Social Work' sectors in the EE-MRIO model. Environmental satellite accounts including GHG emissions accompany each EE-MRIO model. Consumption-based GHG emissions are then calculated using the standard Leontief inverse technique[281]

Modeling for prior years of the *Lancet Countdown* report utilized the WIOD MRIO model; however, the most recent emissions satellite accounts for this model date to 2016 for carbon dioxide emissions or to 2013 when considering other air pollutant emissions. Therefore, in this year's report, the most recent version (v3.8.2) of the EXIOBASE MRIO model was used, which includes macroeconomic tables across multiple years, including the current model year 2020.

EXIOBASE uses euros as the currency unit. Per capital WHO expenditure data in 2020 US dollars were converted to 2020 using the average exchange rate for that year (1 USD = 0.893 EUR) as expressed in the WHO health accounts for that year. Because the expenditure and model years were the same, no deflation adjustments were necessary.

EXIOBASE v3.8.2 satellite accounts include both uncharacterized emissions (physical quantities) and characterized emissions (impacts). Here, the characterized emissions intensities were used for GHG emissions (in CO_2-equivalents) per million 2020 EUR for estimation of health sector carbon footprints. For estimation of public health damages, separate factors for disability-adjusted life-years (DALYs) per million 2020 EUR were used for emissions of $PM_{2.5}$ and ozone precursors.

Data

1. Environmentally extended multi-region input-output tables: EXIOBASE v3.8.3 model for year 2020.
2. Per capita health expenditure data is from the World Health Organization's Global Health Expenditure Database; the latest reporting year is 2020.[282]

Caveats

As only total health expenditure data are available from WHO, all expenditures are assigned to Final Demand, with no separation for investment.

MRIO models are built from aggregated top-down statistical data. Results do not reflect individual health care systems' power purchase agreements for renewable energy or any offsetting activities. Results do not include direct emissions of waste anaesthetic gases from clinical operations nor emissions from metered dose inhalers, as these are not currently reported consistently in national emissions inventories.

Section 4: Economics and Finance

Lead Author: Prof Paul Ekins

Research Fellow: Dr Daniel Scamann

4.1: The Economic Impact of Climate Change and its Mitigation

Indicator 4.1.1: Economic Losses due to Weather-Related Extreme Events

Indicator authors

Dr Daniel Scamman

Methods

The Swiss Re Institute provided the data for this indicator. The Swiss Re Institute sigma catastrophe database is an international commercial database recording both natural and man-made disasters from 1970 and has over 12,000 entries.

The term 'natural catastrophe' refers to an event caused by natural forces. Such an event generally results in a large number of individual losses involving many insurance policies. The scale of the losses resulting from a catastrophe depends not only on the severity of the natural forces concerned, but also on man-made factors, such as building design or the efficiency of disaster control in the afflicted region.

Natural catastrophes are categorised as shown in Table 68.

Category	Peril Group	Peril
	Earthquake	Earthquake
		Tsunami
		Volcano eruption
	Weather-related	Storm
		Flood
		Hail
		Cold, frost
		Drought, bush fires, heat waves
		Other natural catastrophes

Table 68: Categorisation of natural catastrophes in the data provided by the Swiss Re Institute

For this indicator, only data for 'weather-related' events is presented.

Total (insured and uninsured) economic losses reported by Swiss Re are all the financial losses directly attributable to a major event, i.e., damage to buildings, infrastructure, vehicles etc. This also includes losses due to business interruption as a direct consequence of the property damage. Insured losses are gross of any reinsurance, be it provided by commercial or government schemes. Total loss figures do not include indirect financial losses – i.e., loss of earnings by suppliers due to disabled businesses, estimated shortfalls in GDP and non-economic losses, such as loss of reputation or impaired quality of life. Insured losses refer to all insured losses except liability. To calculate uninsured losses, insured losses are subtracted from total losses.

Data are collected from a variety of sources, both internal and external. These include professional insured claims aggregators as well as insurance associations. Among the sources are also official government data, when available. Economic loss data can be estimated on the basis of Swiss Re proprietary catastrophe risk models. Also, if insured loss data are available, economic loss data are estimated on the basis of the local insurance penetration and other event-specific information (such as damages to public infrastructure, number of buildings damaged or destroyed etc.).

Minimum threshold apply to inclusion in the database. At least one of the following must apply, for events recorded in 2022 (with economic values changing each year following changes to US CPI):

- **Insured losses (claims):** $25.2 million (maritime disasters), $50.4 million (aviation), $62.5 million (other)
- **Economic losses**: $120.6 million
- **Casualties**: Dead or missing: 20; Injured: 50; Homeless: 2000

Loss values are presented in US$, or if initially expressed in local currency, converted to US$ using year-end exchange rates.

Prior to the 2021 report, country data were then summed into the four World Bank income groups. From the 2021 report, country data are summed into the four HDI classifications (Very High, High, Medium, Low). Data is also presented according to WHO regions and LC groupings. Further information on the methodology of the sigma explorer database can be found here: https://www.sigma-explorer.com/documentation/Methodology_sigma-explorer.com.pdf. Total insured and uninsured losses are then divided by total GDP for each year and HDI group. GDP data are taken from the IMF's World Economic Outlook (October 2022 Edition). All values reported for this indicator are in $2022.

Data

1. Swiss Re Institute sigma catastrophe database[283]
2. IMF World Economic Outlook (October 2022)[284]

Caveats

Only events with measurable economic losses above the threshold levels are included. Each natural catastrophe event recorded is assigned a direct economic loss, and where applicable, an insured loss. Where available, data is taken from official institutions, but where not, estimates are calculated. The process for estimation depends on what data is available. For example, if loss estimates from insurance market data is available, this data may be combined with data on insurance penetration and other event-specific information to estimate total economic losses. If only low-quality information is available, such as a description of the number of homes damaged or destroyed, assumptions on value and costs are made. Some data (including both losses and GDP values) may be revised compared to previous reports, due to updated information or detailed measurement approaches.

Additional analysis

Charts showing losses as a fraction of GDP are shown below grouped according to HDI band, WHO region and LC group. The underlying data for these charts is available in the online data explorer.

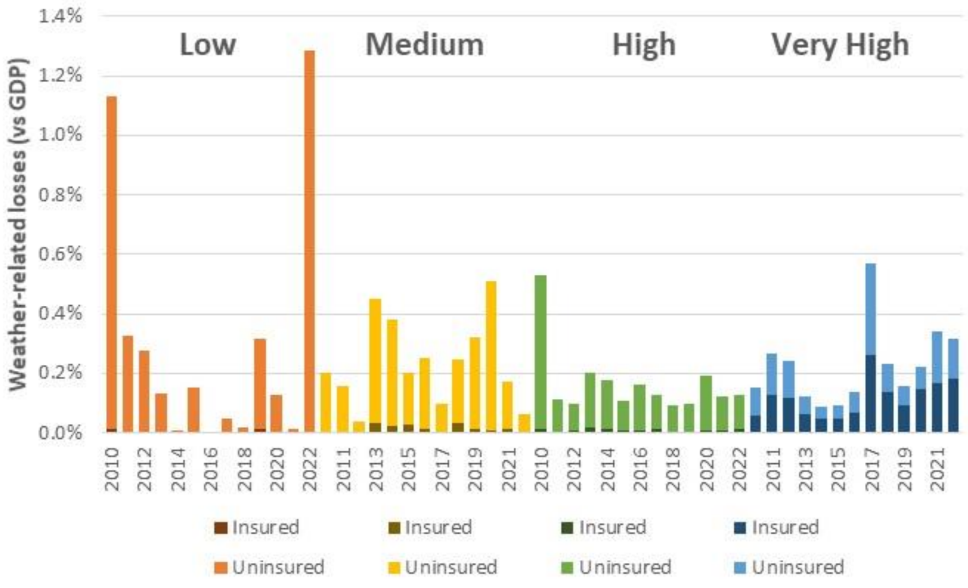

Figure 101: Insured and uninsured losses from weather-related extreme events vs GDP 2010–2022, by HDI band.

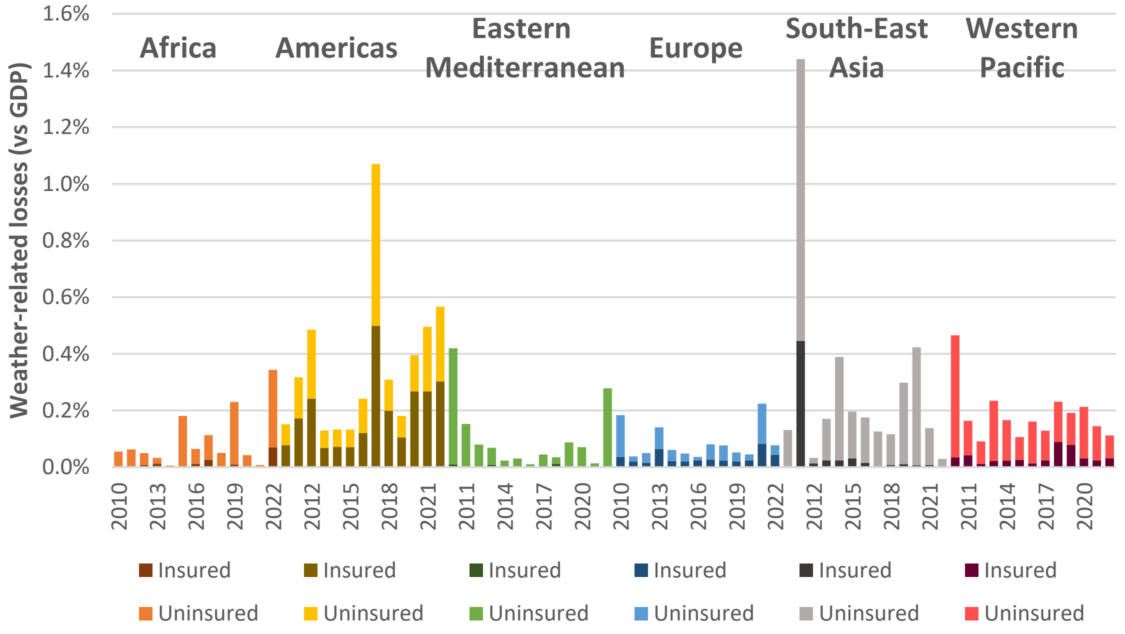

Figure 102: Insured and uninsured losses from weather-related extreme events vs GDP 2010–2022, by WHO region.

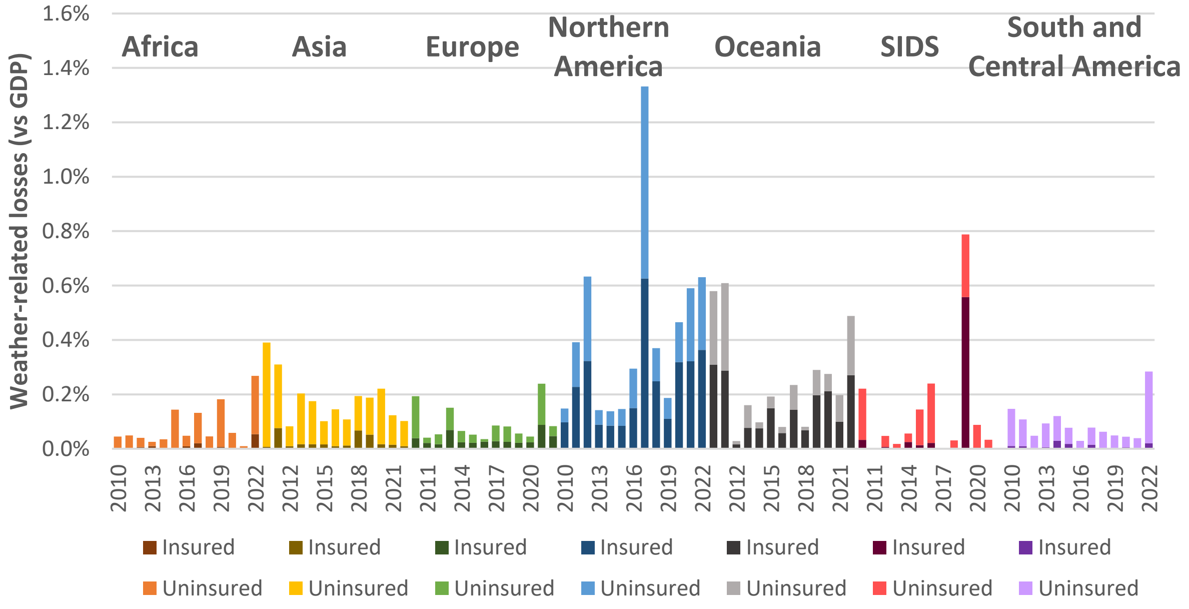

Figure 103: Insured and uninsured losses from weather-related extreme events vs GDP 2010–2022, by LC group.

Indicator 4.1.2: Costs of Heat-related Mortality

Indicator authors

Prof Wenjia Cai, Dr Shihui Zhang

Methods

This indicator used the value of statistical life-year (VSLY) to monetise the years of life lost (YLL) caused by heat-related mortality (data for which is provided by indicator 1.1.5). Compared to last year's method that used the value of a statistical life (VSL) to monetise mortality, the usage of VSLY can reflect age structure differences of heat-related mortalities across countries. VSLY measures how people value the discounted years of remaining life.[285] VSL can be interpreted as the discounted sum of VSLY of each year remained in life, therefore, mathematically, the VSLY can be derived from the VSL and how many remaining years people are expected to live at certain age (Eq.2). As for the change of VSLY to age, some studies assumed that VSLY is constant across age span, while others assumed that VSLY will increase before mid-age and then decrease till death, which is an Inverted-U shape.[286] To avoid ethical challenges caused by variation of life across different income levels, the economic costs are expressed as a proportion of GDP and the equivalent of annual average income. 169 countries spanning six World Health Organization (WHO) regions were included in the estimation. Population and GDP per capita are taken from the World Bank and OECD statistics.[287,288]

The same ratio between VSLY and GDP-per-capita is assumed for each country for years 2000-2022, and data from OCED countries was used as the basis to derive the ratio on account of data availability and method consistency across reports in different years. The assumption is shown in Eq. (1), where *Y* denotes the gross domestic product (GDP) per capita, *i* denotes the country *i* in WHO regions, *t* denotes time.

$$\frac{VSLY_{it}}{Y_{it}} = \frac{VSLY_{OECD}}{Y_{OECD}} \quad (1)$$

The relationship between VSL and VSLY can be obtained by years of remaining life at death (L) and discount rate (r), as shown in Eq.(2). The average VSL for OECD countries (VSL_{OECD}) was estimated at US$ 3.83 million ($2015) in 2015, and the average GDP per capita for OECD countries was $40,494 ($2015) in 2015. Equation 2 indicates that the total discounted value of each year's life value (VSLY) is equal to the value in the whole life span (VSL). Here it is assumed the VSLY remains constant for each remaining life year because only mortality of people aging over 65 is considered, where the fluctuations of VSLYs are very small even under the Inverted-U assumption.[286] Here the average remaining years of life for a middle-aged person is estimated as 40 years as is common practice in studies that translate VSL into VSLY. In economic studies, the discount rate is usually 3-5%, here we use 3%.

$$VSLY_{it} = \frac{VSL_{it} \cdot r}{1-(1+r)^{-L}} \quad (2)$$

In order to calculate the monetised value of years of life loss (YLL) relative to per-capita GDP (*R*), Eq.(3) was applied, where YLL is multiplied by the fixed VSLY-to-GDP per capita-ratio produced by Eq.(1).

$$R_{it} = \frac{VSLY_{it} * YLL_{it}}{Y_{it}} = \frac{VSLY_{OECD}}{Y_{OECD}} * YLL_{it} \quad (3)$$

In order to calculated to monetised value of years of life loss as a proportion of GDP (V), Eq.(4) was applied, where YLL as a proportion of total population (P) is multiplied by the fixed VSLY-to-GDP per capita-ratio in OECD countries.

$$V_{it} = \frac{VSLY_{it} * YLL_{it}}{GNI_{it}} = \frac{VSLY_{it} * YLL_{it}}{Y_{it} * P_{it}} = \frac{VSLY_{OECD}}{Y_{OECD}} * \frac{YLL_{it}}{P_{it}} \quad (4)$$

Country-level results are aggregated according both to WHO regions and HDI level. Considering data availability, some countries in WHO regions are not included: Cabo Verde, Sao Tome and Principe, Saint Vincent and the Grenadines, US Virgin Islands, Samoa, Eritrea, Andorra, Antigua and Barbuda, Bahrain, Barbados, Cook Islands, Dominica, Grenada, Kiribati, Maldives, Malta, Marshall Islands, Micronesia, Monaco, Montenegro, Nauru, Niue, Palau, Saint Kitts and Nevis, Saint Lucia, San Marino, Seychelles, Singapore, South Sudan, Tonga, Tuvalu. The population of these countries accounts for 0.3% of total population in WHO regions. In order to quantify the monetized value of economic costs, we also used the world average GDP per capita (2020 USD) to multiply the equivalent of GDP per capita. The world average GDP per capita in 2020 USD were derived from GDP per capita in current USD and inflation rate.

Data

1. Heat-related mortality data is provided by Indicator 1.1.6 in section 1.
2. Population in each country are taken from World Bank.[287]
3. GDP per capita in OECD members are taken from OECD statistics.[288]
4. VSL in OECD are taken from OECD report on Mortality Risk Valuation in Environment, Health and Transport Policies.[289]
5. Years of remaining life are obtained from WHO.[290]
6. World average GDP per capita in current USD and inflation rate each year are taken from World Bank.[291,292]

Additional Analysis

The charts below show the indicator results, according to HDI, WHO and LC groupings.

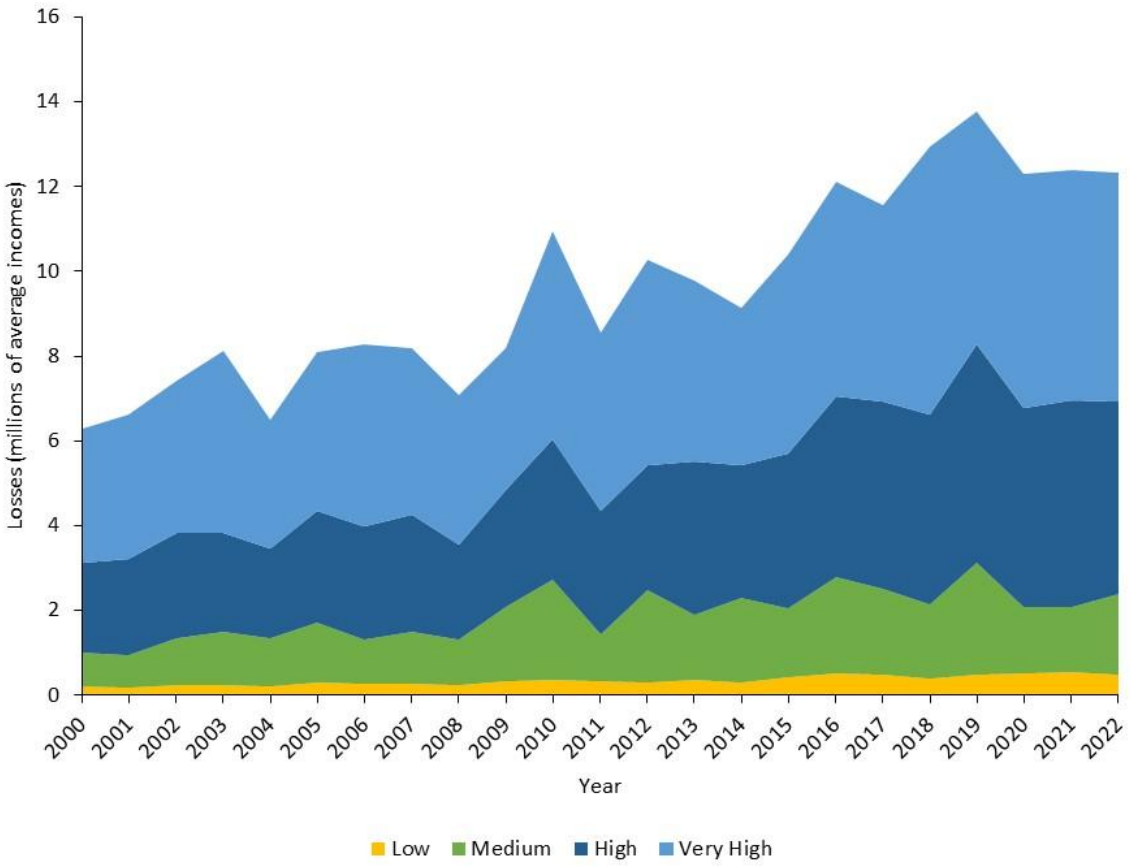

Figure : Monetized value of heat-related mortality losses (in numbers of average incomes expressed as GDP per capita) by HDI bands from 2000 to 2022

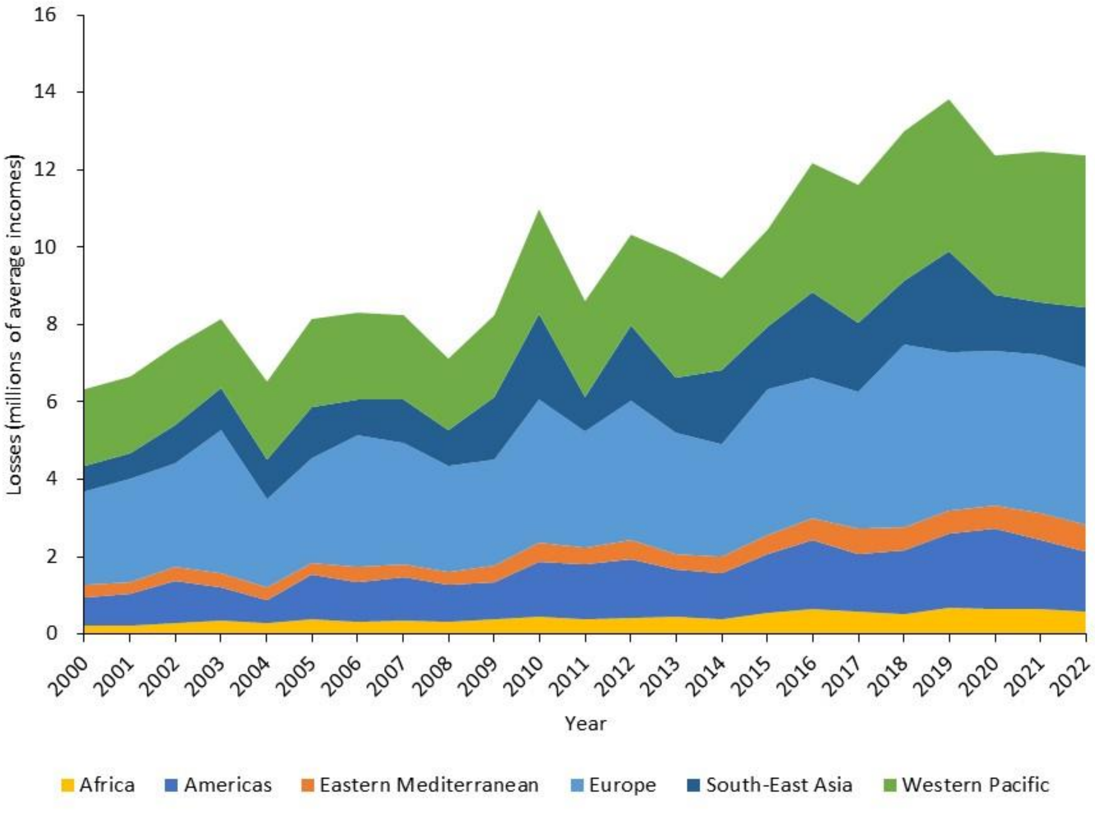

Figure 104: Monetized value of heat-related mortality losses (in numbers of average incomes expressed as GDP per capita) by WHO regions from 2000 to 2022

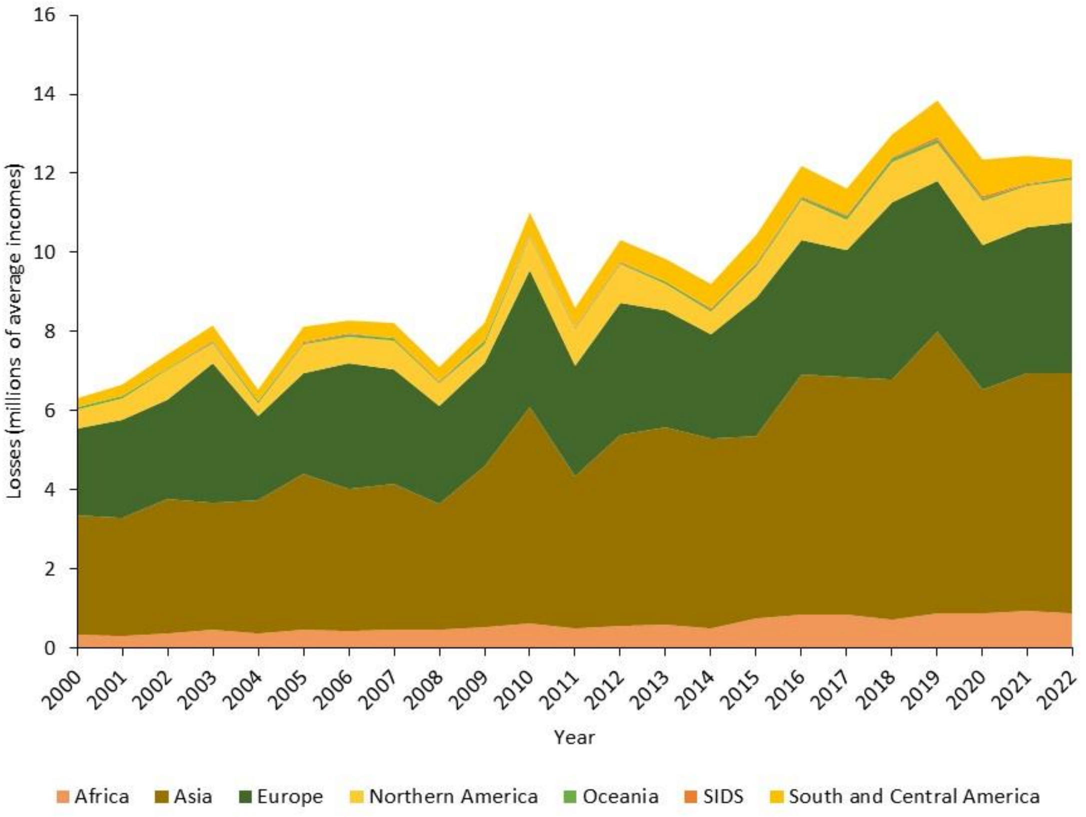

Figure 105. Monetized value of heat-related mortality losses (in numbers of average incomes expressed as GDP per capita) by Lancet Countdown groupings from 2000 to 2022

Caveats

This indicator could be improved through three perspectives. First, the inequality embedded within the economic costs of heat-related mortalities across different social groups are ignored in this indicator due to lack of data. In the future, with heat-related mortality data with more detailed social group aggregations, this indicator might explore further inequalities. Second, this indicator only considered the direct costs from mortalities of elder population, ignoring the potential costs that might derived from it. In the future we will consider estimating the direct and indirect costs from heat-related mortalities of elders. Third, currently we calculate VSLY for different ageing groups using the same remaining life at death (L). If age-specific YLL is provided in the future, this indicator can apply age-specific VSLY according to different remaining life at death (L) for different ageing groups.

Indicator 4.1.3: Potential Loss of Earnings from Heat-Related Labour Capacity Reduction

Indicator authors

Dr Daniel Scamman

Methods

Indicator 1.1.4 provides data on heat-related labour capacity loss, in terms of work hours lost (WHLs), at country scale across four sectors (services, manufacturing, construction and agriculture) for the years 1990-2022 inclusive. In order to calculate potential loss of earnings from this labour capacity loss, it was necessary to compile a dataset of average earnings per hour for each of these countries, sectors and years.

Earnings and income statistics were compiled from the ILOSTAT databases held by the ILO, within the category 'Statistics on Wages'. ILOSTAT includes a number of indicators which are of potential relevance to deriving the average annual hourly wages for the required countries and years. There are variations in the coverage of these indicators, with none having an entirely comprehensive coverage of the countries, sectors and years required for this indicator. Multiple ILOSTAT indicators were therefore used to fill as many gaps as possible. The three main indicator sets used were:

- Mean nominal monthly earnings of employees by sex and economic activity: annual
- Mean nominal monthly earnings of employees by sex and occupation: annual
- Mean nominal hourly earnings of employees by sex and occupation: annual

Within each of these indicator sets, the employment activities most accurately reflecting the four required sectors were selected. In some cases, more than one such activity was available, due to different reporting conventions (for example, the set of activities under ISCO-08 being an update from ISCO-88). Full descriptions of ILO indicators and classifications are available on the ILOSTAT website.[293]

Each indicator and activity was available in US dollar and local currency units. US dollar units were preferred, however in each indicator and activity case, the number of returns in local currency units was slightly higher, so these were selected as well in case more data points could be covered by doing so.

The following tables set out for each of the four employment sectors, the ILOSTAT indicators and activity definitions that were selected in order to supply as much of used the required data as possible. In each table the indicator, activity and currency combinations are arranged in the order of preference with which they were used.

	Indicator	Activity	Currency
1	Mean nominal monthly earnings of employees by sex and economic activity: annual	Aggregate: Trade, transportation, accommodation and food, and business and administrative services	US Dollars
2		Aggregate: Trade, transportation, accommodation and food, and business and administrative services	Local currency
3	Mean nominal monthly earnings of employees by sex and occupation: Annual	ISCO-08: 5. Service and sales workers	US Dollars
4		ISCO-08: 5. Service and sales workers	Local currency
5		ISCO-88: 5. Service workers and shop and market sales workers	US Dollars
6		ISCO-88: 5. Service workers and shop and market sales workers	Local currency
7	Mean nominal hourly earnings of employees by sex and occupation: Annual	ISCO-08: 5. Service and sales workers	US Dollars
8		ISCO-08: 5. Service and sales workers	Local currency
9		ISCO-88: 5. Service workers and shop and market sales workers	US Dollars
10		ISCO-88: 5. Service workers and shop and market sales workers	Local currency
11	Mean nominal monthly earnings of employees by sex and economic activity: annual	ISIC Rev.4: N. Administrative and support service activities	US Dollars
12		ISIC Rev.4: N. Administrative and support service activities	Local currency
13		ISIC Rev. 3.1: K. Real estate, renting and business activities	US Dollars
14		ISIC Rev. 3.1: K. Real estate, renting and business activities	Local currency
15		ISIC Rev.2: 8. Financing, insurance, real estate and business services	US Dollars
16		ISIC Rev.2: 8. Financing, insurance, real estate and business services	Local currency

Table 69: Indicators, activity classes and currencies selected to gather data from the ILOSTAT databases on earnings in the services sector, in order of preference

	Indicator	Activity	Currency
1	Mean nominal monthly earnings of employees by sex and economic activity: annual	Aggregate: Manufacturing	US Dollars
2		Aggregate: Manufacturing	Local currency
3		ISIC Rev.4: C. Manufacturing	US Dollars
4		ISIC Rev.4: C. Manufacturing	Local currency
5		ISIC Rev. 3.1: D. Manufacturing	US Dollars
6		ISIC Rev. 3.1: D. Manufacturing	Local currency
7		ISIC Rev.2: 3. Manufacturing	US Dollars
8		ISIC Rev.2: 3. Manufacturing	Local currency
9	Mean nominal monthly earnings of employees by sex and occupation: Annual	ISCO-08: 8. Plant and machine operators, and assemblers	US Dollars
10		ISCO-08: 8. Plant and machine operators, and assemblers	Local currency
11		ISCO-88: 8. Plant and machine operators and assemblers	US Dollars
12		ISCO-88: 8. Plant and machine operators and assemblers	Local currency
13	Mean nominal hourly earnings of employees by sex and occupation: Annual	ISCO-08: 8. Plant and machine operators, and assemblers	US Dollars
14		ISCO-08: 8. Plant and machine operators, and assemblers	Local currency
15		ISCO-88: 8. Plant and machine operators and assemblers	US Dollars
16		ISCO-88: 8. Plant and machine operators and assemblers	Local currency

Table 70: Indicators, activity classes and currencies selected to gather data from the ILOSTAT databases on earnings in the manufacturing sector, in order of preference

	Indicator	Activity	Currency
1	Mean nominal monthly earnings of employees by sex and economic activity: annual	Aggregate: Agriculture	US Dollars
2		Aggregate: Agriculture	Local currency
3		ISIC Rev.4: A. Agriculture; forestry and fishing	US Dollars
4		ISIC Rev.4: A. Agriculture; forestry and fishing	Local currency
5		ISIC Rev.3.1: A. Agriculture, hunting and forestry	US Dollars
6		ISIC Rev.3.1: A. Agriculture, hunting and forestry	Local currency
7		ISIC Rev.2: 1. Agriculture, hunting, forestry and fishing	US Dollars
8		ISIC Rev.2: 1. Agriculture, hunting, forestry and fishing	Local currency
9	Mean nominal monthly earnings of employees by sex and occupation: Annual	ISCO-08: 6. Skilled agricultural, forestry and fishery workers	US Dollars
10		ISCO-08: 6. Skilled agricultural, forestry and fishery workers	Local currency
11		ISCO-88: 6. Skilled agricultural and fishery workers	US Dollars
12		ISCO-88: 6. Skilled agricultural and fishery workers	Local currency
13	Mean nominal hourly earnings of employees by sex and occupation: Annual	ISCO-08: 6. Skilled agricultural, forestry and fishery workers	US Dollars
14		ISCO-08: 6. Skilled agricultural, forestry and fishery workers	Local currency
15		ISCO-88: 6. Skilled agricultural and fishery workers	US Dollars
16		ISCO-88: 6. Skilled agricultural and fishery workers	Local currency

Table 71: Indicators, activity classes and currencies selected to gather data from the ILOSTAT databases on earnings in the agricultural sector, in order of preference

	Indicator	Activity	Currency
1	Mean nominal monthly earnings of employees by sex and economic activity: annual	Aggregate: Construction	US Dollars
2		Aggregate: Construction	Local currency
3		ISIC Rev.4: F. Construction	US Dollars
4		ISIC Rev.4: F. Construction	Local currency
5		ISIC Rev. 3.1: F. Construction	US Dollars
6		ISIC Rev. 3.1: F. Construction	Local currency
7		ISIC Rev.2: 5. Construction	US Dollars
8		ISIC Rev.2: 5. Construction	Local currency
9	Mean nominal monthly earnings of employees by sex and occupation: Annual	ISCO-08: 9. Elementary occupations	US Dollars
10		ISCO-08: 9. Elementary occupations	Local currency
11		ISCO-88: 9. Elementary occupations	US Dollars
12		ISCO-88: 9. Elementary occupations	Local currency
13	Mean nominal hourly earnings of employees by sex and occupation: Annual	ISCO-08: 9. Elementary occupations	US Dollars
14		ISCO-08: 9. Elementary occupations	Local currency
15		ISCO-88: 9. Elementary occupations	US Dollars
16		ISCO-88: 9. Elementary occupations	Local currency

Table 72: Indicators, activity classes and currencies selected to gather data from the ILOSTAT databases on earnings in the manufacturing sector, in order of preference

A spreadsheet tool was developed to select the relevant data points for all available countries in order of indicator preference – if there was no data point for a given country, year and sector in the first priority indicator, the data point was sought in the next indicator, and so on until a data point was found, or all indicators had been tried.

Monthly earnings data were converted to hourly values using a standard assumption of 40 hours per week and 4.33 weeks per month, i.e., 173.2 hours per month.[294]

Data in nominal local currency units were converted to nominal US dollars at market exchange rates using IMF International Financial Statistics. Nominal US dollar values were converted to real 2022 US dollar values using the US dollar consumer price index from the IMF World Economic Outlook database.

Even after searching 16 variations of ILO indicator, activity and reporting currency for each sector, there were still considerable gaps, with around two thirds of required data points unfilled. In addition, there was a small number of clearly erroneous data points – e.g., with hourly earnings rates orders of magnitude too high, possibly caused by incorrect recording of the currency in which the data were reported, or by episodes of rapid inflation and currency devaluation, with which the recorded market exchange rates were not keeping track.

In order to fill the gaps with no data, as well as to correct data points that were clearly erroneous, a gap filling process was undertaken, using other data points to stand in for the missing or erroneous data. This process was undertaken after all of the data had been corrected to real 2022 US dollar values, so that all of the data were already expressed in constant values. Wherever possible, gaps were filled using data from a different year but from the same sector and country. Where data were available in years before and after the gaps in the same sector and country, linear interpolation was used to fill the gaps. If no future year was available, data were filled using the nearest past year. Likewise, if no previous year was available, the nearest future year was used. If there were no data points available at all for a certain sector or country, the data were taken from the same sector of a different country that was as comparable as possible to the country with missing data. Identification of a reasonably comparable country was achieved primarily by selecting one as close as possible on the current HDI scale, within the same or similar region and current World Bank Income Group (WBIG), of a similar population, and with a reasonable number of datapoints. It should be recognised, however, that countries that currently have similar HDI or WBIG bandings could have had quite different bandings in the past.

A small number of countries with no wage data or HDI value could not be included in the analysis as no suitable substitute could be found.

This process resulted in estimates of hourly earnings for the four sectors for 188 countries for the years 1990-2022 inclusive (the period for which WHL data are available from indicator 1.1.4), for 188 countries. These hourly earnings data were multiplied by the corresponding values for work hours lost (WHL) in each country, sector, and

year, to provide a quantification of potential earnings lost. The WHLs used assumed that work in the agricultural and construction sectors took place in the sun.

These total lost earnings were expressed as a percentage of the country's GDP in each relevant year. GDP data in nominal US dollars at market exchange rates were downloaded from the IMF World Economic Outlook database, and rendered in constant 2022 US dollars using the GDP deflator index from the same source. Gaps in this GDP data for some countries and years imposed a small further restriction on the coverage of this indicator, and not all of the same countries are available for all years. The maximum country-coverage of the indicator is 183 countries, during the years 2002–2022 inclusive. Results are presented as the average value for countries in each of the HDI bandings, WHO regions and Lancet Countdown (LC) regions.

Data

1. Data on working hours lost from indicator 1.1.4
2. Data on earnings by country and sector from ILOSTAT[295]
3. Exchange rate data from IMF International Financial Statistics[296]
4. US Dollar CPI and GDP deflator index from the IMF World Economic Outlook database[284]
5. Country GDP data from the IMF World Economic Outlook database[284]
6. World Bank Income Groups[297]

Caveats

There are several important caveats associated with the analysis:

- The ILOSTAT data do not cover all of the countries, years and sectors required, hence some gap filling was required, as described above. Whilst reasonable care has taken to identify appropriate estimates, gaps filled in the data are subject to uncertainties
- Whilst reasonable efforts have been made to correct for clearly erroneous data points, the analysis is dependent on the reliability of the ILOSTAT data, which could be subject to uncertainties in reporting, collection and processing
- The use of different combinations of ILOSTAT indicators and activity classes, rather than one single indicator and one activity class per sector, was necessary to increase data coverage as much as possible. Nonetheless this entails risks of inconsistencies, for example associated with different classifications and reporting methods
- The conversion of monthly data to hourly was carried out on the basis of a standard assumption of 4.33 weeks per month, and 40 hours per week. Real monthly working times will vary from these assumptions to a greater or lesser extent in different countries

All of these issues mean that caution should be exercised when examining results for any particular country. In addition, it must be emphasised that the results produced are the *potential* loss of earnings, rather than actual. The indicator is not based on evidence as to whether time off work was in fact taken. Further, if time was taken off work, the bearer of the costs of the lost labour could have varied between countries and sectors. In some instances, workers may have been able to claim sick pay, in which case the losses would have been borne by the employer through paying for non-productive time. In other instances, no arrangements for sick pay may have been in place, in which case it would have been the worker who would have borne the cost through a direct loss of earnings due to the inability to work.

Finally, the indicator by definition is an estimate of potential loss of earnings from formal paid sectors. In many countries informal and unpaid labour is also significant. Such activities could include domestic work and small-scale agriculture. The impacts on productivity and health of extreme heat on workers involved in so-called informal sectors, would be in addition to the monetised estimates quantified by this indicator.

Additional analysis

The following graphs present the results according to HDI, WHO, and LC groupings and also changes in results 1990-2022 according to HDI group.

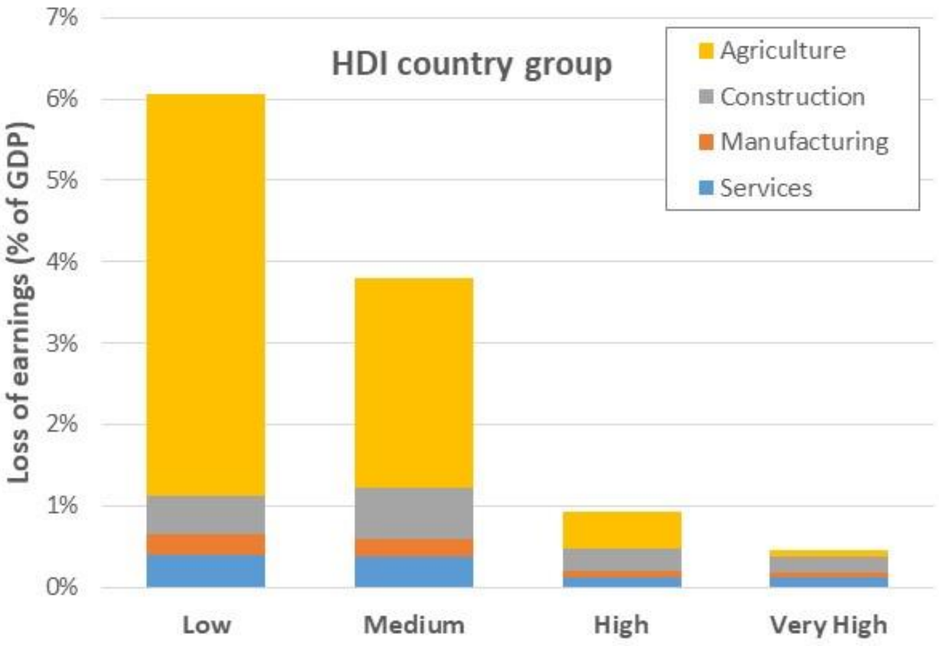

Figure 106: Average potential loss of earnings in 2022 per HDI country group as a result of potential labour loss due to heat exposure. Losses are presented as share of GDP and sector of employment.

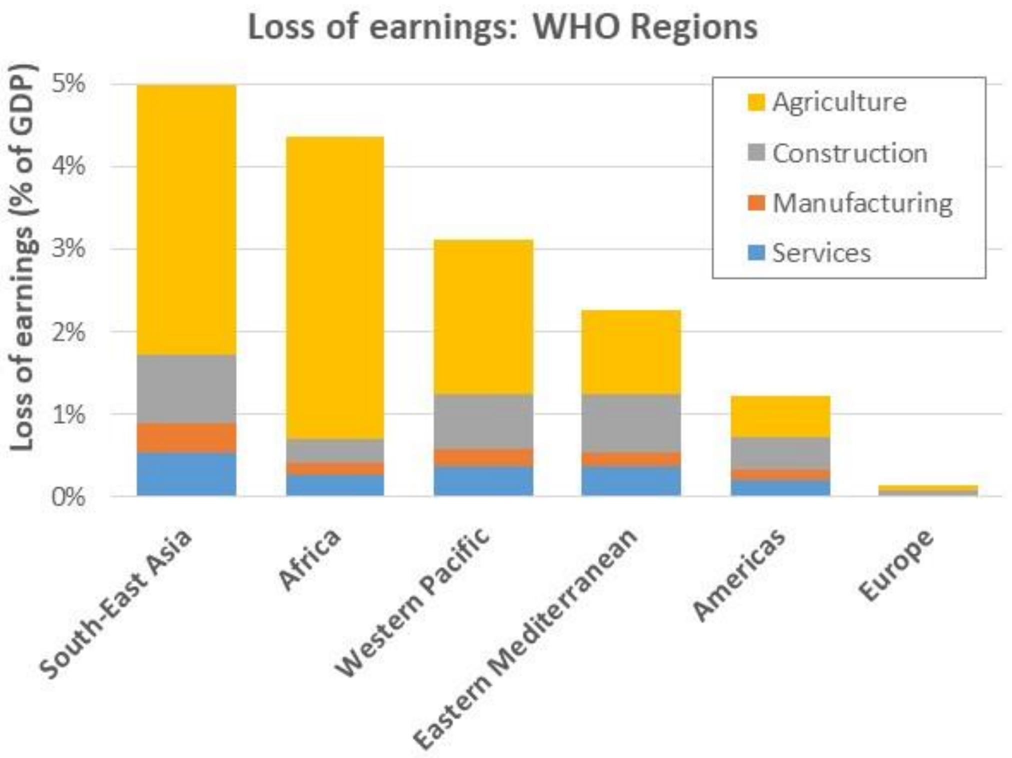

Figure 107: Average potential loss of earnings from heat-related labour capacity reduction in 2022 as a share of GDP according to WHO region and sector of employment

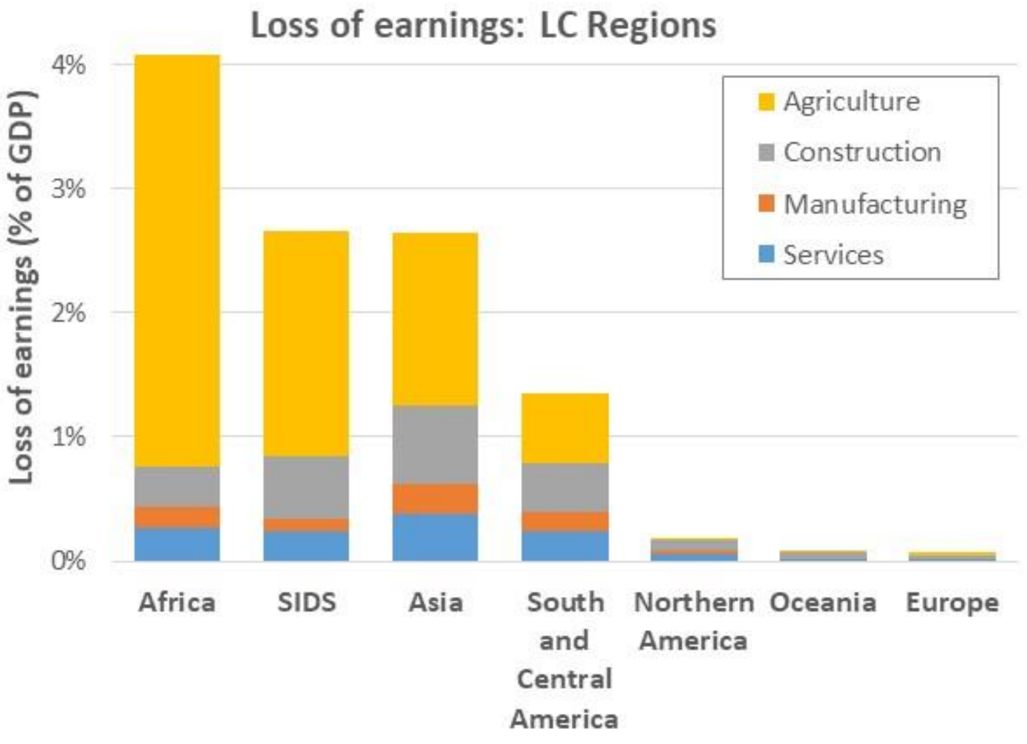

Figure 108: Average potential loss of earnings from heat-related labour capacity reduction in 2022 as a share of GDP according to LC grouping and sector of employment

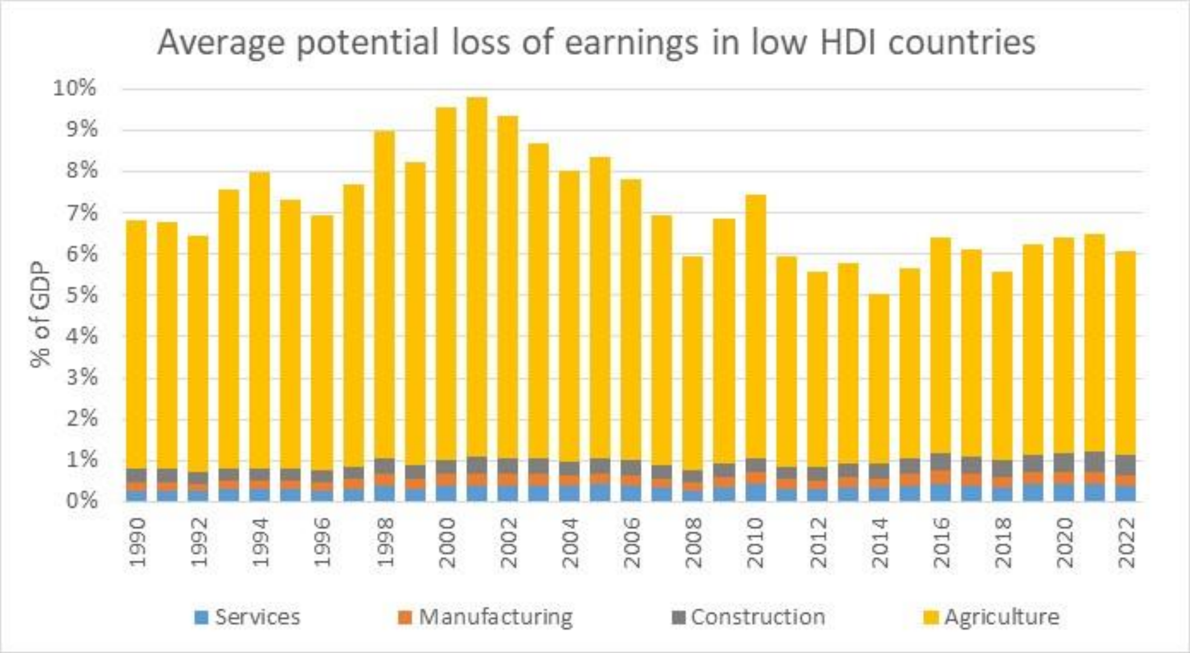

Figure 109: Average potential loss of earnings from heat-related labour capacity reduction as a share of GDP for low HDI countries, by sector of employment

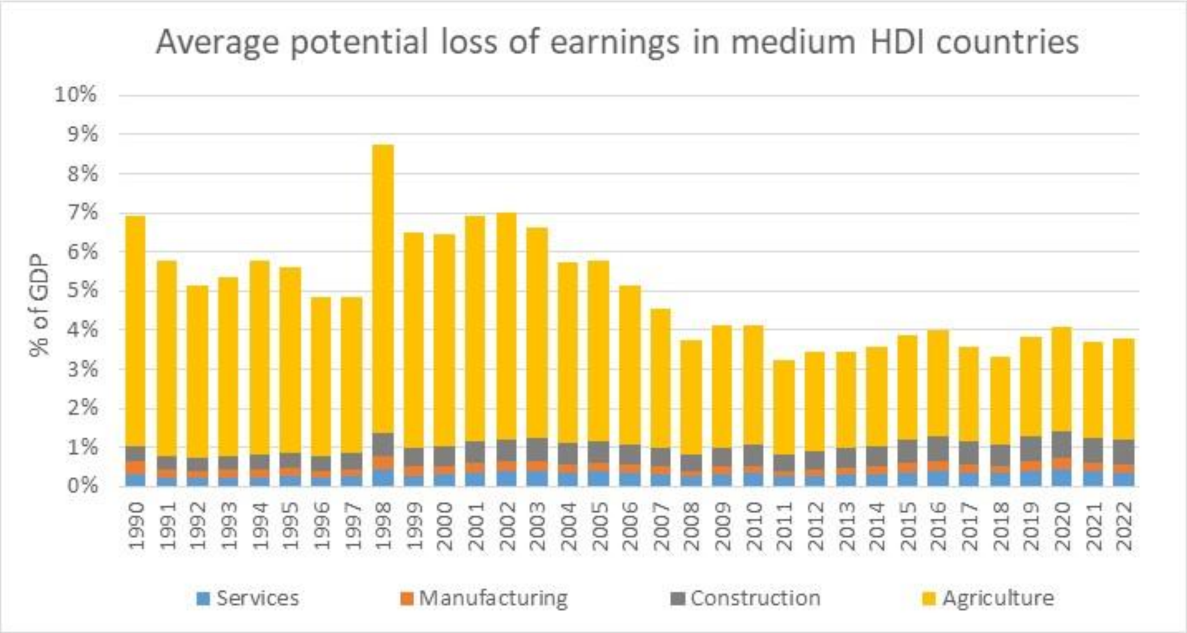

Figure 110. Average potential loss of earnings from heat-related labour capacity reduction as a share of GDP in medium HDI countries, by sector of employment.

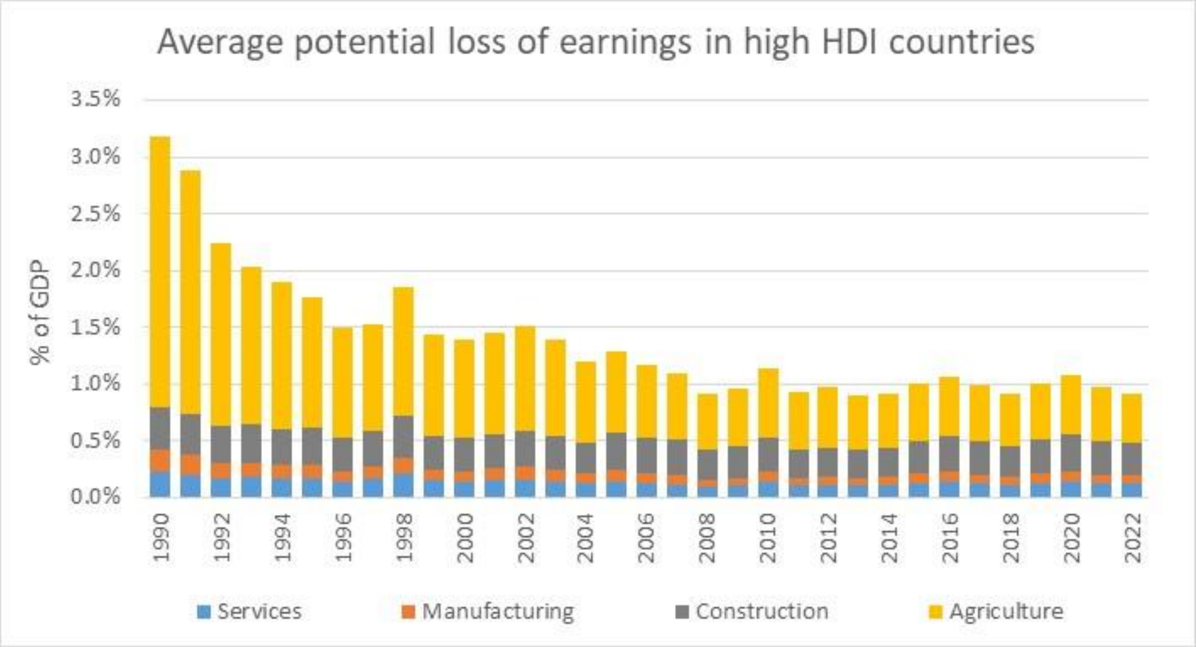

Figure 111. Average potential loss of earnings from heat-related labour capacity reduction as a share of GDP in high HDI countries, by sector of employment.

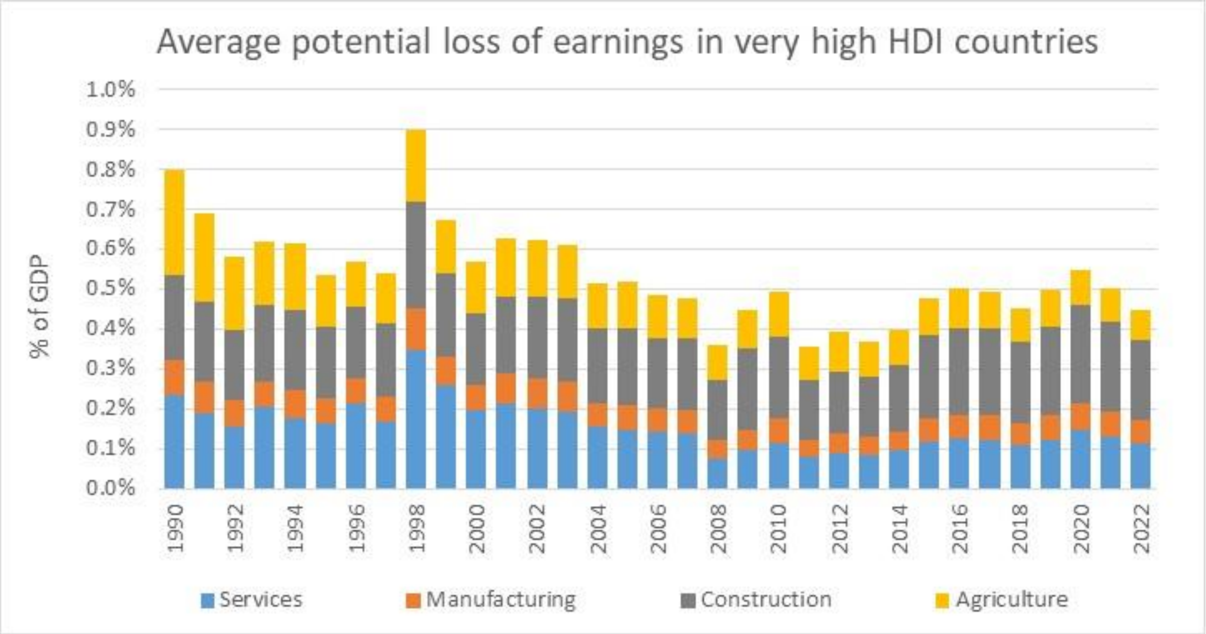

Figure 112. Average potential loss of earnings from heat-related labour capacity reduction as a share of GDP for very high HDI countries, by sector of employment.

Indicator 4.1.4: Costs of the Health Impacts of Air Pollution

Indicator authors

Dr Gregor Kiesewetter, Dr Daniel Scamman

Methods

Indicator 3.3 provides data on deaths attributable to both natural and anthropogenic ambient air pollution. Years of life lost (YLLs) were calculated from the age-specific attributable deaths by summing over the remaining life expectancy at the age of death for each attributable death. To determine YLLs attributable to anthropogenic causes only, the total YLLs are reduced to the country- and year-specific proportion of total deaths attributable to anthropogenic sources only in indicator 3.3. The YLLs calculated this way are a conservative estimate since the remaining life expectancy in real world conditions are used, rather than hypothetical conditions with no pollution, which would be larger. YLLs were calculated for 140 individual countries for the years 2005, 2010, 2015 and 2020. Each country was then classified according to its HDI category, WHO region and LC grouping.[298,299] For the WHO region and LC grouping calculations, the YLL data for 43 additional countries in three 'rest of world' regions from the GAINS model were also included (see Table 73), though population and GDP data had to be excluded for some of these countries where data was unavailable as shown. From 2023, three countries in a fourth GAINS region (Former Soviet Union) were included as separate countries (Tajikistan, Turkmenistan and Uzbekistan). Also the US Virgin Islands have been included in the Americas WHO region, and for the LC groupings French Guiana is included in the SIDS region rather than South and Central America, and Belize in South and Central America rather than SIDS. It was not possible to include countries in the three GAINS regions in the HDI classification, due the heterogeneity of classifications of the countries that constitute each region.

The YLLs for each category and region were then summed. To determine the economic value of the YLLs for each category and region relative to per capita average annual income in each, the results were multiplied by the fixed ratio of the Value of a Statistical Life Year (VSLY) to GDP per capita derived by indicator 4.1.2. To calculate the economic value of the YLLs relative to total GDP for each year, the results of this first calculation were multiplied by average GDP per capita (calculated from the sum of GDP for each category and region, inflated to $2022 from current prices, divided by the sum of the population for each category and region), and then divided by the sum of GDP in $2022 for the category or region in question.

GDP and GDP inflator data were taken from the International Monetary Fund (IMF), and population data were taken from the United Nations (UN). The data and methods used to calculate the fixed ratio between VSLY and GDP per capita are described in indicator 4.1.2.

GAINS Region	WHO Region	LC Group	Country
Caribbean (CARB)	Americas	SIDS	Anguilla, Antigua and Barbuda, Aruba, Bahamas, Barbados, British Virgin Islands*, Caribbean Netherlands*, Cayman Islands*, Cuba, Curaçao*, Dominica, Dominican Republic, French Guiana*, Grenada, Guadeloupe*, Guyana, Haiti, Jamaica, Martinique*, Puerto Rico, Saint Lucia, Saint Vincent and the Grenadines, Suriname, Trinidad and Tobago, United States Virgin Islands*
Central America (CEAM)	Americas	South and Central America	Belize, Costa Rica, Guatemala, Honduras, Nicaragua, Panama, El Salvador
Middle East (MIDE)	Eastern Mediterranean	Asia	United Arab Emirates, Bahrain, Iraq, Jordan, Kuwait, Lebanon, Oman, Occupied Palestinian Territory, Qatar, Syrian Arab Republic, Yemen

*Population and GDP excluded from the calculations due to lack of data of either one or other data point.

Table 73: Countries in GAINS model 'rest of word' region group included in the calculation of costs of air pollution for WHO and LC groupings

Data

1. IMF World Economic Outlook (October 2022)[284]
2. UN World Population Prospects[300]

Caveats

See indicator 3.3, for caveats related to the calculation of reduced life expectancy.

Caveats regarding the calculation of VSLY are discussed under indicator 4.1.2. Countries not listed in the tables above have been excluded from the analysis, due to the lack of individual characterisation in the model used to calculate YLLs. Democratic People's Republic of Korea is excluded from the analysis due to the lack of reliable GDP data. Somalia is excluded from the HDI analysis, as it is not classified. Data for 2022 differs to those presented in the 2022 report due to updated data.

Additional analysis

The monetized losses from premature mortality due to air pollution according to HDI group is shown in the main report. Figure 113 and Figure 114 show the monetized losses according to WHO region and LC group.

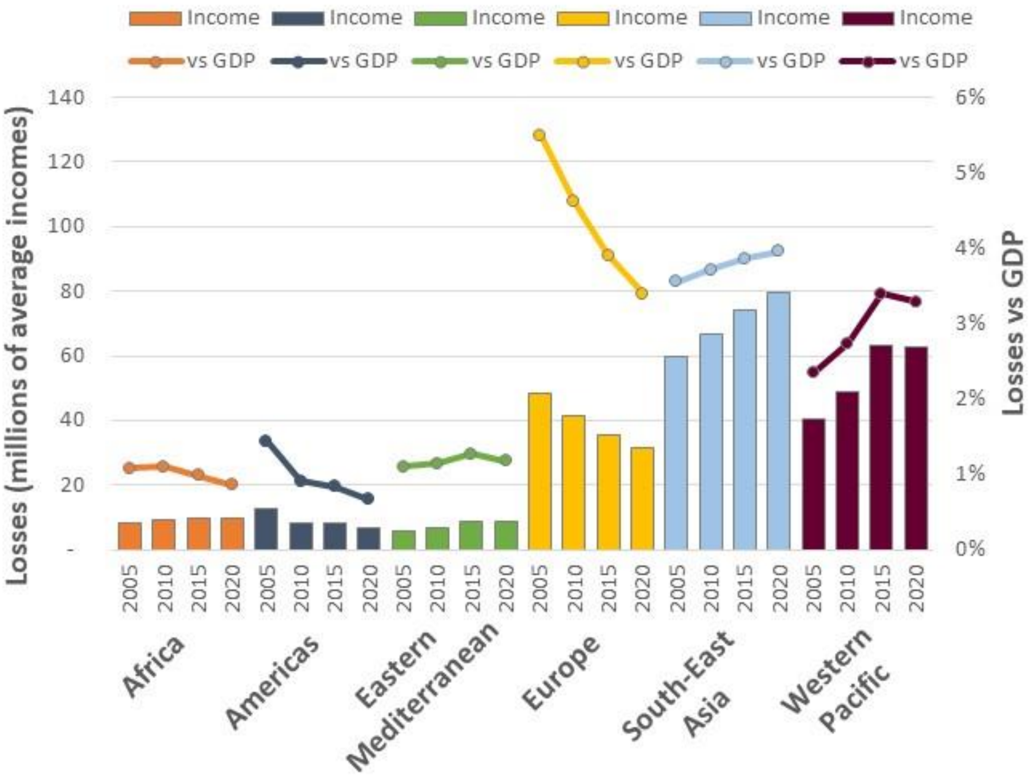

Figure 113. Monetized losses from premature mortality due to air pollution according to WHO region. Columns represent losses as numbers of average incomes, lines as losses vs GDP.

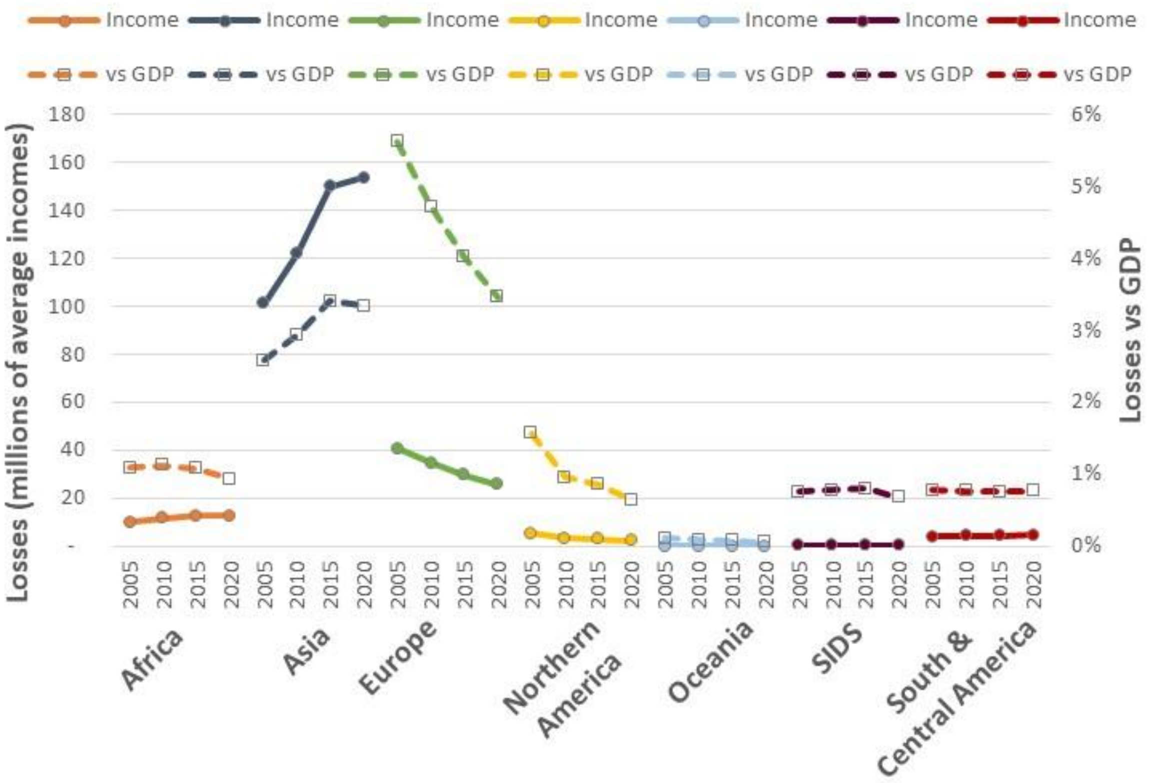

Figure 114. Monetized losses from premature mortality due to air pollution according to LC group. Solid lines represent losses as numbers of average incomes, dashed lines as losses vs GDP.

4.2: The Economics of the Transition to Net Zero-Carbon Economies

Indicator 4.2.1: Zero-carbon Energy Investment

Indicator authors

Dr Daniel Scamman

Methods

The data for this indicator is sourced from the annual IEA World Energy Investment publication.[301] Key categories of investment are defined as follows

Clean energy – investment in renewable power, energy efficiency, electricity networks, electric vehicles, battery storage, nuclear, low-emission fuels (modern liquid and gaseous bioenergy, low-emission hydrogen and low-emission hydrogen-based fuels), CCUS (carbon capture utilisation and storage), and other end-use (renewables for end use and electrification in the buildings, transport and industrial sectors).

Fossil fuels – investment in coal, oil and gas electricity generation capacity and fuel supply without CCS.

Power sector – investment in coal, oil, gas, nuclear and renewable electricity generation capacity, and electricity networks and battery storage. Renewables includes pumped-hydro storage.

Other supply – investment in coal, natural gas, oil and renewable energy supply for non-electricity purposes. This includes upstream mining, drilling and pipeline infrastructure. Renewable energy includes modern liquid and gaseous bioenergy, low-carbon hydrogen, as well as hydrogen-based fuels that do not emit any CO_2 from fossil fuels directly when used and also emit very little when being produced.

Energy efficiency – An energy efficiency investment is defined as the incremental spending on new energy-efficient equipment or the full cost of refurbishments that reduce energy use.

For most sectors, 'investment' is defined as ongoing capital spending on assets. For some sectors, such as power generation, this investment is spread out evenly from the year in which a new plant or upgrade of an existing one begins its construction to the year in which it becomes operational. For other sources, such as upstream oil and gas and liquefied natural gas (LNG) projects, investment reflects the capital spending incurred over time as production from a new source ramps up or to maintain output from an existing asset. This definition and differs from the definition previously employed by the IEA before 2019, in which investment was defined as overnight capital expenditure.

Data

1. IEA World Energy Investment 2023.[301]

Caveats

Other areas of expenditure, including operation and maintenance, research and development, financing costs, mergers and acquisitions or public markets transactions, are not included. Investment estimates are derived from IEA data for energy demand, supply and trade, and estimates of unit capacity costs, For more information, see IEA World Energy Investment 2023.

Additional analysis

The variation in clean energy and fossil fuel investment for 2015-2022 is shown below.

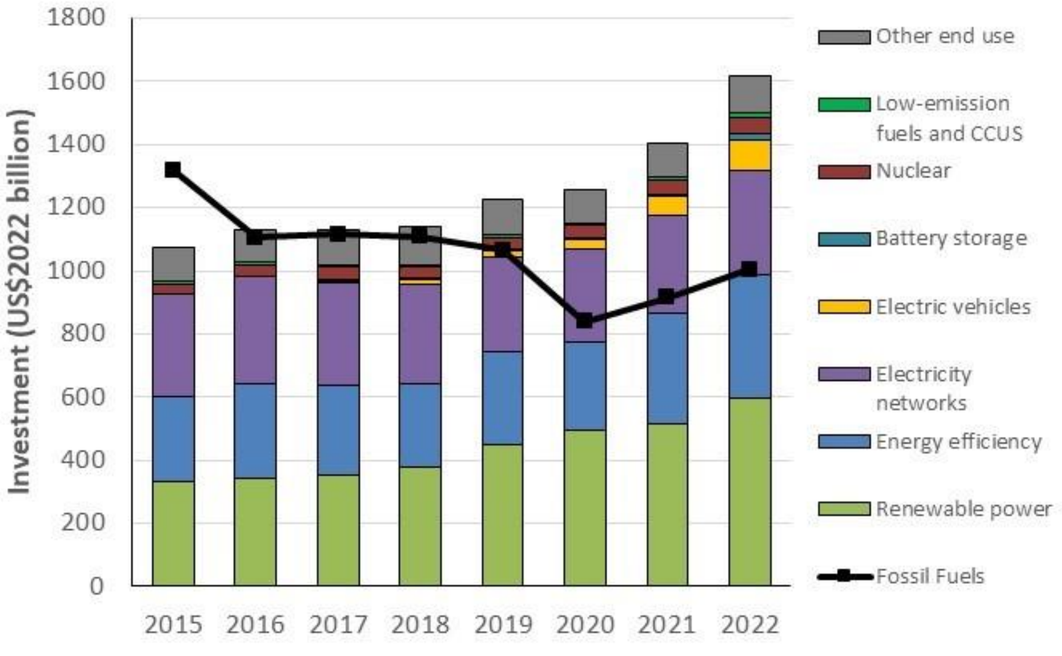

Figure 115: Global energy investment in clean energy (columns) and fossil fuels (solid line).

Indicator 4.2.2: Employment in Renewables and Fossil Fuel Industries

Indicator authors

Dr Daniel Scamman

Methods

The data for this indicator is sourced from IRENA (renewables) and IBISWorld (fossil fuel extraction). Renewable industries included are:

- Hydropower;
- Solar heating/cooling;
- Solar photovoltaic;
- Wind energy;
- Bioenergy;
- Other technologies.

Bioenergy includes liquid biofuels, soil biomass and biogas. ‘Other technologies’ includes geothermal energy, ground-based heat pumps, concentrated solar power, municipal and industrial waste, and ocean energy. Fossil fuel extraction values include direct employment, whereas renewable energy jobs include direct and indirect employment (e.g., equipment manufacturing), except for large hydropower (direct employment only).

Due to an improvement in data collection and estimation methodology, employment values reported for fossil fuel extraction are in some years substantially higher than those reported in the 2018 *Lancet* Countdown report. Similarly, an improvement to the methodology for estimating hydropower has altered historic values for Hydropower (previously called ‘large’ hydropower), and Other Technologies (which previously included small hydropower). From 2018, ‘Other Technologies’ now also includes employment related to ground-based heat pumps.

Data

1. Data for employment in renewables from IRENA[302]
2. Data for employment in fossil fuel extraction from IBISWorld: oil and gas exploration and production; and coal mining[303,304]

Caveats

Fossil fuel extraction values include direct employment, whereas renewable energy jobs include direct and indirect employment (e.g., equipment manufacturing), with the exception of hydropower.

Future form of the indicator

Additional analysis

	Million Jobs									
	**2012**	**2013**	**2014**	**2015**	**2016**	**2017**	**2018**	**2019**	**2020**	**2021**
Solar Photovoltaic	1.36	2.27	2.49	2.77	3.09	3.37	3.68	3.75	3.98	4.29
Bioenergy	2.4	2.5	2.99	2.88	2.74	3.05	3.18	3.58	3.52	3.44
Hydropower	1.66	2.21	2.04	2.16	2.06	1.99	2.05	1.96	2.18	2.37
Wind Energy	0.75	0.83	1.03	1.08	1.16	1.15	1.16	1.17	1.25	1.37
Solar Heating/Cooling	0.89	0.5	0.76	0.94	0.83	0.81	0.8	0.82	0.82	0.77
Other Technologies	0.22	.023	0.19	0.2	0.24	0.16	0.18	0.18	0.27	0.45
**Renewables Total**	**7.28**	**8.54**	**9.5**	**10.03**	**10.12**	**10.53**	**11.05**	**11.46**	**12.02**	**12.69**
**Fossil Fuel Extraction**	**11.79**	**12.16**	**12.38**	**12.35**	**12.36**	**12.05**	**12.07**	**11.82**	**11.22**	**13.42**

Table 74: Employment in renewable energy and fossil fuel extraction industries.

Indicator 4.2.3: Funds Divested from Fossil Fuels

Indicator authors

Dr Daniel Scamman

Methods

The data for this indicator is collected and provided by stand.earth. Prior to this report, they represented the total assets (or assets under management, AUM) for institutions that have publicly committed to divest (for which data is available), with non-US$ values converted using the market exchange rate when the commitment was made, and thus did not directly represent the actual sums divested from fossil fuel companies. For the data used in this report, AUM data has been updated to 2022 levels. A company is committed to 'divestment' if it falls into any of the following five categories:

- 'Fossil Free' - An institution or corporation that does not have any investments (direct ownership, shares, commingled mutual funds containing shares, corporate bonds) in fossil fuel companies (coal, oil, natural gas) and committed to avoid any fossil fuel investments in the future

- 'Full' - An institution or corporation that made a binding commitment to divest (direct ownership, shares, commingled mutual funds containing shares, corporate bonds) from any fossil fuel company (coal, oil, natural gas).

- 'Partial' - An institution or corporation that made a binding commitment to divest across asset classes from some fossil fuel companies (coal, oil, natural gas), or to divest from all fossil fuel companies (coal, oil, natural gas), but only in specific asset classes (e.g. direct investments, domestic equity).

- 'Coal and Tar Sands' - An institution or corporation that made a binding commitment to divest (direct ownership, shares, commingled mutual funds containing shares, corporate bonds) from any coal and tar sands companies.

- 'Coal only' - An institution or corporation that made a binding commitment to divest (direct ownership, shares, commingled mutual funds containing shares, corporate bonds) from any coal companies.

Nine organisations that were originally recorded as non-healthcare institutions have been considered as such for the purpose of this indicator (London School of Hygiene and Tropical Medicine, The Royal College of General Practitioners, New Zealand Nurses Organisation, HESTA, HCF, Berliner Ärzteversorgung, Doctors for the Environment Australia, the Royal College of Emergency Medicine, and the Society for the Psychological Study of Social Issues). Divestment commitments by the American Medical Association, which divested in 2018, was not included in the data provided by 350.org, and was added separately.

Data

1. Stand.earth and Global Fossil Fuel Divestment Commitments Database[305]

Caveats

Data on the number of institutions that have divested, and the value of their assets is dependent on institutions reporting this information to Stand.earth.

Additional analysis

The cumulative value of divestment (both global total and for healthcare institutions) is presented below (Table 75). Organisations that have divested but for which no date of divestment (a total of $2.56 billion) are recorded in a separate column, with the total assumed to begin in 2008 in the absence of more detailed information.

US$ million (2022 data)

	Global	Global (including data with no divestment date)	Healthcare Institutions
2008	$16	$2,562,392	$-
2009	$17	$2,562,392	$-
2010	$17	$2,562,392	$-
2011	$84	$2,562,460	$-
2012	$3,773	$2,566,148	$-
2013	$9,337	$2,571,712	$-
2014	$441,744	$3,004,120	$37,809
2015	$2,569,415	$5,131,791	$38,103
2016	$3,559,438	$6,121,814	$41,010
2017	$5,571,959	$8,134,335	$53,191
2018	$8,875,191	$11,437,567	$54,107
2019	$12,426,839	$14,989,215	$54,120
2020	$28,468,322	$31,030,697	$54,187
2021	$37,890,410	$40,452,785	$54,187
2022	$37,950,405	$40,511,479	$54,187

Table 75: Cumulative fossil fuel divestment.

Due to confidentiality issues, the full dataset is not available for publication. However, interested readers may visit the www.divestmentdatabase.org for further information.

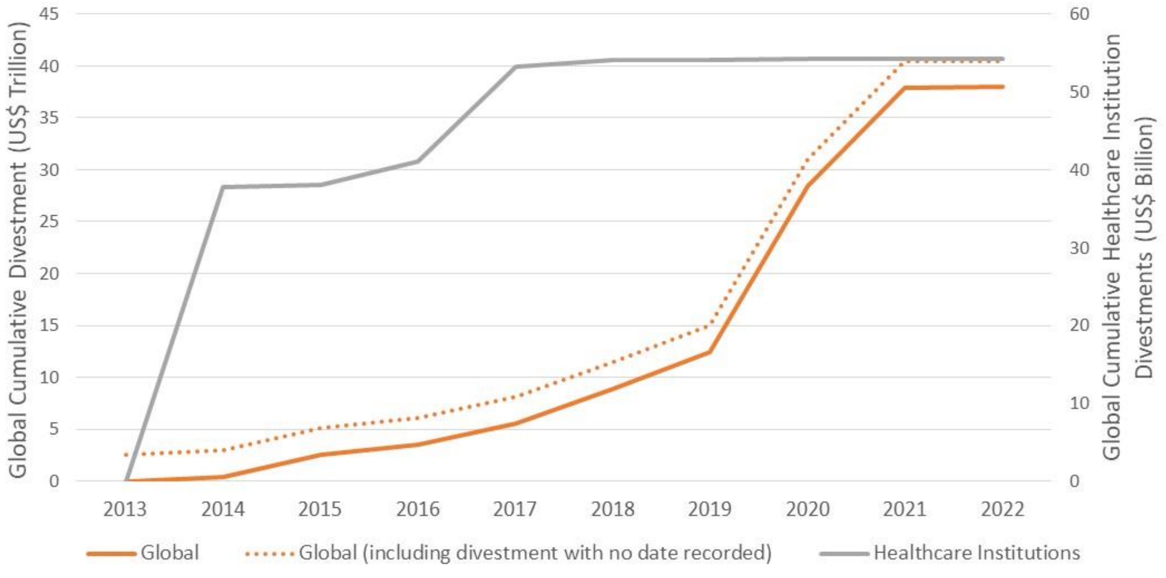

Figure 116. Cumulative divestment – Global total and in healthcare institutions

Indicator 4.2.4: Net Value of Fossil Fuel Subsidies and Carbon Prices

Indicator authors

Dr Daniel Scamman

Methods

Fossil Fuel Subsidies

Data for fossil fuel subsidies were taken from two sources. The IEA provides data on fossil fuel consumption subsidies for 42 countries, calculated using its 'price gap' approach – the difference between the end-user prices paid for fossil fuels in the country, and reference prices that account for the full cost of supply.[306,307] However, the countries provided in this list are mainly non-OECD. The OECD itself provides estimates of fossil fuel subsidies within the 38 OECD countries, plus Argentina, Armenia, Azerbaijan, Belarus, Brazil, China, Georgia, India, Indonesia, Moldova, Russia, South Africa and Ukraine—a total of 51 countries.[308] OECD's estimates are derived from a bottom-up inventory of subsidy mechanisms within each country, and include production and consumption support, infrastructure investments, incentives and R&D. It divides the type of support into three broad categories: Consumer Support Estimate (CSE), Producer Support Estimate (PSE) and General Services Support Estimate (GSE).

Combining the IEA and OECD datasets allows a coverage of 81 countries, after accounting for overlaps and the omission of countries not covered by the *Lancet* Countdown. The OECD describes an approach for combining these two datasets, and reconciling different estimates for the countries covered by both.[309] This involves selecting line items in the OECD inventory that correspond to the price-gap definition of subsidies that is the basis of the IEA data – i.e. measures that bring about reduced consumer prices: 'conceptually, an OECD estimate derived from individual measures that capture transfers to consumers from producers and taxpayers should match the IEA price-gap estimates' (p.22-3).[309]

The description of this approach suggests that in the few cases of countries whose subsidies have been calculated by both OECD and IEA, the OECD estimate would be expected to be the larger of the two.[309] However, analysis of overlapping countries suggests that it is in fact more often the IEA estimate that is larger. This analysis is described in more detail in the appendix of the 2020 *Lancet* Countdown report. The conclusion drawn from this is that attempting to separate some line items from the OECD estimates that seem more directed at consumers is not a reliable way of reconciling the two estimates – on the contrary, in several cases it makes the gap between the two larger by making the OECD estimate smaller. Consequently, in considering countries that overlap between the two datasets as part of preparing this indicator, a comparison was made simply between the total OECD estimate and the total IEA estimate.

Following a simple rule of thumb proposed by OECD, in order to decide which estimate to use in overlapping cases, the source that produces the larger cumulative total for a given country over the years being considered, was the one chosen as the source for that country for this indicator.[309]

Carbon prices and revenues

Information on carbon prices and carbon pricing revenues was sourced from the World Bank Carbon Pricing Dashboard.[310] Revenues from each recorded instrument were allocated to the nation state within which the instrument operated. Shares of the EU ETS revenues were allocated to each of the participants in the EU ETS – that is the 28 members of the EU (which included the UK for the years considered in this analysis), plus Iceland and Norway. The UK was allocated a share of ETS until the end of 2020 as, although the UK left the EU on 31 January 2020, the UK remained subject to EU rules until 31 December 2020 with the UK's own ETS replacing its participation in the EU ETS from 1 January 2021. Liechtenstein is also an EU ETS member but could not be included in this analysis due to lack of CO_2 emissions data. The allocation of EU ETS revenues was made to participating states on the basis of their share of the emissions of all EU ETS states, calculated using IEA CO_2 emissions data.[311] This was considered an acceptable simplification given that for the period 2013–2020, 88% of allowances were allocated for auction to participating states in proportion to their emissions.[312] Also carbon pricing revenue data was included for El Salvador in 2010, but none has been reported since.

Countries were included in the analysis if data were available for CO_2 emissions, and either fossil fuel subsidies or carbon pricing instruments. This yielded a list of 87 countries in 2020 accounting for 93% of global CO_2 emissions.[311]

Net carbon price and revenue calculations

In reality at present, both carbon prices and fossil subsidies are typically applied to individual sectors or fuels, and do not cover the entire economy. Within different particular jurisdictions the sectors covered by subsidies and carbon prices are often not identical. As such the only way of producing a consistent indicator across multiple countries was to average out both subsidies and prices across the CO_2 emissions of the whole economy, resulting in net average economy-wide carbon prices and revenues. Each country's total fossil fuel subsidies were subtracted from its total carbon price revenues to produce a net carbon revenue. These figures were divided by the relevant total country CO_2 emissions for each year, using data from the IEA,[311] resulting in the net carbon price. The net carbon revenue was expressed as a proportion of national expenditure on health, using current annual (i.e. not including capital) health expenditure data from the WHO's Global Health Expenditure Database.[313]

Currency standardisation

All money values are expressed in real 2022 US$. Both the OECD Inventory and the IEA fossil fuel subsidy database provide data in real 2021 US$. These units were corrected to real 2022 values, using the GDP deflator for the US dollar, from the IMF. The World Bank carbon pricing revenue data and the WHO health expenditure data are given in nominal US dollars, so again the US GDP deflator from IMF was applied to correct to real 2022 values.

Data

1. Fossil fuel subsidies data from the IEA and OECD[306,308]
2. Carbon pricing data from the World Bank Carbon Pricing Dashboard[310]
3. CO_2 emissions from fuel combustion from IEA[311]
4. Health expenditure data from WHO[313]
5. US Dollar GDP deflator index from the IMF World Economic Outlook database[284]

Caveats

The principal caveat is that the indicator is strongly dependent on the reliability of the main datasets from the IEA, OECD and World Bank. It is possible that data on individual countries may not be fully comprehensive due to reporting errors, lack of information or other issues, as indeed is acknowledged by OECD.[309] The indicator should be considered as a way of illustrating global trends, and caution should be exercised in attempting to draw out specific conclusions relating to individual countries covered by the indicator.

The nature of indicators that draw on multiple datasets is that the most recent year on which they can report is defined by the most recent year that is common to all datasets used. In this case that year was 2020, which was due to this being the most recent complete year for both CO_2 emissions from fuel combustion and health expenditure.

The economy-wide net carbon price was derived by dividing fossil fuel subsidies and carbon pricing revenues by total CO_2 emissions. This fits well with the subsidies, as these are for fossil fuels, the principal source of CO_2. However, some of the carbon pricing instruments from which the revenue was assessed are not only for fossil fuel combustion but apply to other sectors and non-CO_2 gases. There is therefore a slight inconsistency between the sectoral coverage of the subsidies and the carbon pricing instruments.

Additional analysis

The relevant section in the main report shows net carbon prices, net carbon revenues, and net carbon revenues as a proportion of health spending, by HDI grouping, for the year 2020. The following graphs show results for the same three indicators for WHO and LC groupings. Due to the finite number of countries included in the indicator, some classifications have fewer inclusions than others, as shown in the tables below for 2020 data. Note the number of countries included has grown since 2010 as additional countries have begun reporting data (except for El Salvador, which reported data in 2010 but not since). Also shown are charts for the same three indicators with

all countries grouped together for the years 2010–2020 inclusive. Results for years 2016–2017 differ from those reported in the 2020 Countdown report due to an increased number of countries included in the analysis.

**HDI Band**	**Number**	**Countries**
Low	2	Nigeria, Pakistan
Medium	7	Angola, Bangladesh, Bolivarian Republic of Venezuela, Bolivia, Ghana, India, Iraq
High	21	Algeria, Armenia, Azerbaijan, Brazil, Bulgaria, China, Colombia, Ecuador, Egypt, Gabon, Indonesia, Islamic Republic of Iran, Libya, Mexico, Republic of Moldova, South Africa, Sri Lanka, Turkmenistan, Ukraine, Uzbekistan, Vietnam
Very High	57	Argentina, Australia, Austria, Bahrain, Belarus, Belgium, Brunei Darussalam, Canada, Chile, Costa Rica, Croatia, Cyprus, Czechia, Denmark, Estonia, Finland, France, Georgia, Germany, Greece, Hungary, Iceland, Ireland, Israel, Italy, Japan, Kazakhstan, Kuwait, Latvia, Liechtenstein, Lithuania, Luxembourg, Malaysia, Malta, Netherlands, New Zealand, Norway, Oman, Poland, Portugal, Qatar, Republic of Korea, Romania, Russian Federation, Saudi Arabia, Singapore, Slovakia, Slovenia, Spain, Sweden, Switzerland, Thailand, Trinidad and Tobago, Turkey, United Arab Emirates, United Kingdom, United States of America
**Total**	**87**	

Table 76: Inclusions in HDI Groupings for Indicator 4.2.4 for 2020 data

**WHO Region**	**Number**	**Countries**
Africa	6	Algeria, Angola, Gabon, Ghana, Nigeria, South Africa
Americas	12	Argentina, Bolivarian Republic of Venezuela, Bolivia, Brazil, Canada, Chile, Colombia, Costa Rica, Ecuador, Mexico, Trinidad and Tobago, United States of America
Eastern Mediterranean	11	Bahrain, Egypt, Iraq, Islamic Republic of Iran, Kuwait, Libya, Oman, Pakistan, Qatar, Saudi Arabia, United Arab Emirates
Europe	44	Armenia, Austria, Azerbaijan, Belarus, Belgium, Bulgaria, Croatia, Cyprus, Czechia, Denmark, Estonia, Finland, France, Georgia, Germany, Greece, Hungary, Iceland, Ireland, Israel, Italy, Kazakhstan, Latvia, Liechtenstein, Lithuania, Luxembourg, Malta, Netherlands, Norway, Poland, Portugal, Republic of Moldova, Romania, Russian Federation, Slovakia, Slovenia, Spain, Sweden, Switzerland, Turkey, Turkmenistan, Ukraine, United Kingdom, Uzbekistan
South-East Asia	5	Bangladesh, India, Indonesia, Sri Lanka, Thailand
Western Pacific	9	Australia, Brunei Darussalam, China, Japan, Malaysia, New Zealand, Republic of Korea, Singapore, Vietnam
**Total**	**87**	

Table 77: Inclusions in WHO Groupings for Indicator 4.2.4 for 2020 data

**LC Group**	**Number**	**Countries**
Africa	8	Algeria, Angola, Egypt, Gabon, Ghana, Libya, Nigeria, South Africa
Asia	29	Armenia, Azerbaijan, Bahrain, Bangladesh, Brunei Darussalam, China, Cyprus, Georgia, India, Indonesia, Iraq, Islamic Republic of Iran, Israel, Japan, Kazakhstan, Kuwait, Malaysia, Oman, Pakistan, Qatar, Republic of Korea, Saudi Arabia, Sri Lanka, Thailand, Turkey, Turkmenistan, United Arab Emirates, Uzbekistan, Vietnam
Europe	35	Austria, Belarus, Belgium, Bulgaria, Croatia, Czechia, Denmark, Estonia, Finland, France, Germany, Greece, Hungary, Iceland, Ireland, Italy, Latvia, Liechtenstein, Lithuania, Luxembourg, Malta, Netherlands, Norway, Poland, Portugal, Republic of Moldova, Romania, Russian Federation, Slovakia, Slovenia, Spain, Sweden, Switzerland, Ukraine, United Kingdom
Northern America	2	Canada, United States of America,
Oceania	2	Australia, New Zealand,
SIDS	2	Singapore, Trinidad and Tobago
South and Central America	9	Argentina, Bolivarian Republic of Venezuela, Bolivia, Brazil, Chile, Colombia, Costa Rica, Ecuador, Mexico
**Total**	**87**	

Table 78: Inclusions in LC Groupings for Indicator 4.2.4 for 2020 data

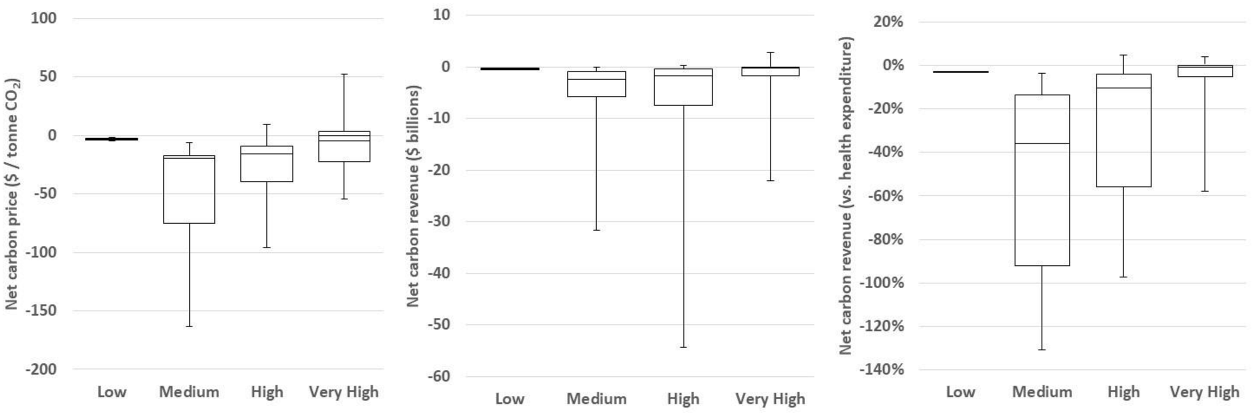

Figure 117: Net carbon prices *(left), net carbon revenues (centre), and net carbon revenue as a share of current national health expenditure (right), across 87 countries in 2020, arranged by HDI country group: low (n=2), medium (n=7), high (n=21) and very high (n=57). Boxes show the interquartile range (IQR), horizontal lines inside the boxes show the medians, and the brackets represent the full range from minimum to maximum.*

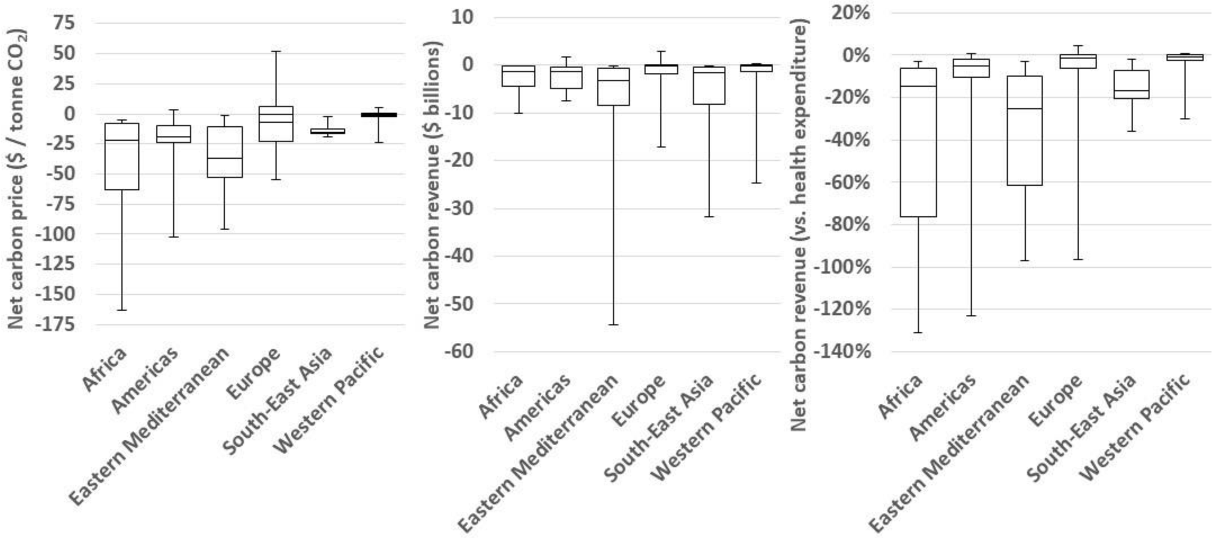

Figure 118: Net carbon prices, net carbon revenues, and net carbon revenue as a share of current national health expenditure, across 87 countries in 2020, grouped by WHO region. Boxes show the interquartile range (IQR), horizontal lines inside the boxes show the medians, and the brackets represent the full range from minimum to maximum.

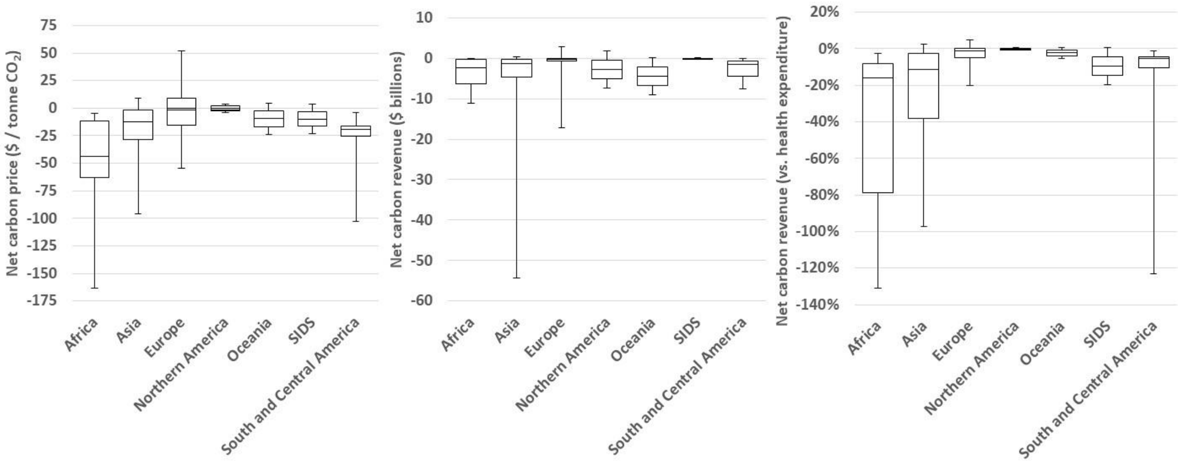

Figure 119: Net carbon prices, net carbon revenues, and net carbon revenue as a share of current national health expenditure, across 87 countries in 2020, grouped by LC grouping.

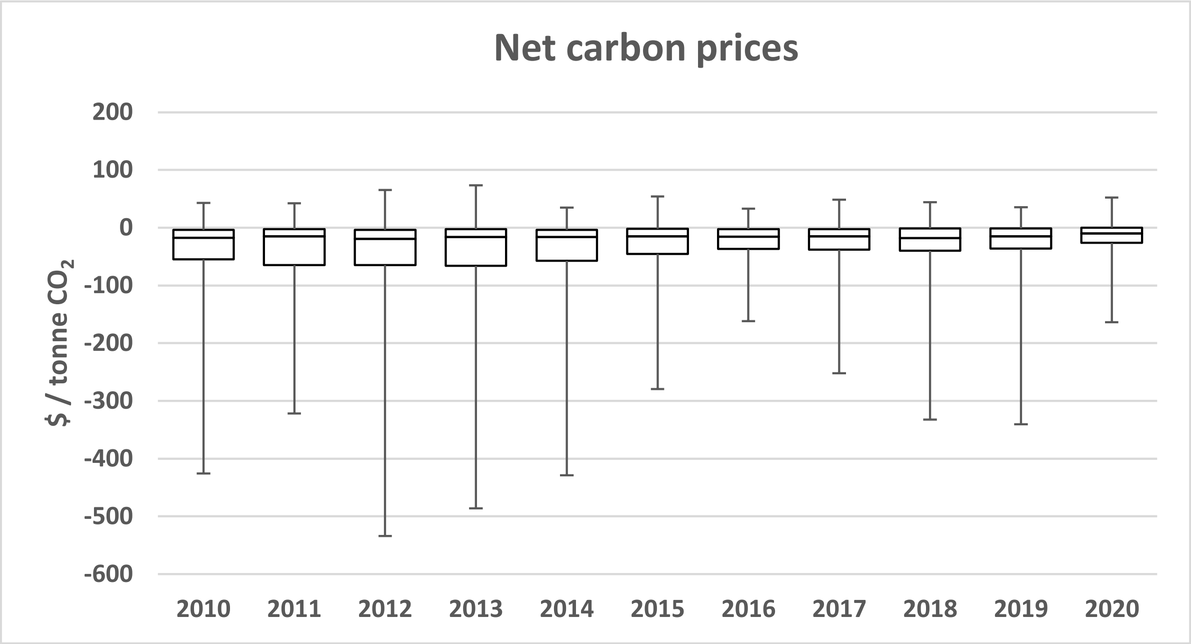

Figure 120. Net carbon prices for all countries included in the analysis, 2010–2020 inclusive.

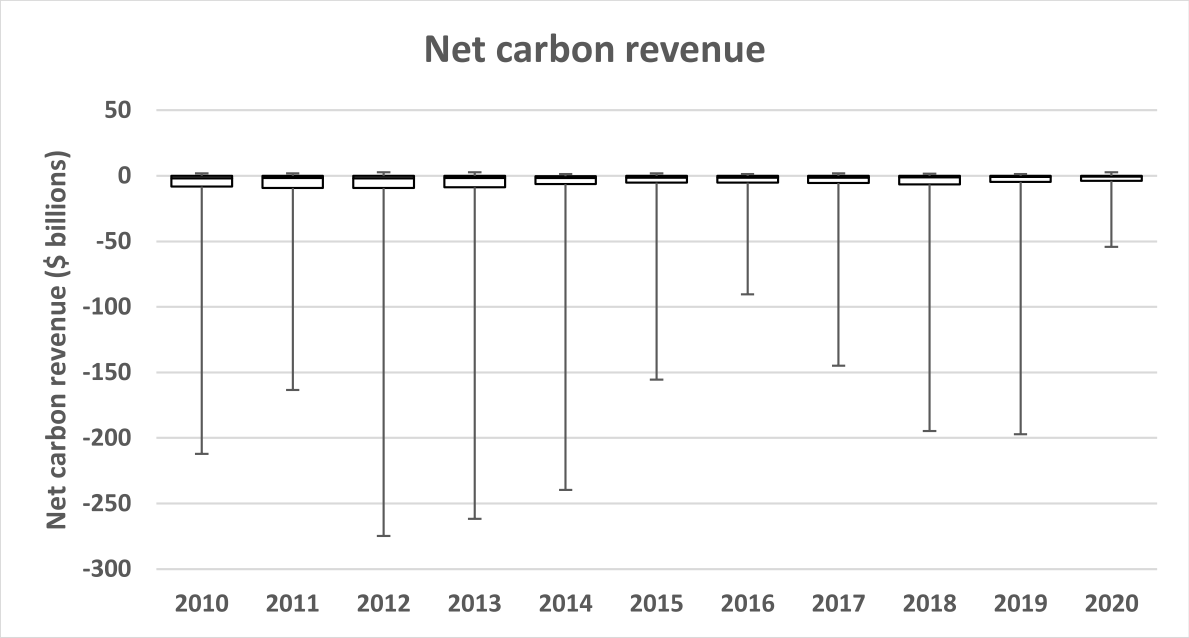

Figure 121. Net carbon revenue for all countries included in the analysis, 2010–2020 inclusive.

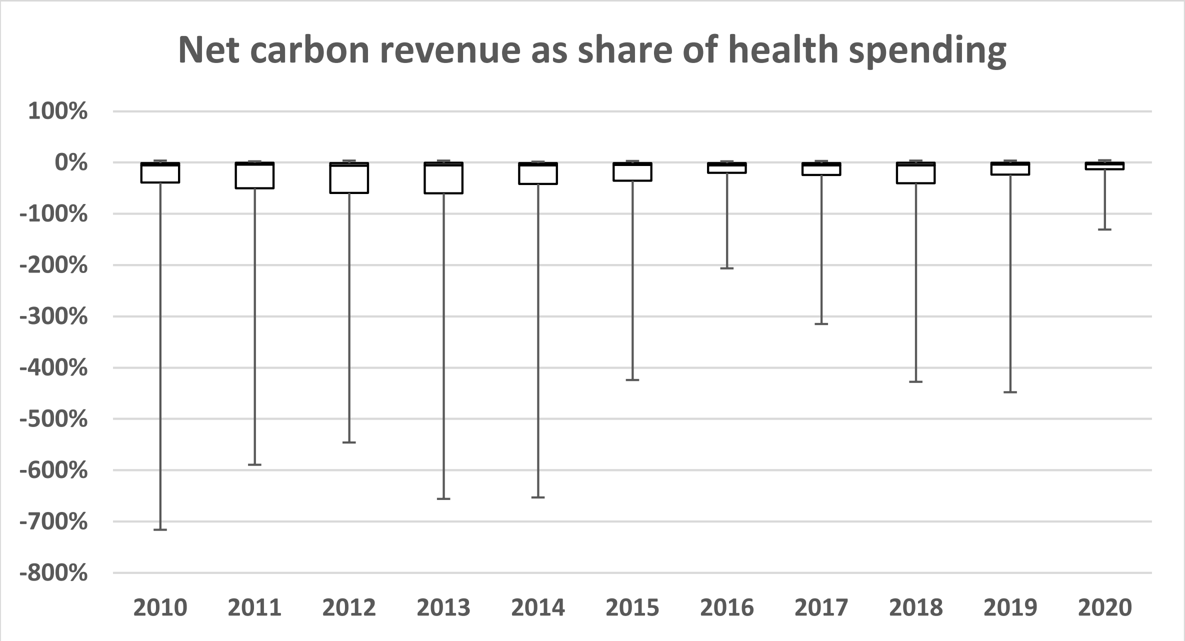

Figure 122. Net carbon revenue expressed as the equivalent share of current (i.e., not capital) annual health spending, for all countries included in the analysis, 2010–2020 inclusive.

Indicator 4.2.5: Production and Consumption-based Attribution of CO_2 and $PM_{2.5}$ Emissions

Indicator authors

Kehan He, Prof Zhifu Mi, Dr Fabian Wagner

Methods

Environmentally Extended Multi-Regional Input-Output Analysis

There are two approaches to measure emissions: production-based (sometimes referred to as territorial-based) accounting and consumption-based accounting. Production-based emissions occur within the geographical territory of a nation, while consumption-based emissions encompass the emissions from the nation's domestic final consumption, as well as those caused by the production of its imports. Since both CO_2 emissions via climate change, and air pollution directly, are detrimental to human health, understanding of the responsibilities of emissions across borders is crucial in the globalised world. This indicator estimates $PM_{2.5}$ and CO_2 emissions embodied in international trade, and then calculates national $PM_{2.5}$ and CO_2 emissions from the consumption perspective. Thus, the responsibility of these emissions and the associated environmental and human health consequences can be distributed for international environmental policy formulation.

Environmentally extended multi-regional input-output (EEMRIO) analysis is used in the calculation of consumption-based emissions.[314] The EEMRIO analysis can reflect production and consumption structures and interdependencies between economic sectors across regions. The relationships between final use and emissions are estimated via Leontief inverse matrix, which is expressed as follows in equation (1):

$$C = E \cdot L \cdot F = E \cdot (I - A)^{-1} \cdot F \qquad \textbf{(1)}$$

C is the total consumption-based emissions, CO_2 or $PM_{2.5}$ emissions in this case. It is mapped directly to emissions inventories. *E* is the row vector of the production-based emission intensity defined as the emissions per unit of output. *F* is the vector of final demand. and *L* is the Leontief inverse matrix calculated by $(I\text{-}A)^{-1}$, where *I* is the identity matrix, and *A* is the technical coefficient matrix describing the inter-sectoral and inter-regional flows per unit of output.

Consumption-based accounting encompasses emissions from domestic final consumption and those caused by the production of its imports, while production-based accounting measures emissions which take place within national territory. The above relationship can also be expressed as follows:

$$C_{CBA} = C_{PBA} - C_{exp} + C_{imp} \qquad \textbf{(2)}$$

where C_{CBA} is the consumption-based emissions, C_{imp} is the emissions embodied in imports, C_{PBA} is the production-based emissions, and C_{exp} is the emissions embodied in exports.

Emission Inventory Mapping with GAINS

To construct the production-based $PM_{2.5}$ emission inventory with the GAINS model, the workflow illustrated in Figure 123 is followed. First, an intermediary aggregation level to which emissions from the GAINS source categories are aggregated is defined.[1] In a second step these aggregated or grouped emissions are distributed among the relevant MRIO sectors according to a specific rule. This process is repeated until the emissions from all relevant GAINS source categories havWG4 e been mapped to the relevant MRIO sectors.

---

[1] In most cases GAINS sectors are used. However, in a few cases the relevant source categories are sector-fuel combinations in the GAINS system: for example, in the power plant sectors, coal-, oil-, gas-, and biomass-fired plants are distinguished [and combustion free generation] so as to be able to map directly to the corresponding MRIO sectors.

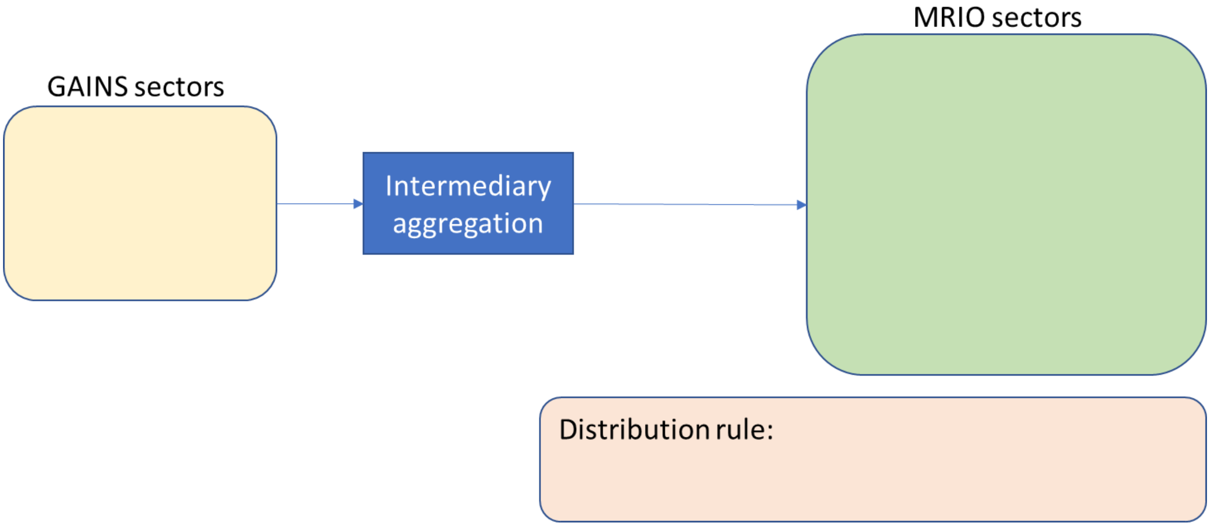

Figure 123. Generic approach for mapping the GAINS sectoral emissions to MRIO sectors.

In practice, the GAINS source categories are clustered into three groups, so that there are three rounds of mappings. These groupings correspond to energy-related emissions (except trucking, see below), process-related emissions, and trucking-related emissions. In a final step, for each MRIO sector the contributions from the three rounds of mappings are summed so that a total emission can be associated with each MRIO sector. In all calculations determining the relative energy share of an MRIO sector in the total energy, the use of electricity is ignored, since the emissions from electricity production are accounted for elsewhere.

On the GAINS side, trucking is related to the sectors TRA_RD_HDT and TRA_RD_LD4T and the fuel-related activities, such as diesel, gasoline, LPG etc, as well as km-related emissions such as abrasion, tyres and braking. On the MRIO side, diesel consumption from road transport by MRIO sector is used to determine the share of each sector in the total. In some countries significant amounts of diesel is also used by cars, a fact that is neglected here. Figure 124 illustrates the mapping process for trucking-related emissions between GAINS and the MRIO sectors.

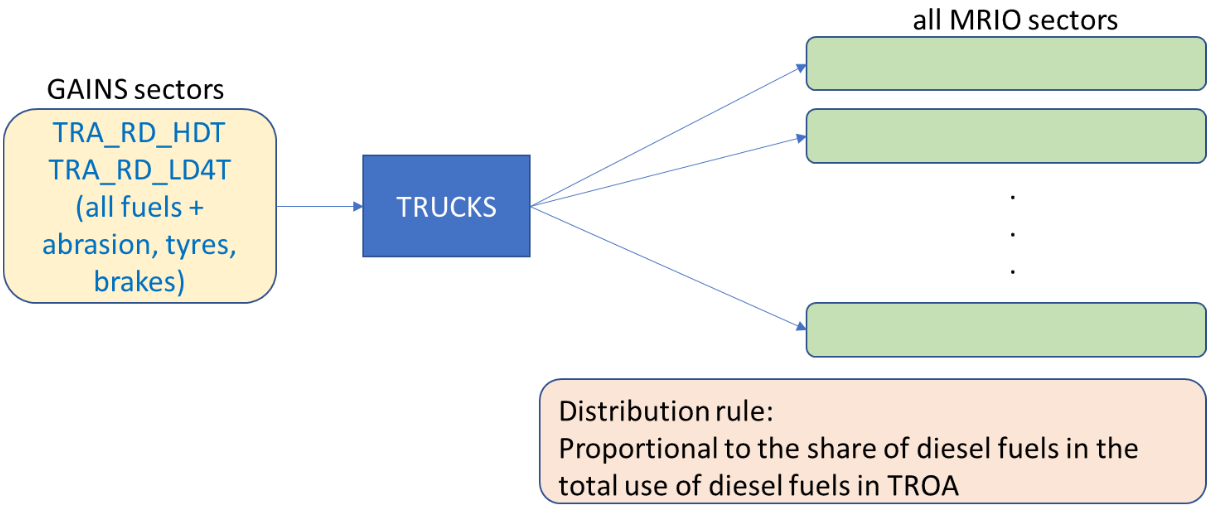

Figure 124 Mapping of trucking-related emissions

The trucking-related emissions in region r for MRIO sector m are thus calculated as:

$$\mathrm{Em}_r(m,t) = \mathrm{Em}_r(\mathrm{TRUCKS}) \cdot \mathrm{sh}_r(m,\mathrm{diesel}) \tag{3}$$

where

$$\mathrm{sh}_r(m,\mathrm{diesel}) = \frac{\mathrm{TROA}_r(m,\mathrm{diesel})}{\sum_{m'} \mathrm{TROA}_r(m',\mathrm{diesel})} \tag{4}$$

is the share of sector m in the road transport related diesel consumption in region r, and $\mathrm{Em}_r(\mathrm{TRUCKS})$ are the total trucking related emissions in region r as calculated by GAINS.

Once the trucking-related emissions and energy use has been separated out what is relevant for distributing the remaining energy (but not trucking-related emissions) is generally the total final energy consumption minus the diesel consumption in TROA. Thus, non-trucking related final energy consumption excluding electricity is referred to as the relevant final energy consumption in each MRIO sector that is used to determine the shares for distributing energy-related emissions into MRIO sectors.

In the mapping of energy-related emissions, intermediary clusters for energy-related emissions are defined as follow:

Table 79. Aggregated energy-related sectors, their description and coverage in terms of GAINS sectors as well as MRIO clusters.

Label	Description	GAINS sector coverage	MRIO clusters
ELE_COAL	Coal-fired power plants	All power plants combusting coal or solid biomass[2]	coal_electricity
ELE_OIL	Oil-fired power plants	All power plants combusting heavy fuel oil or diesel	oil_electricity
ELE_GAS	Gas-fired power plants	All power plants combusting natural gas	gas_electricity
AGR_MACH	Agricultural machinery	TRA_OT_AGR, DOM_OTH	cultivation + livestock_farming + items_dom_oth
IND_IS	Iron and steel industry	IN_OC_ISTE	manuf_is
IND_NFME	Non-ferrous metals	IN_OC_NFME	manuf_nfme
IND_NMMI	Non-metallic minerals	IN_OC_NMMI[3]	manuf_bricks + manuf_cem + manuf_nmmi
IND_CHEM	Chemical industries	IN_BO_CHEM, IN_OC_CHEM	manuf_chem + manuf_fert + manuf_chem_nec
IND_CON	Conversion industries, incl. refineries	IN_BO_CON, CON_COMB	ind_conversion

[2] It seems that no specific provision for biomass was made and thus it is included here.
[3] In GAINS energy-related emissions in NMMI (largely cement production) are all absorbed into process-related emissions, see below.

PPAPER	Pulp and paper	IN_BO_PAP, IN_OC_PAP	manuf_paper
OTH_IND	Other industries	All IN_XX_OTH sectors	other_industries
SERVICES	Services	DOM_COM subsectors, MSW	items_services
RAIL	Trains	TRA_OT_RAI	rail
Ships	Sea-going ships	TRA_OTS_X	ships
INW	Ships on inland waterways	TRA_OT_INW	inw
CONSTRUCTION	Construction machinery	TRA_OT_CNS, TRA_OT_LD2, TRA_OTH_LB	construction

The following approach is used for the mapping. Emissions from GAINS sectors (third column Table 79) are aggregated to an intermediary sector (first column) and then distributed among the MRIO sectors belonging to the clusters in the final column using their relative shares in the energy consumption. This is illustrated further for agricultural machinery and combustion devices in Figure 125.

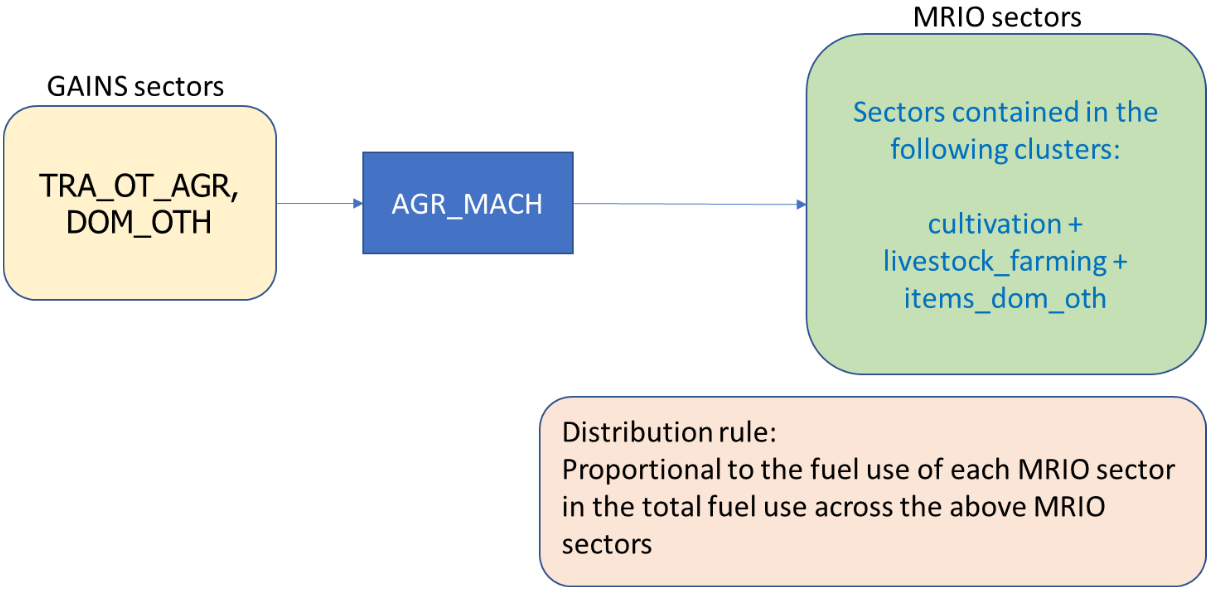

Figure 125 Approach for distributing emissions from agricultural machinery and devices (mobile and stationary) to MRIO sectors.

The energy related emissions in region *r* for MRIO sector *m* are thus:

$$\mathrm{Em}_r(m, e) = \sum_{label} \mathrm{Em}_r(\mathrm{label, e}) \cdot \mathrm{sh}_r(m, \mathrm{e, label}) \quad \textbf{(5)}$$

Where the sum is running over all labels given in Table 79 and the share

$$\mathrm{sh}_r(m, \mathrm{label, e}) = \frac{\mathrm{FE}_r^*(m, label)}{\sum_{m'} \mathrm{FE}_r^*(m', label)} \quad \textbf{(6)}$$

is the share of MRIO sector *m* in the final energy demand (minus trucking) in the total final energy demand (minus trucking) in cluster *label* in region *r*.

Process-related emissions are calculated in GAINS separately from energy-related emissions, i.e. there are separate source categories for these in GAINS. Again, intermediary aggregation sectors, this time relevant for the processes, are defined as follows:

Table 80 Aggregated process-related sectors, their description and coverage in terms of GAINS sectors as well as MRIO clusters.

Label	Description	GAINS sector coverage	MRIO clusters
AGR_PROC	Process emissions related to cultivation	FCON_X, AGR_ARABLE, WASTE_AGR, APPLIC_X, GRAZE_X, STH_NPK, STH_AGR	cultivation
PROC_CATTLE	Emissions related to cattle farming	AGR_COWS, AGR_BEEF	Cattle farming (single sector)
PROC_PIG	Emissions related to pig farming	AGR_PIGS	Pigs farming (single sector)
PROC_POULT	Emissions related to poultry farming	AGR_POULT	Poultry farming (single sector)
PROC_OTANI	Emissions related to farming of other animals	AGR_OTANI	Meat animals nec (single sector)
PROC_BRICK	Emissions related to brick production	PR_BRICK	manuf_bricks
PROC_CEM	Emissions related to cement production	PR_CEM, PR_LIME	manuf_cem
PROC_NMMI	Emissions related to other non-metallic minerals	PR_NMMI, PR_GLASS	manuf_nmmi
PROC_IS	Emissions related to iron and steel production	PR_EARC, PR_BAOX, PR_HEARTH, PR_CAST, PR_SINT, PR_SINT_F, PR_PIGI, PR_PIGI_F, PR_CAST_F	manuf_is
PROC_ALU	Emissions related to aluminium production	PR_ALPRIM, PR_ALSEC	manuf_alu
PROC_FERT	Emissions related to fertilizer production	PR_FERT, FERTPRO	manuf_fert
PROC_CHEM	Emissions related to other chemical processes	PR_SUAC, PR_CBLACK	manuf_chem
PROC_PULP	Emissions related to paper and pulp production	PR_PULP	manuf_paper
PROC_CONVERSION	Emissions related to energy conversion	PR_REF, PR_COKE, STH_COAL, PR_PELL	ind_conversion
PROC_COAL_MINE	Emissions related to coal mining	MINE_HC, MINE_BC, PR_BRIQ	mining_coal_io

PROC_OTHER_MINE	Emissions related to other mining	STH_FEORE, MINE_OTH, STH_OTH_IN	mining_other_io
PROC_SM_IND	Emissions related to other small industries	PR_SMIND_F, OTHER_VOC, PR_OT_NFME, PR_OTHER, OTHER_PM	other_industries
PROC_CONSTRUCT	Emissions related to construction activities	CONSTRUCT	construction

The process related emissions in region *r* for MRIO sector *m* are thus:

$$\mathrm{Em}_r(m,p) = \sum_{label} \mathrm{Em}_r(\mathrm{label},\mathrm{p}) \cdot \mathrm{sh}_r(m,\mathrm{e},\mathrm{label}) \quad \textbf{(7)}$$

Where the sum is running over all labels given in Table 80 and the share

$$\mathrm{sh}_r(m,\mathrm{label},\mathrm{e}) = \frac{\mathrm{FE}_r^*(m, label)}{\sum_{m'} \mathrm{FE}_r^*(m', label)} \quad \textbf{(8)}$$

is the share of MRIO sector *m* in the final energy demand (minus trucking) in the total final energy demand (minus trucking) in cluster *label* in region *r*. The main difference to the energy related emissions is that the clusters are different, and thus the shares for each sector within a cluster may be different.

As noted above it is a simplification to distribute the process emissions proportional to the energy use in the MRIO sector within its corresponding cluster, and refinements could be made on the basis of information which of the MRIO sectors within a cluster are mostly related to the process emissions and in which proportion.

The total emissions associated with MRIO sector *m* is then simply the sum of the above energy-related, process-related, and trucking-related emissions of $PM_{2.5}$:

$$\mathrm{Em}_r(m) = \mathrm{Em}_r(m,e) + \mathrm{Em}_r(m,p) + \mathrm{Em}_r(m,t) \quad \textbf{(9)}$$

Data

EXIOBASE is used for the global MRIO table and CO_2 emission inventory for the year 2021.[315] In EXIOBASE, 44 territories and 5 rest of the world regions are covered in the resolution of 163 industrial sectors. The associated CO_2 emission inventory is mapped on a one-to-one sectorial resolution. Hence, consumption-based CO_2 can be easily obtained using equation (1).

To present the results in HDI country groups, the 44 territories are aggregated in accordance with HDI classification developed by UNDP. In the case of the 5 rest of the world regions, disaggregation of both consumption-based and production-based CO_2 inventories has been conducted in proportion to the national total 2021 production-based CO_2 emissions provided by the Global Carbon Project 2022.[316] Since the 2021 MRIO table and CO_2 emission inventory in EXIOBASE is an extrapolation from historical data, adjustments are made accordingly to both the world MRIO table and CO_2 emission inventory in EXIOBASE. Specifically, change ratio of countries' GDPs[317] from 2020 to 2021 are used to adjust for the domestic intermediate and final consumptions for all countries in the global MRIO table. Change ratio of countries' exports[318] from 2020 to 2021 are used to adjust for the intermediate and final exported consumptions for all countries in the global MRIO table. 2021 global CO_2 emission data from the Global Carbon Project 2022[316] is used to adjust the total CO_2 emissions of countries. Similarly, upon the derivation of production-based $PM_{2.5}$ emission inventory using GAINS model, consumption-based $PM_{2.5}$ emission inventory can be easily obtained using equation (1). As for the 5 rest of the world regions, production-based emissions are disaggregated in proportion to 2015 $PM_{2.5}$ emission inventory of EDGAR database.[319] Consumption-based emission ratio of the 5 rest of the world regions is estimated based on CO_2 emission inventories. Having consumption-based and production-based inventories for both CO_2 and $PM_{2.5}$ emissions ready, countries are grouped according to HDI levels for results analysis (Figure 126).

World Bank population data is used to calculate the per capita CO_2 and PM2.5 emissions of different HDI development groups, in accordance with previous calculations. By simply dividing emissions with populations, Figure 127 is produced to show the difference in per capita emissions.

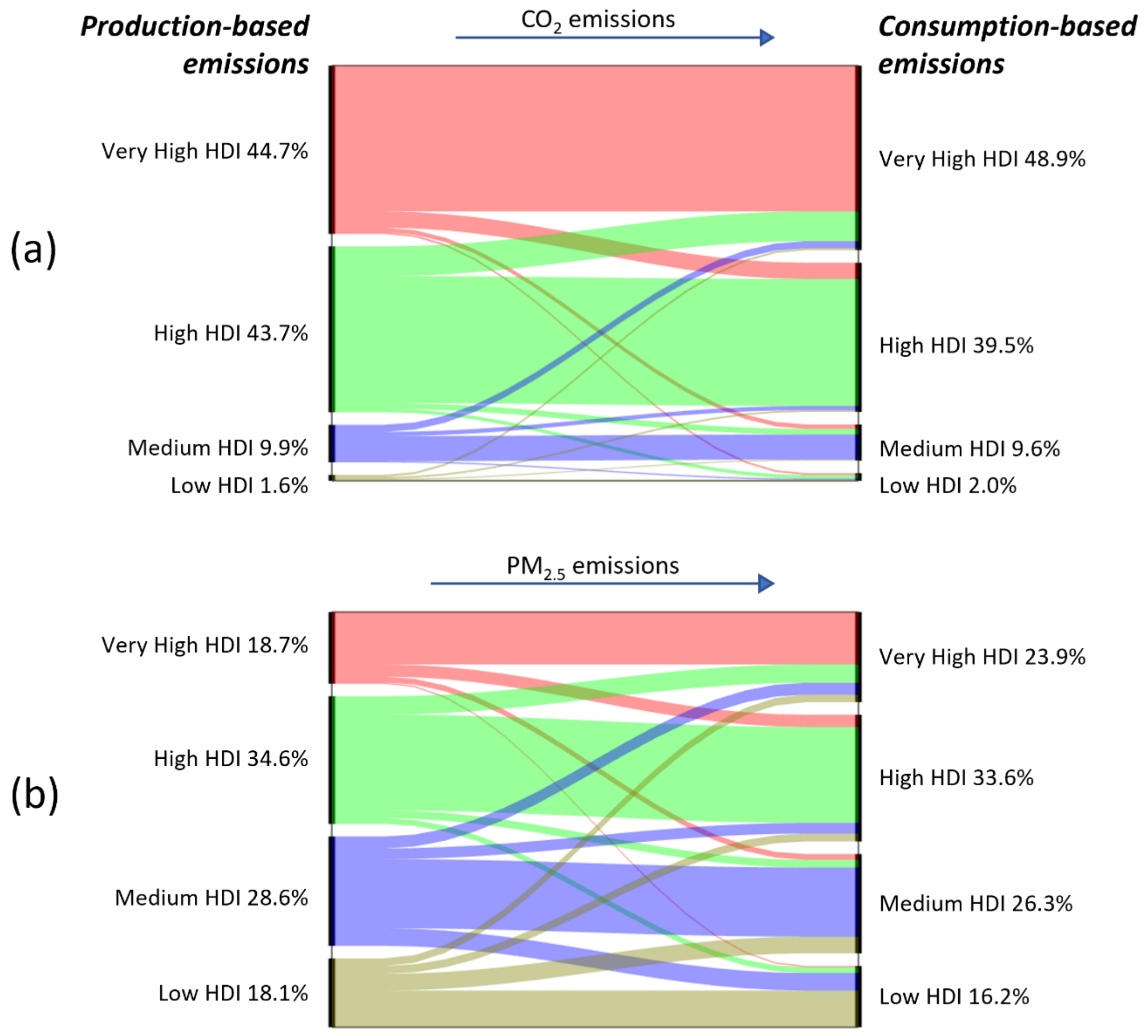

Figure 126: The flows of CO_2 and $PM_{2.5}$ emissions among countries grouped according to Human Development Index (HDI), 2021.

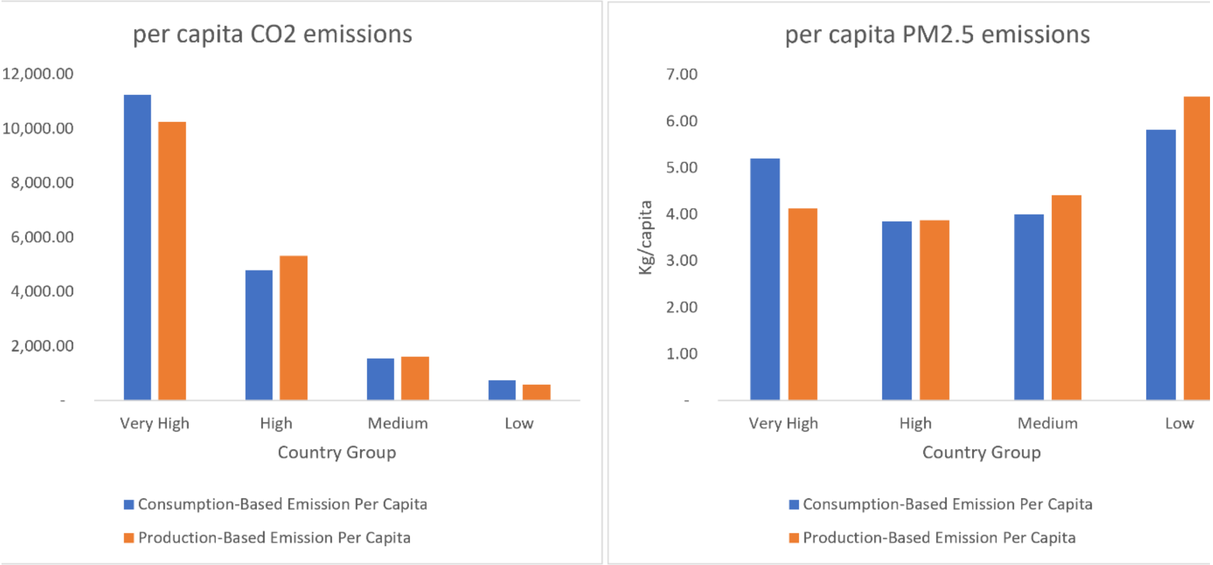

Figure 127 Per capita CO_2 and PM2.5 emissions of different HDI country groups.

Caveats

The GAINS model separating $PM_{2.5}$ emissions into three groupings appears necessary for the following reasons. First, a simplification here is done just on the basis of the total fuel use, rather than on the basis of fuel specific data, though this could be further refined in the next version of this mapping tool. Second, process-related emissions are typically related to specific sectors and thus distributing the emissions among the same cluster as the energy-related emissions seems to introduce a smearing out that is not justified. Thus, process emissions from GAINS are distributed not across all MRIO sectors, but only across those that can be clearly identified with a particular process, and those for which a process emission cannot be further resolved. Finally, trucking-related emissions are distributed among all sectors on the basis of their diesel consumption. It is assumed that the relative share of diesel consumption for road transport in each MRIO sector is generally a good proxy for the relative share in the trucking-related emissions.

In the stage of emission inventory disaggregation, simplifications and assumptions may bring uncertainties into the results. When disaggregating the five rest of the world regions, unavailable data are either filled by emissions from previous years or estimated based on the structure of embodied emissions of other pollutants. The analysis can be updated when more accurate emission inventory becomes available in the future.

Additional analysis

One of the main contributions of this work is a mapping between GAINS sectors and MRIO tables via the EXIOBASE energy extension. This is a powerful tool that maps production-based accounts of primary $PM_{2.5}$ to MRIO tables and therefore easily to consumption-based accounting schemata. So far, the analysis has focused on historical data, but the GAINS framework offers also prominently future perspectives in the form of scenarios. Thus, in conjunction with methods to project MRIO tables, the present methodology could be used to combine future emissions scenarios with future MRIO tables to assess future consumption patterns.

A number of simplifications have been made that could be refined in the next version of the mapping tool to increase the accuracy of the mapping. The mapping in this exercise is a viable tool to relate process-based calculations to consumption-based accounting frameworks. However, it is understood that the linking of frameworks that were built with different purposes (MRIO as an inventory relating economic inputs to economic outputs; GAINS as an integrated tool for air quality policy decision support based on forward looking scenarios) may result in conceptual anomalies. Furthermore, while numerical results are provided at high sectoral and regional resolution, it is important to keep in mind that at this level the results are more uncertain than at an aggregated level. Further to the mapping process, assumptions and estimations made due to unavailable data points in the inventories will exacerbate uncertainties. In the future, the present methodology will be refined to

reflect additional insights that will arise through the application of the method to different circumstances or updated inventories.

Indicator 4.2.6: Compatibility of Fossil Fuel Company Strategies with the Paris Agreement

Indicator authors

Dr Daniel Scamman

Methods

Absolute emission targets

Carbon dioxide (CO_2) is responsible for around three-quarters of total greenhouse gas emissions as measured in gigatonnes of CO_2-equivalent.[320] Climate change has multiple direct impacts on human health, as identified throughout the *Lancet* Countdown. Hence reducing CO_2 emissions from fossil fuels in order to reduce climate change will bring about significantly improved health outcomes.

This indicator connects CO_2 emissions to the activities of major oil and gas (O&G) companies that extract these fossil fuels, and analyses the extent to which their future production plans are consistent with the need to reduce CO_2 emissions in order to avoid dangerous climate change. In particular, this indicator focuses on production projections based on actual corporate activities, which may not always reflect declared targets or aspirations. Those companies whose business strategies fall short of what is required to be compliant with climate change targets can be said to be posing a danger to public health.

The indicator tracks the gap between the projected production of oil and gas companies based on their actual activities, and production trajectories consistent with the Paris target of 1.5°C of warming. The indicator is expressed as a percentage of the projected production that each company is above or below a pathway consistent with the Paris targets. If the indicator value is positive, the company projection is above the climate-consistent plan, and therefore not consistent with the climate target. The indicator analyses both international, publicly traded oil companies (IOCs) and national oil companies (NOCs), which in many cases have larger production volumes than IOCs but are subject to less public or shareholder scrutiny.

A number of organisations analyse the activities of oil and gas companies relative to climate targets, many of them aimed at investors. The Transition Pathways Initiative (TPI) publishes an annual assessment of around sixty large publicly owned O&G companies. However their data is based on companies' own disclosures and reports, so may be more aspirational rather than based on actual production projections, and excludes some of the large state-owned NOCs.[321] The Science-Based Targets Initiative (SBTi) helps businesses set science-based emissions reduction targets, but these are based on companies' own submissions rather than objective assessments of actual activity.[322] The annual Production Gap Report (PGR) tracks the discrepancy between fossil fuel production and climate-consistent production levels, but focuses on 15 countries rather than O&G companies, and relies on government production projections from national energy outlooks and targets.[323] Climate Action 100+ produce an annual Net Zero Company Benchmark;[324] their indicators primarily focuses on ambition, governance and disclosure, but a capital allocation alignment indicator generated by Carbon Tracker Initiative (CTI) evaluates the alignment of company actions with the Paris Agreement. CTI also publish an annual Two Degrees of Separation report determining potential transition risk exposure for upstream oil and gas companies, finding that the asset stranding risk of unsanctioned (pre-FID) assets is severe.[325] In 2020 Oil Change International (OCI) published production projections for eight IOCs, but this was a one-off report restricted to a small number of companies.[326] A recent journal paper found a discrepancy between the discourse and actions of four oil majors, but focussed on historic actions only.[327]

It is best practice in assessing corporate strategies to consider both absolute and intensity emission targets.[328–330] This prevents a situation where a company scores favourably by reducing its absolute emissions, but merely because its production is decreasing while its emission intensity ($kgCO_2/MJ$) could actually be increasing. Or where a company improves its emission intensity but releases more emissions overall because its production is growing. However, an emissions intensity target is not considered here due to the challenges in projecting improvements in operational efficiency (*e.g.*, reduced flaring or leakage) or transitions to lower-carbon fuels such

as renewables or nuclear. Instead this indicator uses absolute reduction targets, which are the most meaningful for reducing global total atmospheric emissions.[328]

Emission Benchmarks

An internationally-recognised standard for reporting emissions used by many companies is the GHG Protocol Corporate Standard, developed in 2004 by the World Resources Institute (WRI) and World Business Council for Sustainable Development (WBCSD).[331,332] This divides emissions into Scope 1 (direct emissions), Scope 2 (indirect energy emissions) and Scope 3 (other indirect emissions). Scope 3 emissions (and Use of Sold Products in particular) can be much higher than Scope 1 and 2 emissions (*e.g.* 86% of total emissions of European companies recently reported to the Carbon Disclosure Project, CDP), particularly for energy companies.[333,334]

Corporate benchmarks for companies operating in different sectors can be established using the Sectoral Decarbonisation Approach (SDA), developed in 2015 by the CDP, WRI, and WWF[329] and used by organisations including TPI and SBTi.[328,335] However the SDA is primarily designed for Scope 1 and 2 emissions, and most companies focus their efforts on scopes 1 and 2 emissions over which they have more direct control.[330] Also, the SDA is intended primarily to help companies in homogenous energy intensive sectors (sectors that can be described with a single physical indicator) rather than the oil and gas sector,[328] with SBTi regarding science-based emission reductions for fossil fuel companies as complex.[336] TPI have generated a sectoral decarbonisation pathway for the O&G sector, but this is an emissions intensity pathway which is not being considered for this indicator.[335]

Paris-compliant least-cost pathways typically generate projections for future global oil and gas production. This oil and gas has to be produced by O&G companies, so this production data can be used to represent the pathway O&G companies must take to be Paris-compliant. In addition, this production data (as opposed to consumption) can be assumed to cover Scope 1, 2 and 3 emissions. Hence this indicator uses oil and gas production data emanating from Paris-compliant modelling to generate Paris-compliant benchmarks for the O&G industry, rather than an SDA approach that generates separate Scope 1, 2 and 3 emission targets.

O&G sector benchmarks are typically derived using climate-compliant pathways from either the IEA or the IPCC. SBTi selected 20 1.5°C scenarios from an ensemble of over 400 peer-reviewed IPCC pathways for their analysis.[337–339] Likewise the Production Gap Report is based on a grouping of 19 IPCC 1.5°C scenarios.[323] Alternatively TPI, Carbon Tracker and OCI used IEA pathways;[325,326,335] some considered both. IEA scenarios provide a greater amount of sectoral granularity,[338] and this indicator (in alignment with TPI) uses the IEA's Net Zero Emissions by 2050 (NZE) scenario for its 1.5°C scenario from the IEA's World Energy Outlook (WEO) report. For the 2022 Lancet Countdown report, the NZE scenario in the 2021 WEO report was used.[340] However the NZE was updated for the 2022 WEO report, and this updated NZE scenario is used here for the 2023 Lancet Countdown report.[341] The updated NZE reaches net zero emissions in 2050 and is consistent with limiting the global temperature rise to 1.5°C without a temperature overshoot; 1.5°C refers to the median temperature rise, meaning there is a 50% probability of remaining below 1.5°C. Table 81 shows a comparison between fossil fuel supply in the 2021 and 2022 versions of the NZE; it shows an increase in the supply of coal and oil in 2030 which is partly offset by a reduction in natural gas but still with a slight rise in overall fossil fuel supply. Coal and oil supply then reduce in the 2030s to reach similar levels to the 2021 NZE in 2040 and 2050, while natural gas continues to decrease to substantially lower levels such that overall fossil fuel supply is lower in 2040 and 2050. Overall fossil fuel supply is still substantially greater than zero in 2050, indicating a significant reliance on offsetting technologies in other sectors.

		**2021 NZE scenario**				**2022 NZE scenario**			
		**2020**	**2030**	**2040**	**2050**	**2020**	**2030**	**2040**	**2050**
Coal	EJ	154	71.9	31.7	17.2	157	89	27	16
Oil	EJ	173	137	79.2	42.2	172	143	76	40
Natural gas	EJ	137	129	74.6	60.7	140	113	54	40
**Total**	**EJ**	**464**	**338**	**186**	**120**	**469**	**345**	**157**	**96**

Table 81: Comparison of fossil fuel supply in 2021 and 2022 IEA NZE scenarios

Emission Projections

A company's future oil and gas production (and hence emissions) can be estimated from changes in its reserves and investments as recorded in their company reports. However this is a challenging and complex process, for example with different definitions of reserves in different regions, reserves being bought and sold according to market conditions, and an observed recent sector-wide reduction in reserve holdings.[342] Hence a number of analyses base their future production projections on data from Rystad Energy,[324–326] an independent oil and gas consultancy that maintains a database of every oil and gas project in the world.[343] Historical and projected production data were downloaded for this indicator from the Rystad Energy UCube database on 24th February 2023 *i.e*. one calendar year after the data reported in the 2022 Lancet Countdown report.

Each company needs its own benchmark pathway against which its projected production is assessed. This is generated by assigning each company its own market share, based on its average market share over the historical period 2015-2020, relative to actual oil and gas production data from IEA's World Energy Balances report.[344] The company is then allocated this fraction of the future oil and gas production trajectory contained in the NZE scenario. Typically this market share is assumed to be constant over time,[329,335] though uncertainties over changing market shares may limit targets to, for example, 15 years ahead.[329] Fixing market shares to current levels allows the future performance of a firm to be compared; if a firm's market share increases, this indicates its non-compliance has increased relative to its peers, whereas compliance improves if its share falls. Rystad data indicated that some firms changed market share slightly over the period 2015-20, but many remained at similar levels. Rystad projections for the future indicated that projected productions for many firms relative to each other remained relatively stable, suggesting that many firms can be expected to rebound from short-term volatility in production.

The effect of uncertainties over future market shares for individual firms can be reduced by assessing firms in groups. Here, companies are grouped into publicly listed International Oil Companies (IOCs), including the widely-known Oil Majors, and state-owned National Oil Companies (NOCs) that in many cases have higher production levels but lower scrutiny than IOCs. The twenty largest O&G firms by projected 2040 production were included in the indicator this year; these included eleven NOCs and nine IOCs, responsible for 37% and 15.5% of total global production respectively in 2022 (52.5% overall).

Data

1. 1.5°C NZE pathway from IEA World Energy Outlook 2022.[341]
2. Oil and Gas firm production projection data from Rystad Energy[343]
3. Historical oil and gas production data from IEA World Energy Balances 2022.[344]

Caveats

There are several caveats to consider with this indicator.

The IEA NZE benchmark used in this analysis only have 50% probability of maintaining temperatures below the 1.5°C target. Although typical for this sort of analysis, it needs to be remembered that, even if O&G firms follow the Paris-compliant pathways outlined here, there is still a substantial chance that temperature targets will be exceeded.

This indicator uses projections of future production of O&G firms from the Rystad Energy database. Although a leading database in the sector, there is a significant possibility that O&G firms will follow different projection pathways to the ones projected by Rystad. These uncertainties are likely to increase over time, meaning projections in the long-term are less certain than in the shorter-term.

O&G firms are assumed here to have constant market shares. This assumption is typical for this sort of analysis but can be expected to introduce errors for at least some firms that increase over time, especially smaller firms. This can be at least partly addressed by aggregating firms into groupings such as IOCs and NOCs.

Future form of the indicator

In upcoming years, this indicator will monitor the extent to which oil and gas company strategies are compliant with the goals of the Paris Agreements, as production strategies change.

Indicator 4.2.7: Fossil Fuel and Green Bank Lending

Indicator authors

Dr Nadia Ameli, Dr Francesca Larosa, Dr Jamie Rickman

Methods

Data for bank lending to the fossil fuel and green sectors was taken from a proprietary Bloomberg dataset covering the global debt market. Fossil fuel lending is defined as being directed towards exploration, production, operation and marketing activities in oil and gas. Green lending is self-identified by the issuer as funding a project or activity with an environmental or sustainability-oriented goal i.e., renewables and energy efficiency, green building and infrastructure, agriculture, and forestry (reforestations, land-use), other sustainability (clean water, waste management).

Data is provided as total loans and bonds issued per year in USD by 787 banks from 2010 to 2021. 14% of green lending and 60% of fossil fuel lending is provided as loans, which can be issued at corporate level or as project finance. The remaining finance is provided as bonds.

The data was augmented by identifying the location of each bank's head office and ownership status (public or private) through Google search and verified through the Bloomberg terminal where necessary. Public banks, defined as banks that are majority owned by one or multiple government entities, were excluded from the dataset (over the reported period public banks provided 6% and 9% of finance to the fossil fuel and green sectors respectively). Private banks are defined as those with over 50% non-governmental ownership; they may or may not be publicly-listed companies. The location of six banks could not be identified due to ambiguity in their names, but these banks represent a negligible portion of data (<0.01% of finance in both the fossil fuel and green sectors) and they were excluded from the regional analysis. 67 countries are represented in the data (48; very high HDI level, 13; high HDI level, 4; medium HDI level, 2; low HDI level). Regional analysis shows that the fossil fuel lending data is dominated by North American banks, while European banks are the biggest group in the green sector (Figure 128). This highlights that international finance flows of fossil fuel and green debt lead to investment decisions being taken in regions different to where the impact of the investment will be felt.

The data was further augmented by identifying which banks were signatories of the Net Zero Banking Alliance (NZBA). If a bank in our dataset is a subsidiary of a bank signed to the NZBA it is also considered a signatory e.g., the Royal Bank of Canada is a signatory of the NZBA and its subsidiary RBC Capital Markets was therefore also considered a signatory. Of the 126 NZBA signatories (as of February 2023), 77 banks are represented in the data. NZBA signatories that are not present in the data may be too small (in terms of lending activity and/or loan volumes) to be covered by Bloomberg analytics or not active in the fossil fuel or green sectors.

Bank-level financing

Lending per bank per year per sector was calculated as the total of their bond and loan issuance. The contribution of banks to overall lending is highly skewed with the top 40 banks providing 83% of fossil fuel lending in the last five years of data (Figure 129).

Tracking bank-level lending activity

The metric to track the change in bank-level lending activity to the fossil fuel sector was calculated as the percentage change in their average annual lending between a pre-Paris (2010-2016) and post-Paris (2017-2021) period. The post-Paris period begins two years after the signing of the Paris Agreement, reflecting the time taken for a change in activities to be enacted.

Data

1. Bloomberg (fossil fuel and green fixed income data as of Jan 2022).
2. NZBA signatories as of February 2023.[345]

Caveats

The main caveat of the data is that it represents only a subset of investments provided by the financial sector, namely debt provided by banks. Equity investments are not covered by the data, nor are contributions from other financial actors such as institutional investors.

In addition, the labelling of a debt as 'green' is reliant on the classification by the issuer, which makes it susceptible to green-washing. There is no independent verification of this classification.

Additional analysis

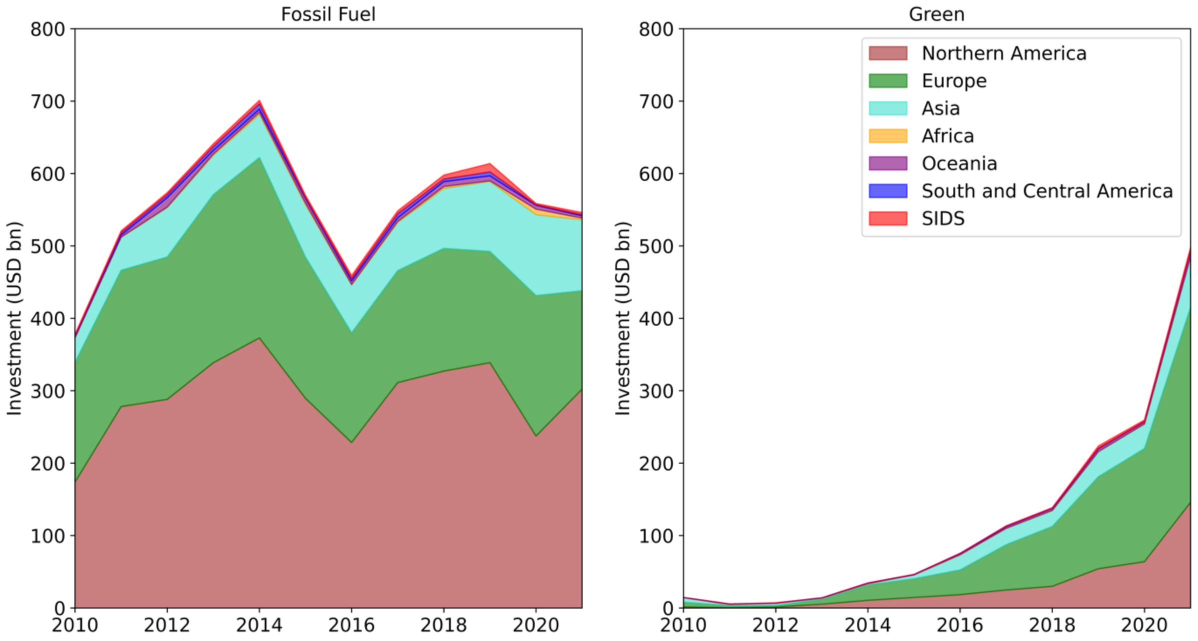

Figure 128: Lending activity of the private banking sector to the fossil fuel and green sectors between 2010 and 2021. The yearly data is disaggregated by LC regions, where the region refers to the location of the banks' headquarters.

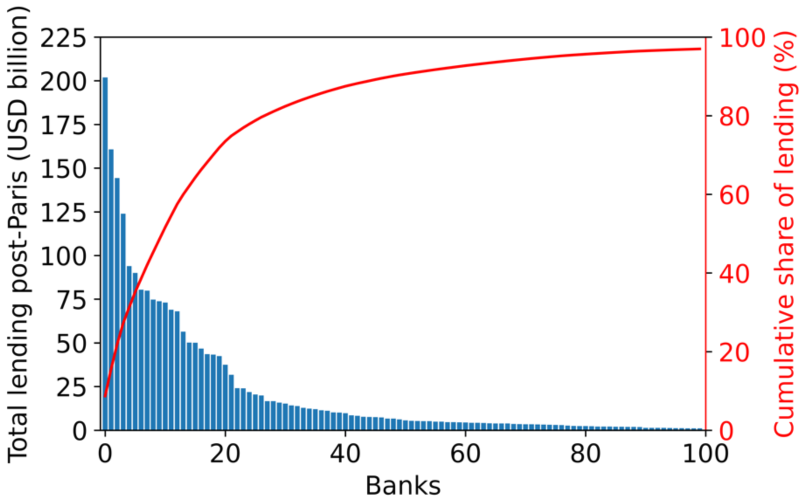

Figure 129: Contribution of the top 100 banks to fossil fuel lending in the post-Paris period (2017-2021). Blue charts show the overall finance provided. Red line shows the cumulative share of these banks to total fossil fuel lending. The top 100 banks have provided 83% of total lending in the last five years.

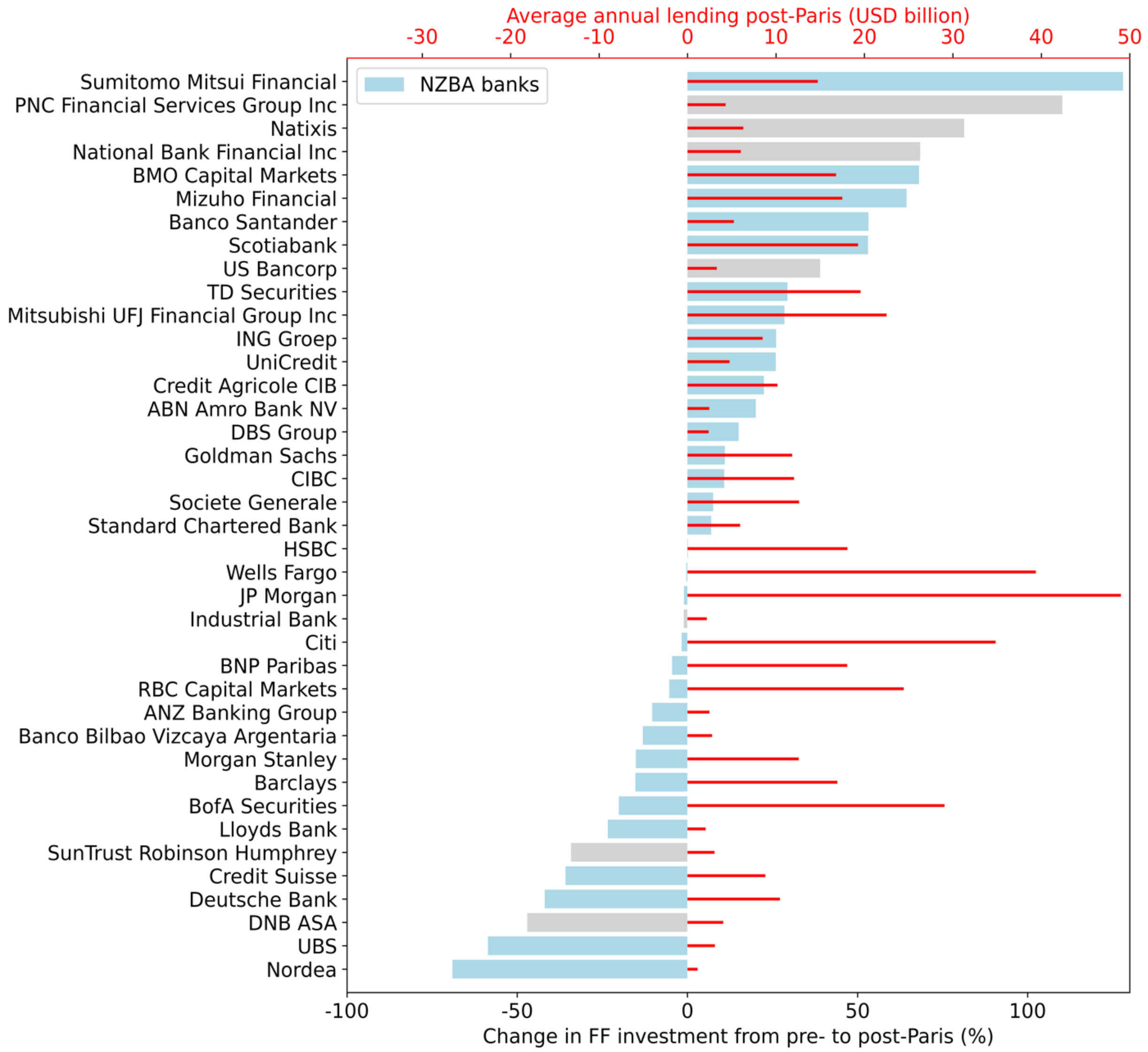

Figure 130: Lending activity of the top 40 banks in the fossil fuel sector. Bars show the percentage change in average annual investment from a pre-Paris (2010-2016) to a post-Paris Agreement (2017-2021) period. Banks that are signatories to the Net Zero Banking Alliance are show in blue. Red lines indicate the share of total lending provided by each bank in the post-Paris period.

Section 5: Public and Political Engagement

Lead Author: Prof Hilary Graham

Research Fellow: Dr Pete Lampard

Indicator 5.1: Media Engagement in Health and Climate Change

Global coverage of health and climate change

Indicator authors

Dr Lucy McAllister, Prof Wenjia Cai, Dr Pete Lampard, Olivia Pearman

Methods

Intersecting trends in coverage of climate change and health were identified in 66 newspaper sources from January 2007 through December 2022. The 65 sources are located across 35 countries, in four languages, and spanning the six World Health Organization (WHO) regions: African Region, Region of the Americas, South-East Asia Region, European Region, Eastern Mediterranean Region, and Western Pacific Region. These sources were monitored through Nexis Uni, Proquest, and Factiva databases accessed via the University of Colorado and University of York libraries.

The 2023 report of the *Lancet* Countdown adopts the search strategy developed for the 2020 and 2021 *Lancet* Countdown reports within these three databases. The search strategy was revised for the 2020 report to increase the precision of the indicator; that is, to reduce the number of 'false positives', while retaining the maximum number of 'true positives'. This was done by retaining those terms that a) produced relevant data, and b) had a low degree of polysemy (i.e. words that have fewer meanings *or* words used in fewer disciplines/domains). Testing for interaction *between* terms also enabled fewer terms to be used (for example, it was found that the term 'morbidity' would usually pull in the term 'mortality', when related to humans).

The terms were translated once the strategy had been finalised with certain terms presenting difficulties in translation. The English terms 'hay-fever' and 'West Nile', for example, correlated with more than one term in Spanish and Portuguese and the decision was made to include all relevant terms in the respective search strategies.

For the final strategy, search functions were compared across databases to ensure consistency, as different databases utilise different search filter operators. The searches were conducted with the following key words in English, Spanish, Portuguese and German respectively:

> **English:** (climate change OR global warming) AND (health OR illness OR epidemiolog* OR malnutrition OR morbidity OR fatalit* OR diarrh* OR malaria OR chikungunya OR west nile OR dengue OR hay-fever OR zika)
>
> **German:** (Klimawandel OR Globale Erwärmung) AND (Gesundheit OR Krankheit OR Epidemiolog* OR Mangelernährung OR Morbidität OR Sterblich* OR Durchfall* OR Malaria OR Chikungunya OR West-Nil-Virus OR Dengue-Fieber OR Heuschnupfen OR Zika)
>
> **Portuguese:** (mudanças climáticas OR aquecimento global) AND (saúde OR doença OR epidemiologi* OR desnutrição OR morbilidade OR fatalidade* OR diarr* OR malária OR chikungunya OR nilo do oeste OR vírus do nilo OR dengue OR febre dos fenos OR rinite alérgica OR zika)
>
> **Spanish:** (cambio climático OR calentamiento global) AND (salud OR enfermedad* OR epidemiología OR epidemiólog* OR desnutrición OR malnutrición OR morbosidad OR muert* OR diarrea* OR malaria OR paludismo OR chikungunya OR nilo del oeste OR nilo occidental OR virus del nilo OR dengue OR fiebre del heno OR rinitis alérgica OR zika)

The signal of the search strategies above was found to be strong enough (over 80% relevance in a systematically randomised sample of 500) to allow a more parsimonious approach to this indicator, requiring no screening of articles during the extraction of the data.

A separate search was undertaken with the inclusion of co-benefit terms ("cobenefit* OR "co-benefit*), in order to locate articles where health keywords, climate change keywords, *and* co-benefits keywords were included. This was undertaken in all English language sources, from 2007 to 2022.

Results were obtained from the databases by entering the relevant search strategy along with the relevant date. Counting occurred month by month and the number of returns for each source was recorded on a Microsoft Excel spreadsheet. Primary counting took place for each source along with a secondary independent count of a systematically randomised 20% sample by another researcher. Tertiary counts were undertaken where any mismatch occurred between primary and secondary counts. All counts were agreed by the whole research team.

Using the Excel spreadsheet constructed through the phases of counting, the data was organised in numerous ways for a better understanding of the patterns in coverage. These included by WHO region, by the most recent (2020) Human Development Index categories, by individual source, and now by new *Lancet* Countdown groupings. The average scores for each month (and aggregated into annual averages) were used as an adjustment for the number of sources selected per region or index category.

Data

1. Three databases were used for the core health and climate change search strategy: Nexis Uni; Proquest; and Factiva databases accessed via the University of Colorado libraries. The 65 newspaper sources are located across 35 countries, in four languages, and spanning the six World Health Organization (WHO) regions.
2. Two databases were used for the health, climate change, and co-benefit search strategies: Nexis Uni; and Factiva databases accessed via the University of Colorado libraries. The 51 newspaper sources are located across 24 countries and span the six World Health Organization (WHO) regions.

Caveats

In developing the search strategy for the 2020 and 2021 *Lancet* Countdown reports it was found that a significant portion of articles may mention both climate change and health but do not engage with them as integrated issues. Including this coverage remains important as it brings both sets of issues – health and climate change – onto the public agenda and into public awareness.

Future form of the indicator

The 2024 report will look to diversify its sources to integrate more from countries in the low and medium HDI groups.

Additional analysis

Percentage change in climate change and health and climate change coverage

Figure 131 shows the percentage change each year of articles with climate change keywords and articles with both climate change and health keywords. While the percentage change from 2020 to 2021 was positive for both climate change (54%) and health and climate change articles (27%), only health and climate change show a positive percentage change from 2021 to 2022 (12%). This increase must be seen within the context of decreasing numbers of climate change articles more generally (-15%), demonstrating an increasing proportion of climate change articles also mentioning health.

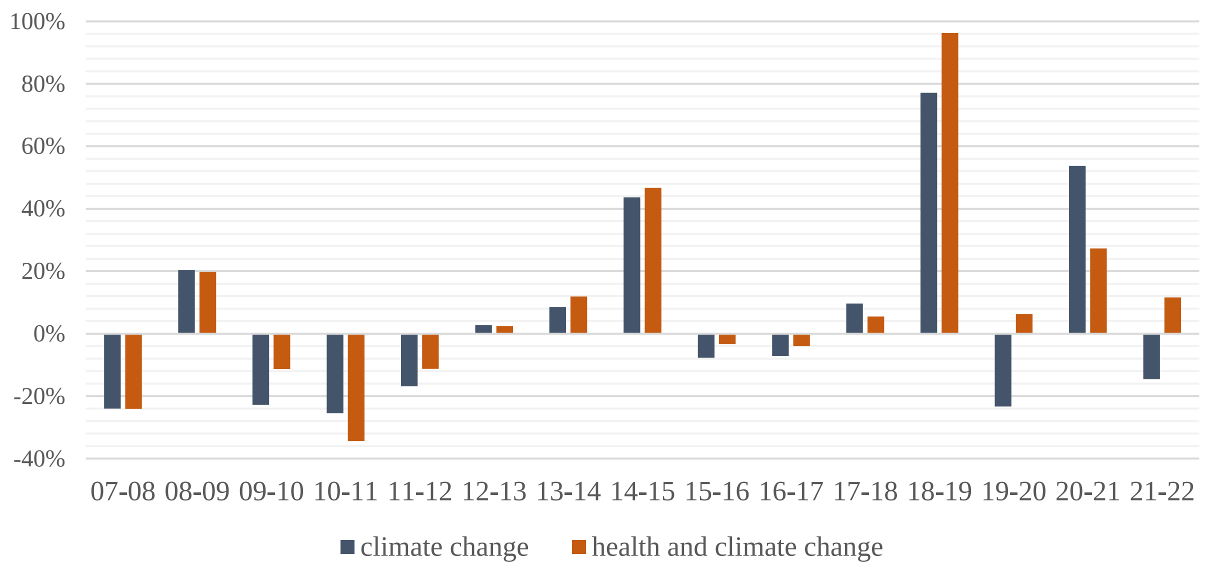

Figure 131. Annual percentage change in coverage of climate change and health and climate change from 2007 to 2022.

Total media engagement across all sources in 2022

The linear trendlines in **Figure 132** demonstrate an increase throughout 2022 in both articles with health and articles with health and climate change, though the increase is sharper in articles mentioning climate change. Due to this, the proportion of articles mentioning climate change and also mentioning health decreases throughout the year.

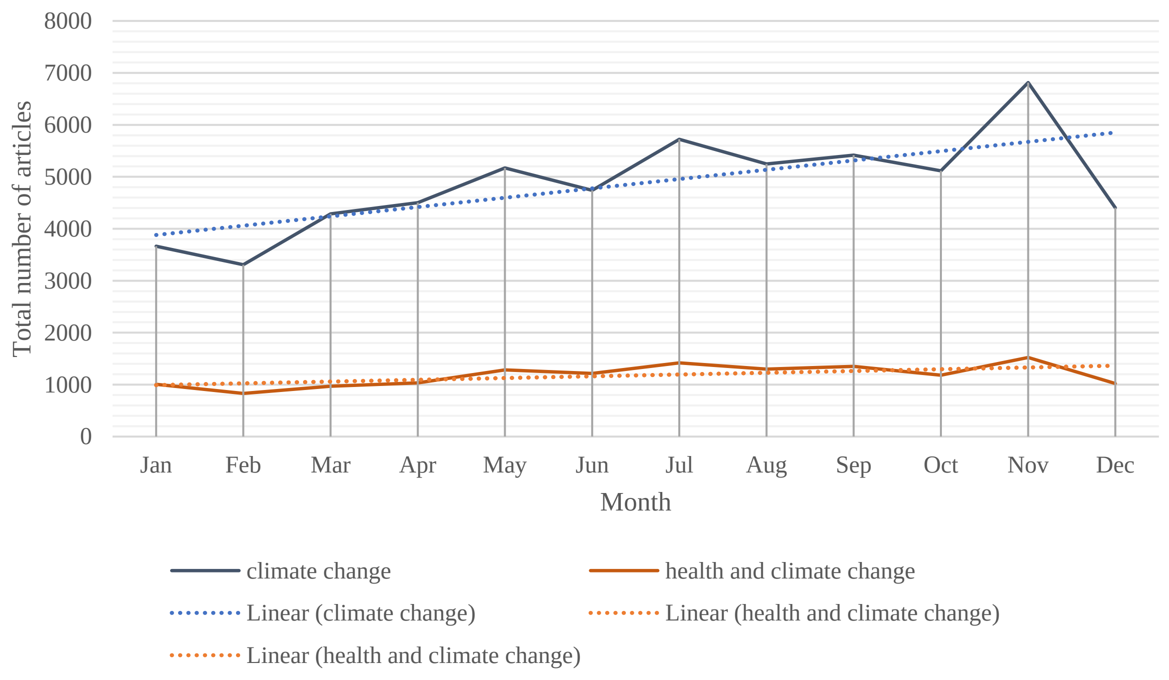

Figure 132. Total media engagement in climate change and health and climate change across all sources in 2022

Geographical distribution of newspaper coverage

Figure 133 shows the geographical distribution of articles with a co-occurrence of health and climate change keywords, by *Lancet* Countdown grouping. While North American sources (n=7 in 2022) had

the highest average coverage from 2013 to 2019, the SIDS region (n=1) now has a higher average number of sources and the next iteration of the *Lancet* Countdown will look to build the number of SIDS sources in the indicator.

Decreases in average coverage can be seen from 2021 to 2022 across four of the *Lancet* Countdown groupings (SIDS by -17%, Northern America by -21%, Europe by -6% – the three carrying the majority of health and climate change co-coverage – and Africa by -7%). With 2022 marking the end of longer trends over time for these regions with SIDS and Europe having seen increases since 2017 and North America since 2018. 2022 also saw the end of a less steep positive trend in African sources (since 2018).

Increases in average coverage can be seen in the remaining three *Lancet* Countdown regional groupings; Oceania region (by 13%), the Asian region (by 8%), and the South and Central American region (by 48%). Positive trends have continued in Asia since 2017 and South and Central America since 2018.

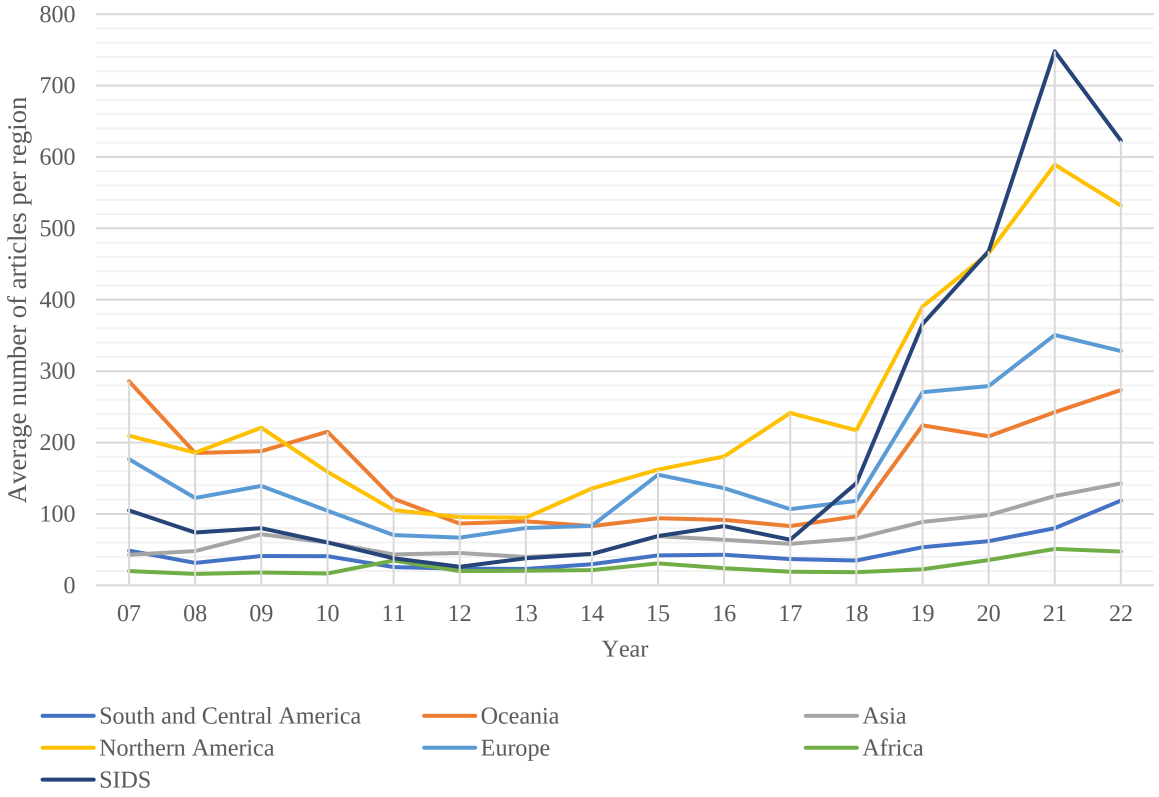

Figure 133. Average annual media engagement by Lancet Countdown grouping from 2007 to 2022.

Distribution of newspaper coverage by Human Development Index

Figure 134 shows the distribution of articles with a co-occurrence of health and climate change keywords, by Human Development Index.

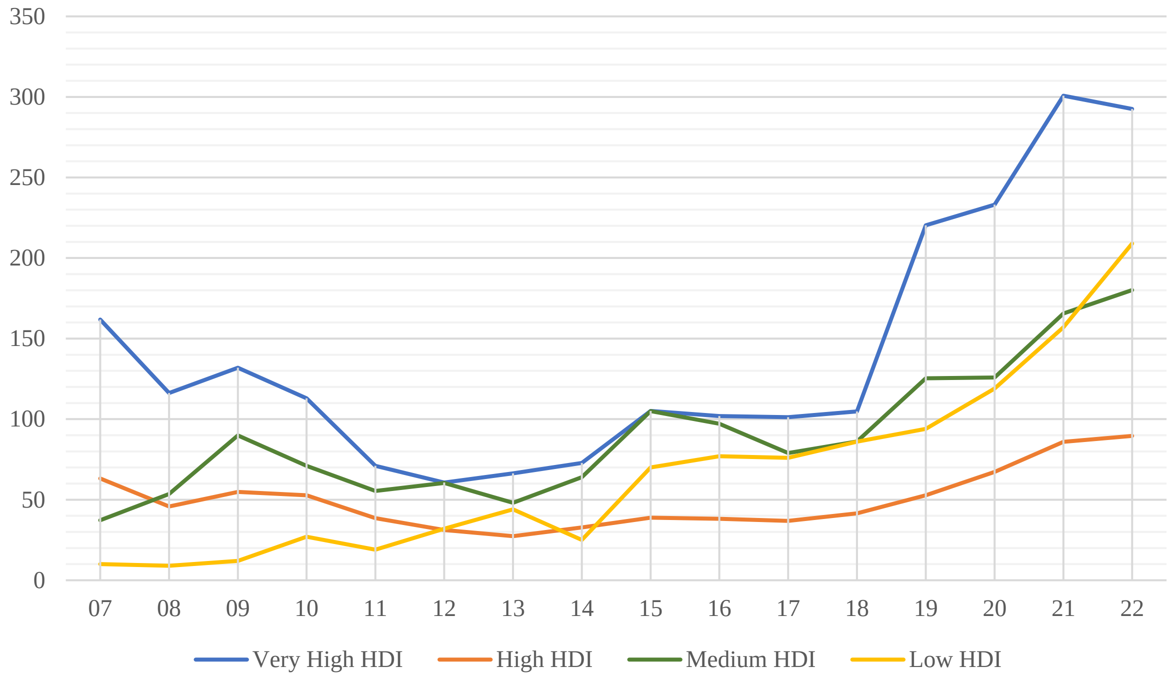

Figure 134. Average annual media engagement by HDI classification from 2007 to 2022.

Media coverage of health and climate change in China's People's Daily

In the 2023 *Lancet* Countdown report, the methodology used in the Lancet Countdown 2022 report was retained, trawling all articles, and searching the keywords in the text with the filtration process by score and keywords ratio as filtration criteria. The detailed steps of the method used in 2022 are shown as below:

Step 1 Trawling all the articles in 2022

Articles published in "People's Daily"[4] in 2022 were trawled

Step 2 Searching for "Climate Change" topic articles

Articles were searched for that contained the keywords in the topic of "Climate Change" (presented in the first column of **Table 82**).

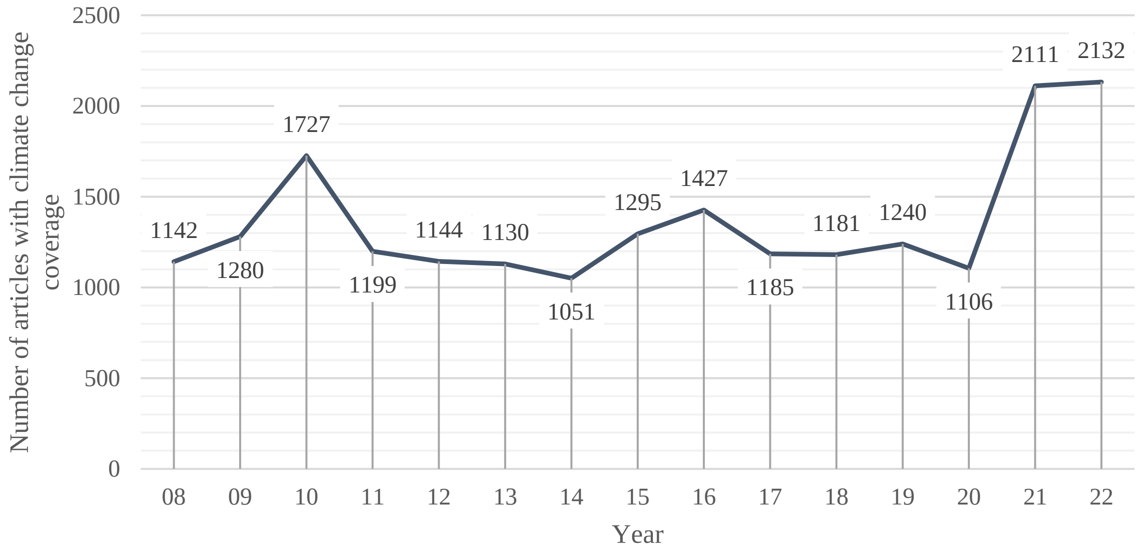

Figure 135. Number of articles identified in People's Daily containing Climate Change keywords

Step 3 Identifying articles that have both climate change and health keywords (first-round search)

In this step, a first-round of filtration was undertaken, aiming to identify articles that have both climate change and health keywords. The results were the basis for the second-round search in Step 4.

Step 4 Machine filtration on the results from step 3 by score and ratio (second-round search)

The articles obtained from step 3 were first scored based on the times of appearance of the keywords shown in the articles. For example, if the keywords of climate change and health appeared 12 times in one article, then the **score** for this article was 12. If the keyword found is one of the "mis-hit words" (phrase that containing a keyword but with different meaning), the appearance will not be counted as one score.

At the same time, the ratio of times of appearance of the keywords to the total number of characters in the article (short for "the ratio" thereafter) was also calculated. When the score and the ratio of one article are both higher than the manually-set thresholds, the article was considered relevant for health and climate change. Via this step, the numbers of relevant articles are illustrated by the orange line in **Figure 136**.

---

[4] http://paper.people.com.cn/rmrb/html/2022-03/05/nbs.D110000renmrb_01.htm

The threshold of score for each article is set to be 10, meaning the times of appearance of the keywords from both climate change and health in one article should be no less than 10. The threshold of ratio for each article was set to be no less than 1%, meaning in every 100 characters in the article, there should be no less than 1 keyword.

If the two thresholds were set too low, it would increase the workload of manual screening and increase the "false rate" of machine filtration. And if the two thresholds were set too high, it would possibly exclude the "true" articles. After several trial tests, the thresholds for score and the ratio were set as no less than 10 and 1% respectively.

Step 5: Manual screening of the results after machine filtration

The fifth step was manually screening the filtered articles. If the manual screening confirmed that the topic was Health and Climate Change, it was retained. The orange line in **Figure 136** shows the number of articles that passed the manual screening.

In **Figure 136**, the number of health and climate change coverage articles in 2022 had a contrast before and after manual screening. Before manually screening there were 92 articles, however, only 7 articles were identified by manual screening as being truly related to the topic, which was lower than the average. The abnormal results indicated that there were a lot of false positive articles appearing during the machine filtration process in 2022. After manually checking the false positive articles, it was found that public health and climate change were two separate topics that were always mentioned in the speech of the important leaders.

Titles of the 7 positive articles are presented in the additional information as **Table 83**.

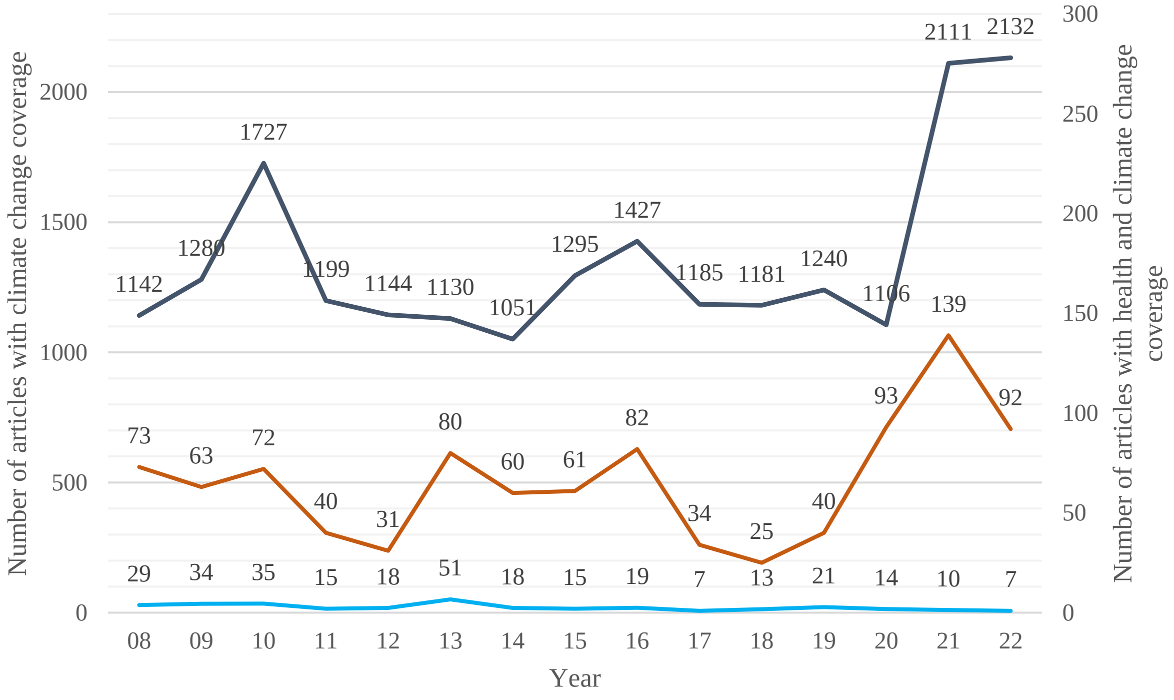

Figure 136. Numbers of articles for climate change only (dark blue line), for both health and climate change after machine filtration only (orange line), and for both health and climate change after machine filtration and manual screening (blue line)

气候变化关键词	气候变化二级关键词	健康关键词	剔除词
气候变化	霾	疟疾	口蹄疫
全球变暖	空气污染	腹泻	黑烂病
温室	大气污染	感染	珊瑚死亡
极端天气		肺炎	沙虫死亡
全球环境变化		流行病	高温加热
低碳		公共卫生	低碳水
可再生能源		卫生	健康发展
碳排放		发病	生态健康
二氧化碳排放		营养	河流健康
气候污染		精神障碍	生态环境健康
气候		发育	
全球升温		传染	
再生能源		疾患	
CO2排放		症	
~~污染~~		瘟疫	
极端气候		流感	
高温		流行感冒	
变暖		治疗	
排放		保健	
环境变化		健康	
升温		死亡	
全球温升		精神疾病	
热浪		精神病	
暴雨		登革热	
气温		饥饿	
洪水		粮食	
洪灾		有害	
气候反常		皮肤病	

野火		风湿	
山火		呼吸系统疾病	
雪灾		人类健康	
低温		人体健康	
年代际		身体健康	
冰雪		心脏病	
可持续发展		糖尿病	
海洋酸化		疾病	
静稳		热死	
温室气体		口罩	
寒潮		防护	
强降雪			
暴雪			
台风			
干旱			
水灾			
极端降雨			
冻害			

Table 82. Chinese keywords for the search in People's Daily

"Climate Change": Climate change, Global warming, Greenhouse, Extreme weather, Global environment change, Low carbon, Carbon dioxide emissions, Renewable energy, Carbon Production, Air pollution, Climate , Global worming, Renewable energy, CO2 emissions, Pollution , Extreme weather, High temperature, Warming, Emission, Environmental change, Warming, Global warming, Heat wave, Rainstorm, Temperature, Flood, Flood, Abnormal weather, Wildfire, Mountain fire, Snowstorm, Low temperature, Interdecadal, Ice and snow, Sustainable development, Ocean acidification, Stagnant, Greenhouse gas, Cold wave, Heavy snowfall, Blizzard, Typhoon, Drought, Flood, Extreme rainfall, Frost damage

Sub- level keywords of "Climate Change": Haze, Air pollution, Atmospheric Pollution

"Health": Malaria, Diarrhea, Infected, Pneumonia, Epidemic, Public health, Hygiene, Disease outbreak, Nutrition, Mental disorders, Growth, Infection, Affection, Symptom, Epidemic, Flu, Influenza, Treatment, Health care, Health, Death, Mental disease, Mental illness, Dengue, Hunger, Food, Harmful, Skin disease, Rheumatism, Respiratory diseases, Human health, Body health, Heart disease, Diabetes, Illnesses, Heat death, Mask, Protection, Survive

Removal words: Aftosa, Black shank, Coral death, Sandworm death, Heating to higher temperature, Low carbohydrate, Healthy development, Ecological health, River health, Eco-

Box 1. English translation of the Chinese keywords

Data

All the articles from 2008 to the present published on People's Daily (from the official website of People's Daily).

Additional information

Titles of the articles in 2022

文章名字	Title of the article
国务院调查组相关负责人就河南郑州 "7·20"特大暴雨灾害调查工作答记者问	Officials of The State Council investigation team answer reporters' questions on the investigation of the July 20 torrential rain disaster in Zhengzhou, Henan Province"
美国频繁遭遇重大气象灾害	The United States is frequently hit by weather disasters
非洲之角积极应对严重旱灾	Horn of Africa is responding to a severe drought
欧洲多国经受高温"烤"验	Many European countries are subjected to high temperature "roast"
加强高温天气下劳动者权益保障	To ensure the protection of laborers' rights under high temperature
携手应对全球气候灾害风险	Coping with the risk in global climate disaster together
巴基斯坦全力抗击洪涝灾害	Pakistan is fighting with floods

Table 83. Title of the health and climate change articles in People's Daily

Indicator 5.2: Individual Engagement in Health and Climate Change

Indicator authors

Prof Simon Munzert

Method

This indicator provides an individual-level indicator of public engagement. It tracks engagement with climate change and health through people's usage of the online encyclopedia Wikipedia. Over the years, Wikipedia has grown to be a major and trusted source of information that has outpaced traditional encyclopaedias in terms of reach, coverage, and comprehensiveness.[346] It is regularly listed among the ten most-visited websites worldwide.[347] The English edition covers more than 6.6 million articles and over 40,000 active editors. People around the world use it to engage in topics they are interested in. Fortunately, the traffic that goes to Wikipedia – and even that which goes to individual articles of the encyclopedia – can be analysed over time because the Wikimedia foundation makes these statistics available to everyone for free. This makes it a global indicator of what people pay attention to on a daily basis. What is more – and of particular relevance in the context of this report – the platform's health content makes it one of the most frequently used resources for information on health on the internet.[348]

The indicator

To investigate to what extent people do not only pay attention to climate change and human health in isolation, but also to the connection between both, this indicator draws on clickstream statistics from the English Wikipedia.

Clickstream refers to a dataset provided by the Wikimedia foundation.[349] It reports "streams of clicks", or in other words: how people get to a Wikipedia article and what links they click on. This is reported on a monthly basis and in pairs of resources; the first being where the visit came from, the second which page was visited. This gives an indicator of monthly-level global attention towards one issue (if both articles are representative of the same issue) or two issues (if articles come from different domains, such as climate change and health). By looking at climate change–health article pairs, an indicator of attention towards climate change consequences for human health over time can be generated.

Measurement strategy

Our approach to using clickstream data as an indicator of public engagement in climate change and health is based on the following premises:

1. The Wikipedia platform is a globally used source for information on a multitude of topics. (See https://stats.wikimedia.org/wikimedia/squids/SquidReportPageViewsPerCountryOverview.htm for an overview of Wikipedia usage by country and languages)
2. Citizens use the platform to inform themselves about topics they are interested in
3. By tracking engagement with Wikipedia articles that are related to climate change as well as with articles on health, it is possible to identify public engagement with the relationship between both topics

The following behavioural patterns are relevant for the validity of the measure as a proxy for public engagement with climate change and health:

a. A person is generally interested in the nexus between climate change and public health and informs her/himself about the topic online by, e.g., reading the Wikipedia article on Effects of

climate change on human health (https://en.wikipedia.org/wiki/Effects_of_climate_change_on_human_health)

b. A person is interested in climate change and the consumption of information about the topic, this then sparks interest in its consequences for human health. For instance, the person reads the article on Climate change (https://en.wikipedia.org/wiki/Climate_change) and then turns to the article on Malnutrition (https://en.wikipedia.org/wiki/Malnutrition)

c. A person is interested in a certain aspect of human health or consequences of climate change with an immediate impact on human health, and then turns attention to climate change issues. For instance, the person reads the article on Malaria (https://en.wikipedia.org/wiki/Malaria) and then turns to the article on climate change (https://en.wikipedia.org/wiki/Climate_change).

Indicator construction

In order to use the Wikipedia viewership statistics as a proxy for public engagement with climate change and health, it is key to select articles that are representative of these topics. To generate the populations of articles related to climate change on the one hand and health on the other, a semi-automated approach is implemented. Based on an initial set of keywords, a search was undertaken for related articles using the internal Wikipedia search.

Keywords

For climate change articles, the keywords were:

carbon dioxide, carbon emission, carbon neutral, carbon neutrality, carbon-dioxide, carbon-neutral, changing climate, climat, climate action, climate change, climate crisis, climate decay, climate emergency, climate neutrality, climate pollutant, climate variability, co2, co2 emission, decarbonisation, decarbonization, extreme temperature, extreme weather, ghge, ghges, glacial, global environmental change, global warming, green house, green new, greenhouse, greenhouse-gas, ipcc, low carbon, net zero, net-zero, ozone, renewable energy, sea ice, sea level, sphere, temperature record.

For health articles, the seed keywords were:

air pollution, asthma, cancer, communicable disease, diagnosis, diarrhoea, disease, diseases, disorder, epidemic, epidemics, epidemiolog, epidemiology, epidemy, fever, health, health care, healthcare, hunger, icide, illness, illnesses, infection, infectious, itis, malaria, malnourishment, malnutrition, measles, mental disorder, mental disorders, morbidity, mortality, ncd, ncds, non-communicable disease, noncommunicable disease, nutrition, nutrition, osis, pandemic, pandemics, pediatric, pneumonia, psychiatric, public health, sars, stunting, syndrome.

Article processing

For each search using one of the keywords, the first 100 results were extracted and identified that led to an article with a minimum word count of 300, ensuring that the articles that were chosen as seed articles had been given a certain degree of attention by Wikipedia editors, therefore being more likely to link to other relevant articles.

Next, the articles collected via the Wikipedia search for categories were screened, which are used on the Wikipedia to categorise pages in a meaningful way (e.g., using categories such as Climate change or Effects of climate_change). Those categories were then themselves screened for relevant articles. All additional articles were once more filtered such that those with a title matching one of the initial keywords was chosen. For the health-related articles, several articles were excluded manually after they turned out to be irrelevant for the purposes of this indicator. Health topics are covered extensively on Wikipedia, but a decision was made to prioritise articles and topics that, in principle, can be related to climate change. In addition, the fact that the Wikipedia page on the effects of climate change on human health offers a variety of links to further health-related articles was exploited. This

is seen as a curated list of relevant health articles and added those links to the list. The complete list of articles is recorded under Additional Information.

For the clickstream analysis, the set of articles was extended by also taking “second-level pages” into account, that is, pages that are linked to in the initially identified set of climate change or health articles and that are also somewhat related to climate change or health. Sometimes, people might not directly jump from one of the major articles on climate change to another one on health, but travel through an intermediary page (e.g., a possible individual stream of clicks could be: Climate change > Human impact on the environment > Respiratory disease). The clickstream data only allows the identification of click volume for pairs of articles, but by extending the network, it is possible to also capture clickstreams involving relevant pages that are linked in the original set of articles.

Technically, the fact that the population of health articles is far larger than the population of climate change articles does not invalidate the measurement strategy. It seems plausible that there are much more articles on health-related than on climate change-related topics because the health field is so much broader (which is one reason why the health articles cluster in the network plot is not that dense – some health topics are really far apart from each other, although both could be covering health issues that are affected by climate change). But this should not directly affect the metrics. Even if there are many more health than climate change articles, it could still be that health topics are mentioned (and clicked on) much more often in climate change articles than the other way around.

What is key in the analysis is not that one or the other topic is more extensively covered on the platform, but the co-visit patterns.

Data

The indicator draws on publicly available data from the Wikimedia foundation. It considers data from all platforms, i.e., accesses to the Wikipedia via desktop machines, mobile browsers, and mobile apps.

The clickstream data were downloaded from the Wikimedia Dumps (https://dumps.wikimedia.org/other/clickstream/). Spider traffic (i.e., traffic generated by automated bots crawling the platform) is excluded. Referrer-resource pairs (i.e., the pairs of the article of origin and the target article) that had less than 10 clicks were removed in the original dataset, so there is an expectation that the indicator will slightly underreport the actual clickstream traffic. However, it is not expected that this will add any systematic bias to the indicators in particular since the interest lies mainly in changes of engagement over time.

Clickstream data are available from November 2017 onwards. In this report, data is used from 2018 to 2022. The analyses are limited to the English Wikipedia.

The benefits of the Wikipedia usage metadata for the purpose of tracking public engagement in climate change and health are that these data (a) are globally available, (b) cover the time period of interest, (c) are collectible at virtually no cost, and, most importantly, (d) have high face validity to measure engagement in this very specific topic. Reading articles on Wikipedia is motivated by attention towards a particular issue. Individuals invest time to inform themselves about a topic, which is one manifestation of engagement. Aggregate reading behaviour can therefore be seen as an *a priori* valid approximation of public issue engagement.

Caveat

All clickstream information is only available at the aggregate level. It is not possible to link the data to information about individuals who visited the platform. Also, the data are not geo-referenced, so it is not possible to infer where page visits came from. Although the English Wikipedia is predominantly used in English-speaking countries (according to the Wikimedia Traffic Analysis Report, about 40% of the traffic on the English Wikipedia comes from the United States), it is a globally popular resource. It makes up for 50% of the global traffic to all Wikipedia language editions. Therefore, it

can be seen as a global indicator of public attention that is somewhat biased towards attention from countries such as the United States, United Kingdom, India, Canada, and Australia. Extending the analyses to other language editions will help to remedy this bias and uncover potential geographic engagement heterogeneity in the future.

More generally, the measure represents an online proxy for an offline phenomenon. In addition, it is sensitive towards the selection of articles used to capture engagement. The global popularity of the platform, which consistently ranks among the ten most visited websites worldwide, speaks in favour of its usefulness for this application. However, more direct indicators of public engagement, such as survey-based measures, might provide a useful supplement and source for validation in the future.

While the data are available for free, access to future data depends on the Wikimedia API. There is no indication of Wikimedia restricting access in the future. Instead, Wikimedia has invested in data quality and making access more robust and convenient.

Additional information

List of English Wikipedia articles used to track public engagement in climate change

2001 United Nations Climate Change Conference, 2014 People's Climate March, 2016 United Nations Climate Change Conference, 2017 People's Climate March, 2019 in climate change, 2020 in climate change, 2021 in climate change, 2021 Leaders Summit on Climate, 2021 United Nations Climate Change Conference, 2022 in climate change, 2022 United Nations Climate Change Conference, 4 Degrees and Beyond International Climate Conference, A Green New Deal, Abrupt climate change, Action for Climate Empowerment, Advisory Group on Greenhouse Gases, American Association of State Climatologists, American College & University Presidents' Climate Commitment, Amundsen-Nobile Climate Change Tower, Anjali Sharma (climate activist), Antarctic sea ice, APEC Climate Center, Arctic Climate Impact Assessment, Arctic sea ice decline, Asia-Pacific Partnership on Clean Development and Climate, Asilomar International Conference on Climate Intervention Technologies, Association for Renewable Energy and Clean Technology, Atmosphere of Earth, Attorney General of Virginia's climate science investigation, Attribution of recent climate change, Australian Greenhouse Office, Australian Renewable Energy Agency, Aventine Renewable Energy, Aviation and climate change, Avoiding Dangerous Climate Change (2005 conference), Bali Declaration by Climate Scientists, Bangladesh Climate Change Resilience Fund, Bangladesh Climate Change Trust, Bay Area Climate Collaborative, Biber-Danube interglacial, Bioclimatology, Bjerknes Centre for Climate Research, Boulder Climate Action Plan, Bristol Youth Strike 4 Climate, Business action on climate change, C40 Cities Climate Leadership Group, California Climate Action Registry, California Climate Credit, California Climate Executive Orders, Camp for Climate Action, Campaign against Climate Change, Canadian Youth Climate Coalition, Carbon dioxide, Carbon dioxide (data page), Carbon dioxide angiography, Carbon dioxide clathrate, Carbon dioxide cleaning, Carbon dioxide flooding, Carbon dioxide generator, Carbon dioxide in Earth's atmosphere, Carbon Dioxide Information Analysis Center, Carbon dioxide reforming, Carbon dioxide removal, Carbon dioxide scrubber, Carbon Neutral Cities Alliance, Carbon neutrality, Carbon Neutrality Coalition, Carbon-dioxide laser, Carbon-neutral fuel, Center for Climate and Energy Solutions, Center for Climate and Life, Center for the Study of Carbon Dioxide and Global Change, Centre for Climate Change Economics and Policy, Centre for International Climate and Environmental Research, Centre for Renewable Energy, Centre for Renewable Energy Systems Technology, Chemosphere (journal), Chesapeake Climate Action Network, Chicago Climate Action Plan, Chicago Climate Exchange, Cities for Climate Protection program, Citizens Convention for Climate, Citizens' Climate Lobby, Civil Society Coalition on Climate Change, Climate, Climate 200, Climate across Cretaceous–Paleogene boundary, Climate action, Climate Action Network, Climate Action Network Latin America, Climate Action Tracker, Climate Alliance, Climate and Clean Air Coalition to Reduce Short-Lived Climate Pollutants, Climate and Development, Climate and Development Knowledge Network, Climate and Ecological Emergency Bill, Climate and energy, Climate apocalypse, Climate appraisal, Climate as complex networks, Climate Audit, Climate Capitalism, Climate Case Ireland, Climate categories in viticulture, Climate Central, Climate change, Climate Change (Scotland) Act 2009, Climate Change Accountability Act (Bill C-224), Climate change acronyms, Climate Change Act 2008, Climate change adaptation, Climate change adaptation strategies on the German coast, Climate Change Agreement (UK), Climate change and agriculture in the United States, Climate change and birds, Climate change and children, Climate change and cities, Climate Change and Emissions Management Amendment Act, Climate change and fisheries, Climate change and gender, Climate change and indigenous peoples, Climate change and infectious diseases, Climate change and invasive species, Climate change and poverty, Climate Change and Sustainable Energy Act 2006, Climate change and wildfires, Climate change art, Climate Change Authority, Climate Change Capital, Climate Change Commission, Climate Change Committee, Climate change conspiracy theory, Climate Change Denial, Climate Change Denial Disorder, Climate change education, Climate change feedback, Climate change in Afghanistan, Climate change in Alabama, Climate change in Alaska, Climate change in Algeria, Climate change in American Samoa, Climate change in Antarctica, Climate change in Argentina, Climate change in Arizona, Climate change in Arkansas, Climate change in Asia, Climate change in Australia, Climate change in Austria, Climate change in Bangladesh, Climate change in Belgium, Climate change in Bosnia and

Herzegovina, Climate change in Brazil, Climate change in California, Climate change in Cambodia, Climate change in Canada, Climate change in China, Climate change in Colorado, Climate change in Connecticut, Climate change in Cyprus, Climate change in Delaware, Climate change in Ethiopia, Climate change in Fiji, Climate change in Finland, Climate change in Florida, Climate change in France, Climate change in Georgia (U.S. state), Climate change in Germany, Climate change in Ghana, Climate change in Greenland, Climate change in Grenada, Climate change in Guam, Climate change in Guatemala, Climate change in Honduras, Climate change in Idaho, Climate change in Illinois, Climate change in India, Climate change in Indiana, Climate change in Indonesia, Climate change in Iowa, Climate change in Iran, Climate change in Iraq, Climate change in Israel, Climate change in Italy, Climate change in Japan, Climate change in Jordan, Climate change in Kansas, Climate change in Kentucky, Climate change in Kenya, Climate change in Kyrgyzstan, Climate change in Liberia, Climate change in Louisiana, Climate change in Luxembourg, Climate change in Madagascar, Climate change in Maine, Climate change in Malaysia, Climate change in Maryland, Climate change in Massachusetts, Climate change in Mexico, Climate change in Michigan, Climate change in Minnesota, Climate change in Mississippi, Climate change in Missouri, Climate change in Mongolia, Climate change in Montana, Climate change in Morocco, Climate change in Myanmar, Climate change in Nebraska, Climate change in Nepal, Climate change in Nevada, Climate change in New Hampshire, Climate change in New Jersey, Climate change in New Mexico, Climate change in New York (state), Climate change in New York City, Climate change in New Zealand, Climate change in Nigeria, Climate change in North Carolina, Climate change in North Dakota, Climate change in North Korea, Climate change in Norway, Climate change in Ohio, Climate change in Oklahoma, Climate change in Oregon, Climate change in Pakistan, Climate change in Pennsylvania, Climate change in popular culture, Climate change in Puerto Rico, Climate change in Rhode Island, Climate change in Russia, Climate change in Saskatchewan, Climate change in Scotland, Climate change in Senegal, Climate change in South Africa, Climate change in South Asia, Climate change in South Carolina, Climate change in South Dakota, Climate change in South Korea, Climate change in Spain, Climate change in Sri Lanka, Climate change in Suriname, Climate change in Sweden, Climate change in Taiwan, Climate change in Tanzania, Climate change in Tennessee, Climate change in Thailand, Climate change in the Arctic, Climate change in the Caribbean, Climate change in the Democratic Republic of the Congo, Climate change in the Gambia, Climate change in the Maldives, Climate change in the Marshall Islands, Climate change in the Middle East and North Africa, Climate change in the Netherlands, Climate change in the Pacific Islands, Climate change in the Philippines, Climate change in the Republic of Ireland, Climate change in the United Kingdom, Climate change in the United States, Climate change in Turkey, Climate change in Tuvalu, Climate change in Uganda, Climate change in Uruguay, Climate change in Utah, Climate change in Vermont, Climate change in Vietnam, Climate change in Virginia, Climate change in Washington (state), Climate change in Washington, D.C., Climate change in West Virginia, Climate change in Wisconsin, Climate change in Wyoming, Climate Change Levy, Climate change litigation, Climate change mitigation, Climate change mitigation framework, Climate Change Performance Index, Climate change policy of California, Climate change policy of the George W. Bush administration, Climate change policy of the United States, Climate Change Response (Emissions Trading) Amendment Act 2008, Climate Change Response (Zero Carbon) Amendment Act, Climate Change Response Act 2002, Climate change scenario, Climate Change Science Program, Climate Change TV, Climate change vulnerability, Climate Change: Global Risks, Challenges and Decisions, Climate classification, Climate Commission, Climate commitment, Climate communication, Climate Council, Climate crisis, Climate Crisis Advisory Group, Climate Data Operators, Climate debt, Climate Denial Crock of the Week, Climate Disclosure Standards Board, Climate Doctrine of the Russian Federation, Climate Dynamics, Climate emergency declaration, Climate emergency declarations in Australia, Climate emergency declarations in New Zealand, Climate emergency declarations in the United Kingdom, Climate engineering, Climate ensemble, Climate fiction, Climate footprint, Climate forcing, Climate governance, Climate Hawks Vote, Climate Hustle, Climate inertia, Climate Investment Funds, Climate justice, Climate Justice Action, Climate Justice Alliance, Climate Justice Now!, Climate Law and Governance Initiative, Climate Leadership Council, Climate migrant, Climate Mirror, Climate model, Climate Monitoring and Diagnostics Laboratory, Climate movement, Climate of Agra, Climate of Albania, Climate of ancient Rome, Climate of Argentina, Climate of Armenia, Climate of Australia, Climate of Azerbaijan, Climate of Bangladesh, Climate of Barcelona, Climate of Bihar, Climate of Bilbao, Climate of Brazil, Climate of Budapest, Climate of Buenos Aires, Climate of Cebu, Climate of Chile, Climate of China, Climate of Colombia, Climate of Cyprus, Climate of Delhi, Climate of Dubai, Climate of East Anglia, Climate of Ecuador, Climate of Egypt, Climate of Estonia, Climate of Ethiopia, Climate of Finland, Climate of Ghana, Climate of Gibraltar, Climate of Greece, Climate of Gujarat, Climate of Himachal Pradesh, Climate of Hungary, Climate of Iceland, Climate of India, Climate of Indonesia, Climate of Ireland, Climate of Italy, Climate of Kaziranga National Park, Climate of Kolkata, Climate of Kosovo, Climate of Lisbon, Climate of Madrid, Climate of Malta, Climate of Manitoba, Climate of Mexico, Climate of Moscow, Climate of Mumbai, Climate of Myanmar, Climate of New England, Climate of New Zealand, Climate of Nigeria, Climate of Norway, Climate of Nova Scotia, Climate of Pakistan, Climate of Paraguay, Climate of Paris, Climate of Peru, Climate of Porto, Climate of Puerto Rico, Climate of Rajasthan, Climate of Romania, Climate of Rome, Climate of Russia, Climate of Saudi Arabia, Climate of Seoul, Climate of Serbia, Climate of Sochi, Climate of South Africa, Climate of South Brazil, Climate of Southeast Brazil, Climate of Spain, Climate of Svalbard, Climate of Sweden, Climate of Tamil Nadu, Climate of Tasmania, Climate of the British Isles, Climate of the Falkland Islands, Climate of the Philippines, Climate of the United Kingdom, Climate of the United States, Climate of Turkey, Climate of Uruguay, Climate of Uttar Pradesh, Climate of Valencia, Climate of Venezuela, Climate of Vietnam, Climate of Wales, Climate of West Bengal, Climate of Zambia, Climate One, Climate Policy (journal), Climate Policy Initiative, Climate prediction, Climate Prediction Center, Climate psychology, Climate Refugees, Climate reparations, Climate Research (journal), Climate resilience, Climate restoration, Climate risk,

Climate risk insurance, Climate risk management, Climate Savers Computing Initiative, Climate Science Legal Defense Fund, Climate Science Rapid Response Team, Climate security, Climate sensitivity, Climate Solutions Caucus, Climate Solutions Road Tour, Climate spiral, Climate Stewardship Acts, Climate TRACE, Climate variability and change, Climate Vulnerability Monitor, Climate Vulnerable Forum, Climate Watch, Climate Week NYC, Climate-Alliance Germany, Climate-friendly gardening, Climate-friendly school, Climate-smart agriculture, Climate: Long range Investigation, Mapping, and Prediction, Climatic Change (journal), Climatic geomorphology, Climatic regions of Argentina, Climatic Research Unit documents, Climatic Research Unit email controversy, Climatological normal, Climatology, Cloud formation and climate change, Co-benefits of climate change mitigation, CO2 (opera), CO2 Coalition, CO2 fertilization effect, CO2 is Green, CO2balance, Committee on Climate Change Science and Technology Integration, Cool It: The Skeptical Environmentalist's Guide to Global Warming, Copenhagen Climate Challenge, Copper in renewable energy, Criticism of the IPCC Fourth Assessment Report, Danube-Gunz interglacial, Debate over China's economic responsibilities for climate change mitigation, Decarbonisation measures in proposed UK electricity market reform, Deep Decarbonization Pathways Project, Deforestation and climate change, Dendroclimatology, Department of the Environment, Climate and Communications, Description of the Medieval Warm Period and Little Ice Age in IPCC reports, Desert climate, Disability and climate change, Drawdown (climate), Earth rainfall climatology, East Asia Climate Partnership, Economic impacts of climate change, Economics of climate change, Economics of climate change mitigation, Economists' Statement on Climate Change, Ecosphere (social enterprise), Ed Hawkins (climatologist), Effects of climate change, Effects of climate change on agriculture, Effects of climate change on ecosystems, Effects of climate change on human health, Effects of climate change on mental health, Effects of climate change on oceans, Effects of climate change on plant biodiversity, Effects of climate change on small island countries, Effects of climate change on terrestrial animals, Effects of climate change on the water cycle, Effects of global warming, Effects of global warming on human health, Effects of global warming on humans, Effects of global warming on the United Arab Emirates, Electrochemical reduction of carbon dioxide, Euro-Mediterranean Center on Climate Change, European Assembly for Climate Justice, European Climate Change Programme, European Climate Exchange, European Climate Forum, European Climate Foundation, European Climate, Infrastructure and Environment Executive Agency, European Renewable Energy Council, European Union climate and energy package, Evangelical Climate Initiative, Exposing Microorganisms in the Stratosphere, Extreme weather, ExxonMobil climate change denial, Federal Ministry for Economic Affairs and Climate Action, Fennec (climate program), Fourth National Climate Assessment, Freedom of Information requests to the Climatic Research Unit, G8 Climate Change Roundtable, Garnaut Climate Change Review, Generation Climate Europe, Geologic temperature record, German Climate Action Plan 2050, German Climate Consortium, German Renewable Energy Sources Act, Ghana Climate Innovation Centre, Glasgow Climate Pact, Global Atmosphere Watch, Global Climate Action (portal), Global Climate Action Summit, Global Climate and Energy Project, Global Climate and Health Alliance, Global Climate Coalition, Global Climate March, Global Climate Network, Global climate regime, Global Covenant of Mayors for Climate & Energy, Global Day of Climate Action 2020, Global Environmental Change, Global Historical Climatology Network, Global Roundtable on Climate Change, Global temperature record, Global warming controversy, Global warming game, Global warming hiatus, Global Warming Pollution Reduction Act of 2007, Global warming potential, Global Warming Solutions Act of 2006, Global Warming: The Signs and The Science, Global Warming: What You Need to Know, Glossary of climate change, Gorgon Carbon Dioxide Injection Project, Grande-Synthe climate case, Great March for Climate Action, Green Climate Fund, Green House Data, Green New Deal, Greenhouse and icehouse Earth, Greenhouse debt, Greenhouse Development Rights, Greenhouse effect, Greenhouse gas, Greenhouse gas emissions, Greenhouse gas emissions by Australia, Greenhouse gas emissions by China, Greenhouse gas emissions by Russia, Greenhouse gas emissions by the United Kingdom, Greenhouse gas emissions by the United States, Greenhouse gas emissions by Turkey, Greenhouse gas emissions from agriculture, Greenhouse gas emissions in Kentucky, Greenhouse gas inventory, Greenhouse gas monitoring, Greenhouse Gas Pollution Pricing Act, Greenhouse Gases Observing Satellite, Greenhouse Gases Observing Satellite-2, Greenhouse Mafia, Greenhouse Solutions with Sustainable Energy, Ground-level ozone, Gussing Renewable Energy, High Council on Climate, High Level Advisory Group on Climate Financing, High Plains Regional Climate Center, Highest temperature recorded on Earth, Highland temperate climate, Historical climatology, History of climate change policy and politics, History of climate change science, Holocene climatic optimum, Homogenization (climate), How Global Warming Works, How to Prepare for Climate Change, Human rights and climate change, Humid temperate climate, Ice cap climate, Idealized greenhouse model, Illustrative model of greenhouse effect on climate change, Index of climate change articles, India Climate Collaborative, Indian Network on Climate Change Assessment, Indian Youth Climate Network, Indigenous Peoples Climate Change Assessment Initiative, Individual action on climate change, Inside Climate News, Instrumental temperature record, Intergovernmental Panel on Climate Change, Interim Climate Change Committee, International Climate Change Partnership, International Comprehensive Ocean-Atmosphere Data Set, International Conference on Climate Change, International Geosphere-Biosphere Programme, International Indigenous Peoples Forum on Climate Change, International Journal of Climatology, International Journal of Greenhouse Gas Control, International Renewable Energy Agency, International Satellite Cloud Climatology Project, IPCC Fifth Assessment Report, IPCC First Assessment Report, IPCC Fourth Assessment Report, IPCC list of greenhouse gases, IPCC Second Assessment Report, IPCC Sixth Assessment Report, IPCC Summary for Policymakers, IPCC supplementary report, 1992, IPCC Third Assessment Report, Johannesburg Renewable Energy Coalition, Journal for Geoclimatic Studies, Journal of Applied Meteorology and Climatology, Journal of Climate, Laboratoire des sciences du climat et de l'environnement, Land surface effects on climate, Life-cycle greenhouse gas emissions of energy sources, Liquid carbon dioxide, List of abbreviations relating to climate change, List of books about renewable energy, List of climate activists, List of climate

change books, List of climate change initiatives, List of climate engineering topics, List of climate research satellites, List of climate scientists, List of countries and territories by extreme temperatures, List of countries by carbon dioxide emissions, List of countries by carbon dioxide emissions per capita, List of countries by greenhouse gas emissions, List of countries by greenhouse gas emissions per capita, List of extreme temperatures in Australia, List of extreme temperatures in Canada, List of extreme temperatures in Denmark, List of extreme temperatures in Finland, List of extreme temperatures in France, List of extreme temperatures in Germany, List of extreme temperatures in Greece, List of extreme temperatures in Italy, List of extreme temperatures in Japan, List of extreme temperatures in Portugal, List of extreme temperatures in Spain, List of extreme temperatures in Sweden, List of extreme temperatures in Vatican City, List of extreme weather records in Pakistan, List of films about renewable energy, List of ministers of climate change, List of periods and events in climate history, List of planned renewable energy projects, List of renewable energy organizations, List of renewable energy topics by country and territory, List of school climate strikes, List of U.S. states and territories by carbon dioxide emissions, List of women climate scientists and activists, Lists of renewable energy topics, London Climate Change Agency, Low Carbon Communities, Low Carbon Vehicle Event, Lowest temperature recorded on Earth, Major Economies Forum on Energy and Climate Change, Maldivian Youth Climate Network, Mandatory renewable energy target, Mayors National Climate Action Agenda, Media coverage of climate change, Mercator Research Institute on Global Commons and Climate Change, Microclimate, Mid-24th century BCE climate anomaly, Midwestern Greenhouse Gas Reduction Accord, Millennium Alliance for Humanity and the Biosphere, Minister for Climate Change (New Zealand), Minister for Climate Change and Energy, Ministry of Climate and Environment (Poland), Ministry of Climate Change (Pakistan), Ministry of Climate, Energy and Utilities (Denmark), Ministry of Economic Affairs and Climate Policy, Ministry of Electricity and Renewable Energy (Egypt), Ministry of Environment, Forest and Climate Change, Ministry of New and Renewable Energy, Monsoon continental climate, Monthly Climatic Data for the World, Mumbai Climate Action Plan, Music of the Spheres World Tour, Muslim Seven Year Action Plan on Climate Change, Mycorrhizae and climate change, National Action Plan for Climate Change, National Climate Assessment, National Climate Change Secretariat, National Climatic Data Center, National Initiative on Climate Resilient Agriculture, National Oceanic and Atmospheric Administration Climate and Societal Interactions Program, National Renewable Energy Action Plan, National Solar Conference and World Renewable Energy Forum 2012, Nature Climate Change, New and Renewable Energy Authority, New England Governors and Eastern Canadian Premiers Climate Change Action Plan 2001, New South Wales Greenhouse Gas Abatement Scheme, New York City Panel on Climate Change, Nigeria Renewable Energy Master Plan, Noordwijk Climate Conference, North African climate cycles, Nuclear power proposed as renewable energy, NZ Climate Party, Office of Energy Efficiency and Renewable Energy, OneClimate, Ozone, Ozone depletion, Ozone depletion and climate change, Pacific Climate Warriors, Palaeogeography, Palaeoclimatology, Palaeoecology, Pan African Climate Justice Alliance, Pan-African Media Alliance on Climate Change, Pan-Canadian Framework on Clean Growth and Climate Change, Pastoral Greenhouse Gas Research Consortium, People's Climate Movement, Photochemical reduction of carbon dioxide, Photoelectrochemical reduction of carbon dioxide, Physical properties of greenhouse gases, Poland National Renewable Energy Action Plan, Political economy of climate change, Politics of climate change, Portal:Climate change, Portal:Renewable energy, Potsdam Institute for Climate Impact Research, Premier's Climate Change Council, Presbyterian Church (U.S.A.) Carbon Neutral Resolution, Presidential Climate Action Plan, Program for Climate Model Diagnosis and Intercomparison, Program on Energy Efficiency in Artisanal Brick Kilns in Latin America to Mitigate Climate Change, Proxy (climate), Psychological impact of climate change, Psychology of climate change denial, Public opinion on climate change, Punjab Renewable Energy Systems Pvt. Ltd., R20 Regions of Climate Action, Rapid Climate Change-Meridional Overturning Circulation and Heatflux Array, Recognizing the duty of the Federal Government to create a Green New Deal, Reflective surfaces (climate engineering), Regional climate levels in viticulture, Regional Greenhouse Gas Initiative, Regulation of greenhouse gases under the Clean Air Act, Renewable energy, Renewable Energy (journal), Renewable energy and mining, Renewable Energy Certificate (United States), Renewable Energy Certificate System, Renewable Energy Certificates Registry, Renewable energy commercialization, Renewable energy cooperative, Renewable energy debate, Renewable energy in Brunei, Renewable energy in developing countries, Renewable energy in Luxembourg, Renewable energy industry, Renewable Energy Payments, Renewable energy policy of Bangladesh, Renewable energy sculpture, Renewable Energy Sources and Climate Change Mitigation, Runaway greenhouse effect, Running on Climate, San Diego Climate Action Plan, San Francisco Climate Action Plan, Save the Climate, School Strike for Climate, Scientific consensus on climate change, Scorcher: The Dirty Politics of Climate Change, Sea ice emissivity modelling, Sea ice growth processes, Sea ice thickness, Sea level rise, Seawater greenhouse, September 2019 climate strikes, Singularity (climate), Skin temperature (of an atmosphere), Soft climate change denial, Soil-plant-atmosphere continuum, Solar activity and climate, Solar Renewable Energy Certificate, South Pacific Sea Level and Climate Monitoring Project, Space mirror (climate engineering), Space-based measurements of carbon dioxide, Special Report on Climate Change and Land, Special Report on the Ocean and Cryosphere in a Changing Climate, State of the Climate, Stratospheric Processes And their Role in Climate, Subhumid temperate climate, Supercritical carbon dioxide, Surface Ocean Lower Atmosphere Study, Surveys of scientists' views on climate change, Sweden National Renewable Energy Action Plan, Table of historic and prehistoric climate indicators, Tarawa Climate Change Conference, Task Force on Climate Related Financial Disclosures, Temperature record of the last 2,000 years, Template:Climate change in Canada, Template:Climate-change-book-stub, Template:Climate-change-stub, Template:Climate-journal-stub, Template:Climate-stub, Territorial Approach to Climate Change, The Climate Group, The Climate Mobilization, The Climate Reality Project, The Climate Registry, The Discovery of Global Warming, The Doubt Machine: Inside the Koch Brothers' War on Climate Science, The Great Derangement: Climate Change and the Unthinkable, The Great Global

Warming Swindle, The Greenhouse Conspiracy, The Islamic Declaration on Global Climate Change, The New Climate War, Theoretical and Applied Climatology, Thornthwaite climate classification, Tianjin Climate Exchange, Timeline of international climate politics, Tipping points in the climate system, Top contributors to greenhouse gas emissions, Tornado climatology, Toronto Conference on the Changing Atmosphere, Transatlantic Climate Bridge, Transient climate response to cumulative carbon emissions, Transportation and Climate Initiative, Trewartha climate classification, Tropical cyclones and climate change, Tropospheric ozone depletion events, U.S. Climate Action Partnership, U.S. Climate Change Technology Program, U.S. Special Presidential Envoy for Climate, UK Climate Assembly, UK Health Alliance on Climate Change, United Kingdom Climate Change Programme, United Kingdom National Renewable Energy Action Plan, United Nations Special Envoy on Climate Change, United States Climate Alliance, United States federal register of greenhouse gas emissions, United States House Select Committee on Energy Independence and Global Warming, United States House Select Committee on the Climate Crisis, United States rainfall climatology, United States Senate Environment Subcommittee on Clean Air, Climate and Nuclear Safety, Urban climate, Urban climatology, Urbanization and Global Environmental Change Project, US Climate Reference Network, VAMOS Ocean-Cloud-Atmosphere-Land Study, Vatican Climate Forest, Wadebridge Renewable Energy Network, Walloon platform for the IPCC, Waterborne disease and climate change, Weather, Climate, and Society, Western Climate Initiative, Western Hemisphere Warm Pool, Weyburn-Midale Carbon Dioxide Project, White House Office of Energy and Climate Change Policy, Whole Atmosphere Community Climate Model, Women in climate change, World Climate Change Conference, Moscow, World Climate Conference, World Climate Programme, World Mayors Council on Climate Change, World People's Conference on Climate Change, World Renewable Energy Network, World Wide Views on Global Warming, Wuppertal Institute for Climate, Environment and Energy, XCO2, Yale Program on Climate Change Communication, Young Voices on Climate Change, Youth Climate Movement

List of English Wikipedia articles used to track public engagement in health

1510 influenza pandemic, 1557 influenza pandemic, 1707-08 Iceland smallpox epidemic, 1775–1782 North American smallpox epidemic, 1793 Philadelphia yellow fever epidemic, 1837 Great Plains smallpox epidemic, 1847 North American typhus epidemic, 1856 Guam smallpox epidemic, 1862 Pacific Northwest smallpox epidemic, 1870 Barcelona yellow fever epidemic, 1881–1896 cholera pandemic, 1896 Gloucester smallpox epidemic, 1906 malaria outbreak in Ceylon, 1915 typhus and relapsing fever epidemic in Serbia, 1918 flu, pandemic in India, 1974 smallpox epidemic in India, 1983 West Bank fainting epidemic, 1998 Winter Olympics flu epidemic, 2009 swine flu pandemic, 2009 swine flu pandemic in India, 2009 swine flu pandemic timeline, 2013 Swansea measles epidemic, 2018 Madagascar measles outbreak, 2021 South Sudan disease outbreak, 412 BC epidemic, Abrazo Community Health Network, Academy of Nutrition and Dietetics, Access Health CT, Accredited Social Health Activist, Action on Smoking and Health, Acute eosinophilic pneumonia, Adenovirus infection, Adult-onset Still's disease, AdventHealth, AdventHealth Celebration, AdventHealth Lake Wales, AdventHealth Nicholson Center, AdventHealth Ocala, AdventHealth Orlando, AdventHealth Shawnee Mission, AdventHealth station, Adventist Health, Adventist Health Bakersfield, Adventist Health Community Care-Hanford, Adventist Health Feather River, Adventist Health Glendale, Adventist Health Portland, Affordable Medicines Facility-malaria, Africa Centres for Disease Control and Prevention, Africa Fighting Malaria, African Health Economics and Policy Association, African Malaria Network Trust, African Nutrition Leadership Programme, Against Malaria Foundation, Aging-associated diseases, Air pollution, Air pollution and traffic congestion in Tehran, Air pollution forecasting, Air pollution in Hong Kong, Air pollution in Macau, Air pollution measurement, Air Quality Health Index (Canada), Airport malaria, Alberta Health Insurance Act (1935), Alcohol and health, Alcoholic liver disease, Alcoholic lung disease, Alexander disease, Alliance for Health Policy and Systems Research, Alliance for Healthy Cities, AllianceHealth Durant, Alzheimer's disease biomarkers, Alzheimer's Disease Cooperative Study, Alzheimer's disease in the media, Alzheimer's Disease Neuroimaging Initiative, Amazon Malaria Initiative, American Association for the Study and Prevention of Infant Mortality, American Association of Public Health Dentistry, American Association of Public Health Physicians, American College of Epidemiology, American Journal of Epidemiology, American Public Health Association, American School Health Association, American Sexual Health Association, American Society for Nutrition, American Society for Parenteral and Enteral Nutrition, AMITA Health, Anaerobic infection, Andersen healthcare utilization model, Animal nutrition, AnMed Health Women's & Children's Hospital, Annals of Epidemiology, Anti-AQP4 disease, Anti-IgLON5 disease, Antidiarrhoeal, Antimalarial medication, Apparent infection rate, Applications of sensitivity analysis in epidemiology, Asia Pacific Leaders Malaria Alliance, Aspiration pneumonia, Aspirin-exacerbated respiratory disease, Association for Community Health Improvement, Association of Medical Microbiology and Infectious Disease Canada, Association of Public Health Laboratories, Atrium Health, Atrium Health Cabarrus, Atrium Health Mercy, Atrium Health Pineville, Atrium Health Union, Atrium Health University City, Atrium Health Wake Forest Baptist, Atypical pneumonia, Australian Longitudinal Study on Women's Health, Australian Measles Control Campaign, Autoimmune disease, Autoimmune disease in women, Autoimmune inner ear disease, Autoimmune skin diseases in dogs, Autoinflammatory diseases, Autosomal dominant polycystic kidney disease, Autosomal recessive polycystic kidney disease, Avalere Health, Bacterial pneumonia, Balwadi Nutrition Programme, Bamrasnaradura Infectious Diseases Institute, Bandim Health Project, Bangladesh Institute of Child and Mother Health, Bangladesh Institute of Child Health, Bangladesh National Nutrition Council, Baptist Health System, Batten disease, Bayfront Health Punta Gorda, Bayfront Health St. Petersburg, Behavior change (public health), Belgian Health Care Knowledge Centre, BENTA disease, Bill of health, Bills of mortality, Binswanger's disease, Biochemistry of Alzheimer's disease, Biologically based mental illness, Biphasic disease, Black Maternal Health Caucus, Black Women's Health Study,

Blackheart (plant disease), Blood-borne disease, Bloodstream infections, Blount's disease, Bluetongue disease, Bombay plague epidemic, Bone health, Boroughs of Montreal during the COVID-19 pandemic, Brazilian Health Regulatory Agency, British Nutrition Foundation, Bronchopneumonia, Brookwood Baptist Health, Busselton Health Study, Caerphilly Heart Disease Study, Calcium pyrophosphate dihydrate crystal deposition disease, California Center for Public Health Advocacy, Canadian Public Health Association, Canadian Society for Epidemiology and Biostatistics, Canavan disease, Cancer Epidemiology (journal), Cancer Epidemiology, Biomarkers & Prevention, Canine vector-borne disease, Capitation (healthcare), Cardiovascular disease, Caribbean Public Health Agency, Carlos III Health Institute, Caroli disease, Carolinas College of Health Sciences, Carondelet Health Network, Carrion's disease, Case management (mental health), Castleman disease, Cat-scratch disease, Catheter-associated urinary tract infection, Causes of mental disorders, Center for Global Infectious Disease Research, Center for Infectious Disease Research and Policy, Centre for Health Protection, Centre for History in Public Health, London School of Hygiene and Tropical Medicine, Cerebrovascular disease, Chagas disease, CHAI disease, Child health in Uganda, Child mortality, Childhood chronic illness, Children's Healthcare of Atlanta, Children's Healthcare of Atlanta at Egleston, Children's Healthcare of Atlanta at Hughes Spalding, Children's Healthcare of Atlanta at Scottish Rite, Children's right to adequate nutrition in New Zealand, Chinese Center for Disease Control and Prevention, Chinese Classification of Mental Disorders, Chlamydia pneumoniae, Chloroquine and hydroxychloroquine during the COVID-19 pandemic, Choosing Healthplans All Together, Chronic kidney disease, Chronic Lyme disease, Chronic obstructive pulmonary disease, Cinematography in healthcare, Circle of Health International, Classification of mental disorders, Classification of pneumonia, Climate change and infectious diseases, Clinical epidemiology, Clinical Epidemiology (journal), Clinical Health Promotion, Clinical nutrition, Clinton health care plan of 1993, Clostridioides difficile infection, CNS demyelinating autoimmune diseases, Coalition for Epidemic Preparedness Innovations, Cognitive epidemiology, Cohorts for Heart and Aging Research in Genomic Epidemiology, Coinfection, Cold agglutinin disease, Colorado Department of Health Care Policy and Financing, Commission on Health Research for Development, Commission on the Accreditation of Healthcare Management Education, Common disease-common variant, CommonSpirit Health, Community Dentistry and Oral Epidemiology, Community health, Community Health Clubs in Africa, Community Health Systems, Community-acquired pneumonia, Comorbidity, Comparison of the healthcare systems in Canada and the United States, Compartmental models in epidemiology, Compression of morbidity, Computational epidemiology, Cone Health, Cone Health Behavioral Health Hospital, Cone Health Women's Hospital, Conflict epidemiology, Congenital cytomegalovirus infection, Congenital malaria, Connecting Organizations for Regional Disease Surveillance, Consortium of Universities for Global Health, Contagious bovine pleuropneumonia, Contagious disease, Convention on Long-Range Transboundary Air Pollution, Core Cities Health Improvement Collaborative, Corn stunt disease, Coronary artery disease, Coronavirus diseases, Coughs and sneezes spread diseases, Council on Education for Public Health, COVID-19 pandemic, COVID-19 pandemic by country and territory, COVID-19, pandemic in Abkhazia, COVID-19 pandemic in Afghanistan, COVID-19 pandemic in Africa, COVID-19 pandemic in Akrotiri and Dhekelia, COVID-19 pandemic in Albania, COVID-19 pandemic in Algeria, COVID-19 pandemic in American Samoa, COVID-19 pandemic in Andorra, COVID-19 pandemic in Angola, COVID-19 pandemic in Antigua and Barbuda, COVID-19 pandemic in Argentina, COVID-19 pandemic in Armenia, COVID-19 pandemic in Australia, COVID-19 pandemic in Austria, COVID-19 pandemic in Azerbaijan, COVID-19 pandemic in Bahrain, COVID-19 pandemic in Bangladesh, COVID-19 pandemic in Barbados, COVID-19 pandemic in Belarus, COVID-19 pandemic in Belgium, COVID-19 pandemic in Belize, COVID-19 pandemic in Benin, COVID-19 pandemic in Bhutan, COVID-19 pandemic in Bolivia, COVID-19 pandemic in Bosnia and Herzegovina, COVID-19 pandemic in Botswana, COVID-19 pandemic in Brazil, COVID-19 pandemic in Brunei, COVID-19 pandemic in Bulgaria, COVID-19 pandemic in Burkina Faso, COVID-19 pandemic in Burundi, COVID-19 pandemic in Cambodia, COVID-19 pandemic in Cameroon, COVID-19 pandemic in Canada, COVID-19 pandemic in Cape Verde, COVID-19 pandemic in Ceuta, COVID-19 pandemic in Chad, COVID-19 pandemic in Chile, COVID-19 pandemic in China, COVID-19 pandemic in Christmas Island, COVID-19 pandemic in Colombia, COVID-19 pandemic in Costa Rica, COVID-19 pandemic in Croatia, COVID-19 pandemic in Cuba, COVID-19 pandemic in Cyprus, COVID-19 pandemic in Denmark, COVID-19 pandemic in Djibouti, COVID-19 pandemic in Dominica, COVID-19 pandemic in East Timor, COVID-19 pandemic in Easter Island, COVID-19 pandemic in Ecuador, COVID-19 pandemic in Egypt, COVID-19 pandemic in El Salvador, COVID-19 pandemic in Equatorial Guinea, COVID-19 pandemic in Eritrea, COVID-19 pandemic in Estonia, COVID-19 pandemic in Eswatini, COVID-19 pandemic in Ethiopia, COVID-19 pandemic in Fiji, COVID-19 pandemic in Finland, COVID-19 pandemic in France, COVID-19 pandemic in French Polynesia, COVID-19 pandemic in Gabon, COVID-19 pandemic in Georgia (country), COVID-19 pandemic in Germany, COVID-19 pandemic in Ghana, COVID-19 pandemic in Greece, COVID-19 pandemic in Greenland, COVID-19 pandemic in Grenada, COVID-19 pandemic in Guatemala, COVID-19 pandemic in Guernsey, COVID-19 pandemic in Guinea, COVID-19 pandemic in Guinea-Bissau, COVID-19 pandemic in Guyana, COVID-19 pandemic in Haiti, COVID-19 pandemic in Honduras, COVID-19 pandemic in Hungary, COVID-19 pandemic in Iceland, COVID-19 pandemic in India, COVID-19 pandemic in Indonesia, COVID-19 pandemic in Iran, COVID-19 pandemic in Iraq, COVID-19 pandemic in Israel, COVID-19 pandemic in Italy, COVID-19 pandemic in Ivory Coast, COVID-19 pandemic in Jamaica, COVID-19 pandemic in Japan, COVID-19 pandemic in Jersey, COVID-19 pandemic in Jordan, COVID-19 pandemic in Kazakhstan, COVID-19 pandemic in Kenya, COVID-19 pandemic in Kiribati, COVID-19 pandemic in Kosovo, COVID-19 pandemic in Kuwait, COVID-19 pandemic in Kyrgyzstan, COVID-19 pandemic in Lesotho, COVID-19 pandemic in Liberia, COVID-19 pandemic in Libya, COVID-19 pandemic in Madagascar, COVID-19 pandemic in mainland China, COVID-19 pandemic in Malawi, COVID-19 pandemic in Maldives, COVID-19 pandemic in Mali, COVID-19 pandemic in Mauritania, COVID-19 pandemic in Mauritius, COVID-19 pandemic in

Mayotte, COVID-19 pandemic in Melilla, COVID-19 pandemic in Morocco, COVID-19 pandemic in Mozambique, COVID-19 pandemic in Namibia, COVID-19 pandemic in New South Wales, COVID-19 pandemic in Niger, COVID-19 pandemic in Nigeria, COVID-19 pandemic in Norfolk Island, COVID-19 pandemic in Normandy, COVID-19 pandemic in Northern Cyprus, COVID-19 pandemic in Northern Ireland, COVID-19 pandemic in Puntland, COVID-19 pandemic in Queensland, COVID-19 pandemic in Rwanda, COVID-19 pandemic in Senegal, COVID-19 pandemic in Seychelles, COVID-19 pandemic in Sierra Leone, COVID-19 pandemic in Somalia, COVID-19 pandemic in Somaliland, COVID-19 pandemic in South Africa, COVID-19 pandemic in South Australia, COVID-19 pandemic in South Ossetia, COVID-19 pandemic in South Sudan, COVID-19 pandemic in Sudan, COVID-19 pandemic in Tanzania, COVID-19 pandemic in Tasmania, COVID-19 pandemic in the Australian Capital Territory, COVID-19 pandemic in the Bahamas, COVID-19 pandemic in the Canary Islands, COVID-19 pandemic in the Central African Republic, COVID-19 pandemic in the Cocos (Keeling) Islands, COVID-19 pandemic in the Comoros, COVID-19 pandemic in the Cook Islands, COVID-19 pandemic in the Czech Republic, COVID-19 pandemic in the Democratic Republic of the Congo, COVID-19 pandemic in the Dominican Republic, COVID-19 pandemic in the Donetsk People's Republic, COVID-19 pandemic in the Faroe Islands, COVID-19 pandemic in the Federated States of Micronesia, COVID-19 pandemic in the Gambia, COVID-19 pandemic in the Guantanamo Bay Naval Base, COVID-19 pandemic in the Isle of Man, COVID-19 pandemic in the Kurdistan Region, COVID-19 pandemic in the Northern Territory, COVID-19 pandemic in the Republic of Artsakh, COVID-19 pandemic in the Republic of Ireland, COVID-19 pandemic in the Republic of the Congo, COVID-19 pandemic in the Sahrawi Arab Democratic Republic, COVID-19 pandemic in Togo, COVID-19 pandemic in Tunisia, COVID-19 pandemic in Uganda, COVID-19 pandemic in Victoria, COVID-19 pandemic in Western Australia, COVID-19 pandemic in Western Sahara, COVID-19 pandemic in Zambia, COVID-19 pandemic in Zimbabwe, COVID-19 pandemic on Charles de Gaulle, COVID-19 pandemic on Diamond Princess, Creativity and mental health, Creutzfeldt-Jakob Disease Surveillance System, Creutzfeldt–Jakob disease, Critical illness insurance, Crohn's disease, Cryptogenic organizing pneumonia, Cytomegaloviral disease, Cytomegalovirus infection, Dance and health, Deen Dayal Mobile Health Clinic, Degenerative disease, Dementia and Alzheimer's disease in Australia, Dengue pandemic in Sri Lanka, Depression of Alzheimer disease, Desquamative interstitial pneumonia, Diabetic foot infection, Diagnosis of malaria, Diagnostic and Statistical Manual of Mental Disorders, Diarrhoea, Dignity Health, Dignity Health St. Joseph's Hospital and Medical Center, Director-General of the World Health Organization, Directorate of Health, Disability and women's health, Discovery of disease-causing pathogens, Disease, Disease burden, Disease cluster, Disease diffusion mapping, Disease ecology, Disease management (health), Disease outbreak, Disease resistance, Disease surveillance, Disease vector, Disease X, Disease-modifying osteoarthritis drug, Diseases, Diseases of abnormal polymerization, Diseases of despair, Diseases of poverty, Disneyland measles outbreak, Disseminated disease, Doctor of Public Health, Dole Nutrition Institute, Drugs for Neglected Diseases Initiative, Dukes' disease, Dunedin Multidisciplinary Health and Development Study, Dust pneumonia, E-epidemiology, Early-onset Alzheimer's disease, Ecological health, Economic epidemiology, Economic impact of the COVID-19 pandemic in the Republic of Ireland, Ecosystem health, Ehrlichiosis ewingii infection, EMBRACE Healthcare Reform Plan, Emerging infectious disease, Emerging Themes in Epidemiology, Endemic (epidemiology), Endogenous infection, Environmental disease, Environmental epidemiology, Environmental health, Environmental health ethics, Environmental health officer, Environmental health policy, Eosinophilic pneumonia, Epidemic, Epidemic curve, Epidemic Diseases Act, 1897, Epidemic Intelligence Service, Epidemic models on lattices, Epidemic polyarthritis, Epidemic typhus, Epidemics (journal), Epidemiology, Epidemiology (journal), Epidemiology and Infection, Epidemiology and Psychiatric Sciences, Epidemiology data for low-linear energy transfer radiation, Epidemiology in Country Practice, Epidemiology in Relation to Air Travel, Epidemiology of asthma, Epidemiology of attention deficit hyperactive disorder, Epidemiology of bed bugs, Epidemiology of binge drinking, Epidemiology of breast cancer, Epidemiology of cancer, Epidemiology of chikungunya, Epidemiology of child psychiatric disorders, Epidemiology of childhood obesity, Epidemiology of depression, Epidemiology of diabetes, Epidemiology of malnutrition, Epidemiology of measles, Epidemiology of metabolic syndrome, Epidemiology of pneumonia, Epidemiology of schizophrenia, Epidemiology of suicide, Epidemiology of syphilis, Eradication of infectious diseases, Escape Fire: The Fight to Rescue American Healthcare, EuroHealthNet, European Centre for Disease Prevention and Control, European Health Examination Survey, European Health Forum Gastein, European Journal of Epidemiology, European Parliament Committee on the Environment, Public Health and Food Safety, European Prospective Investigation into Cancer and Nutrition, European Public Health Alliance, European Public Health Association, European Society for Clinical Nutrition and Metabolism, European Society for Paediatric Infectious Diseases, European Working Group for Legionella Infections, Evaluation & the Health Professions, Evolution of Infectious Disease, Face masks during the COVID-19 pandemic, Fair Share Health Care Act, Febrile infection-related epilepsy syndrome, Federal aid during the COVID-19 pandemic in Canada, Federal Service for Surveillance in Healthcare, Federation of European Nutrition Societies, Fifth disease, Finnish Institute for Health and Welfare, Fire breather's pneumonia, First Nations nutrition experiments, Focus of infection, Foodborne illness, Foot-and-mouth disease, Free-market healthcare, Fungal infection, Fungal pneumonia, Gamaleya Research Institute of Epidemiology and Microbiology, Gastrointestinal disease, Gender disparities in health, General Health Questionnaire, Genetic epidemiology, Genetic Epidemiology (journal), George Institute for Global Health, Germ theory of disease, German government response to the COVID-19 pandemic, Ghana Infectious Disease Centre, Ghanaian government response to the COVID-19 pandemic, GIS and public health, Global Acute Malnutrition, Global Alliance for Improved Nutrition, Global Burden of Disease Study, Global Climate and Health Alliance, Global Coalition Against Pneumonia, Global Forum for Health Research, Global health, Global Health Security Index, Global Health Security Initiative, Global Infectious Disease Epidemiology Network, Global Malaria Action Plan, Global mental health, Global

Network for Neglected Tropical Diseases, Global Public Health Intelligence Network, Globalization and disease, Glossary of the COVID-19 pandemic, Goal-oriented health care, Gold Coast Influenza Epidemic, Graduate School of Health Economics and Management, Graves' disease, Groningen epidemic, Grossman model of health demand, Group B streptococcal infection, Gulf War Health Research Reform Act of 2014, Handbook of Religion and Health, HCA Healthcare, Health, Health (Preservation and Protection and other Emergency Measures in the Public Interest) Act 2020, Health 21, Health Action International, Health administration, Health advocacy, Health Alliance International, Health and Social Protection Federation, Health and wealth, Health belief model, Health Canada, Health care, Health Care Compact, Health care efficiency, Health care efficiency measures, Health care finance in the United States, Health Care for Women International, Health care prices in the United States, Health care ratings, Health care rationing, Health care reforms proposed during the Obama administration, Health care time and motion study, Health Code, Health consequences of the Deepwater Horizon oil spill, Health crisis, Health Data Insight, Health departments in the United States, Health disaster, Health Dynamics Inventory, Health ecology, Health economics, Health economics (Germany), Health Economics, Policy and Law, Health education, Health Education & Behavior, Health effect, Health Effects Institute, Health effects of caffeine, Health effects of chocolate, Health effects of coffee, Health effects of green tea, Health effects of salt, Health effects of sugar, Health effects of sugary drinks, Health effects of tea, Health effects of wine, Health effects of wood smoke, Health Emergencies Programme (WHO), Health equity, Health fair, Health For All, Health geography, Health humanities, Health impact assessment, Health in Poland, Health Information National Trends Survey, Health information on Wikipedia, Health insurance, Health insurance coverage in the United States, Health insurance marketplace, Health literacy, Health marketing, Health measures during the construction of the Panama Canal, Health of Hillary Clinton, Health of Towns Association, Health policy, Health policy and management, Health policy in Bangladesh, Health politics, Health promotion, Health promotion in higher education, Health Promotion International, Health Promotion Practice, Health Protection Surveillance Centre, Health risk assessment, Health Sciences Online, Health security, Health Security Express, Health services research, Health Services Workers' Union, Health surveillance, Health system, Health Threat Unit, Health Utilities Index, Health-related embarrassment, Healthcare number 1450, Healthcare rationing in the United States, Healthcare reform debate in the United States, Healthcare reform in the United States, Healthcare transport, HealthCare.gov, Healthlink Worldwide, HealthOne, Healthy city, Healthy community design, Healthy development measurement tool, Healthy Life Years, HealthyWomen, Heat illness, High-deductible health plan, High-dependency unit (mental health), Hispanic Health Council, History of health care reform in the United States, History of malaria, History of mental disorders, Holozoic nutrition, Hookworm infection, Hospital-acquired infection, Hospital-acquired pneumonia, Household air pollution, How to Have Sex in an Epidemic, How to Prevent the Next Pandemic, Human genetic resistance to malaria, Human Heredity and Health in Africa, Human nutrition, Human papillomavirus infection, Hypertensive disease of pregnancy, Idiopathic disease, Idiopathic interstitial pneumonia, Idiopathic multicentric Castleman disease, Idiopathic orbital inflammatory disease, Idiopathic pneumonia syndrome, IgG4-related disease, IgG4-related ophthalmic disease, IgG4-related skin disease, Illinois Health Benefits Exchange, Imagine No Malaria, Impact of the COVID-19 pandemic on children, Impact of the COVID-19 pandemic on education in Ghana, Impact of the COVID-19 pandemic on education in the Republic of Ireland, Impact of the COVID-19 pandemic on Gaelic games, Impact of the COVID-19 pandemic on human rights in Argentina, Independent Panel for Pandemic Preparedness and Response, Indian government response to the COVID-19 pandemic, Indian migrant workers during the COVID-19 pandemic, Indian state government responses to the COVID-19 pandemic, Inequality in disease, Infant mortality, Infection, Infection Control Society of Pakistan, Infection prevention and control, Infection rate, Infections associated with diseases, Infectious Disease (Notification) Act 1889, Infectious Disease Pharmacokinetics Laboratory, Infectious diseases, Infectious diseases (medical specialty), Infectious Diseases, Institute, Infectious Diseases Society of America, Inflammatory bowel disease, Inflammatory demyelinating diseases of the central nervous system, Influenza pandemic, Influx of disease in the Caribbean, Institute for Health Metrics and Evaluation, Institute of Public Health (Bangladesh), Integrated Disease Surveillance Programme, Intentional contagion of infection, Interdisciplinary Association for Population Health Science, International Association of National Public Health Institutes, International Classification of Diseases, International Conference on Emerging Infectious Diseases, International Health Exhibition, International Journal of Epidemiology, International Journal of Men's Health, International Lyme and Associated Diseases Society, International Men's Health Week, International Network of Health Promoting Hospitals and Health Services, International Partnership on Avian and Pandemic Influenza, International reactions to the COVID-19 pandemic in Italy, International Society for Environmental Epidemiology, International Society for Infectious Diseases in Obstetrics and Gynaecology, International Society for Pharmacoepidemiology, International Union of Air Pollution Prevention and Environmental Protection Associations, International Volcanic Health Hazard Network, Intestinal infectious diseases, Intradialytic parenteral nutrition, Irish Nutrition and Dietetic Institute, Iron Triangle of Health Care, Isolation (health care), James C. Robinson (health economist), James Thornton (health economist), Jembrana disease, Johns Hopkins Center for Health Security, Joondalup Family Health Study, Journal of Clinical Epidemiology, Journal of Epidemiology, Journal of Epidemiology and Biostatistics, Journal of Epidemiology and Community Health, Journal of Exposure Science and Environmental Epidemiology, Journal of Health Economics, Journal of Urban Health, Kawasaki disease, Kettering Health, Kettering Health Dayton, Kettering Health Main Campus, Kettering Health Washington Township, Kids for World Health, Korea Disease Control and Prevention Agency, Krabbe disease, Kyasanur Forest disease, Laboratory-acquired infection, Landscape epidemiology, Latent period (epidemiology), Legacy Health, Legionnaires' disease, Lenox Health Greenwich Village, Leveraging Agriculture for Improving Nutrition and Health, Lifestyle disease, Lipid pneumonia, List of AdventHealth hospitals, List of autoimmune diseases, List of countries by total health expenditure per capita, List of

diseases eliminated from the United States, List of epidemics, List of foodborne illness outbreaks, List of foodborne illness outbreaks by death toll, List of foodborne illness outbreaks in the United States, List of infections of the central nervous system, List of infectious diseases, List of infectious diseases causing flu-like syndrome, List of insect-borne diseases, List of Legionnaires' disease outbreaks, List of mental disorders, List of national public health agencies, List of people with motor neuron disease, List of pneumonia deaths, List of sexually transmitted infections by prevalence, List of species named after the COVID-19 pandemic, List of types of malnutrition, Liver disease, Liverpool Neurological Infectious Diseases Course, Lobar pneumonia, Localized disease, London Declaration on Neglected Tropical Diseases, Lower Mississippi Valley yellow fever epidemic of 1878, Lower respiratory tract infection, Lutheran Health Network, Lyme disease, Lyme Disease Awareness Month, Lyme disease microbiology, Lymphocytic interstitial pneumonia, Lysosomal storage disease, Madras motor neuron disease, Malaria, Malaria and the Caribbean, Malaria antigen detection tests, Malaria Atlas Project, Malaria Consortium, Malaria Control Project, Malaria culture, Malaria Day in the Americas, Malaria Eradication Scientific Alliance, Malaria in Benin, Malaria in Madagascar, Malaria in Palestine, Malaria Journal, Malaria No More, Malaria No More UK, Malaria Policy Advisory Committee, Malaria prophylaxis, Malaria vaccine, Malarial nephropathy, Malnutrition, Malnutrition in India, Malnutrition in Kerala, Malnutrition in Peru, Malnutrition in South Africa, Malnutrition in Zimbabwe, Management of Crohn's disease, Managerial epidemiology, Marburg virus disease, Mass psychogenic illness, Massachusetts smallpox epidemic, Maternal and child health in Tanzania, Maternal health, Maternal health in Angola, Maternal health in Ethiopia, Maternal health in Rwanda, Maternal health in Uganda, Maternal healthcare in Texas, Maternal oral health, Mayaro virus disease, Measles, Measles & Rubella Initiative, Measles hemagglutinin, Measles morbillivirus, Measles resurgence in the United States, Measles vaccine, Measles: A Dangerous Illness, Medical and Health Workers' Union of Nigeria, Medical officer of health, Medicines for Malaria Venture, Mekong Basin Disease Surveillance, Melanie's Marvelous Measles, Men's health, Men's health in Australia, Meningococcal disease, Mental disorder, Mental disorders and gender, Mental health, Mental health consumer, Mental health first aid, Mental health literacy, Mental illness, Mental illness denial, Mental illness in ancient Greece, Mental illness in ancient Rome, Mental illness portrayed in media, Milk borne diseases, Ministry of Health and Welfare (South Korea), Ministry of Health Promotion and Sport (Ontario), Miscarriage and mental illness, Mission Health System, Mississippi Health Project, Mitochondrial disease, Mobile source air pollution, MOG antibody disease, Morbidity and Mortality Weekly Report, Mosquito-borne disease, Mosquito-malaria theory, Motor neuron diseases, Motor Neurone Disease Association, Mount Sinai Health System, MRC Human Nutrition Research, Muesli belt malnutrition, Multifactorial disease, Multimorbidity, Music therapy for Alzheimer's disease, Mycobacterium avium-intracellulare infection, Mycoplasma hominis infection, Mycoplasma ovipneumoniae, Mycoplasma pneumonia, National Aboriginal Health Organization, National Air Pollution Symposium, National Association for Public Health Policy, National Association of County and City Health Officials, National Center for Disease Control and Public Health (Georgia), National Centre for Disease Control, National Centre for Infectious Diseases, National Children's Center for Rural and Agricultural Health and Safety, National Comorbidity Survey, National Emerging Infectious Diseases Laboratories, National Foundation for Infectious Diseases, National Health Fund, National health insurance, National Health Insurance Fund, National Health Interview Survey, National Health Mission, National Health Policy, National Institute for Communicable Diseases, National Institute for Health and Care Excellence, National Institute for Health and Disability Insurance, National Institute for Public Health and the Environment, National Institute of Health, Islamabad, National Institute of Malaria Research, National Institute of Nutrition, Hyderabad, National Institute of Public Health of Japan, National Malaria Eradication Program, National Perinatal Epidemiology Unit, National Prostate Health Month, National Public Health Emergency Team (2020), National Public Health Organization (Greece), National responses to the COVID-19 pandemic in Africa, National School of Public Health (Spain), Native American disease and epidemics, Native American Women's Health Education Resource Center, Navicent Health Baldwin, Necrotizing pneumonia, Neglected tropical diseases, Neglected tropical diseases in India, Neonatal infection, Network for Capacity Development in Nutrition, Neuroepidemiology (journal), Nevada Health Link, New Mexico Health Insurance Exchange, NewYork-Presbyterian Healthcare System, Nigel Edwards (health), Nigeria Centre for Disease Control, Nigerian government response to the COVID-19 pandemic, NINCDS-ADRDA Alzheimer's Criteria, Nipah virus infection, Noma (disease), Non-alcoholic fatty liver disease, Non-communicable disease, Non-communicable diseases, Non-pharmaceutical intervention (epidemiology), Non-specific interstitial pneumonia, Northwell Health, Norwegian Institute of Public Health, Notifiable disease, Notifiable diseases in Sweden, Notifiable diseases in the United Kingdom, Novant Health, Novant Health Forsyth Medical Center, Nurses' Health Study, Nutrition, Nutrition analysis, Nutrition and cognition, Nutrition and Education International, Nutrition and pregnancy, Nutrition education, Nutrition Foundation of the Philippines, Nutritional epidemiology, Nutritional genomics, Nutritional neuroscience, Nutritional rating systems, Nutritional science, NutritionDay, Nutritionist, Obstacles to receiving mental health services among African American youth, Occult pneumonia, Occupational exposure to Lyme disease, Occupational safety and health, Office for Health Improvement and Disparities, Office on Women's Health, One Health, Opportunistic infection, Oregon Medicaid health experiment, OSF HealthCare, Ottawa Charter for Health Promotion, Outline of air pollution dispersion, Outline of public health, Outline of the COVID-19 pandemic, Overwhelming post-splenectomy infection, Paediatric and Perinatal Epidemiology, Paget's disease of bone, Pandemic, Pandemic fatigue, Pandemic predictions and preparations prior to the COVID-19 pandemic, Pandemic Preparedness and Response Act, Pandemic prevention, Pandemic Severity Assessment Framework, Pandemic severity index, Papaya Bunchy Top Disease, Parasitic disease, Parasitic pneumonia, Parenteral nutrition, Parkinson's disease, Pathogens and Global Health, Pay for performance (healthcare), Pelvic inflammatory disease, Pervasive developmental disorder, Pervasive developmental disorder not otherwise specified, Peyronie's disease, Pinta (disease), Pinworm infection, Plague (disease), Plague epidemics in Malta, Plant nutrition,

Pneumococcal infection, Pneumococcal pneumonia, Pneumocystis pneumonia, Pneumonia, Pneumonia (non-human), Pneumonia jacket, Pneumonia of unknown etiology (PUE) surveillance system, Pneumonia severity index, Pogosta disease, Population health, Population health policies and interventions, Population, health, and the environment, Portal:Pandemics, Postorgasmic illness syndrome, Pott disease, Pravastatin or atorvastatin evaluation and infection therapy - thrombolysis in myocardial infarction 22, Prebiotic (nutrition), Pregnancy-associated malaria, President's Malaria Initiative, Prevalence of mental disorders, Prevention of mental disorders, Preventive nutrition, Price-Pottenger Nutrition Foundation, Primary Health Centre (India), Prime Healthcare Services, Prison healthcare, Program for Jewish Genetic Health, Progressive disease, Providence Health & Services, Providence St. Joseph Health, Psychiatric epidemiology, Psychogenic disease, Public health, Public Health Agency of Canada, Public Health Agency of Sweden, Public Health England, Public health insurance option, Public health observatory, Public health policy, Public health problems in the Aral Sea region, Public Health Scotland, Public health system in India, Public Health Wales, Publicly funded health care, Pullorum disease, Qapqal disease, Quantum suicide and immortality, Race and health, Race and maternal health in the United States, RAND Health Insurance Experiment, Rare disease, Reactive airway disease, Real-time outbreak and disease surveillance, Regional Forum on Environment and Health in Southeast and East Asian Countries, Reproductive health care for incarcerated women in the United States, Reproductive system disease, Respiratory disease, Respiratory tract infection, Rheumatoid disease of the spine, Right to health, Salt and cardiovascular disease, Samaritan Health Services, Saprotrophic nutrition, SARS, SARS-CoV-1, SARS-CoV-2, SARS-CoV-2 Beta variant, SARS-CoV-2 Delta variant, SARS-CoV-2 Eta variant, SARS-CoV-2 Gamma variant, SARS-CoV-2 Kappa variant, SARS-CoV-2 Mu variant, SARS-CoV-2 Omicron variant, Scandinavian Journal of Work, Environment & Health, School-based health centers, Science diplomacy and pandemics, Second plague pandemic, Self-rated health, Sentara Healthcare, Serious mental illness, Services for mental disorders, Seventh cholera pandemic, Sexual and reproductive health, Sexual and Reproductive Health Matters, Sexual health clinic, Sexually transmitted infection, Single-payer healthcare, Sissel v. United States Department of Health & Human Services, Skin and skin structure infection, Skin infection, Smallpox epidemic, Social Psychiatry and Psychiatric Epidemiology, Society for Family Health Nigeria, Society of Infectious Diseases Pharmacists, Socioeconomic status and mental health, South African Malaria Initiative, South Texas Center for Emerging Infectious Diseases, Southern tick-associated rash illness, Spanish National Health System, Spatial and Spatio-temporal Epidemiology, Spatial epidemiology, Specific replant disease, St. Patrick Hospital and Health Sciences Center, St. Vincent's Health System, Stateville Penitentiary Malaria Study, Statistics of the COVID-19 pandemic in Argentina, Statistics of the COVID-19 pandemic in Australia, Statistics of the COVID-19 pandemic in Bangladesh, Statistics of the COVID-19 pandemic in Brazil, Statistics of the COVID-19 pandemic in Chile, Statistics of the COVID-19 pandemic in Germany, Statistics of the COVID-19 pandemic in India, Statistics of the COVID-19 pandemic in Indonesia, Statistics of the COVID-19 pandemic in Italy, Statistics of the COVID-19 pandemic in Japan, Statistics of the COVID-19 pandemic in Tamil Nadu, STOP Foodborne Illness, Strengthening the reporting of observational studies in epidemiology, Streptococcus pneumoniae, Study of Health in Pomerania, Suicide epidemic, Superinfection, Susceptibility and severity of infections in pregnancy, Sutter Health, Sweating sickness epidemics, Swedish Association of Health Professionals, Systemic disease, Taiwan Centers for Disease Control, Tanganyika laughter epidemic, Target Malaria, Tay–Sachs disease, Template:Ascension Health, Template:Epidemic-stub, Template:Eradication of infectious disease, Template:Infectious-disease-stub, Template:Malaria, Template:Mental disorders, Template:Plant nutrition, Template:Vertically transmitted infection, Template:Women's health, Tenet Healthcare, Texas Health Huguley Hospital Fort Worth South, The European Journal of Health Economics, The Global Fund to Fight AIDS, Tuberculosis and Malaria, The Journal of Mental Health Policy and Economics, The Medical Center, Navicent Health, The Office of Health Economics, Theiler's disease, Tick-borne disease, Tick-Borne Disease Alliance, Timeline of peptic ulcer disease and Helicobacter pylori, Timeline of the COVID-19 pandemic in Afghanistan, Timeline of the COVID-19 pandemic in Argentina, Timeline of the COVID-19 pandemic in Australia, Timeline of the COVID-19 pandemic in Australia (2022), Timeline of the COVID-19 pandemic in Bangladesh, Timeline of the COVID-19 pandemic in Belarus, Timeline of the COVID-19 pandemic in Belarus (2020), Timeline of the COVID-19 pandemic in Belarus (2021), Timeline of the COVID-19 pandemic in Belarus (2022), Timeline of the COVID-19 pandemic in Brazil, Timeline of the COVID-19 pandemic in Canada, Timeline of the COVID-19 pandemic in Colombia, Timeline of the COVID-19 pandemic in Croatia, Timeline of the COVID-19 pandemic in Fiji, Timeline of the COVID-19 pandemic in Ghana, Timeline of the COVID-19 pandemic in India, Timeline of the COVID-19 pandemic in India (2021), Timeline of the COVID-19 pandemic in Indonesia, Timeline of the COVID-19 pandemic in Indonesia (2020), Timeline of the COVID-19 pandemic in Indonesia (2021), Timeline of the COVID-19 pandemic in Indonesia (2022), Timeline of the COVID-19 pandemic in Italy, Timeline of the COVID-19 pandemic in Japan, Timeline of the COVID-19 pandemic in Malaysia, Timeline of the COVID-19 pandemic in Malta, Timeline of the COVID-19 pandemic in Mexico, Timeline of the COVID-19 pandemic in Nepal, Timeline of the COVID-19 pandemic in New Zealand, Timeline of the COVID-19 pandemic in Nigeria, Timeline of the COVID-19 pandemic in Pakistan, Timeline of the COVID-19 pandemic in Romania, Timeline of the COVID-19 pandemic in Russia, Timeline of the COVID-19 pandemic in Serbia, Timeline of the COVID-19 pandemic in Singapore, Timeline of the COVID-19 pandemic in South Africa, Timeline of the COVID-19 pandemic in Spain, Timeline of the COVID-19 pandemic in Sweden, Timeline of the COVID-19 pandemic in Thailand, Timeline of the COVID-19 pandemic in the Philippines, Timeline of the COVID-19 pandemic in the Republic of Ireland, Timeline of the COVID-19 pandemic in the Republic of Ireland (2021), Timeline of the COVID-19 pandemic in the Republic of Ireland (2022), Timeline of the COVID-19 pandemic in the United Kingdom, Timeline of the COVID-19 pandemic in the United States, Timeline of the COVID-19 pandemic in Trinidad and Tobago, Timeline of the COVID-19 pandemic in Turkey, Timeline of the COVID-19 pandemic in Uruguay, Timeline of the COVID-19 pandemic in Vietnam,

Timeline of the SARS-CoV-2 Omicron variant, Top dying disease, Trauma model of mental disorders, Treatment of mental disorders, Tropical disease, Typhus epidemic in Goose Village, Montreal, UCLA Health, UCLA Health Training Center, UCSC Malaria Genome Browser, UK Health Alliance on Climate Change, UK Health Security Agency, Undernutrition, Undernutrition in children, Uni Health, Unicentric Castleman disease, UnityPoint Health, UnityPoint Health - Allen Hospital, Universal Declaration on the Eradication of Hunger and Malnutrition, University of Edinburgh School of Health in Social Science, Ureaplasma urealyticum infection, Use of technology in treatment of mental disorders, Usual interstitial pneumonia, Vaccine-preventable diseases, Value-based health care, Vanguard Health Systems, Variant Creutzfeldt–Jakob disease, Vegetarian nutrition, Venereal Disease Research Laboratory test, Ventilator-associated pneumonia, Vermont health care reform, Vertically transmitted infection, Very early onset inflammatory bowel disease, Victorian Health Promotion Foundation, Viral disease testing, Viral pneumonia, Virgin soil epidemic, Waterborne disease and climate change, Waterborne diseases, Weather and climate effects on Lyme disease exposure, WHO Hub for Pandemic and Epidemic Intelligence, Whole Health Action Management, Wilt disease, Women's health, Women's health movement in the United States, Working Environment (Air Pollution, Noise and Vibration) Convention, 1977, Workplace health promotion, World Chagas Disease Day, World Health Assembly, World Health Organization, World Health Organization Composite International Diagnostic Interview, World Health Organization response to the COVID-19 pandemic in Africa, World Malaria Day, World Pneumonia Day, Your Health Idaho

List of English Wikipedia articles used to track public engagement in the COVID-19 pandemic

Boroughs of Montreal during the COVID-19 pandemic, Chloroquine and hydroxychloroquine during the COVID-19 pandemic, Coronavirus diseases, COVID-19, COVID-19 and cancer, COVID-19 pandemic, COVID-19 pandemic by country and territory, COVID-19 pandemic in Abkhazia, COVID-19 pandemic in Afghanistan, COVID-19 pandemic in Africa, COVID-19 pandemic in Akrotiri and Dhekelia, COVID-19 pandemic in Albania, COVID-19 pandemic in Algeria, COVID-19 pandemic in American Samoa, COVID-19 pandemic in Andorra, COVID-19 pandemic in Angola, COVID-19 pandemic in Antigua and Barbuda, COVID-19 pandemic in Argentina, COVID-19 pandemic in Armenia, COVID-19 pandemic in Australia, COVID-19 pandemic in Austria, COVID-19 pandemic in Azerbaijan, COVID-19 pandemic in Bahrain, COVID-19 pandemic in Bangladesh, COVID-19 pandemic in Barbados, COVID-19 pandemic in Belarus, COVID-19 pandemic in Belgium, COVID-19 pandemic in Belize, COVID-19 pandemic in Benin, COVID-19 pandemic in Bhutan, COVID-19 pandemic in Bolivia, COVID-19 pandemic in Bosnia and Herzegovina, COVID-19 pandemic in Botswana, COVID-19 pandemic in Brazil, COVID-19 pandemic in Brunei, COVID-19 pandemic in Bulgaria, COVID-19 pandemic in Burkina Faso, COVID-19 pandemic in Burundi, COVID-19 pandemic in Cambodia, COVID-19 pandemic in Cameroon, COVID-19 pandemic in Canada, COVID-19 pandemic in Cape Verde, COVID-19 pandemic in Ceuta, COVID-19 pandemic in Chad, COVID-19 pandemic in Chile, COVID-19 pandemic in China, COVID-19 pandemic in Christmas Island, COVID-19 pandemic in Colombia, COVID-19 pandemic in Costa Rica, COVID-19 pandemic in Croatia, COVID-19 pandemic in Cuba, COVID-19 pandemic in Cyprus, COVID-19 pandemic in Denmark, COVID-19 pandemic in Djibouti, COVID-19 pandemic in Dominica, COVID-19 pandemic in East Timor, COVID-19 pandemic in Easter Island, COVID-19 pandemic in Ecuador, COVID-19 pandemic in Egypt, COVID-19 pandemic in El Salvador, COVID-19 pandemic in Equatorial Guinea, COVID-19 pandemic in Eritrea, COVID-19 pandemic in Estonia, COVID-19 pandemic in Eswatini, COVID-19 pandemic in Ethiopia, COVID-19 pandemic in Fiji, COVID-19 pandemic in Finland, COVID-19 pandemic in France, COVID-19 pandemic in French Polynesia, COVID-19 pandemic in Gabon, COVID-19 pandemic in Georgia (country), COVID-19 pandemic in Germany, COVID-19 pandemic in Ghana, COVID-19 pandemic in Greece, COVID-19 pandemic in Greenland, COVID-19 pandemic in Grenada, COVID-19 pandemic in Guatemala, COVID-19 pandemic in Guernsey, COVID-19 pandemic in Guinea, COVID-19 pandemic in Guinea-Bissau, COVID-19 pandemic in Guyana, COVID-19 pandemic in Haiti, COVID-19 pandemic in Honduras, COVID-19 pandemic in Hungary, COVID-19 pandemic in Iceland, COVID-19 pandemic in India, COVID-19 pandemic in Indonesia, COVID-19 pandemic in Iran, COVID-19 pandemic in Iraq, COVID-19 pandemic in Israel, COVID-19 pandemic in Italy, COVID-19 pandemic in Ivory Coast, COVID-19 pandemic in Jamaica, COVID-19 pandemic in Japan, COVID-19 pandemic in Jersey, COVID-19 pandemic in Jordan, COVID-19 pandemic in Kazakhstan, COVID-19 pandemic in Kenya, COVID-19 pandemic in Kiribati, COVID-19 pandemic in Kosovo, COVID-19 pandemic in Kuwait, COVID-19 pandemic in Kyrgyzstan, COVID-19 pandemic in Lesotho, COVID-19 pandemic in Liberia, COVID-19 pandemic in Libya, COVID-19 pandemic in Madagascar, COVID-19 pandemic in mainland China, COVID-19 pandemic in Malawi, COVID-19 pandemic in Maldives, COVID-19 pandemic in Mali, COVID-19 pandemic in Mauritania, COVID-19 pandemic in Mauritius, COVID-19 pandemic in Mayotte, COVID-19 pandemic in Melilla, COVID-19 pandemic in Morocco, COVID-19 pandemic in Mozambique, COVID-19 pandemic in Namibia, COVID-19 pandemic in New South Wales, COVID-19 pandemic in Niger, COVID-19 pandemic in Nigeria, COVID-19 pandemic in Norfolk Island, COVID-19 pandemic in Normandy, COVID-19 pandemic in Northern Cyprus, COVID-19 pandemic in Northern Ireland, COVID-19 pandemic in Puntland, COVID-19 pandemic in Queensland, COVID-19 pandemic in Rwanda, COVID-19 pandemic in Senegal, COVID-19 pandemic in Seychelles, COVID-19 pandemic in Sierra Leone, COVID-19 pandemic in Somalia, COVID-19 pandemic in Somaliland, COVID-19 pandemic in South Africa, COVID-19 pandemic in South Australia, COVID-19 pandemic in South Ossetia, COVID-19 pandemic in South Sudan, COVID-19 pandemic in Sudan, COVID-19 pandemic in Tanzania, COVID-19 pandemic in Tasmania, COVID-19 pandemic in the Australian Capital Territory, COVID-19 pandemic in the Bahamas, COVID-19 pandemic in the Canary Islands, COVID-19 pandemic in the Central African Republic, COVID-19 pandemic in the Cocos (Keeling) Islands, COVID-19 pandemic in the Comoros, COVID-19 pandemic in the Cook Islands, COVID-19 pandemic in the Czech Republic, COVID-19 pandemic in the

Democratic Republic of the Congo, COVID-19 pandemic in the Dominican Republic, COVID-19 pandemic in the Donetsk People's Republic, COVID-19 pandemic in the Faroe Islands, COVID-19 pandemic in the Federated States of Micronesia, COVID-19 pandemic in the Gambia, COVID-19 pandemic in the Guantanamo Bay Naval Base, COVID-19 pandemic in the Isle of Man, COVID-19 pandemic in the Kurdistan Region, COVID-19 pandemic in the Northern Territory, COVID-19 pandemic in the Republic of Artsakh, COVID-19 pandemic in the Republic of Ireland, COVID-19 pandemic in the Republic of the Congo, COVID-19 pandemic in the Sahrawi Arab Democratic Republic, COVID-19 pandemic in Togo, COVID-19 pandemic in Tunisia, COVID-19 pandemic in Uganda, COVID-19 pandemic in Victoria, COVID-19 pandemic in Western Australia, COVID-19 pandemic in Western Sahara, COVID-19 pandemic in Zambia, COVID-19 pandemic in Zimbabwe, COVID-19 pandemic on Charles de Gaulle, COVID-19 pandemic on Diamond Princess, Economic impact of the COVID-19 pandemic in the Republic of Ireland, Face masks during the COVID-19 pandemic, Federal aid during the COVID-19 pandemic in Canada, German government response to the COVID-19 pandemic, Ghanaian government response to the COVID-19 pandemic, Glossary of the COVID-19 pandemic, Impact of the COVID-19 pandemic on children, Impact of the COVID-19 pandemic on education in Ghana, Impact of the COVID-19 pandemic on education in the Republic of Ireland, Impact of the COVID-19 pandemic on Gaelic games, Impact of the COVID-19 pandemic on human rights in Argentina, Indian government response to the COVID-19 pandemic, Indian migrant workers during the COVID-19 pandemic, Indian state government responses to the COVID-19 pandemic, International reactions to the COVID-19 pandemic in Italy, List of species named after the COVID-19 pandemic, National responses to the COVID-19 pandemic in Africa, Nigerian government response to the COVID-19 pandemic, Outline of the COVID-19 pandemic, Pandemic predictions and preparations prior to the COVID-19 pandemic, Statistics of the COVID-19 pandemic in Argentina, Statistics of the COVID-19 pandemic in Australia, Statistics of the COVID-19 pandemic in Bangladesh, Statistics of the COVID-19 pandemic in Brazil, Statistics of the COVID-19 pandemic in Chile, Statistics of the COVID-19 pandemic in Germany, Statistics of the COVID-19 pandemic in India, Statistics of the COVID-19 pandemic in Indonesia, Statistics of the COVID-19 pandemic in Italy, Statistics of the COVID-19 pandemic in Japan, Statistics of the COVID-19 pandemic in Tamil Nadu, Timeline of the COVID-19 pandemic in Afghanistan, Timeline of the COVID-19 pandemic in Argentina, Timeline of the COVID-19 pandemic in Australia, Timeline of the COVID-19 pandemic in Australia (2022), Timeline of the COVID-19 pandemic in Bangladesh, Timeline of the COVID-19 pandemic in Belarus, Timeline of the COVID-19 pandemic in Belarus (2020), Timeline of the COVID-19 pandemic in Belarus (2021), Timeline of the COVID-19 pandemic in Belarus (2022), Timeline of the COVID-19 pandemic in Brazil, Timeline of the COVID-19 pandemic in Canada, Timeline of the COVID-19 pandemic in Colombia, Timeline of the COVID-19 pandemic in Croatia, Timeline of the COVID-19 pandemic in Fiji, Timeline of the COVID-19 pandemic in Ghana, Timeline of the COVID-19 pandemic in India, Timeline of the COVID-19 pandemic in India (2021), Timeline of the COVID-19 pandemic in Indonesia, Timeline of the COVID-19 pandemic in Indonesia (2020), Timeline of the COVID-19 pandemic in Indonesia (2021), Timeline of the COVID-19 pandemic in Indonesia (2022), Timeline of the COVID-19 pandemic in Italy, Timeline of the COVID-19 pandemic in Japan, Timeline of the COVID-19 pandemic in Malaysia, Timeline of the COVID-19 pandemic in Malta, Timeline of the COVID-19 pandemic in Mexico, Timeline of the COVID-19 pandemic in Nepal, Timeline of the COVID-19 pandemic in New Zealand, Timeline of the COVID-19 pandemic in Nigeria, Timeline of the COVID-19 pandemic in Pakistan, Timeline of the COVID-19 pandemic in Romania, Timeline of the COVID-19 pandemic in Russia, Timeline of the COVID-19 pandemic in Serbia, Timeline of the COVID-19 pandemic in Singapore, Timeline of the COVID-19 pandemic in South Africa, Timeline of the COVID-19 pandemic in Spain, Timeline of the COVID-19 pandemic in Sweden, Timeline of the COVID-19 pandemic in Thailand, Timeline of the COVID-19 pandemic in the Philippines, Timeline of the COVID-19 pandemic in the Republic of Ireland, Timeline of the COVID-19 pandemic in the Republic of Ireland (2021), Timeline of the COVID-19 pandemic in the Republic of Ireland (2022), Timeline of the COVID-19 pandemic in the United Kingdom, Timeline of the COVID-19 pandemic in the United States, Timeline of the COVID-19 pandemic in Trinidad and Tobago, Timeline of the COVID-19 pandemic in Turkey, Timeline of the COVID-19 pandemic in Uruguay, Timeline of the COVID-19 pandemic in Vietnam, World Health Organization response to the COVID-19 pandemic in Africa.

Additional analyses

Complementing the analysis presented in the 2023 *Lancet* Countdown report, the figures below provide additional evidence on dynamics in pageviews and co-click networks.

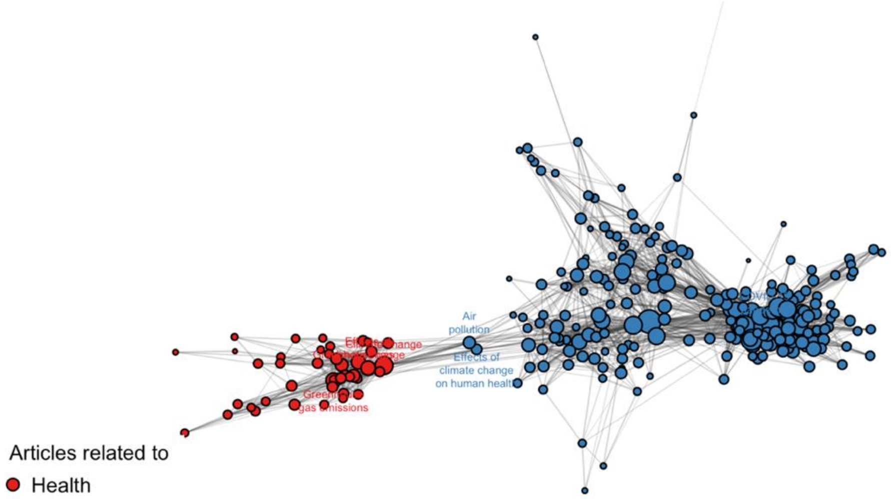

Figure 137. Connectivity graph of Wikipedia articles on climate change (red) and health (blue). Popularity of articles displayed by node size. Edges represent co-visits in the 2022 clickstream data.

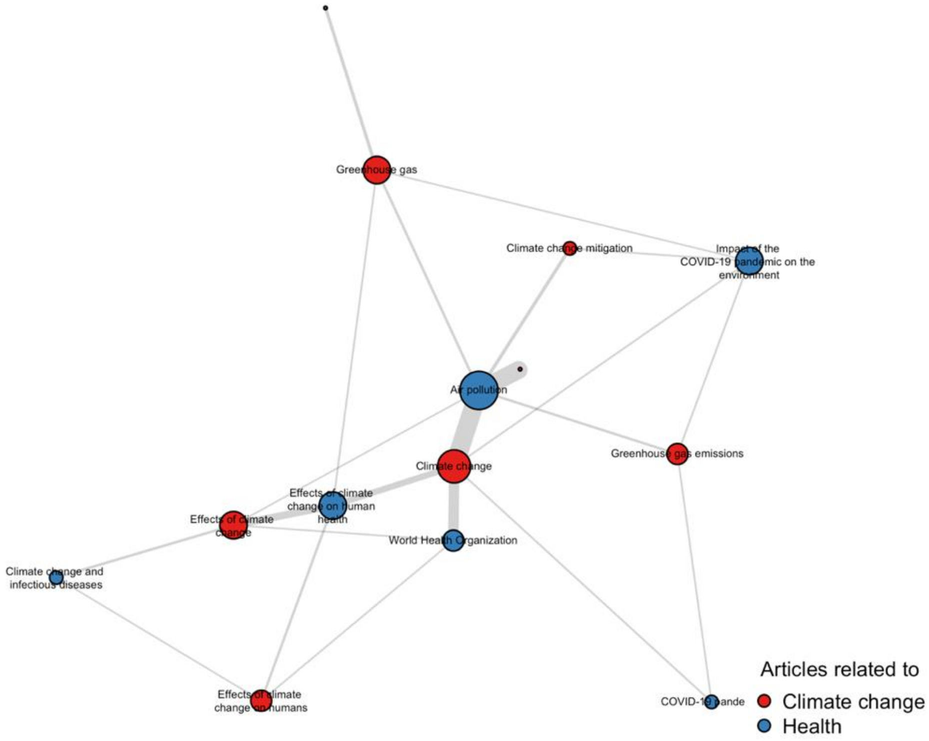

Figure 138. Connectivity graph of Wikipedia articles on climate change (red) and health (blue), filtered to co-click activity between the two domains. Popularity of articles displayed by node size. Edges represent co-visits in the 2022 clickstream data.

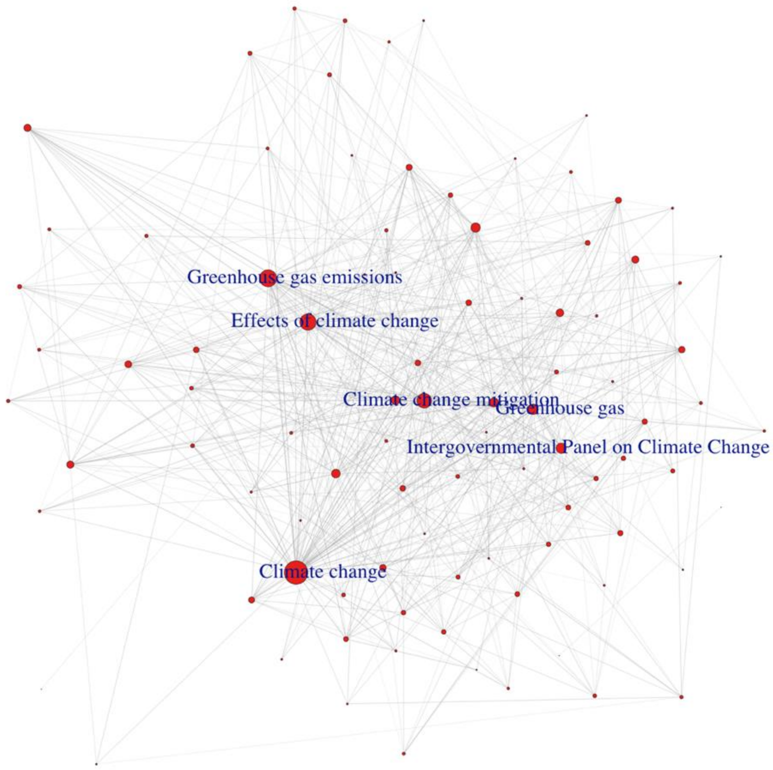

Figure 139. Connectivity graph of Wikipedia articles on climate change. Popularity of articles displayed by node size. Edges represent co-visits in the 2022 clickstream data.

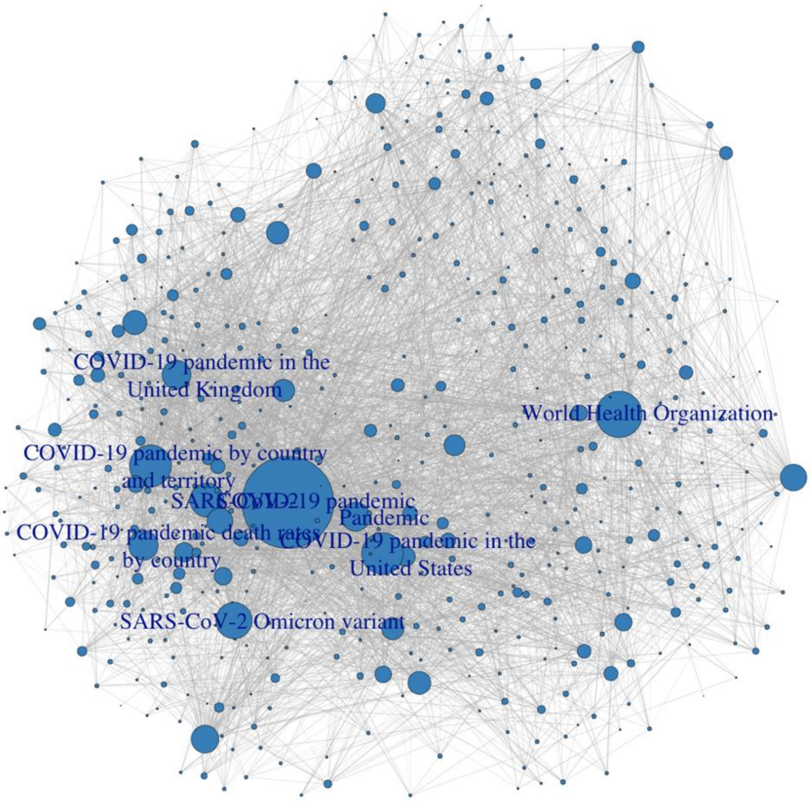

Figure 140. Connectivity graph of Wikipedia articles on health. Popularity of articles displayed by node size. Edges represent co-visits in the 2022 clickstream data.

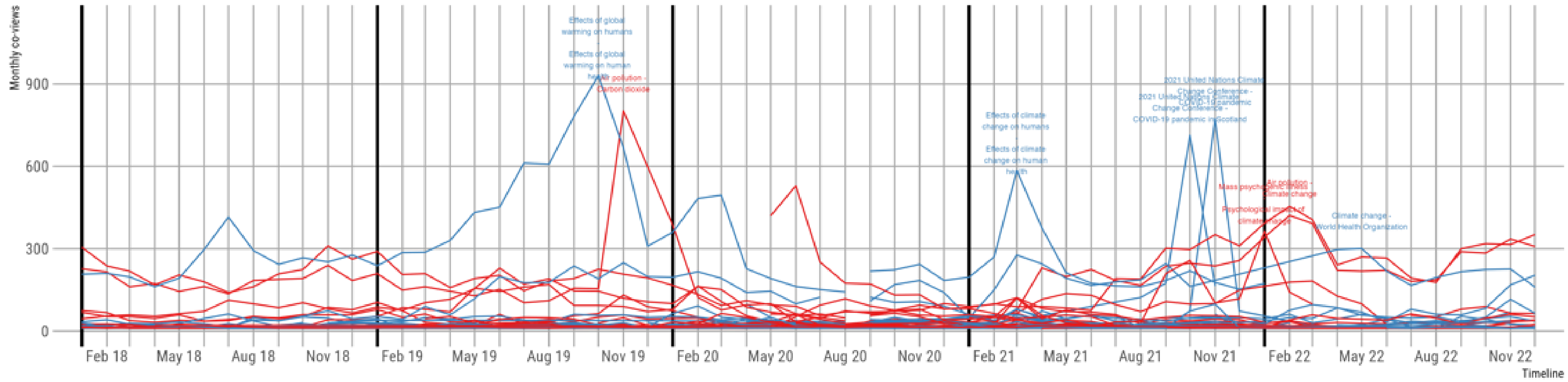

Figure 141. Co-views of climate change-health article pairs over time, 2018–2022. Dominant pairs labelled.

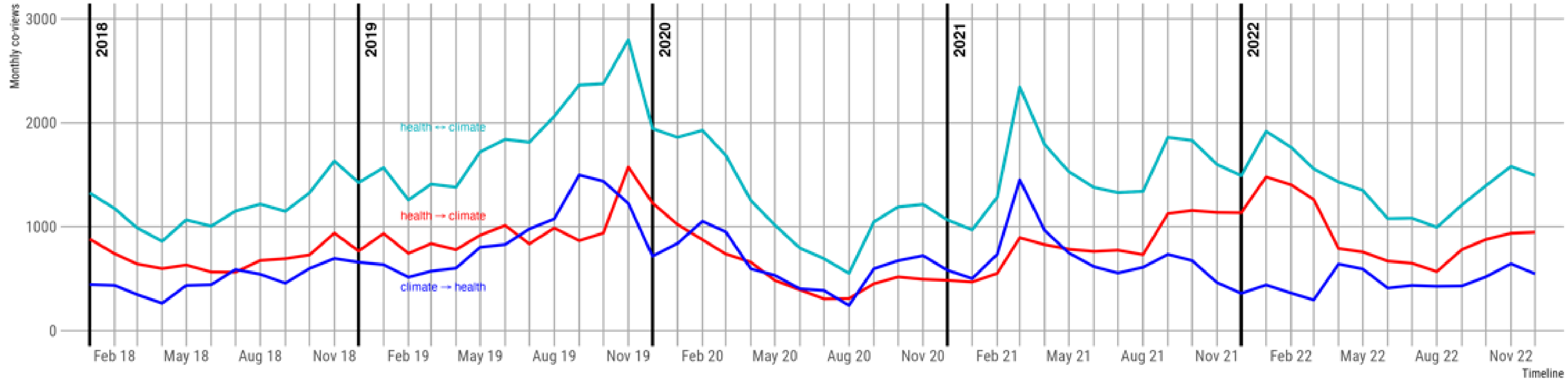

Figure 142. Aggregate monthly co-views of articles related to human health and climate change, 2018–2022 (excluding COVID-19 related articles).

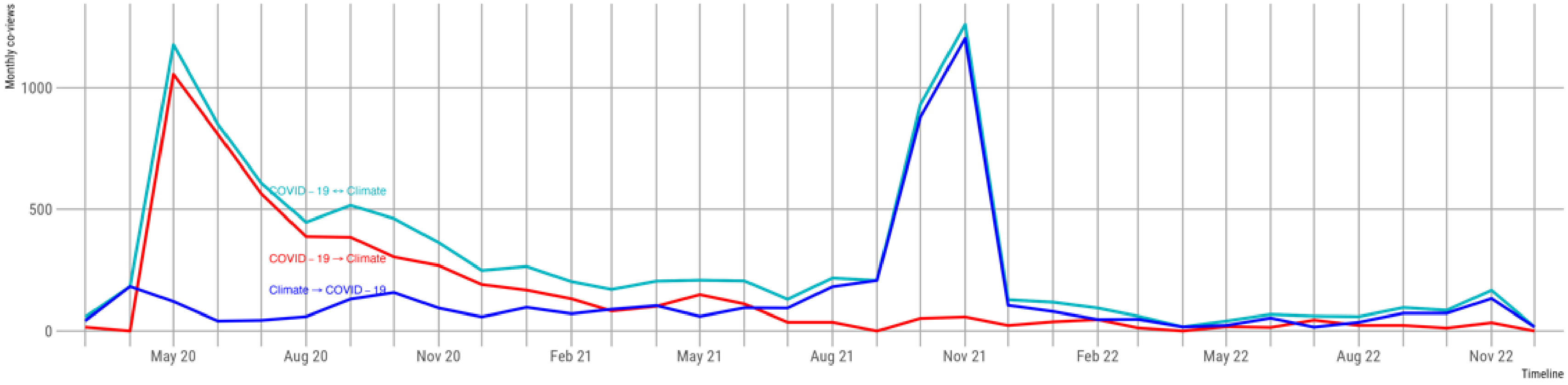

Figure 143. Aggregate monthly co-views of articles related to COVID-19 and climate change, 2020–2022

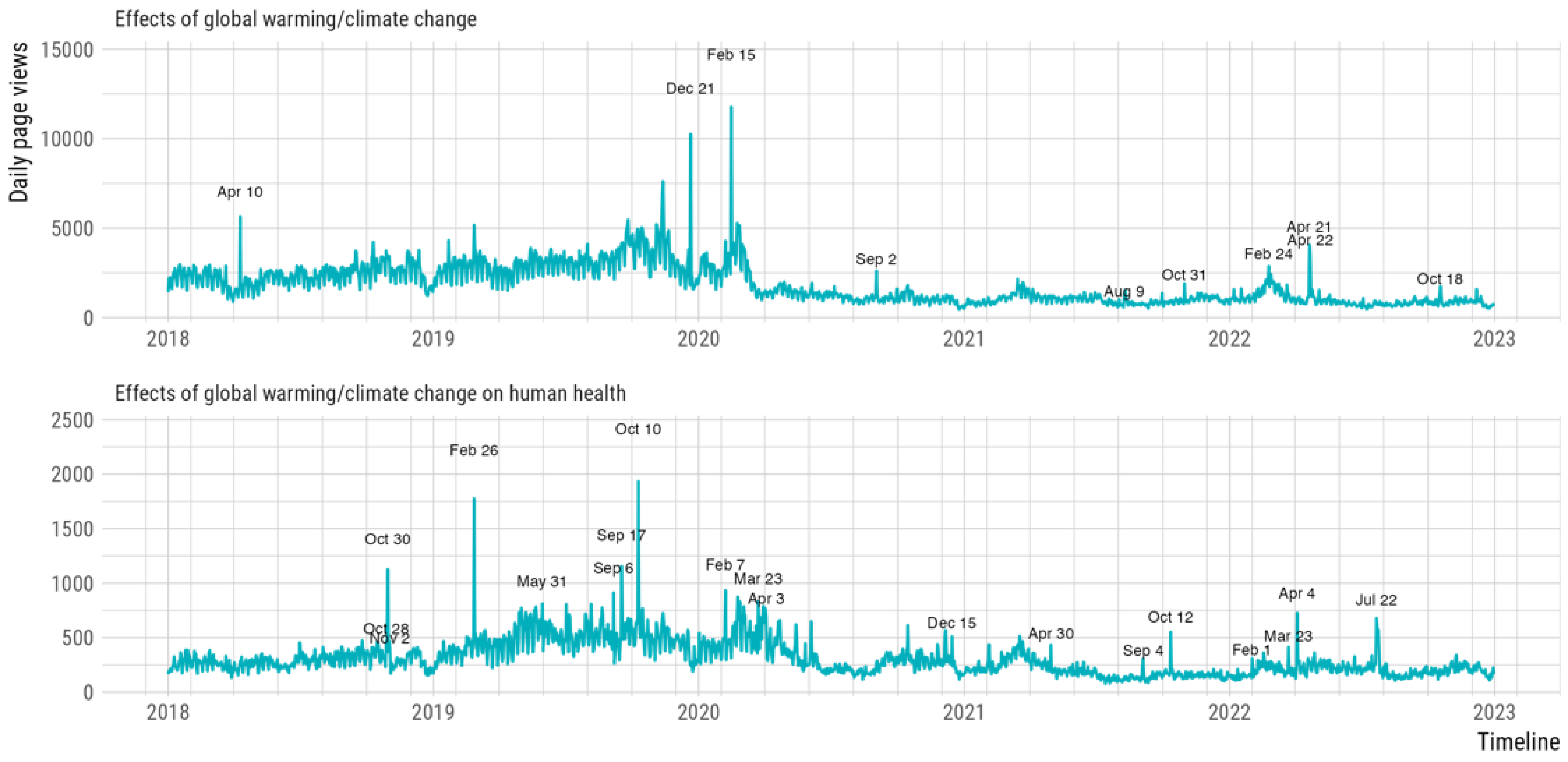

Figure 144. Daily page views 2018 to 2022 for Wikipedia articles directly related to the effects of climate change in general and on human health.

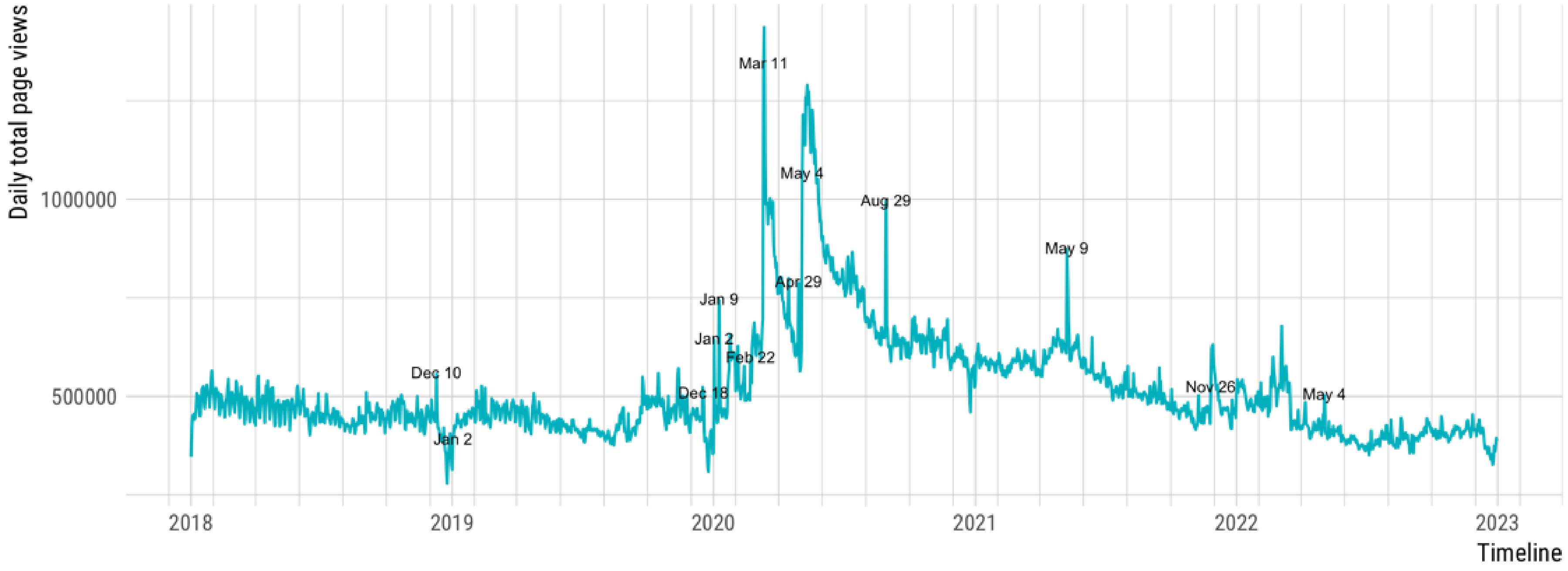

Figure 145. Aggregate daily page views 2018 to 2021 for all 1,414 selected articles on the English Wikipedia related to health.

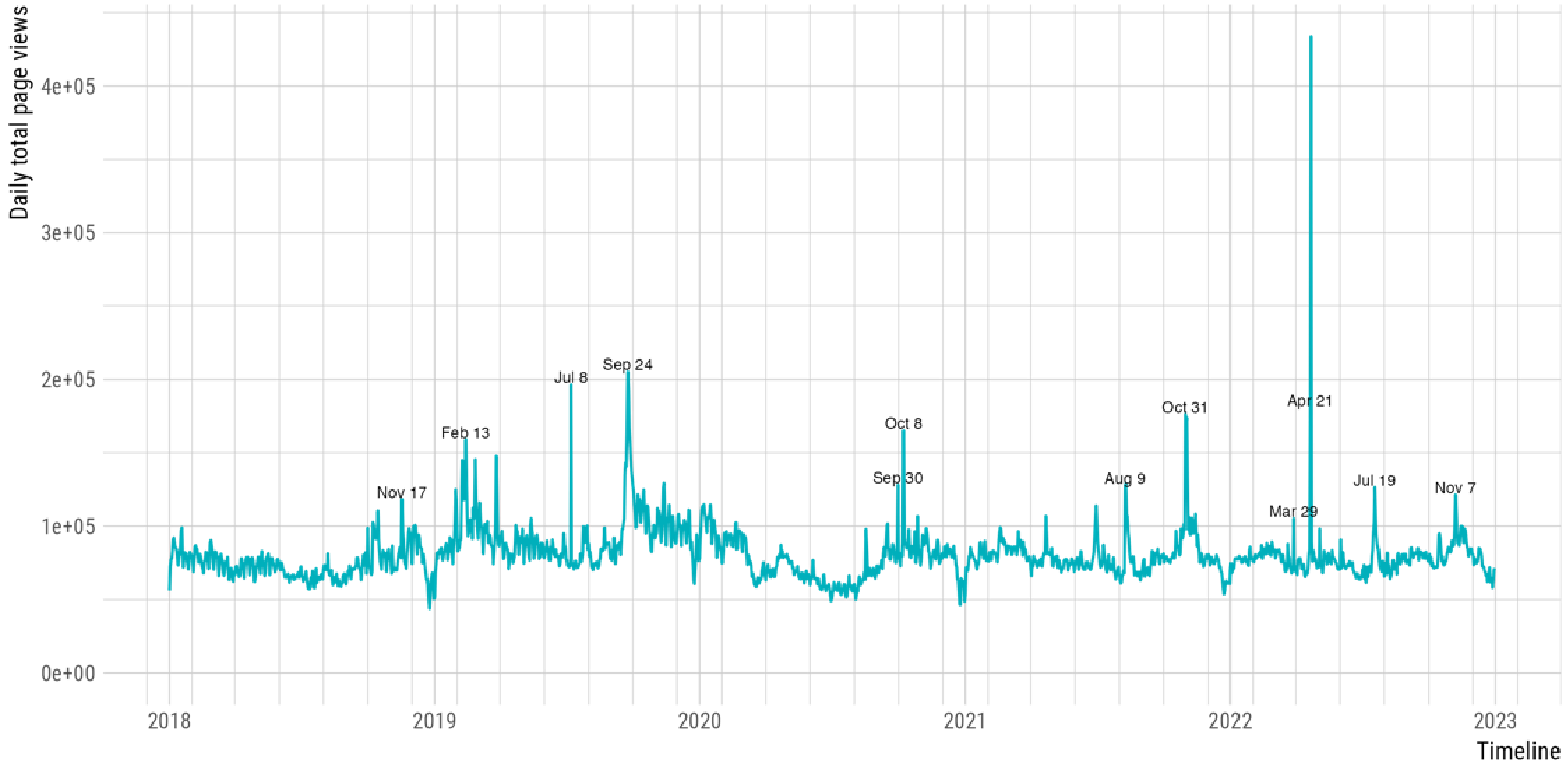

Figure 146. Aggregate daily page views 2018 to 2021 for all 610 selected articles on the English Wikipedia related to climate change

5.3: Scientific Engagement in Health and Climate Change

Indicator 5.3.1: Scientific Articles on Health and Climate Change 1990-2022

Indicator authors

Prof Lea Berrang Ford, Dr Max Callaghan, Prof Jan C. Minx

Method

Scientific engagement in health and climate change is central to the *Lancet* Countdown mission: this is to facilitate, support, and track progress on health and climate change. Scientific evidence is the major resource on which such progress rests; it also informs engagement in the key domains of global action, including public, governmental and corporate domains. Based on a machine-learning methodology developed elsewhere,[350] this indicator quantifies engagement in the topic of climate change and health by tracking the number of publications over time. A machine-learning approach allows for a more granular picture of the research landscape, including developments across major domains of research (mitigation, adaptation, impacts), the health impacts covered, locations studied, as well as patterns of authorship.

The objective for this indicator is to systematically track the evidence on the relationship between climate change, climate variability, and weather (CCVW) and human health globally. Such a broad framing is necessary to adequately represent the relevant scientific work in such a diverse field, where not all relevant work involves formal climate change attribution. The evidence map is framed using a PICoST approach: population/problem (P), interest (I), context (Co), and scope and time (S/T). The scope is global, interest is in empirical evidence on the relationships between climate change, climate variability, as well as weather (CCVW) and human health, and coverage includes any scientific article or review covered by bibliographic records in OpenAlex. Interest is in any context, i.e., any component of the nexus between climate change, climate variability, and weather (CCVW) and human health, including impacts on health, and responses to reduce health impacts from climate change (e.g., adaptation, mitigation), without prejudice to any climate-health pathway. This search as well as inclusion and exclusion criteria are framed within this PICoST framework.

Relevant documents were those which were indexed in English; provided a clear link to actual, projected, or perceived impacts of climate change, responses to reduce the impacts of climate change (adaptation), or the mitigation of greenhouse gas emissions; and included substantial focus on a perceived, experienced, or observed eligible health-related outcome or health system; and presented empirically driven research or a review of such research.

A support vector machine (SVM) classifier[351] was trained using document abstracts to reproduce the inclusion/exclusion decisions as well as the impacts/mitigation/adaptation labels. Classifier performance was evaluated using 10-fold cross-validation. The inclusion/exclusion classifier achieved an accuracy of 87.1% with 80% recall and 76% precision.

Here the same machine learning model is applied to classify new studies which were not available when the original paper[350] was produced. Those studies predicted to be irrelevant were discarded. Then the multilabel impacts/mitigation/adaptation classifier was applied to those documents which were included. Documents can be classified as belonging to one or more of the noted categories.

Finally, a neural network based “geoparser”[352] was applied to titles and abstracts of the texts to extract the geographical entities mentioned in the texts. These locations were allocated to countries, and then to WHO regions. Country names were also extracted from the institutional affiliations recorded by the bibliographic databases.

Theme	Key concepts	String (Scopus)	Attributable Hits (scopus)
**Climate change** *(contains at least one of the following climate terms, from any category)*	General climate change terms	(climat* OR "global warming" OR "greenhouse effect*")	**35,052**
	Greenhouse gasses, including short-lived greenhouse gasses, when linked to emission or mitigation. Some astronomy results are filtered out.	(("carbon dioxide" OR co2 OR methane OR ch4 OR "nitrous oxide" OR n2o OR "nitric oxide" OR "nitrogen dioxide" OR nox OR *chlorofluorocarbon* OR *cfc* OR refrigerant OR hydrofluorocarbon* OR hfc* OR *chlorocarbon* OR "carbon tetrachloride" OR ccl4 OR halogen* OR ozone OR o3 OR ammonia OR nh3 OR "carbon monoxide" OR co OR "volatile organic compounds" OR nmvoc OR "hydroxyl radical" OR "oh" OR "pm2.5" OR aerosol OR "black carbon" OR "organic carbon" OR "sulphur dioxide" OR "oxidized sulphur" OR "so2" OR "sox" OR "sulphuric acid" OR so4*) W/2 (emit* OR emission OR releas* OR mitigat*) AND NOT(star OR "solar system"))	**7,871**
	Climate variability indicators/climate indices	(temperature* OR precipitat* OR rainfall OR "heat ind*" OR "extreme-heat event*" OR "heat-wave" OR "extreme-cold*" OR "cold ind*" OR humidity OR drought* OR hydroclim* OR monsoon OR "el ni$o" OR enso OR SOI OR "sea surface temperature*" OR sst)	**199,558**
	Complex climate indices, including extreme weather events, floods, wildfire, and coastal changes. Some paleo-climatic events are excluded.	(snowmelt* OR flood* OR storm* OR cyclone* OR hurricane* OR typhoon* OR "sea-level" OR wildfire* OR "wild-fire*" OR "forest-fire*" OR ((extreme W/1 event*) AND NOT paleo*) OR "coast* erosion" OR "coastal change*" OR (disaster* W/1 (risk OR manag* OR natural)))	**22,031**
**AND**	General health terms	(health* OR well?being OR ill OR illness OR disease* OR syndrome* OR infect* OR medical*)	**49,773**

**Health**<br><br>*(contains at least one of the following health terms, from any category)*	General health outcomes	(mortality OR daly OR morbidity OR injur* OR death* OR hospital* OR {a&e} OR emergency OR emergencies OR doctor OR gp)	**33,571**
	Nutrition, including obesity and undernutrition	(obes* OR over?weight OR under?weight OR hunger OR stunting OR wasting OR undernourish* OR undernutrition OR anthropometr* OR malnutrition OR malnour* OR anemia OR anaemia OR "micronutrient*" OR "micro?nutrient*" OR diabet*)	**2,239**
	Cardio-vascular terms. Some studies on Chemical Vapour Deposition (CVD) are excluded.	(hypertension OR "blood pressure" OR stroke OR *vascular OR (cvd AND NOT(vapour or vapor)) OR "heart disease" OR isch?emic OR cardio?vascular OR "heart attack*" OR coronary OR chd)	**6,047**
	Renal health terms	(ckd OR renal OR cancer OR kidney OR lithogenes*)	**4,934**
	Effects of temperature extremes	((heat W/2 (stress OR fatigue OR burn* OR stroke OR exhaustion OR cramp*)) OR skin OR fever* OR renal* OR rash* OR eczema* OR "thermal stress" OR hypertherm* OR hypotherm*)	**23,846**
	Maternal health outcomes	(pre?term OR stillbirth OR birth?weight OR lbw OR maternal OR pregnan* OR gestation* OR *eclampsia OR sepsis OR oligohydramnios OR placenta* OR haemorrhage OR hemorrhage)	**2,041**
	Vector-borne diseases	(malaria OR dengue* OR mosquito* OR chikungunya OR leishmaniasis OR encephalit* OR vector-borne OR pathogen OR zoonos* OR zika OR "west nile" OR onchocerciasis OR filiariasis OR lyme OR tick?borne)	**2,257**
	Bacterial, parasitic and viral infections, including waterborne	(waterborne OR “water borne” OR diarrhoea* OR diarrhe*l OR gastro* OR enteric OR *bacteria* OR viral OR *virus* OR parasit* OR vibrio* OR cholera OR protozoa* OR salmonella OR giardia OR shigella OR campylobacter OR food?borne	**46,064**

	and foodborne diseases	OR aflatoxin OR poison* OR ciguatera OR((snake* OR adder*) W/2 bite*))	
	Respiratory outcomes	(respiratory OR allerg* OR lung* OR asthma* OR bronchi* OR pulmonary* OR copd OR rhinitis OR wheez*)	**3,432**
	Mental health outcomes	(mental OR depress* OR *stress* OR anxi* OR ptsd OR psycho* OR *trauma* OR suicide* OR solastalgi*)	**12,616**
	Health systems	[no additional terms needed]	

Table 84. Search strategy

Data

This indicator uses data from bibliographic records in the online scientific database OpenAlex.

Caveats

Only English-language search terms are used within this protocol. Any language bias is limited, however, given that key international scientific platforms index all papers (including non-English papers) with keywords, title, and abstract in English translation. The protocol uses title, keywords, and abstract to retrieve and assess the scientific literature, meaning that non-English papers can still be retrieved as long as they are indexed in English.

The methodology provided here enables a quantitative appraisal of the research question. The use of machine learning means that there will be some uncertainty as to the number of relevant documents, although this uncertainty can be communicated. Further, the quality of the individual studies and the specifics of their content will not be assessed by the indicator team. However, with the outputs all published in peer-reviewed journals, there is a de facto check on quality. For this reason, the indicator does not cover grey literature.

Focusing on peer-reviewed literature will mean some scientific work of interest will be missed, particularly from the grey literature. Systematic search of grey literature is challenging, but future work could develop web-crawling approaches to find relevant reports and other publications outside of the peer reviewed literature.

Additional information

There is a large scientific literature on climate and health, comprising approximately 25,000 publications. 80% of these have been published in the last 10 years. Publications in 2022 remained high. The 3,149 papers published in 2022 is about three times higher than the number published in 2012, although it is 8% lower than the figure for 2021. Publication counts in 2022 were 15% higher than in 2020, and the 5-year compound annual growth rate stood at 15%, compared to 18% in 2021, and just 8% in 2019. It remains to be seen whether 2022 represents a slowdown, or rather a return to previous trends after exceptionally high years in 2020 and 2021.

Climate and health literature is found in all regions, albeit with substantial variations between regions. The region most studied in 2022 was Asia, with 1,095 studies. Northern America and Europe followed with 398 and 305 studies respectively. 254 studies addressed locations in Africa, 142 studies investigated South and Central America, and 80 and 51 studies addressed Oceania and Small Island Developing States (SIDS).

Climate and health research continues to be dominated by studies on climate impacts, while there is comparatively little research on co-benefits and adverse impacts of mitigation and adaptation policies. Studies on health impacts fell by the largest amount from 2021 to 2022, both in absolute terms from

2,411 papers to 2,182 and in relative terms (9,4%). Studies on adaptation fell by 7.1% from 154 to 143 papers, while studies on mitigation fell 6.2% from 192 to 180 papers (**Figure 147**).

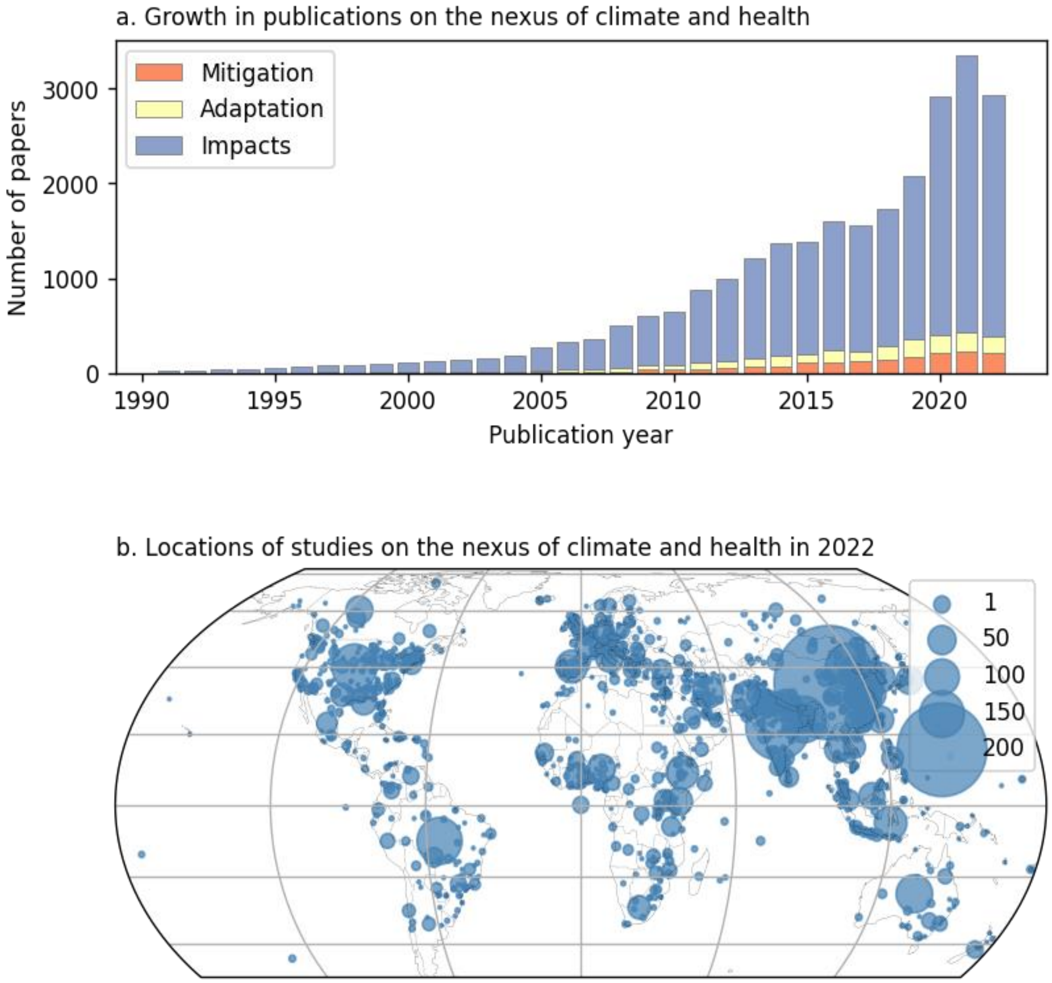

Figure 147. a) Growth in publications around health and climate change, and b) locations of studies

Indicator 5.3.2: Scientific Engagement on the Health Impacts of Climate Change

Attribution Studies of Extreme Events and Their Health Outcomes

Indicator authors

Prof Lea Berrang Ford, Dr Max Callaghan, Prof Jan C. Minx

Methods

Extreme climate events from attribution studies were categorized according to the relative impact of anthropogenic climate change on their likelihood and/or extremity, and the corresponding mortality and morbidity impacts. Detection and attribution is widely used within climate science and other fields, and increasingly being used to within public health as a means of characterising and quantifying current health impacts and future risks.[353]

Each event is categorically considered a) more likely or severe due to climate change, b) less likely or severe due to climate change or c) the impact of climate change on likelihood or severity of event is neutral or unclear. Events are also categorised by the type of event (for example: heavy precipitation and flood events, wildfires, extreme heat events, storms, tornadoes and cyclones, and drought and low precipitation events) and the region most affected. The studies were separated by LCD region and HDI levels to consider the differing impacts of these extreme events and the number of studies by region. A content analysis was also conducted to determine the proportion of attribution studies that made direct reference to health impacts.

Data

Attribution data included for analysis was sourced from studies published by major attribution research groups and their associated publications, namely: World Weather Attribution and American Meteorological Society. Although these groups captured the majority of detection and attribution studies, an additional literature search was conducted on online databases Scopus and Web of Science. Compliance with the IPCC standards for detection and attribution research was considered an inclusion criterion.[354]

Correlation of attributable events was made, where possible, with the Centre for Research on the Epidemiology of Disasters (CRED) Emergency Events Database (EM-DAT), from which morbidity and mortality data was preferentially sourced. Further health impact data was drawn from attribution studies where available, as well as through literature searches on online databases Scopus and Web of Science. In a few instances, health impact data was also sourced from non-academic sources including health system datasets and press releases from healthcare authorities.

Caveats

This indicator is limited by the number of published attribution studies and their chosen focus. As past detection and attribution studies have predominantly focused on extreme events occurring in more developed region[175], the paucity of data relating to the health impacts of attributable events in less developed regions is recognised.

Although mortality data are discrete, numerical data that are easily aggregable and comparable across events, measures of morbidity are multiple and varied, which can constrain data analysis, thereby limiting interpretations. Additionally, health impacts attributed to extreme climate events systematically underestimate true health burdens due to data availability and methodological limitations.[175,355]

Future form of the indicator

The proposed indicator will consolidate detection and attribution as an effective and reliable means of quantifying the effects of climate change on human health. It can eventually be expanded to work directly with groups that conduct detection and attribution research to more directly attribute health burdens to climate change. There will be some work to aim towards this within the next two years.

Additionally, the contribution of human induced climate change on each event can be presented as a percentage relative contribution on its severity and/or the likelihood of it occurring, such as expressed in the form of a

probability ratio. Although the analysis of this data is currently limited by variable statistical expression of these relationships and large confidence intervals, it may represent a future opportunity for additional quantitative analysis.

Additional analysis

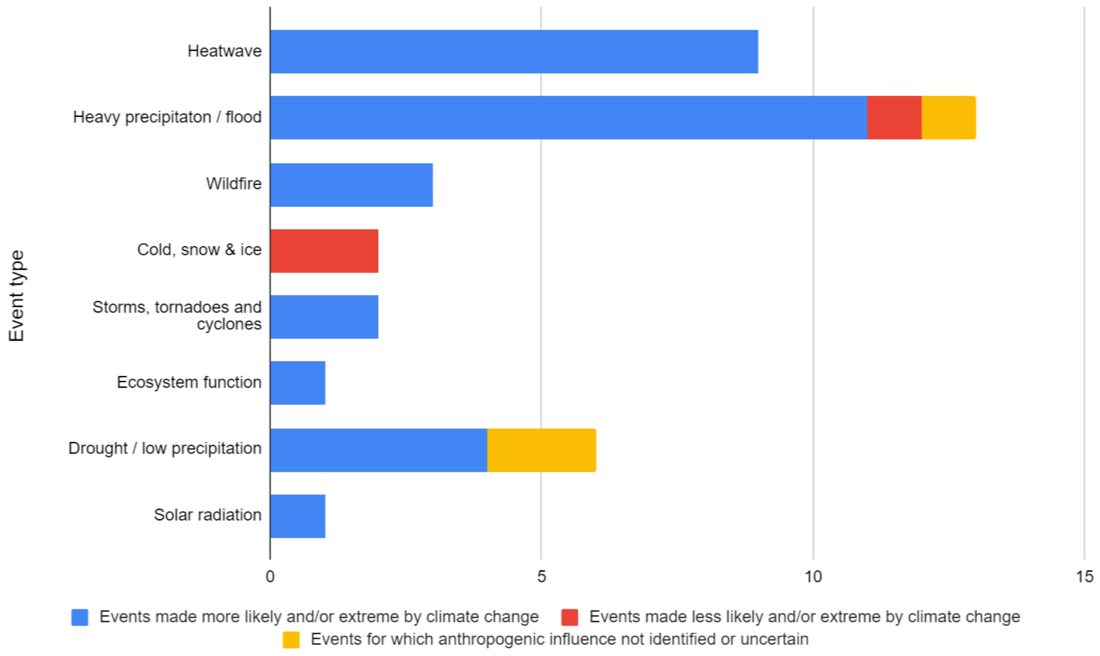

Figure 148. Attribution of extreme events published 2022-2023: Impact of climate change on the likelihood and/or severity of extreme event by event type. Direct mortality and injuries for all events totalled 12,436 and 16,524, respectively.

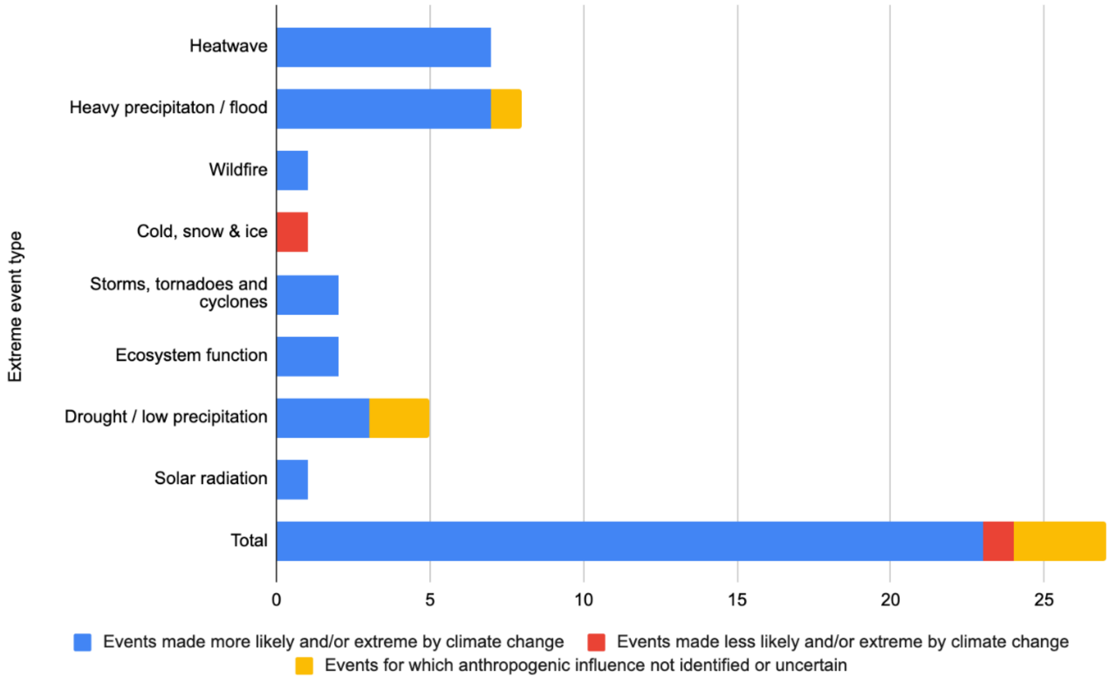

Figure 149. Detection and attribution of the influence of climate change on extreme events, by event type

Scientific studies on the health impacts of climate change

Methods

There are multiple pathways in which climate and health outcomes are interlinked. A large and growing literature maps out the ways climate can impact human health, without necessarily isolating the role of human influence on the climate in driving the impacts. This indicator identifies this literature, classifying studies according to the climate drivers and health impacts studied, and locating where such impacts are studied. Using observational data and climate models, it identifies the subset of the literature which finds health impacts driven by climate variables, that are located in areas where changes in those variables can be attributed to human influence on the climate. These studies are referred to here as partially attributable impact studies.

Scientific studies form the basis of our understanding of how climate change and health are linked. Indicator 5.3 already identifies and tracks studies which provide evidence on climate and health. However, for those studies which address the impacts of climate on health, which form the majority of the evidence base, the studies themselves do not necessarily address the full chain of links between anthropogenic climate change, changes in a climate variable, and health impacts. Many focus only on the links between the latter two concepts. A novel approach is followed here,[356] to link studies on climate impacts with data from climate models and the observational record, in order to shed light on attribution across the whole chain from human influence on the climate, to the health impacts of climate change. The result is a new cross-working-group indicator that characterizes the available evidence from scientific studies on attributable climate impacts on human health.

The indicator starts from data from indicator 5.3, which identifies the relevant scientific literature on climate and health. Machine-learning classifiers from natural language processing were trained and applied to identify and classify a subset of documents with regard to the climate driver, the specific type of health impact, and the type of evidence provided as well as the time and location of the impact. By combining this geo-referenced set of documented climate-related health impacts with grid-cell-level human-attributable changes in temperature and precipitation, it is possible to provide a

comprehensive evidence base on attributable health impacts, which is the basis for the indicator calculations.

The open access database Open Alex is searched for documents related to climate and health using a query developed in Berrang-Ford et al.[350] Two thousand examples were screened, including where documents mentioned a climate variable or extreme event AND a health impact or exposure, as defined in the *Lancet* Countdown's model of climate and health linkages. Reading the abstracts, the climate variable, the extreme event, the health impact, the exposure, and the attribution type were coded. Each document was double coded, with all inconsistencies resolved manually in discussion with a third coder if necessary.

A machine learning classifier was subsequently trained to reproduce the screening and coding decisions from the initial training sample. For each task, hyperparameters were optimized using tree-structured Parzen estimator[357] and nested cross-validation, testing 3 different transformer-based models. The results of the optimization process are reported for each model and each task below.

	Category	bert-tiny	climatebert	distilroberta-base	scincl
**0**	Relevance	0.44 (0.29)	**0.85 (0.0082)**	0.82 (0.017)	0.84 (0.016)
**1**	Climate driver	0.54 (0.0048)	0.44 (0.23)	0.55 (0.0098)	**0.61 (0.062)**
**2**	Health impact	0.21 (0.11)	0.59 (0.044)	0.58 (0.013)	**0.68 (0.058)**
**3**	Attribution type	0.13 (0.027)	0.58 (0.047)	0.5 (0.047)	**0.6 (0.018)**
**4**	Extreme event	0.012 (0.017)	**0.73 (0.071)**	0.71 (0.09)	0.66 (0.014)
**5**	Exposure	0.0019 (0.0027)	0.36 (0.11)	0.36 (0.11)	**0.51 (0.14)**

Table 85. F1 scores (the mean of precision and recall) for each classifier and task

Trends in temperature and precipitation in grid cells are attributed following the procedure from Knutson et al. [358] and Knutson and Zeng.[359] As in Callaghan et al.,[356] this indicator shows where impact studies coincide geographically with attributable trends in the climate driver investigated.

Caveats

It is important to acknowledge that although English is the *lingua franca* in scientific literature, searching for studies in English may result in additional gaps in coverage, which it is hoped can be addressed with multilingual searches in future versions of the indicator.

The method here cannot fully attribute the health outcomes identified in each study to human influence on the climate. Rather, it shows where the health outcomes of changes in climate variables coincide geographically with changes in those variables that can be attributed to human influence.

Additional Information

There is a large literature that investigates the impacts of climate on health, although the role of human-induced climate change is not always addressed in each study. 31,254 publications on the impacts of climate on health were identified, of which 21,509 investigate how either temperature or precipitation have influenced health outcomes (68.8%). By comparing observed trends in temperature and precipitation with model-based counterfactuals exploring a world without anthropogenic forcing and with climate model runs that model the effects of anthropogenic forcing,[358,359] the indicator demonstrates where trends in temperature and precipitation can be attributed to human influence on the climate. 16,708 of the studies showing impacts driven by changes in temperature or precipitation

cover geographies where at least 50% of the area has been exposed to trends in the respective climate variable which can be attributed to human influence.

There is a large geographic variability in the number of studies, which is not proportional to the number of people exposed to trends in climate variables that can be attributed to human influence. For instance, there are 22 million people in Oceania living in 2.5 degree grid cells where trends in either temperature or precipitation can be attributed to human influence on the climate, and 1,139 climate and health studies focussing on a location in which the climate variable driving the impact can be attributed to human influence for over 50% of the area. That is a ratio of 49.8 studies per million exposed people. In North America, Europe, and South America the ratios of studies per million people stand at 13.4, 6.6, and 4.0 respectively. Asia has 7,481 studies for its 4 billion inhabitants exposed to attributable trends in temperature or precipitation, a ratio of 1.8 per million, while Africa displays a ratio of 2.0 per million.

The disparity highlights the unequal access to resources across the world and shows that there are many areas exposed to climate change and vulnerable to its effects, of which there is little knowledge in the scientific literature.

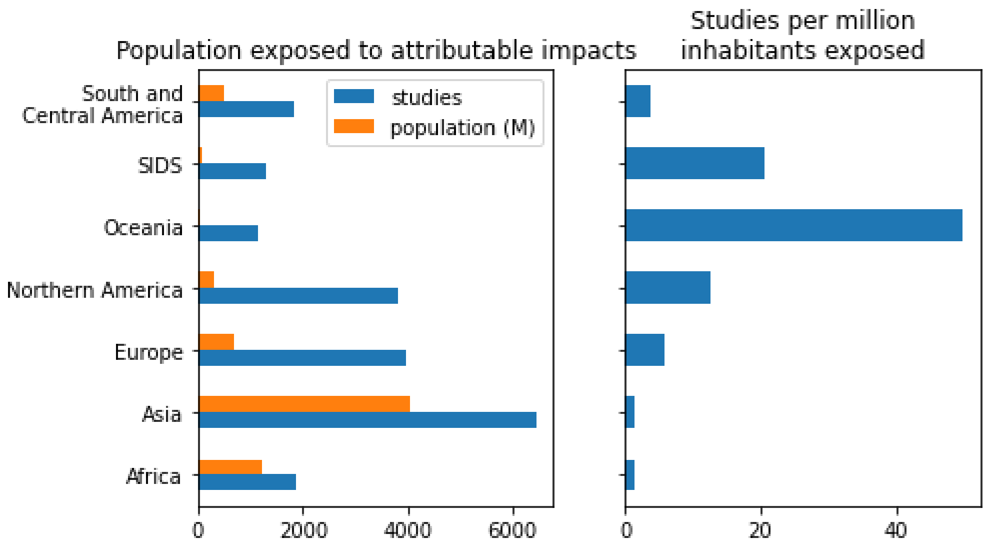

Figure 150. The number of people in each Lancet Countdown region exposed to trends in temperature or precipitation that can be attributed to human influence on the climate, compared to the number of studies exploring health impacts of climate drivers where those drivers display attributable trends.

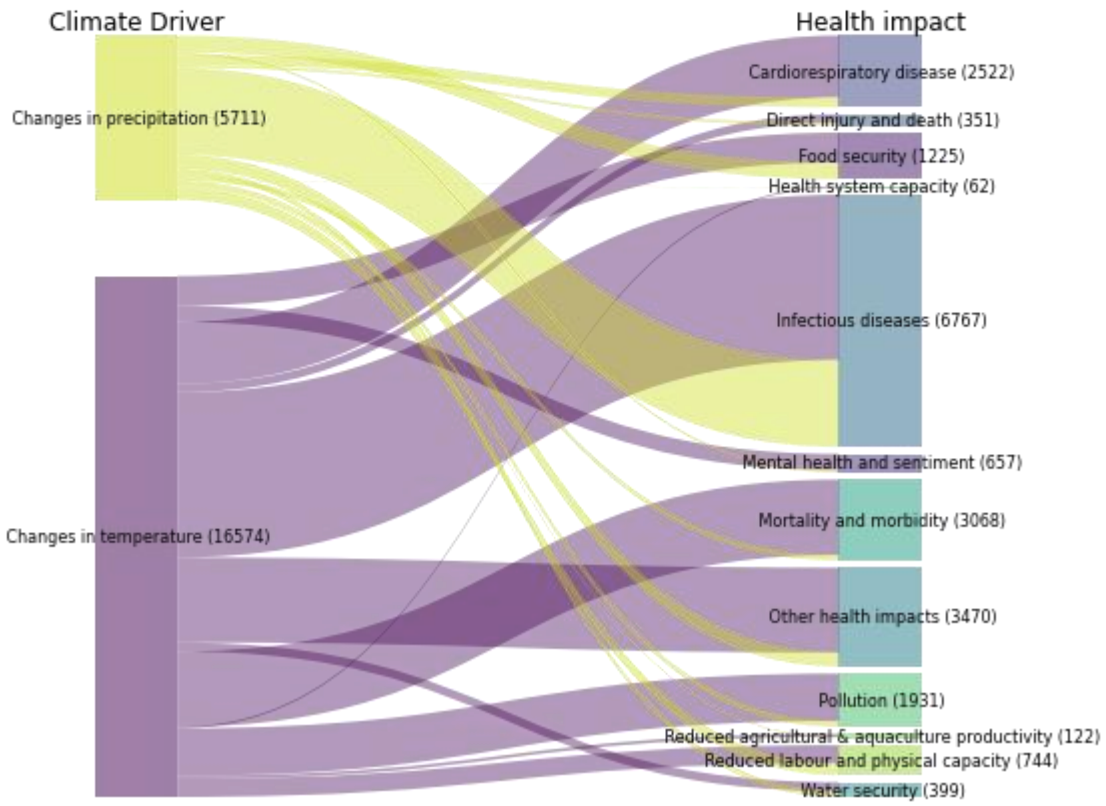

Figure 151. The number of papers exploring the links between temperature and precipitation and health outcomes, where the study focuses on area where trends in the relevant variable can be attributed to human influence on the climate. Note that single studies may investigate multiple drivers or health impacts.

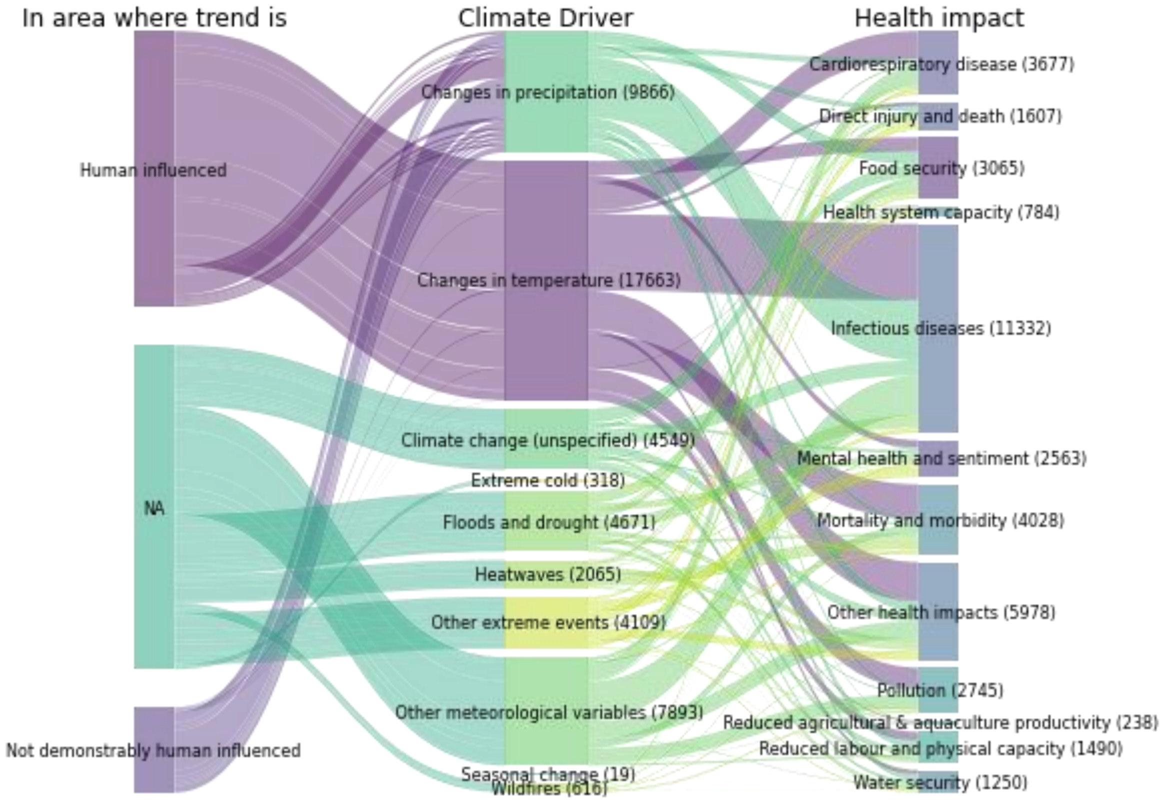

Figure 152. The number of papers addressing links between climate drivers and health impacts.

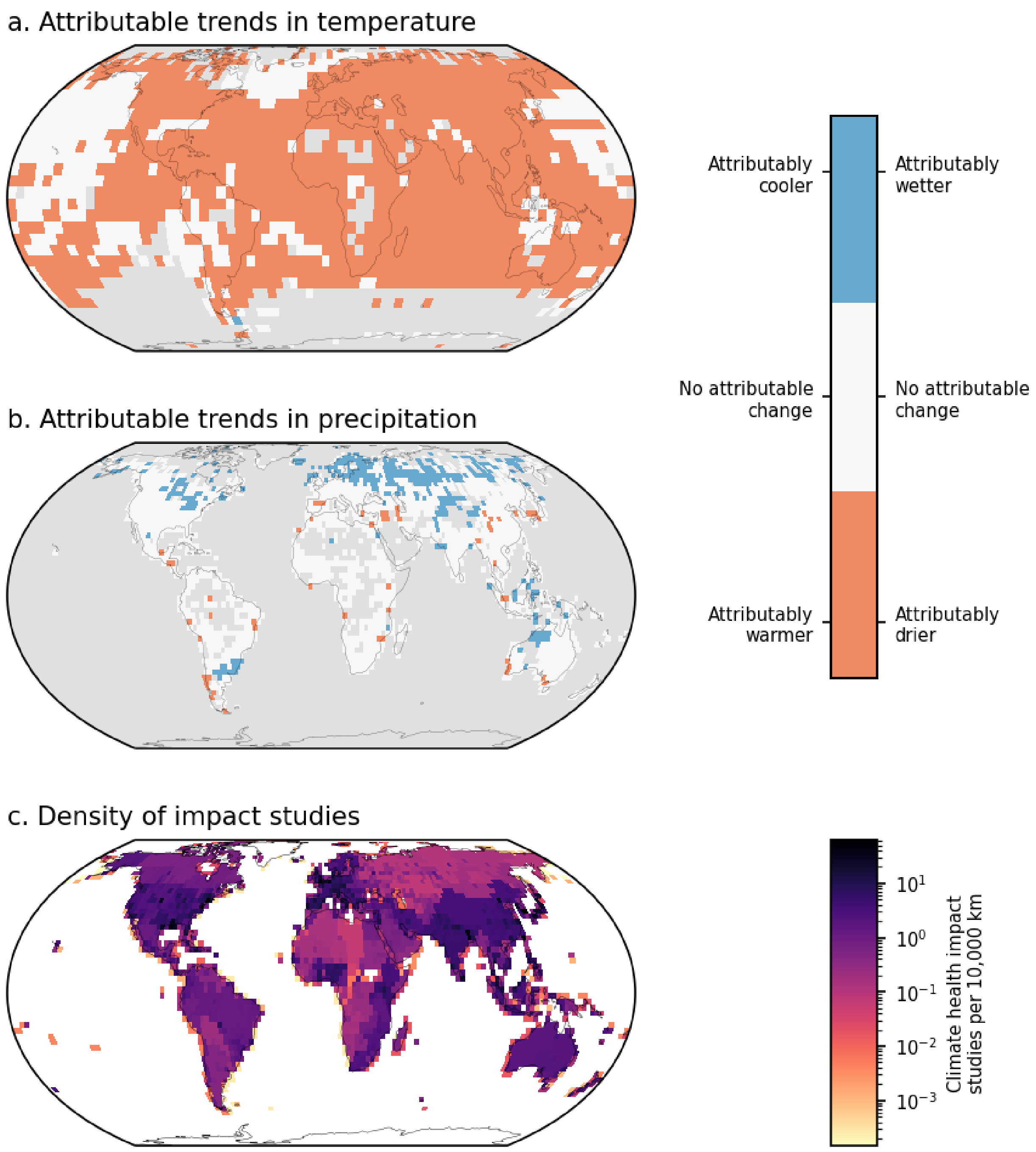

Figure 153.Attributable trends in temperature (top), precipitation (middle), and the number of climate health impact studies weighted per cell (bottom)

5.4: Political Engagement in Health and Climate Change

5.4.1. Government Engagement

Indicator authors

Dr Niheer Dasandi, Prof Slava Jankin, Dr Pete Lampard

Engagement in health and climate change in the United Nations General Assembly

Methods

To produce the measure of high-level political engagement with climate change and health in the UN General Assembly, a new dataset of UN General Debate statements was used, which is discussed below. The approach to using UNGD statements to produce the indicators here is based on the application of natural language processing to the corpus of UNGD statements. References to key search terms linked to (a) health, and (b) climate change are identified:

> **Health:** malaria, diarrhoea, infection, disease, diseases, sars, measles, pneumonia, epidemic, epidemics, pandemic, pandemics, epidemiology, healthcare, health, mortality, morbidity, nutrition, illness, illnesses, ncd, ncds, air pollution, nutrition, malnutrition, malnourishment, mental disorder, mental disorders, stunting.
>
> **Climate change:** climate change, changing climate, climate emergency, climate action, climate crisis, climate decay, global warming , green house, temperature, extreme weather, global environmental change, climate variability, greenhouse, greenhouse-gas, low carbon, ghge, ghges, renewable energy, carbon emission, carbon emissions, carbon dioxide, carbon-dioxide , co2 emission, co2 emissions, climate pollutant, climate pollutants, decarbonization, decarbonisation, carbon neutral, carbon-neutral, carbon neutrality, climate neutrality, net-zero, net zero.

These key terms have been updated to reflect the changing terminology used to discuss climate change. In order to produce an indicator of engagement with the intersection of climate change and health, this approach focused on whether any of the climate change related terms appeared immediately before or after any health terms in the GD statements. This was based on a search of the 25 words before and after a reference to a health-related term. The choice of 25-word window context corresponds to approximately half a paragraph of text. Given that UNGD statements are highly structured and methodically developed by governments over prolonged periods of time, it is assumed that half a paragraph of text around public health terms captures a sufficiently narrow context. The number of climate change term references were counted in these contexts to produce the measure of engagement with the link between health and climate change. A robustness analysis – varying the size of the context (5, 10, and 50 words) – was also undertaken. This substantively produced the same trends over time. A sample of the references produced by the search were also examined as an additional check to ensure that the references identified reflect engagement with the health impacts of climate change.

Data

This indicator draws on a new and updated dataset of GD statements: *the United Nations General Debate corpus*, in which the annual GD statements have been pre-processed and prepared for the application of natural language processing to the official English versions of the statements.[6] The dataset contains all the country speeches made in the UN General Debate between 1970 and 2021. **Table 86** presents summary of the data by year:

Year	General Debate statements	Total sentences	Total words
**1970**	70	11854	303791
**1971**	116	19901	508506
**1972**	125	21201	540994
**1973**	120	21450	536413
**1974**	129	22041	568739
**1975**	126	21365	534375
**1976**	134	23799	599949
**1977**	140	24799	606549
**1978**	141	25236	626163
**1979**	144	26462	654000
**1980**	149	27191	659225
**1981**	145	26063	633579
**1982**	147	23435	638691
**1983**	149	26803	643068
**1984**	150	27928	662654
**1985**	137	19258	592666
**1986**	149	19030	577525
**1987**	152	18336	563132
**1988**	154	18595	569493
**1989**	153	19440	574379
**1990**	156	17885	522197
**1991**	162	18552	538351
**1992**	167	18597	543138
**1993**	175	20165	587448
**1994**	178	19944	580530
**1995**	172	17870	536741
**1996**	181	18046	522699
**1997**	176	17701	514492
**1998**	181	18883	514836
**1999**	181	18529	531306
**2000**	178	16259	464312
**2001**	189	14748	414683
**2002**	188	13977	380481
**2003**	189	14716	399397
**2004**	192	14899	405290
**2005**	185	13012	353065
**2006**	193	14646	390476
**2007**	191	14586	387883
**2008**	192	14294	384881
**2009**	193	16029	423395
**2010**	189	14439	391954
**2011**	194	16293	429974
**2012**	195	16837	444519
**2013**	193	16400	440898
**2014**	194	15859	421947
**2015**	193	16129	436378
**2016**	194	15990	420155
**2017**	196	16806	439624
**2018**	196	16980	455205
**2019**	195	17526	466114
**2020**	193	15165	396548
**2021**	194	16675	442530
**2022**	193	17240	448117

Table 86. Summary information for UN General Debate Corpus.

The data was pre-processed for analysis by removing punctuation, symbols, numbers, stop words, and URLs. In addition, all tokens were normalised (lower-cased). All pre-processing and analysis was carried out in R using the "quanteda" package.[360]

Caveat

The search for climate change terms in the context of public health references is a proxy for the semantic linkage between the two sets of terms in GD statements. This approach produces a scalable and reproducible measure with a high degree of reliability that does not involve human judgement or subjective biases. However, there may be examples of governments referring to climate change and health but not the direct linkages between the two, which are included in the count; and there may be examples of governments discussing the health impacts of climate change in their UNGD statements, which are not included in the measure because the distance between the mention of the climate change term and the health term exceeds 25 words. Based on an analysing a sample of the speeches and references, such cases are relatively rare and do not have a significant bearing on the indicator or the trends uncovered.

It is also worth noting that the analysis here is based on a narrow range of search terms, which excludes reference to many of indirect links between climate change and health. A number of GD statements in this time period refer to such indirect connections, such as the effects of climate change on water and agriculture – however, these are not included here. Therefore, the results present a somewhat conservative estimate of high-level political engagement with the intersection of climate change and health. Future work in this area will consider engagement with these indirect links.

Future Form of Indicator

In the future, it is planned that this indicator will look more closely at the references to indirect links between climate change and health. For example, this would question the main ways in which governments view climate change impacting on health and whether this changes over time based on awareness of the multiple ways in which climate change and health are connected. Some of the references to the indirect links between climate change and health made in UNGD statements are highlighted in the main report.

Additional Information

Figure 154 shows the proportion of countries that made references to climate change, health and the intersection of both in the UNGD between 1970 and 2022. **Figure 155** shows the total number of references to health, climate change, and the intersection of the two between 1970 and 2022. **Figure 156** presents the total number of references to the intersection in UNGD statements between 1970 and 2022. **Figure 157** shows the proportion of countries that engage with the intersection of climate change and health between 1970 and 2022. The figures show the substantial increase in engagement with the health dimensions of climate change that occurred in 2020 and 2021, with a slight decline in 2022. In 2019 there were 109 separate references – which was significantly higher than in previous years – and in 2021 this more than tripled to 346 individual references to the intersection of climate change and health. In 2022, there is a decline in references to 245 individual references to the climate change-health intersection.

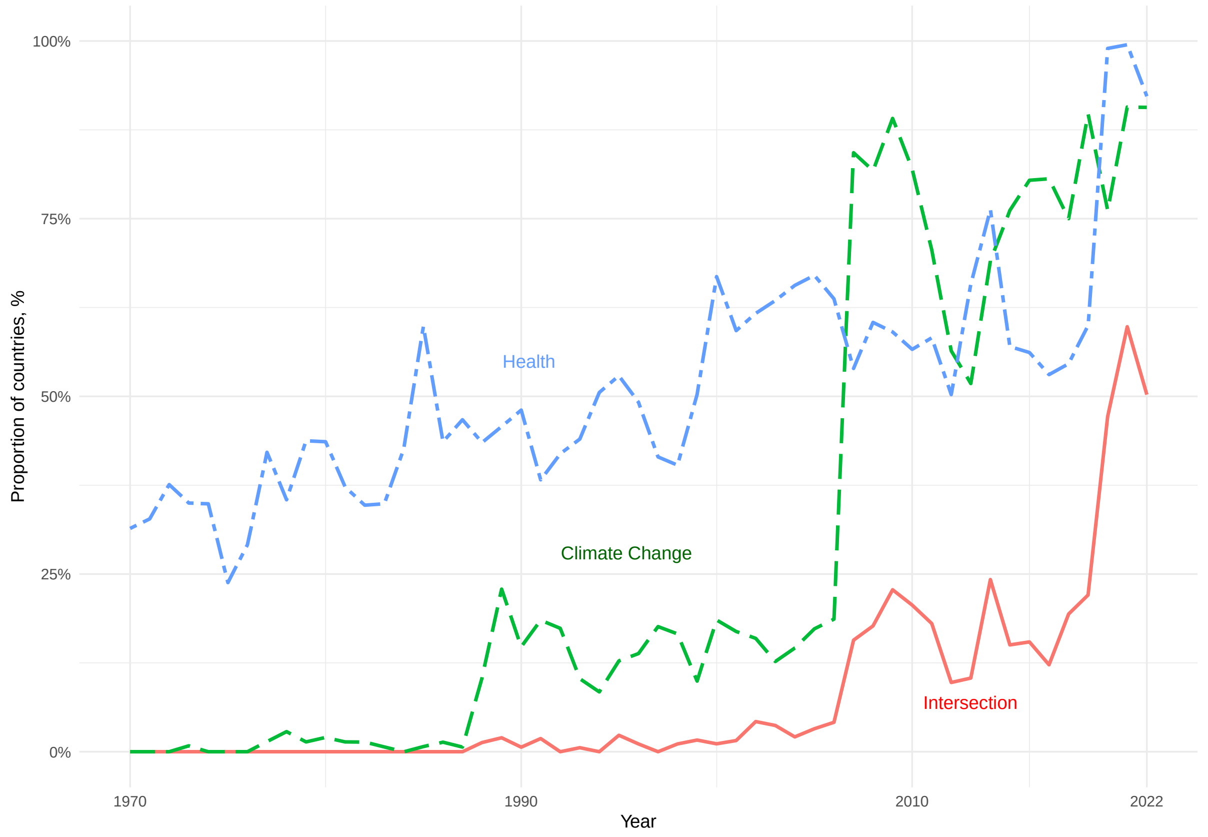

Figure 154: Proportion of countries referring to climate change, health, and the intersection between the two, UNGD, 1970-2022.

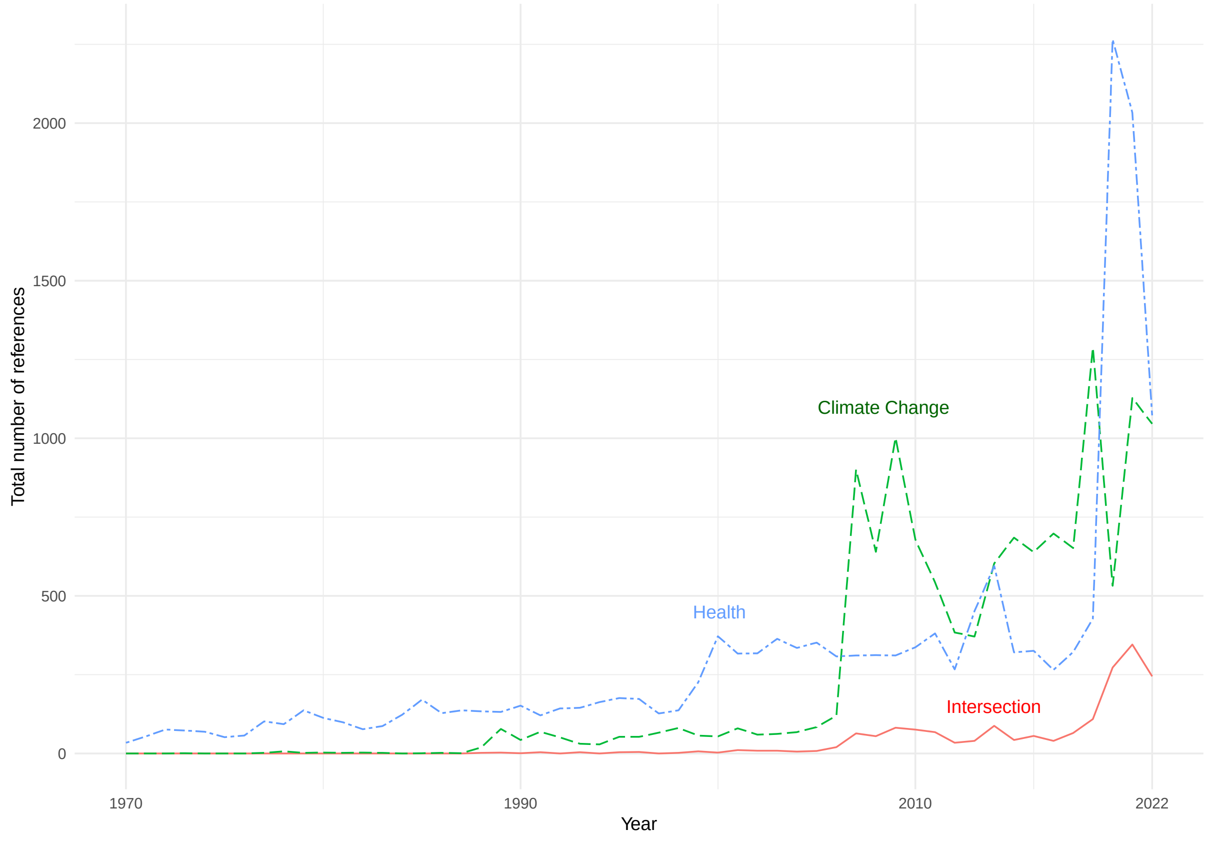

Figure 155. Total number of references to health, climate change, and intersection, 1970–2022

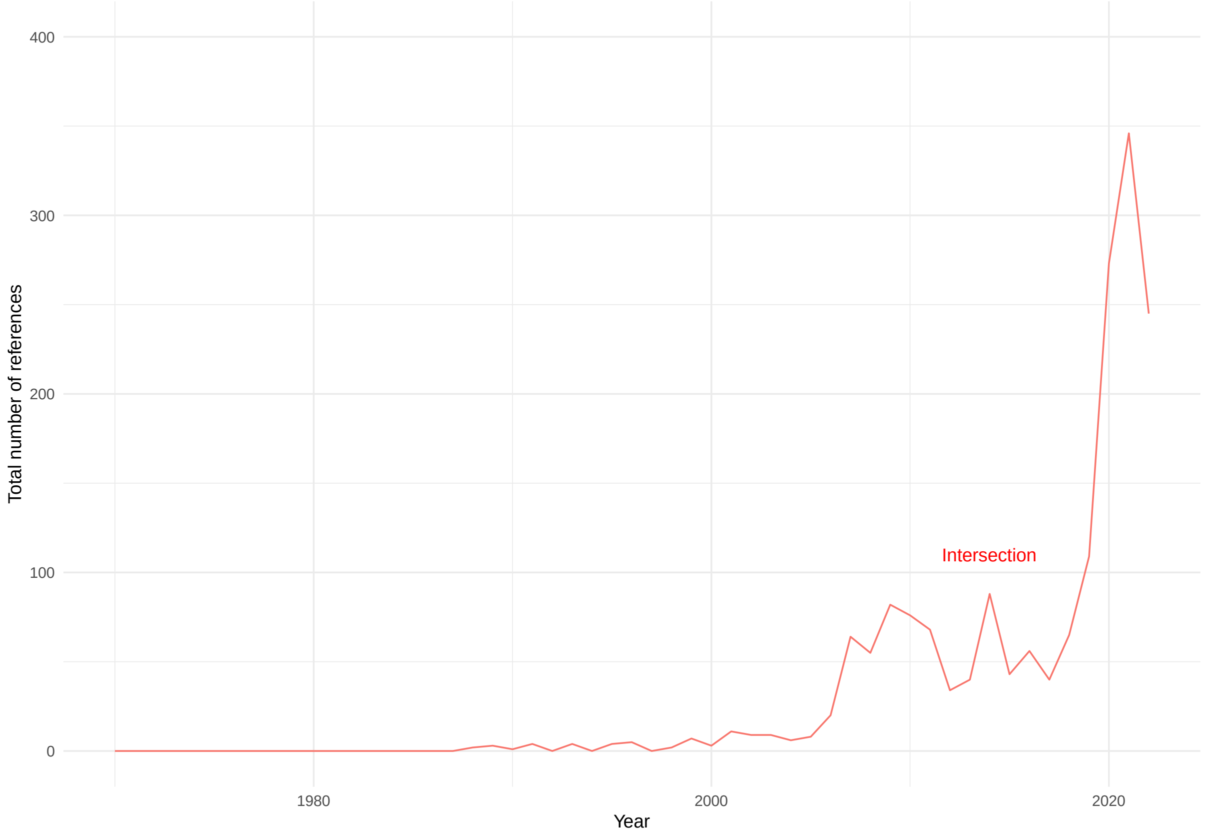

Figure 156. Total number of references to intersection, 1970–2022

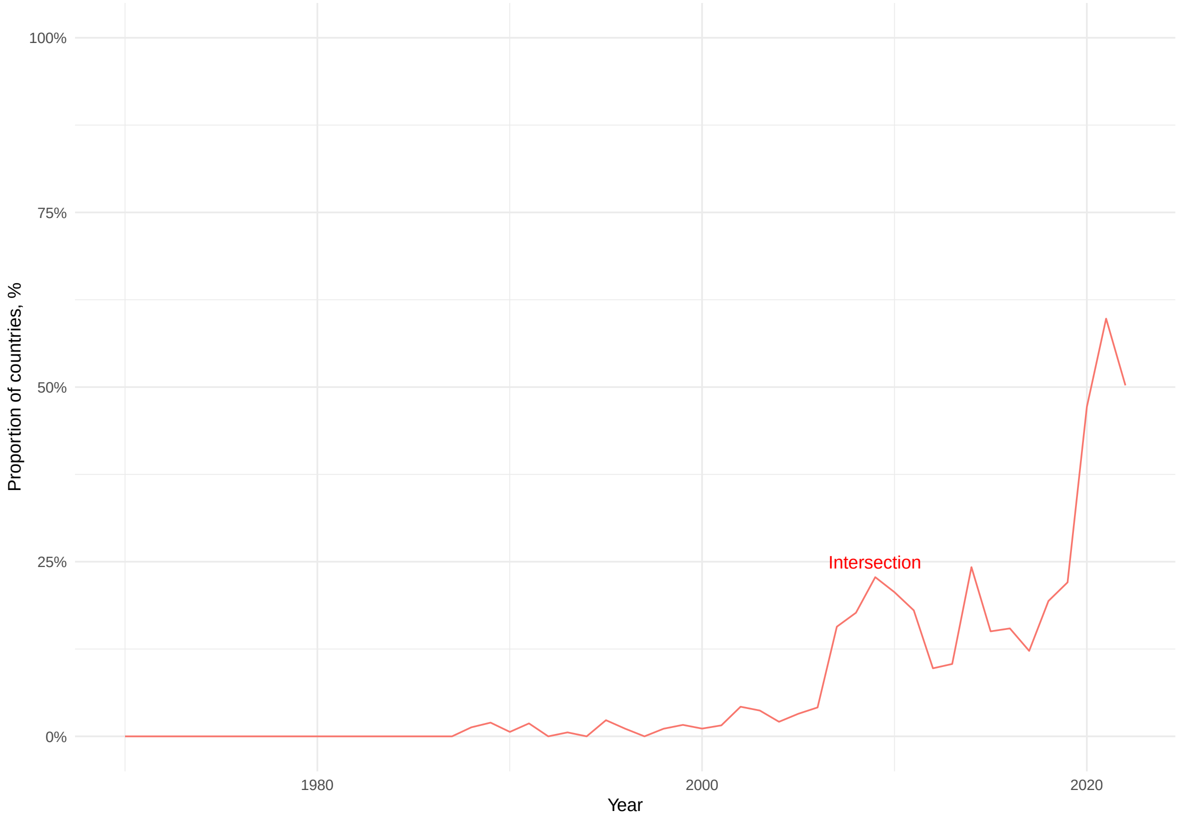

Figure 157. Proportion of countries referring to the intersection of health and climate, 1970–2022

There is growing awareness of the gendered impacts of climate change and health. This indicator considers the extent to which references to the health dimensions of climate change in countries UN General Debate statements engage with gender issues. This is undertaken by further examining the references to the intersection of climate change and health. Once all of the references to this intersection in UNGD statements for 1970–2022 were identified, additional search terms related to gender were used to identify which of the intersection references also engaged with gender issues. The gender-related search terms used were as follows: women, women's, maternal, inequality, inequalities, gender, empowerment, sex, sexual, violence, violent, girls, reproduction, reproductive. Hence, the analysis considers whether the 25 words of text identified in the primary search (for climate change and health terms) includes a reference to at least one of these gender-related keywords. **Figure 158** shows that only 2% of all references to the intersection of climate change and health also include a mention of gender. The figure shows that this is lower than in previous years, with the 2014 seeing 26% of all climate change-health references including a gender mention.

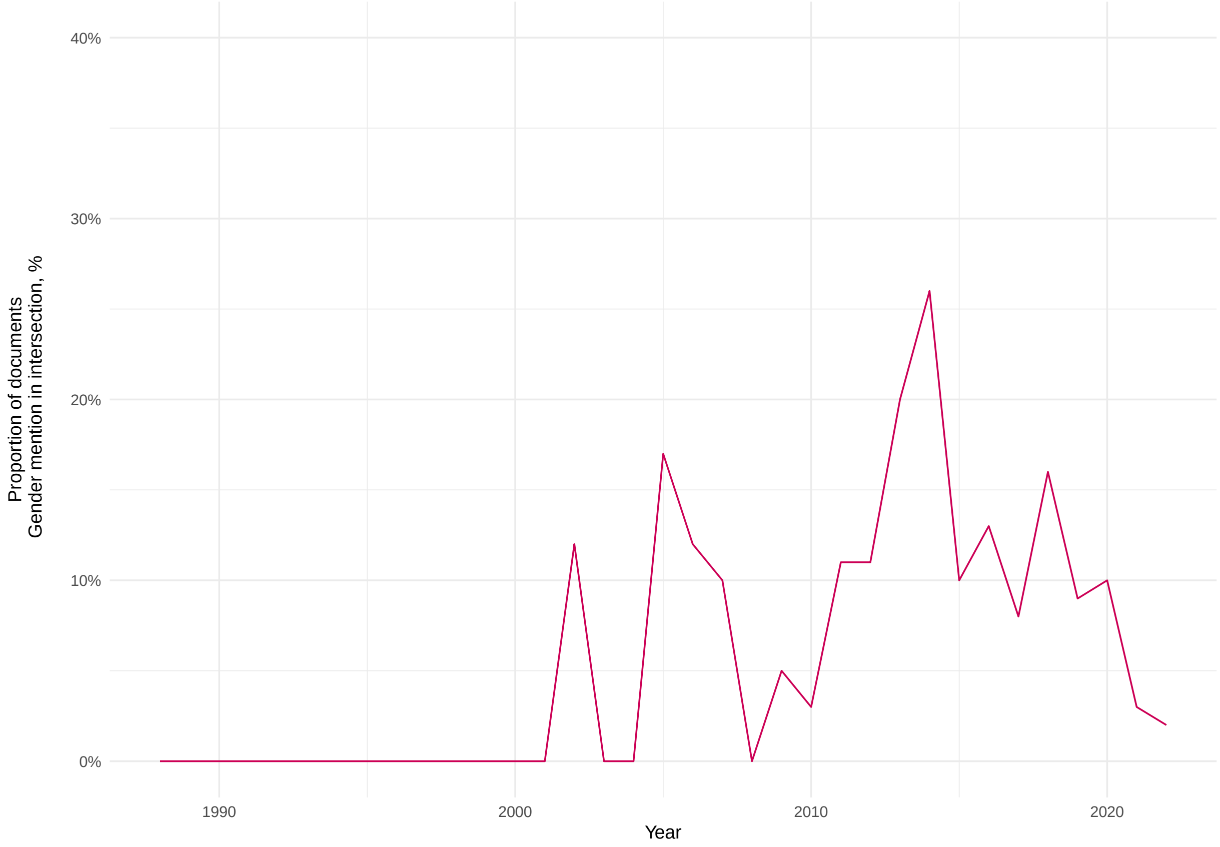

Figure 158. Proportion of references to the intersection of health and climate change that include a reference to gender, 1970–2022

Figure 159 below presents the proportion of countries that engage with the intersection of climate change and health by WHO region. There has been a significant increase in engagement with health and climate change across all regions in the past three years. In 2022, there is a slight decline across most regions compared to 2021, though at least 35% of countries in all the regions refer to the health dimensions of climate change in their 2022 UNGD statements. As in previous years there is especially high engagement from countries in the Western Pacific region, with 78% of countries referring to the intersection of climate change and health. The analysis finds that all countries in the South-East Asia and North American region refer to the intersection of health and climate change, though it is worth noting that these two regions consist of a small number of countries (9 and 2 respectively). It is also worth noting that the relatively higher level of political engagement by countries in the Western

Pacific is especially driven by the Small Island Development States (SIDS) in this region. The lowest engagement is by countries in the Eastern Mediterranean regions with 37% of countries in this region referring to the intersection of climate change and health.

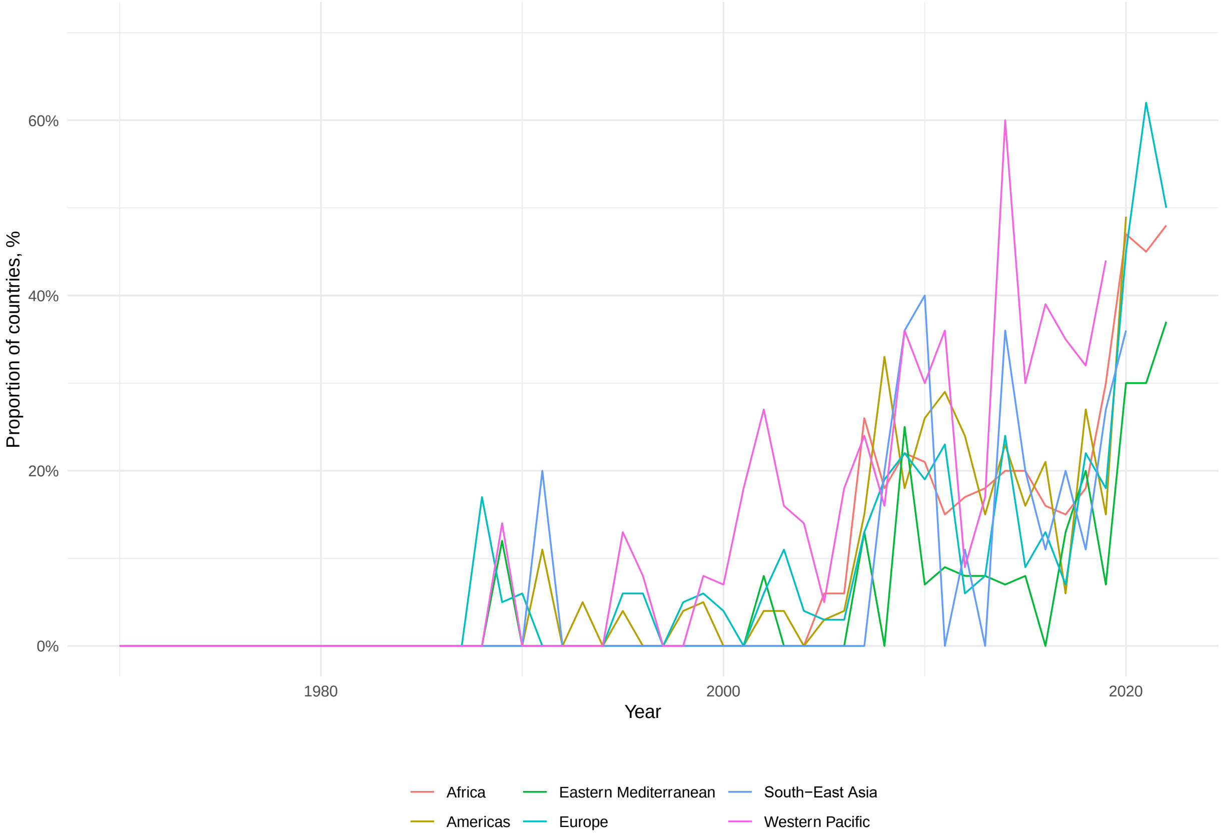

Figure 159. Proportion of countries referring to intersection of health and climate change by WHO region, 1970–2022

Figure 160, below, presents the total number of references to the climate change-health link between 1970 and 2022 by WHO region. The figure shows that the highest number of references to the intersection of climate change and health come from four regions: Africa, Europe, Latin America and the Caribbean, and the Western Pacific. In general, the figure suggests that there is lower engagement among countries in the Eastern Mediterranean, North America, and South-East Asia.

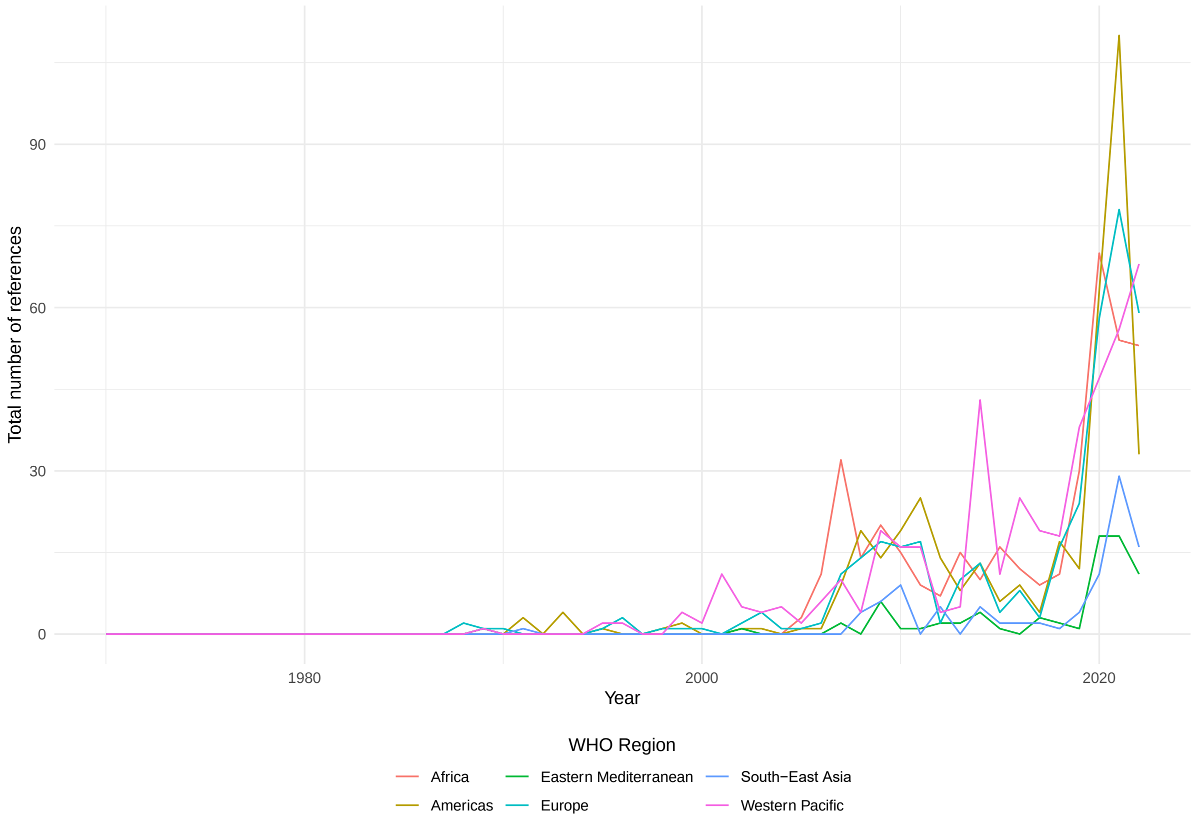

Figure 160. Total number of references to intersection by WHO region, 1970–2022

In addition to grouping countries by WHO region, countries are also considered by continental regions. This is provided in Figure 161 and Figure 162. As noted in previous years' reports, the SIDS have driven much of the engagement with the health impacts of climate change, as well as climate change more generally, in the UN General Assembly. As such, included is a separate SIDS grouping.

Figure 161 shows the proportion of countries that engage with the intersection of climate change and health based on these country groupings. Figure 162 shows the total number of references to the climate change-health intersection according to these groupings. Both figures demonstrate the high level of engagement with the climate change-health linkages by SIDS. It is worth noting that some of the regions (e.g., Northern America and Oceania) contain very few countries, and hence they deviate in engagement between 0 and 100% in different years.

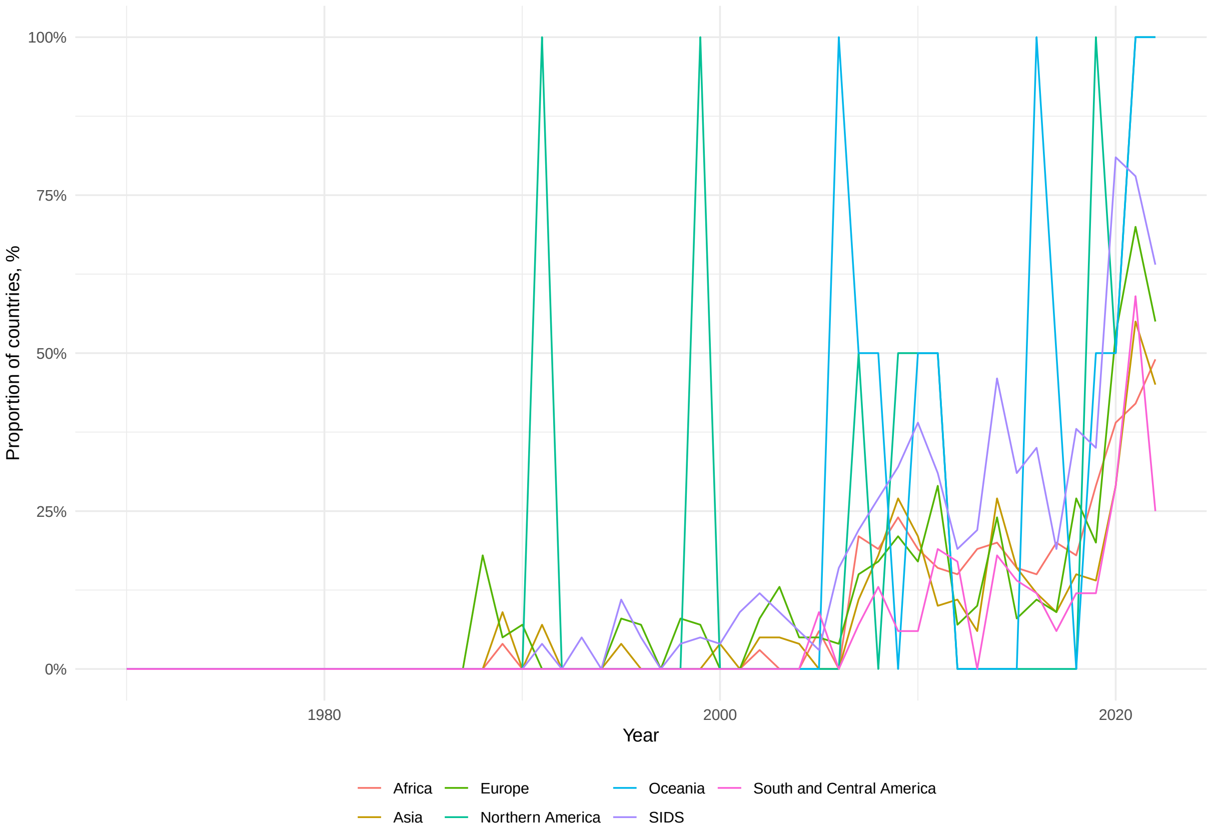

Figure 161. Proportion of countries referring to intersection of health and climate change by continental region, 1970–2022

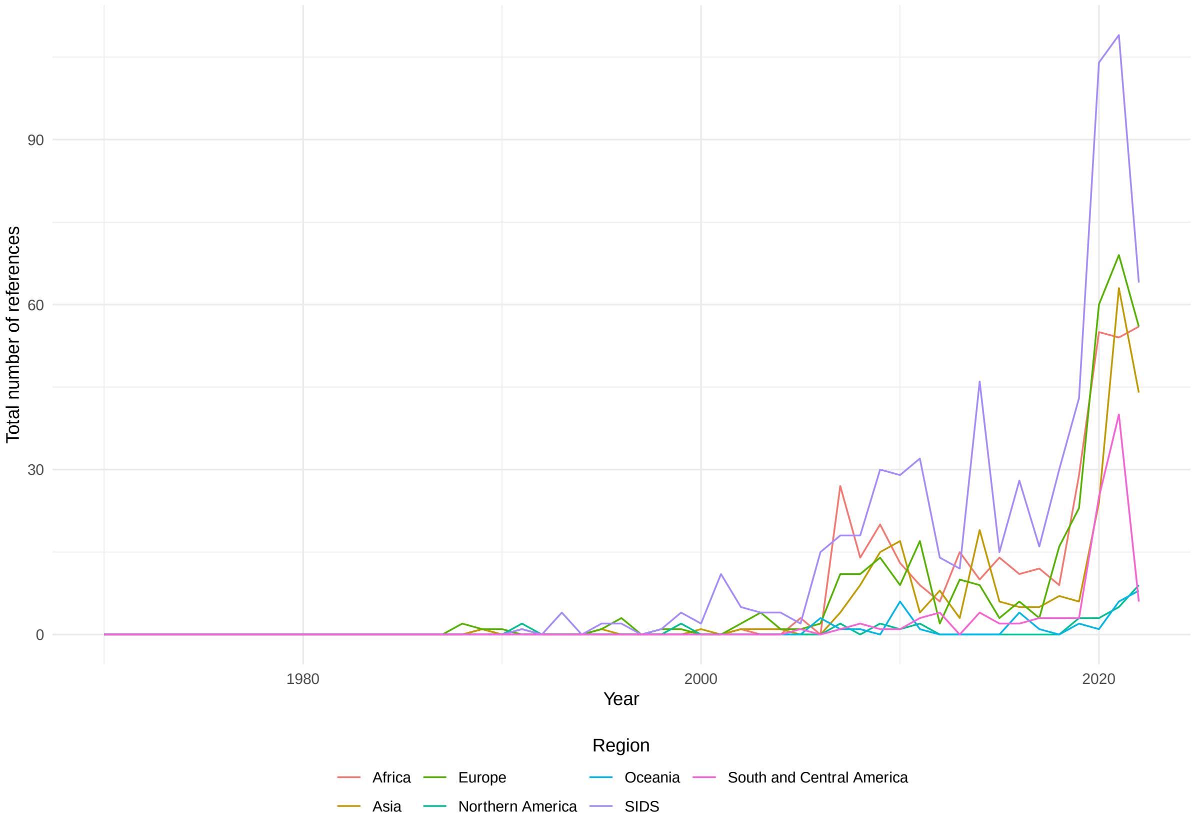

Figure 162. Total number of references to intersection by continental region, 1970–2022

Government engagement with the health dimensions of climate according to countries' Human Development Index (HDI) categories is also considered here. **Figure 163** shows the proportion of countries engaging with the intersection of climate change and health by HDI category, and **Figure 164** shows the total number of references by countries' HDI categories. Both figures show the significant increase in engagement across different HDI groupings since 2019 with a slight drop in 2022. The very high HDI category has the highest engagement with the intersection of health and climate change in 2022.

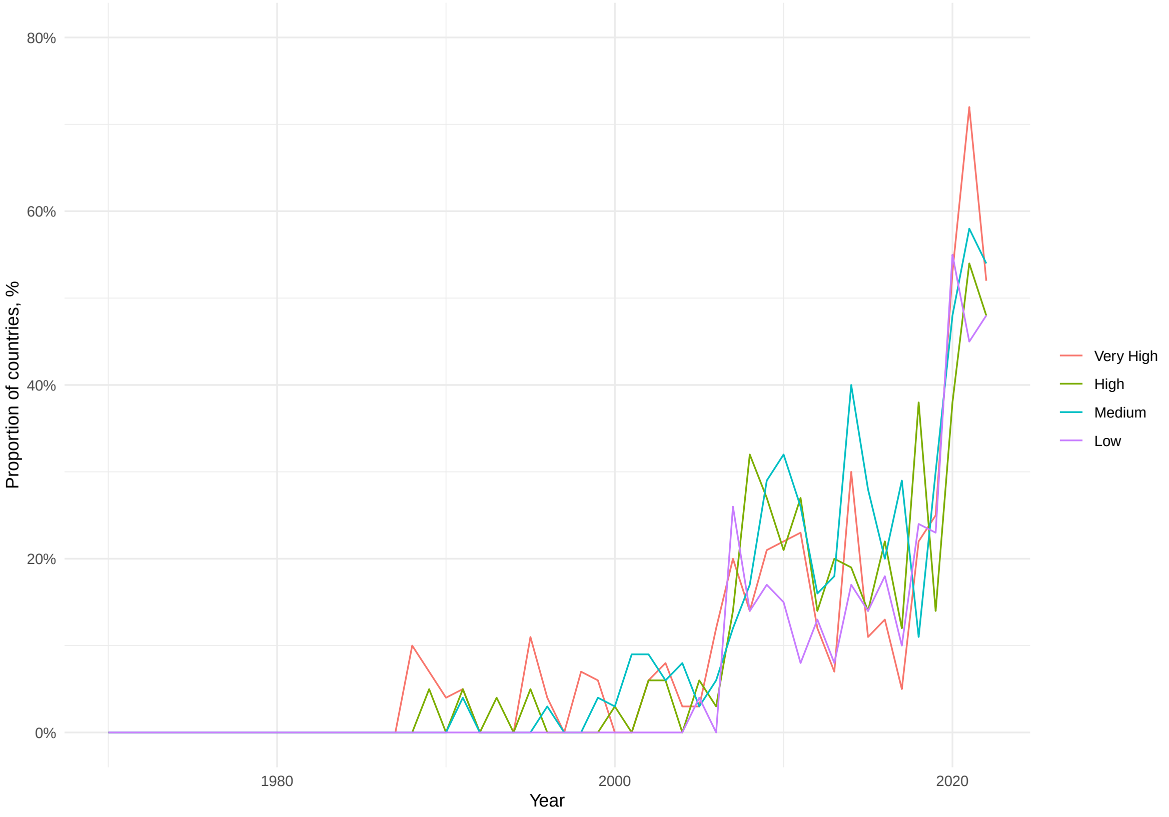

Figure 163. Proportion of countries referring to intersection of health and climate change by HDI categories, 1970–2022

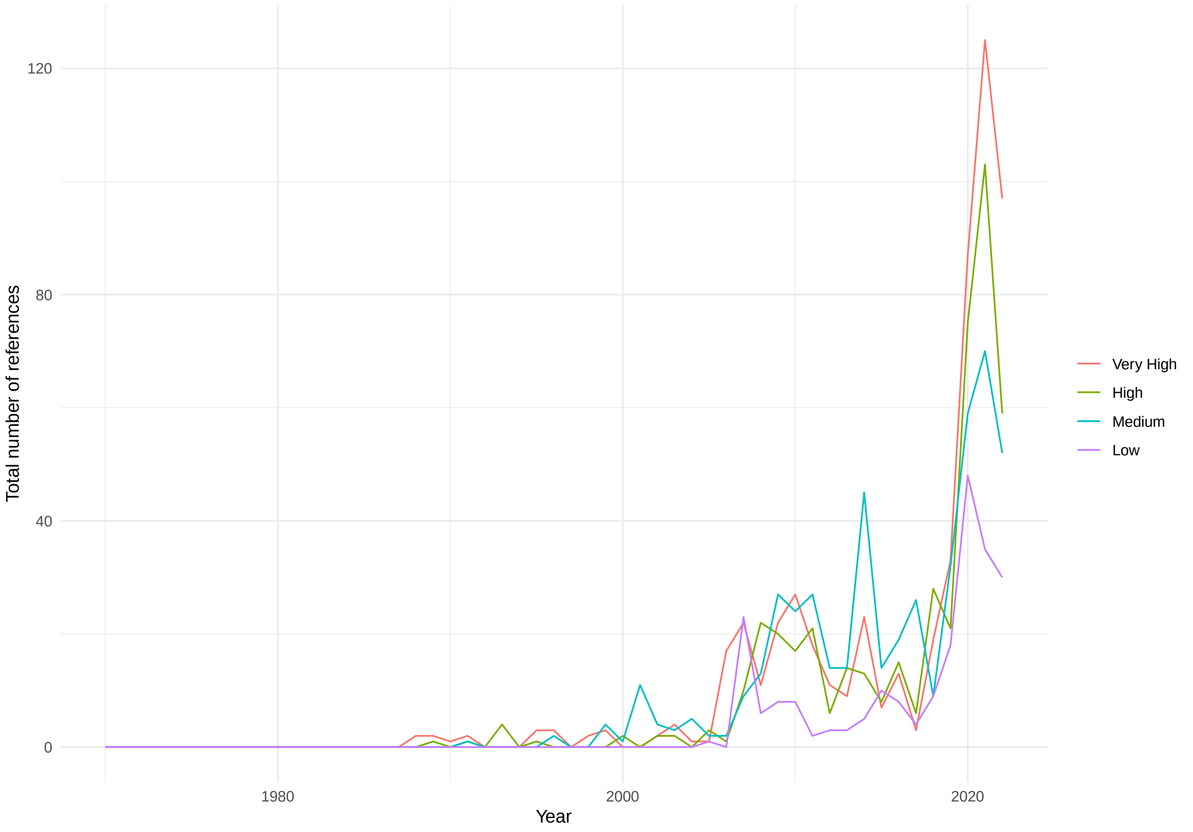

Figure 164. Total number of references to intersection by HDI categories, 1970–2022

Figure 165 below presents a world map, which shows the countries that refer to the intersection of climate change and health in their 2022 UNGD statements, and the number of individual references they make. The map shows that half of all countries mentioned the intersection of health and climate change in their 2022 address. The map also shows that despite the higher engagement, there is still evidence of a divide between high-income countries on the one side, and low- and middle income countries on the other side, though this divide is much smaller than in previous years. The latter (low- and middle-income countries) tend to engage more with climate change and health, particularly when the SIDS are included. Due to their size, the SIDS do not show up on the map. As previously noted, the SIDS tend to be highly represented among nations engaging with the health-climate change links.

Figure 166 and **Figure 167** present world maps, which show the countries that refer to public health and climate change respectively in their 2022 UNGD statements, as well as indicating the number of references made by each country. The figures demonstrate that there is considerable engagement with the issues of climate change and health separately. In 2022, 91% of countries referred to climate change and 92% of countries mentioned health in their UNGD statements, as can be seen in **Figure 166** and **Figure 167**. **Figure 166** and **Figure 167** show that as well as a much larger share of countries around the world discussing climate change and health in their GD statements compared to those discussing the intersection, there is also much deeper engagement with these two areas individually, in that countries tend to make a number of references to climate change and health in their GD statements.

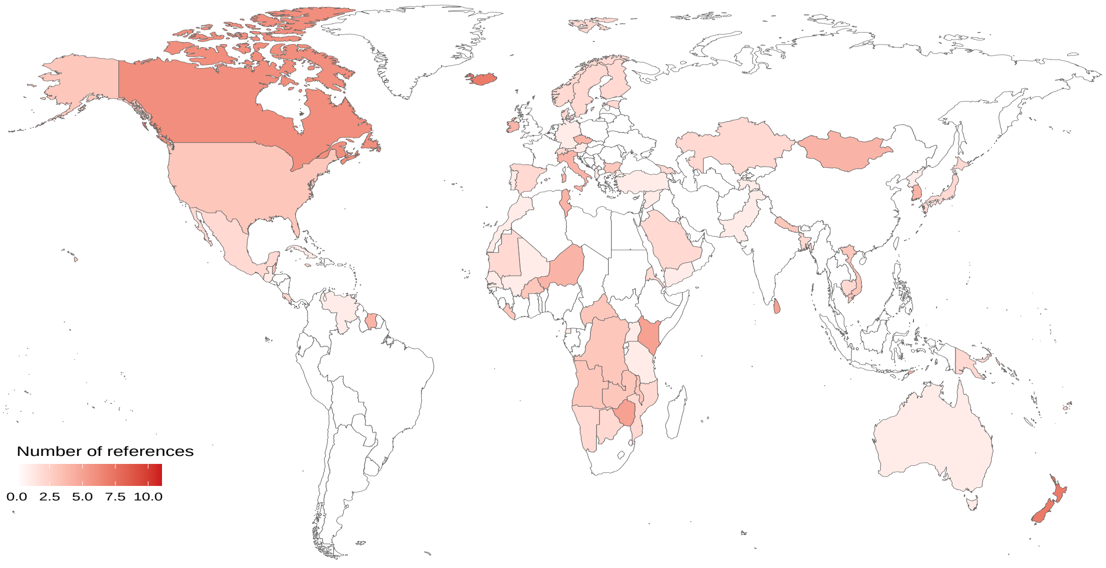

Figure 165. World map showing references to intersection of climate change and health, 2022

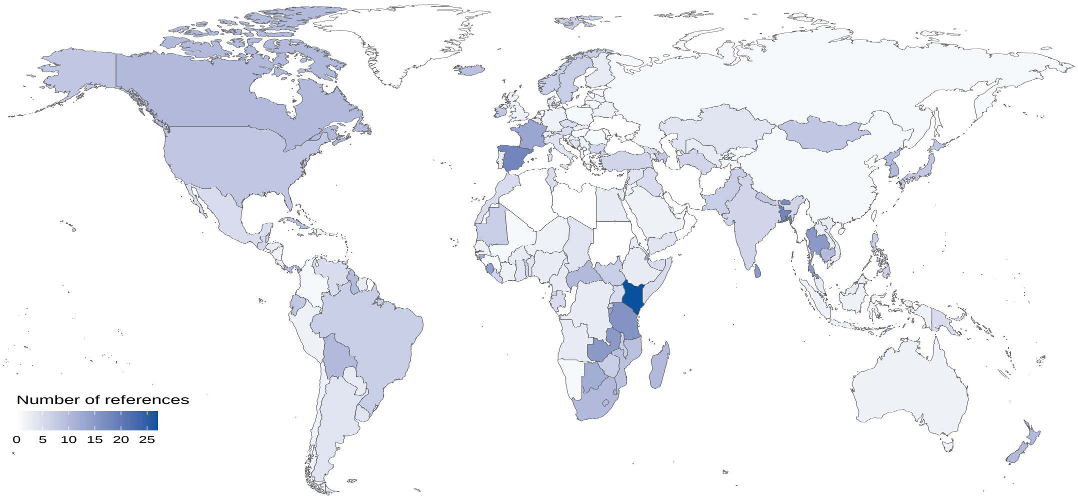

Figure 166. World map showing references to public health, 2022

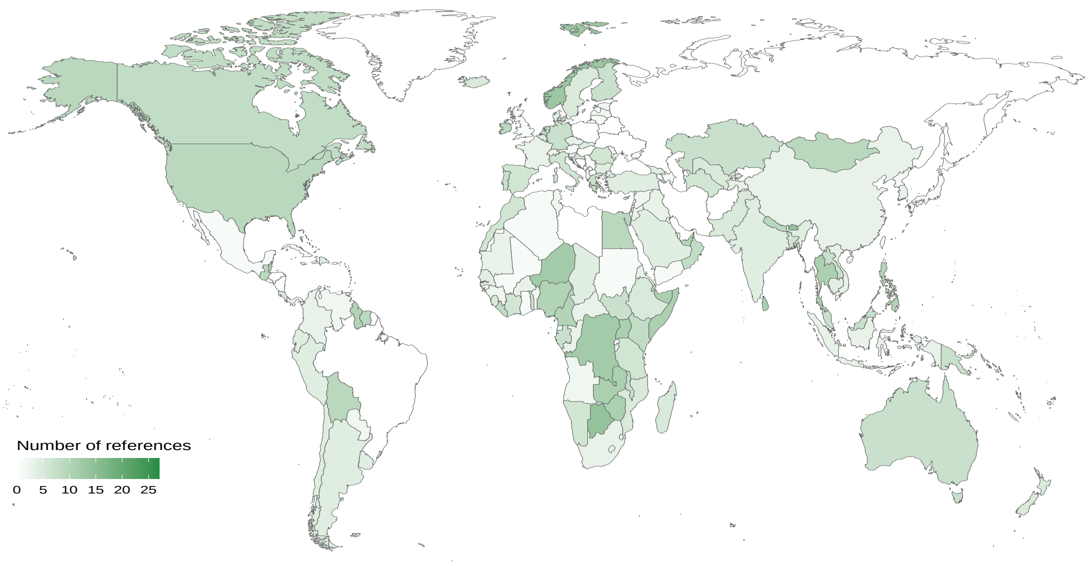

Figure 167. World map showing references to climate change, 2022

Engagement in health and climate change in the Nationally Determined Contributions

Methods

Under the Paris Agreement, Nationally Determined Contributions (NDCs) for each Party to the Agreement are communicated through the NDC registry. As a measure of engagement across climate change and health, and in particular of governments' appreciation of the health risks of climate change, all available first, second, third, and fourth NDCs (as of March 01, 2023) were analysed with respect to their inclusion of health-related terms. Here any update or entirely new NDC is regarded as a separate NDC. Analysis of first and second, and for some third and fourth, NDCs allows some indication of changes in climate-related health concerns over time.

This iteration of the report aligns with a change in the UNFCCC online archive of NDCs. Whereas in the last report NDCs were split between pre- and post-2020 NDCs, for this year's report these are categorised by the specific iteration per country. This means that some nations have four NDCs to their name while others may only have one. (More information on the specific countries and their number of iterations can be found below).

All NDCs were downloaded from the NDC registry. These were organised by iteration as per the UNFCCC registry spreadsheet (https://unfccc.int/NDCREG). From here the NDCs were uploaded into R and tokenised into sentence form using the *Tabulizer* package [361]. This enabled all NDCs to be broken up into dataframes of sentences, which could then be searched for health and climate change terms. By aggregating further, different resolutions with frequency of health-related terms, from country level to *Lancet* Countdown grouping, HDI classification, and WHO Region, could be achieved.

The two categories used in analysis (health-related terms and climate change-related terms) were developed iteratively (see Table 87 below). Other terms frequently used alongside health terms were also counted, again, developed through exploring the data or based on previous research around health and climate change (see Table 88 below).

category	keywords
health	health, illness
death	fatal*, mortal*, loss_of_life, death*
wellbeing	wellbeing
disease	infectious_disease*, disease*, morbid*, syndrome*
nutrition	malnutrition, starvation, undernutrition, nutrition
infectious disease	malaria, chikungunya, dengue, fever*, ebola, zika, leishmaniasis, leptospirosis, epidemic*, typhoid, vector*, aedes, mosquito*, pandemic*
psychological	emotion*, psychology*, mental_health
medical	medic*, hospital, hospitali*ation, patients, emergency_department, A&E, diagnos*, clinical
heat	heat_stress, heat_disorder*
injury	injur*
covid	covid, corona*, sars_cov_II, sars_cov_2

Table 87. Health keywords.

category	keywords
adaptation	adapt*
mitigation	mitig*
co-benefits	co_benefit*, win_win, secondary benefit*
trade off	trade_off*
gender	gender*, wom*n*, reproduct*, maternal
sector	sector*, infrastructure*
knowledge	university*, train*, educat*, research*
rights	human_right*, *equalit*, *equit*, rights
one health	one_health, healthy_island

Table 88. Context keywords.

Data

Table 89 shows the number of NDCs available by iteration. Figure 168 demonstrates the proportion of these NDCs by *Lancet* Countdown grouping. Box 2 demonstrates how many iterations each UN member state has uploaded to the UNFCCC website. Several NDCs were in either French or Spanish and were therefore translated using Google Translate. While each document covered broadly the same material (all had mitigation strategies, most had adaptation sections, coverage of national circumstances, and an account of fairness and ambition within the NDC), most were different in presentation, making extraction of text difficult in some instances. For example, some NDCs were screenshots of pages, which meant Adobe DC was used for its Optical Character Recognition capacity, converting image into text.

	count
**First**	191
**Second**	163
**Third**	28
**Fourth**	2

Table 89. NDC count per iteration.

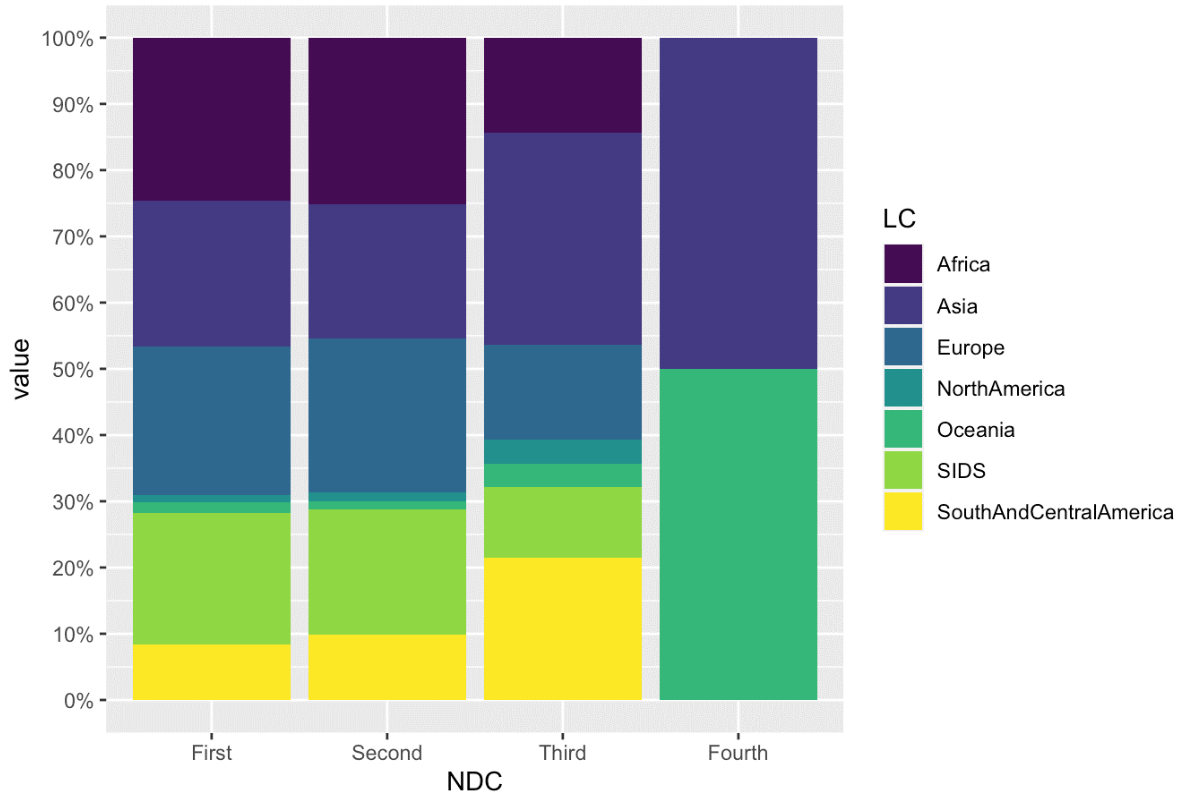

Figure 168. Proportion of NDCs per iteration by Lancet Countdown grouping.

Afghanistan: 1, Albania: 2, Algeria: 1, Andorra: 3, Angola: 2, Antigua and Barbuda: 2, Argentina: 3, Armenia: 2, Australia: 4, Austria: 2, Azerbaijan: 1, Bahamas: 2, Bahrain: 2, Bangladesh: 3, Barbados: 2, Belarus: 2, Belgium: 2, Belize: 2, Benin: 2, Bhutan: 2, Bolivarian Republic of Venezuela: 1, Bolivia (Plurinational State of): 2, Bosnia and Herzegovina: 2, Botswana: 1, Brazil: 3, Brunei Darussalam: 1, Bulgaria: 2, Burkina Faso: 2, Burundi: 2, Cabo Verde: 2, Cambodia: 12, Cameroon: 2, Canada: 3, Central African Republic: 2, Chad: 2, Chile: 2, China: 2, Colombia: 2, Comoros: 2, Congo: 2, Cook Islands: 1, Costa Rica: 3, Côte d'Ivoire: 2, Croatia: 2, Cuba: 2, Cyprus: 2, Czechia: 2, Democratic People's Republic of Korea: 2, Democratic Republic of the Congo: 2, Denmark: 2, Djibouti: 1, Dominica: 2, Dominican Republic: 2, Ecuador: 1, Egypt: 2, El Salvador: 3, Equatorial Guinea: 2, Eritrea: 1, Estonia: 2, Eswatini: 2, Ethiopia: 2, Fiji: 2, Finland: 2, France: 2, Gabon: 2, Gambia: 2, Georgia: 2, Germany: 2, Ghana: 2, Greece: 2, Grenada: 2, Guatemala: 3, Guinea: 2, Guinea-Bissau: 2, Guyana: 1, Haiti: 2, Honduras: 2, Hungary: 2, Iceland: 2, India: 2, Indonesia: 3, Iraq: 1, Ireland: 2, Israel: 2, Italy: 2, Jamaica: 2, Japan: 4, Jordan: 2, Kazakhstan: 1, Kenya: 2, Kiribati: 1, Kuwait: 2, Kyrgyzstan: 2, Lao People's Democratic Republic: 2, Latvia: 2, Lebanon: 2, Lesotho: 2, Liberia: 2, Liechtenstein: 1, Lithuania: 2, Luxembourg: 2, Madagascar: 1, Malawi: 2, Malaysia: 2, Maldives: 2, Mali: 1, Malta: 2, Marshall Islands: 3, Mauritania: 2, Mauritius: 2, Mexico: 3, Micronesia (Federated States of): 2, Monaco: 2, Mongolia: 2, Montenegro: 2, Morocco: 2, Mozambique: 2, Myanmar: 2, Namibia: 2, Nauru: 2, Nepal: 2, Netherlands: 2, New Zealand: 2, Nicaragua: 2, Niger: 2, Nigeria: 3, Niue: 1, North Macedonia: 2, Norway: 3, Occupied Palestinian territory: 1, Oman: 12, Pakistan: 3, Palau: 1, Panama: 2, Papua New Guinea: 2, Paraguay: 2, Peru: 2, Philippines: 1, Poland: 2, Portugal: 2, Qatar: 2, Republic of Korea: 3, Republic of Moldova: 2, Romania: 2, Russian Federation: 1, Rwanda: 2, Saint Kitts and Nevis: 2, Saint Lucia: 2, Saint Vincent and the Grenadines: 1, Samoa: 2, San Marino: 1, Sao Tome and Principe: 2, Saudi Arabia: 2, Senegal: 1, Serbia: 2, Seychelles: 2, Sierra Leone: 2, Singapore: 3, Slovakia: 2, Slovenia: 2, Solomon Islands: 2, Somalia: 2, South Africa: 2, South Sudan: 2, Spain: 2, Sri Lanka: 3, State of Palestine: 2, Sudan: 3, Suriname: 2, Sweden: 2, Switzerland: 3, Syrian Arab Republic: 1, Tajikistan: 2, Thailand: 3, Timor-Leste: 2, Togo: 2, Tonga: 2, Trinidad and Tobago: 1, Tunisia: 2, Türkiye: 1, Turkmenistan: 1, Tuvalu: 1, Uganda: 3, Ukraine: 2, United Arab Emirates: 3, United Kingdom: 2, United

Box 2. NDC count per member state.

Caveats

There may be cases within the NDCs where the discussion of health and climate change is split over two or more sentences, and where key identifiers for the health-related category are only implied.

Future form of indicator

This indicator uses the data from all available first NDCs held on the UNFCCC registry (https://www4.unfccc.int/sites/NDCStaging/Pages/All.aspx) as of March 1st 2023. Future reports will report on NDCs added after this date, where relevant, either as a full indicator on as a smaller section in the report.

Future research could also provide a full document analysis looking at the role of health in the NDCs, but this would require further resourcing.

Additional analysis

Given how few fourth NDCs there are, additional analyses will be limited to the first, second and third NDCs.

Types of health keywords present

Table 90 shows how different health keywords are distributed across the iterations of NDCs. It demonstrates that health mentions as a whole increase from the first NDCs (73%) to the second (94%). Third NDCs show a reduction once more in health mentions (79%).

The second and third iteration demonstrated a higher proportion of NDCs mentioning keywords relating to deaths, nutrition, infectious diseases, psychological or emotional health, and heat-related issues. Of these, the biggest increase appears to be in references to infectious disease keywords (from 28% to 80%).

**keywords**	**First (n=191)**	**Second (n=163)**	**Third (n=28)**
All health	73%	94%	79%
health	70%	92%	75%
deaths	18%	36%	36%
wellbeing	21%	39%	50%
disease	40%	56%	36%
nutrition	20%	40%	36%
infectious	28%	80%	64%
psychological	4%	19%	29%
medical	17%	43%	39%
heat	2%	13%	14%
injury	5%	9%	11%
covid	1%	74%	64%

Table 90. Health related terms and their distribution across NDC iterations. Note: COVID19-related terms are not included in the "all health" terms category.

Lancet *Countdown grouping*

By *Lancet* Countdown grouping (see Figure 169), the South and Central American region and the African region demonstrate the highest proportion of first NDCs making reference to health in some way (100% and 98% respectively). This is followed by the Asian region (88%) and the SIDS nations

(84%). The European (14%), Oceanic (1 out of 3, 33%), and North American (1 out of 2, 50%) regions have the lowest proportion of NDCs mentioning health in the first round. Each of these, besides the Oceanic region, shows significant improvement in the second round, with the North American region, South and Central American region, and SIDS nations all at 100% of NDCs mentioning health, and the African region once again with 98%. 92% of NDCs representing the European region – a dramatic increase, up from 14% – mention a health-related term. 100% of North American NDCs also mention health in the second round, though this is only made up of two NDCs. While one out of three Oceanic first NDCs mentioned health, neither of the two second round NDCs mention health in any way.

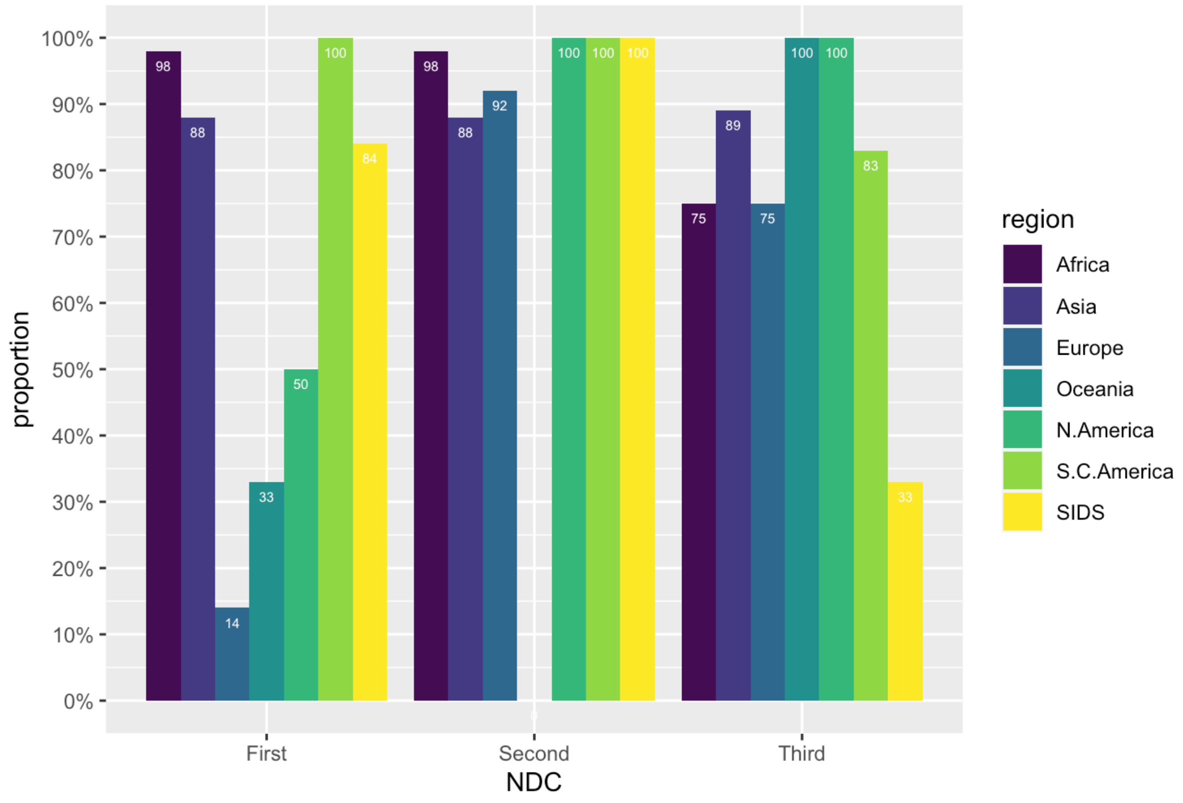

Figure 169. Proportion of NDCs per Lancet Countdown grouping mentioning a health keyword across iterations.

The Figures below also show the proportion of NDCs from each of these groupings, in each iteration, using keywords from particular health keyword categories.

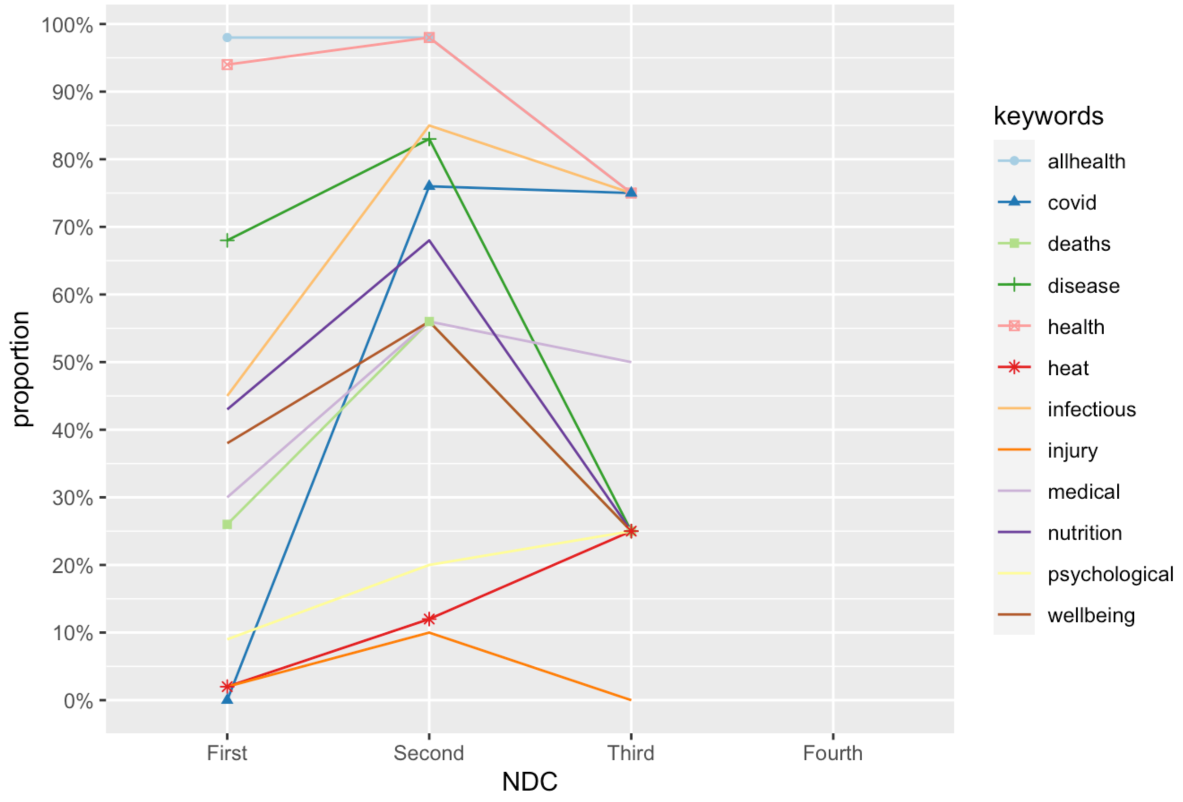

Figure 170. Proportion of NDCs in Africa Lancet Countdown grouping using different health terms, across iterations (first NDC n=47, second NDC n=41, third NDC n=4, fourth NDC n=0)

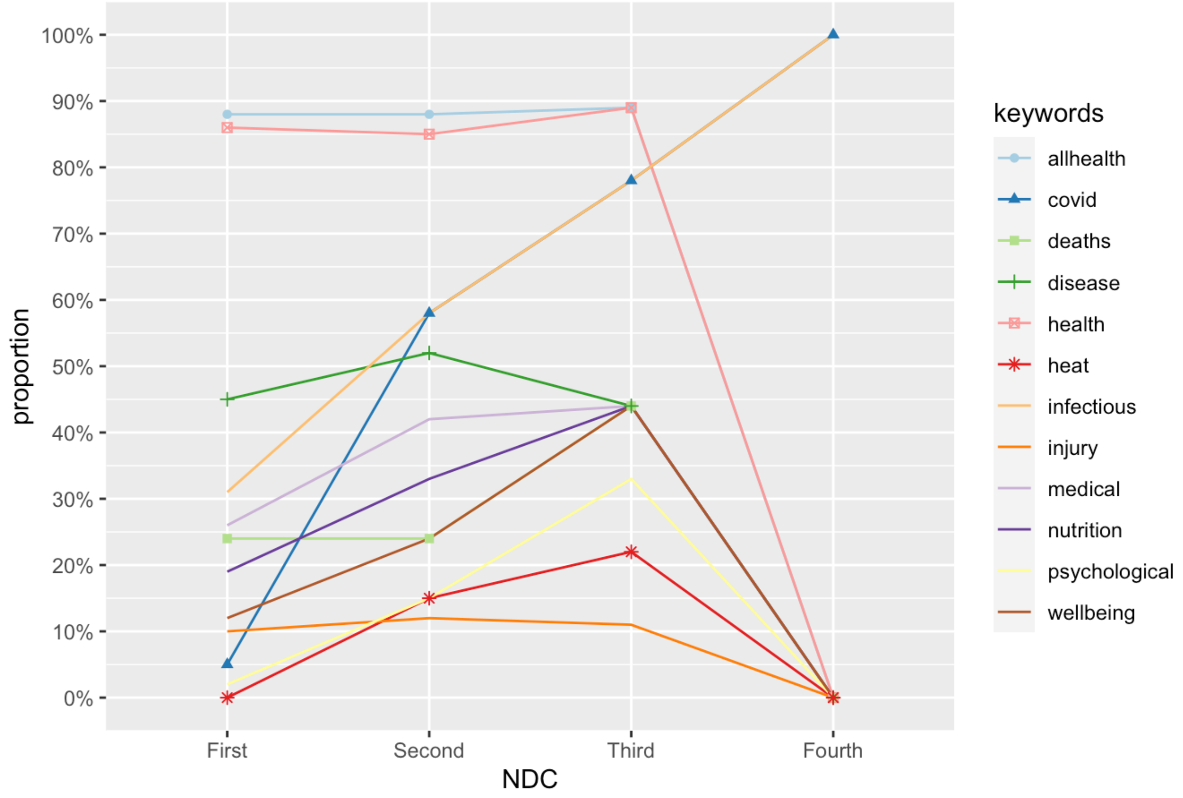

Figure 171. Proportion of NDCs in Asia Lancet Countdown grouping using different health terms, across iterations (first NDC n=42, second NDC n=33, third NDC n=9, fourth NDC n=1)

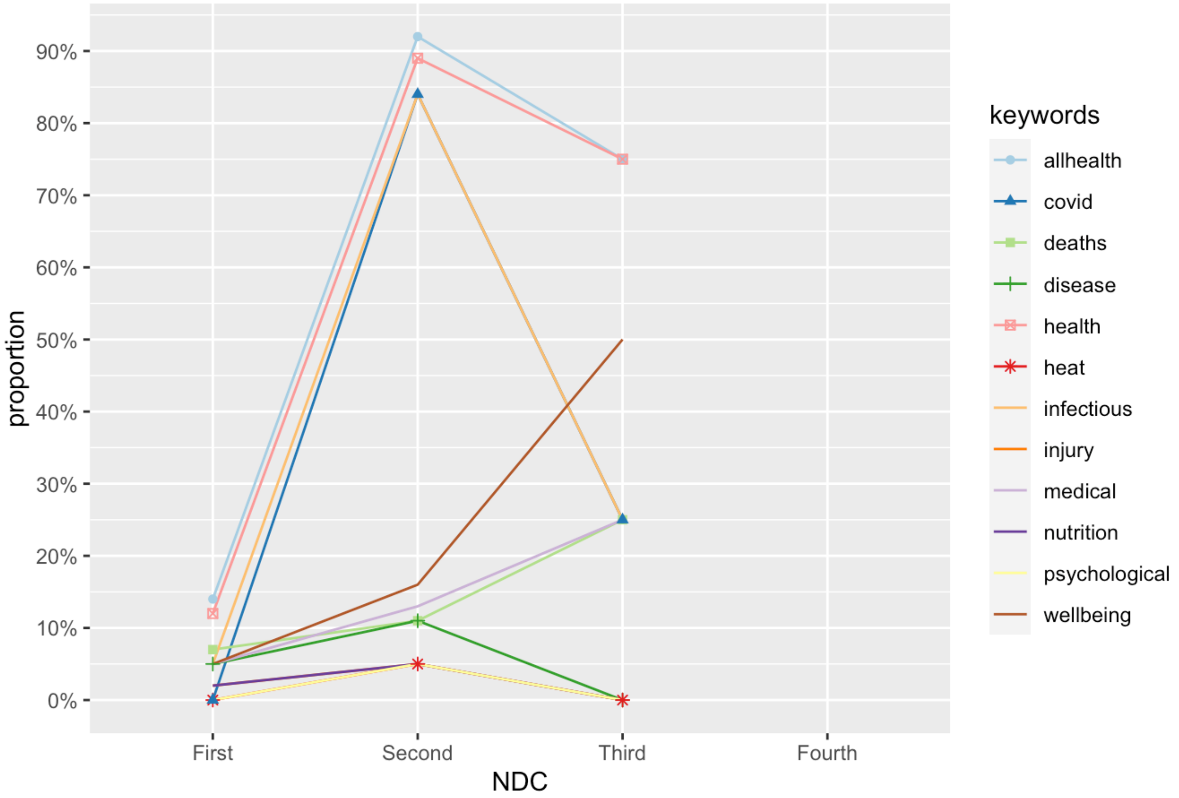

Figure 172. Proportion of NDCs in Europe Lancet Countdown grouping using different health terms, across iterations (first NDC n=43, second NDC n=38, third NDC n=4, fourth NDC n=0)

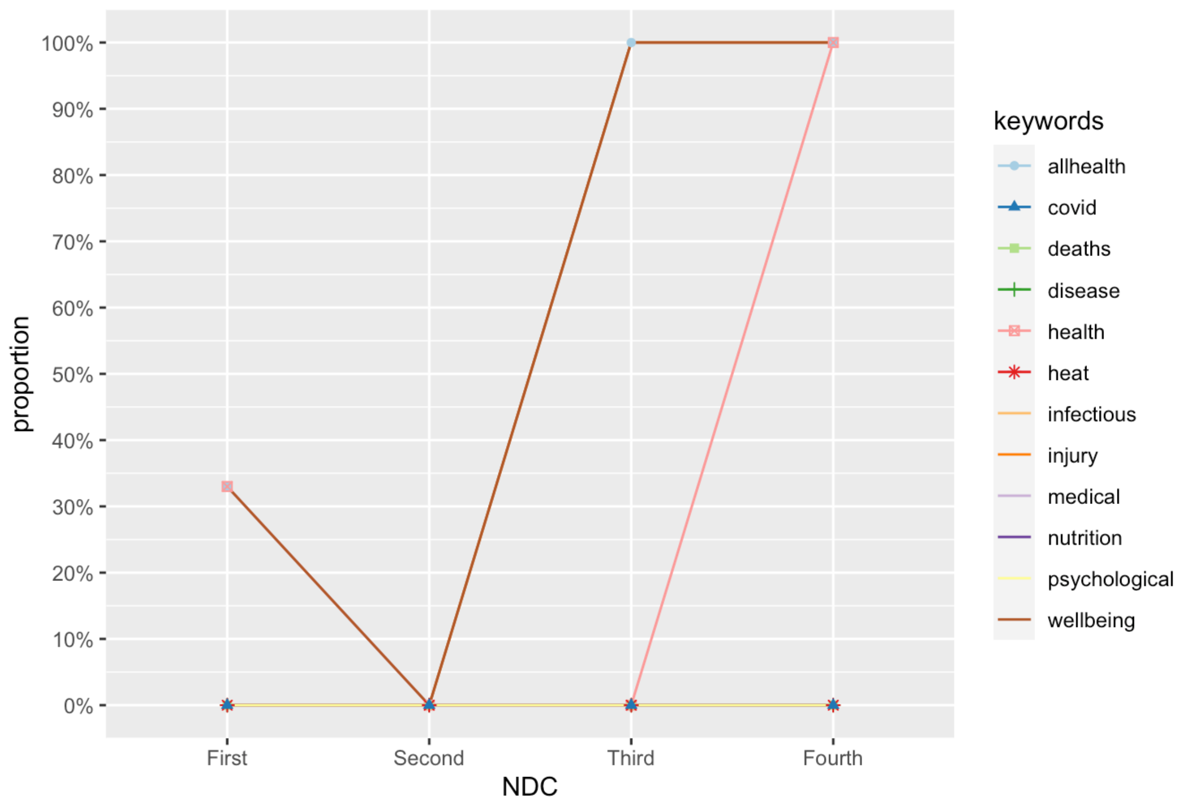

Figure 173. Proportion of NDCs in Oceania Lancet Countdown grouping using different health terms, across iterations (first NDC n=3, second NDC n=2, third NDC n=1, fourth NDC n=1)

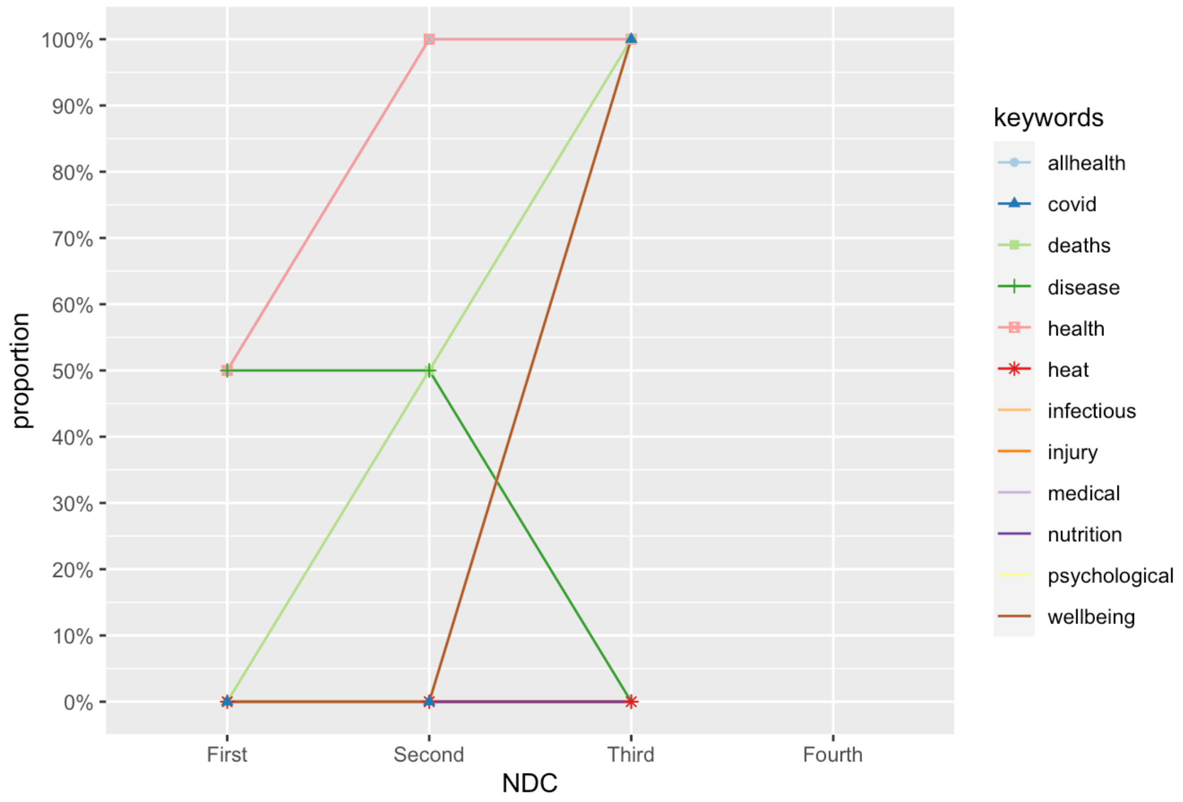

Figure 174. Proportion of NDCs in North American Lancet Countdown grouping using different health terms, across iterations (first NDC n=2, second NDC n=2, third NDC n=1, fourth NDC n=0)

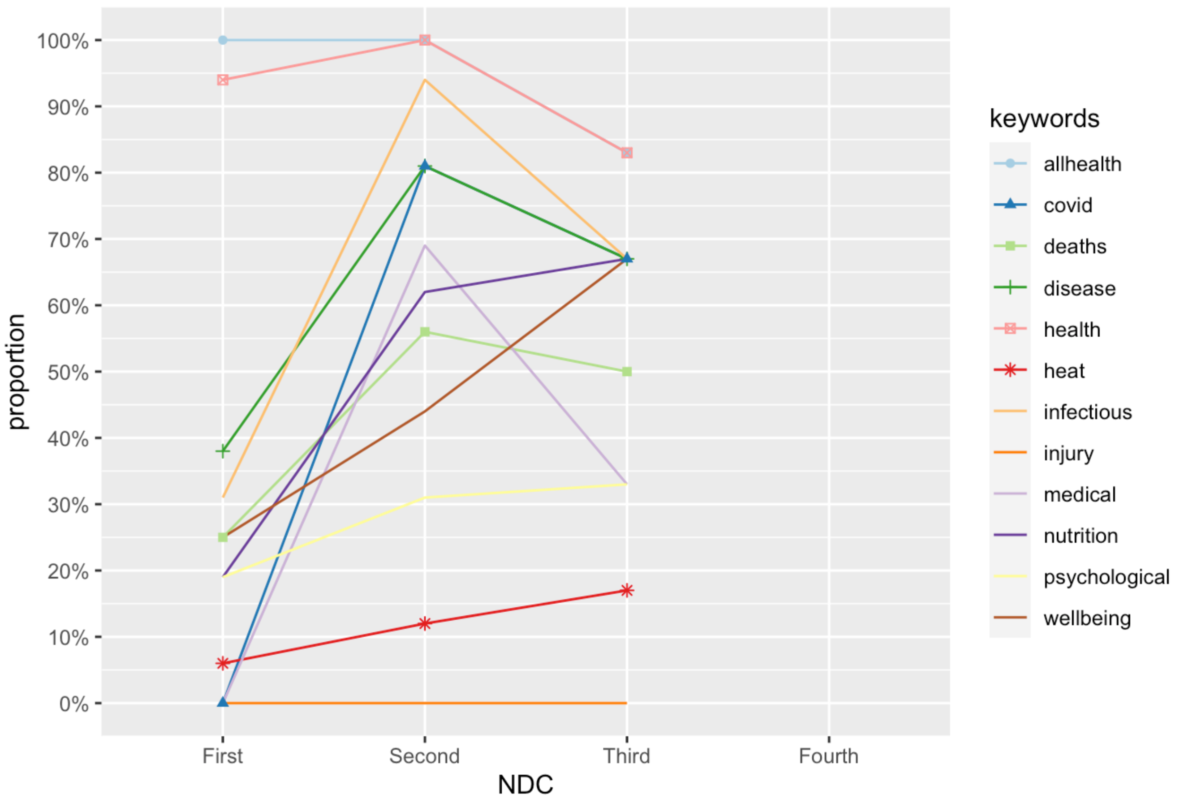

Figure 175. Proportion of NDCs in South and Central America Lancet Countdown grouping using different health terms, across iterations (first NDC n=16, second NDC n=16, third NDC n=6, fourth NDC n=0)

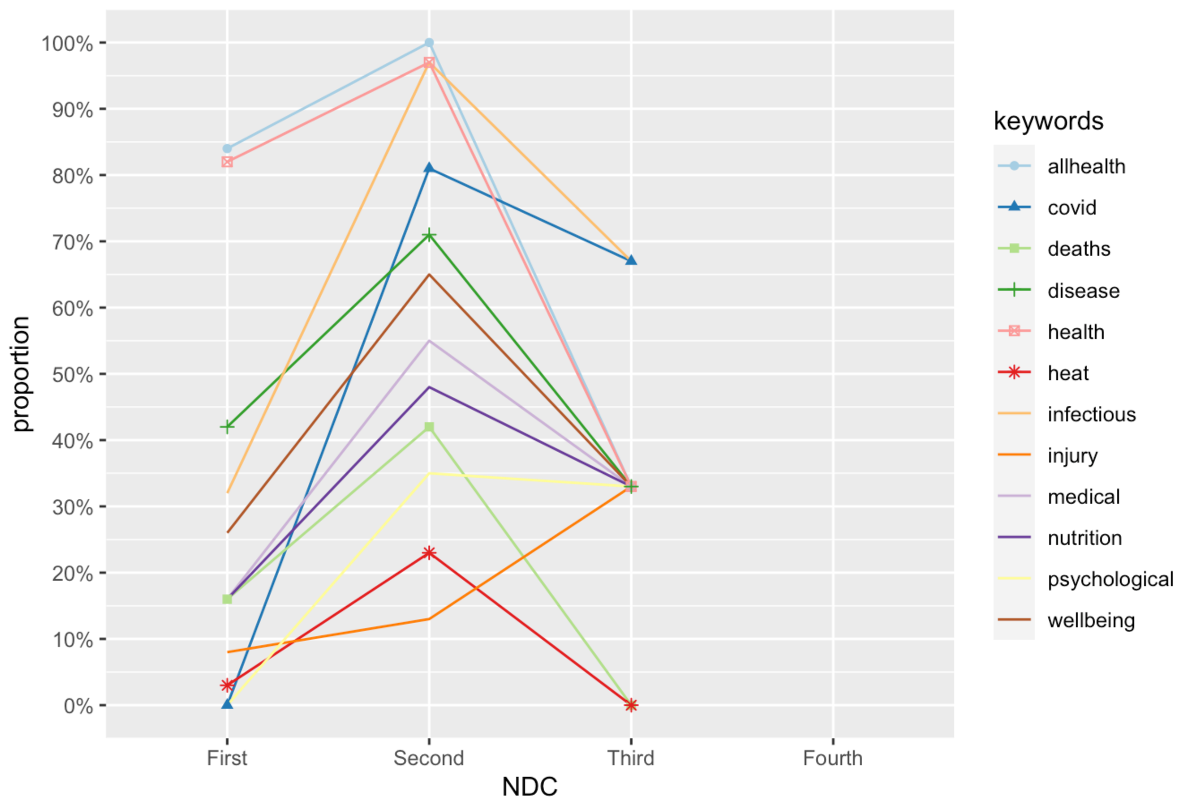

Figure 176. Proportion of NDCs in Small Island Developing States Lancet Countdown grouping using different health terms, across iterations (first NDC n=38, second NDC n=31, third NDC n=3, fourth NDC n=0)

HDI Classification

Figure 177 demonstrates the proportion of NDCs mentioning health by HDI group. Those in the very high HDI group consistently mention health the least across rounds (with 38%, 87%, and 69% respectively). The high HDI group has the second lowest proportion of NDCs mentioning health in the first round (83%), but then 100% of NDCs mention health across the second and third round of NDCs. The Medium HDI group mentions health in 83% of its first round NDCs, 100% of its second round NDCs, and 83% of its third round NDCs. Finally, the Low HDI group starts off, in the first round of NDCs, with the highest proportion of NDCs mentioning health, but drops off in the second (92%) and third round (75%).

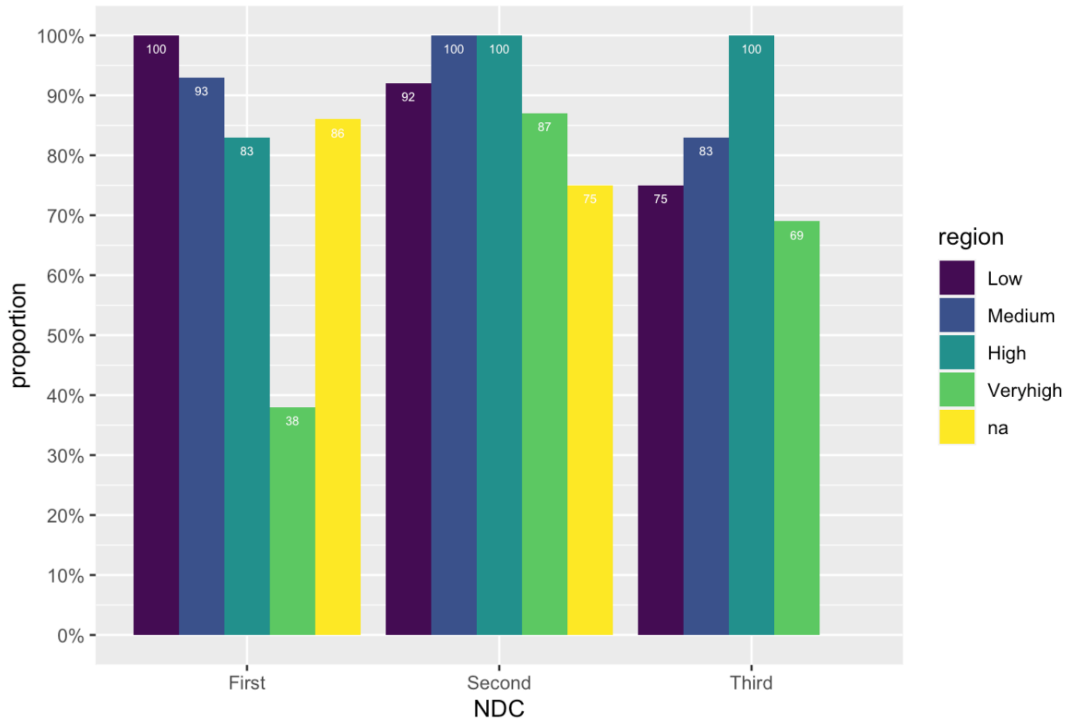

Figure 177. Proportion of NDCs per HDI classification mentioning a health keyword across iterations.

Contextual keywords

The analysis makes it possible to show the proportion of mentions that co-occur in sentences with contextual keywords, around adaptation, mitigation, co-benefits, and other areas. As is shown in Table 91 cross all three rounds of NDC, health mentions tend to co-occur with adaptation (between 26% and 28% of all mentions) and sector-based keywords (between 39% and 43%), not that these are mutually exclusive (e.g. "Adaptation is required in the healthcare and industrial sector"). Co-occurrences with mitigation keywords increase in each round, though remain between 7.8% and 11%. References to co-benefits, though low in number, are higher in the second and third round (from 1.9% to 2.6% and 2.4% of all NDCs). Keywords relating to gender appear to co-occur with health mentions far more in the second and third round of NDCs (12.5% and 9.3%, respectively, up from 6%).

NDC	adaptation	mitigation	co-benefits	trade-off	gender	sector	knowledge	rights
First	27.7%	7.8%	1.9%	0%	6%	43.4%	16.8%	2.8%
Second	25.6%	9%	2.6%	0%	12.5%	40.1%	12.5%	5.2%
Third	26.8%	11%	2.4%	0%	9.3%	38.7%	12.3%	5%

Table 91. Contextual keyword co-occurrences across all NDCs, in all iterations

5.4.2: Engagement by International Organisations

Indicator authors

Dr Olga Gasparyan, Dr Pete Lampard, Prof Cathryn Tonne

Methods

While the direct health benefits of climate change mitigation are often longer-term and diffuse, health co-benefits have been shown to provide strong, tangible local impacts, especially for developing countries, in relatively short time frames. They therefore can provide compelling arguments for increasing mitigation ambition. In many instances, the health co-benefits have been shown to outweigh the mitigation costs. Nonetheless, a gap remains between the potential and the actual role of health co-benefits in the development of national climate change mitigation policy and engagement with the concept by major international organisations. Possible explanations include the lack of political traction of health co-benefits, a dominant focus on cost minimisation (as opposed to cost-benefit analysis)[362] and barriers in research translation.[363] Natural language processing (a subfield of machine learning) is used here to track the uptake and engagement of health co-benefits in policy discourse on social media of major international organisations (IOs) involved in climate change adaptation and mitigation work.

With the dataset of IO's tweets a search through the text of each tweet was performed to identify if they discuss co-benefits. First, this included the standard list of keywords used in WG5 analysis. This produced limited results as the terms are both not specific enough to co-benefits and also do not match well to the mode of communication on social media platforms. Next, a list of keywords was developed corresponding to seven exposure pathways linking mitigation action and health (e.g. air pollution, plant-based diets)[364], and terms directly relating to the concept of health co-benefits (e.g. ancillary benefits), and specific mitigation interventions expected to have health co-benefits (e.g. transition to renewable electricity generation). This approach was selected to encompass the overall concept of health co-benefits of mitigation as well as the specific ways in which they might be achieved. A full list of search terms is provided below.

An indicator of engagement intensity was developed as a monthly proportion of tweets containing at least one term from the search term list in relation to the total number of tweets by that IO. An originally written computational algorithm was developed to identify the lists of tweets that contain each of the keywords from a given list of keywords for each international organisation in each month of the 2010–2022 period. All lists were combined and a list of unique tweets was identified by excluding any duplicate tweets that were the results of several keywords being mentioned in the same tweet. The number of tweets in these unique tweets list was the total number of keyword-mentioned tweets, used as the numerator. The total number of tweets written by each IO in a given month was calculated and the total sum as a denominator. The resulting indicator is simply a proportion calculated by dividing keywords mentions by the total sum.

To identify the intensity for individual pathways the exact same calculations were performed but only using the keywords form a given pathway. Due to the presence of multiple keywords in the same tweet the sum of the number of tweets with each of the pathway keywords is not equal to the total number of tweets with all the keywords.

Keywords:

> **Direct co-benefits terms**: health benefit, win-win, double dividend, benefit, cobenefit, co-benefit, secondary benefit, ancillary benefit, side benefit, collateral benefit, associated benefit, ancillary effect, knock on effect, ancillary impact, side effect, co-control, carbon benefit, reduction benefit, synergy, side effect, spillover, trade-off, distributional aspect, distributional effect, mortality impact.

Policy related terms: Paris Agreement, 2 degrees, 2C, 2°C, 1.5°C, 1.5C, Climate pledge, Climate goals, Energy pledge, Net-zero, NetZero, Zero emission, Decarbonisation, Decarbonization, Mitigate, Mitigation, Carbon neutral, Carbon neutrality, Low carbon.

Intervention, Energy: Renewables, Solar, Photovoltaics, PV, Batteries, Wind, Coal, Clean energy, Energy demand, Energy use, Energy efficiency, Heat pumps, Building retrofit, Smart thermostat, Insulation, Net-zero buildings, Green roof, Cool roof, Electric vehicles, Clean cooking, LED lighting, Geothermal power, Fuel poverty, Energy poverty, Nuclear, Electricity, Hydrogen, Fossil fuel, Energy crisis, Energy investment, Affordable energy, Natural gas

Intervention, Land use: Forest restoration, Tree plantation

Pathway, Air Pollution: Air quality, Air pollutants, Air toxin, PM2.5 , PM25, particles, particulates, ozone, Smog, Soot, black carbon , short-lived climate pollutants, SLCP , SLCPs

Pathway, Road transport noise: Traffic noise, Aircraft noise

Pathway, Temperature: Urban heat island, Heat, Overheat, Carbon sequestration, Cooling, Humidity, Mold

Pathway: Diet: Dietary, Nutrition, Meat, Dairy, Vegetarian, Vegan , Plant-based, Plant-rich,

Pathway, Physical activity: Exercise, Active travel, Walking, Walkable, Cycling , Bicycle,

Pathway, Sustainable mobility: Public transport, Rail, Trains

Pathway, Nature exposure: Green, greenspace, green space, Cooling, Trees, Forest , Nature based solution, Nature

Data

41 international organisations were selected based on IOs representing various sectors: economic, financial, environment, regional development, etc. The sample of IOs is based on a recent study on policy discourse in climate change adaptation IOs [365] and extended to cover mitigation IOs. Twitter is used as the social media platform to collect official communication by IOs. All the tweets written by official Twitter accounts of the selected IOs were extracted for the period of 2010-2022. Since social media platforms have become one of the most common sources of information for journalists and the general public, policy actors utilise the platforms as the key mode of media and public engagement. Additionally, using the same platform and data structure allows a comparison of engagement across international organisations controlling for institutional variability in formats and modes.

The final dataset contains 1,392,892 tweets written by 41 international organizations through the period of 2010-2022, from which 1,354,924 are English language tweets. These are all the tweets published by official twitter accounts of these organizations, including retweets and quotations. Since the majority of tweets are written in English and all keywords are in English, the further analysis below only works with the English language tweets and excludes all the tweets that do not have “lang=en” in the tweets language meta-data identifier.

Organization	Acronym	Twitter handle	Field	Sector

				**Classification**
African Union	AU	_AfricanUnion	Regional Cooperation	adaptation
Asian Development Bank	ADB	ADB_HQ	Global Development Banking	both
African Development Bank	AFDB	AfDB_Group	Global Development Banking	adaptation
The Africa Rice Center, formerly known as the West Africa Rice Development Association	WARDA	AfricaRice	Food and Agriculture	adaptation
Asia-Pacific Economic Cooperation	APEC	APEC	Regional Cooperation	both
Association of Southeast Asian Nations	ASEAN	ASEAN	Regional Cooperation	both
European Bank for Reconstruction and Development	EBRD	EBRD	Global Development Banking	adaptation
Economic Community of West African States	ECOWAS	ecowas_cedeao	Trade and Economy	adaptation
European Investment Bank	EIB	EIB	Global Development Banking	both
European Union	EU	EU_Commission	Regional Cooperation	both
UN Food and Agriculture Organisation	FAO	FAO	Food and Agriculture	adaptation
Pacific Islands Forum	PIF	ForumSEC	Regional Cooperation	adaptation
International Energy Agency	IEA	IEA	Energy Policy	mitigation
International Fund for Agricultural Development	IFAD	IFAD	Food and Agriculture	adaptation
International Finance Corporation	IFC	IFC_org	Global Development Banking	both

International Monetary Fund	IMF	IMFNews	Global Development Banking	both
International Renewable Energy Agency	IRENA	IRENA	Energy Policy	mitigation
North Atlantic Treaty Organization	NATO	NATO	Peace and Security	adaptation
Organization of American States	OAS	OAS_official	Regional Cooperation	both
Organization for Economic Co-operation and Development	OECD	OECD	Development	both
Organization for Security and Co-operation in Europe	OSCE	OSCE	Peace and Security	adaptation
United Nations High Commissioner for Refugees	UNHCR	Refugees	Migration	adaptation
South Asian Association for Regional Cooperation	SAARC	SaarcSec	Regional Cooperation	adaptation
Southern African Development Community	SADC	SADC_News	Development	adaptation
Inter-American Development Bank	IADB	the_IDB	Global Development Banking	adaptation
UN Security Council	UNSC	UN	Peace and Security	adaptation
UN Development Programme	UNDP	UNDP	Development	adaptation
United Nations Office for Disaster Risk Reduction	UNDRR	UNDRR	Disaster Risk Management	adaptation
United Nations Economic Commission for Europe	UNECE	UNECE	Development	both
United Nations Environment	UNEP	UNEP	Environment Policy	mitigation

Programme				
United Nations Framework Convention on Climate Change	UNFCC	UNFCCC	Environment Policy	mitigation
United Nations Population Fund	UNFPA	UNFPA	Health	adaptation
United Nations Children's Fund	UNICEF	UNICEF	Development	adaptation
International Organisation for Migration	IOM	UNmigration	Migration	adaptation
United Nations Office for the Coordination of Humanitarian Affairs	OCHA	UNOCHA	Disaster Risk Management	adaptation
World Economic Forum	WEF	wef	Trade and Economy	both
World Food Programme	WFP	WFP	Food and Agriculture	adaptation
World Health Organization	WHO	WHO	Health	both
World Bank	WB	WorldBank	Global Development Banking	both
World Trade Organisation	WTO	wto	Trade and Economy	both
East African Community	EAC	jumuiya	Regional Cooperation	adaptation

List of International Organizations with Sectors and Field Classification

Caveats

There are several limitations of the current analysis to be improved in the next iterations of this study. First, working with a limited predefined set of international organisations: the plan is to expand the set for the near universal set of international organisations with the active Twitter accounts. Second, the set of search terms will be further fine-tuned to provide a richer picture of co-benefits discussions.

Additional Analysis

IOs intensify their engagement with health co-benefits in their public discourse over time. **Figure 178** shows the dynamic of the intensity of engagement, which is the proportion of an IO’s Twitter posts mentioning co-benefits with respect to the total number of tweets by that IO. The structure of the measure suggests that the observed trend is not simply reflecting IOs’ increasing use of this specific social media platform, but rather a genuine increase in co-benefit topic engagement. The results show that by November 2022 22% (1896 tweets with key words /8625 total number of tweets) of all tweets written by the analysed IOs mention co-benefits.

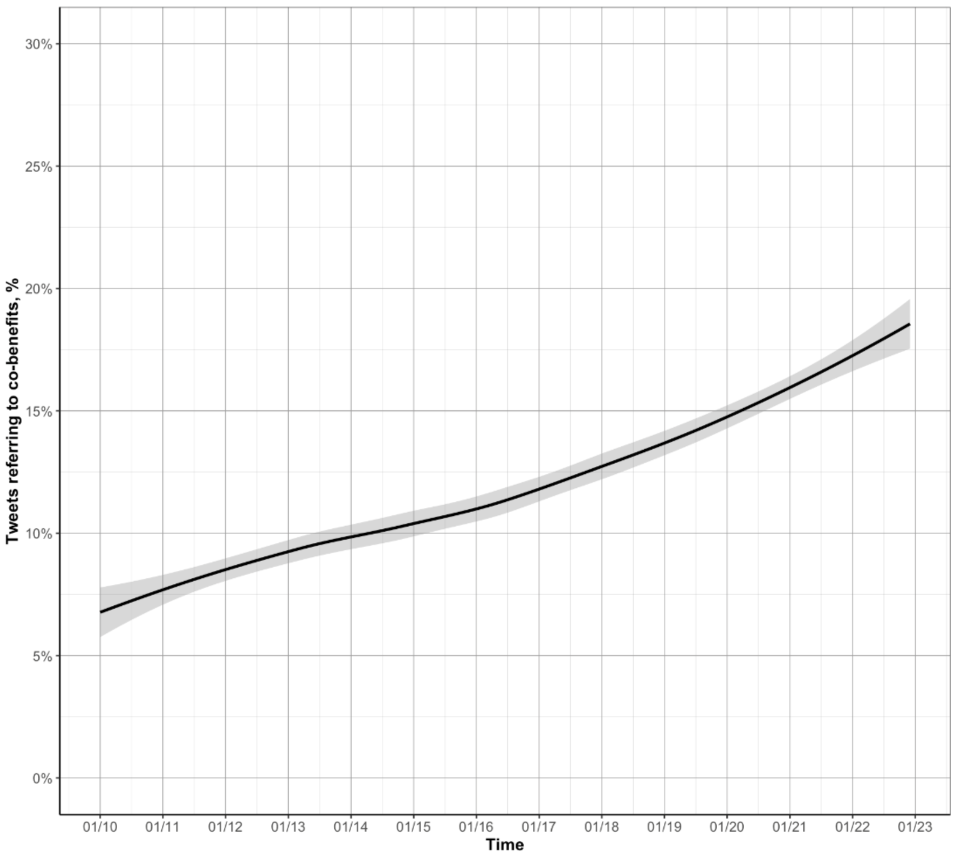

Figure 178. Engagement intensity of IOs with co-benefits on Twitter (Note: Trend line is a fitted loess line with the shaded area representing a 95% confidence interval.)

IOs in the sample are diverse both in terms of their sectoral focus and in terms of key operational focus on mitigation, adaptation or a mix of adaptation and mitigation work. **Figure 179** shows the results of the analysis across the mitigation/adaptation/both categorisations. Mitigation IOs have a higher co-benefits engagement intensity compared to adaptation IOs (or IOs working both on mitigation and adaptation). Increasing time trends for all three groups of IOs can be observed, with the most evident growth after early 2016 likely linked to the Paris Agreement.

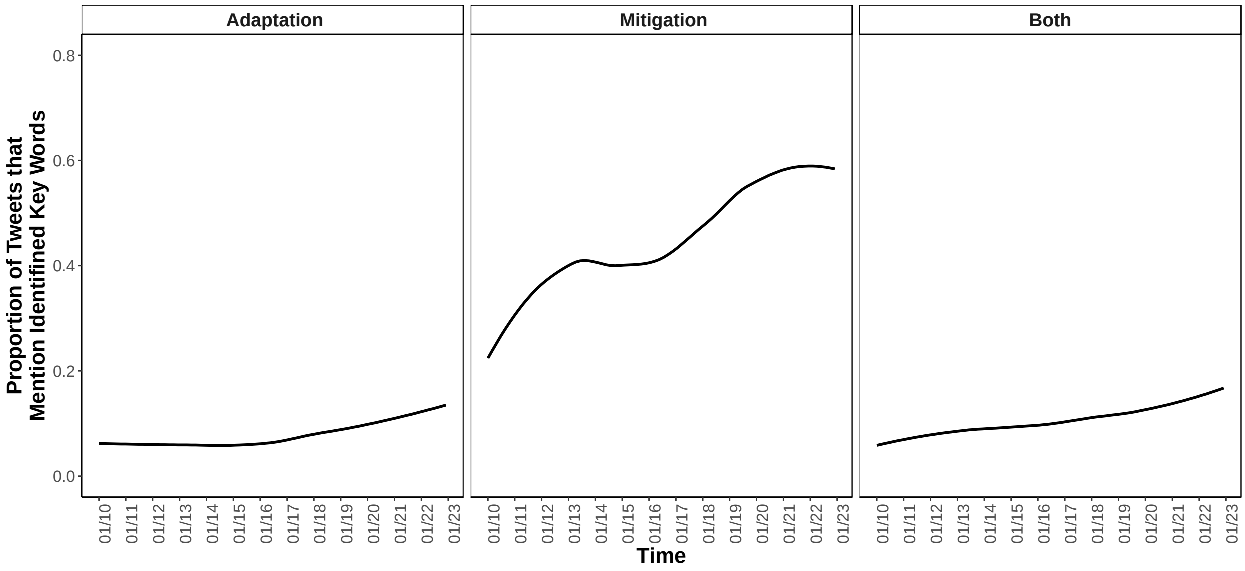

Figure 179. Engagement intensity of IOs with co-benefits on Twitter across adaptation, mitigation or both categorisation

Sectoral analysis results are provided in **Figure 180**, showing clear sectoral differences in engagement intensity with co-benefits. The sectoral classification is adopted from Kural et al [365]. The most intensive co-benefits engagement is from IOs in the Energy, Environment, Food and Agriculture, and Global Development Banking sectors.

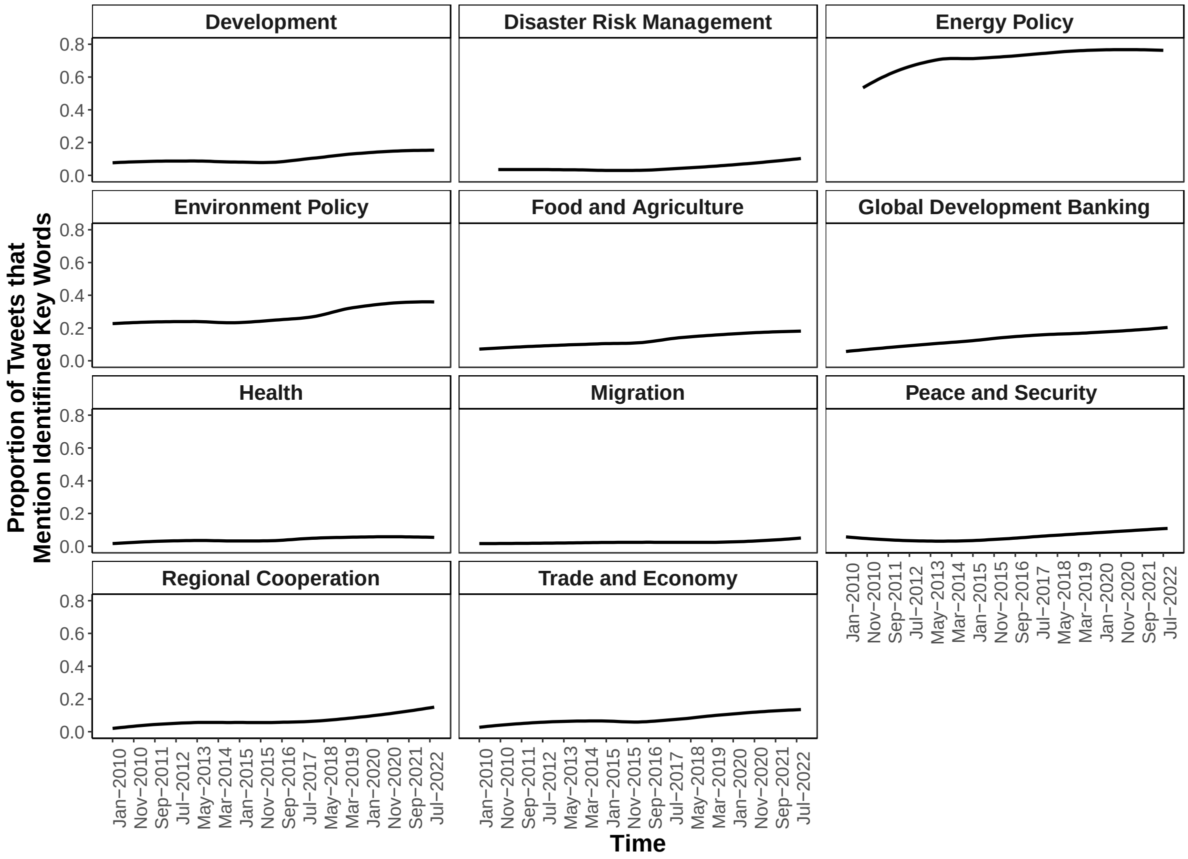

Figure 180. Sectoral analysis of engagement intensity of IOs

The results for individual IOs are presented in **Figure 181**. IRENA and IEA (both from the energy sector) are the most prominent in their engagement.

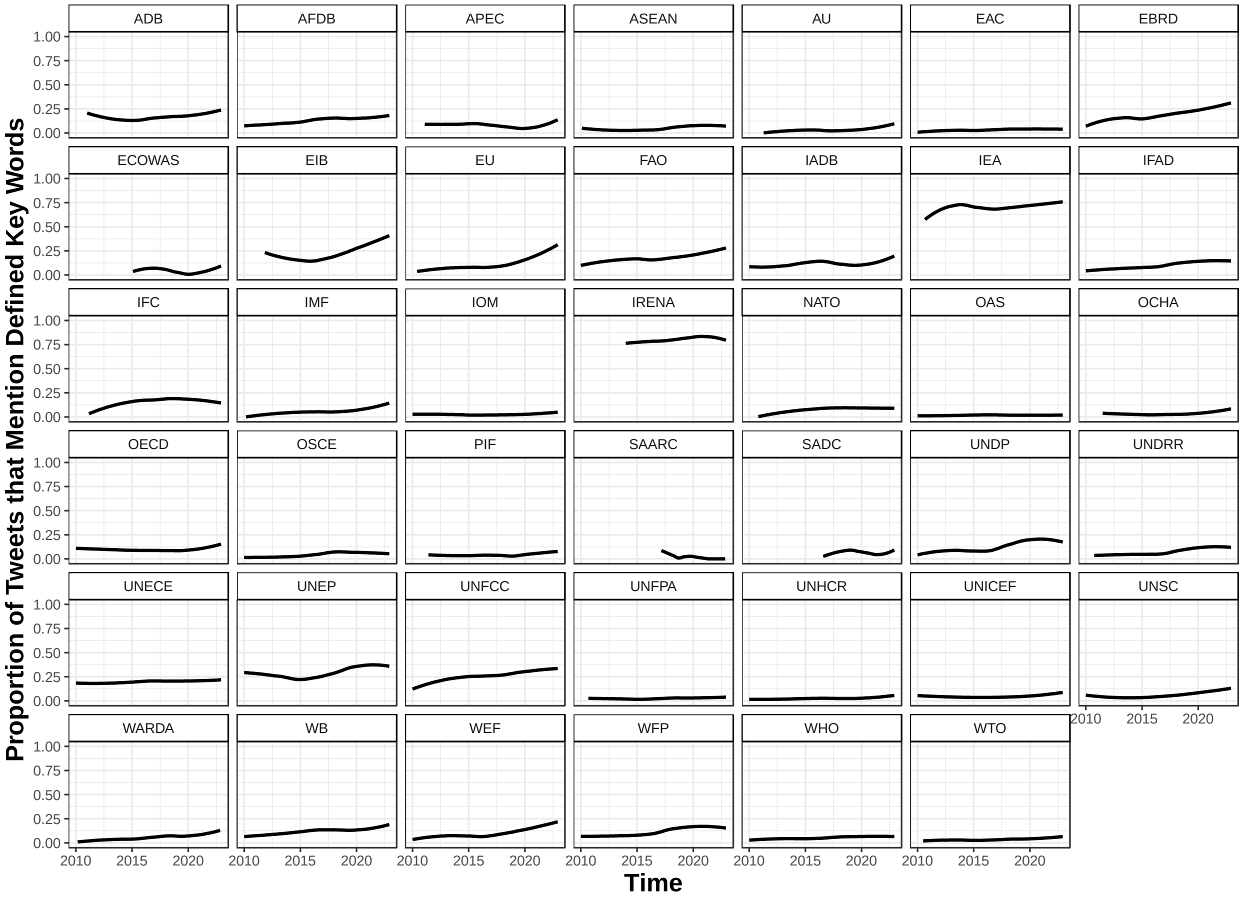

Figure 181. Engagement intensity for individual IOs (Note: Names of the IOs listed according to the official acronyms, which are listed in Table A.1.)

Topic modelling

Figure 182 shows that the highest engagement intensity is around energy, nature exposure, temperature, policy specific, and co-benefits specific terms. As a validation exercise, the indicator also looked at the content of the tweets that contain the search terms. To do this at scale, probabilistic topic models are used [366] - a group of unsupervised machine learning techniques applied to the collected text corpus (tweets containing the keywords). However, for validation, in addition to identifying topic clusters in tweets, the indicator looked to understand if the topics identified are related to a set of covariates that capture specific characteristics of IOs and time-series dynamics. Structural topic models (STM) [367,368] are utilised for this task. Overall, there is evidence that, indeed, the content of the tweets picked up through the search terms is related structural IO and pathway characteristics.

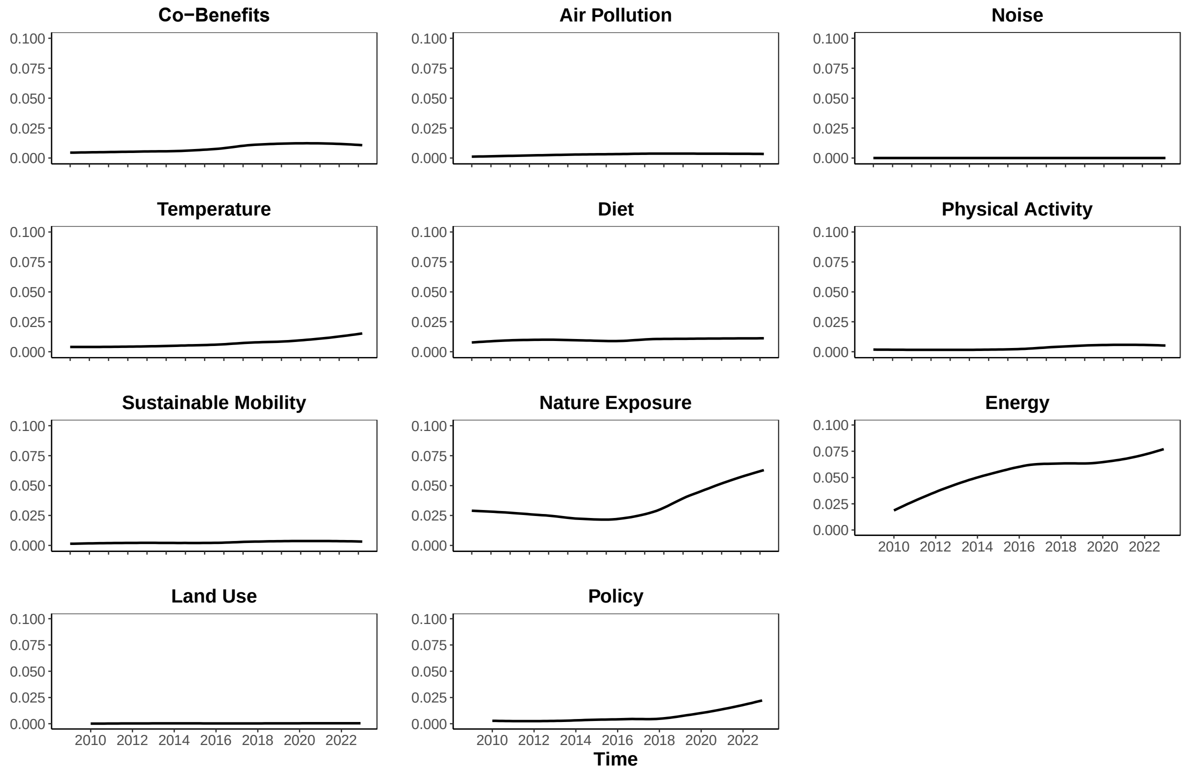

Figure 182. Exposure intensity by search term category

Selecting the number of topics for analysis

To identify the appropriate number of topics for the structural topic models, two main diagnostics measures are presented: semantic coherence (which is generally decreasing with the increase in the number of topics) and exclusivity (which is increasing with the increase in the number of topics). The indicator operates based on some known heuristics about the optimal balance between semantic coherence and exclusivity. Diagnostics for two main models of interest were performed:

(1). Topic prevalence = beta0+beta1*IO_sector+beta2*Y+error

(2). Topic prevalence = beta0+beta2*IO_field+beta2*Y+error

where Y is a year, sector is a three-point factor variable (adaptation, mitigation, both), and field is an 11-point factor variable, representing IOs field of operation (Development, Disaster Risk Management, Energy Policy, Environment Policy, Food and Agriculture Global Development Banking, Health, Migration, Peace and Security, Regional Cooperation, Trade and Economy). **Figure 183** presents diagnostics results for Model 1, and **Figure 184** presents diagnostics results for Model 2. As a result of these diagnostics, K=8 was chosen as the number of topics for Model 1, and K=6 as the number of topics for the Model 2.

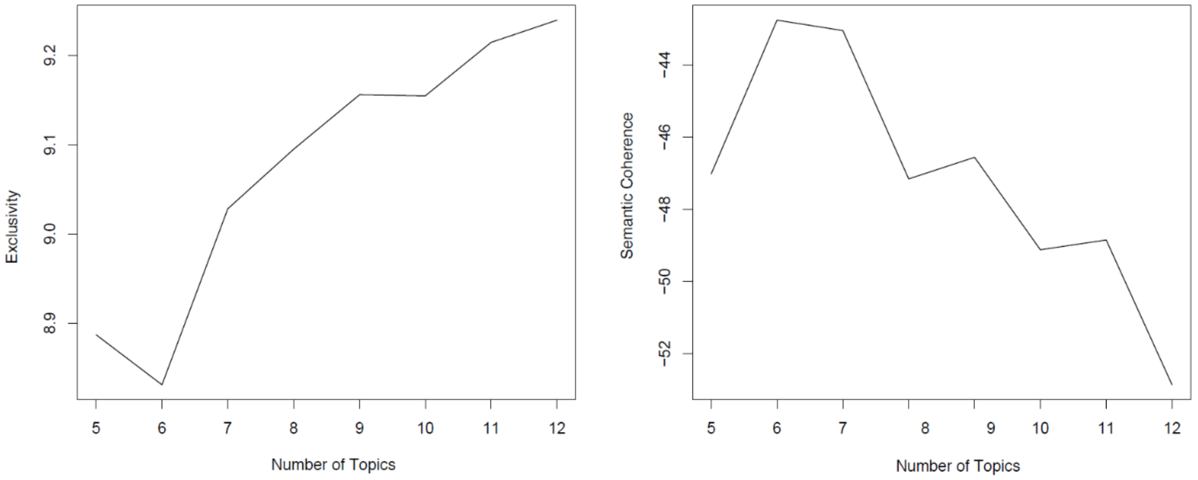

Figure 183. Diagnostics for the Number of Topics Choice (for Sector Models)

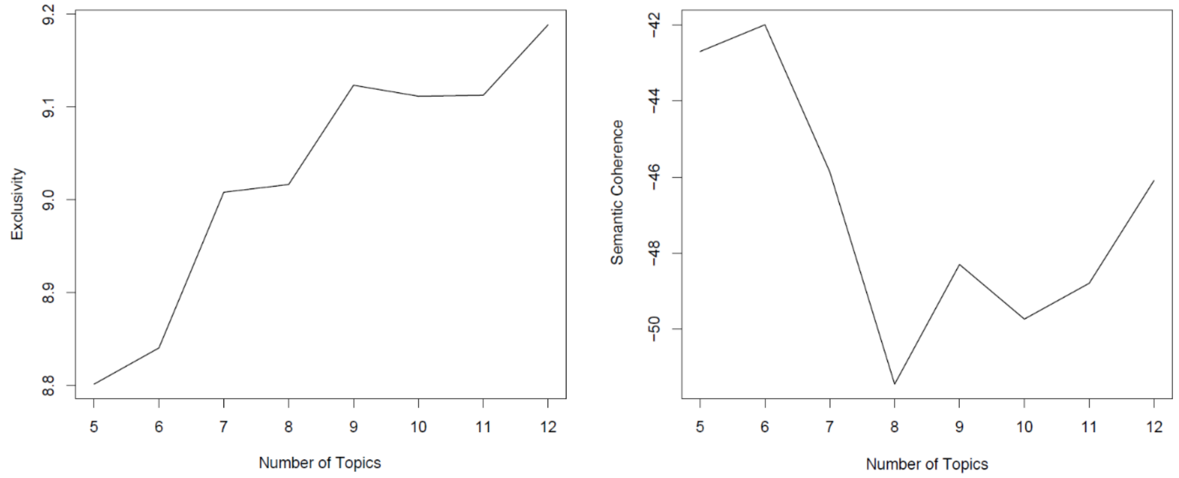

Figure 184. Diagnostics for the Number of Topics Choice (for Field Models)

Sector-based STM

The results below follow the first topic model, evaluating the effect of topic prevalence controlling for IO sectors: adaptation, mitigation, or both and the dynamic year effect. **Figure 185** indicates the distribution of topics and **Figure 186** presents the examples of the words for each of the topics.

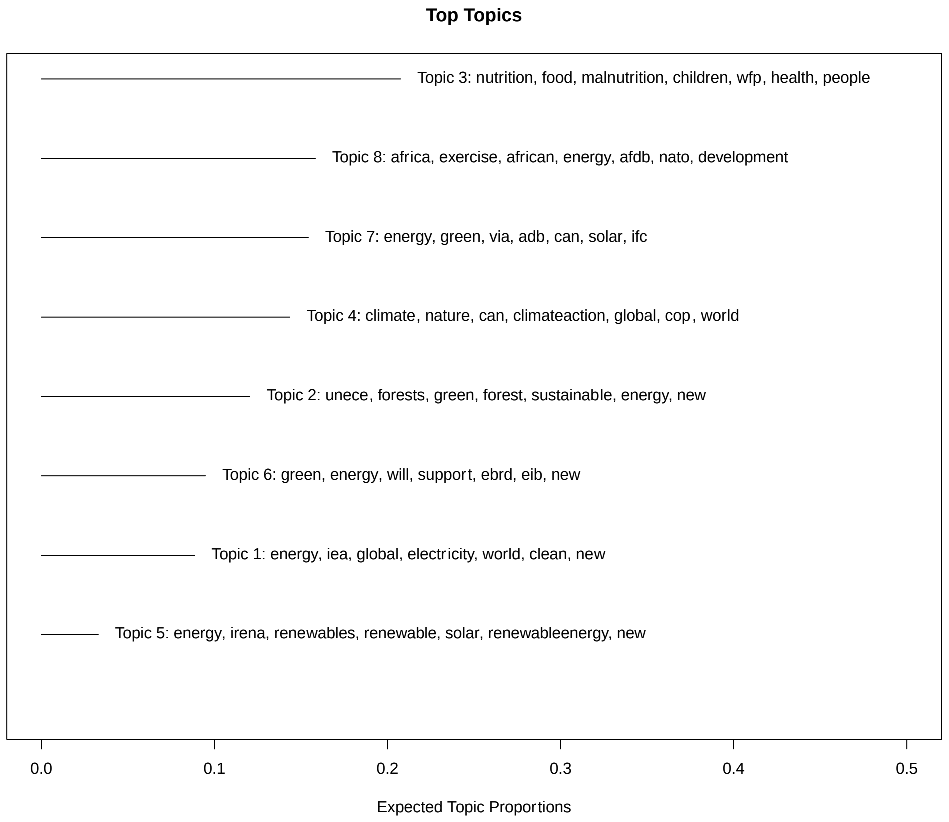

Figure 185. Topic Distribution

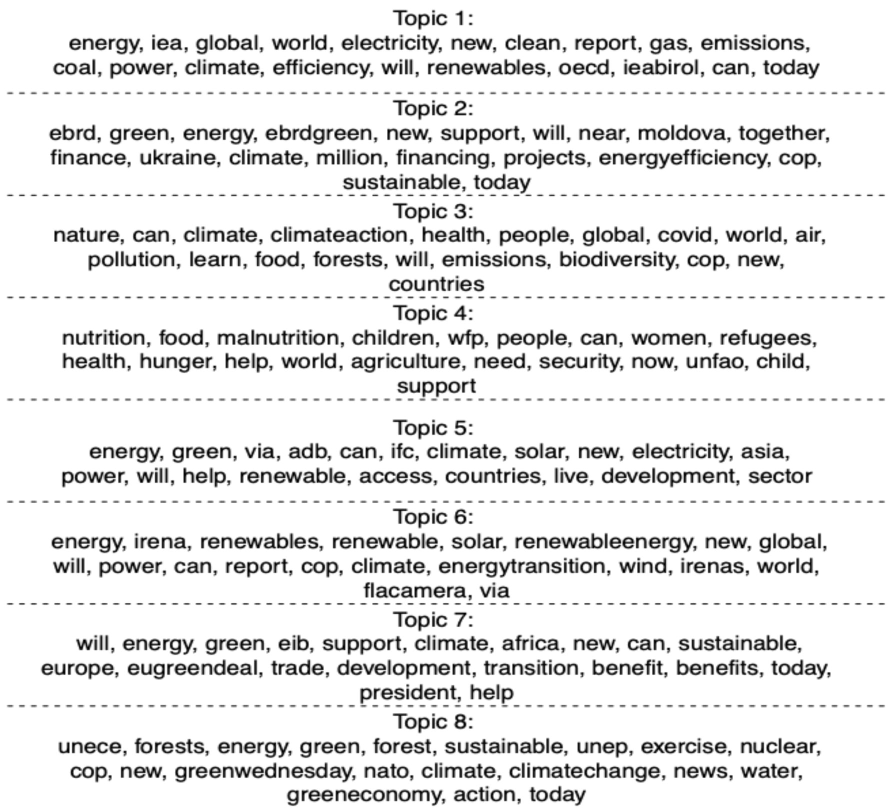

Figure 186. The most indicative words for K=8 Topics

Four topics were chosen with the largest prevalence across the documents: topics 4, 7, 3, and 8. By looking at the most indicative words in these topics these were labelled by hand as Topic 3: Food and Nutrition; Topic 8: Development and Economy; Topic 7: Green and Energy; Topic 4: Climate Action and Nature. The effect of covariates of interest for each of these four topics was estimated. Figure 187 shows coefficient plots for the coefficients of interests for IO sectors, where all the models were evaluated controlling for the yearly dynamic trends. Here it can be seen that adaptation IOs mostly talk about food and nutrition topics, and development. Mitigation-focused IOs are mostly active in climate change and support as well as energy and green advocacy rhetoric.

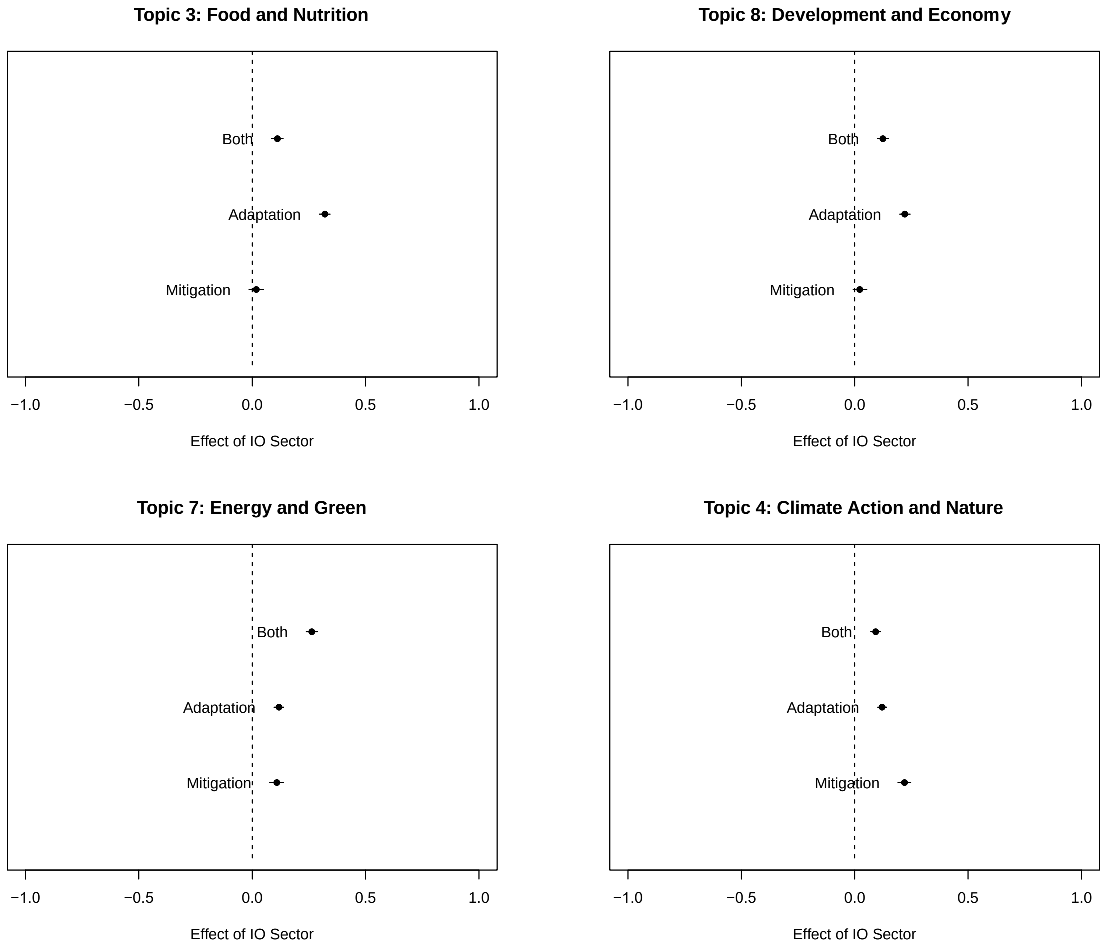

Figure 187. Coefficients plots of the IO sectors for Selected Topics

Another topic model evaluated was with respect to the sectors of the IOs, classifying selected international organisations over 11 sectors. 6 topics were derived from this, and Figure 188 presents topic prevalence with the seven highest probability words for each topic. Top words per topic are presented in Figure 189.

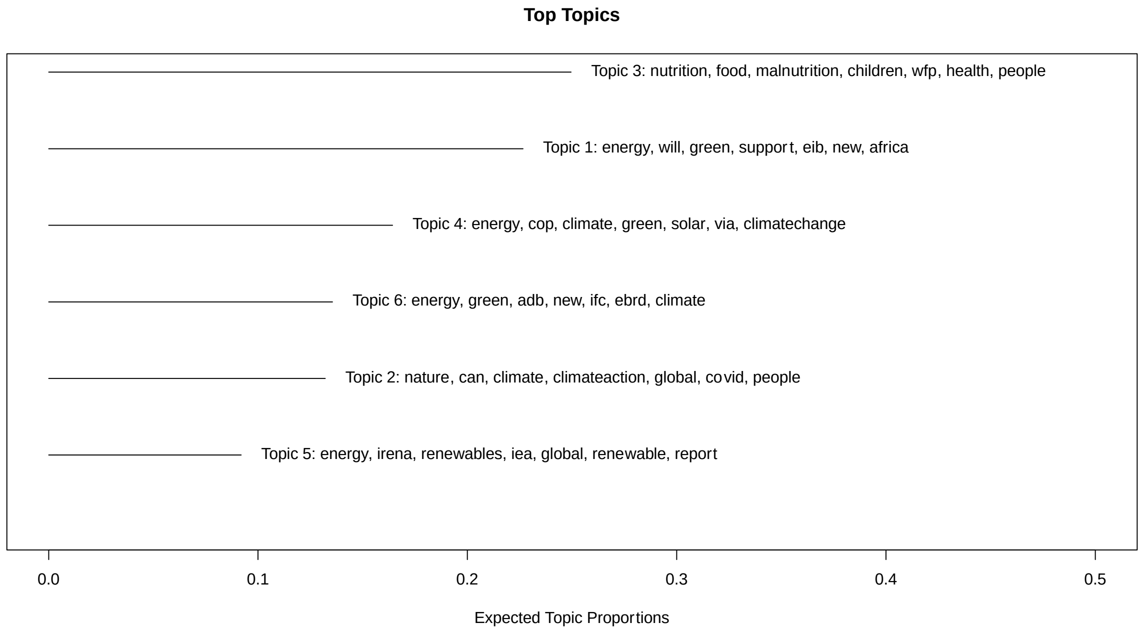

Figure 188. Topic Distribution

Topic 1:
energy, will, green, support, eib, new, africa, today, climate, europe, sustainable, transition, trade, development, european, projects, exercise, eugreendeal, flacamera, african

---

Topic 2:
nature, can, climate, climateaction, global, covid, people, world, forests, unece, sustainable, biodiversity, emissions, countries, air, learn, food, will, action, help

---

Topic 3:
nutrition, food, malnutrition, children, wfp, health, people, can, women, healthy, help, refugees, hunger, world, benefits, need, agriculture, now, security, unfao

---

Topic 4:
energy, cop, climate, green, solar, via, climatechange, new, can, forests, world, global, environment, power, renewable, read, unep, carbon, news, says

---

Topic 5:
energy, irena, renewables, iea, global, renewable, report, new, electricity, world, power, solar, can, will, clean, climate, today, renewableenergy, sector, emissions

---

Topic 6:
energy, green, adb, new, ifc, ebrd, climate, will, can, via, solar, help, asia, power, electricity, sector, ebrdgreen, million, support, finance

Figure 189. The most indicative words for K=6 Topics

The three most prevalent topics for further analysis were selected: Topic 2: Food and Nutrition; Topic 4: Energy, Green, and Sustainability; Topic 6: Energy, Green, Development; Topic 1: Climate, Nature, Climate Action. Figure 190 shows coefficient plots for these four topics for each of the IO fields, estimated on the model with additional controls on the yearly time trends. There is some variation in the topics and pathways that different IO fields speak about with topics of food, nutrition, health, and children is mostly covered by IOs specializing in health, food and agriculture, and migration. Energy and sustainability topics are mostly covered by regional, development, peace and security, and trade and economy IOs. Whereas climate change actions topics are expressed by environment policy oriented IOs.

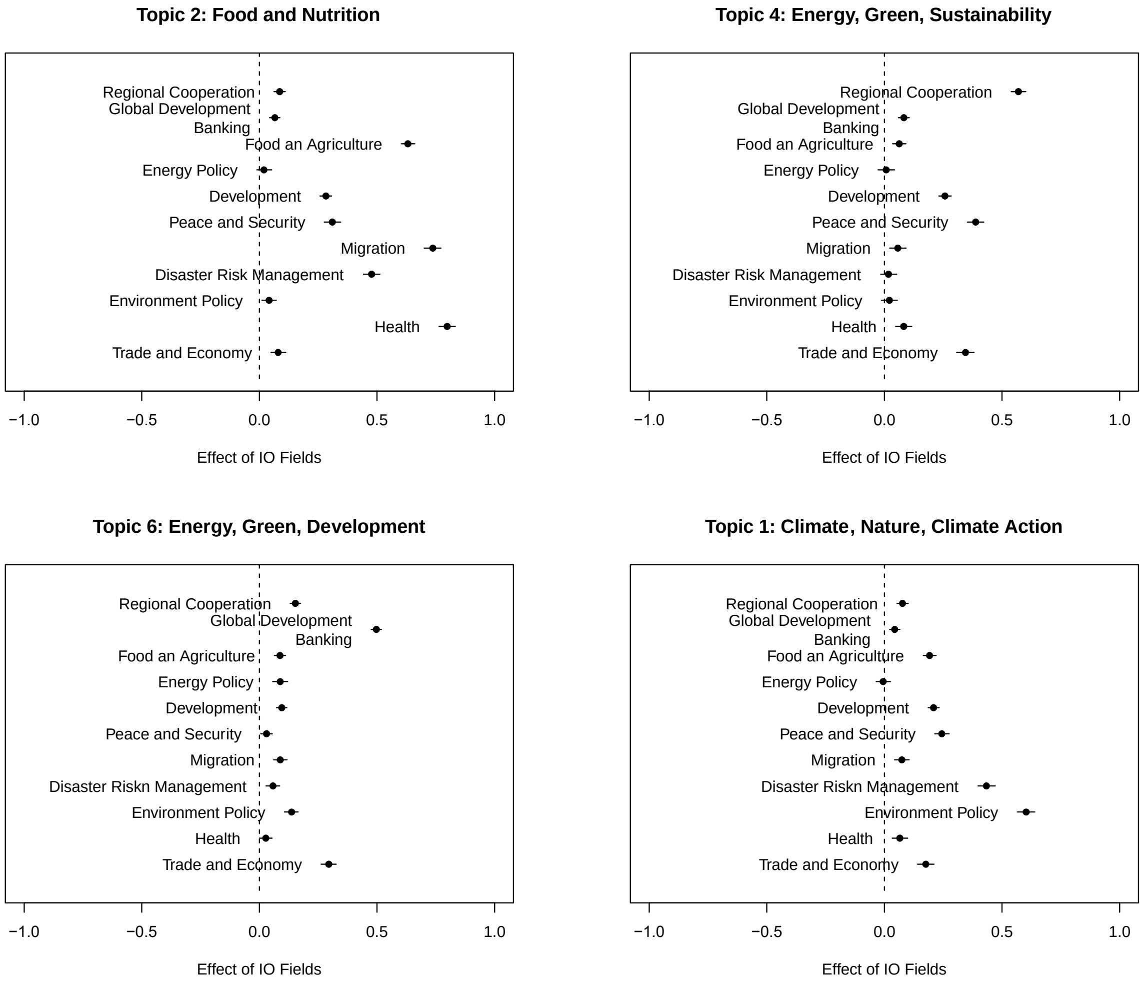

Figure 190. Coefficients plots of the IO fields for Selected Topics

Indicator 5.5: Corporate Sector Engagement in Health and Climate Change

Indicator authors

Dr Niheer Dasandi, Prof Slava Jankin

Methods

To produce the measure of engagement with climate change and health in companies' UN Global Compact Communication of Progress (GCCOP) reports, the publicly available GCCOP reports were used. This approach to using the GCCOP reports to produce the indicators is based on identifying references to key search terms linked to (a) health, and (b) climate change:

> **Health:** malaria, diarrhoea, infection, disease, diseases, sars, measles, pneumonia, epidemic, epidemics, pandemic, pandemics, epidemiology, healthcare, health, mortality, morbidity, nutrition, illness, illnesses, ncd, ncds, air pollution, nutrition, malnutrition, malnourishment, mental disorder, mental disorders, stunting.
>
> **Climate change:** climate change, changing climate, climate emergency, climate action, climate crisis, climate decay, global warming , green house, temperature, extreme weather, global environmental change, climate variability, greenhouse, greenhouse-gas, low carbon, ghge, ghges, renewable energy, carbon emission, carbon emissions, carbon dioxide, carbon-dioxide , co2 emission, co2 emissions, climate pollutant, climate pollutants, decarbonization, decarbonisation, carbon neutral, carbon-neutral, carbon neutrality, climate neutrality, net-zero, net zero.

These key terms have been updated from previous years to reflect the changing terminology used to discuss climate change. In order to produce an indicator of engagement with the intersection of climate change and health, the analysis focused on whether any of the climate change related terms appeared immediately before or after any public health terms in the COP reports. This was based on a search of the 25 words before and after a reference to a public health related term.

Data

To produce this indicator, the publicly available UN Global Compact COP reports are used. A total of 39,159 reports were downloaded from COP. The reports are available for companies based in 129 countries. GCCOP reports are submitted in 30 different languages. While in past years, the focus was only on the reports available in English; this year reports from all languages are included. In total, reports were submitted in 30 languages.

Sector	intersection	climate	health
Aerospace & Defense	396	3489	7779
Alternative Energy	588	7326	7980

Automobiles & Parts	792	12788	20106
Banks	1014	19053	25927
Basic Resources	0	5	17
Beverages	771	9036	19889
Chemicals	2454	17544	43072
Construction & Materials	1796	23284	55997
Diversified	2621	28677	59166
Electricity	1418	24525	35642
Electronic & Electrical Equ...	755	10854	21283
Equity Investment Instruments	175	1605	2798
Financial Services	2159	35162	42392
Fixed Line Telecommunications	354	4215	10212
Food & Drug Retailers	280	3106	8184
Food Producers	2572	17733	71076
Forestry & Paper	440	7832	9942
Gas, Water & Multiutilities	833	13487	21458
General Industrials	2441	29539	62901
General Retailers	790	13664	27864
Health Care	0	7	50
Health Care Equipment & Ser...	1174	4865	47869
Household Goods & Home Cons...	455	8434	15071
Industrial Engineering	817	9288	19209
Industrial Goods & Services	2	50	675
Industrial Metals & Mining	1051	12438	29758
Industrial Transportation	926	11603	21891
Leisure Goods	97	2366	4001
Life Insurance	749	4122	14059
Media	237	4822	10931
Mining	596	6057	16037
Mobile Telecommunications	539	7410	13738
Nonequity Investment Instru...	26	275	557
Nonlife Insurance	429	4035	11201
Not Applicable	201	3345	16174
Oil & Gas Producers	2126	25404	42037
Oil Equipment, Services & D...	332	3782	8306
Other	0	26	146
Personal Goods	556	6677	15847
Pharmaceuticals & Biotechno...	2253	9054	73826
Real Estate Investment & Se...	807	11032	18265
Real Estate Investment Trusts	405	3105	3769
Retail	3	49	415

Software & Computer Services	983	13004	25394
Support Services	2083	27420	67211
Technology	0	8	51
Technology Hardware & Equip...	824	13657	23083
Tobacco	2	48	137
Travel & Leisure	660	10171	18993

Table 93These reports were translated into English using the open-source pretrained neural machine translation model Opus-MT[v] under the Huggingface[7] pipeline to implement the translation task. A number of the files were corrupt or could not be converted into plain text format for analysis. The distribution of available GCCOP reports over time is presented in Table 92:

Year	Number of reports	Year	Number of reports	Year	Number of reports
2011	2057	2015	3472	2019	4060
2012	3015	2016	3573	2020	3554
2013	3231	2017	3724	2021	5737
2014	3186	2018	3751	2022	6089

Table 92. GCCOP reports by year.

There are only single GCCOP report submissions before 2011, thus analysis is limited to the sample of GCCOP reports to the period 2011-2022. These were translated, pre-processed and prepared for the application of natural language processing by converting the reports to plain text format; removing punctuation and numbers; removing stopwords; regularising (lowercasing); and stemming. All pre-processing and analysis was carried out in R using the "quanteda" package [360].

In total, the GCCOP reports were submitted in 41 different languages. The majority were submitted in English with 24,360 GCCOP reports (54%) submitted in English.

Caveat

This analysis here is based on a narrow range of search terms, which excludes reference to many of indirect links between climate change and health. Reports may also discuss indirect connections, such as the effect of climate change on agriculture, however, these are not included here. Therefore, the results present a somewhat conservative estimate of high corporate engagement with the intersection of climate change and health. Future work in this area will consider engagement with these indirect links, as well as providing additional forms of analysis.

Future Form of Indicator

In the future, the indicator will look to include search terms based on indirect links between climate change and health (e.g., agriculture) to capture references to indirect links.

Additional Information

Figure 191 presents the total number of references to climate change, health, and the intersection of climate change and health across for the GCCOP reports. Despite the increase in the proportion of

[v] https://github.com/Helsinki-NLP/Opus-MT.git

companies engaging with the climate change-health linkages, the overall number of references remains fairly low and consistent, relative to the individual references to health and climate change – though, there has been an increase in references to the intersection of climate change and heath in the past two years.

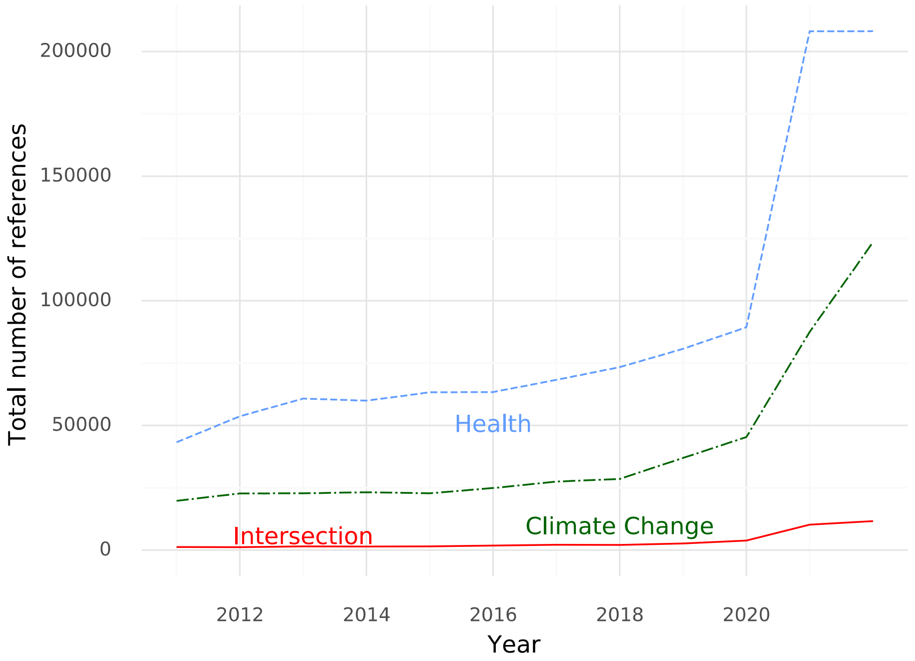

Figure 191. Total references to climate change, health, and the intersection of climate change and health, 2011-2022.

In **Figure 192**, below, the total references with the intersection of climate change and health is presented to better show any trends occurring in engagement. The figure shows that since 2018 – and particularly since 2020 – there has been a sharp rise in the number of references.

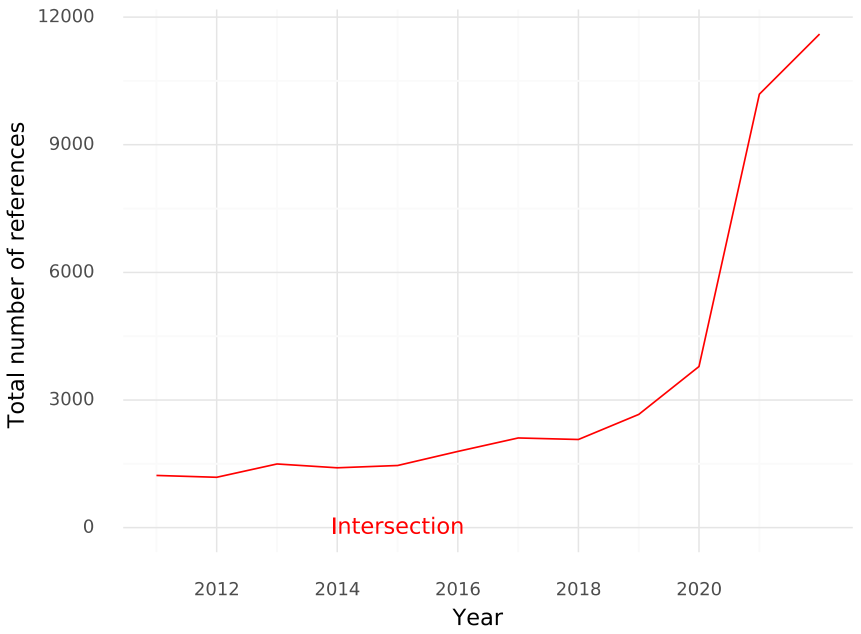

Figure 192. Total references to the intersection of climate change and health, 2011-2022.

Figure 193 shows the average number of references to climate change, health, and the intersection in GCCOP reports. The figure again demonstrates the relatively low level of engagement with the health impacts of climate change in GCCOP reports, compared to the separate references to health and climate change.

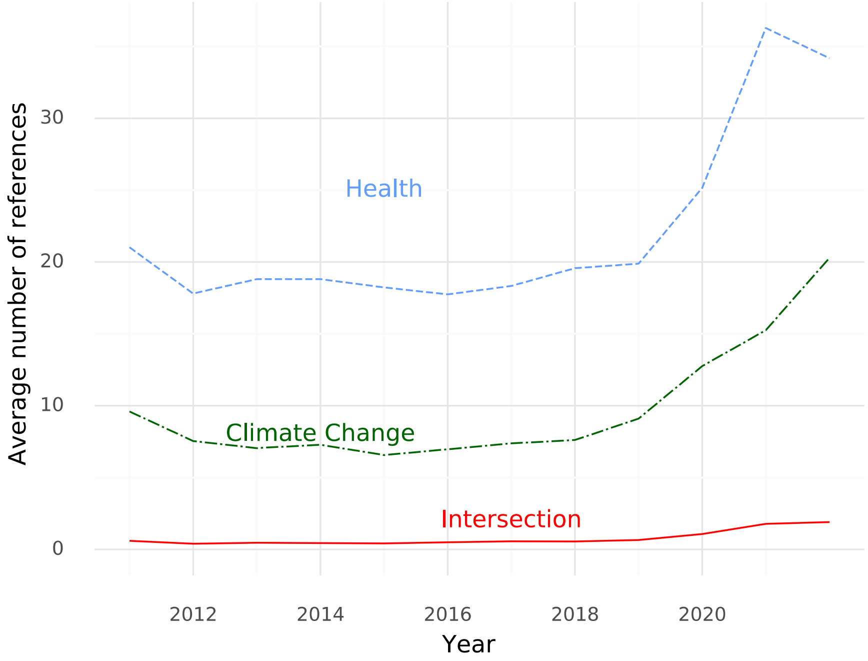

Figure 193. Average references to climate change, health, and the intersection of climate change and health in GCCOP reports, 2011-2022.

There is growing awareness of the gendered impacts of climate change and health. Considered here is the extent to which references to the health dimensions of climate change in companies' GCCOP reports engage with gender issues, by further examining the references to the intersection of climate change and health. Once all references to this intersection in GCCOP reports for 2011-2022 were identified, additional search terms related to gender to identify which of the intersection references also engaged with gender issues. The gender-related search terms used were as follows: women, women's, maternal, inequality, inequalities, gender, empowerment, sex, sexual, violence, violent, girls, reproduction, reproductive. Hence, the analysis considers whether the 25 words of text identified in the primary search (for climate change and health terms) includes a reference to at least one of these gender-related keywords.

Based on the additional search of the references to the climate change-health intersection using these gender-related keywords, references to the health dimensions of climate change with a gender focus were identified in companies' annual GCCOP reports. **Figure 194** presents annual references to the gender dimensions of climate change and health in UN Global Compact COP reports between 2011 and 2022. The figure shows a steady increase in engagement between 2015 and 2018. In 2019, there was a sharp rise, with 11% of all references to the intersection of climate change and health including a mention of one of the gender keywords, followed by a fall in 2020. Engagement with gender increased in 2022 to 10%.

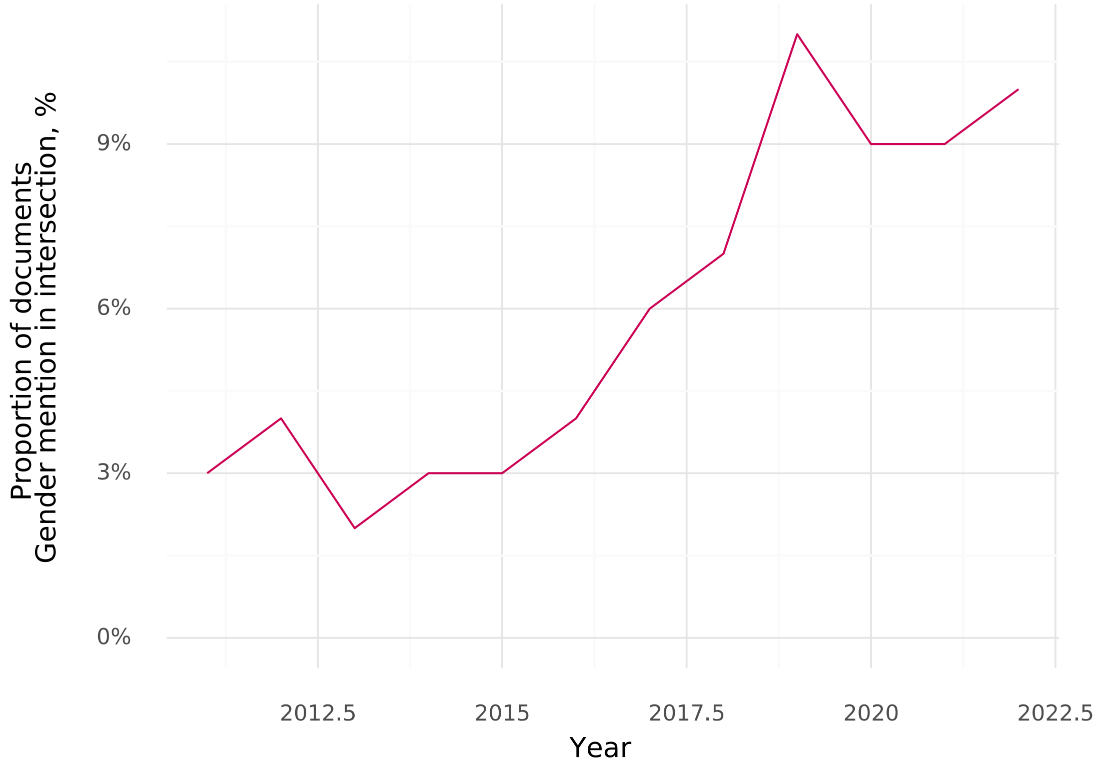

Figure 194. Proportion of references to intersection of health and climate change in GCCOP reports that include a reference to gender, 2011-2022.

Also considered here is engagement with climate change and health in the UN Global Compact COP reports by WHO region. **Figure 195** shows the total number of references to the climate change-health intersection based on which of the WHO regions a company is based on, and **Figure 196** shows the proportion of companies based in the different WHO regions that refer to the health impacts of climate change in their annual GCCOP report.

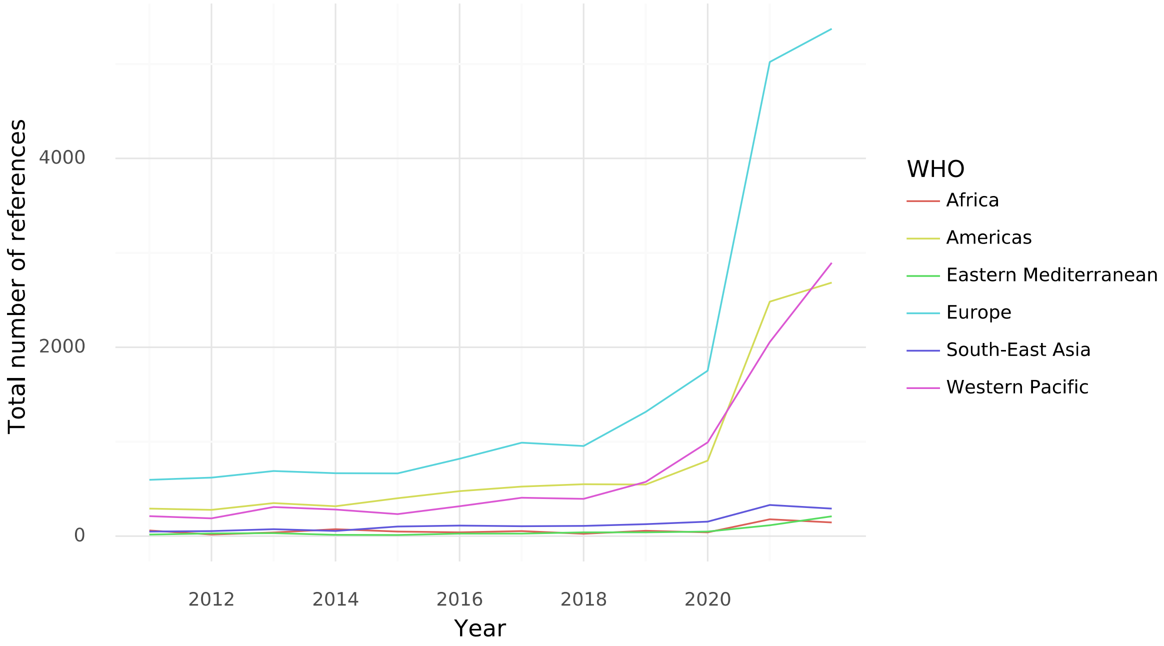

Figure 195. Total references with the intersection of climate change and health by WHO region, 2011-2022.

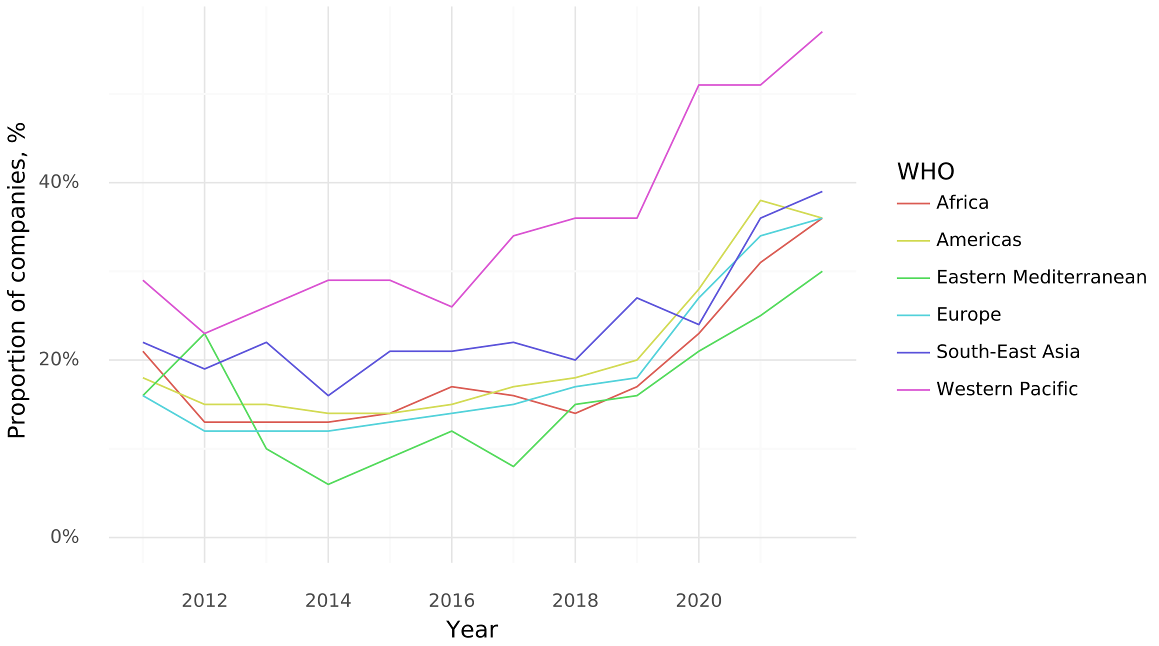

Figure 196. Proportion of companies referring to intersection of health and climate change by WHO region, 2011-2022.

Figure 195 and **Figure 196** show that the highest proportion of GCCOP reports engaging with the climate change-health intersection in recent years has come from corporations based in the Western Pacific. The lowest engagement comes from corporations based in the Eastern Mediterranean region. There has been rising engagement with health and climate change across all the regions.

The indicator also considers engagement across different sectors. Table A3 shows the total number of references to climate change, health, and the intersection across the different sectors in 2022. **Figure 197** presents the proportion of corporations engaging with the climate change-health relations in each sector in 2022.

Sector	intersection	climate	health
Aerospace & Defense	396	3489	7779
Alternative Energy	588	7326	7980
Automobiles & Parts	792	12788	20106
Banks	1014	19053	25927
Basic Resources	0	5	17
Beverages	771	9036	19889
Chemicals	2454	17544	43072
Construction & Materials	1796	23284	55997
Diversified	2621	28677	59166
Electricity	1418	24525	35642
Electronic & Electrical Equ...	755	10854	21283
Equity Investment Instruments	175	1605	2798
Financial Services	2159	35162	42392
Fixed Line Telecommunications	354	4215	10212
Food & Drug Retailers	280	3106	8184

Food Producers	2572	17733	71076
Forestry & Paper	440	7832	9942
Gas, Water & Multiutilities	833	13487	21458
General Industrials	2441	29539	62901
General Retailers	790	13664	27864
Health Care	0	7	50
Health Care Equipment & Ser...	1174	4865	47869
Household Goods & Home Cons...	455	8434	15071
Industrial Engineering	817	9288	19209
Industrial Goods & Services	2	50	675
Industrial Metals & Mining	1051	12438	29758
Industrial Transportation	926	11603	21891
Leisure Goods	97	2366	4001
Life Insurance	749	4122	14059
Media	237	4822	10931
Mining	596	6057	16037
Mobile Telecommunications	539	7410	13738
Nonequity Investment Instru...	26	275	557
Nonlife Insurance	429	4035	11201
Not Applicable	201	3345	16174
Oil & Gas Producers	2126	25404	42037
Oil Equipment, Services & D...	332	3782	8306
Other	0	26	146
Personal Goods	556	6677	15847
Pharmaceuticals & Biotechno...	2253	9054	73826
Real Estate Investment & Se...	807	11032	18265
Real Estate Investment Trusts	405	3105	3769
Retail	3	49	415
Software & Computer Services	983	13004	25394
Support Services	2083	27420	67211
Technology	0	8	51
Technology Hardware & Equip...	824	13657	23083
Tobacco	2	48	137
Travel & Leisure	660	10171	18993

Table 93. Total number of references to health, climate change, and the intersection of climate change and health by sector in 2022.

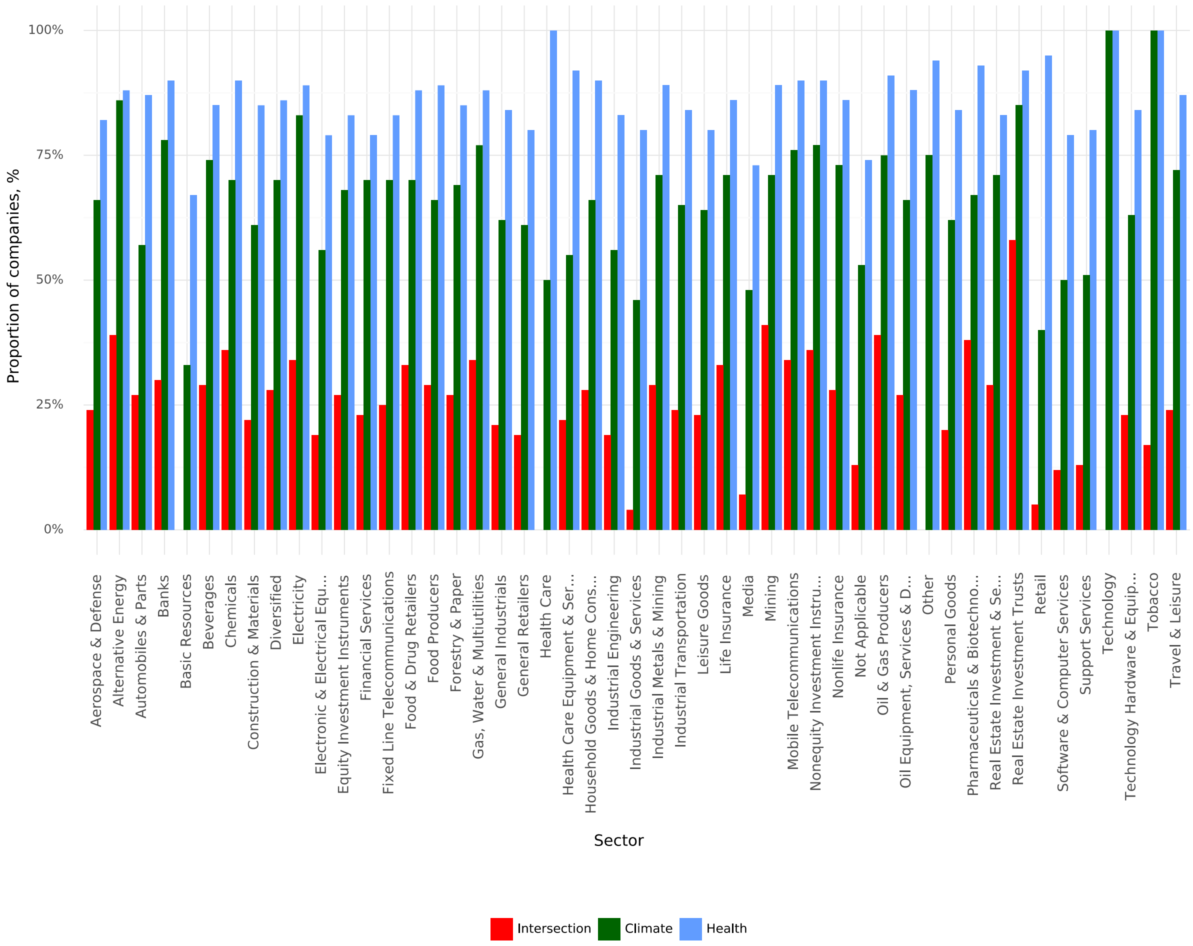

Figure 197. Proportion of corporations referring to climate change, health, and the intersection of climate change and health by sector in 2022.

The highest level of engagement with the intersection of climate change and health in 2022 can be seen in real estate investment trusts (58% of companies), followed by mining (41%), alternative energy (39%), oil & gas producers (39%), and pharmaceuticals and biotechnology companies (38%). In contrast, there are surprisingly lower levels of engagement in the healthcare equipment and services sector, where 33% of companies refer to the intersection of health and climate change in their 2022 GCCOP reports, while companies representing other parts of the health care sector do not refer to the intersection at all (0%). This does, however, represent a significant increase in companies in the healthcare equipment and services sector engaging with health and climate change in the GCCOP reports compared to 2021 (19% in 2021)..

In addition to looking at companies by by WHO region, the indicator also considers companies from different types of countries in terms of their IPCC groupings. As noted in previous years' reports, the SIDS have driven much of the engagement with the health impacts of climate change, as well as climate change more generally, in the international fora. As such, a SIDS grouping is included. These are presented in **Figure 198** and **Figure 199**. The highest proportion of engagement from companies in North America, the SIDS, Oceania, and Asia.

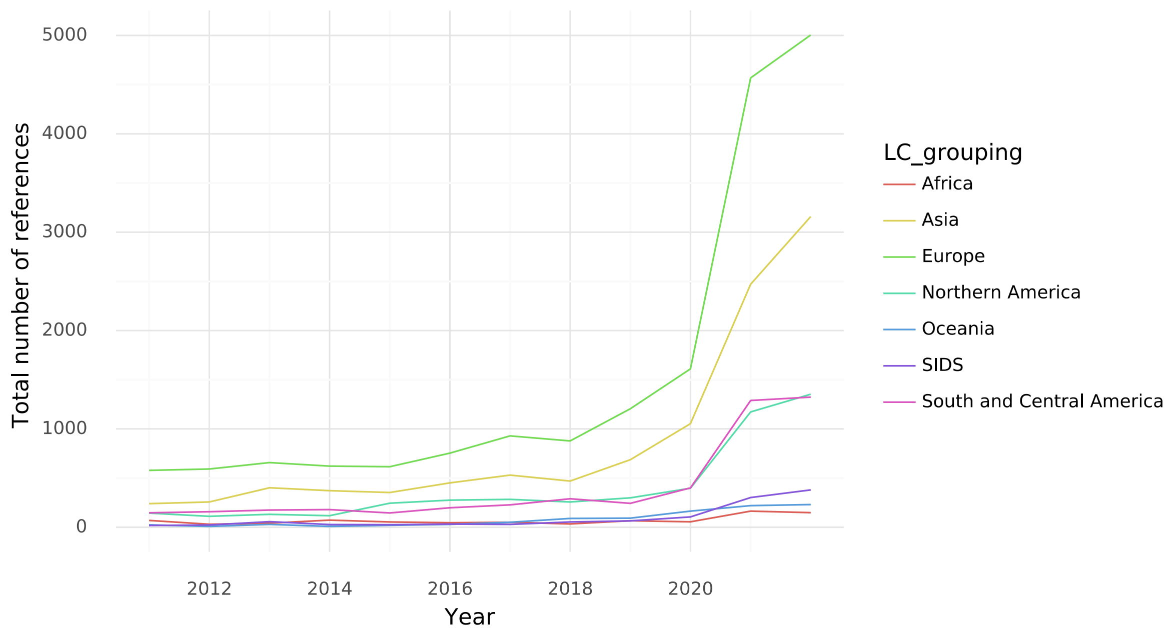

Figure 198. Total references to the climate change-health intersection by country groupings, 2011-2022.

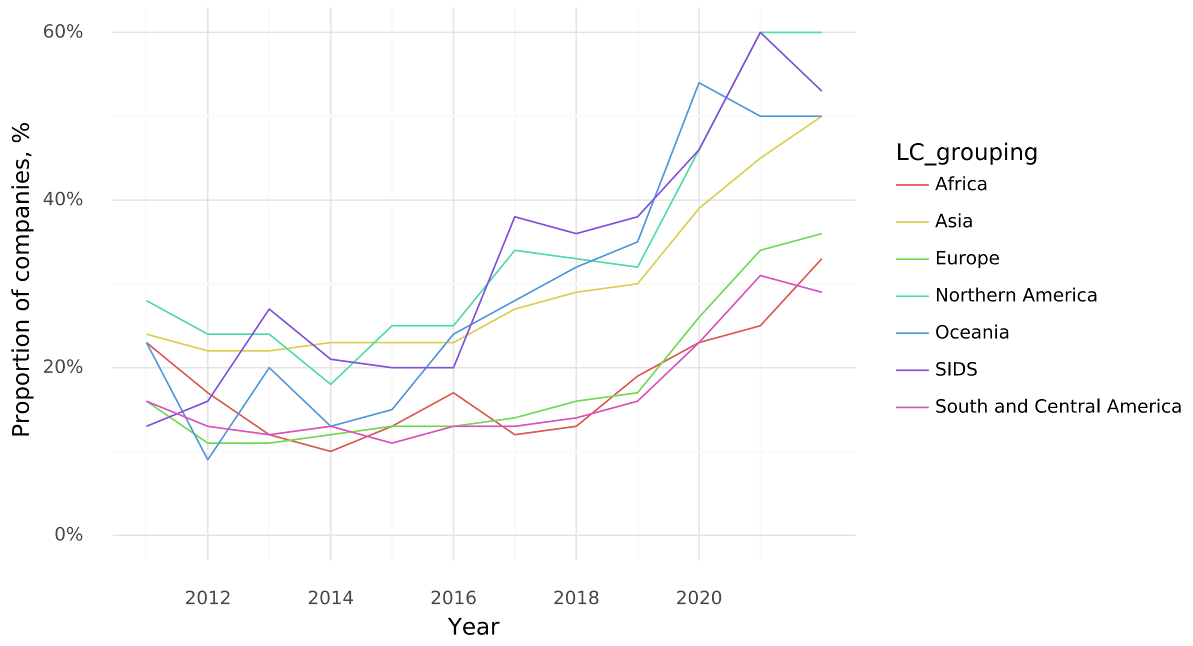

Figure 199. Proportion of corporations referring to the climate change-health intersection by country groupings, 2011-2022.

The indicator also considers corporate engagement with the health dimensions of climate according to the Human Development Index (HDI) categories of the countries in which companies are based. Figure 200 shows the total references to the intersection of climate change and health in companies' GCCOP reports based on the country HDI category (in 2021) and Figure 201 shows the proportion of companies engaging with climate change and health in their GCCOP report by HDI category. Figure 200 shows significantly higher references to climate change and health made by countries based in countries that have very high human development compared to companies based in countries with other levels of human development. However, this reflects the fact that the majority of companies included in the analysis are based in countries with very high human development levels. It is worth noting that even when considering the proportion of companies that engage with climate change and health (Figure 201),

it is the companies based in countries with very high human development that have highest engagement, followed by those with a high HDI. Lower engagement can be seen with climate change and health by companies based in countries with low human development levels.

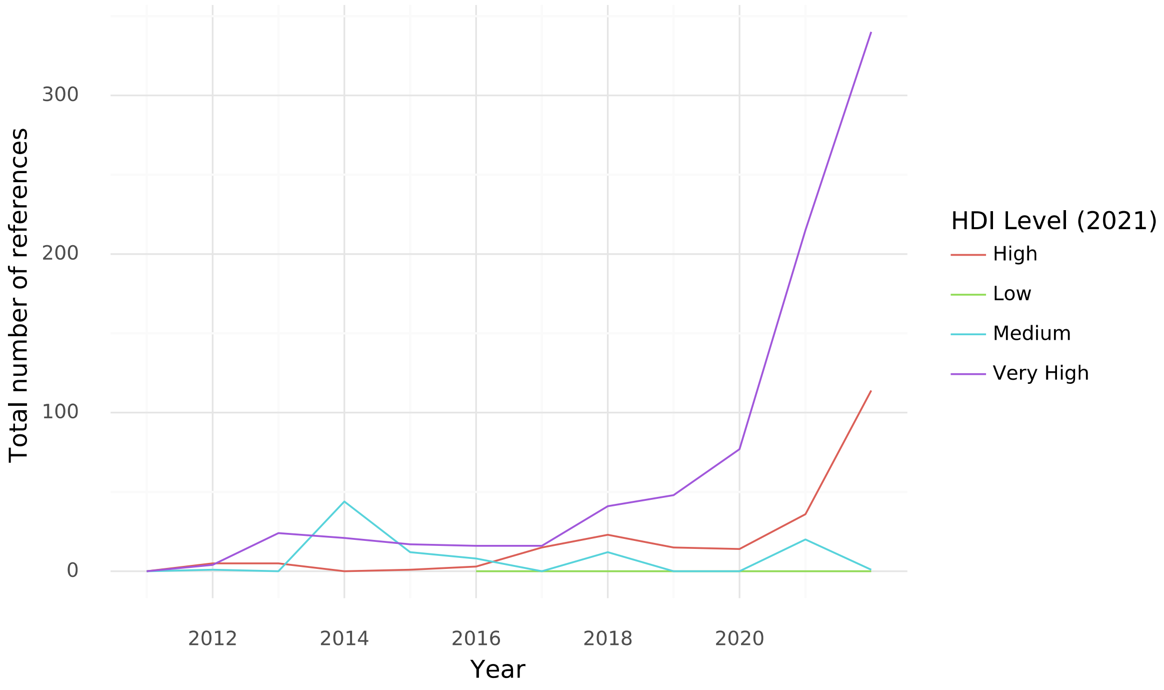

Figure 200. Total references to the climate change-health intersection by country HDI categories, 2011-2022.

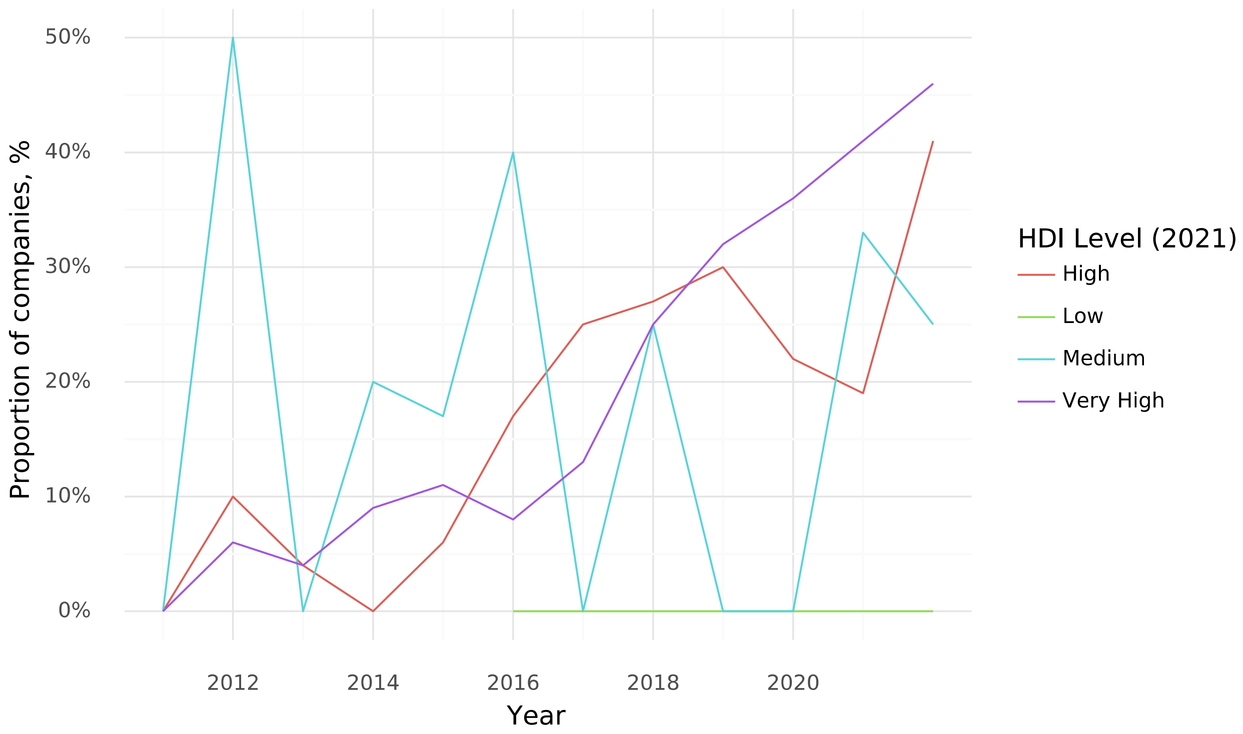

Figure 201. Proportion of corporations referring to the climate change-health intersection by country HDI categories, 2011-2022.

References

1 Lancet Countdown on Health and Climate Change. Our Science. Lancet Countdown. https://www.lancetcountdown.org/our-science/ (accessed April 27, 2023).

2 Masson-Delmotte V P Zhai, A Pirani, SL Connors, C Péan, S Berger, N Caud, Y Chen, L Goldfarb, MI Gomis, M Huang, K Leitzell, E Lonnoy, JBR Matthews, TK Maycock, T Waterfield, O Yelekçi, R Yu, and B Zhou. IPCC, 2021: Climate Change 2021: The Physical Science Basis. Contribution of Working Group I to the Sixth Assessment Report of the Intergovernmental Panel on Climate Change. 2021.

3 Hersbach H, Bell B, Berrisford P, *et al.* ERA5 monthly averaged data on single levels from 1979 to present. Copernicus Climate Change Service (C3S) Climate Data Store (CDS). Available at https://doi.org/10.24381/cds.f17050d7. 2019.

4 NASA Socioeconomic Data and Applications Center (SEDAC) Gridded Population of the World (GPWv4). Available at https://beta.sedac.ciesin.columbia.edu/data/collection/gpw-v4. 2021.

5 ISIMIP. The Inter-Sectoral Impact Model Intercomparison Project (ISIMP). Input data set: Historical, gridded population. Available at https://www.isimip.org/gettingstarted/input-data-bias-correction/details/31/. 2021.

6 de Perez EC, van Aalst M, Bischiniotis K, *et al.* Global predictability of temperature extremes. *Environmental Research Letters* 2018; **13**. DOI:10.1088/1748-9326/aab94a.

7 Di Napoli C, Pappenberger F, Cloke HL. Verification of Heat Stress Thresholds for a Health-Based Heat-Wave Definition. *Journal of Applied Meteorology and Climatology* 2019; **58**: 1177–94.

8 Xu Z, Sheffield PE, Su H, Wang X, Bi Y, Tong S. The impact of heat waves on children’s health: a systematic review. *Int J Biometeorol* 2014; **58**: 239–47.

9 Chambers J. Global and cross-country analysis of exposure of vulnerable populations to heatwaves from 1980 to 2018. *Climatic Change* 2020; **163**: 539–58.

10 Hersbach H, Bell B, Berrisford P, *et al.* ERA5 hourly data on single levels from 1979 to present. Copernicus Climate Change Service (C3S) Climate Data Store (CDS). Available at https://doi.org/10.24381/cds.adbb2d47. 2018.

11 The Inter-Sectoral Impact Model Intercomparison Project. ISIMIP3b Bias Adjustment. 2022. https://www.isimip.org/gettingstarted/isimip3b-bias-adjustment/.

12 Chambers J. Chambers, J. Hybrid gridded demographic data for the world, 1950-2020 0.25˚ resolution. Available at https://doi.org/10.5281/zenodo.6011021. 2022.

13 United Nations. 2020 Revision of World Population Prospects. Available at https://population.un.org/wpp/. 2021.

14 Briggs DJ. A global downscaled age structure data set for assessing human impacts of climate change, 1970-2100. 2021. http://dx.doi.org/10.13140/RG.2.2.15429.17124.

15 Romanello M, Di Napoli C, Drummond P, *et al.* The 2022 report of the Lancet Countdown on health and climate change: health at the mercy of fossil fuels. *The Lancet* 2022; **400**: 1619–54.

16 Jay O, Broderick C, Smallcombe J. Extreme heat policy. Available at https://sma.org.au/sma-site-content/uploads/2021/02/SMA-Extreme-Heat-Policy-2021-Final.pdf, 2021.

17 Thieurmel B, Elmarhraoui A. Package ‘suncalc’. 2022. https://github.com/datastorm-open/suncalc.

18 Luedeling E. Interpolating hourly temperatures for computing agroclimatic metrics. *International journal of biometeorology* 2018; **62**: 1799–807.

19 Center for International Earth Science Information Network - CIESIN - Columbia University. Gridded Population of the World, Version 4 (GPWv4): Population Count Adjusted to Match 2015 Revision of UN WPP Country Totals, Revision 11. 2018. https://doi.org/10.7927/H4PN93PB.

20 Vanos JK, Baldwin JW, Jay O, Ebi KL. Simplicity lacks robustness when projecting heat-health outcomes in a changing climate. *Nat Commun* 2020; **11**: 1–5.

21 Kjellstrom T, Freyberg C, Lemke B, Otto M, Briggs D. Estimating population heat exposure and impacts on working people in conjunction with climate change. *Int J Biometeorol* 2018; **62**: 291–306.

22 Climate Vulnerable Forum (CVF) and Vulnerable Twenty (V20) Group Secretariat, Global Center on Adaptation, Aroha, Climate Analytics, Lancet Countdown. Climate Vulnerability Monitor Third Edition: A Planet on Fire | Knowledge for policy. 2022 https://knowledge4policy.ec.europa.eu/publication/climate-vulnerability-monitor-third-edition-planet-fire_en (accessed Feb 27, 2023).

23 Hersbach H, Bell B, Berrisford P, *et al.* The ERA5 global reanalysis. *Q J Roy Meteor Soc* 2020; **146**: 1999–2049.

24 ILO. ILOSTAT database. 2021. https://ilostat.ilo.org/data/.

25 WHO, ILO. WHO/ILO joint estimates of the work-related burden of disease and injury, 2000-2016: Technical Report. Geneva: World Health Organization and International Labour Organization, 2021.

26 WHO, ILO. WHO/ILO joint estimates of the work-related burden of disease and injury, 2000-2016: Global Monitoring Report. Geneva: World Health Organization, 2021.

27 Pega F, Náfrádi B, Momen NC, *et al.* Global, regional, and national burdens of ischemic heart disease and stroke attributable to exposure to long working hours for 194 countries, 2000–2016: A systematic analysis from the WHO/ILO Joint Estimates of the Work-related Burden of Disease and Injury. *Environment International* 2021; **154**: 106595.

28 Pega F, Hamzaoui H, Náfrádi B, Momen NC. Global, regional and national burden of disease attributable to 19 selected occupational risk factors for 183 countries, 2000–2016: A systematic analysis from the WHO/ILO Joint Estimates of the Work-related Burden of Disease and Injury. *Scandinavian journal of work, environment & health* 2022; **48**: 158.

29 WHO. Handbook on health inequality monitoring: with a special focus on low-and middle-income countries. World Health Organization, 2013.

30 Pega F, Momen NC, Streicher KN. Global, regional and national burdens of non-melanoma skin cancer attributable to occupational exposure to solar ultraviolet radiation for 183 countries, 2000–2019: A systematic analysis from the WHO/ILO Joint Estimates of the Work-related Burden of Disease and Injury. *Environ Int* 2023; : 108226.

31 WHO. Technical Advisory Group on Occupational Burden of Disease Estimation (TAG-OBoDE). 2023. https://www.who.int/groups/technical-advisory-group-on-occupational-burden-of-disease-estimation.

32 ILO. ISCO–08: International Standard Classification of Occupations. Geneva: International Labour Organization, 2012.

33 ILO. ISCO–88: International Standard Classification of Occupations. Geneva: International Labour Organization, 1987.

34 WHO. WHO Coronavirus (COVID-19) Dashboard. 2023; published online March 22. https://covid19.who.int/.

35 Bonjour S, Adair-Rohani H, Wolf J, *et al.* Solid fuel use for household cooking: country and regional estimates for 1980–2010. *Environmental health perspectives* 2013; **121**: 784–90.

36 Wolf J, Bonjour S, Prüss-Ustün A. An exploration of multilevel modeling for estimating access to drinking-water and sanitation. *Journal of Water and Health* 2013; **11**: 64–77.

37 Stoner O, Lewis J, Martínez IL, Gumy S, Economou T, Adair-Rohani H. Household cooking fuel estimates at global and country level for 1990 to 2030. *Nature communications* 2021; **12**: 5793.

38 UN. SDG indicator metadata. Indicator 3.9.1 Mortality rate attributed to household and ambient air pollution. 2022 https://unstats.un.org/sdgs/metadata/files/Metadata-03-09-01.pdf.

39 UN. 2022 Revision of World Population Prospects. 2022. https://population.un.org/wpp/Download/Standard/Population/ (accessed March 15, 2023).

40 Pega F, Momen NC, Abubakar A. Monitoring workers' health: focus on rights, determinants, and equity. *The Lancet* 2023; **402**: 1306–8.

41 Romanello M, Napoli CD, Drummond P, *et al.* The 2022 report of the Lancet Countdown on health and climate change: health at the mercy of fossil fuels. *The Lancet* 2022; **400**: 1619–54.

42 Perishing A, Gilford D, Giguere J. Climate Central Methods Temperature Attribution System. 2022; published online June 14. https://assets.ctfassets.net/cxgxgstp8r5d/4PyuCIhY6ZT5LEGyfq01rc/9b81e5f911baad59575550e4dd549f57/Climate_Central_Methods_Temperature_Attribution_System_June-2022.pdf (accessed Jan 29, 2023).

43 Honda Y, Kondo M, McGregor G, *et al.* Heat-related mortality risk model for climate change impact projection. *Environ Health Prev Med* 2014; **19**: 56–63.

44 Global Burden of Disease Collaborative Network. Global Burden of Disease Study 2019 (GBD 2019) Results. Seattle, United States: Institute for Health Metrics and Evaluation (IHME), 2020. Available from http://ghdx.healthdata.org/gbd-results-tool. http://ghdx.healthdata.org/gbd-results-tool.

45 FIRMS. Fire Information for Resource Management System (FIRMS). Fire Information for Resource Management System (FIRMS). 2023. https://www.earthdata.nasa.gov/learn/find-data/near-real-time/firms (accessed Feb 23, 2023).

46 Wilson AM, Jetz W. Remotely Sensed High-Resolution Global Cloud Dynamics for Predicting Ecosystem and Biodiversity Distributions. *PLoS Biol* 2016; **14**: e1002415.

47 EarthEnv. EarthEnv. Global, remote-sensing supported environmental layers for assessing status and trends in biodiversity, ecosystems, and climate. 2023. https://www.earthenv.org/cloud (accessed Feb 23, 2023).

48 CMES. Fire danger indices historical data from the Copernicus Emergency Management Service. 2019. DOI:10.24381/CDS.0E89C522.

49 CIESIN. Documentation for Gridded Population of the World, Version 4 (GPWv4). *Center For International Earth Science Information Network-CIESIN-Columbia University* 2016. DOI:10.7927/H4D50JX4.

50 Gao J, Pesaresi M. Global 1-km Downscaled Urban Land Extent Projection and Base Year Grids by SSP Scenarios, 2000–2100. *NASA Socioeconomic Data and Applications Center (SEDAC)* 2021; **3**.

51 Hänninen R, Sofiev M, Uppstu A, Kouznetsov R. Daily surface concentration of fire related PM2.5 for 2003-2021, modelled by SILAM CTM when using the MODIS satellite data for the fire radiative power. 2022; : 0 file, 0.0 B.

52 Sofiev M, Vankevich R, Lotjonen M, *et al.* An operational system for the assimilation of the satellite information on wild-land fires for the needs of air quality modelling and forecasting. *Atmospheric Chemistry and Physics* 2009; **9**: 6833–47.

53 Sofiev M, Vankevich R, Ermakova T, Hakkarainen J. Global mapping of maximum emission heights and resulting vertical profiles of wildfire emissions. *Atmospheric Chemistry and Physics* 2013; **13**: 7039–52.

54 Sofiev M, Ermakova T, Vankevich R. Evaluation of the smoke-injection height from wild-land fires using remote-sensing data. *Atmospheric Chemistry and Physics* 2012; **12**: 1995–2006.

55 Soares J, Sofiev M, Hakkarainen J. Uncertainties of wild-land fi res emission in AQMEII phase 2 case study. *Atmospheric Environment* 2015. DOI:10.1016/j.atmosenv.2015.01.068.

56 Sofiev M, Vira J, Kouznetsov R, Prank M, Soares J, Genikhovich E. Construction of an Eulerian atmospheric dispersion model based on the advection algorithm of M. Galperin: dynamic cores v.4 and 5 of SILAM v.5.5. *Geoscientific Model Development* 2015; **8**: 3497–522.

57 Kollanus V, Prank M, Gens A, *et al.* Mortality due to Vegetation Fire–Originated PM $_{2.5}$ Exposure in Europe—Assessment for the Years 2005 and 2008. *Environmental Health Perspectives* 2017; **125**: 30–7.

58 Wooster M, Zhukov B, Oertel D. Fire radiative energy for quantitative study of biomass burning: derivation from the BIRD experimental satellite and comparison to MODIS fire products. *Remote Sensing of Environment* 2003; **86**: 83–107.

59 Wooster MJ, Roberts G, Perry GLW, Kaufman YJ. Retrieval of biomass combustion rates and totals from fire radiative power observations: FRP derivation and calibration relationships between biomass consumption and fire radiative energy release. *J Geophys Res* 2005; **110**: D24311.

60 Beukes JP, Van Zyl PG, Sofiev M, *et al.* The use of satellite observations of fire radiative power to estimate the availabilities (activity patterns) of pyrometallurgical smelters. *J S Afr Inst Min Metall* 2018; **118**. DOI:10.17159/2411-9717/2018/v118n6a9.

61 Beguería S, Vicente-Serrano SM, Reig F, Latorre B. Standardized precipitation evapotranspiration index (SPEI) revisited: parameter fitting, evapotranspiration models, tools, datasets and drought monitoring. *International Journal of Climatology* 2014; **34**: 3001–23.

62 Fan Y, Van den Dool H. A global monthly land surface air temperature analysis for 1948–present. *Journal of Geophysical Research: Atmospheres* 2008; **113**.

63 Schamm K, Ziese M, Becker A, *et al.* Global gridded precipitation over land: a description of the new GPCC First Guess Daily product. *Earth System Science Data* 2014; **6**: 49–60.

64 MeteoSwiss. SPI and SPEI. 2018. https://www.meteoswiss.admin.ch/home/climate/swiss-climate-in-detail/climate-indicators/drought-indices/spi-and-spei.html.

65 Global SPEI database. Available at https://spei.csic.es/database.html. 2023. https://spei.csic.es/database.html.

66 Baylis P, Obradovich N, Kryvasheyeu Y, *et al.* Weather impacts expressed sentiment. *PLOS ONE* 2018; **13**: e0195750.

67 Baylis P. Temperature and temperament: Evidence from Twitter. *Journal of Public Economics* 2020; **184**: 104161.

68 Wang J, Obradovich N, Zheng S. A 43-Million-Person Investigation into Weather and Expressed Sentiment in a Changing Climate. *One Earth* 2020; **2**: 568–77.

69 Minor K, Moro E, Obradovich N. Adverse weather amplifies social media activity. 2023; published online Feb 16. http://arxiv.org/abs/2302.08456 (accessed March 3, 2023).

70 Noelke C, McGovern M, Corsi DJ, *et al.* Increasing ambient temperature reduces emotional well-being. *Environmental Research* 2016; **151**: 124–9.

71 Romanello M, McGushin A, Napoli CD, *et al.* The 2021 report of the Lancet Countdown on health and climate change: code red for a healthy future. *The Lancet* 2021; **398**: 1619–62.

72 Burke M, González F, Baylis P, *et al.* Higher temperatures increase suicide rates in the United States and Mexico. *Nature Clim Change* 2018; **8**: 723–9.

73 Moore FC, Obradovich N, Lehner F, Baylis P. Rapidly declining remarkability of temperature anomalies may obscure public perception of climate change. *PNAS* 2019; **116**: 4905–10.

74 Carleton TA, Hsiang SM. Social and economic impacts of climate. *Science* 2016; **353**: aad9837–aad9837.

75 Hsiang S. Climate Econometrics. *Annual Review of Resource Economics* 2016; **8**: 43–75.

76 Diener E, Larsen RJ, Levine S, Emmons RA. Intensity and frequency: Dimensions underlying positive and negative affect. *Journal of Personality and Social Psychology* 1985; **48**: 1253–65.

77 Pennebaker JW, Boyd RL, Jordan K, Blackburn K. The Development and Psychometric Properties of LIWC2015. ; : 26.

78 Pennebaker JW, Chung CK, Ireland M, Gonzales A, Booth RJ. The Development and Psychometric Properties of LIWC2007. ; : 22.

79 Tausczik YR, Pennebaker JW. The Psychological Meaning of Words: LIWC and Computerized Text Analysis Methods. *Journal of Language and Social Psychology* 2010; **29**: 24–54.

80 Coppersmith G, Dredze M, Harman C. Quantifying Mental Health Signals in Twitter. In: Proceedings of the Workshop on Computational Linguistics and Clinical Psychology: From Linguistic Signal to Clinical Reality. Baltimore, Maryland, USA: Association for Computational Linguistics, 2014: 51–60.

81 Kahn JH, Tobin RM, Massey AE, Anderson JA. Measuring Emotional Expression with the Linguistic Inquiry and Word Count. *The American Journal of Psychology* 2007; **120**: 263–86.

82 Beasley A, Mason W. Emotional States vs. Emotional Words in Social Media. In: Proceedings of the ACM Web Science Conference. New York, NY, USA: Association for Computing Machinery, 2015: 1–10.

83 Settanni M, Marengo D. Sharing feelings online: studying emotional well-being via automated text analysis of Facebook posts. *Frontiers in Psychology* 2015; **6**: 1045.

84 Hersbach H, Bell B, Berrisford P, *et al.* The ERA5 global reanalysis. *Quarterly Journal of the Royal Meteorological Society* 2020; **146**: 1999–2049.

85 Myhre G, Alterskjær K, Stjern CW, *et al.* Frequency of extreme precipitation increases extensively with event rareness under global warming. *Sci Rep* 2019; **9**: 16063.

86 Dunn RJH, Alexander LV, Donat MG, *et al.* Development of an Updated Global Land In Situ-Based Data Set of Temperature and Precipitation Extremes: HadEX3. *Journal of Geophysical Research: Atmospheres* 2020; **125**: e2019JD032263.

87 Contractor S, Donat MG, Alexander LV. Changes in Observed Daily Precipitation over Global Land Areas since 1950. *Journal of Climate* 2021; **34**: 3–19.

88 Alexander LV. Global observed long-term changes in temperature and precipitation extremes: A review of progress and limitations in IPCC assessments and beyond. *Weather and Climate Extremes* 2016; **11**: 4–16.

89 Obradovich N, Tingley D, Rahwan I. Effects of environmental stressors on daily governance. *PNAS* 2018; **115**: 8710–5.

90 Obradovich N, Migliorini R, Paulus MP, Rahwan I. Empirical evidence of mental health risks posed by climate change. *PNAS* 2018; **115**: 10953–8.

91 Obradovich N, Rahwan I. Risk of a feedback loop between climatic warming and human mobility. *Journal of The Royal Society Interface* 2019; **16**: 20190058.

92 Minor K, Bjerre-Nielsen A, Jonasdottir SS, Lehmann S, Obradovich N. Rising temperatures erode human sleep globally. *One Earth* 2022; **5**: 534–49.

93 Nations U. Human Development Report 2021-22. United Nations, 2022 https://hdr.undp.org/content/human-development-report-2021-22 (accessed March 3, 2023).

94 Copernicus Climate Change Service. E-OBS daily gridded meteorological data for Europe from 1950 to present derived from in-situ observations. 2020. DOI:10.24381/CDS.151D3EC6.

95 Pinto I, Zachariah M, Wolski P, *et al.* Climate change exacerbated rainfall causing devastating flooding in Eastern South Africa. *World Weather Atribution* 2022.

96 Zachariah M, Vautard R, Schumacher DL, *et al.* Without human-caused climate change temperatures of 40oC in the UK would have been extremely unlikely. *World Weather Atribution* 2022.

97 Factsheet about West Nile virus infection. https://www.ecdc.europa.eu/en/west-nile-fever/facts (accessed March 5, 2023).

98 Epidemiological update: West Nile virus transmission season in Europe, 2018. 2018; published online Dec 14. https://www.ecdc.europa.eu/en/news-events/epidemiological-update-west-nile-virus-transmission-season-europe-2018 (accessed March 5, 2023).

99 Ronca SE, Ruff JC, Murray KO. A 20-year historical review of West Nile virus since its initial emergence in North America: Has West Nile virus become a neglected tropical disease? *PLoS Negl Trop Dis* 2021; **15**: e0009190.

100 Heidecke J, Schettini AL, Rocklöv J. West Nile virus eco-epidemiology and climate change. *PLOS Climate* 2023; **2**: e0000129.

101 Shocket MS, Verwillow AB, Numazu MG, *et al.* Transmission of West Nile and five other temperate mosquito-borne viruses peaks at temperatures between 23°C and 26°C. *eLife* 2020; **9**: e58511.

102 Farooq Z, Rocklöv J, Wallin J, *et al.* Artificial intelligence to predict West Nile virus outbreaks with eco-climatic drivers. *The Lancet Regional Health - Europe* 2022; **17**: 100370.

103 Marini G, Pugliese A, Wint W, Alexander NS, Rizzoli A, Rosà R. Modelling the West Nile virus force of infection in the European human population. *One Health* 2022; **15**: 100462.

104 Paz S. Climate change impacts on West Nile virus transmission in a global context. *Phil Trans R Soc B* 2015; **370**: 20130561.

105 Di Pol G, Crotta M, Taylor RA. Modelling the temperature suitability for the risk of West Nile Virus establishment in European *Culex pipiens* populations. *Transbounding Emerging Dis* 2022; **69**. DOI:10.1111/tbed.14513.

106 Lourenço J, Barros SC, Zé-Zé L, *et al.* West Nile virus transmission potential in Portugal. *Commun Biol* 2022; **5**: 6.

107 Smith DL, Battle KE, Hay SI, Barker CM, Scott TW, McKenzie FE. Ross, Macdonald, and a Theory for the Dynamics and Control of Mosquito-Transmitted Pathogens. *PLOS Pathogens* 2012; **8**: e1002588.

108 DiSera L, Sjödin H, Rocklöv J, *et al.* The Mosquito, the Virus, the Climate: An Unforeseen Réunion in 2018. *GeoHealth* 2020; **4**. DOI:10.1029/2020GH000253.

109 Muñoz-Sabater J, Dutra E, Agustí-Panareda A, *et al.* ERA5-Land: a state-of-the-art global reanalysis dataset for land applications. *Earth Syst Sci Data* 2021; **13**: 4349–83.

110 Muñoz Sabater J. ERA5-Land monthly averaged data from 1950 to 1980. 2021.

111 Muñoz Sabater J. ERA5-Land monthly averaged data from 1981 to present. 2019.

112 Muñoz Sabater J. ERA5-Land monthly averaged data from 1981 to present. Copernicus Climate Change Service (C3S) Climate Data Store (CDS). Available at https://doi.org/10.24381/cds.68d2bb30. 2019.

113 Stanaway JD, Shepard DS, Undurraga EA, *et al.* The global burden of dengue: an analysis from the Global Burden of Disease Study 2013. *The Lancet infectious diseases* 2016; **16**: 712–23.

114 Hales S, De Wet N, Maindonald J, Woodward A. Potential effect of population and climate changes on global distribution of dengue fever: an empirical model. *The Lancet* 2002; **360**: 830–4.

115 Rocklöv J, Tozan Y. Climate change and the rising infectiousness of dengue. *Emerging Topics in Life Sciences* 2019; **3**: 133–42.

116 Rocklöv J, Quam MB, Sudre B, *et al.* Assessing seasonal risks for the introduction and mosquito-borne spread of Zika virus in Europe. *EBioMedicine* 2016; **9**: 250–6.

117 Rocklöv J, Tozan Y, Ramadona A, *et al.* Using big data to monitor the introduction and spread of Chikungunya, Europe, 2017. *Emerging infectious diseases* 2019; **25**: 1041.

118 Colón-González FJ, Sewe MO, Tompkins AM, *et al.* Projecting the risk of mosquito-borne diseases in a warmer and more populated world: a multi-model, multi-scenario intercomparison modelling study. *The Lancet Planetary Health* 2021; **5**: e404–14.

119 Liu-Helmersson J, Quam M, Wilder-Smith A, *et al.* Climate change and Aedes vectors: 21st century projections for dengue transmission in Europe. *EBioMedicine* 2016; **7**: 267–77.

120 Liu-Helmersson J, Stenlund H, Wilder-Smith A, Rocklöv J. Vectorial capacity of Aedes aegypti: effects of temperature and implications for global dengue epidemic potential. *PloS one* 2014; **9**: e89783.

121 Patz JA, Graczyk TK, Geller N, Vittor AY. Effects of environmental change on emerging parasitic diseases. *International Journal for Parasitology* 2000; **30**: 1395–405.

122 Boualam MA, Pradines B, Drancourt M, Barbieri R. Malaria in Europe: A Historical Perspective. *Frontiers in Medicine* 2021; **8**. DOI:10.3389/FMED.2021.691095/FULL.

123 Grover-Kopec EK, Blumenthal MB, Ceccato P, Dinku T, Omumbo JA, Connor SJ. Web-based climate information resources for malaria control in Africa. *Malaria journal* 2006; **5**. DOI:10.1186/1475-2875-5-38.

124 Laporta GZ, Linton YM, Wilkerson RC, *et al.* Malaria vectors in South America: Current and future scenarios. *Parasites and Vectors* 2015; **8**: 1–13.

125 Sinka ME, Rubio-Palis Y, Manguin S, *et al.* The dominant Anopheles vectors of human malaria in the Americas: occurrence data, distribution maps and bionomic précis. *Parasites & Vectors 2010 3:1* 2010; **3**: 1–26.

126 Lyon B, Dinku T, Raman A, Thomson MC. Temperature suitability for malaria climbing the Ethiopian Highlands. *Environmental Research Letters* 2017; **12**: 064015.

127 Muñoz-Sabater J, Dutra E, Agustí-Panareda A, *et al.* ERA5-Land: A state-of-the-art global reanalysis dataset for land applications. *Earth System Science Data* 2021; **13**: 4349–83.

128 Alduchov O, Meteorology RE-J of A, 1996 U. Improved Magnus form approximation of saturation vapor pressure. *journals.ametsoc.org* 1996. https://journals.ametsoc.org/view/journals/apme/35/4/1520-0450_1996_035_0601_imfaos_2_0_co_2.xml.

129 Guerra CA, Snow RW, Hay SI. A global assessment of closed forests, deforestation and malaria risk. *Ann Trop Med Parasitol* 2006; **100**: 189–204.

130 Patz JA, Graczyk TK, Geller N, Vittor AY. Effects of environmental change on emerging parasitic diseases. *International Journal for Parasitology* 2000; **30**: 1395–405.

131 Gething PW, Patil AP, Smith DL, *et al.* A new world malaria map: Plasmodium falciparum endemicity in 2010. *Malaria Journal* 2011; **10**: 1–16.

132 Snow RW, Sartorius B, Kyalo D, *et al.* The prevalence of Plasmodium falciparum in sub-Saharan Africa since 1900. *Nature* 2017; **550**: 515–8.

133 Asenso-Okyere K, Asante F, Tarekegn J, Andam K. The linkages between agriculture and malaria: Issues for policy, research, and capacity strengthening. 2009. https://www.semanticscholar.org/paper/The-linkages-between-agriculture-and-malaria%3A-for-Asenso-Okyere-Asante/3b6e03f48df78605e48ac18f97355fa43ca859a2?p2df (accessed Feb 8, 2023).

134 Baker-Austin C, Oliver JD, Alam M, *et al.* Vibrio spp. infections. *Nat Rev Dis Primers* 2018; **4**: 8.

135 Martinez-Urtaza J, van Aerle R, Abanto M, *et al.* Genomic Variation and Evolution of Vibrio parahaemolyticus ST36 over the Course of a Transcontinental Epidemic Expansion. *mBio* 2017; **8**. DOI:10.1128/mBio.01425-17.

136 Parveen S, Hettiarachchi KA, Bowers JC, *et al.* Seasonal distribution of total and pathogenic Vibrio parahaemolyticus in Chesapeake Bay oysters and waters. *Int J Food Microbiol* 2008; **128**: 354–61.

137 Baker-Austin C, Trinanes JA, Taylor NG, Hartnell R, Siitonen A, Martinez-Urtaza J. Emerging Vibrio risk at high latitudes in response to ocean warming. *Nature climate change* 2013; **3**: 73–7.

138 Semenza JC, Trinanes J, Lohr W, *et al.* Environmental suitability of Vibrio infections in a warming climate: an early warning system. *Environmental health perspectives* 2017; **125**: 107004.

139 Trinanes J, Martinez-Urtaza J. Future scenarios of risk of Vibrio infections in a warming planet: a global mapping study. *The Lancet Planetary Health* 2021; **5**: e426–35.

140 Eyring V, Bony S, Meehl GA, *et al.* Overview of the Coupled Model Intercomparison Project Phase 6 (CMIP6) experimental design and organization. *Geoscientific Model Development* 2016; **9**: 1937–58.

141 Warszawski L, Frieler K, Huber V, Piontek F, Serdeczny O, Schewe J. The inter-sectoral impact model intercomparison project (ISI–MIP): project framework. *Proceedings of the National Academy of Sciences* 2014; **111**: 3228–32.

142 Newton A, Kendall M, Vugia DJ, Henao OL, Mahon BE. Increasing rates of vibriosis in the United States, 1996–2010: review of surveillance data from 2 systems. *Clinical Infectious Diseases* 2012; **54**: S391–5.

143 Ralston EP, Kite-Powell H, Beet A. An estimate of the cost of acute health effects from food-and water-borne marine pathogens and toxins in the USA. *Journal of water and health* 2011; **9**: 680–94.

144 Riahi K, van Vuuren DP, Kriegler E, *et al.* The Shared Socioeconomic Pathways and their energy, land use, and greenhouse gas emissions implications: An overview. *Global Environmental Change* 2017; **42**: 153–68.

145 Good S, Fiedler E, Mao C, *et al.* The current configuration of the OSTIA system for operational production of foundation sea surface temperature and ice concentration analyses. *Remote Sensing* 2020; **12**: 720.

146 Copernicus Marine Environment Monitoring Service. Mercator Ocean Reanalysis. Available at http://marine.copernicus.eu/. 2021.

147 WCRP. CMIP6. 2023. https://esgf-node.llnl.gov/search/cmip6/.

148 Jones B, O'Neill BC. Spatially explicit global population scenarios consistent with the Shared Socioeconomic Pathways. *Environmental Research Letters* 2016; **11**: 084003.

149 CIA. The World Factbook. 2023. https://www.cia.gov/the-world-factbook/.

150 Dasgupta S, Robinson EJZ. Attributing changes in food insecurity to a changing climate. *Scientific Reports* 2022; **12**: 4709.

151 Monfreda C, Ramankutty N, Foley JA. Farming the planet: 2. Geographic distribution of crop areas, yields, physiological types, and net primary production in the year 2000. *Global Biogeochemical Cycles* 2008; **22**. DOI:https://doi.org/10.1029/2007GB002947.

152 Muñoz-Sabater J, Dutra E, Agustí-Panareda A, *et al.* ERA5-Land: A state-of-the-art global reanalysis dataset for land applications. *Earth System Science Data* 2021; **13**: 4349–83.

153 Dasgupta S, Robinson EJZ. Improving Food Policies for a Climate Insecure World: Evidence from Ethiopia. *National Institute Economic Review* 2021; **258**: 66–82.

154 Vicente-Serrano SM, Beguería S, López-Moreno JI. A Multiscalar Drought Index Sensitive to Global Warming: The Standardized Precipitation Evapotranspiration Index. *Journal of Climate* 2010; **23**: 1696–718.

155 Cafiero C, Viviani S, Nord M. Food security measurement in a global context: The food insecurity experience scale. *Measurement* 2018; **116**: 146–52.

156 Dasgupta S, Maanen N van, Gosling SN, Piontek F, Otto C, Schleussner C-F. Effects of climate change on combined labour productivity and supply: an empirical, multi-model study. *The Lancet Planetary Health* 2021; **5**: e455–65.

157 Muñoz Sabater J. ERA5-Land hourly data from 1950 to present. Copernicus Climate Change Service (C3S) Climate Data Store (CDS). Available at https://doi.org/10.24381/cds.e2161bac. 2019.

158 Cafiero C, Viviani S, Nord M. Food security measurement in a global context: The food insecurity experience scale. *Measurement* 2018; **116**: 146–52.

159 Copernicus Climate Change Service (C3S). ORAS5 global ocean reanalysis monthly data from 1958 to present. Available at https://doi.org/10.24381/cds.67e8eeb7. 2023.

160 FAO. New Food Balance Sheets. Available at http://www.fao.org/faostat/en/#data/FBS. 2021.

161 World Health Organization. Alliance for action on climate change and health (ATACH). https://www.who.int/initiatives/alliance-for-transformative-action-on-climate-and-health (accessed July 6, 2023).

162 WHO. 2021 WHO Health and Climate Change Survey Report. Geneva, 2021 https://www.who.int/publications-detail-redirect/9789240038509 (accessed April 2, 2023).

163 2022 Full Cities Dataset | CDP Open Data Portal. https://data.cdp.net/Governance/2022-Full-Cities-Dataset/gd5v-pfcg (accessed April 2, 2023).

164 World Meteorological Organization. World Meteorological Organization Country Profile database. 2021.

165 Florczyk A., Corbane C,. Schiavina M., Pesaresi M., Maffenini L., Melchiorri, M., Politis P., Sabo F., Freire S., Ehrlich D., Kemper T., Tommasi P., Airaghi D., Zanchetta L. GHS Urban Centre Database 2015, multitemporal and multidimensional attributes, R2019A. European Commission, Joint Research Centre (JRC). https://data.jrc.ec.europa.eu/dataset/53473144-b88c-44bc-b4a3-4583ed1f547e (accessed April 3, 2023).

166 Center for International Earth Science Information Network - CIESIN, - Columbia University. Gridded Population of the World, Version 4 (GPWv4): Population Density Adjusted to Match 2015 Revision UN WPP Country Totals, Revision 11. 2018. https://doi.org/10.7927/H4F47M65.

167 Kriegler F, Malila W, Nalepka R, Richardson W. Preprocessing Transformations and Their Effects on Multispectral Recognition. In: Proceedings of the Sixth International Symposium on Remote Sensing of Environment. 1969: 97.

168 Landsat Missions. Landsat Data Access | U.S. Geological Survey. https://www.usgs.gov/landsat-missions/landsat-data-access (accessed April 3, 2023).

169 Beck HE, Zimmermann NE, McVicar TR, Vergopolan N, Berg A, Wood EF. Present and future Köppen-Geiger climate classification maps at 1-km resolution. *Sci Data* 2018; **5**: 180214.

170 NASA Socioeconomic Data and Applications Center (SEDAC) Gridded Population of the World (GPWv4). Available at https://beta.sedac.ciesin.columbia.edu/data/collection/gpw-v4. 2021.

171 Rugel EJ, Henderson SB, Carpiano RM, Brauer M. Beyond the Normalized Difference Vegetation Index (NDVI): Developing a Natural Space Index for population-level health research. *Environmental Research* 2017; **159**: 474–83.

172 Fong KC, Hart JE, James P. A Review of Epidemiologic Studies on Greenness and Health: Updated Literature Through 2017. *Curr Environ Health Rep* 2018; **5**: 77–87.

173 World Health Organization. Electronic IHR States Parties Self-Assessment Annual Reporting Tool. https://extranet.who.int/e-spar (accessed April 28, 2023).

174 World Health Organization. WHO Simulation Exercise Manual. Geneva: World Health Organization, 2017 https://extranet.who.int/sph/sites/default/files/document-library/document/WHO-WHE-CPI-2017.10-eng.pdf (accessed April 3, 2023).

175 Watts N, Amann M, Arnell N, *et al.* The 2020 report of The Lancet Countdown on health and climate change: responding to converging crises. *The Lancet* 2021; **397**: 129–70.

176 Centre for Research on the Epidemiology of Disasters. EM-DAT The International Disaster Database. 2022. https://www.emdat.be/.

177 Nations U. Human Development Index. United Nations https://hdr.undp.org/data-center/human-development-index (accessed April 3, 2023).

178 Kulp SA, Strauss BH. CoastalDEM: A global coastal digital elevation model improved from SRTM using a neural network. *Remote Sensing of Environment* 2018; **206**: 231–9.

179 Chambers J. Hybrid gridded demographic data for the world, 1950-2020 0.25˚ resolution. 2022; published online Feb 8. DOI:10.5281/zenodo.6011021.

180 Kopp RE, DeConto RM, Bader DA, *et al.* Evolving Understanding of Antarctic Ice-Sheet Physics and Ambiguity in Probabilistic Sea-Level Projections. *Earth's Future* 2017; **5**: 1217–33.

181 Cazenave A, Moreira L. Contemporary sea-level changes from global to local scales: a review. *Proceedings of the Royal Society A: Mathematical, Physical and Engineering Sciences* 2022; **478**: 20220049.

182 Kulp SA, Strauss BH. New elevation data triple estimates of global vulnerability to sea-level rise and coastal flooding. *Nat Commun* 2019; **10**: 4844.

183 Oppenheimer, M., B.C. Glavovic , J. Hinkel, R. van de Wal, A.K. Magnan, A. Abd-Elgawad, R. Cai, M. Cifuentes-Jara, R.M., DeConto, T. Ghosh, J. Hay, F. Isla, B. Marzeion, B. Meyssignac, and Z. Sebesvari. The Ocean and Cryosphere in a Changing Climate: Special Report of the Intergovernmental Panel on

Climate Change. Cambridge University Press, Cambridge, UK and New York, NY, USA: Cambridge University Press, 2019.

184 Slater T, Hogg AE, Mottram R. Ice-sheet losses track high-end sea-level rise projections. *Nat Clim Chang* 2020; **10**: 879–81.

185 Mukherji A, Thorne P, Cheung WWL, *et al.* AR6 Synthesis Report: Climate Change 2023. *IPCC* 2023. https://report.ipcc.ch/ar6syr/pdf/IPCC_AR6_SYR_SPM.pdf.

186 Thomas MA, Lin T. Illustrative Analysis of Probabilistic Sea Level Rise Hazard. *Journal of Climate* 2020; **33**: 1523–34.

187 McMichael C, Dasgupta S, Ayeb-Karlsson S, Kelman I. A review of estimating population exposure to sea-level rise and the relevance for migration. *Environ Res Lett* 2020; **15**: 123005.

188 Ayeb-Karlsson S, Kniveton D, Cannon T. Trapped in the prison of the mind: Notions of climate-induced (im)mobility decision-making and wellbeing from an urban informal settlement in Bangladesh. *Palgrave Commun* 2020; **6**: 1–15.

189 Duijndam SJ, Botzen WJW, Hagedoorn LC, Aerts JCJH. Anticipating sea-level rise and human migration: A review of empirical evidence and avenues for future research. *WIREs Climate Change* 2022; **13**: e747.

190 Hauer ME, Fussell E, Mueller V, *et al.* Sea-level rise and human migration. *Nat Rev Earth Environ* 2020; **1**: 28–39.

191 Lassiter A. Rising seas, changing salt lines, and drinking water salinization. *Current Opinion in Environmental Sustainability* 2021; **50**: 208–14.

192 Dannenberg AL, Frumkin H, Hess JJ, Ebi KL. Managed retreat as a strategy for climate change adaptation in small communities: public health implications. *Climatic Change* 2019; **153**: 1–14.

193 McMichael C, Powell T. Planned Relocation and Health: A Case Study from Fiji. *Int J Environ Res Public Health* 2021; **18**: 4355.

194 Liu Y, Zhu J, Shao X, Adusumilli NC, Wang F. Diffusion patterns in disaster-induced internet public opinion: based on a Sina Weibo online discussion about the 'Liangshan fire' in China. *Environmental Hazards* 2021; **20**: 163–87.

195 Romanello M, McGushin A, Napoli CD, *et al.* The 2021 report of the Lancet Countdown on health and climate change: code red for a healthy future. *The Lancet* 2021; **398**: 1619–62.

196 Watts N, Amann M, Arnell N, *et al.* The 2019 report of The Lancet Countdown on health and climate change: ensuring that the health of a child born today is not defined by a changing climate. *The Lancet* 2019; **394**: 1836–78.

197 Watts N, Amann M, Ayeb-Karlsson S, *et al.* The Lancet Countdown on health and climate change: from 25 years of inaction to a global transformation for public health. *The Lancet* 2018; **391**: 581–630.

198 Kelman I. Imaginary Numbers of Climate Change Migrants? *Social Sciences* 2019; **8**: 131.

199 Cissé, G., R. McLeman, H. Adams, P. et al. 2022: Health, Wellbeing, and the Changing Structure of Communities. In: Climate Change 2022: Impacts, Adaptation and Vulnerability. Contribution of Working Group II to the Sixth Assessment Report of the Intergovernmental Panel on Climate Change [H.-O. Pörtner, D.C. Roberts, M. Tignor, E.S. Poloczanska, K. Mintenbeck, A. Alegría, M. Craig, S. Langsdorf, S. Löschke, V. Möller, A. Okem, B. Rama (eds.)]. Cambridge University Press, Cambridge, UK and New York, NY, USA, pp. 1041–1170, doi:10.1017/9781009325844.009. .

200 IEA. CO2 Emissions from Fuel Combustion (2022 edition). 2022.

201 IEA. CO2 Emissions in 2022 – Analysis. Paris: IEA, 2023.

202 IEA. CO2 Emissions From Fuel Combustion: Database Documentation (2020 Edition). 2020.

203 IEA. World Extended Energy Balances (2022 edition). 2022.

204 IEA. World Energy Balances: Data Documentation (2020 edition). 2020.

205 IEA. Methodology. Defining energy access. 2020.

206 WHO. Global database of household air pollution measurements. Geneva: WHO, 2018 https://www.who.int/data/gho/data/themes/air-pollution/hap-measurement-db.

207 Stoner O, Shaddick G, Economou T, *et al.* Global household energy model: a multivariate hierarchical approach to estimating trends in the use of polluting and clean fuels for cooking. *Journal of the Royal Statistical Society: Series C (Applied Statistics)* 2020; **69**: 815–39.

208 UNDESA. World Population Prospects 2019. 2021; published online April 21.

209 Woodcock J, Givoni M, Morgan AS. Health impact modelling of active travel visions for England and Wales using an Integrated Transport and Health Impact Modelling Tool (ITHIM). *PLoS One* 2013; **8**: e51462.

210 Kole PJ, Löhr AJ, Van Belleghem FGAJ, Ragas AMJ. Wear and Tear of Tyres: A Stealthy Source of Microplastics in the Environment. *Int J Environ Res Public Health* 2017; **14**: 1265.

211 Amann M, Bertok I, Borken-Kleefeld J, *et al.* Cost-effective control of air quality and greenhouse gases in Europe: Modeling and policy applications. *Environmental Modelling & Software* 2011; **26**: 1489–501.

212 IEA. World Energy Outlook 2021. Paris: IEA, 2021 https://www.iea.org/reports/world-energy-outlook-2021 (accessed June 16, 2022).

213 IEA. World Energy Outlook 2022. Paris: IEA, 2023 https://www.iea.org/news/world-energy-outlook-2022-shows-the-global-energy-crisis-can-be-a-historic-turning-point-towards-a-cleaner-and-more-secure-future.

214 Simpson D, Benedictow A, Berge H, *et al.* The EMEP MSC-W chemical transport model – technical description. *Atmospheric Chemistry and Physics* 2012; **12**: 7825–65.

215 Kiesewetter G, Borken-Kleefeld J, Schöpp W, *et al.* Modelling street level PM 10 concentrations across Europe: source apportionment and possible futures. *Atmospheric Chemistry and Physics* 2015; **15**: 1539–53.

216 Markus Amann, Gregor Kiesewetter, Wolfgang Schöpp, *et al.* Reducing global air pollution: the scope for further policy interventions. *Phil Trans R Soc A* 2020; **378**.

217 WHO. WHO Global Urban Ambient Air Pollution Database (update 2018). 2018.

218 GBD 2019 Risk Factors Collaborators. Global burden of 87 risk factors in 204 countries and territories, 1990-2019: a systematic analysis for the Global Burden of Disease Study 2019. *Lancet* 2020; **396**: 1223–49.

219 Global Burden of Disease Collaborative Network. Particulate Matter Risk Curves. Seattle: Institute for Health Metrics and Evaluation (IHME), 2021.

220 Forouzanfar MH, Alexander L, Anderson HR, *et al.* Global, regional, and national comparative risk assessment of 79 behavioural, environmental and occupational, and metabolic risks or clusters of risks in 188 countries, 1990–2013: a systematic analysis for the Global Burden of Disease Study 2013. *The Lancet* 2015; **386**: 2287–323.

221 IHME. GBD Results Tool. 2019.

222 UNDESA. World Population Prospects: The 2017 revision. 2017.

223 Chen J, Hoek G. Long-term exposure to PM and all-cause and cause-specific mortality: A systematic review and meta-analysis. *Environ Int* 2020; **143**: 105974.

224 IEA. World Extended Energy Balances (2021 edition). Paris: IEA, 2021.

225 Alexandratos N. World Agriculture towards 2030/2050: the 2012 revision. .

226 IFA. International Fertiziler Association Database. Paris: IFA, 2022.

227 Shupler M, Godwin W, Frostad J, Gustafson P, Arku RE, Brauer M. Global estimation of exposure to fine particulate matter (PM2.5) from household air pollution. *Environment International* 2018; **120**: 354–63.

228 Smith KR, Bruce N, Balakrishnan K, *et al.* Millions Dead: How Do We Know and What Does It Mean? Appendix. *Annu Rev Public Health* 2014; **35**: 185–206.

229 WHO. Household Air Pollution and Health. Geneva: WHO, 2022 https://www.who.int/news-room/fact-sheets/detail/household-air-pollution-and-health.

230 Klimont Z, Kupiainen K, Heyes C, *et al.* Global anthropogenic emissions of particulate matter including black carbon. *Atmospheric Chemistry and Physics* 2017; **17**: 8681–723.

231 Yip W, NASA. Global Daily Downscaled Projections (NEX-GDDP-CMIP6). 2021 http://www.nasa.gov/nex/gddp (accessed April 8, 2023).

232 WHO. Indicator 7.1.2: Proportion of population with primary reliance on clean fuels and technology. 2016; published online July 19.

233 Du W, Wang J, Wang Z, *et al.* Influence of COVID-19 lockdown overlapping Chinese Spring Festival on household PM2.5 in rural Chinese homes. *Chemosphere* 2021; **278**: 130406.

234 Shen H, Shen G, Chen Y, *et al.* Increased air pollution exposure among the Chinese population during the national quarantine in 2020. *Nature Human Behaviour* 2021; **5**: 239–46.

235 Dalin C, Tuninetti M, Carlson K, *et al.* Variability, drivers and interactions of key environmental stressors from food production worldwide. 21st EGU General Assembly, 2019.

236 Herrero M, Havlik P, Valin H, *et al.* Biomass use, production, feed efficiencies, and greenhouse gas emissions from global livestock systems. *Proceedings of the National Academy of Sciences* 2013; **110**: 20888–93.

237 Havlík P, Valin H, Herrero M, *et al.* Climate change mitigation through livestock system transitions. *Proceedings of the National Academy of Sciences* 2014; **111**: 3709–14.

238 Cecile De Klein, Rafael S.A. Novoa, Stephen Ogle, Keith A. Smith,, *et al.* IPCC Guidelines for National Greenhouse Gas Inventories. Agriculture, Forestry and Other Land Use. Hayama: Institute for Global Environmental Strategies, 2006.

239 FAO. FAOSTAT. 2023.

240 Chang J, Ciais P, Herrero M, *et al.* Combining livestock production information in a process-based vegetation model to reconstruct the history of grassland management. 2016; : 21.

241 Carlson KM, Gerber JS, Mueller ND, *et al.* Greenhouse gas emissions intensity of global croplands. *Nature Clim Change* 2017; **7**: 63–8.

242 Dalin C, Wada Y, Kastner T, Puma MJ. Groundwater depletion embedded in international food trade. *Nature* 2017; **543**: 700–4.

243 Kastner T, Kastner M, Nonhebel S. Tracing distant environmental impacts of agricultural products from a consumer perspective. *Ecological Economics* 2011; **70**: 1032–40.

244 Poore J, Nemecek T. Reducing food's environmental impacts through producers and consumers. *Science* 2018; **360**: 987–92.

245 Mbow C, Rosenzweig C. Chapter 5 : Food Security — Special Report on Climate Change and Land. IPCC https://www.ipcc.ch/srccl/chapter/chapter-5/ (accessed March 31, 2022).

246 Chen C, Chaudhary A, Mathys A. Nutritional and environmental losses embedded in global food waste. *Resources, Conservation and Recycling* 2020; **160**: 104912.

247 Miller V, Singh GM, Onopa J, *et al.* Global Dietary Database 2017: data availability and gaps on 54 major foods, beverages and nutrients among 5.6 million children and adults from 1220 surveys worldwide. *BMJ Global Health* 2021; **6**: e003585.

248 Gobbo DLC, Khatibzadeh S, Imamura F, *et al.* Assessing global dietary habits: a comparison of national estimates from the FAO and the Global Dietary Database. *The American Journal of Clinical Nutrition* 2015; **101**: 1038–46.

249 Micha R, Khatibzadeh S, Shi P, *et al.* Global, regional and national consumption of major food groups in 1990 and 2010: a systematic analysis including 266 country-specific nutrition surveys worldwide. *BMJ Open* 2015; **5**: e008705.

250 Rennie KL, Coward A, Jebb SA. Estimating under-reporting of energy intake in dietary surveys using an individualised method. *British Journal of Nutrition* 2007; **97**: 1169–76.

251 Freedman LS, Commins JM, Moler JE, *et al.* Pooled results from 5 validation studies of dietary self-report instruments using recovery biomarkers for energy and protein intake. *American journal of epidemiology* 2014; **180**: 172–88.

252 N. C. D. Risk Factor Collaboration. Trends in adult body-mass index in 200 countries from 1975 to 2014: a pooled analysis of 1698 population-based measurement studies with 19·2 million participants. *The Lancet* 2016; **387**: 1377–96.

253 Food and Agriculture Organization of the United Nations. Food balance sheets: a handbook. Rome: FAO, 2001.

254 Gustavsson J, Cederberg C, Sonesson U, Van Otterdijk R, Meybeck A. Global food losses and food waste: extent, causes and prevention. 2011.

255 Murray CJL, Ezzati M, Lopez AD, Rodgers A, Vander Hoorn S. Comparative quantification of health risks: conceptual framework and methodological issues. *Population Health Metrics* 2003; **1**: 1.

256 Lim SS, Vos T, Flaxman AD, *et al.* A comparative risk assessment of burden of disease and injury attributable to 67 risk factors and risk factor clusters in 21 regions, 1990–2010: a systematic analysis for the Global Burden of Disease Study 2010. *The Lancet* 2012; **380**: 2224–60.

257 Forouzanfar MH, Afshin A, Alexander LT, *et al.* Global, regional, and national comparative risk assessment of 79 behavioural, environmental and occupational, and metabolic risks or clusters of risks, 1990–2015: a systematic analysis for the Global Burden of Disease Study 2015. *The Lancet* 2016; **388**: 1659–724.

258 Murray CJL, Ezzati M, Flaxman AD, *et al.* GBD 2010: design, definitions, and metrics. *Lancet* 2012; **380**: 2063–6.

259 Wang H, Abbas KM, Abbasifard M, *et al.* Global age-sex-specific fertility, mortality, healthy life expectancy (HALE), and population estimates in 204 countries and territories, 1950–2019: a comprehensive demographic analysis for the Global Burden of Disease Study 2019. *The Lancet* 2020; **396**: 1160–203.

260 Afshin A, Micha R, Khatibzadeh S, Mozaffarian D. Consumption of nuts and legumes and risk of incident ischemic heart disease, stroke, and diabetes: a systematic review and meta-analysis. *The American Journal of Clinical Nutrition* 2014; **100**: 278–88.

261 Singh GM, Danaei G, Farzadfar F, *et al.* The Age-Specific Quantitative Effects of Metabolic Risk Factors on Cardiovascular Diseases and Diabetes: A Pooled Analysis. *PLoS One* 2013; **8**: e65174.

262 Micha R, Shulkin ML, Peñalvo JL, *et al.* Etiologic effects and optimal intakes of foods and nutrients for risk of cardiovascular diseases and diabetes: Systematic reviews and meta-analyses from the Nutrition and Chronic Diseases Expert Group (NutriCoDE). *PLoS One* 2017; **12**: e0175149.

263 Aune D, Giovannucci E, Boffetta P, *et al.* Fruit and vegetable intake and the risk of cardiovascular disease, total cancer and all-cause mortality—a systematic review and dose-response meta-analysis of prospective studies. *International Journal of Epidemiology* 2017; **46**: 1029–56.

264 Bechthold A, Boeing H, Schwedhelm C, *et al.* Food groups and risk of coronary heart disease, stroke and heart failure: A systematic review and dose-response meta-analysis of prospective studies. *Critical Reviews in Food Science and Nutrition* 2019; **59**: 1071–90.

265 Schwingshackl L, Knüppel S, Schwedhelm C, *et al.* Perspective: NutriGrade: A Scoring System to Assess and Judge the Meta-Evidence of Randomized Controlled Trials and Cohort Studies in Nutrition Research. *Advances in Nutrition: An International Review Journal* 2016; **7**: 994–1004.

266 Schwingshackl L, Hoffmann G, Lampousi AM, *et al.* Food groups and risk of type 2 diabetes mellitus: a systematic review and meta-analysis of prospective studies. *European Journal of Epidemiology* 2017; **32**: 363–75.

267 Schwingshackl L, Knüppel S, Michels N, *et al.* Intake of 12 food groups and disability-adjusted life years from coronary heart disease, stroke, type 2 diabetes, and colorectal cancer in 16 European countries. *European Journal of Epidemiology* 2019; **34**: 765–75.

268 World Cancer Research Fund/American Institute for Cancer R. Diet, Nutrition, Physical Activity and Cancer: A Global Perspective. Continuous Update Project Expert Report. 2018.

269 Aune D, Lau R, Chan DSM, *et al.* Dairy products and colorectal cancer risk: a systematic review and meta-analysis of cohort studies. *Annals of Oncology: Official Journal of the European Society for Medical Oncology* 2012; **23**: 37–45.

270 Aune D, Norat T, Romundstad P, Vatten LJ. Dairy products and the risk of type 2 diabetes: a systematic review and dose-response meta-analysis of cohort studies. *The American Journal of Clinical Nutrition* 2013; **98**: 1066–83.

271 Mohan D, Mente A, Dehghan M, *et al.* Associations of Fish Consumption With Risk of Cardiovascular Disease and Mortality Among Individuals With or Without Vascular Disease From 58 Countries. *JAMA Internal Medicine* 2021; published online March 8. DOI:10.1001/jamainternmed.2021.0036.

272 Institute of Medicine. Dietary Reference Intakes for Energy, Carbohydrate, Fiber, Fat, Fatty Acids, Cholesterol, Protein, and Amino Acids. Washington, DC: Institute of Medicine., 2005.

273 Aune D, Keum N, Giovannucci E, *et al.* Nut consumption and risk of cardiovascular disease, total cancer, all-cause and cause-specific mortality: a systematic review and dose-response meta-analysis of prospective studies. *BMC medicine* 2016; **14**: 207.

274 Aune D, Keum N, Giovannucci E, *et al.* Whole grain consumption and risk of cardiovascular disease, cancer, and all cause and cause specific mortality: systematic review and dose-response meta-analysis of prospective studies. *BMJ (Clinical research ed)* 2016; **353**: i2716.

275 Global Bmi Mortality Collaboration DE, Di Angelantonio E, Bhupathiraju S, *et al.* Body-mass index and all-cause mortality: individual-participant-data meta-analysis of 239 prospective studies in four continents. *Lancet (London, England)* 2016; **388**: 776–86.

276 Satija A, Yu E, Willett WC, Hu FB. Understanding Nutritional Epidemiology and Its Role in Policy. *Advances in Nutrition* 2015; **6**: 5–18.

277 Prospective Studies Collaboration, Whitlock G, Lewington S, *et al.* Body-mass index and cause-specific mortality in 900 000 adults: collaborative analyses of 57 prospective studies. *Lancet* 2009; **373**: 1083–96.

278 Zheng J, Huang T, Yu Y, Hu X, Yang B, Li D. Fish consumption and CHD mortality: an updated meta-analysis of seventeen cohort studies. *Public Health Nutrition* 2012; **15**: 725–37.

279 Schwingshackl L, Hoffmann G, … KI… A, Undefined. Food groups and intermediate disease markers: a systematic review and network meta-analysis of randomized trials. *American Journal of Clinical Nutrition* 2018; **108**: 576–86.

280 Xun P, Qin B, Song Y, *et al.* Fish consumption and risk of stroke and its subtypes: accumulative evidence from a meta-analysis of prospective cohort studies. *European Journal of Clinical Nutrition* 2012; **66**: 1199–207.

281 Miller RE, Blair PD. Input-Output Analysis: Foundations and Extensions, 2nd edn. Cambridge: Cambridge University Press, 2009 DOI:10.1017/CBO9780511626982.

282 WHO. Global Health Expenditure Database: Indicators and data. 2021.

283 Swiss Re. Sigma explorer. 2023; published online April 11.

284 IMF. World Economic Outlook Database. 2022. https://www.imf.org/en/Publications/SPROLLs/world-economic-outlook-databases (accessed Feb 10, 2023).

285 Aldy JE, Viscusi WK. Age Differences in the Value of Statistical Life: Revealed Preference Evidence. *Review of Environmental Economics and Policy* 2007; **1**: 241–60.

286 Robinson LA, Sullivan R, Shogren JF. Do the Benefits of COVID-19 Policies Exceed the Costs? Exploring Uncertainties in the Age–VSL Relationship. *Risk Analysis* 2020.

287 World Bank Group. Population, total,. Washington, DC, USA, 2022.

288 OECD. Gross domestic product (GDP) : GDP per head, US $, constant prices, constant PPPs, reference year 2015, millions. 2021.

289 OECD. Mortality Risk Valuation in Environment, Health and Transport Policies. 2012.

290 WHO. WHO methods and data sources for global burden of disease estimates 2000-2015. 2017.

291 World Bank Group. GDP per capita (current USD). Washington DC, USA, 2022.

292 World Bank Group. inflation rate. Washington DC, USA, 2022.

293 ILO. ILOSTAT: Concepts and Definitions. 2023. https://ilostat.ilo.org/resources/concepts-and-definitions/ (accessed April 1, 2023).

294 International Labour Organization. Wages and Working Time Statistics (COND database). https://ilostat.ilo.org/resources/concepts-and-definitions/description-wages-and-working-time-statistics/ (accessed May 17, 2023).

295 ILO. ILOSTAT Statistics on wages. 2023.

296 IMF. International Finance Statistics. 2023.

297 World Bank. Country and Lending Groups. 2022; published online July 1. https://datahelpdesk.worldbank.org/knowledgebase/articles/906519 (accessed April 1, 2023).

298 WHO. Wolrd Health Organisation Countries Listing. 2021; published online April 21. https://www.who.int/countries (accessed April 3, 2023).

299 UNDP (United Nations Development Programme). Human Development Report 2021-22. *UNDP (United Nations Development Programme)* 2022. http://report.hdr.undp.org,.

300 UNDESA. World Population Prospects. 2023. https://population.un.org/dataportal/home (accessed March 22, 2023).

301 International Energy Agency (IEA). World Energy Investment 2023. France: IEA, 2023.

302 IRENA and ILO. Renewable Energy and Jobs: Annual Review 2022. Abu Dhabi (IRENA), Geneva (ILO), 2022 https://www.irena.org/publications/2022/Sep/Renewable-Energy-and-Jobs-Annual-Review-2022.

303 Gonzales, Eddie. Global Coal Mining Industry Report. IBISWorld, 2022 https://my.ibisworld.com/gl/en/industry/b0511-gl.

304 Buchko M. Global Oil & Gas Exploration & Production Industry Report. IBISWorld, 2022 https://my.ibisworld.com/gl/en/industry/b0531-gl.

305 Stand. earth. Global Fossil Fuel Commitments Database. 2022.

306 IEA. Fossil Fuel Subsidies Database. Paris: International Energy Agency, 2022 https://www.iea.org/data-and-statistics/data-product/fossil-fuel-subsidies-database (accessed April 3, 2023).

307 IEA. Energy subsidies. Methodology and assumptions - the price-gap approach. 2023. https://www.iea.org/topics/energy-subsidies (accessed April 3, 2023).

308 OECD. OECD Inventory of support measures for fossil fuels. 2022. https://stats.oecd.org/Index.aspx?DataSetCode=FFS_AUS (accessed Feb 9, 2023).

309 OECD. OECD Companion to the Inventory of Support Measures for Fossil Fuels 2018. Paris, France: OECD Publishing, 2018.

310 World Bank. World Bank Carbon Pricing Dashboard. 2022. https://carbonpricingdashboard.worldbank.org/ (accessed Jan 13, 2023).

311 IEA. International Energy Agency Greenhouse Gas Emissions from Energy, 1751-2021. [data collection], 13th edn. UK Data Service. SN 5181, 2022 DOI: 10.5257/iea/co2/2022 (accessed Feb 2, 2023).

312 European Commision. Auctioning. 2022. https://climate.ec.europa.eu/eu-action/eu-emissions-trading-system-eu-ets/auctioning_en (accessed April 3, 2023).

313 WHO. World Health Organization Global Health Expenditure Database. 2022. https://apps.who.int/nha/database/Select/Indicators/en (accessed Feb 14, 2023).

314 Mi Z, Zheng J, Meng J, *et al.* Economic development and converging household carbon footprints in China. *Nature Sustainability* 2020; **3**: 529–37.

315 Stadler K, Wood R, Bulavskaya T, *et al.* EXIOBASE 3: Developing a Time Series of Detailed Environmentally Extended Multi-Regional Input-Output Tables. *Journal of Industrial Ecology* 2018; **22**: 502–15.

316 Friedlingstein P, Jones MW, O'Sullivan M, *et al.* Global Carbon Budget 2021. *Earth System Science Data Discussions* 2021; **2021**: 1–191.

317 World Bank. World Bank Open Data. 2023.

318 WTO. WTO STATS. 2023.

319 Crippa M, Oreggioni G, Guizzardi D, *et al.* Fossil CO2 and GHG emissions of all world countries. Luxembourg (Luxembourg): Publications Office of the European Union, 2019.

320 P. R. Shukla JS. IPCC, 2022: Climate Change 2022: Mitigation of Climate Change. Contribution of Working Group III to the Sixth Assessment Report of the Intergovernmental Panel on Climate Change. Cambridge, UK and New York, NY, USA, 2022.

321 TPI. Transition Pathway Initiative: Oil & Gas Sector. 2022. https://www.transitionpathwayinitiative.org/tpi/sectors/oil-gas%0A (accessed April 12, 2022).

322 SBTi. The Science Based Targets initiative (SBTi): About Us. 2022. https://sciencebasedtargets.org/about-us.

323 SEI, IISD, ODI, E3G, UNEP. The Production Gap Report 2021. 2021 https://productiongap.org/2021report/.

324 Climate Action. Net Zero Company Benchmark. 2022.

325 CTI. Adapt to Survive: Why oil companies must plan for net zero and avoid stranded assets. Carbon Tracker Initiaitve, 2021 https://carbontracker.org/reports/adapt-to-survive/%0A.

326 OCI. Big Oil Reality Check: Assessing Oil And Gas Company Climate Plans. Washington DC: Oil Change International, 2020 https://priceofoil.org/2020/09/23/big-oil-reality-check/.

327 Li M, Trencher G, Asuka J. The clean energy claims of BP, Chevron, ExxonMobil and Shell: A mismatch between discourse, actions and investments. *PLoS ONE* 2022; **17**: e0263596.

328 SBTi. Sectoral Decarbonization Approach (SDA): A method for setting corporate emission reduction targets in line with climate science. Science-Based Targets Initiative, 2015 https://sciencebasedtargets.org/resources/files/Sectoral-Decarbonization-Approach-Report.pdf.

329 Krabbe O, Linthorst G, Blok K, *et al.* Aligning corporate greenhouse-gas emissions targets with climate goals. *Nature Climate Change* 2015; **5**: 1057–60.

330 SBTi. Value Change in the Value Chain: Best Practices In Scope 3 Greenhouse Gas Management. Science-Based Targets Initiative, 2018 https://sciencebasedtargets.org/resources/legacy/2018/12/SBT_Value_Chain_Report-1.pdf.

331 World Resources I, Laurent Corbier World Business Council for Sustainable D. Greenhouse Gas Protocol: a Corporate Accounting and Reporting Standard. 2004.

332 H. M. Government. Environmental reporting guidelines: including Streamlined Energy and Carbon Reporting requirements. London, UK, 2019.

333 CDP. CDP S&P 500 Climate Change Report 2013. 2013 https://www.cdp.net/en/reports/downloads/626.

334 CDP. Now For Nature: The Decade of Delivery. CDP Europe Report 2021. Berlin, Germany, 2021 https://www.cdp.net/en/research/cdp-europe-reports/now-for-nature.

335 Dietz S, Gardiner D, Hastreiter N, Jahn V, Noels J. Carbon Performance assessment of oil & gas producers: note on methodology. London, 2021.

336 SBTi. What is the SBTi's policy on fossil fuel companies? 2022. https://sciencebasedtargets.org/sectors/oil-and-gas#what-is-the-sb-tis-policy-on-fossil-fuel-companies.

337 Rogelj J, Shindell D, Jiang K, *et al.* Mitigation Pathways Compatible with 1.5°C in the Context of Sustainable Development. In: Masson-Delmotte V, Zhai P, Pörtner HO, *et al.*, eds. Global Warming of 1.5°C. An IPCC Special Report on the impacts of global warming of 1.5°C above pre-industrial levels and related global greenhouse gas emission pathways, in the context of strengthening the global response to the threat of climate change. 2018.

338 SBTi. Foundations of Science-based Target Setting. Science-Based Targets Initiative, 2019 https://sciencebasedtargets.org/resources/legacy/2019/04/foundations-of-SBT-setting.pdf.

339 Huppmann D, Kriegler E, Krey V, *et al.* IAMC 1.5°C Scenario Explorer and Data hosted by IIASA. 2019; published online Aug 8. DOI:10.5281/ZENODO.3363345.

340 IEA. World Energy Outlook 2021. Paris, France: International Energy Agency, 2021 https://www.iea.org/reports/world-energy-outlook-2021.

341 IEA. World Energy Outlook 2022. Paris, France: International Energy Agency, 2022 https://www.iea.org/reports/world-energy-outlook-2022.

342 Chopra P, Karagiannopoulos L. Big Oil faces major reserves challenge as new discoveries fail to replace production. Rystad Energy. 2021; published online May 5. https://www.rystadenergy.com/newsevents/news/press-releases/big-oil-faces-major-reserves-challenge-as-new-discoveries-fail-to-replace-production/.

343 Rystad Energy. UCube Database. 2023. https://www.rystadenergy.com/services/upstream-solution (accessed Feb 24, 2023).

344 IEA. World Energy Balances. Paris: International Energy Agency, 2022 https://www.iea.org/data-and-statistics/data-product/world-energy-balances-highlights (accessed March 9, 2023).

345 United Nations Environment Finance Initiative. Net-Zero Banking Alliance: Members. 2023. https://www.unepfi.org/net-zero-banking/members/ (accessed April 4, 2023).

346 Giles J. Special Report Internet encyclopaedias go head to head. *Nature* 2005; **438**: 900–1.

347 Similarweb. Top websites ranking. Most visited websites in the world. 2023. https://www.similarweb.com/top-websites/. (accessed March 1, 2023).

348 Smith DA. Situating Wikipedia as a health information resource in various contexts: A scoping review. *PloS one* 2020; **15**: e0228786.

349 Wikimedia. Wikipedia clickstream. https://meta.wikimedia.org/wiki/Research:Wikipedia_clickstream (accessed March 1, 2023).

350 Berrang-Ford L, Sietsma AJ, Callaghan M, *et al.* Systematic mapping of global research on climate and health: a machine learning review. *The Lancet Planetary Health* 2021; **5**: e514–25.

351 Chang C-C, Lin C-J. LIBSVM: A Library for Support Vector Machines. *ACM Trans Intell Syst Technol* 2011; **2**. DOI:10.1145/1961189.1961199.

352 Halterman A. Mordecai: Full Text Geoparsing and Event Geocoding. *J Open Source Softw* 2017; **2**: 91.

353 Ebi KL, Ogden NH, Semenza JC, Woodward A. Detecting and Attributing Health Burdens to Climate Change. *Environ Health Perspect* 2017; **125**: 085004.

354 Bindoff, N.L., P.A. Stott, K.M. AchutaRao, M.R. Allen, N. Gillett, D. Gutzler, K. Hansingo, G. Hegerl, Y. Hu, S. Jain, I.I., Mokhov, J. Overland, J. Perlwitz, R. Sebbari and X. Zhang, Intergovernmental Panel on Climate Change. Detection and Attribution of Climate Change: from Global to Regional. In: Climate Change 2013 – The Physical Science Basis, 1st edn. Cambridge, United Kingdom and New York, NY, USA: Cambridge University Press, 2014: 867–952.

355 Campbell-Lendrum DH, Woodruff R, Prüss-Üstün A, Corvalán CF, Organization WH. Climate change : quantifying the health impact at national and local levels. World Health Organization, 2007 https://apps.who.int/iris/handle/10665/43708 (accessed April 4, 2023).

356 Callaghan M, Schleussner C-F, Nath S, *et al.* Machine-learning-based evidence and attribution mapping of 100,000 climate impact studies. *Nature climate change* 2021; **11**: 966–72.

357 Bergstra J, Bardenet R, Bengio Y, Kégl B. Algorithms for hyper-parameter optimization. *Advances in neural information processing systems* 2011; **24**.

358 Knutson TR, Zeng F, Wittenberg AT. Multimodel assessment of regional surface temperature trends: CMIP3 and CMIP5 twentieth-century simulations. *Journal of Climate* 2013; **26**: 8709–43.

359 Knutson TR, Zeng F. Model assessment of observed precipitation trends over land regions: detectable human influences and possible low bias in model trends. *Journal of Climate* 2018; **31**: 4617–37.

360 Benoit K, Watanabe K, Wang H, *et al.* quanteda: An R package for the quantitative analysis of textual data. *Journal of Open Source Software*; **3**: 774.

361 Leeper TJ. tabulizer: Bindings for tabula PDF table extractor library. *R package version 02* 2018; **2**.

362 Karlsson M, Alfredsson E, Westling N. Climate policy co-benefits: a review. *Climate Policy* 2020; **20**: 292–316.

363 Workman A, Blashki G, Bowen KJ, Karoly DJ, Wiseman J. The political economy of health co-benefits: embedding health in the climate change agenda. *International journal of environmental research and public health* 2018; **15**: 674.

364 Romanello M, van Daalen K, Anto JM, *et al.* Tracking progress on health and climate change in Europe. *The Lancet Public Health* 2021; **6**: e858–65.

365 Kural E, Dellmuth LM, Gustafsson M-T. International organizations and climate change adaptation: A new dataset for the social scientific study of adaptation, 1990–2017. *Plos one* 2021; **16**: e0257101.

366 Blei DM. Probabilistic topic models. *Communications of the ACM* 2012; **55**: 77–84.

367 Roberts ME, Stewart BM, Tingley D, *et al.* Structural topic models for open-ended survey responses. *American journal of political science* 2014; **58**: 1064–82.

368 Roberts ME, Stewart BM, Tingley D. Stm: An R package for structural topic models. *Journal of Statistical Software* 2019; **91**: 1–40.
